# Genomic and Metabolic Studies of the Impact of Probiotics on a Model Gut Symbiont and Host

**DOI:** 10.1371/journal.pbio.0040413

**Published:** 2006-11-28

**Authors:** Justin L Sonnenburg, Christina T. L Chen, Jeffrey I Gordon

**Affiliations:** Center for Genome Sciences, Washington University School of Medicine, St. Louis, Missouri, United States of America; University of California Davis Genome Center, United States of America

## Abstract

Probiotics are deliberately ingested preparations of live bacterial species that confer health benefits on the host. Many of these species are associated with the fermentation of dairy products. Despite their increasing use, the molecular details of the impact of various probiotic preparations on resident members of the gut microbiota and the host are generally lacking. To address this issue, we colonized germ-free mice with *Bacteroides thetaiotaomicron,* a prominent component of the adult human gut microbiota, and *Bifidobacterium longum,* a minor member but a commonly used probiotic. Simultaneous whole genome transcriptional profiling of both bacterial species in their gut habitat and of the intestinal epithelium, combined with mass-spectrometric analysis of habitat-associated carbohydrates, revealed that the presence of B. longum elicits an expansion in the diversity of polysaccharides targeted for degradation by B. thetaiotaomicron (e.g., mannose- and xylose-containing glycans), and induces host genes involved in innate immunity. Although the overall transcriptome expressed by B. thetaiotaomicron when it encounters B. longum in the cecum is dependent upon the genetic background of the mouse (as assessed by a mixed analysis of variance [ANOVA] model of co-colonization experiments performed in NMRI and C57BL/6J animals), B. thetaiotaomicron's expanded capacity to utilize polysaccharides occurs independently of host genotype, and is also observed with a fermented dairy product-associated strain, *Lactobacillus casei*. This gnotobiotic mouse model provides a controlled case study of how a resident symbiont and a probiotic species adapt their substrate utilization in response to one another, and illustrates both the generality and specificity of the relationship between a host, a component of its microbiota, and intentionally consumed microbial species.

## Abstract 2

A gnotobiotic mouse model enabled transcriptional profiling of both host and bacteria. This provides insights into the intestinal response to colonization and reveals how all organisms involved adapt and respond to one another.

## Introduction

Over the millennia, our species has evolved to cope with, and perhaps benefit from, consuming microbes present in decomposing food. Some have postulated that ingestion of food-borne microbial species was common in the Pleistocene diet of hominins practicing subterranean food storage [1,2]. Eventually these microbes were co-opted by humans to transform foods via fermentation [3]. During the last century, technological improvements, such as refrigeration and pasteurization, have resulted in dramatic decreases in consumption of food-borne microbes in many countries. This sanitization has coincided with increased prevalence of a number of disorders, both within and outside of the gastrointestinal tract, including inflammatory bowel disease and atopic states (e.g., asthma and food allergies). Although links between certain “western” diseases and decreased exposure to microbes are being considered at the present time (e.g., the hygiene hypothesis in the case of atopic disorders) [4], the positive health effects of consuming fermented foods were noted more than 100 y ago when Elie Metchnikoff correlated their ingestion with increased life span in Bulgarian peasants [5]. More recently, the effects of consuming live microbial supplements with presumptive health benefits on human physiology have become the subject of various clinical studies (reviewed in [6,7]). However, the impact of these “probiotic” species on the composition and functioning of the human gut microbiota, the degree to which their effects are mediated through the resident microbiota versus direct signaling to the host, and the impact of host genotype on probiotic responses remain open questions. The answers have important implications for designing and interpreting clinical trials that explore the specific and general effects of different probiotics, and whether their consumption should be dictated by the microbial ecology or other properties of the host, including diet and genotype.

The task of determining the mechanism of action of probiotics in humans is daunting. Our intestines contain 10–100 trillion microbes. A recent comprehensive 16S rRNA sequence-based enumeration has provided detailed snapshots of the composition of this community in a few healthy individuals [8]. Our 21st century microbiota contains members of at least nine of the 70 described divisions of Bacteria [9], although it is dominated by just two: the Firmicutes and the Bacteroidetes. One methanogenic archaeon, *Methanobrevibacter smithii,* which belongs to the Euryarchaea, is very prominent, comprising up to 10% of microbes in the colonic microbiota [10]. Variations in the microbial composition of the gut exist between individuals, primarily at the level of species and subspecies [8]. Moreover, within a given intestinal habitat (address), some microbial members appear to be entrenched “residents” (autochthonous components), whereas others act more like hitchhikers (allochthonous members) that pass through together with food, water, and other components of the environment [11].

These considerations emphasize the importance of creating simplified and defined model systems for studying the functional properties and operating principles of our gut microbial communities, and the consequences of adding probiotics. In this report, we colonize germ-free mice with Bacteroides thetaiotaomicron with or without *Bifidobacterium longum. B. thetaiotaomicron* was chosen because it is a prominent saccharolytic member of our distal gut microbiota (6% of all bacteria in the enumeration study described above) [8], that possesses the largest arsenal of glycoside hydrolases and polysaccharide lyases among sequenced human gut symbionts [12]. Thus, its highly evolved “glycobiome” helps us to process otherwise indigestible glycans in our diet. B. longum is a normal, albeit less prominent member of our microbiota [8,13] that belongs to the Actinobacteria and is commonly used as a probiotic [14]. Since both bacterial genomes have been sequenced, we were able to use a *B. thetaiotaomicron/B. longum* “community” GeneChip to simultaneously examine how each organism affects the other's transcriptome in a specified intestinal habitat of a host consuming a consistent and defined diet. Using a combination of in silico reconstructions of microbial metabolism based on these transcriptional profiles, mass spectroscopic analysis of glycan utilization, and whole genome transcriptional profiling of laser capture microdissected intestinal epithelium from germ-free and colonized mice, we were able to ascertain how a resident symbiont and probiotic species affect one another's carbohydrate substrate range and host biology. The generality of our findings was explored by testing the effect of two different host genetic backgrounds on the behavior of this model bi-component microbiota, and by comparing the responses of B. thetaiotaomicron to two other probiotic strains added to a variety of fermented milks: Bifidobacterium animalis and Lactobacillus casei.

## Results/Discussion

### Bi-association of Germ-Free Mice with B. thetaiotaomicron and B. longum

Three groups of 7-wk-old, germ-free male mice belonging to the NMRI inbred strain and maintained on a standard polysaccharide-rich chow diet, were colonized with a single gavage of 10^8^ colony forming units (CFU) of either B. thetaiotaomicron (ATCC 29148; isolated from the fecal microbiota of a healthy adult human) [15], B. longum (NCC2705; recovered from an infant) [14], or both bacterial species (*n* = 5 animals/group). Mice were sacrificed 10 d later, a period of time sufficient for the intestinal epithelium and its overlying mucus layer to turn over several times. The number of viable bacteria was determined in the cecum of each mouse at the time of their sacrifice. This gut habitat was chosen as the site for our analyses because it is a readily defined anatomic structure, interposed between the distal small intestine and colon, that normally houses a very dense and diverse microbiota (>10^12^ organisms/ml luminal contents; seven divisions of bacteria in a recent comprehensive 16S rRNA gene sequence-based survey) [16]. The cecal microbiota is sustained, at least in part, by its ability to access large quantities of plant-derived polysaccharides that are delivered from the small intestine in an undigested state because the mouse (and human) host lacks the requisite glycoside hydrolases to break them down (e.g., xylans, arabinogalactans, and polygalacturonates/pectins) [17,18].

B. thetaiotaomicron and B. longum colonized the cecum in a highly reproducible fashion. Moreover, microbial density was not significantly different for either species when they were introduced alone (10^10^–10^11^ CFU per milliliter of cecal contents; *p >* 0.05 according to Student *t*-test). Furthermore, the co-colonization (bi-association) experiment revealed that the two bacterial species can co-exist in the cecum without a statistically significant impact on one another's population size (unpublished data).

To gain a genome-wide view of the effects of co-colonization on each organism's transcriptome, RNA was isolated from the cecal contents of each animal in each treatment group. The resulting RNA preparations were highly enriched for microbial RNA (>90% as judged by quantitative reverse transcriptase PCR [qRT-PCR] assays of 16S and 18S rRNAs) [17]. Transcripts expressed in B. thetaiotaomicron and B. longum were profiled using a custom “community” GeneChip (Affymetrix, Santa Clara, California, United States) that contains oligonucleotides representing 4,719 of *B. thetaiotaomicron'*s 4,779 predicted chromosomal protein-coding genes (98.7%) [17] and 1,722 of *B. longum'*s 1,730 genes (99.5%). Each known or predicted gene in each genome was represented by an average of 13 perfect match and control mismatch 25-mer “probe pairs” (together designated as a gene's “probe set”) (Table S1). Probe pairs were designed to minimize cross-reactivity between genes contained within and between the two bacterial genomes: this was confirmed experimentally by hybridizing the GeneChips to cDNA targets prepared from each of five cecal RNAs from one or the other group of mice colonized with a single bacterial species (“mono-associated” animals). When the five B. thetaiotaomicron mono-association–derived cDNA targets were hybridized to the *B. thetaiotaomicron/B. longum* GeneChip, a “present” call (defined by GeneChip software) was obtained from an average of 67.7% of the B. thetaiotaomicron probe sets, but from only 4.3% of B. longum probe sets. Similarly, cDNAs generated from the cecal RNAs of B. longum mono-associated mice produced “present” calls for 63.2% of B. longum probe sets versus 5.6% of B. thetaiotaomicron probe sets. B. thetaiotaomicron probe sets called “present” in at least three of the five GeneChips hybridized with B. longum targets (153 probe sets) were designated as cross-reacting, and eliminated from further analysis using an electronic probe mask. The reciprocal analysis resulted in electronic masking of 84 cross-reacting B. longum probe sets.

The five GeneChip datasets produced from the group of co-colonized animals were then referenced to five GeneChip datasets from the *B. thetaiotaomicron* mono-associated group, yielding a cohort of B. thetaiotaomicron genes showing changed expression as a function of colonization state. A total of 681 B. thetaiotaomicron genes were designated as differentially expressed upon co-colonization using selection criteria that maintained a false discovery rate (FDR) of less than 0.01: 359 (53%) of these genes were expressed at higher levels in the presence of *B. longum,* whereas 322 were expressed at lower levels. Functional categorization of the differentially expressed genes using clusters of orthologous groups (COGs) revealed a “metabolism”-biased response to co-colonization, including statistically significant over-representation of up-regulated B. thetaiotaomicron genes involved in “carbohydrate transport and metabolism” and “energy production and conversion” (*p <* 0.01 based on a binomial distribution test; Figure S1A and S1B; see Table S2 for a list of genes sorted by COGs).

### Co-colonization Prompts B. thetaiotaomicron to Expand Expression of Genes Involved in the Acquisition and Breakdown of Polysaccharides

**Figure 1:**
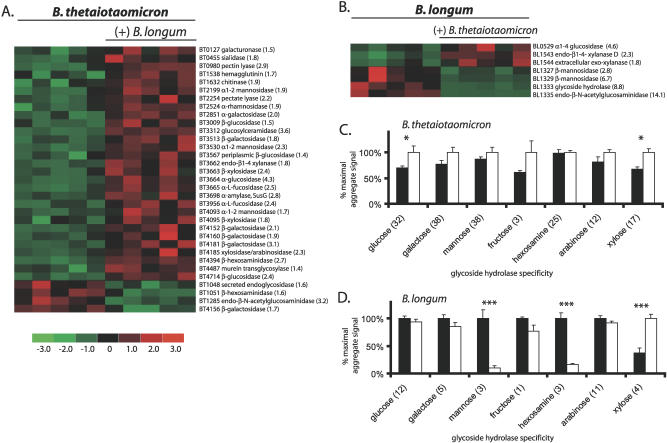
Impact of Co-colonization on B. thetaiotaomicron and B. longum Glycoside Hydrolase and Polysaccharide Lyase Gene Expression (A) and (B) Bacterial glycoside hydrolase and polysaccharide lyase genes (rows) exhibiting differential expression in mono-associated versus co-colonized mice. Colors indicate the deviation of a gene's signal above (red) and below (green) its mean expression value across all ten samples. Thirty-three B. thetaiotaomicron enzymes and seven B. longum enzymes meet the selection criteria for differential expression described in Materials and Methods (FDR < 0.01). Fold-differences in average expression levels between the two colonization states are indicated in parenthesis for each gene. (C) and (D) Average aggregate signal intensity in the two colonization states for the seven groups of glycoside hydrolases/polysaccharide lyases that are common to B. thetaiotaomicron (C) and B. longum (D). The number of enzymes in each group is indicated in parenthesis. Values obtained from mono-associated mice are shown with black bars, and from co-colonized mice with white bars. See Tables S14 and S15 for a list of the B. thetaiotaomicron and B. longum genes, respectively. See Materials and Methods for a description of how “% maximum aggregate signal” was calculated. Mean values ± standard error (S.E.) of five samples are shown: an asterisk (*) indicates *p <* 0.05, and double asterisks (**) indicate *p <* 0.001 according to Student *t*-test.

The B. thetaiotaomicron genome encodes 226 known or predicted glycoside hydrolases and 15 polysaccharide lyases involved liberating oligo- and monosaccharides from polysaccharides (classified in Table S3 according to the nomenclature employed by the Carbohydrate Active enZymes database [CAZy; http://afmb.cnrs-mrs.fr/CAZY/]). The presence of B. longum produced statistically significant (FDR < 0.01) changes in expression of 31 B. thetaiotaomicron glycoside hydrolases and two polysaccharide lyases when compared to B. thetaiotaomicron residing in the ceca of mono-associated mice: the vast majority of these genes (29/33) were up-regulated (Figure 1A). The substrate specificities represented in the 29 up-regulated glycoside hydrolases (20 CAZy families; Figure 1A and Table S3) suggests that B. thetaiotaomicron undergoes an expansion in the diversity of polysaccharides it targets for degradation when in the presence of B. longum.

B. thetaiotaomicron contains 163 paralogs of two components of a well-characterized starch utilization system (Sus): 106 paralogs of the gene encoding SusC, a TonB-dependent, β-barrel-type outer membrane porin that participates in energy-dependent transport of malto-oligosaccharides into the periplasmic space, and 57 paralogs of the gene encoding SusD, a secreted starch-binding protein with an N-terminal lipid tail that allows it to associate with the outer membrane [12]. These paralogs likely confer the ability to bind and import diverse carbohydrate substrates encountered in the gut [19]. Expression of 31 *B. thetaiotaomicron SusC/D* genes was significantly altered (1.4–6.7-fold) by B. longum: nearly two thirds (19 of 31) display higher expression with co-colonization; 12 of these are present in the genome as adjacent pairs of SusC/D paralogs (Figure S2).

Sixty-one B. longum genes showed significantly changed expression upon co-colonization with B. thetaiotaomicron (26 up-regulated, 35 down-regulated; FDR < 0.01). Together, these genes represent a much smaller proportion of the B. longum genome than does the cohort of B. thetaiotaomicron genes affected by B. longum (3.5% versus 14.2%, respectively). Comparison of B. longum genes up- versus down-regulated upon co-colonization revealed that the only significant difference in COG representation involved down-regulated genes associated with “carbohydrate transport and metabolism” (*p <* 0.01; Figure S3; Table S4). Moreover, all down-regulated genes in this COG are located in one region of the B. longum genome (BL1327–BL1337) that is dedicated to the degradation, import, and metabolism of mannosylated glycans. All four glycoside hydrolases with reduced expression are positioned within this gene cluster (see below).

### Co-colonization Results in Differential Expression of Genes Encoding Mannosidases and Related Enzymes

Mannose is highly coveted as a source of energy for many saccharolytic bacteria: it is a ubiquitous monosaccharide found in both plant and animal glycans, and is easily shunted into the glycolytic pathway via an isomerization of mannose-6-phosphate to fructose-6-phosphate. The desirability of mannose as a substrate is evidenced by the fact that the B. thetaiotaomicron genome dedicates over 15% of its repertoire of glycoside hydrolases to the liberation of glycosidically linked mannose (30 mannosidases; ten mannanases). *B. thetaiotaomicron'*s 40 mannose-liberating enzymes contrasts with the three mannosidases present in B. longum.

**Figure 2:**
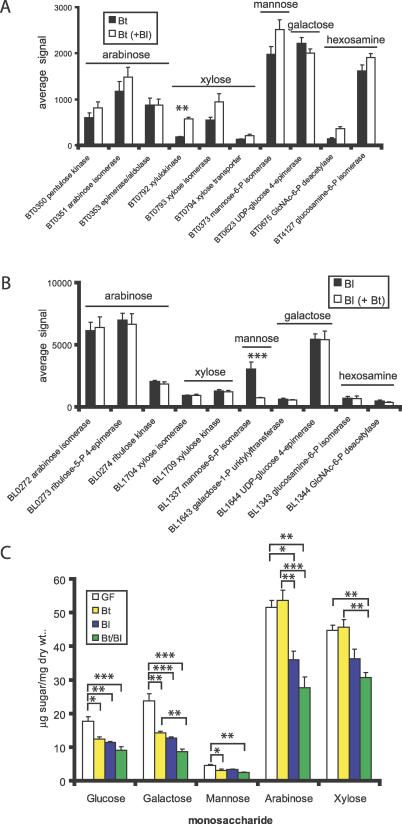
Carbohydrate Utilization by B. thetaiotaomicron and B. longum in the Presence or Absence of One Another (A) and (B) Average expression levels for five cecal samples are shown for genes encoding enzymes that shunt the indicated monosaccharides into the glycolytic or pentose phosphate pathways. Values obtained from mono-associated mice are shown with black bars, and from co-colonized mice with white bars. Significant differences in expression levels were determined using Student *t*-test; double asterisks (**) indicate *p <* 0.01, and triple asterisks (***) indicate *p <* 0.001. (C) GC-MS determination of monosaccharide composition in the ceca of germ-free (GF), B. thetaiotaomicron mono-associated (Bt), B. longum mono-associated (Bl), or *B. thetaiotaomicron/B. longum* co-colonized (Bt/Bl) mice. Mean values ± S.E. of biological triplicates are plotted. Significant changes in the abundance of a monosaccharide were identified using a one-way ANOVA followed by a Tukey post test; a single asterisk (*) indicates *p <* 0.05, double asterisks (**) indicate *p <* 0.01, and triple asterisks (***) indicate *p <* 0.001.

Three of *B. thetaiotaomicron'*s mannosidases are more highly expressed with bi-association (BT2199, BT3530, and BT4093; 1.9-, 2.3-, and 1.7-fold, respectively), whereas two of *B. longum'*s mannosidases show reduced expression (BL1327 and BT1329; 2.8- and 6.7-fold, respectively) (Figure 1). These two B. longum α-mannosidases are positioned in the gene cluster referred to above that contains two linked permease genes (BL1331 and BL1332; down-regulated 7.4- and 10.1-fold, respectively) and two other glycoside hydrolase activities (BL1333 [substrate specificity unknown]; BL1335 [endo-β-N-acetylglucosaminidase]; down-regulated 8.8- and 14.1-fold, respectively; Figure 1B and 1D). Expression of nine of the ten genes encoding B. longum enzymes that deliver monosaccharides to the glycolytic and pentose phosphate pathways is unchanged by the presence of B. thetaiotaomicron (Figure 2). The lone exception is another component of the gene cluster, a mannose-6-phosphate isomerase (BL1337) that shows 4.2-fold lower expression with co-colonization (*p <* 0.001; Figure 2B). Thus, B. thetaiotaomicron and B. longum exhibit changes in their transcriptome that suggest differences in either the extent or efficiency of mannoside utilization occur during co-colonization compared to when either species is the sole occupant of the cecum. The apparent decreased catabolism of mannose by B. longum is consistent with its decreased expression of mannosidases. Nonetheless, despite the relative disparity in mannoside-degrading enzymes between the two organisms (40 in B. thetaiotaomicron versus three in B. longum), we must be cautious before concluding that B. longum is a relatively poor competitor for mannosylated glycans. For example, in the co-colonized cecum, other substrates may be available that B. longum prefers over mannose, resulting in down-regulation of its mannosidase genes, increases in mannose levels, and subsequent up-regulation of *B. thetaiotaomicron'*s mannosidase genes. Alternatively, co-colonization could result in changes in the environment, such as pH or osmolarity, that render *B. longum'*s mannosidases more stable, or more efficient, resulting in their subsequent down-regulation.

### B. thetaiotaomicron Up-Regulates Machinery Involved in Xyloside Degradation and Xylose Catabolism in the Presence of B. longum

In contrast to their disparate responses to mannose-containing glycans, expression of enzymes dedicated to degradation of xylose-containing glycans is enhanced in both bacterial species when they co-exist in the cecum. B. thetaiotaomicron increases expression of four xyloside-degrading glycoside hydrolases: an endo-xylanase predicted to be secreted (BT3662; 1.8-fold), an exo-xylanase (BT3663; 2.4-fold), and two secreted xylosidases (BT4095 and BT4185; 1.8- and 2.3-fold, respectively) (FDR < 0.01; Figure 1A and 1C).

Two of the three glycoside hydrolases that B. longum up-regulates are secreted xylanases that occur in a putative operon: BL1543, an endo-xylanase (2.3-fold), and BL1544, an exo-xylanase (1.8-fold), both of which contain sortase motifs [20], indicating that they are located on the surface of this Gram-positive bacterium (Figure 1B). Xylose-liberating hydrolases are the only group of B. longum glycoside hydrolases that show significantly higher expression when *B. thetaiotaomicron is* present (*p <* 0.001; Figure 1D).

### Verification of Bacterial Glycan Utilization Behaviors within the Ceca of Colonized Mice

The coincident regulation of glycoside-degrading machinery by B. thetaiotaomicron and B. longum suggests that when both components of this model community are present in their cecal habitat, utilization of a subset of available polysaccharide substrates changes. To examine this notion further, gas chromatography–mass spectrometric (GC-MS) analysis was employed to establish which monosaccharide components of polysaccharides were significantly depleted in each colonization state. When compared to germ-free controls, mono-association with B. thetaiotaomicron produced a preferential depletion of the three most prominent hexoses (glucose, galactose, and mannose), whereas there were no significant changes in the most prominent pentoses (arabinose and xylose). B. longum mono-association also resulted in significant depletion of galactose, glucose, and mannose: however, unlike B. thetaiotaomicron mono-association, the pentose arabinose was also depleted (Figure 2C). This latter observation is consistent with *B. longum'*s considerable arabinoside-degrading capability (11 arabinosidases, exceeded only by glucosidases as the most highly represented class of glycoside hydrolases in its genome; Table S3). Consistent with the documented co-colonization–associated up-regulation of xylose-degrading enzymes by both B. thetaiotaomicron and *B. longum,* xylose was depleted to a significant degree when both bacterial species were present in the cecal habitat (Figure 2C).

To determine if depletion of xylose in the co-colonized state could be attributed to differential usage by either bacterial species, we performed in silico metabolic reconstructions to visualize expression of genes encoding enzymes that shunt monosaccharides liberated by the glycoside hydrolases/polysaccharide lyases into either the glycolytic or pentose phosphate pathways. With co-colonization, B. thetaiotaomicron increases expression of only one of its ten genes that can be assigned to these pathways: a xylulokinase (BT0792) that catalyzes a key step in D-xylose catabolism (3.1-fold increase with co-colonization, *p <* 0.01; Figure 2A). In contrast, neither of the two enzymes assigned to xylose-specific metabolism in B. longum—a xylose isomerase (BL1704) and xylulokinase (BL1709)—showed differences in their expression between the two colonization states.

The observed up-regulation of genes involved in both liberation and catabolism of xylose by B. thetaiotaomicron indicates that it is responsible for at least some of the depletion that occurs during co-colonization. The up-regulation of xyloside-degrading machinery by B. thetaiotaomicron and B. longum may be a result of both bacterial species turning to an available, but less-coveted substrate that is neglected in mono-association, but accessed when their preferred substrates are depleted in the more competitive environment of co-colonization. Although less parsimonious, an alternative possibility is that each bacterial species, when present alone in the cecum, possesses a complement of glycoside hydrolases that is insufficient for efficient degradation of available xylose-containing glycans. However, when their repertoires of glycoside hydrolases are combined, complementary enzymes activities allow the two bacterial species to participate in a “synergistic” harvest of this class of glycans, a phenomenon observed in other mixed microbial communities that degrade cellulose [21,22]. A third, formal possibility is that in mono-associated mice, each species restrains expression of these genes (despite the presence of substrate), but with co-colonization, in response to some signal from the other species, they up-regulate the genes to the combined benefit of both species. This is cooperation (the sacrifice of individual fitness for the fitness of the group) and is unlikely to be taking place because of the inevitability of “cheating” [23].

### B. longum Induces an Expansion of *B. thetaiotaomicron'*s Substrate Range Independent of Host Genotype

Mice belonging to the NMRI inbred strain were selected as the initial host for our experiments because of the ease of rearing them in a germ-free state. To determine the generality of *B. thetaiotaomicron'*s response to co-colonization with *B. longum,* we also performed a series of experiments, identical to those described above (including use of the same autoclaved chow), but in animals belonging to the C57BL/6J (B6) inbred strain. Ten day–long mono- and bi-associations of germ-free 6–8-wk-old male B6 mice produced levels of colonization similar to that documented in their NMRI counterparts (*p >* 0.05; unpublished data).

We identified genes whose expression changes significantly as a function of host genotype, by applying a two-stage mixed analysis of variance (ANOVA) model to our 20 GeneChip datasets (i.e., five NMRI B. thetaiotaomicron mono-associated, five NMRI *B. thetaiotaomicron/B. longum* co-colonized, five B6 B. thetaiotaomicron mono-associated, and five B6 *B. thetaiotaomicron/B. longum* co-colonized). The ANOVA model identified a total of 2,369 B. thetaiotaomicron genes that were differentially expressed in the two host mouse strains independent of colonization state (50.2% of the 4,719 genes surveyed; FDR < 0.01; see Figure S4, plus Table S5 for a full list of genes identified by the ANOVA model and the associated *p*-values and *q*-values of each gene, as well as Tables S6 and S7, plus Figure S5 for a COG-based functional categorization of the 1,168 B. thetaiotaomicron genes that exhibit higher expression in NMRI versus B6 mice, and the 1,201 genes that exhibit higher expression in B6 versus NMRI).

Despite the large effect of host genotype on B. thetaiotaomicron gene expression, the B. longum–induced expansion in the carbohydrate substrate range of B. thetaiotaomicron occurs independently of host genotype. The ANOVA model identified 1,806 genes whose expression is significantly changed as a function of colonization state (mono-association or co-colonization; Figure S6A). Comparison of the 801 genes that are expressed at higher levels during co-colonization, with the 1,005 genes expressed at reduced levels (Tables S8 and S9), revealed that “carbohydrate transport and metabolism” is one of the most significantly over-represented COGs (15% of the response, *p <* 10^−10^; Figure S6B). Nearly twice as many glycoside hydrolases/polysaccharide lyases exhibit higher expression during co-colonization compared to mono-association (61 up-regulated versus 33 down-regulated; Figure S7A and S7B). Similarly, *SusC/D* paralogs whose expression changes as a function of colonization state show a strong bias toward up-regulation with co-colonization (43 up-regulated versus 22 down-regulated; Figure S7A and S7B). In several instances, co-regulated *SusC/D* paralogs and glycoside hydrolases/polysaccharide lyases are present together in putative operons, a genomic feature that would allow B. thetaiotaomicron to efficiently coordinate glycan binding and import with glycan degradation (Figure S7A and S7B).

A comparison of the glycoside hydrolase/polysaccharide lyase families represented in the groups of genes that increase or decrease their expression during co-colonization irrespective of host genotype further substantiated that *B. thetaiotaomicron'*s polysaccharide substrate preferences change in the presence of B. longum. The 61 polysaccharide-degrading enzymes that show increased expression fall into 27 different glycoside hydrolase/polysaccharide lyase families (44 families are represented in the entire B. thetaiotaomicron genome), consistent with a broad diversification of glycan utilization. The most highly represented glycoside hydrolase family, 43 (11 members), provides evidence that substrates containing β-xylose and α-arabinose become a major focus of B. thetaiotaomicron when B. longum is present (Figure S7C). Alpha-mannosidases and β-galactosidases (glycoside hydrolase families 2 and 92, respectively) are also abundantly represented (Figure S7C).

The 33 glycan-degrading enzymes with decreased expression also display diverse activities: they represent 18 glycoside hydrolase/polysaccharide lyase families, and show a distribution dissimilar to the up-regulated enzymes (Figure S7C). Highly represented activities distinct from those present in the up-regulated group include β-hexosaminidases (glycoside hydrolase family 20) and α-fucosidases (glycoside hydrolase family 95), the enzymes that liberate monosaccharides commonly found in host mucus glycans, which are coveted substrates of B. thetaiotaomicron in vivo [17]. Together, these data indicate that B. longum induces a shift in B. thetaiotaomicron substrate preferences away from B6 and NMRI mucus glycans and towards arabinose- and xylose-containing plant polysaccharides in the host diet. This change in glycan foraging behavior could also be responsible for altering the functional relationships between B. thetaiotaomicron and its host (see below).

### Expansion of the Carbohydrate Substrate Range of B. thetaiotaomicron Can Be Induced by Multiple Probiotic Species

Probiotic bacterial species vary in their ability to establish residency within the human intestinal microbiota. Another *Bifidobacterium* species, *B. animalis,* and *L. casei,* a member of a different bacterial division (the Firmicutes), are common ingredients of fermented milk products [24,25]. Both species, although less well adapted than B. thetaiotaomicron or B. longum to take up residency in the human gut, can remain viable during their transit through our gastrointestinal tracts [26–28].

To determine the specificity of *B. thetaiotaomicron'*s response to *B. longum,* we performed expression profiling of B. thetaiotaomicron in the presence of either B. animalis DN-173 010 (Danone Vitapole, Jouy-en-Josas, France) or L. casei DN-114 001 (Danone Vitapole, CNCM number, I-1518). To ensure continuous interaction between B. thetaiotaomicron and these two frequently ingested strains, B6 mice were mono-associated with each of the three species, or were first given B. thetaiotaomicron followed by B. animalis or L. casei every 3 d over a 10-d span (five mice/treatment group). Importantly, at the time of sacrifice, the level of cecal colonization was similar in mono- or bi-associated animals for all bacterial species (8 × 10^10^ to 3 × 10^12^; *p >* 0.1).

**Figure 3:**
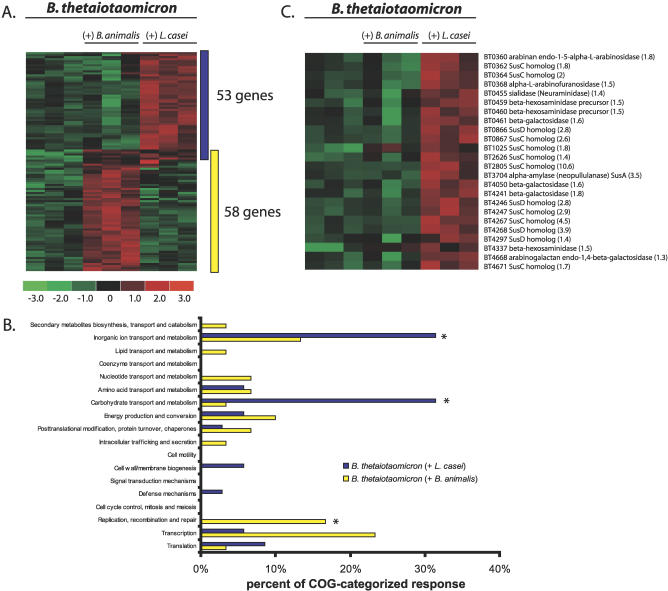
Changes in the B. thetaiotaomicron Transcriptome in Response to Co-colonization with *B. animalis* or *L. casei* (A) B. thetaiotaomicron genes that show significantly increased expression levels upon co-colonization with either of the two fermented dairy product–associated strains are shown. Selection criteria: (1) a 1.2-fold or greater difference in expression using the 90% confidence bound of the fold-change; (2) difference in signal ≥ 25; (3) *p*-value ≤ 0.025 using Student *t*-test; (4) transcript called “present” in 66% or more of the GeneChips in the co-colonized group; and (5) a median FDR less than 0.05 based on 50 permutated comparisons. (B) COG-based functional classification of genes identified in (A). Thirty-five of 53 genes with higher expression in the presence of L. casei and 30 of 58 genes with higher expression in the presence of B. animalis could be assigned to COGs. Functional categories with significant over-representation in the dataset of differentially expressed genes, compared to their representation in the B. thetaiotaomicron genome, were determined using a hypergeometric distribution; an asterisk (*) indicates *p <* 0.01. (C) All B. thetaiotaomicron SusC/D paralogs and glycoside hydrolases/polysaccharide lyases that show differential expression between the three colonization states are up-regulated in the presence of L. casei. Fold-differences are indicated in parenthesis and reflect average expression across three cecal samples when B. thetaiotaomicron is co-colonized with *L. casei,* compared to their average expression in three cecal samples obtained from B. thetaiotaomicron mono-associated animals.

Whole genome transcriptional profiling of cecal populations of B. thetaiotaomicron revealed that the impact of B. animalis and L. casei on the B. thetaiotaomicron transcriptome was modest when compared with the response elicited by B. longum. Fifty-eight genes showed increased levels of expression in the presence of B. animalis and 53 increased in the presence of L. casei (FDR < 0.05; Figure 3A; see Tables S10 and S11 for gene lists). Moreover, the principal COG categories representing the response to B. animalis were “transcription” and “replication” (Figure 3B), whereas the two COG categories that dominate the response to L. casei are “carbohydrate transport and metabolism” and “inorganic ion transport and metabolism.” These latter two categories are composed largely of glycoside hydrolase genes and *SusC/D* paralogs, respectively, and represent over 60% of *B. thetaiotaomicron'*s functionally categorized response (Figure 3B). The enrichment of glycoside hydrolases (11 genes; four galactosidases, three hexoaminidases, two arabinosidases, one amylase, and one sialidase) and *SusC/D* paralogs (13 genes) in the dataset of 53 B. thetaiotaomicron genes up-regulated by L. casei (Figure 3C) is functionally similar to the B. longum–induced response. This stands in contrast to *B. animalis,* which does not significantly impact expression of any glycoside hydrolase or *SusC/D* paralog in the B. thetaiotaomicron genome.

The L. casei up-regulation of three B. thetaiotaomicron glycoside hydrolase family 20 hexosaminidases contrasts with the down-regulation of this family's hexosaminidases that occurs in the presence of B. longum (Figure S7C). Two of the induced hexosaminidases (BT0459 and BT0460) are present in a putative operon containing an up-regulated α-sialidase and β-galactosidase dedicated to degrading sialylated glycans from host mucus (BT0455–BT0461; Figure 3C). In addition to the expanded degradation of host glycans, *B. thetaiotaomicron'*s up-regulation of three enzymes that target arabinose-containing polysaccharides (BT0360, endo-α-arabinosidase; BT0368, α-arabinofuranosidase; and BT4668, arabinogalactan endo-β-galactosidase) suggests that accessing dietary plant-derived substrates is also part of the response to L. casei.

In summary, comparison of *B. thetaiotaomicron'*s response to the three bacterial species revealed that they are able to induce distinct responses in *B. thetaiotaomicron,* both in terms of the magnitude of the response (i.e., the number of B. thetaiotaomicron genes that undergo significant changes in expression) and the function of affected genes. The disparity in identity and diversity of polysaccharide substrates targeted by B. thetaiotaomicron in response to B. longum versus L. casei suggests that the nature of the expansion, the resulting impact on the intestinal nutrient environment, and ultimately the effect on the host, are likely to show significant specificity with regard to the micro-organism consumed.

### Host Epithelial Response to B. thetaiotaomicron plus B. longum Is Characterized by a Synergistic Induction of Interferon-Responsive Genes

The transcriptional responses of B. thetaiotaomicron and B. longum to co-colonization, and the resulting transformation in the cecal nutrient environment raised the question of how host epithelial responses differ between colonization states. Therefore, we performed comprehensive transcriptional profiling on cecal epithelia harvested from the same groups of NMRI mice that were used to profile bacterial gene expression (i.e., B. thetaiotaomicron mono-associated; B. longum mono-associated, and *B. thetaiotaomicron/B. longum* co-colonized). A group of age-matched, untreated male germ-free NMRI mice that were given the same batch of autoclaved chow served as additional controls.

Laser capture microdissection (LCM) was performed on cecal cryosections prepared from four mice/treatment group (~5,000 epithelial cells recovered/cecum). RNA was prepared from epithelial cells harvested from the cecum of each individual mouse in each treatment group. Equivalent amounts of RNA from each cecum were then pooled from each mouse per group, targets were generated by two independent amplifications of each pooled RNA sample, and each amplified RNA preparation was hybridized to a separate Affymetrix mouse 430 v2.0 GeneChip.

**Figure 4:**
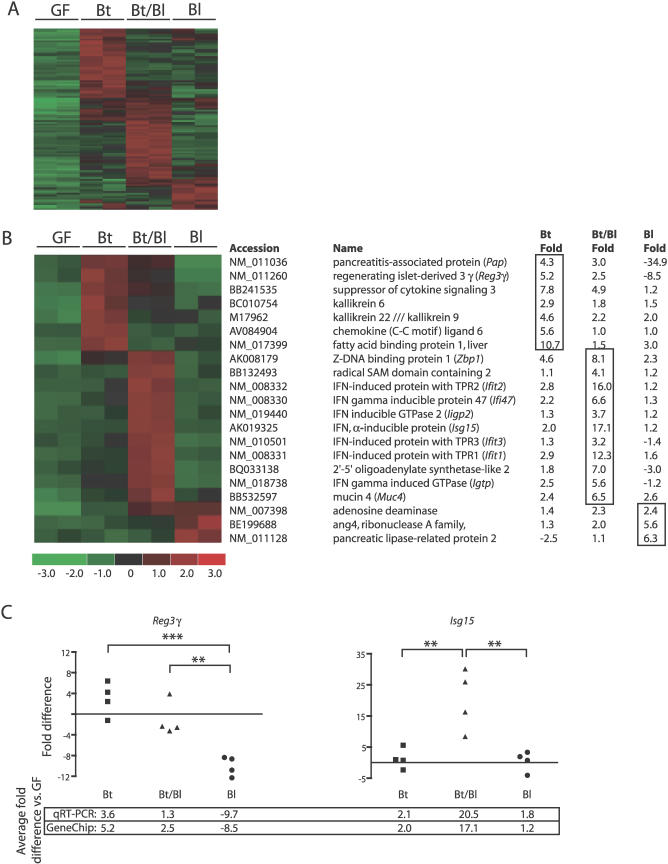
Gene Expression Changes in the Cecal Epithelium Induced by Colonization (A) Cluster diagram from GeneChip studies of laser capture microdissected mouse cecal epithelium illustrates genes that meet the four criteria described in Materials and Methods including a 2-fold or greater increase in expression (using the 90% confidence bound of fold-change) in B. thetaiotaomicron mono-associated (Bt), B. longum mono-associated (Bl), or co-colonized ceca (Bt/Bl) compared with germ-free (GF) controls. (B) Genes involved in immuno-inflammatory responses that are up-regulated in at least one colonization state; fold-increase in expression in each colonization state is relative to the germ-free state. The expression pattern for Pap (Pancreatitis associated protein, a member of the C-type family of lectins known to bind terminal glycan structures in fungi and bacteria), and several other genes was confirmed using an independent GeneChip platform that queries a subset of genes in the mouse genome relevant to carbohydrate metabolism (Glyco-GeneChips; see Figure S9). (C) Real-time quantitative PCR assays of laser-captured microdissected cecal epithelial RNA isolated from individual animals colonized with B. thetaiotaomicron (Bt), B. longum (Bl), or co-colonized with both organisms (Bt/Bl) (*n* = 4 mice/group), using primers specific for *Reg3γ* or *Isg15*. Fold-changes are expressed relative to a reference RNA created by pooling equivalent masses of laser-captured microdissected cecal epithelial RNA from four germ-free animals. Average fold-changes for each group are indicated at the bottom, together with fold-changes derived from the GeneChip analysis. Significant differences in expression between groups for each gene were identified using a one-way ANOVA and Tukey post test; double asterisks (**) indicate *p <* 0.01, and triple asterisks (***) indicate *p <* 0.001.

Expression profiles from each of the three colonization states were referenced to datasets obtained from germ-free controls to identify significantly up-regulated genes using criteria described in Materials and Methods. These comparisons yielded 50 genes in the cecal epithelium of B. thetaiotaomicron mono-associated mice, 24 genes in B. longum mono-associated hosts, and 52 genes in co-colonized animals, whose expression was defined as significantly increased relative to germ-free epithelium. Comparison of genes up-regulated in each colonization state revealed that the host response to co-colonization was distinct from the response to mono-association with either organism (Figure 4A and 4B): i.e., of the 52 genes identified in the epithelial response to the two-component *B. thetaiotaomicron/B. longum* model community, only 15 genes are shared with the response to B. thetaiotaomicron alone, and only seven genes with the response to B. longum.

Since these conclusions were based on a GeneChip analysis of pooled RNAs, we performed follow-up qRT-PCR assays of laser capture microdissected cecal epithelial RNAs isolated from the individual animals used to prepare the pooled samples (*n* = 4/colonization state). *Reg3γ* and *Isg15* were selected for verification because they had exhibited different expression profiles across the three colonization states. The qRT-PCR results established that GeneChip data obtained from pooled RNA samples were representative of individual animals within each group (Figure 4C).

To gain a broader perspective of the host responses to each of the three colonization states, we categorized all up-regulated host genes using Ingenuity Pathways Analysis software (http://www.ingenuity.com). A right-tailed Fisher exact test was used to determine significantly over-represented (*p <* 0.05) functional categories containing a minimum of eight genes (Table S12). Gene interaction networks were then generated for each colonization state using these up-regulated genes as “focus genes” among the thousands of genes called “present” for each condition (B. thetaiotaomicron mono-association, 10,795 genes; B. longum mono-association, 10,479 genes; and co-colonization, 12,138 genes). Unsupervised generation of molecular interaction networks, based on (1) focus genes, (2) genes called “present”, and (3) unrepresented but highly connected genes was performed by the Ingenuity Pathway Analysis software using a knowledge base that consists of more than 1,000,000 functional and physical interactions for more than 23,900 mammalian genes (including 8,200 for mouse). These networks reveal that the host response to B. thetaiotaomicron is centered on tumor necrosis factor-α (TNF-α; Figure S8A), an LPS-responsive cytokine produced by natural killer (NK) and T cells and macrophages. This contrasts to the network generated by the B. longum response which is centered on the T cell–produced cytokine, interferon-γ (IFN-γ; Figure S8B).

The network generated from the co-colonization gene set revealed that both TNF-α and IFN-γ are central highly connected nodes (Figure S8C), suggesting that the cytokine milieu in the cecal epithelium of co-colonized mice shares characteristics with each mono-association state.

Consistent with the Ingenuity Pathway Analysis network construction, the group of genes showing highest expression in the co-colonized state relative to germ-free controls includes three members of the interferon-inducible 47kDa GTPases (*Igtp,* 5.6-fold; *Ifi47,* 6.6-fold; and *Iigp2,* 3.7-fold) and three members of the interferon-inducible p56 family (*Ifit1,* 12.3-fold; *Ifit2,* 16.0-fold; and *Ifit3,* 3.2-fold). The interferon-stimulated gene-15 *(Isg15)*, a ubiquitin family member implicated in mediating viral resistance [29,30], is induced 17.1-fold with co-colonization (20.5-fold by qRT-PCR) compared to 2.0-fold (2.1-fold by qRT-PCR) and 1.2-fold (1.8-fold by qRT-PCR) with B. thetaiotaomicron and B. longum mono-associations, respectively (Figure 4B and 4C). Two other interferon-responsive genes, *Zbp-1,* a Z-DNA binding adenosine deaminase, and *Muc4,* a mucin capable of inhibiting viral capsid binding to target glycans [31] are up-regulated in all colonization states but show highest expression with co-colonization (Figure 4B).

In addition to inducing host cytokine-responsive genes, B. longum appears to possess the capacity to regulate host antibacterial proteins. Reg3γ (regenerating islet-derived-3γ) is a C-type lectin that is secreted by an epithelial cell lineage that plays an important role in innate immune responses. Reg3γ binds with high affinity to peptidoglycan, and exhibits broad microbicidal activity against Gram-positive bacteria [32]. We found that although Reg3γ is induced in mice colonized with B. thetaiotaomicron alone (5.2-fold above germ free; 3.6-fold by qRT-PCR), it is significantly down-regulated when B. longum is present by itself (repressed 8.5-fold compared to germ free; 9.7-fold by qRT-PCR) (Figure 4B and 4C). Pancreatitis-associated protein (Pap; *Reg3β*), another member of the Reg family that also contains a lectin domain, displayed an expression profile similar to *Reg3γ* on two different types of Affymetrix GeneChips, including a custom GlycoGeneChip containing components of the mouse gene involved in carbohydrate metabolism (http://www.functionalglycomics.org see Figures 4B and S9). The ability of B. longum to repress host expression of antibacterial proteins may not only promote its own survival in the gut, but also influence the composition, structure, and function of its microbial community.

### Prospectus

Using gnotobiotic mice, we have investigated the effects of introducing three commonly consumed live microbial strains that vary in their ability to colonize the human gut, on *B. thetaiotaomicron,* a prominent symbiotic member of our microbiota, and on the host. Our findings indicate that when B. thetaiotaomicron encounters *B. longum,* it expands its repertoire of prominently expressed glycoside hydrolases, including multiple enzymes involved in mannoside and xyloside degradation, and up-regulates xylose catabolism. By comparing the responses in two different inbred strains of gnotobiotic mice, we find that host genotype affects the specific suite of microbial genes deployed when B. thetaiotaomicron encounters *B. longum.* However, B. longum–induced diversification in the carbohydrate substrates accessed by B. thetaiotaomicron occurs independent of host genotype and is characterized by a general shift in *B. thetaiotaomicron'*s focus away from mucus glycans and towards dietary plant polysaccharides.

The B. animalis and L. casei strains selected for our study are common components of the fermented dairy foods we ingest. Co-colonization of either species with B. thetaiotaomicron has allowed us to conclude that (1) if glycoside gene expression is used as a biomarker of substrate range, the impact of B. longum on B. thetaiotaomicron is not a generic effect of Bifidobacteria (it is not detectable with B. animalis); and (2) *L. casei,* a member of a different division of Bacteria, can emulate the effects of B. longum on *B. thetaiotaomicron'*s polysaccharide utilization—albeit by altering expression of a different constellation of B. thetaiotaomicron genes. Once genome sequences for these strains become available, it will be possible to ascertain how B. thetaiotaomicron influences their transcriptomes, and thus establish more about the nature and consequences of their interactions. The changes that we observe may be due to direct effects of the co-habitating species on one another (e.g., through production of chemical messengers), or indirect effects (e.g., changes in substrate availability or changes in host response).

Together, our findings emphasize the importance of assessing the impact of probiotics on our gut microbiota. Humans have complex and varied microbiotas, non-uniform diets, and diverse genotypes. Further tests of the mechanism of action of commonly ingested live microbial supplements could include creation of more elaborate gnotobiotic mouse models containing intentional communities composed of sequenced representatives of other prominently represented divisions of bacteria in our gut microbiota (e.g., a member of the Firmicutes combined with a member of Bacteroidetes). These studies should utilize strains of commonly used probiotics whose genomes have been sequenced, pay attention to variables such as the diet and genetic background of the mouse, look at effects in different regions of the digestive tract, and seek to identify serum- or fecal-based biomarkers of host responses that may be applicable to humans. Since we do not yet know the degree to which differences in our microbial ecology reflect differences in the metabolic activities of our microbiotas, it seems reasonable to suggest that current clinical trials of the effects of presumptive probiotics also pay careful attention to documenting a subject's microbiota prior to, during, and after treatment. In principle, the impending revolution in metagenomics may help by allowing comprehensive documentation of changes in the gut “microbiome” of a given individual as a function of probiotic administration. As illustrated by a recent study, computational tools are now in hand to take metagenomic datasets of a fecal microbiome, and infer features of the metabolic properties of a person's gut microbial community [33].

## Materials and Methods

### Colonization of germ-free mice.

Germ-free mice belonging to either the NMRI-KI or C57BL/6J inbred lines were housed in plastic gnotobiotic isolators [34] under a strict 12-h light cycle (lights on at 0600 h) and fed a standard autoclaved chow diet ad libitum (B&K Universal, East Yorkshire, United Kingdom; see Table S13 for detailed nutrient information). Six- to 8-wk-old male mice were each inoculated with 10^8^ CFU of (1) B. thetaiotaomicron (ATCC 29148, also known as VPI-5482), that had been grown overnight in TYG medium (1% tryptone, 0.5% yeast extract, 0.2% glucose, w/v) supplemented with 100 mM potassium phosphate buffer, (pH 7.2), 4.1 mM cysteine, 200 μM histidine, 6.8 μM CaCl_2_, 140 nM FeSO_4_, 81 μM MgSO_4_, 4.8 mM NaHCO_3_, 1.4 mM NaCl, 1.9 μM hematin, plus 5.8 μM Vitamin K_3_, and/or (2) B. longum (NCC2705) grown overnight in de Man-Rogosa-Sharpe medium (MRS; Sigma-Aldrich, St. Louis, Missouri, United States).

Following a 10-d colonization, animals were sacrificed, their ceca dissected, and the contents of each cecum extruded from its distal end. Bacterial densities were determined by performing serial dilutions of cecal contents in PBS and plating onto brain-heart infusion blood agar plates (supports B. thetaiotaomicron and B. longum growth) and MRS-agar plates (supports B. longum growth), followed by incubation at 37 °C for 48 h under anaerobic conditions. B. thetaiotaomicron and B. longum colonies could be distinguished based on differences in their morphology: these differences were confirmed by colony-based PCR using species-specific 16S rRNA primers (B. thetaiotaomicron [forward, 5′GGTAGTCCACACAGTAAACGATGAA; reverse, 5′CCCGTCAATTCCTTTGAGTTTC]; B. longum [forward, 5′AAGCTTTCGCGGTATGG; reverse 5′GGCTTGCGCCCATTGTG]).

L. casei DN-114 001 and B. animalis DN-173 010 were administered to 8-wk-old germ-free male C57BL/6J mice that had been mono-associated with B. thetaiotaomicron for 10 d. Animals were gavaged with 10^8^ CFU of bacteria every third day. Control *B. thetaiotaomicron–*colonized mice received a gavage of culture medium alone (MRS). All animals were sacrificed 10 d after initial administration of the bacterial strain or MRS medium. Cecal contents were serially diluted into PBS and selectively plated on to MRS-agar (to isolate either L. casei or B. animalis) and blood agar plus 20 μg /ml of ampicillin (to isolate B. thetaiotaomicron).

### Preparation of bacterial RNA from cecal contents and GeneChip target synthesis.

The remaining cecal contents were flash frozen in liquid nitrogen immediately after their harvest from each mouse in each treatment group, and stored at −80 °C until use. An aliquot of the frozen material (200 mg) was thawed in two to three volumes of RNAProtect (Qiagen, Valencia, California, United States). The suspension was centrifuged (3000 × *g,* 10 min), and 500 μl of a solution containing 200 mM NaCl/20 mM EDTA was added to the resulting pellet, together with 200 μl of 20% SDS (prepared in water) and 500-μl phenol:chloroform:isoamyl alcohol (125:24:1; [pH 4.5]; Ambion, Austin, Texas, United States). Acid-washed silica beads (Sigma; 212–300-μm diameter; 250 mg) were added, and bacteria were lysed mechanically using a Mini-Beadbeater (BioSpec Products, Bartlesville, Oklahoma, United States) set on high (5 min; room temperature). After centrifugation (13,000 × *g,* 3 min, 4 °C), the sample was re-extracted in phenol:chloroform:isoamyl alcohol, precipitated with 60 μl of 3 M sodium acetate (pH 5.2) plus 600-μl cold isopropanol, and RNA was purified using an RNeasy kit (Qiagen).

GeneChip targets were prepared as described [17]. Briefly, genomic DNA was removed using a DNA-free kit (Ambion). cDNA targets were prepared from 5–10-μg aliquots of each RNA sample using protocols described in the *Escherichia coli* Antisense Genome Array manual (Affymetrix). RNA was reverse-transcribed using SuperScript-II RT (Invitrogen, Carlsbad, California, United States). The RNA template was then destroyed by incubation with 0.25 M NaOH for 30 min at 65 °C. The cDNA product was isolated (QIAquick spin columns; Qiagen), fragmented (DNase-I; Amersham Biosciences, Little Chalfont, United Kingdom), and biotinylated (Enzo-BioArray Terminal Labeling Kit; Affymetrix). Standard Affymetrix protocols were used for hybridization of the cDNA targets to each *B. thetaiotaomicron–B. longum* community GeneChip.

### Analysis of bacterial GeneChip datasets.

GeneChip data were analyzed using DNA-Chip Analyzer (dChip; http://www.biostat.harvard.edu/complab/dchip/). GeneChips were normalized, and model-based expression values were generated (PM-MM model). Signals from spike-in control transcripts and oligo-B2 (Affymetrix) were used to assess the quality of the target preparation and target hybridization, respectively. Comparisons are performed on specified GeneChip datasets to identify genes up-regulated in the experimental group (E) relative to the baseline group (B). Criteria used to determine significant changes in expression were guided by the median FDR of 50 permutations in which samples were randomly shuffled between groups. The following four criteria were routinely used to identify significantly up-regulated genes while maintaining an empirical FDR of less than 0.01: (1) E/B > 1.2 using 90% confidence bound of fold-change; (2) E − B ≥ 50; (3) E = B *p* ≤ 0.05; and (4) called “present” in 60% or more of E GeneChips.

### GeneChip ANOVA.

Probe intensities from *B. thetaiotaomicron/B. longum* GeneChips were imported and the total GeneChip intensities were normalized using dChip [35]. All probe intensities for each gene on each GeneChip were combined in dChip and exported to SAS/STAT Software Version 9 (SAS Institute, Cary, North Carolina, United States). Exploratory data analysis was done and the log2-transformed expression values were normally distributed, which is a necessary criterion when applying any ANOVA model. We then applied a two-step mixed ANOVA model to these log-transformed values [36]**.** The first mixed ANOVA model removed the genome-wide effect of different colonization states and different host genetic backgrounds on the expression levels:

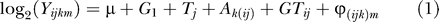
where *Y_ijkm_* is the gene expression value, *μ* is the baseline expression of each gene independent of all other factors, *G_i_* is the average genotype effect (NMRI versus B6), *T_j_* is the average treatment effect (mono-association versus co-colonization), *GT_ij_* is the average genotype by treatment interaction effect, *A_k(ij)_* is the average array effect, and *ϕ_(ijk)m_* is the residual.

We calculated standardized residuals by 
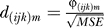
 , where *MSE* = 5.6468. Any value that had *d_(ijk)m_* > 4 was considered an outlier and removed. In total, 178 expression values were removed. In addition, “BT1174,” “BT1703,” and “BT2856” were excluded from further analysis since only a few of their expression values remained in the dataset after the removal of outliers. The remaining gene values were reanalyzed using the first ANOVA model shown above. Since the coefficients for the GeneChip effect were not significantly different from zero, suggesting there was no GeneChip effect, we did not model the GeneChip effect in the second ANOVA model. The second ANOVA model was applied to each gene separately using the residual *φ_(ijk)m_* from each gene as a response variable:

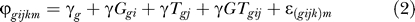
where *ϕ_gijkm_* is the residual from the first ANOVA model, *γ_g_* is the average gene expression for each gene *g, γG_gi_* is the gene expression due to genotype *i* (NMRI versus B6), *γT_gj_* is the gene expression due to treatment *j* (mono-association versus co-colonization), *γGT_gij_* is the gene expression due to genotype *i* interacting with treatment *j,* and *ɛ_(gijk)m_* is the residual.

To avoid problems associated with multiple hypotheses testing, we did not use *p*-values from the second ANOVA model to determine the statistical significance of each effect for each gene. Instead, we calculated the *q*-value for each gene based on the distribution of *p*-values across all genes. The *q*-value is a measure of the FDR: the percentage of genes incorrectly deemed to have significant expression changes [37]. The advantage of using *q*-values, rather than *p*-values, is that the calculation of *q*-values automatically takes into account that many hypotheses are being tested simultaneously [37]. For each gene, we calculated the *q*-values for genotype effect, treatment effect, and genotype by treatment interaction effect, using the program QVALUE [37]. We considered a gene's expression to exhibit a significant effect (genotype, treatment, or genotype-by-treatment interactions) if its *q*-value for that effect is less than 0.01. A *q*-value of 0.01 for genotype effect is equivalent to a *p*-value of 0.03937. A *q-*value of 0.01 for treatment effect is equivalent to a *p*-value of 0.01833, and a *q*-value of 0.01 for the interaction effect is equivalent to a *p*-value of 0.006818.

The ANOVA provided us with sets of genes displaying significant genotypic effect, treatment effect, and interaction effects. We divided these genes into seven sets: set 1 includes genes with only genotypic effect; set 2 includes genes with only treatment effect; set 3 includes genes with only interaction effect; set 4 includes genes with both genotypic and treatment effects; set 5 includes genes with both genotypic and interaction effects; set 6 includes genes with both treatment and interaction effects; and set 7 includes genes with all three effects. For genes with genotypic effects (set 1, set 4, set 5, and set 7), fold-changes were calculated by dividing the average gene expression when host is NMRI by the average gene expression when host is B6. For genes with treatment effects (set 2, set 4, set 6, and set 7), fold-changes were calculated by dividing the average gene expression from mono-association by the average gene expression from co-colonization.

### Bionomial and hypergeometric analysis of COG functional categorizations.

To assess whether the difference between the observed numbers of regulated genes belonging to each COG group was significant, we compared regulated genes in experimental and control groups (B6 versus NMRI or mono-associated versus co-colonization) belonging to each of the 18 COG categories, and calculated two *p*-values, using a binomial distribution that assumes a given treatment group is the background distribution, and then asks how likely it is to obtain the observed number of genes in that COG category from the other group. The bi-directional binomial test requires that both *p*-values be significant (*p <* 0.01) for the difference between the two groups in that COG category to be considered significant. To determine whether a treatment group is enriched with genes from particular COG category, compared to the representation of all genes within the genome, we calculated the probability of obtaining the observed number of genes in each category using a hypergeometric probability density function, with the distribution of all genes in the genome into each COG category as the background (*p <* 0.01 considered significant).

### Calculation of aggregate GeneChip expression signals for glycoside hydrolase and polysaccharide lyase genes.

Glycoside hydrolase and polysaccharide lyase predictions made by CAZy (http://afmb.cnrs-mrs.fr/CAZY/) were used for the sequenced strains of B. thetaiotaomicron (241 enzymes) and B. longum (45 enzymes). Enzyme substrate specificity was inferred using published annotations or CAZy enzyme family activity. In rare cases in which a single substrate could not be identified, genes were excluded from aggregate signal calculation. A full listing of the categorization is provided in Tables S14 and S15.

For each category of glycoside hydrolase/polysaccharide lyase activity, an aggregate signal for each sample (GeneChip) was calculated by summing the signal intensities for all genes called “present” by Affymetrix GeneChip Operating Software (GCOS) within that category. Aggregate signals were averaged across the five samples in each group. The signal with the greatest intensity between the two sample groups was defined as the maximum (100%).

### GC-MS of cecal carbohydrates.

GC-MS was performed as described previously [17]. Briefly, cecal contents were recovered from gnotobiotic mice, desiccated overnight over P_2_O_5_, and then flushed with nitrogen for 1 min. Methanolysis was performed in 250-ml methanol containing 1 M HCl (80 °C, 16 h). Samples were dried under nitrogen and then re-acetylated in 200 ml of a 1:1 mixture of pyridine/acetic anhydride (1 h at room temperature). After drying under nitrogen, de-O-acetylation was performed (200 ml of 0.5 M methanolic HCl; 65 °C for 1 h). Drying and overnight desiccation were repeated, and trimethylsilyl (TMS) derivatives were prepared by adding 100 ml of Tri-Sil reagent (Pierce Biotechnology, Rockford, Illinois, United States) at room temperature. Samples were dried (nitrogen), and extracted twice with 200-ml pure hexane. Pooled extracts were dried, resuspended in hexane, and injected onto a DB5 fused silica capillary column (30 m × 0.25 mm; J & W Scientific, Folsom California, United States) linked to a Hewlett Packard Model 5890 Series II Gas Chromatograph and Model 5971 Mass Selective Detector (Agilent Technologies, Palo Alto, California, United States). Each sample contained an internal mannitol standard. Reference standards included arabinose, rhamnose, fucose, xylose, mannose, galactose, glucose, N-acetylgalactosamine, N-acetylglucosamine, and mannitol (all from Sigma).

### LCM and expression profiling of mouse cecal epithelium.

LCM was performed on cecal samples prepared according to a protocol described in an earlier publication [38]. Epithelium was harvested from 7 μm–thick cecal cryosections using a PixCell IIe system (laser spot diameter 7.5 μm), and CapSure HS LCM caps (Arcturus, Molecular Devices, Sunnyvale, California, United States; 5,000 cells recovered per cecum). Total RNA was purified from microdissected epithelial cells using the PicoPure RNA isolation kit (Arcturus) with on-column DNase (Qiagen) treatment. RNA quality was assessed on a 2100 Bioanalyzer using an RNA 6000 Pico LabChip kit (Agilent Technologies). Intact RNA was then pooled from each group (*n* = 4 mice/group), with each animal contributing an equivalent mass (1.25 ng) of material. Duplicate 5-ng aliquots of each pooled RNA sample were amplified and biotinylated using a RiboAmp HS RNA Amplification Kit (Arcturus). The resulting aRNA targets were hybridized to Mouse Genome 430 2.0 GeneChips using standard protocols (Affymetrix). GeneChip datasets were analyzed using DNA-chip analyzer (dChip), as described above. GeneChip quality and amplification linearity were assessed using poly-adenylated spike-in control transcripts and oligo-B2 hybridization control (Affymetrix). The quality of duplicates was determined by using intensities for probe sets called “present” by Affymetrix GCOS software (yielding more than 7,800 probe sets for each set of duplicates), and plotting the respective signal intensities for the duplicate chips. Lines fitted to these plots yielded *R^2^* > 0.95 for each of the four sets of duplicates. Genes up-regulated in each colonization state (E) relative to germ free (B) were identified using the following criteria: (1) E/B > 2-fold using 90% confidence bound of fold-change; (2) E − B > 10; (3) called “present” in at least one of two E GeneChips; and (4) median replicate variation across four sample groups greater than 0.3 as assessed by standard deviation/mean.

### qRT-PCR.

Total RNA (1–10 ng), isolated from laser-captured microdissected cecal epithelium from individual mice was reverse transcribed in 20-μl reactions using the Sensiscript RT Kit (Qiagen) according to the manufacturer's protocol, together with oligo-dT_15_ (1 μM final concentration; Roche, Boulder, Colorado, United States), and random primers (10 μM final concentration; Invitrogen). An aliquot of the cDNA product (100–200 pg) was added to 25-μl qRT-PCR reaction mixtures containing SYBR Green reagent (12.5 μl; ABgene, Rochester, New York, United States) or Power SYBR Green reagent (12.5 μl; Applied Biosystems, Foster City, California, United States), and primers specific for *Isg15* (5′AGTTCTGGCTGAGCTTCGAG; 5′AACCAACACTGGCTCTGGAT) or *Reg3γ* (5′AGTGGAGCAATGCTGATGTG; 5′GATTTCGATTGTGGGGAGAA) (final concentration 800 nM). Assays were performed in triplicate using a Mx3000P QPCR System instrument (Stratagene, La Jolla, California, United States). Transcript levels within samples were normalized to ribosomal protein L32 mRNA (5′CCTCTGGTGAAGCCCAAGATC; 5′TCTGGGTTTCCGCCAGTTT), and fold changes were determined for each colonized mouse versus a pooled germ-free reference sample composed of equivalent masses of laser-captured cecal epithelial RNA (*n* = 4 germ-free mice; ΔΔC_T_ method) [38].

## Supporting Information

Figure S1 Changes in *B. thetaiotaomicron'*s Transcriptome Produced by Co-colonization of NMRI Mice with B. longum
(A) 681 B. thetaiotaomicron genes showing differential expression between cecal samples from five mono-associated mice (five left columns) and five B. longum co-colonized mice (five right columns).
(B) B. thetaiotaomicron response to B. longum categorized using COG terms. The fractional representation of differentially expressed genes within a given COG is indicated on the *x*-axis. COG categories with significant over-representation in one group compared to the other group were determined using a bi-directional comparison of a binomial distribution; an asterisk (*) indicates *p <* 0.01. Color code: yellow bars represent 230 COG-sorted B. thetaiotaomicron genes exhibiting significantly increased expression in the presence of *B. longum;* blue bars represent 153 COG-sorted B. thetaiotaomicron genes whose expression decreases significantly when B. longum is present.
(573 KB PDF)

Figure S2 B. thetaiotaomicron Genes Involved in Nutrient Binding and Acquisition Whose Expression Is Changed by the Presence of B. longum
(A–C) *SusC/D* paralogs displaying significantly different levels of expression between colonization states (FDR < 0.01) are shown. Fold-differences were determined using the average signal intensity for each group of five NMRI cecal samples. Adjacent *SusC* and *SusD* pairs are indicated by boxes.
(394 KB PDF)

Figure S3 Changes in the B. longum Transcriptome When It Co-colonizes the Ceca of NMRI Mice Containing B. thetaiotaomicron
(A) Expression pattern of 61 B. longum genes showing differential expression between cecal samples from five mono-associated mice (five left columns) and five B. thetaiotaomicron co-colonized mice (five right columns).
(B) B. longum response to B. thetaiotaomicron organized using COG terms. Functional categories with significant over-representation in one group compared to the other group were determined using a bi-directional comparison of a binomial distribution; an asterisk (*) indicates *p <* 0.01. Color code: yellow bars represent 28 COG-categorized B. longum genes showing significantly increased expression in the presence of *B. thetaiotaomicron;* blue bars represent 20 COG-sorted genes with significantly reduced expression.
(545 KB PDF)

Figure S4 Summary of ANOVA Results for the Effect of Host Genotype and Colonization State on B. thetaiotaomicron Gene Expression Venn diagram illustrating the number of B. thetaiotaomicron genes identified by ANOVA (indicated in black) that display significant host genetic background effect, colonization state effect, an interaction effect between host-strain and colonization state, or combinations of these effects. All genes and corresponding *q*-values and *p*-values for each effect can be found in Table S5, organized by gene set (indicated in red).
(427 KB PDF)

Figure S5 ANOVA-Identified Effects of Host Genotype on Changes in *B. thetaiotaomicron'*s Transcriptome (A) Expression pattern of 2,369 B. thetaiotaomicron genes identified by ANOVA as showing differential expression in the ceca of inbred NMRI versus B6 hosts, irrespective of colonization state (B. thetaiotaomicron mono-association, or *B. thetaiotaomicron/B. longum* co-colonization).
(B) The B. thetaiotaomicron host genetic background-specific response was categorized using COG terms. COGs with significant over-representation were identified using a bi-directional comparison of a binomial distribution; an asterisk (*) indicates *p <* 0.01. Color code: yellow bars represent 619 COG-categorized B. thetaiotaomicron genes with increased expression in C57BL/6J animals; blue bars represent 784 COG-categorized B. thetaiotaomicron genes with increased expression in NMRI hosts.
(1.5 MB PDF)

Figure S6 ANOVA-Identified Changes in the B. thetaiotaomicron Transcriptome Induced upon B. longum Co-colonization in Two Host Mouse Strains (A) Expression pattern of 1,806 B. thetaiotaomicron genes identified by ANOVA as differentially expressed upon co-colonization with *B. longum,* independent of host genotype.
(B) The B. thetaiotaomicron response to B. longum was categorized using COG terms. Functional categories with significant over-representation in one group compared to the other group were determined using a bi-directional comparison of a binomial distribution; an asterisk (*) indicates *p <* 0.01. Color code: yellow bars represent 494 COG-categorized B. thetaioatomicron genes with increased expression in the presence of *B. longum;* blue bars represent 535 COG-categorized B. thetaiotaomicron genes with decreased expression.
(1.7 MB PDF)

Figure S7 B. thetaiotaomicron Genes Involved in Polysaccharide Degradation, Binding, and Import That Exhibit Changed Expression in Response to Co-colonization with *B. longum*
(A) and (B) B. thetaiotaomicron glycoside hydrolases/polysaccharide lyases and *SusC/D* paralogs identified by ANOVA as displaying increased expression (A) or decreased expression (B) when colonized alone or with B. longum in NMRI and B6 hosts. CAZy glycoside hydrolase (GH) and polysaccharide lyase (PL) families are indicated for the relevant genes. Genes occurring in putative operons are boxed: glycoside hydrolases and/or *SusC/D* paralogs were considered to be in the same operon if all intervening pair-wise probability scores are ≥0.5. (http://www.cse.wustl.edu/~jbuhler/research/operons/btheta_operon_display.html) [1].
(C) CAZy family categorization of B. thetaiotaomicron glycoside hydrolases and polysaccharide lyases identified by ANOVA that change expression in response to B. longum is shown (blue bars indicate increased expression; yellow bars indicate decreased expression). Eleven B. thetaiotaomicron glycoside hydrolases display increased expression upon L. casei co-colonization (red bars). The known or predicted glycoside specificities of select families are indicated (for a full listing see Table S3).
(2.2 MB PDF)

Figure S8 Gene Networks of Cecal Epithelia Generated Using Ingenuity Pathway Analysis Software (A–C) Gene networks are shown for epithelium harvested by LCM from the ceca of NMRI mice that were mono-associated with B. thetaiotaomicron (A), mono-associated with B. longum (B), or co-colonized with *B. thetaiotaomicron/B. longum* (C). Networks were generated based on (1) genes up-regulated (red) in each colonization state relative to germ free, (2) genes called “present” (gray), and (3) genes implicated in the network based on connectivity to genes in (1) and (2). Gene symbols and fold-changes relative to germ free are indicated. TNF-α and IFN-γ are indicated by blue boxes.
(653 KB PDF)

Figure S9 Glyco-GeneChip Studies of Colonization-Associated Changes in Gene Expression in Laser Capture Microdissected Cecal Epithelium Harvested from NMRI Mice Selection criteria: a 1.2-fold or greater increase in average expression using the lower 90% confidence bound of fold-change relative to germ free, and called “present” in at least one of the two duplicate GeneChips for any of the three colonization states. Right panel, expression profiles for transcripts called “present” in at least one of the colonized RNA samples analyzed with the commercially available Mouse 430 v2.0 GeneChip. Average fold-differences between each of the three colonization states relative to germ free are shown. Note that the Pancreatitis-associated protein 1 (Pap) is represented by two probe sets on each array. Bl, B. longum mono-association; Bt, B. thetaiotaomicron mono-association, Bt/Bl; *B. thetaiotaomicron/B. longum* bi-association.
(858 KB PDF)

Table S1 Features of the *B. thetaiotaomicron/B. longum* GeneChip (31 KB PDF)

Table S2 COG Functional Categorization of B. thetaiotaomicron Genes with Increased (Red) or Decreased (Green) Expression upon Co-colonization with B. longum in NMRI Mice (853 KB PDF)

Table S3 CAZy Annotation of B. thetaiotaomicron and B. longum Genomes (420 KB PDF)

Table S4 COG Functional Categorization of B. longum Genes with Increased (Red) or Decreased (Green) Expression upon Co-colonization with B. thetaiotaomicron in NMRI Mice (126 KB PDF)

Table S5 All B. thetaiotaomicron Genes Identified by ANOVA as Displaying Significant Host Effect, Condition Effect, Interaction Effect, or Some Combination of the Three Effects Refer to Figure S4 for a Venn diagram illustrating the relationship between set number and effect(s).
(7.2 MB PDF)

Table S6 COG Functional Group–Based Categorization of B. thetaiotaomicron Genes That Are More Highly Expressed in the Ceca of NMRI Mice Compared to B6 Mice Irrespective of Colonization State (1.4 MB PDF)

Table S7 COG Functional Group–Based Categorization of B. thetaiotaomicron That Are More Highly Expressed in the Ceca of B6 Mice Compared to NMRI Mice Irrespective of Colonization State (1.4 MB PDF)

Table S8 COG Functional Group–Based Categorization of B. thetaiotaomicron Genes with Increased Expression upon Co-colonization with B. longum
(990 KB PDF)

Table S9 COG Functional Group–Based Categorization of B. thetaiotaomicron Genes That Show Decreased Expression upon Co-colonization with B. longum
(1.3 MB PDF)

Table S10 COG Functional Categorization of B. thetaiotaomicron Genes with Increased Expression upon Co-colonization with L. casei in B6 Mice (95 KB DOC)

Table S11 COG Functional Categorization of B. thetaiotaomicron Genes with Increased Expression upon Co-colonization with B. animalis in B6 Mice (104 KB DOC)

Table S12 Genes Enriched in Ingenuity Functional Categories for Host Epithelial Responses to Colonization (39 KB DOC)

Table S13 Nutrient Information for B&K Universal Autoclaved Mouse Chow Diet (40 KB DOC)

Table S14 B. thetaiotaomicron Genes Used for Aggregate Signal Determination (92 KB DOC)

Table S15 B. longum Genes Used for Aggregate Signal Determination (43 KB DOC)

### Accession Numbers

GeneChip datasets have been deposited in the Gene Expression Omnibus (GEO; http://www.ncbi.nlm.nih.gov/geo/) under accession numbers GSE5869 and GSE5870.

## Abbreviations

ANOVA: analysis of variance
CAZy: Carbohydrate Active enZymes database
CFU: colony forming units
COG: cluster of orthologous groups
FDR: false discovery rate
GC-MS: gas chromatography–mass spectrometry
LCM: laser capture microdissection
qRT-PCR: quantitative reverse transcriptase PCR
