# Supplementary material for: Genomic and Metabolic Studies of the Impact of Probiotics on a Model Gut Symbiont and Host
Source: PLoS Biol. 2006 Nov 28;4(12):e413. doi: 10.1371/journal.pbio.0040413 (PMC1661682; doi:10.1371/journal.pbio.0040413)

Sonnenburg et al.
Figure S1

A.

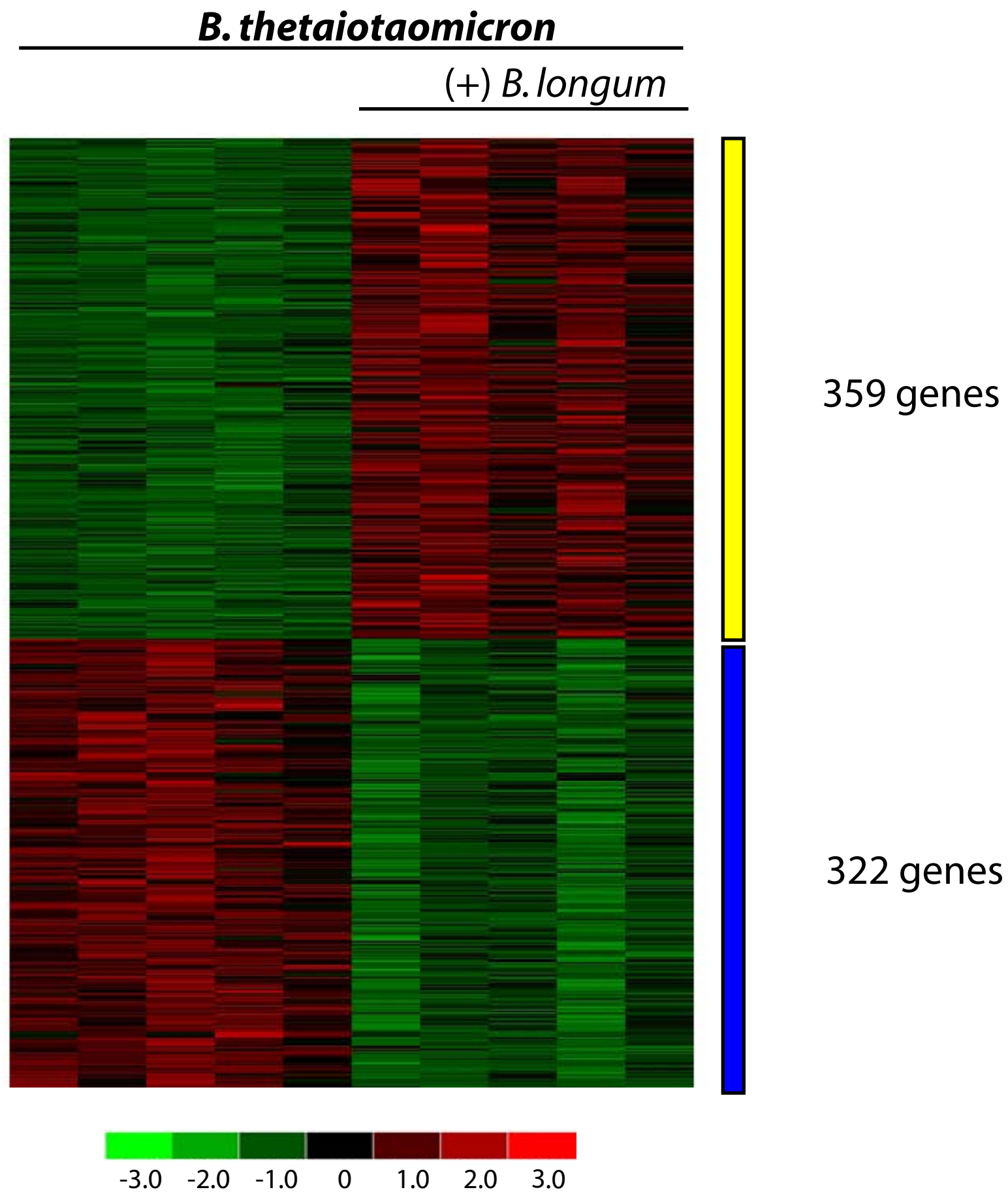


B.

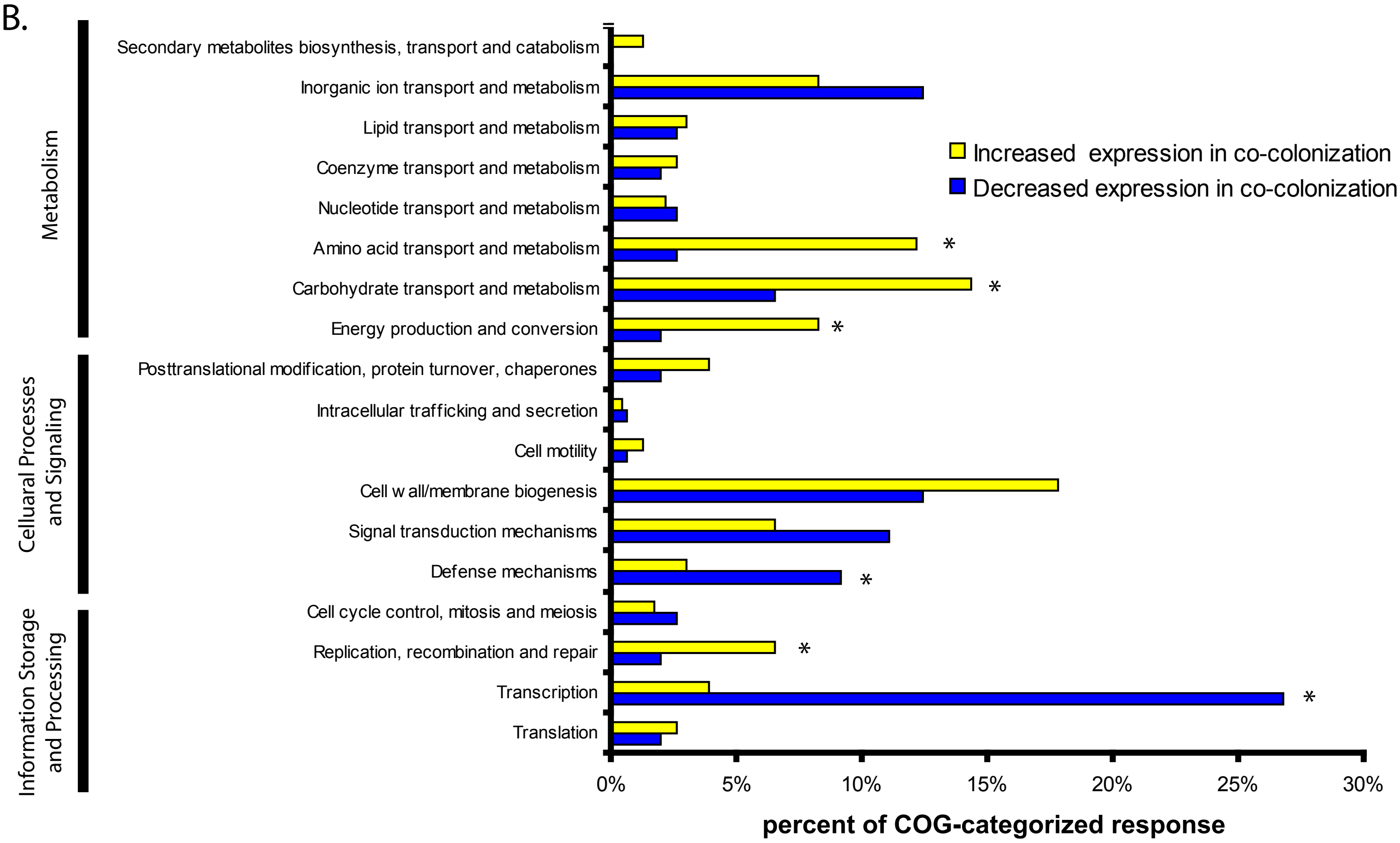

Supplement: Supplementary file 1 [file pbio.0040413.sg001.pdf]
