# Supplementary material for: Genomic and Metabolic Studies of the Impact of Probiotics on a Model Gut Symbiont and Host
Source: PLoS Biol. 2006 Nov 28;4(12):e413. doi: 10.1371/journal.pbio.0040413 (PMC1661682; doi:10.1371/journal.pbio.0040413)
Supplement: Supplementary file 2 [file pbio.0040413.sg002.pdf]

Sonnenburg et al.
Figure S2

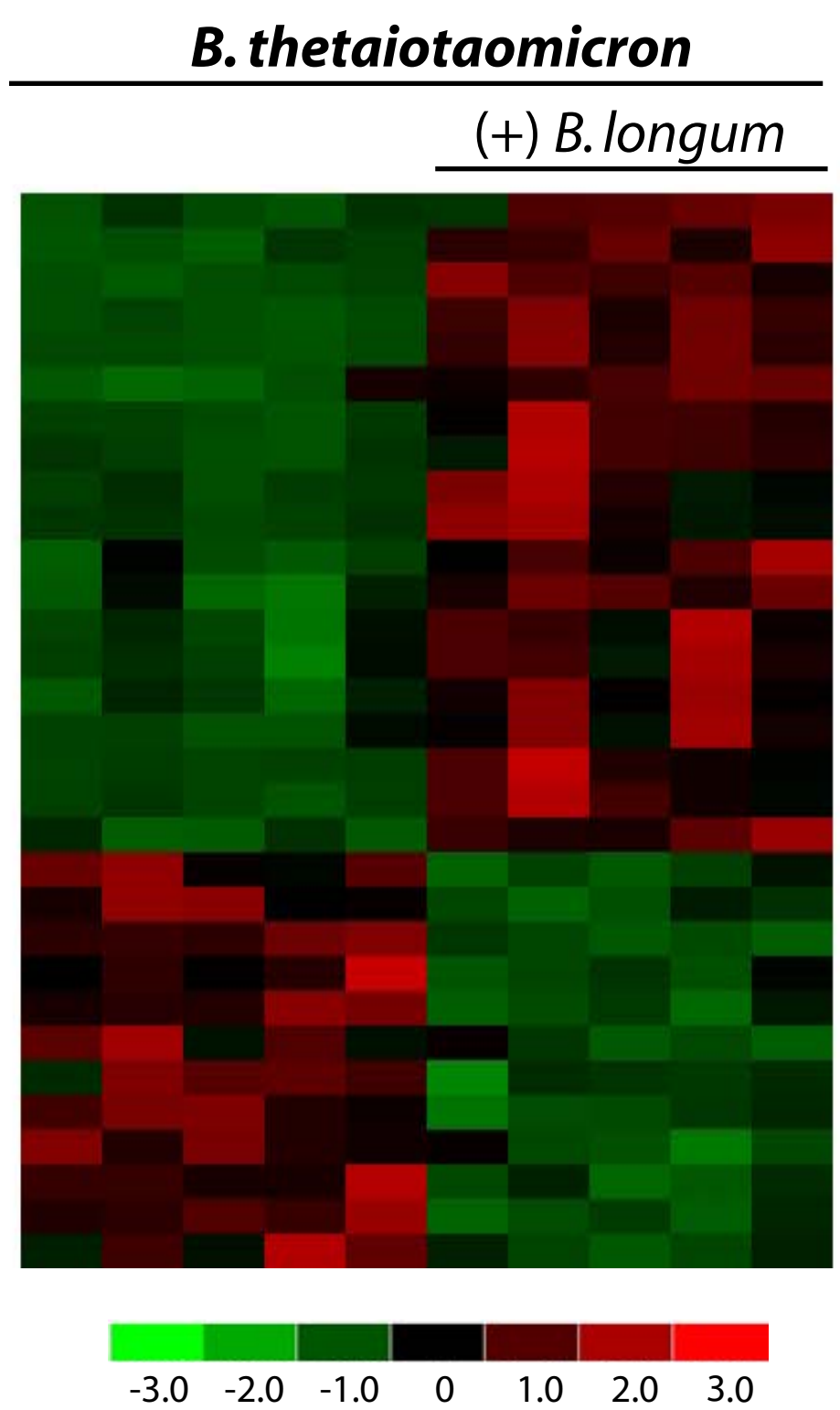

Gene ID	Description	Fold
BT0141	SusD homolog	1.8
BT0191	SusD homolog	3.6
BT0207	SusD homolog	6.0
**BT0866**	**SusD homolog**	**4.8**
**BT0867**	**SusC homolog**	**5.2**
BT1682	SusD homolog	2.7
**BT2364**	**SusC homolog**	**3.2**
**BT2365**	**SusD homolog**	**3.1**
**BT2625**	**SusD homolog**	**2.0**
**BT2626**	**SusC homolog**	**2.6**
BT3102	SusD homolog	1.8
BT3474	SusD homolog	2.1
**BT3568**	**SusD homolog**	**1.8**
**BT3569**	**SusC homolog**	**1.6**
BT3670	SusC homolog	1.7
BT4122	SusD homolog	4.0
**BT4267**	**SusC homolog**	**6.7**
**BT4268**	**SusD homolog**	**4.0**
BT4659	SusD homolog	2.2
BT1046	SusC homolog	1.8
BT1280	SusC homolog	1.8
BT2172	SusC homolog	2.7
**BT2460**	**SusD homolog**	**1.7**
**BT2461**	**SusC homolog**	**2.9**
BT2821	SusD homolog	2.3
BT2905	SusC homolog	1.4
BT3495	SusD homolog	1.4
BT3505	SusC homolog	1.7
BT3750	SusC homolog	2.1
BT4088	SusC homolog	2.3
BT4404	SusC homolog	2.1
