# Supplementary material for: Genomic and Metabolic Studies of the Impact of Probiotics on a Model Gut Symbiont and Host
Source: PLoS Biol. 2006 Nov 28;4(12):e413. doi: 10.1371/journal.pbio.0040413 (PMC1661682; doi:10.1371/journal.pbio.0040413)

Sonnenburg et al.
Figure S7

A.

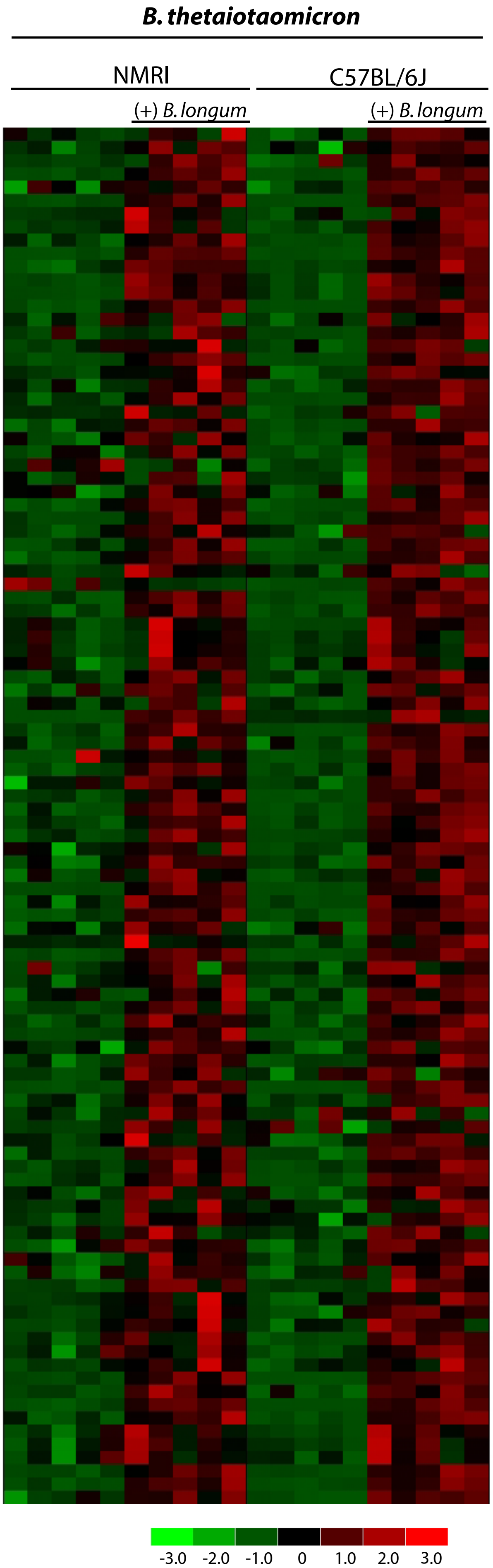

Gene ID	CAZy Family	Description
BT0065	GH27	alpha-galactosidase precursor
BT0127	GH28	transmembrane protein
BT0141		SusD homolog
BT0145	GH43	beta-xylosidase
BT0190		SusC homolog
BT0191		SusD homolog
BT0206		SusC homolog
BT0207		SusD homolog
BT0264	GH43	conserved hypothetical protein
BT0272		SusC homolog
BT0455	GH33	sialidase
BT0866		SusD homolog
BT0867		SusC homolog
BT0980	PL1	conserved hypothetical protein
BT0983	GH2	beta-galactosidase
BT0993	GH2	beta-galactosidase
BT1001	GH78	alpha-rhamnosidase
BT1024		SusD homolog
BT1025		SusC homolog
BT1440		SusC homolog
BT1538	GH73	hemagglutinin
BT1552		SusC homolog
BT1632	GH18	chitinase
BT1663	GH13	pullulanase precursor
BT1776	GH2	beta-galactosidase
BT1873	GH43	endo-arabinase
BT1878	GH92	conserved hypothetical protein
BT2112	GH43	conserved hypothetical protein
BT2199	GH92	alpha-1,2-mannosidase
BT2201		SusD homolog
BT2254	PL10	pectate lyase
BT2363		SusD homolog
BT2524	GH78	alpha-rhamnosidase
BT2620	GH97	alpha-glucosidase
BT2820		SusC homolog
BT2851	GH36	alpha-galactosidase (melibiase)
BT2858		SusD homolog
BT2892	GH93	conserved hypothetical protein
BT2894		SusC homolog
BT2895	GH43	endo-1,4-beta-xylanase
BT2898	GH43	endo-1,4-beta-xylanase D precursor
BT2912	GH43	glycosyslhydrolase
BT2913	GH88	unsaturated glucuronylhydrolase
BT2920		SusC homolog
BT2949	GH76	alpha-1,6-mannanase
BT2967		SusD homolog
BT3009	GH3	beta-glucosidase (gentiobiase)
BT3026	GH30	glycosylhydrolase, xylanase
BT3086	GH31	alpha-glucosidase II
BT3087	GH66	cycloisomaltooligosaccharide glucanotransferase
BT3089		SusD homolog
BT3096	GH51	alpha-L-arabinofuranosidase A precursor
BT3102		SusD homolog
BT3114	GH2	beta-galactosidase
BT3130	GH92	conserved hypothetical protein
BT3163	GH97	alpha-glucosidase,
BT3174		SusC homolog
BT3175		SusD homolog
BT3310		SusC homolog
BT3311		SusD homolog
BT3314	GH3	thermostable beta-glucosidase B
BT3331		SusD homolog
BT3474		SusD homolog
BT3475		SusC homolog
BT3483		SusC homolog
BT3494		SusC homolog
BT3513	GH2	beta-galactosidase
BT3516	GH43	arabinan endo-1,5-alpha-L-arabinosidase A precursor
BT3530	GH92	alpha-1,2-mannosidase
BT3633		SusC homolog
BT3656	GH43	beta-xylosidase
BT3663	GH43	beta-xylosidase
BT3664	GH97	alpha-glucosidase
BT3670		SusC homolog
BT3671		SusD homolog
BT3702		SusC
BT3773	GH92	conserved hypothetical protein
BT3782	GH76	hydrolase
BT3952		SusC homolog
BT3956	GH29	conserved hypothetical protein
BT3958		SusC homolog
BT3983		SusC homolog
BT3990	GH92	alpha-1,2-mannosidase
BT4038		SusD homolog
BT4050	GH2	beta-galactosidase
BT4073	GH92	conserved hypothetical protein
BT4093	GH92	conserved hypothetical protein
BT4095	GH43	beta-xylosidase (1,4-beta-D-xylan xylosidase)
BT4119	PL1	conserved hypothetical protein
BT4121		SusC homolog
BT4146	GH28	exo-poly-alpha-D-galacturonosidase precursor
BT4151	GH2	beta-glucuronidase
BT4155	GH28	conserved hypothetical protein
BT4160	GH35	beta-galactosidase precursor
BT4181	GH2	beta-galactosidase
BT4268		SusD homolog
BT4394	GH20	beta-hexosaminidase precursor
BT4659		SusD homolog
BT4667	GH2	beta-galactosidase
BT4668	GH53	arabinogalactan endo-1,4-beta-galactosidase
BT4671		SusC homolog
BT4675	PL13	heparin lyase I precursor
BT4714	GH3	periplasmic beta-glucosidase precursor, xylosidase/arabinosidase
BT4724		SusC homolog

Sonnenburg et al.
Figure S7

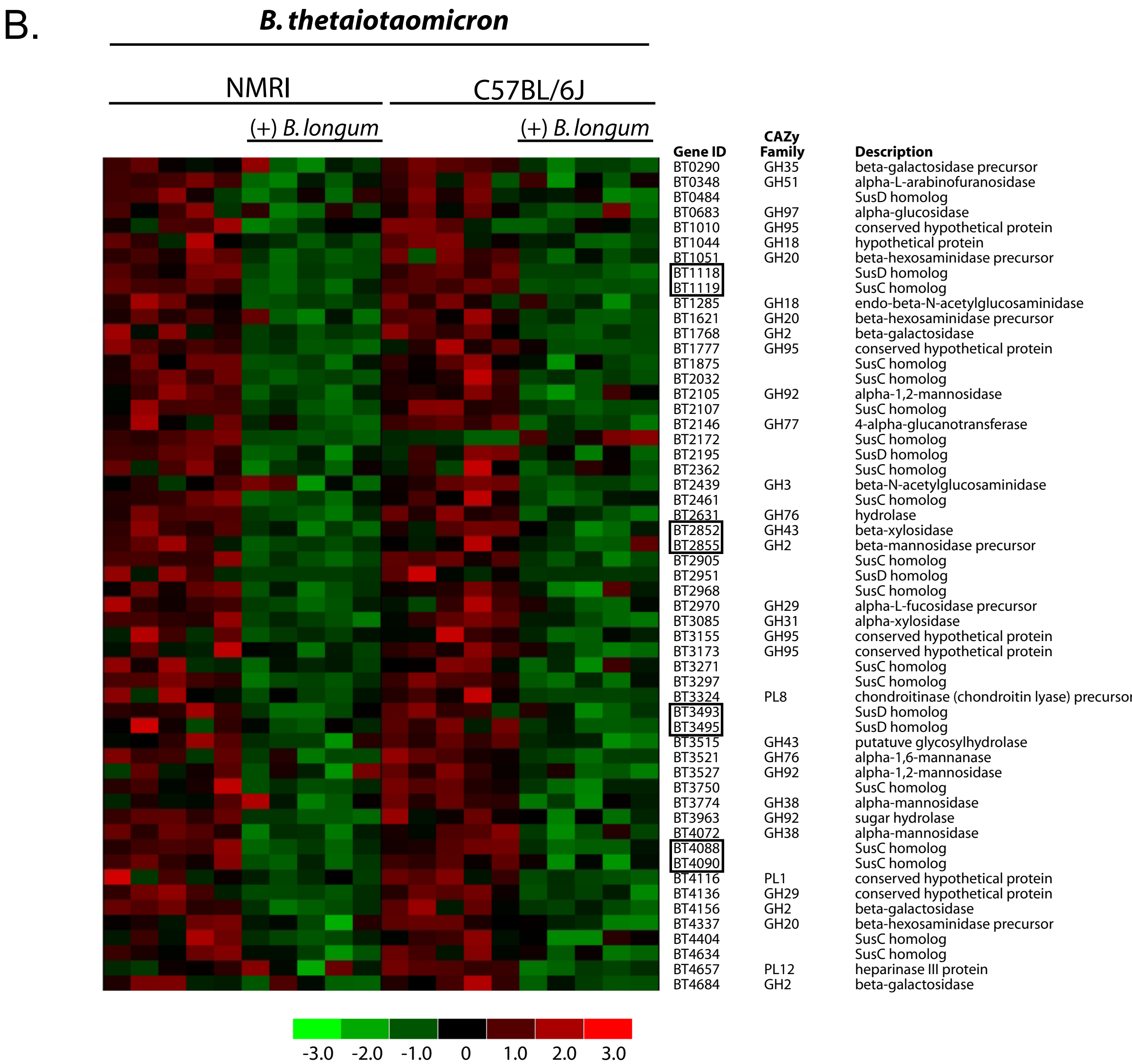

Reference

1. Westover BP, Buhler JD, Sonnenburg JL, Gordon JI (2005) Operon prediction without a training set. Bioinformatics 21: 880-888.

Sonnenburg et al.
Figure S7

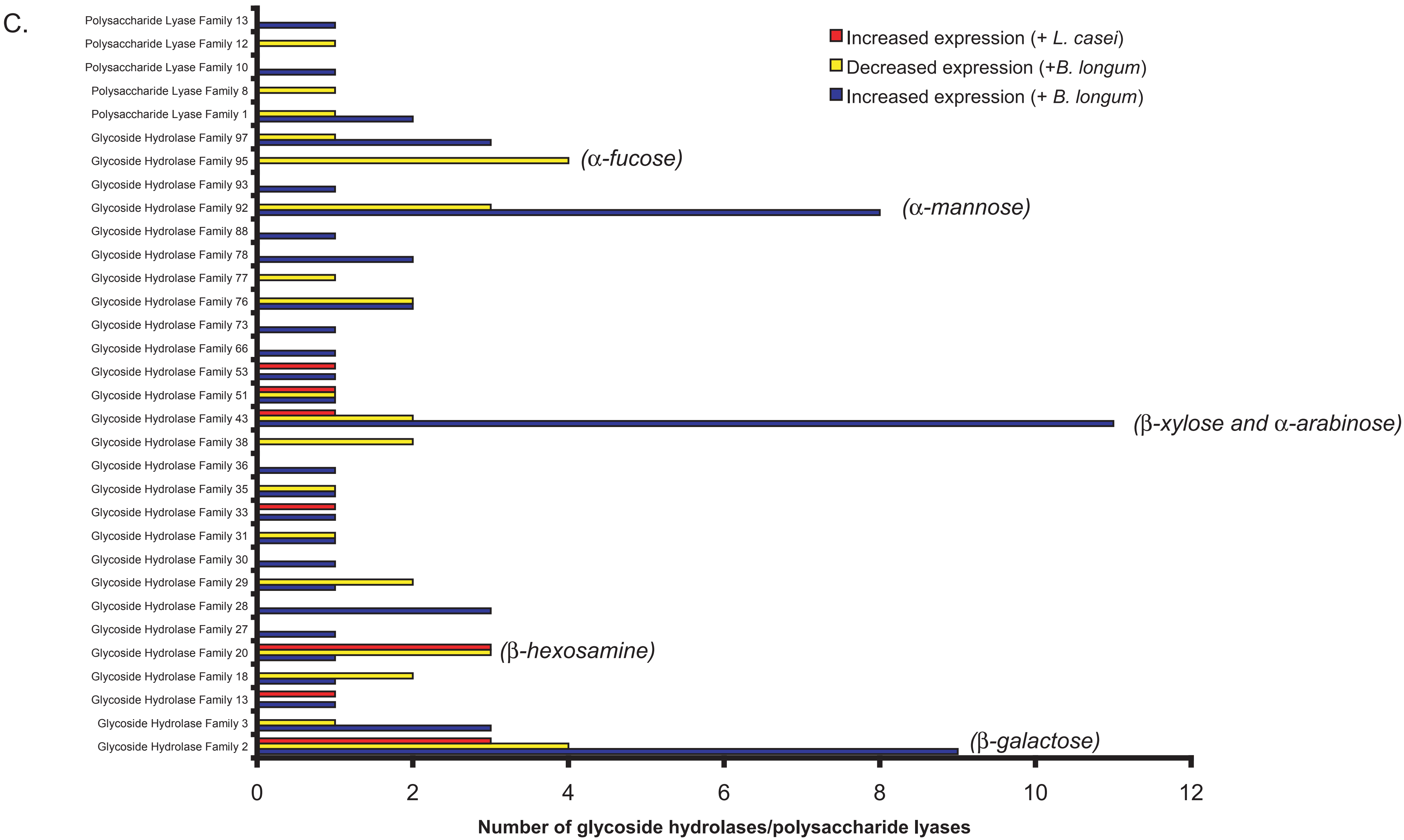

Supplement: Supplementary file 7 [file pbio.0040413.sg007.pdf]
