# Supplementary material for: Genomic and Metabolic Studies of the Impact of Probiotics on a Model Gut Symbiont and Host
Source: PLoS Biol. 2006 Nov 28;4(12):e413. doi: 10.1371/journal.pbio.0040413 (PMC1661682; doi:10.1371/journal.pbio.0040413)

Sonnenburg et al.
Figure S8

A.

***B. thetaiotaomicron* mono-association**

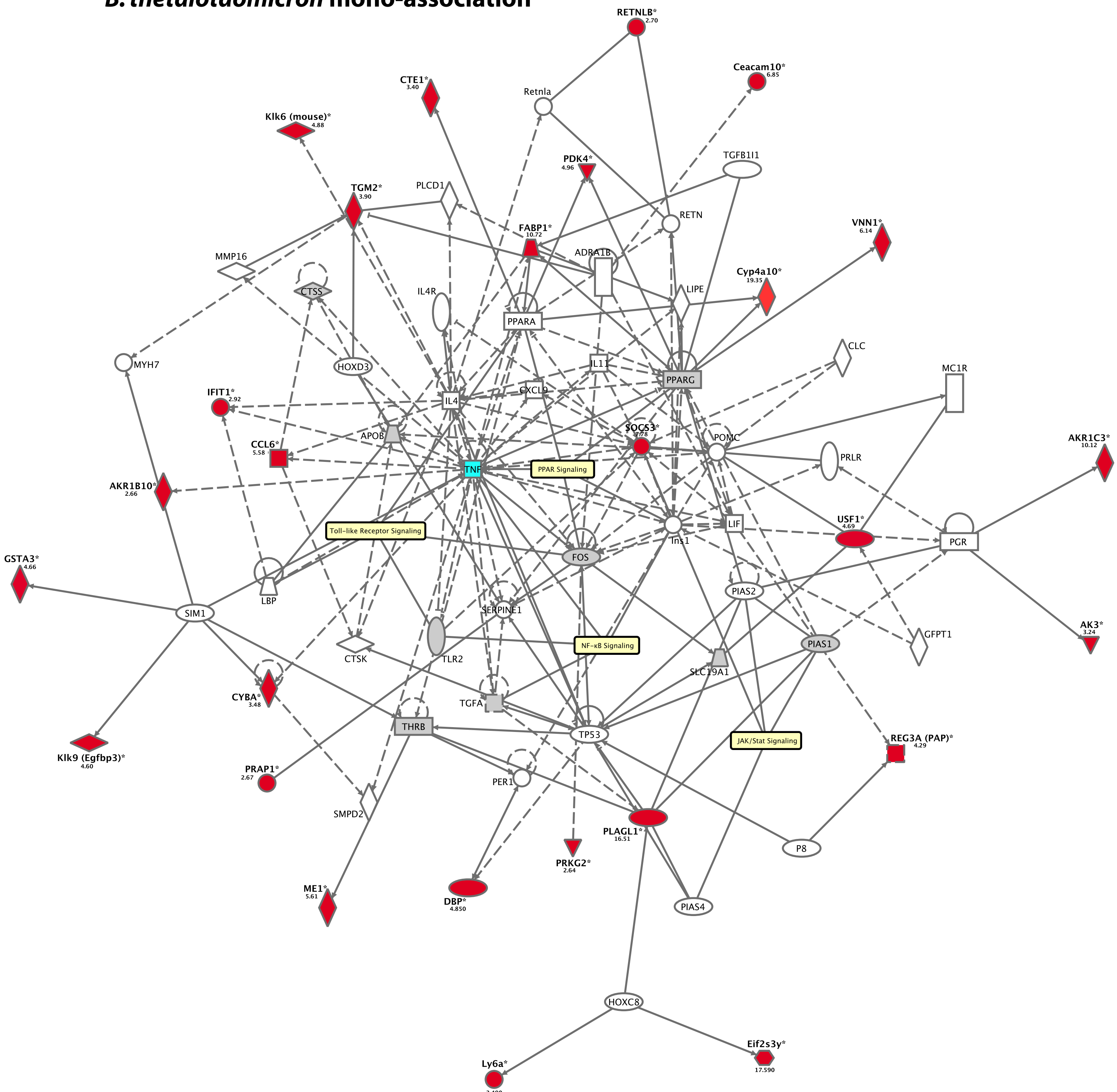

Sonnenburg et al.
Figure S8

B. ***B. longum* mono-association**

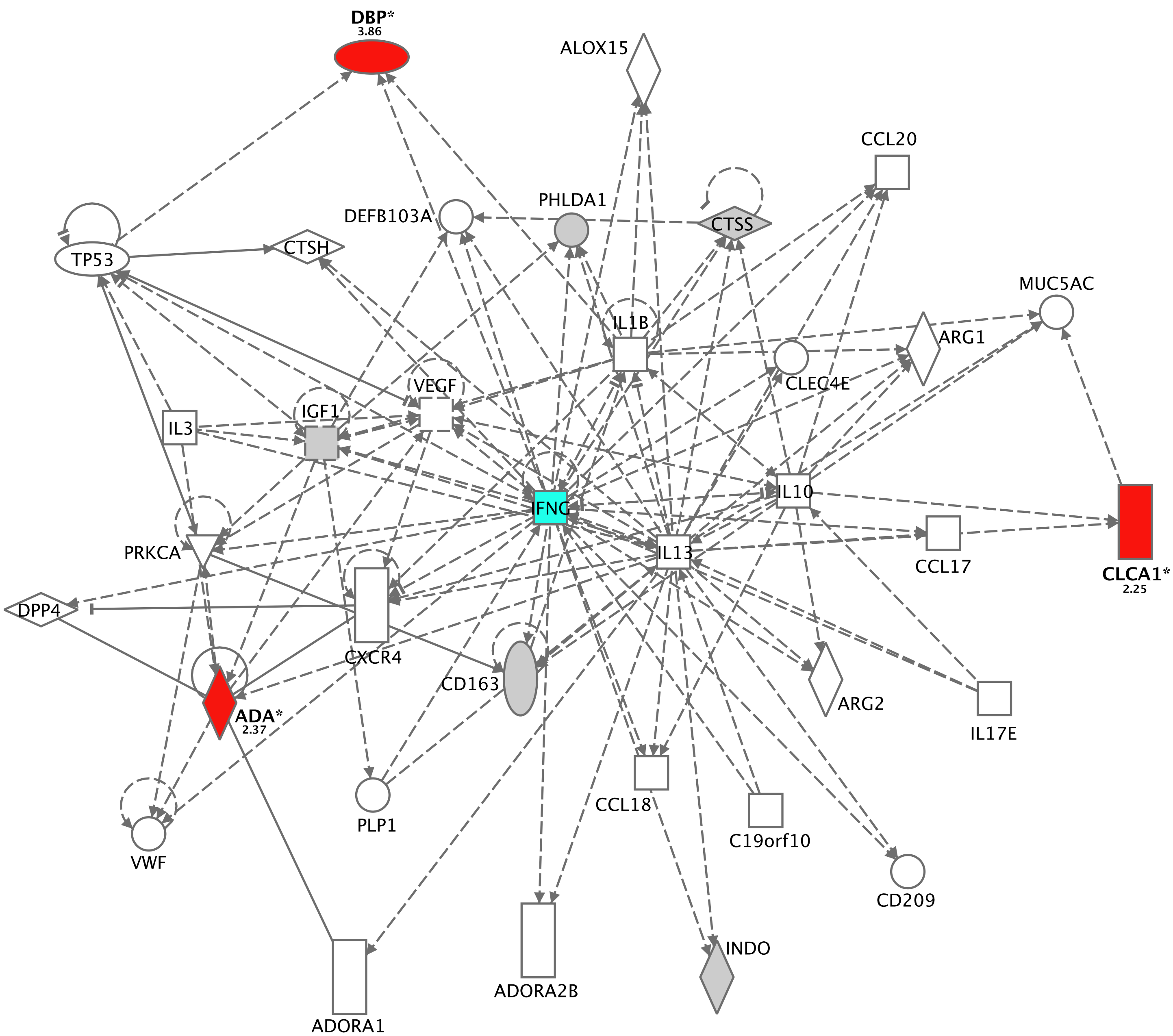

Sonnenburg et al.
Figure S8

C. ***B. thetaiotaomicron/B. longum* bi-association**

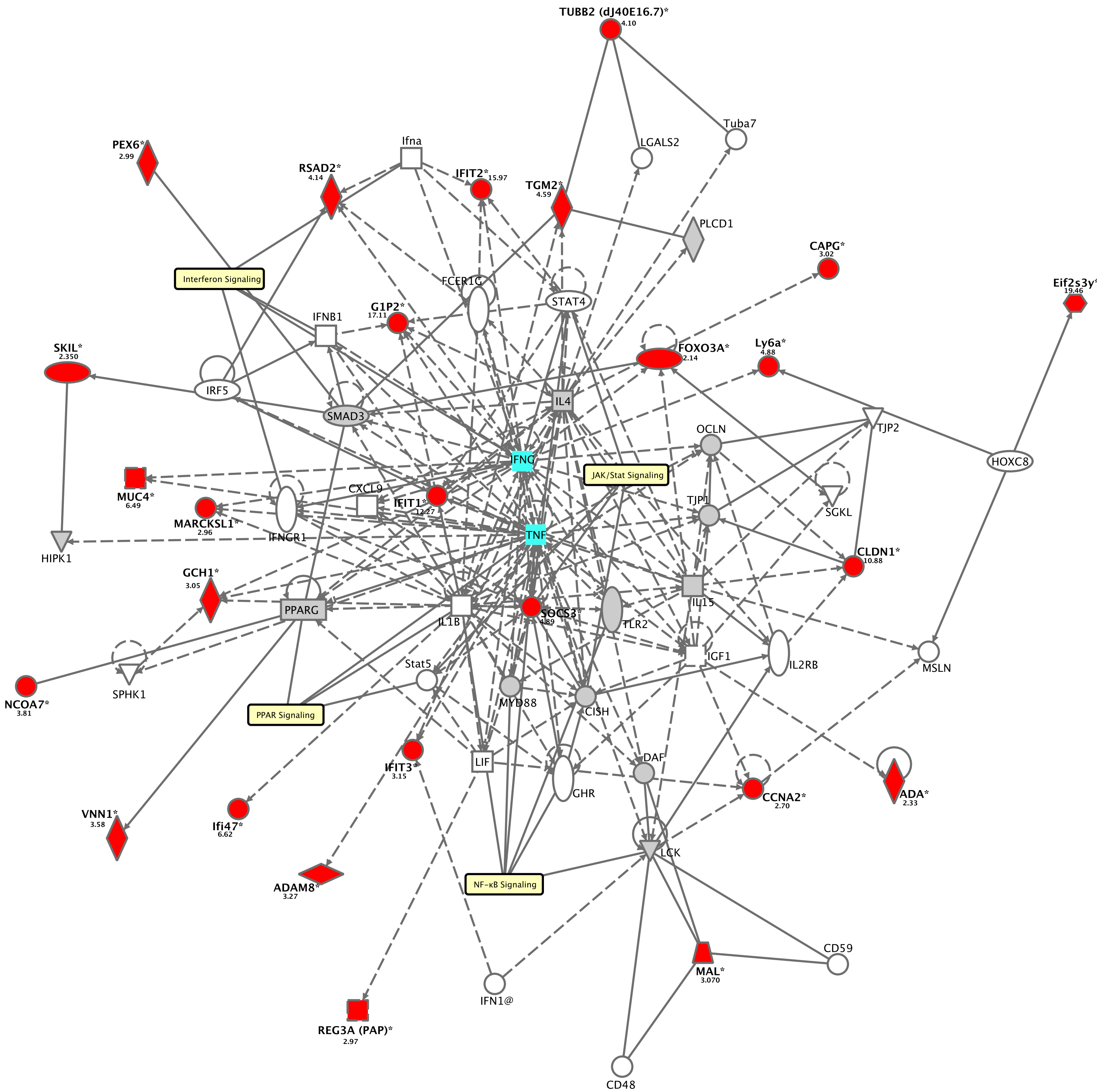

Supplement: Supplementary file 8 [file pbio.0040413.sg008.pdf]
