# Supplementary material for: Genomic and Metabolic Studies of the Impact of Probiotics on a Model Gut Symbiont and Host
Source: PLoS Biol. 2006 Nov 28;4(12):e413. doi: 10.1371/journal.pbio.0040413 (PMC1661682; doi:10.1371/journal.pbio.0040413)
Supplement: Supplementary file 9 [file pbio.0040413.sg009.pdf]

Sonnenburg et al.
Figure S9

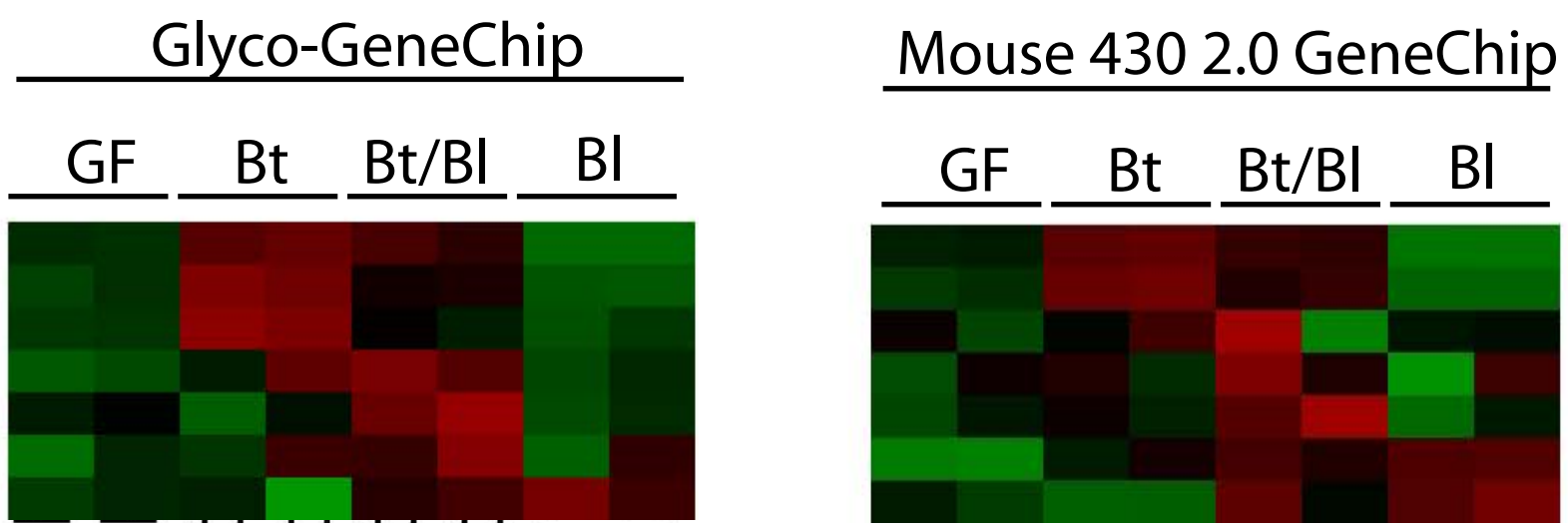

fold change relative to GF

	Bt		BtBl		Bl	
	Glyco	430	Glyco	430	Glyco	430
Pancreatitis-associated protein 1 (Pap)	5.8	4.3	3.1	3.0	-5.9	-34.9
Pancreatitis-associated protein 1 (Pap)	3.1	2.3	2.6	1.8	-20.1	-29.8
b4GalT3 (B4galt3)	1.6	1.1	1.1	1.1	-1.1	1.0
INF-gamma R1 (Ifngr1)	1.4	1.1	1.7	1.3	1.1	-1.0
Amphiregulin (Areg)	-1.2	1.3	1.4	2.3	-1.3	-1.2
ST6GalNAc VI (St6galnac6)	1.4	1.4	1.8	1.6	1.2	1.7
UDP-Gal-4-Epimerase (Gale)	-1.2	-1.1	1.3	1.2	1.4	1.4

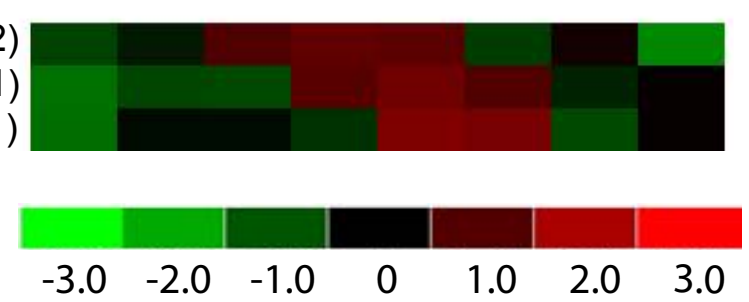

	Bt	BtBl	Bl
Gln-fructose-6-P transaminase 2 (Gfpt2)	1.8	1.4	-1.1
ST6GalNAc I (St6galnac1)	1.5	2.0	1.4
GNT4A (Mgat4a)	1.2	2.1	1.1
