# Supplementary material for: Genomic and Metabolic Studies of the Impact of Probiotics on a Model Gut Symbiont and Host
Source: PLoS Biol. 2006 Nov 28;4(12):e413. doi: 10.1371/journal.pbio.0040413 (PMC1661682; doi:10.1371/journal.pbio.0040413)
Supplement: Supplementary file 10 [file pbio.0040413.st001.doc]

Table S1. Features of the *B. thetaiotaomicron/B. longum* GeneChip

| Functional category | Naming prefix of probesets | No. of genes (probesets) represented | No. of probe pairs | Average no. of probe pairs per probeset |
| --- | --- | --- | --- | --- |
| Control sequences | AFFX | 51 | 831 | 16.3 |
| *Bt* chromosomal genes^a^ | BT | 4719 | 61737 | 13 |
| *Bt* genes on p5482^b^ | p5482 | 38 | 494 | 13 |
| *Bt*  tRNA genes | tRNA | 36 | 468 | 13 |
| *Bl* genes^c^ | AAN | 1722 | 22451 | 13 |

^a^Based on results reported in ref. Xu et al., 2003. GenBank accession number AE015928

^b^GenBank accession number AY171301

^c^ Based on ref. Schell *et al.* 2002. GenBank accession number AE014295
