# Supplementary material for: Genomic and Metabolic Studies of the Impact of Probiotics on a Model Gut Symbiont and Host
Source: PLoS Biol. 2006 Nov 28;4(12):e413. doi: 10.1371/journal.pbio.0040413 (PMC1661682; doi:10.1371/journal.pbio.0040413)
Supplement: Supplementary file 11 [file pbio.0040413.st002.doc]

Table S2. COG functional categorization of *B. thetaiotaomicron* genes with increased (red) or decreased (green) expression upon co-colonization with *B. longum* in NMRI mice.

| **COG function** | **Gene** | **Description** | **Fold difference in expression (co-colonization versus mono-association)** |
| --- | --- | --- | --- |
| Translation |  |  |  |
|  | BT0835 | tRNA-guanine transglycosylase | 3.3 |
|  | BT0865 | putative chitobiase | 3.8 |
|  | BT2730 | 30S ribosomal protein S7 | 1.9 |
|  | BT3888 | putative tRNA (5-methylaminomethyl-2-thiouridylate) methyltransferase | 1.7 |
|  | BT4332 | hypothetical protein | 1.5 |
|  | BT4429 | putative pteridine-dependent dioxygenase | 2.3 |
|  |  |  |  |
|  | BT1454 | putative peptide chain release factor | -2.0 |
|  | BT3237 | hypothetical protein | -1.5 |
|  | BT3879 | putative translation initiation factor SUI1 | -1.5 |
| Transcription |  |  |  |
|  | BT0223 | two-component system response regulator | 1.9 |
|  | BT0462 | putative transcriptional regulator | 2.9 |
|  | BT1130 | hypothetical protein | 1.9 |
|  | BT1595 | transcription termination factor rho | 3.2 |
|  | BT1656 | putative transcriptional regulator | 6.3 |
|  | BT1968 | transcriptional regulator | 2.3 |
|  | BT2733 | DNA-directed RNA polymerase beta' chain | 2.2 |
|  | BT3052 | transcriptional regulator | 1.7 |
|  | BT3258 | two-component system response regulator | 2.2 |
|  |  |  |  |
|  | BT0008 | hypothetical protein | -2.2 |
|  | BT0248 | RNA polymerase ECF-type sigma factor | -1.4 |
|  | BT0326 | putative RNA polymerase ECF-type sigma factor | -6.0 |
|  | BT0582 | hypothetical protein | -1.6 |
|  | BT0583 | hypothetical protein | -1.6 |
|  | BT0596 | putative transcriptional regulator | -1.9 |
|  | BT0635 | putative transcriptional regulator | -1.7 |
|  | BT0636 | putative transcriptional regulator | -1.5 |
|  | BT0728 | transcriptional regulator | -1.5 |
|  | BT0812 | putative transcriptional regulator | -1.7 |
|  | BT0890 | hypothetical protein | -1.5 |
|  | BT1076 | two-component system response regulator | -2.1 |
|  | BT1404 | putative transcriptional regulator | -1.7 |
|  | BT1559 | RNA polymerase ECF-type sigma factor | -1.4 |
|  | BT1568 | hypothetical protein | -1.8 |
|  | BT1572 | RNA polymerase ECF-type sigma factor | -2.0 |
|  | BT1817 | RNA polymerase ECF-type sigma factor | -1.7 |
|  | BT2044 | RNA polymerase ECF-type sigma factor | -1.8 |
|  | BT2103 | transcriptional regulator | -1.6 |
|  | BT2184 | RNA polymerase ECF-type sigma factor | -2.4 |
|  | BT2198 | RNA polymerase ECF-type sigma factor | -2.2 |
|  | BT2492 | RNA polymerase ECF-type sigma factor | -2.6 |
|  | BT2546 | transcriptional regulator | -1.7 |
|  | BT2565 | transcription elongation factor greA | -1.4 |
|  | BT2689 | hypothetical protein | -1.6 |
|  | BT2778 | RNA polymerase ECF-type sigma factor | -6.1 |
|  | BT2975 | putative nicotinate mononucleotide:5,6-dimethylbenzimidazole phosphoribosyltransferase | -1.8 |
|  | BT3153 | hypothetical protein | -1.6 |
|  | BT3190 | putative RNA polymerase ECF-type sigma factor | -5.2 |
|  | BT3396 | RNA polymerase ECF-type sigma factor | -2.2 |
|  | BT3517 | RNA polymerase ECF-type sigma factor | -1.6 |
|  | BT3899 | transcriptional regulator | -1.8 |
|  | BT3974 | hypothetical protein | -1.7 |
|  | BT4035 | hypothetical protein | -2.0 |
|  | BT4201 | hypothetical protein | -1.6 |
|  | BT4418 | hypothetical protein | -1.7 |
|  | BT4515 | hypothetical protein | -1.6 |
|  | BT4571 | RNA polymerase ECF-type sigma factor | -5.3 |
|  | BT4647 | RNA polymerase ECF-type sigma factor | -6.5 |
|  | BT4720 | putative RNA polymerase ECF-type sigma factor | -2.1 |
|  | BT4733 | hypothetical protein | -1.8 |
| Replication, recombination and repair |  |  |  |
|  | BT0244 | putative exonuclease | 2.3 |
|  | BT0252 | transcription-repair coupling factor | 1.7 |
|  | BT0899 | DNA gyrase subunit A | 1.5 |
|  | BT1054 | ATP-dependent helicase | 1.8 |
|  | BT1205 | putative ATPase, AAA family | 2.2 |
|  | BT1361 | DNA repair protein recN (Recombination protein N) | 3.1 |
|  | BT1671 | endonuclease III | 1.8 |
|  | BT2056 | hypothetical protein | 1.6 |
|  | BT2073 | putative helicase | 2.3 |
|  | BT2890 | transposase | 2.2 |
|  | BT2999 | helicase IV | 1.4 |
|  | BT3924 | ATP-dependent DNA helicase recG | 1.6 |
|  | BT4255 | DNA replication and repair protein RecF, ABC family ATPase | 3.6 |
|  | BT4343 | hypothetical protein | 2.8 |
|  | BT4651 | endonuclease IV | 2.0 |
|  |  |  |  |
|  | BT1498 | A/G-specific adenine glycosylase | -1.6 |
|  | BT3820 | putative DNA polymerase III, delta subunit | -1.5 |
|  | BT3860 | hypothetical protein | -1.8 |
| Cell cycle control, mitosis and meiosis |  |  |  |
|  | BT0841 | hypothetical protein | 2.1 |
|  | BT1376 | putative ATP-binding protein involved in cell division | 9.5 |
|  | BT1676 | Maf protein | 1.5 |
|  | BT3445 | cell division protein FtsA | 6.6 |
|  |  |  |  |
|  | BT2458 | putative pyridine nucleotide-disulphide oxidoreductase | -1.6 |
|  | BT3166 | hypothetical protein | -1.6 |
|  | BT3437 | hypothetical protein | -2.3 |
|  | BT3754 | hypothetical protein | -1.6 |
| Defense mechanisms |  |  |  |
|  | BT0301 | ATP-binding transport protein natA (Na+ ABC transporter) | 1.9 |
|  | BT0563 | putative ABC transporter ATP-binding protein or permease protein | 27.3 |
|  | BT0956 | conserved hypothetical protein, putative transmembrane protein | 1.8 |
|  | BT1519 | N-acetylmuramoyl-L-alanine amidase | 2.5 |
|  | BT3804 | putative transport protein, multidrug efflux protein | 1.6 |
|  | BT4326 | ABC transporter ATP-binding protein | 1.7 |
|  | BT4543 | putative type I restriction enzyme specificity protein | 2.0 |
|  |  |  |  |
|  | BT0073 | putative ABC transporter, ATP-binding protein | -1.6 |
|  | BT0562 | putative ABC transporter ATP-binding protein | -1.7 |
|  | BT0694 | ABC transporter, ATP-binding protein | -2.8 |
|  | BT0695 | ABC transporter, permease protein | -2.0 |
|  | BT1214 | putative ABC transporter ATP-binding protein | -3.3 |
|  | BT1215 | ABC transporter, permease protein | -4.2 |
|  | BT1306 | putative ABC transporter ATP-binding protein | -2.7 |
|  | BT1532 | ABC transporter permease | -2.9 |
|  | BT1533 | hypothetical protein | -2.5 |
|  | BT1535 | ABC transporter ATP-binding protein | -2.6 |
|  | BT2494 | ABC transporter, ATP-binding protein | -2.0 |
|  | BT3200 | putative N-acetylmuramoyl-L-alanine amidase | -1.8 |
|  | BT4522 | Type I restriction-modification enzyme | -2.0 |
|  | BT4540 | putative Type I restriction enzyme MjaXP specificity protein | -2.6 |
| Signal transduction mechanisms |  |  |  |
|  | BT0524 | putative two-component system response regulator | 1.7 |
|  | BT0616 | hypothetical protein | 11.3 |
|  | BT1183 | Hybrid two-component system | 1.8 |
|  | BT1264 | two-component system sensor histidine kinase | 2.2 |
|  | BT1545 | conserved hypothetical protein, putative regulatory protein with a cAMP-binding domain, | 1.7 |
|  | BT2023 | two-component system sensor histidine kinase | 2.4 |
|  | BT2132 | hypothetical protein | 4.7 |
|  | BT2366 | hypothetical protein | 1.5 |
|  | BT2860 | Hybrid two-component system | 2.0 |
|  | BT2897 | Hybrid two-component system | 2.9 |
|  | BT3800 | Hybrid two-component system | 4.7 |
|  | BT3951 | transcriptional regulator | 6.3 |
|  | BT4124 | Hybrid two-component system | 1.6 |
|  | BT4375 | transcriptional regulator | 1.4 |
|  | BT4552 | two-component system sensor histidine kinase | 1.5 |
|  |  |  |  |
|  | BT0324 | hypothetical protein | -4.0 |
|  | BT0700 | GTP pyrophosphokinase | -1.5 |
|  | BT0928 | two-component system response regulator | -2.0 |
|  | BT1075 | putative two-component system sensor protein, but no histidine kinase domain | -2.2 |
|  | BT1123 | transcriptional regulator | -1.7 |
|  | BT1216 | putative two-component system sensor protein, without kinase domain | -2.3 |
|  | BT1334 | PhoH-like protein | -1.7 |
|  | BT1469 | two-component system response regulator | -1.9 |
|  | BT1528 | two-component system sensor histidine kinase | -1.8 |
|  | BT1810 | GAF domain-containing protein, involved in signal transduction | -2.1 |
|  | BT2628 | Hybrid two-component system | -2.2 |
|  | BT2923 | Hybrid two-component system | -2.0 |
|  | BT3150 | transcriptional regulator | -2.1 |
|  | BT3660 | transcriptional regulator | -1.5 |
|  | BT3786 | Hybrid two-component system | -2.0 |
|  | BT3801 | rteB, two-component system response regulator | -1.8 |
|  | BT4178 | Hybrid two-component system | -1.5 |
| Cell wall/membrane biogenesis |  |  |  |
|  | BT0051 | putative glycosyltransferase | 2.4 |
|  | BT0294 | hypothetical protein | 5.5 |
|  | BT0297 | putative outer membrane lipoprotein silC precursor | 1.9 |
|  | BT0298 | cation efflux system protein (outer membrane protein) | 3.1 |
|  | BT0306 | outer membrane protein oprM precursor | 1.6 |
|  | BT0523 | putative glycosyltransferase | 3.5 |
|  | BT0554 | glucosamine--fructose-6-phosphate aminotransferase | 7.3 |
|  | BT0656 | hypothetical protein | 1.8 |
|  | BT0743 | penicillin-binding protein 1A (PBP-1a) | 1.9 |
|  | BT0954 | hypothetical protein | 1.5 |
|  | BT1163 | hypothetical protein | 1.6 |
|  | BT1164 | hypothetical protein | 2.0 |
|  | BT1165 | hypothetical protein | 6.2 |
|  | BT1167 | hypothetical protein | 2.2 |
|  | BT1180 | putative glycosyltransferase | 2.0 |
|  | BT1452 | hypothetical protein | 1.8 |
|  | BT1472 | hypothetical protein | 1.9 |
|  | BT1501 | major outer membrane protein OmpA | 1.7 |
|  | BT1644 | putative CPS biosynthesis glycosyltransferase | 3.5 |
|  | BT1645 | putative glycosyltransferase | 9.8 |
|  | BT1646 | putative glycosyltransferase | 8.0 |
|  | BT1648 | putative glycosyltransferase | 4.5 |
|  | BT1649 | hypothetical protein | 6.8 |
|  | BT1650 | putative teichoic acid biosynthesis protein F | 8.9 |
|  | BT1654 | polysialic acid transport protein kpsD precursor | 7.4 |
|  | BT2001 | membrane-associated zinc metalloprotease | 2.7 |
|  | BT2003 | putative membrane peptidase | 2.4 |
|  | BT2681 | alginate O-acetylation protein | 1.7 |
|  | BT2945 | UDP-N-acetyl-D-mannosamine dehydrogenase | 1.6 |
|  | BT3306 | putative sugar isomerase | 1.5 |
|  | BT3312 | glucosylceramidase precursor | 3.6 |
|  | BT3388 | hypothetical protein | 1.8 |
|  | BT3690 | putative membrane protein | 1.7 |
|  | BT3996 | putative peptidase | 1.5 |
|  | BT4206 | UDP-3-O-[3-hydroxymyristoyl] N-acetylglucosamine deacetylase | 1.9 |
|  | BT4282 | glycosyltransferase | 2.4 |
|  | BT4283 | hypothetical protein | 2.8 |
|  | BT4391 | putative metalloendopeptidase | 1.8 |
|  | BT4487 | putative membrane-bound lytic murein transglycosylase | 1.4 |
|  | BT4576 | hypothetical protein | 2.3 |
|  | BT4703 | chloromuconate cycloisomerase | 1.8 |
|  |  |  |  |
|  | BT0251 | dolichol-phosphate mannosyltransferase | -1.4 |
|  | BT0381 | capsular polysaccharide biosynthesis protein capD | -1.7 |
|  | BT0599 | UDP-glucose 6-dehydrogenase | -2.0 |
|  | BT0602 | UDP-N-acetyl-D-mannosaminuronic acid dehydrogenase | -1.6 |
|  | BT0610 | lipopolysaccharide biosynthesis protein, putative glycosyltransferase | -2.1 |
|  | BT0611 | glycosyltransferase | -2.2 |
|  | BT0612 | putative aminotransferase | -1.6 |
|  | BT1212 | outer membrane efflux protein | -5.6 |
|  | BT1213 | periplasmic component of efflux system | -3.9 |
|  | BT1536 | ABC transporter permease | -2.0 |
|  | BT1922 | putative N-acetylmuramoyl-L-alanine amidase | -1.7 |
|  | BT2117 | outer membrane protein, putative | -1.5 |
|  | BT2762 | TonB | -1.7 |
|  | BT2781 | putative mannose-1-phosphate guanylyltransferase | -2.2 |
|  | BT3207 | hypothetical protein | -1.6 |
|  | BT3366 | putative glycosyltransferase yibD | -1.5 |
|  | BT3724 | cationic outer membrane protein precursor | -1.6 |
|  | BT3807 | hypothetical protein | -1.5 |
|  | BT4481 | outer membrane protein | -1.7 |
| Cell motility |  |  |  |
|  | BT0142 | hypothetical protein | 1.4 |
|  | BT0845 | hypothetical protein | 1.5 |
|  | BT1538 | hemagglutinin | 1.7 |
|  |  |  |  |
|  | BT3413 | hypothetical protein | -1.5 |
| Intracellular trafficking and secretion |  |  |  |
|  | BT4103 | putative Sec-independent protein translocase | 1.8 |
|  |  |  |  |
|  | BT1072 | hypothetical protein | -14.2 |
| Posttranslational modification, protein turnover, chaperones |  |  |  |
|  | BT0212 | protease | 9.4 |
|  | BT0237 | hypothetical protein | 1.8 |
|  | BT1055 | pyruvate formate-lyase activating enzyme | 1.7 |
|  | BT1199 | putative protease | 1.7 |
|  | BT2577 | hypothetical protein | 2.1 |
|  | BT2846 | magnesium chelatase, subunit ChlI | 3.7 |
|  | BT3408 | conserved hypothetical protein, putative ABC transporter permease component | 1.4 |
|  | BT4007 | AAA-metalloprotease FtsH, with ATPase domain | 1.8 |
|  | BT4371 | peptidyl-prolyl cis-trans isomerase | 2.0 |
|  |  |  |  |
|  | BT0987 | putative cytochrome C-type biogenesis protein | -1.7 |
|  | BT3418 | putative thiol:disulfide interchange protein | -2.1 |
|  | BT4194 | putative integral membrane protein | -1.7 |
| Energy production and conversion |  |  |  |
|  | BT0174 | hypothetical protein | 1.7 |
|  | BT0331 | ketoisovalerate oxidoreductase subunit vorB | 1.7 |
|  | BT0430 | indolepyruvate oxidoreductase subunit iorA | 1.9 |
|  | BT0620 | Na+-transporting NADH:ubiquinone oxidoreductase, Electron transport complex protein rnfG | 2.6 |
|  | BT0844 | hypothetical protein | 1.7 |
|  | BT1207 | glycerate dehydrogenase (NADH-dependent) | 2.1 |
|  | BT1296 | V-type ATP synthase subunit I | 1.7 |
|  | BT1297 | V-type ATP synthase subunit D | 1.8 |
|  | BT1299 | V-type ATP synthase subunit A | 1.9 |
|  | BT1451 | hypothetical protein | 1.9 |
|  | BT1689 | oxaloacetate decarboxylase beta chain | 2.0 |
|  | BT1935 | hypothetical protein | 2.1 |
|  | BT2836 | 2-oxoglutarate synthase subunit korA | 1.7 |
|  | BT3055 | succinate dehydrogenase iron-sulfur protein | 1.6 |
|  | BT3193 | putative coenzyme A transferase | 1.8 |
|  | BT3252 | hypothetical protein | 2.4 |
|  | BT3582 | hypothetical protein | 8.6 |
|  | BT3767 | lactaldehyde reductase | 1.7 |
|  | BT4058 | NADH dehydrogenase I, chain N | 1.8 |
|  |  |  |  |
|  | BT0217 | hypothetical protein | -1.8 |
|  | BT0787 | succinyl-CoA synthetase alpha chain | -1.7 |
|  | BT3223 | hypothetical protein | -91.9 |
| Carbohydrate transport and metabolism |  |  |  |
|  | BT0258 | glucosamine-6-phosphate isomerase | 1.4 |
|  | BT0455 | sialidase (Neuraminidase) | 1.8 |
|  | BT0792 | xylulose kinase (xylulokinase) | 3.3 |
|  | BT1098 | methylglyoxal synthase | 1.4 |
|  | BT1273 | L-fucose isomerase | 2.3 |
|  | BT1397 | putative membrane protein | 2.1 |
|  | BT1437 | hypothetical protein | 5.5 |
|  | BT1632 | chitinase | 1.9 |
|  | BT1647 | conserved hypothetical protein, putative integral membrane protein | 4.9 |
|  | BT1670 | putative transmembrane transport protein | 2.2 |
|  | BT2156 | putative sugar phosphate isomerase/epimerase | 2.2 |
|  | BT2199 | alpha-1,2-mannosidase | 1.9 |
|  | BT2851 | alpha-galactosidase (melibiase) | 2.0 |
|  | BT3009 | beta-glucosidase (gentiobiase) | 1.5 |
|  | BT3513 | beta-galactosidase | 1.8 |
|  | BT3530 | putative alpha-1,2-mannosidase | 2.3 |
|  | BT3532 | aldose 1-epimerase precursor | 1.9 |
|  | BT3567 | periplasmic beta-glucosidase precursor | 1.4 |
|  | BT3662 | putative endo-1,4-beta-xylanase | 1.8 |
|  | BT3663 | putative beta-xylosidase | 2.4 |
|  | BT3665 | alpha-L-fucosidase precursor | 2.5 |
|  | BT3698 | alpha-amylase, susG | 2.8 |
|  | BT3929 | triosephosphate isomerase | 1.6 |
|  | BT3956 | hypothetical protein | 2.4 |
|  | BT4093 | hypothetical protein | 1.7 |
|  | BT4095 | beta-xylosidase (1,4-beta-D-xylan xylosidase) | 1.8 |
|  | BT4152 | beta-galactosidase I | 2.1 |
|  | BT4160 | beta-galactosidase precursor | 1.9 |
|  | BT4181 | beta-galactosidase | 3.1 |
|  | BT4185 | xylosidase/arabinosidase | 2.3 |
|  | BT4311 | glucose/galactose transporter | 1.6 |
|  | BT4379 | putative oxalate:formate antiporter | 1.7 |
|  | BT4394 | beta-hexosaminidase precursor | 2.7 |
|  | BT4714 | periplasmic beta-glucosidase precursor, xylosidase/arabinosidase | 2.4 |
|  |  |  |  |
|  | BT0959 | hypothetical protein | -1.7 |
|  | BT1048 | putative secreted endoglycosidase | -1.6 |
|  | BT1051 | beta-hexosaminidase precursor | -1.6 |
|  | BT1477 | hypothetical protein | -1.7 |
|  | BT2150 | methylglyoxal synthase | -1.5 |
|  | BT3673 | TonB | -1.6 |
|  | BT3941 | hypothetical protein | -1.4 |
|  | BT4094 | hypothetical protein | -1.5 |
|  | BT4156 | beta-galactosidase | -1.7 |
|  | BT4406 | hypothetical protein | -2.6 |
| Amino acid transport and metabolism |  |  |  |
|  | BT0182 | osmo-regulated proline transporter (Na+/proline symporter) | 15.6 |
|  | BT0201 | histidinol dehydrogenase | 15.7 |
|  | BT0327 | glutamine ABC transporter, periplasmic glutamine-binding protein (glnH) | 2.4 |
|  | BT0414 | sulfate adenylyltransferase subunit 2 | 1.8 |
|  | BT0457 | sialic acid-specific 9-O-acetylesterase | 2.2 |
|  | BT0526 | L-asparaginase I | 1.7 |
|  | BT0832 | putative phosphoserine phosphatase | 2.2 |
|  | BT0878 | ABC transporter, ATP-binding protein | 2.1 |
|  | BT1077 | putative aspartate aminotransferase | 1.7 |
|  | BT1291 | spermidine/putrescine transport ATP-binding protein | 3.0 |
|  | BT1409 | hypothetical protein | 3.4 |
|  | BT1685 | lactoylglutathione lyase and related protein | 1.6 |
|  | BT1749 | glycine betaine-binding protein precursor | 1.6 |
|  | BT1838 | putative alanyl dipeptidyl peptidase | 1.6 |
|  | BT2077 | acetolactate synthase large subunit | 4.6 |
|  | BT2078 | dihydroxy-acid dehydratase | 2.2 |
|  | BT2186 | 3-phosphoshikimate 1-carboxyvinyltransferase | 1.5 |
|  | BT2401 | threonine synthase | 2.0 |
|  | BT2403 | aspartokinase/homoserine dehydrogenase | 2.3 |
|  | BT2571 | putative glutaminase | 1.5 |
|  | BT2573 | glutamate/gamma-aminobutyrate anti-porter | 1.6 |
|  | BT3394 | putative arginine decarboxylase | 2.1 |
|  | BT3395 | putative acetylglutamate kinase | 1.8 |
|  | BT3623 | sodium/iodide co-transporter | 3.0 |
|  | BT3935 | aminotransferase | 1.7 |
|  | BT3936 | prephenate dehydratase | 2.1 |
|  | BT4257 | putative histidinol-phosphatase | 1.5 |
|  | BT4612 | hypothetical protein | 1.7 |
|  |  |  |  |
|  | BT4078 | hypothetical protein | -1.6 |
|  | BT4421 | hypothetical protein | -1.6 |
|  | BT4424 | sorbitol dehydrogenase | -2.1 |
|  | BT4512 | putative zinc-type alcohol dehydrogenase | -1.4 |
| Nucleotide transport and metabolism |  |  |  |
|  | BT0748 | ribose-phosphate pyrophosphokinase | 2.2 |
|  | BT1236 | 2',3'-cyclic-nucleotide 2'-phosphodiesterase precursor | 1.5 |
|  | BT1381 | formyltetrahydrofolate deformylase | 1.9 |
|  | BT1733 | putative phosphoribosylformylglycinamidine synthase | 2.4 |
|  | BT4386 | hypoxanthine-guanine phosphoribosyltransferase | 1.8 |
|  |  |  |  |
|  | BT1009 | dihydroorotate dehydrogenase | -1.8 |
|  | BT1588 | hypothetical protein | -1.5 |
|  | BT2093 | hypothetical protein | -1.3 |
|  | BT3360 | phosphoribosylglycinamide formyltransferase | -1.8 |
| Coenzyme transport and metabolism |  |  |  |
|  | BT0698 | 3-methyl-2-oxobutanoate hydroxymethyltransferase | 3.1 |
|  | BT1335 | folylpolyglutamate synthase | 1.5 |
|  | BT2011 | putative nicotinate-nucleotide adenylyltransferase | 2.3 |
|  | BT4700 | isochorismate synthase entC | 1.7 |
|  | BT4701 | 2-oxoglutarate decarboxylase and 2-succinyl-6-hydroxy-2,4-cyclohexadiene-1-carboxylate synthase | 1.6 |
|  | BT4702 | dihydroxynapthoic acid synthetase | 1.7 |
|  |  |  |  |
|  | BT0923 | putative periplasmic protein | -2.7 |
|  | BT1089 | lipoate-protein ligase B | -1.6 |
|  | BT2905 | SusC/D paralog | -1.4 |
| Lipid transport and metabolism |  |  |  |
|  | BT0684 | putative long-chain-fatty-acid--CoA ligase | 3.9 |
|  | BT1192 | putative xylanase | 2.2 |
|  | BT1651 | pyrophosphorylase | 5.6 |
|  | BT1688 | biotin carboxyl carrier protein (BCCP) | 1.7 |
|  | BT2090 | methylmalonyl-CoA mutase large subunit | 1.5 |
|  | BT2232 | CDP-diacylglycerol--serine O-phosphatidyltransferase | 1.5 |
|  | BT4704 | O-succinylbenzoic acid--CoA ligase | 4.6 |
|  |  |  |  |
|  | BT0316 | putative hemin receptor | -1.5 |
|  | BT1170 | hypothetical protein | -2.2 |
|  | BT2114 | hypothetical protein | -1.7 |
|  | BT2382 | putative cardiolipin synthetase | -2.7 |
| Inorganic ion transport and metabolism |  |  |  |
|  | BT0412 | putative Na+/sulfate symporter | 1.6 |
|  | BT0413 | putative adenylylsulfate kinase | 1.9 |
|  | BT0415 | sulfate adenylyltransferase subunit 1/adenylylsulfate kinase | 2.0 |
|  | BT0756 | iduronate 2-sulfatase precursor | 4.8 |
|  | BT0867 | SusC/D paralog | 5.2 |
|  | BT1628 | putative sulfatase yidJ | 1.6 |
|  | BT1636 | arylsulfatase precursor | 1.7 |
|  | BT1673 | thiamine biosynthesis protein, putative | 2.2 |
|  | BT2364 | SusC/D paralog | 3.2 |
|  | BT2541 | putative calcium-transporting ATPase | 2.2 |
|  | BT2626 | SusC/D paralog | 2.6 |
|  | BT3095 | putative sulfatase | 1.5 |
|  | BT3569 | SusC/D paralog | 1.6 |
|  | BT3670 | SusC/D paralog | 1.7 |
|  | BT3739 | Na+/dicarboxylate or sulfate symporter | 2.0 |
|  | BT4097 | Trk system K+ uptake protein trkH | 2.3 |
|  | BT4098 | Trk system K+ uptake protein trkA | 3.2 |
|  | BT4267 | SusC/D paralog | 6.7 |
|  | BT4486 | voltage-gated K+ channel protein | 1.4 |
|  |  |  |  |
|  | BT1046 | SusC/D paralog | -1.8 |
|  | BT1052 | putative anti-sigma factor | -1.6 |
|  | BT1876 | putative anti-sigma factor | -2.1 |
|  | BT2039 | cation efflux system protein, AcrB/AcrD/AcrF family protein | -1.4 |
|  | BT2171 | putative anti-sigma factor | -3.9 |
|  | BT2172 | SusC/D paralog | -2.7 |
|  | BT2461 | SusC/D paralog | -2.9 |
|  | BT2462 | putative anti-sigma factor | -2.2 |
|  | BT2511 | putative transcription regulator | -3.4 |
|  | BT2512 | cation-transporting ATPase, P-type, putative zinc-transporting ATPase | -1.5 |
|  | BT2664 | putative phosphate ABC transporter, phosphate-binding component | -2.4 |
|  | BT3505 | SusC/D paralog | -1.7 |
|  | BT3565 | putative TonB-dependent receptor | -2.1 |
|  | BT3749 | putative anti-sigma factor | -3.7 |
|  | BT3750 | SusC/D paralog | -2.1 |
|  | BT3977 | hypothetical protein | -1.7 |
|  | BT4403 | putative anti-sigma factor | -2.9 |
|  | BT4404 | SusC/D paralog | -2.1 |
|  | BT4650 | voltage-gated chloride channel protein | -3.4 |
| Secondary metabolites biosynthesis, transport and catabolism |  |  |  |
|  | BT0171 | hypothetical protein | 3.7 |
|  | BT2525 | cephalosporin-C deacetylase | 2.1 |
|  | BT3104 | hypothetical protein | 1.6 |
| General function prediction only |  |  |  |
|  | BT0148 | putative hemolysin | 1.6 |
|  | BT0388 | galactoside O-acetyltransferase | 2.3 |
|  | BT0421 | tetratricopeptide repeat (TPR) family protein | 1.7 |
|  | BT0740 | flavoredoxin | 1.7 |
|  | BT1146 | putative metallo-beta-lactamase | 1.6 |
|  | BT1177 | putative polysaccharide biosynthesis protein | 3.9 |
|  | BT1652 | lipopolysaccharide biosynthesis protein | 7.1 |
|  | BT2013 | putative phosphohydrolases | 5.1 |
|  | BT2092 | putative membrane protein | 2.9 |
|  | BT2127 | putative beta-phosphoglucomutase | 1.6 |
|  | BT2159 | putative oxidoreductase | 1.9 |
|  | BT2211 | hypothetical protein | 1.9 |
|  | BT2212 | magnesium chelatase, subunit I, putative ATPase | 1.8 |
|  | BT2365 | SusC/D paralog | 3.1 |
|  | BT3462 | TPR domain protein | 1.5 |
|  | BT3551 | putative zinc protease | 1.4 |
|  | BT3840 | putative RNA-binding protein rbpA | 1.7 |
|  | BT3885 | putative phosphohydrolase, Icc family | 1.6 |
|  | BT4131 | hypothetical protein | 1.5 |
|  | BT4176 | conserved hypothetical protein, putative cytoplasmic protein | 2.2 |
|  | BT4190 | putative S-adenosylmethionine-dependent methytransferase | 2.8 |
|  | BT4230 | hypothetical protein | 2.3 |
|  | BT4271 | hypothetical protein | 3.2 |
|  | BT4292 | hypothetical protein | 1.4 |
|  | BT4324 | hypothetical protein | 1.6 |
|  | BT4463 | phosphatidate cytidylyltransferase | 1.6 |
|  |  |  |  |
|  | BT0163 | hypothetical protein | -1.6 |
|  | BT0164 | hypothetical protein | -1.5 |
|  | BT0168 | hypothetical protein | -1.5 |
|  | BT0221 | hypothetical protein | -1.6 |
|  | BT0434 | hypothetical protein | -1.9 |
|  | BT0660 | hypothetical protein | -1.6 |
|  | BT1239 | putative metal-dependent membrane protease | -1.4 |
|  | BT1263 | putative protease I | -1.5 |
|  | BT1839 | hypothetical protein | -1.5 |
|  | BT1894 | TPR-repeat-containing proteins | -1.4 |
|  | BT2174 | ABC transporter ATP-binding protein | -1.5 |
|  | BT2209 | putative nucleotidyltransferase | -1.6 |
|  | BT3224 | putative lysine decarboxylase | -6.5 |
|  | BT3508 | hypothetical protein | -1.6 |
|  | BT3583 | putative dehydrogenases and relate proteins | -1.7 |
|  | BT4140 | hypothetical protein | -1.6 |
|  | BT4302 | hypothetical protein | -1.6 |
|  | BT4465 | conserved hypothetical protein, putative outer membrane protein | -3.0 |
|  | BT4496 | putative oxidoreductase | -1.4 |
|  | BT4697 | transcriptional regulator | -6.8 |
| Function unknown |  |  |  |
|  | BT0210 | leucine-rich repeat protein, function unknown | 2.8 |
|  | BT0579 | putative transcription regulator | 6.2 |
|  | BT1315 | ribonuclease BN | 6.1 |
|  | BT2896 | hypothetical protein | 2.6 |
|  | BT3288 | putative integral membrane protein | 2.3 |
|  | BT3536 | hypothetical protein | 2.3 |
|  | BT3666 | hypothetical protein | 1.9 |
|  | BT3669 | hypothetical protein | 1.7 |
|  | BT3679 | conserved hypothetical protein, with a conserved domain | 2.9 |
|  | BT4008 | hypothetical protein | 1.4 |
|  | BT4220 | flotillin-like protein | 1.6 |
|  | BT4266 | hypothetical protein | 8.4 |
|  | BT4272 | hypothetical protein | 1.5 |
|  | BT4390 | hypothetical protein | 1.8 |
|  | BT4452 | hypothetical protein | 2.7 |
|  |  |  |  |
|  | BT1256 | hypothetical protein | -1.8 |
|  | BT1422 | hypothetical protein | -1.5 |
|  | BT1510 | hypothetical protein | -1.5 |
|  | BT1826 | hypothetical protein | -1.6 |
|  | BT2036 | hypothetical protein | -1.6 |
|  | BT2080 | conserved hypothetical protein, putative inner membrane protein | -1.5 |
|  | BT2606 | hypothetical protein | -1.8 |
|  | BT2650 | hypothetical protein | -1.5 |
|  | BT3390 | hypothetical protein | -2.9 |
|  | BT3410 | hypothetical protein | -4.1 |
|  | BT3650 | hypothetical protein | -2.0 |
|  | BT3697 | UDP-2,3-diacylglucosamine hydrolase | -1.5 |
|  | BT3863 | hypothetical protein | -1.6 |
|  | BT4212 | hypothetical protein | -1.5 |
|  | BT4349 | hypothetical protein | -2.8 |
|  | BT4607 | hypothetical protein | -1.5 |
|  | BT4732 | hypothetical protein | -2.1 |
| not in COGs |  |  |  |
|  | BT0127 | putative transmembrane protein | 1.5 |
|  | BT0141 | SusC/D paralog | 1.8 |
|  | BT0172 | hypothetical protein | 4.5 |
|  | BT0191 | SusC/D paralog | 3.6 |
|  | BT0207 | SusC/D paralog | 6.0 |
|  | BT0271 | hypothetical protein | 2.0 |
|  | BT0277 | conserved hypothetical protein | 1.6 |
|  | BT0292 | conserved hypothetical protein | 7.2 |
|  | BT0501 | hypothetical protein | 1.7 |
|  | BT0522 | hypothetical protein | 4.2 |
|  | BT0663 | hypothetical protein | 1.7 |
|  | BT0664 | hypothetical protein | 1.7 |
|  | BT0739 | hypothetical protein | 1.6 |
|  | BT0840 | hypothetical protein | 2.0 |
|  | BT0842 | hypothetical protein | 2.0 |
|  | BT0866 | SusC/D paralog | 4.8 |
|  | BT0939 | hypothetical protein | 1.7 |
|  | BT0950 | hypothetical protein | 1.6 |
|  | BT0980 | conserved hypothetical protein | 2.9 |
|  | BT1179 | glycosyltransferase, family 8 | 1.8 |
|  | BT1182 | hypothetical protein | 1.6 |
|  | BT1184 | conserved hypothetical protein | 2.4 |
|  | BT1300 | hypothetical protein | 1.7 |
|  | BT1305 | hypothetical protein | 2.2 |
|  | BT1372 | hypothetical protein | 1.7 |
|  | BT1506 | hypothetical protein | 2.7 |
|  | BT1573 | conserved hypothetical protein | 1.4 |
|  | BT1653 | hypothetical protein | 6.9 |
|  | BT1682 | SusC/D paralog | 2.7 |
|  | BT1687 | hypothetical protein | 2.1 |
|  | BT1771 | putative cell surface protein | 2.0 |
|  | BT1803 | conserved hypothetical protein | 1.9 |
|  | BT1934 | hypothetical protein | 1.7 |
|  | BT1936 | hypothetical protein | 1.6 |
|  | BT2006 | hypothetical protein | 2.2 |
|  | BT2110 | hypothetical protein | 2.6 |
|  | BT2131 | hypothetical protein | 1.6 |
|  | BT2155 | hypothetical protein | 1.7 |
|  | BT2157 | conserved hypothetical protein | 1.6 |
|  | BT2233 | hypothetical protein | 4.1 |
|  | BT2254 | putative pectate lyase | 2.2 |
|  | BT2524 | alpha-rhamnosidase | 1.9 |
|  | BT2625 | SusC/D paralog | 2.0 |
|  | BT2767 | hypothetical protein | 2.7 |
|  | BT2828 | hypothetical protein | 2.7 |
|  | BT2899 | hypothetical protein | 1.8 |
|  | BT2965 | hypothetical protein | 2.8 |
|  | BT3000 | conserved hypothetical protein | 1.7 |
|  | BT3056 | hypothetical protein | 2.1 |
|  | BT3059 | hypothetical protein | 3.7 |
|  | BT3079 | conserved hypothetical protein | 7.9 |
|  | BT3102 | SusC/D paralog | 1.8 |
|  | BT3243 | hypothetical protein | 1.5 |
|  | BT3262 | hypothetical protein | 1.5 |
|  | BT3313 | hypothetical protein | 1.7 |
|  | BT3380 | putative membrane protein | 1.8 |
|  | BT3423 | hypothetical protein | 3.2 |
|  | BT3446 | cell division protein FtsQ | 1.5 |
|  | BT3474 | SusC/D paralog | 2.1 |
|  | BT3539 | hypothetical protein | 2.5 |
|  | BT3568 | SusC/D paralog | 1.8 |
|  | BT3664 | putative alpha-glucosidase | 4.3 |
|  | BT3668 | hypothetical protein | 3.1 |
|  | BT3682 | hypothetical protein | 2.3 |
|  | BT3699 | outer membrane protein SusF | 2.4 |
|  | BT3700 | outer membrane protein SusE | 2.9 |
|  | BT3745 | hypothetical protein | 1.8 |
|  | BT3794 | hypothetical protein | 2.6 |
|  | BT3850 | hypothetical protein | 3.3 |
|  | BT3959 | conserved hypothetical protein, putative outer membrane protein | 1.9 |
|  | BT4056 | conserved hypothetical protein | 4.0 |
|  | BT4122 | SusC/D paralog | 4.0 |
|  | BT4132 | putative chitobiase | 2.0 |
|  | BT4145 | conserved hypothetical protein | 2.2 |
|  | BT4235 | hypothetical protein | 1.7 |
|  | BT4251 | hypothetical protein | 2.5 |
|  | BT4268 | SusC/D paralog | 4.0 |
|  | BT4269 | hypothetical protein | 7.5 |
|  | BT4284 | hypothetical protein | 2.5 |
|  | BT4285 | hypothetical protein | 2.4 |
|  | BT4286 | hypothetical protein | 2.0 |
|  | BT4333 | hypothetical protein | 3.1 |
|  | BT4497 | conserved hypothetical protein | 1.6 |
|  | BT4505 | hypothetical protein | 2.7 |
|  | BT4593 | conserved hypothetical protein | 2.0 |
|  | BT4626 | conserved hypothetical protein | 1.6 |
|  | BT4659 | SusC/D paralog | 2.2 |
|  |  |  |  |
|  | BT0001 | conserved hypothetical protein | -2.2 |
|  | BT0074 | hypothetical protein | -1.7 |
|  | BT0234 | putative transposase | -1.5 |
|  | BT0255 | hypothetical protein | -1.9 |
|  | BT0315 | hypothetical protein | -1.5 |
|  | BT0325 | hypothetical protein | -4.9 |
|  | BT0444 | hypothetical protein | -1.5 |
|  | BT0511 | hypothetical protein | -1.5 |
|  | BT0586 | hypothetical protein | -3.3 |
|  | BT0603 | conserved hypothetical protein | -2.3 |
|  | BT0646 | hypothetical protein | -1.6 |
|  | BT0708 | hypothetical protein | -1.8 |
|  | BT0731 | hypothetical protein | -1.7 |
|  | BT0819 | hypothetical protein | -2.5 |
|  | BT0922 | hypothetical protein | -2.6 |
|  | BT0932 | hypothetical protein | -1.6 |
|  | BT1047 | hypothetical protein | -1.5 |
|  | BT1049 | putative patatin-like protein | -1.9 |
|  | BT1050 | hypothetical protein | -1.8 |
|  | BT1073 | hypothetical protein | -11.1 |
|  | BT1074 | hypothetical protein | -28.6 |
|  | BT1142 | hypothetical protein | -8.4 |
|  | BT1143 | conserved hypothetical protein | -1.4 |
|  | BT1233 | hypothetical protein | -1.8 |
|  | BT1240 | conserved hypothetical protein | -1.6 |
|  | BT1255 | hypothetical protein | -2.0 |
|  | BT1280 | SusC/D paralog | -1.8 |
|  | BT1283 | conserved hypothetical protein | -2.0 |
|  | BT1284 | putative endo-beta-N-acetylglucosaminidase F1 precursor (mannosyl-glycoprotein endo-beta-N-acetyl-glucosaminidase F1) | -1.5 |
|  | BT1285 | endo-beta-N-acetylglucosaminidase | -3.2 |
|  | BT1366 | hypothetical protein | -1.5 |
|  | BT1534 | hypothetical protein | -3.9 |
|  | BT1558 | hypothetical protein | -1.4 |
|  | BT1784 | hypothetical protein | -1.7 |
|  | BT1788 | hypothetical protein | -3.1 |
|  | BT1792 | putative cell surface protein | -1.7 |
|  | BT1818 | hypothetical protein | -1.9 |
|  | BT1819 | hypothetical protein | -1.7 |
|  | BT1903 | hypothetical protein | -3.2 |
|  | BT1985 | hypothetical protein | -1.6 |
|  | BT2037 | hypothetical protein | -1.5 |
|  | BT2043 | hypothetical protein | -2.4 |
|  | BT2087 | hypothetical protein | -1.7 |
|  | BT2170 | hypothetical protein | -4.9 |
|  | BT2178 | hypothetical protein | -3.3 |
|  | BT2281 | hypothetical protein | -1.7 |
|  | BT2341 | conserved hypothetical protein | -1.7 |
|  | BT2369 | hypothetical protein | -1.5 |
|  | BT2448 | conserved hypothetical protein | -1.5 |
|  | BT2460 | SusC/D paralog | -1.7 |
|  | BT2491 | hypothetical protein | -2.8 |
|  | BT2500 | hypothetical protein | -6.3 |
|  | BT2502 | conserved hypothetical protein | -5.7 |
|  | BT2503 | hypothetical protein | -7.2 |
|  | BT2504 | hypothetical protein | -6.8 |
|  | BT2507 | hypothetical protein | -1.6 |
|  | BT2678 | hypothetical protein | -4.9 |
|  | BT2695 | hypothetical protein | -1.8 |
|  | BT2776 | hypothetical protein | -1.7 |
|  | BT2821 | SusC/D paralog | -2.3 |
|  | BT2822 | conserved hypothetical protein | -2.0 |
|  | BT2823 | hypothetical protein | -2.8 |
|  | BT2903 | hypothetical protein | -1.9 |
|  | BT2906 | conserved hypothetical protein | -1.8 |
|  | BT2926 | hypothetical protein | -1.6 |
|  | BT2997 | hypothetical protein | -1.6 |
|  | BT3064 | hypothetical protein | -3.6 |
|  | BT3088 | hypothetical protein | -1.7 |
|  | BT3125 | hypothetical protein | -2.1 |
|  | BT3183 | hypothetical protein | -1.5 |
|  | BT3188 | hypothetical protein | -5.9 |
|  | BT3189 | hypothetical protein | -5.5 |
|  | BT3191 | hypothetical protein | -5.2 |
|  | BT3221 | hypothetical protein | -52.8 |
|  | BT3222 | hypothetical protein | -99.5 |
|  | BT3225 | hypothetical protein | -4.3 |
|  | BT3315 | hypothetical protein | -3.2 |
|  | BT3384 | hypothetical protein | -1.6 |
|  | BT3397 | hypothetical protein | -2.6 |
|  | BT3400 | hypothetical protein | -1.6 |
|  | BT3436 | hypothetical protein | -2.3 |
|  | BT3438 | hypothetical protein | -1.9 |
|  | BT3495 | SusC/D paralog | -1.4 |
|  | BT3506 | hypothetical protein | -1.5 |
|  | BT3507 | hypothetical protein | -1.6 |
|  | BT3535 | hypothetical protein | -1.3 |
|  | BT3547 | hypothetical protein | -1.7 |
|  | BT3651 | hypothetical protein | -1.9 |
|  | BT3721 | hypothetical protein | -1.6 |
|  | BT3751 | outer membrane protein | -2.1 |
|  | BT3769 | hypothetical protein | -1.8 |
|  | BT3822 | hypothetical protein | -3.4 |
|  | BT3856 | hypothetical protein | -1.6 |
|  | BT3859 | hypothetical protein | -1.4 |
|  | BT3874 | hypothetical protein | -2.2 |
|  | BT3886 | hypothetical protein | -2.0 |
|  | BT3901 | hypothetical protein | -1.8 |
|  | BT3913 | hypothetical protein | -3.2 |
|  | BT3914 | hypothetical protein | -2.4 |
|  | BT3954 | hypothetical protein | -1.7 |
|  | BT3970 | hypothetical protein | -2.4 |
|  | BT4005 | hypothetical protein | -2.5 |
|  | BT4036 | hypothetical protein | -2.4 |
|  | BT4071 | hypothetical protein | -1.7 |
|  | BT4079 | hypothetical protein | -1.7 |
|  | BT4088 | SusC/D paralog | -2.3 |
|  | BT4196 | hypothetical protein | -1.6 |
|  | BT4249 | hypothetical protein | -1.9 |
|  | BT4341 | hypothetical protein | -1.9 |
|  | BT4348 | hypothetical protein | -2.0 |
|  | BT4361 | hypothetical protein | -2.0 |
|  | BT4384 | hypothetical protein | -3.6 |
|  | BT4405 | hypothetical protein | -2.0 |
|  | BT4407 | conserved hypothetical protein | -3.7 |
|  | BT4466 | hypothetical protein | -2.1 |
|  | BT4467 | hypothetical protein | -2.1 |
|  | BT4569 | hypothetical protein | -3.7 |
|  | BT4570 | hypothetical protein | -3.2 |
|  | BT4648 | hypothetical protein | -5.9 |
|  | BT4649 | hypothetical protein | -2.4 |
|  | BT4719 | hypothetical protein | -4.0 |
|  | BT4721 | hypothetical protein | -2.5 |
|  | BT4741 | hypothetical protein | -1.6 |
