# Supplementary material for: Genomic and Metabolic Studies of the Impact of Probiotics on a Model Gut Symbiont and Host
Source: PLoS Biol. 2006 Nov 28;4(12):e413. doi: 10.1371/journal.pbio.0040413 (PMC1661682; doi:10.1371/journal.pbio.0040413)
Supplement: Supplementary file 12 [file pbio.0040413.st003.doc]

Table S3. CAZy annotation of *B. thetaiotaomicron* and *B. longum* genomes.

| ***B. thetaiotaomicron*** | | | ***B. longum*** | | |
| --- | --- | --- | --- | --- | --- |
| **CAZy Family/ Known Activities** | **Gene** | **Description** |  | **Gene** | **Description** |
| **GH2** | **beta-galactosidase (EC 3.2.1.23) ; beta-mannosidase (EC 3.2.1.25); beta-glucuronidase (EC 3.2.1.31).** | | | | |
|  | BT0458 | beta-mannosidase precursor |  | BL0978 | beta-galactosidase |
|  | BT0461 | beta-galactosidase |  |  |  |
|  | BT0757 | beta-galactosidase |  |  |  |
|  | BT0983 | beta-galactosidase |  |  |  |
|  | BT0992 | beta-galactosidase |  |  |  |
|  | BT0993 | beta-galactosidase |  |  |  |
|  | BT0996 | beta-galactosidase |  |  |  |
|  | BT1626 | beta-galactosidase |  |  |  |
|  | BT1768 | beta-galactosidase |  |  |  |
|  | BT1776 | beta-galactosidase |  |  |  |
|  | BT2190 | beta-galactosidase |  |  |  |
|  | BT2191 | beta-galactosidase |  |  |  |
|  | BT2432 | beta-mannosidase precursor |  |  |  |
|  | BT2680 | beta-galactosidase |  |  |  |
|  | BT2855 | beta-mannosidase precursor |  |  |  |
|  | BT2922 | beta-galactosidase |  |  |  |
|  | BT2969 | beta-galactosidase |  |  |  |
|  | BT3092 | beta-galactosidase |  |  |  |
|  | BT3114 | beta-galactosidase |  |  |  |
|  | BT3179 | beta-galactosidase |  |  |  |
|  | BT3292 | beta-glucuronidase |  |  |  |
|  | BT3293 | beta-galactosidase |  |  |  |
|  | BT3340 | beta-galactosidase |  |  |  |
|  | BT3513 | beta-galactosidase |  |  |  |
|  | BT3599 | beta-mannosidase precursor |  |  |  |
|  | BT4050 | beta-galactosidase |  |  |  |
|  | BT4074 | beta-mannosidase precursor |  |  |  |
|  | BT4151 | beta-glucuronidase |  |  |  |
|  | BT4156 | beta-galactosidase |  |  |  |
|  | BT4181 | beta-galactosidase |  |  |  |
|  | BT4241 | beta-galactosidase |  |  |  |
|  | BT4667 | beta-galactosidase |  |  |  |
|  | BT4684 | beta-galactosidase |  |  |  |
| **GH3** | **beta-glucosidase (EC 3.2.1.21); xylan 1,4-beta-xylosidase (EC 3.2.1.37); beta-N-acetylhexosaminidase (EC 3.2.1.52); Glucan 1,3-beta-glucosidase (EC 3.2.1.58); Glucan 1,4-beta-glucosidase (EC 3.2.1.74); exo-1,3-1,4-glucanase (EC 3.2.1.-); alpha-L-arabinofuranosidase (EC 3.2.1.55).** | | | | |
|  | BT1778 | beta-glucosidase (gentiobiase) |  | BL1031 | possible B-hexosaminidase |
|  | BT1780 | periplasmic beta-glucosidase precursor |  | BL1757 | possible beta-glucosidase |
|  | BT1872 | periplasmic beta-glucosidase precursor |  | BL1763 | possible beta-glucosidase B |
|  | BT2439 | beta-N-acetylglucosaminidase |  |  |  |
|  | BT2854 | periplasmic beta-glucosidase precursor |  |  |  |
|  | BT3009 | beta-glucosidase (gentiobiase) |  |  |  |
|  | BT3300 | beta-glucosidase |  |  |  |
|  | BT3314 | thermostable beta-glucosidase B |  |  |  |
|  | BT3567 | periplasmic beta-glucosidase precursor |  |  |  |
|  | BT4714 | periplasmic beta-glucosidase precursor, xylosidase/arabinosidase |  |  |  |
| **GH5** | **Chitosanase (EC 3.2.1.132); beta-mannosidase (EC 3.2.1.25); Cellulase (EC 3.2.1.4); Glucan 1,3-beta-glucosidase (EC 3.2.1.58); Licheninase (EC 3.2.1.73); Glucan endo-1,6-beta-glucosidase (EC 3.2.1.75); Mannan endo-1,4-beta-mannosidase (EC 3.2.1.78); Endo-1,4-beta-xylanase (EC 3.2.1.8); Cellulose 1,4-beta-cellobiosidase (EC 3.2.1.91); endo-1,6-beta-galactanase (EC 3.2.1.-); beta-1,3-mannanase (EC 3.2.1.-)** | | | | |
|  | BT3043 | putative xylanase |  | BL0157 | narrowly conserved hypothetical protein |
|  |  |  |  | BL1333 | narrowly conserved hypothetical protein |
|  |  |  |  | BL1761 | probable beta-1,3-exoglucanase |
| **GH13** | **alpha-amylase (EC 3.2.1.1); pullulanase (EC 3.2.1.41); cyclomaltodextrin glucanotransferase (EC 2.4.1.19); cyclomaltodextrinase (EC 3.2.1.54); trehalose-6-phosphate hydrolase (EC 3.2.1.93); oligo-alpha-glucosidase (EC 3.2.1.10); maltogenic amylase (EC 3.2.1.133); neopullulanase (EC 3.2.1.135); alpha-glucosidase (EC 3.2.1.20); maltotetraose-forming alpha-amylase (EC 3.2.1.60); isoamylase (EC 3.2.1.68); glucodextranase (EC 3.2.1.70); maltohexaose-forming alpha-amylase (EC 3.2.1.98); branching enzyme (EC 2.4.1.18); trehalose synthase (EC 5.4.99.16); 4-alpha-glucanotransferase (EC 2.4.1.25); maltopentaose-forming alpha-amylase (EC 3.2.1.-) ; amylosucrase (EC 2.4.1.4) ; sucrose phosphorylase (EC 2.4.1.7); malto-oligosyltrehalose trehalohydrolase (EC 3.2.1.141); isomaltulose synthase (EC 5.4.99.11).** | | | | |
|  | BT0771 | 1,4-alpha-glucan branching enzyme (isoamylase or pullulanase type II) |  | BL0388 | possible glycanase or glycogenase with amylase domain |
|  | BT0773 | alpha-amylase 3 |  | BL0529 | probable alpha-1,4-glucosidase; maltase-like enzyme |
|  | BT1663 | pullulanase precursor |  | BL0536 | sucrose phosphorylase |
|  | BT3698 | SusG |  | BL0907 | possible cyclomaltodextrinase or neopullalanase |
|  | BT3704 | SusA |  | BL0982 | probable glycogen operon protein GlgX |
|  | BT4689 | pullulanase precursor |  | BL0999 | 1,4-alpha-glucan branching enzyme |
|  | BT4690 | alpha-amylase precursor |  | BL1526 | oligo-1,6-glucosidase |
|  |  |  |  | BL1573 | probable glycogen operon protein GlgX |
| **GH16** | **Xyloglucan:xyloglucosyl transferase (EC 2.4.1.207); Keratan-sulfate endo-1,4-beta-galactosidase (EC 3.2.1.103); Glucan endo-1,3-beta-D-glucosidase (EC 3.2.1.39); Endo-1,3(4)-beta-glucanase (EC 3.2.1.6); Licheninase (EC 3.2.1.73); Agarase (EC 3.2.1.81); kappa-carrageenase (EC 3.2.1.83)** | | | | |
|  | BT2550 | beta-glucanase precursor |  |  |  |
|  | BT2824 | beta-glucanase precursor |  |  |  |
|  | BT3683 | beta-glucanase precursor |  |  |  |
| **GH18** | **chitinase (EC 3.2.1.14); endo-beta-N-acetylglucosaminidase (EC 3.2.1.96); non-catalytic proteins: xylanase inhibitors; concanavalin B; narbonin** | | | | |
|  | BT1044 | hypothetical protein |  |  |  |
|  | BT1048 | putative secreted endoglycosidase |  |  |  |
|  | BT1285 | endo-beta-N-acetylglucosaminidase |  |  |  |
|  | BT1632 | chitinase |  |  |  |
|  | BT2825 | chitinase |  |  |  |
|  | BT3987 | endo-beta-N-acetylglucosaminidase F1 precursor |  |  |  |
|  | BT4709 | glycosyl hydrolase |  |  |  |
| **GH20** | **beta-hexosaminidase (EC 3.2.1.52); lacto-N-biosidase (EC 3.2.1.140).** | | | | |
|  | BT0456 | beta-hexosaminidase precursor |  | BL0056 | possible beta-hexosaminidase A |
|  | BT0459 | beta-hexosaminidase precursor |  |  |  |
|  | BT0460 | beta-hexosaminidase precursor |  |  |  |
|  | BT0506 | beta-hexosaminidase precursor |  |  |  |
|  | BT1051 | beta-hexosaminidase precursor |  |  |  |
|  | BT1621 | beta-hexosaminidase precursor |  |  |  |
|  | BT1627 | beta-hexosaminidase precursor |  |  |  |
|  | BT2459 | beta-hexosaminidase precursor |  |  |  |
|  | BT3178 | beta-hexosaminidase precursor |  |  |  |
|  | BT3598 | beta-hexosaminidase precursor |  |  |  |
|  | BT3868 | beta-N-hexosaminidase, glycosyl hyrolase family 20 |  |  |  |
|  | BT4337 | beta-hexosaminidase precursor |  |  |  |
|  | BT4394 | beta-hexosaminidase precursor |  |  |  |
|  | BT4681 | beta-hexosaminidase precursor |  |  |  |
| **GH23** | **lysozyme type G (EC 3.2.1.17).** | | | | |
|  | BT0183 | transglycosylase |  |  |  |
|  | BT3999 | membrane-bound lytic murein transglycosylase D presursor |  |  |  |
|  | BT4487 | putative membrane-bound lytic murein transglycosylase |  |  |  |
| **GH25** | **lysozyme (EC 3.2.1.17).** | | | | |
|  | BT0308 | putative glycosyl hydrolase |  |  |  |
| **GH27** | **alpha-galactosidase (EC 3.2.1.22); alpha-N-acetylgalactosaminidase (EC 3.2.1.49); isomalto-dextranase (EC 3.2.1.94).** | | | | |
|  | BT0065 | alpha-galactosidase |  | BL0177 | possible alpha-galactosidase |
|  | BT2662 | alpha-galactosidase precursor |  |  |  |
|  | BT3065 | alpha-galactosidase precursor |  |  |  |
|  | BT3592 | alpha-galactosidase precursor |  |  |  |
|  | BT4157 | alpha-galactosidase precursor |  |  |  |
| **GH28** | **polygalacturonase (EC 3.2.1.15); exo-polygalacturonase (EC 3.2.1.67); exo-polygalacturonase (EC 3.2.1.82); rhamnogalacturonase (EC not defined).** | | | | |
|  | BT0127 | putative transmembrane protein 120368:123661 forward MW:124232 |  |  |  |
|  | BT1018 | polygalacturonase (Pectinase) |  |  |  |
|  | BT2378 | endo-polygalacturonase |  |  |  |
|  | BT4123 | exo-poly-alpha-D-galacturonosidase precursor |  |  |  |
|  | BT4146 | exo-poly-alpha-D-galacturonosidase precursor |  |  |  |
|  | BT4149 | exo-poly-alpha-D-galacturonosidase precursor |  |  |  |
|  | BT4153 | Exopolygalacturonase precursor |  |  |  |
|  | BT4155 | conserved hypothetical protein |  |  |  |
|  | BT4187 | exo-poly-alpha-D-galacturonosidase precursor |  |  |  |
| **GH29** | **alpha-L-fucosidase (EC 3.2.1.51)** | | | | |
|  | BT1625 | conserved hypothetical protein |  |  |  |
|  | BT1842 | alpha-L-fucosidase precursor |  |  |  |
|  | BT2192 | conserved hypothetical protein |  |  |  |
|  | BT2970 | alpha-L-fucosidase precursor |  |  |  |
|  | BT3665 | alpha-L-fucosidase precursor |  |  |  |
|  | BT3798 | conserved hypothetical protein |  |  |  |
|  | BT3956 | conserved hypothetical protein |  |  |  |
|  | BT4136 | conserved hypothetical protein |  |  |  |
|  | BT4713 | conserved hypothetical protein |  |  |  |
| **GH30** | **glucosylceramidase (EC 3.2.1.45); beta-1,6-glucanase (EC 3.2.1.75).** | | | | |
|  | BT3026 | glycosylhydrolase, putative xylanase |  |  |  |
|  | BT3312 | glucosylceramidase precursor |  |  |  |
| **GH31** | **alpha-glucosidase (EC 3.2.1.20); glucoamylase (EC 3.2.1.3); sucrase-isomaltase (EC 3.2.1.48) (EC 3.2.1.10); alpha-xylosidase (EC 3.2.1.-); alpha-glucan lyase (EC 4.2.2.13); isomaltosyltransferase (EC 2.4.1.-).** | | | | |
|  | BT0339 | alpha-glucosidase |  | BL0523 | possible xylosidase or glucosidase |
|  | BT3085 | alpha-xylosidase |  | BL0542 | truncated alpha-glucosidase |
|  | BT3086 | alpha-glucosidase II |  | BL1334 | probable pentosidase or hexosidase |
|  | BT3169 | alpha-xylosidase |  |  |  |
|  | BT3299 | alpha-glucosidase II |  |  |  |
|  | BT3659 | alpha-xylosidase |  |  |  |
| **GH32** | **invertase (EC 3.2.1.26); inulinase (EC 3.2.1.7); levanase (EC 3.2.1.65); exo-inulinase (EC 3.2.1.80); sucrose:sucrose 1-fructosyl transferase (EC 2.4.1.99); fructan fructan 1-fructosyltransferase (EC 2.4.1.100).** | | | | |
|  | BT1759 | levanase precursor (2,6-beta-D-fructofuranosidase) |  | BL0105 | beta-fructofuranosidase (sucrase/invertase); possible inulinase |
|  | BT1760 | glycosylhydrolase |  |  |  |
|  | BT1765 | levanase precursor (2,6-beta-D-fructofuranosidase) |  |  |  |
|  | BT3082 | 2,6-beta-D-fructofuranosidase precursor |  |  |  |
| **GH33** | **sialidase or neuraminidase (EC 3.2.1.18); trans-sialidase (EC 2.4.1.-).** | | | | |
|  | BT0455 | sialidase (Neuraminidase) |  |  |  |
| **GH35** | **beta-galactosidase (EC 3.2.1.23).** | | | | |
|  | BT0290 | beta-galactosidase precursor |  |  |  |
|  | BT3654 | beta-galactosidase precursor |  |  |  |
|  | BT4160 | beta-galactosidase precursor |  |  |  |
| **GH36** | **alpha-galactosidase (EC 3.2.1.22); alpha-N-acetylgalactosaminidase (EC 3.2.1.49); stachyose synthase (EC 2.4.1.67); raffinose synthase (EC 2.4.1.82)** | | | | |
|  | BT2851 | alpha-galactosidase (melibiase) |  | BL1518 | alpha-galactosidase |
|  | BT3131 | alpha-galactosidase (melibiase) |  |  |  |
|  | BT3797 | conserved hypothetical protein |  |  |  |
| **GH38** | **alpha-mannosidase (EC 3.2.1.24) (EC 3.2.1.114).** | | | | |
|  | BT3774 | alpha-mannosidase |  | BL1327 | alpha-mannosidase |
|  | BT4072 | alpha-mannosidase |  | BL1328 | alpha-mannosidase |
|  |  |  |  | BL1329 | alpha-mannosidase |
| **GH42** | **beta-galactosidase (EC 3.2.1.23).** | | | | |
|  | BT4152 | beta-galactosidase I |  | BL0259 | beta-galactosidase I |
|  |  |  |  | BL1168 | beta-galactosidase I |
| **GH43** | **beta-xylosidase (EC 3.2.1.37); alpha-L-arabinofuranosidase (EC 3.2.1.55); arabinanase (EC 3.2.1.99); xylanase (EC 3.2.1.8).** | | | | |
|  | BT0145 | beta-xylosidase |  | BL0146 | possible arabinosidase |
|  | BT0264 | conserved hypothetical protein |  | BL0158 | very narrowly conserved hypothetical protein |
|  | BT0265 | conserved hypothetical protein |  | BL0182 | possible endo-1,5-alpha-L-arabinosidase |
|  | BT0360 | putative arabinan endo-1,5-alpha-L-arabinosidase |  | BL0183 | possible endo-1,5-alpha-L-arabinosidase |
|  | BT0367 | putative endo-arabinase |  | BL0184 | large protein with C-terminal homology to alpha-arabinofuranosidase II |
|  | BT0369 | endo-1,4-beta-xylanase D precursor |  | BL0187 | exo-alpha-L-arabinofuranosidase II |
|  | BT1021 | arabinosidase |  | BL0420 | probable extracellular protein possibly involved in xylan or arabinan degradation |
|  | BT1781 | xylosidase/arabinosidase |  | BL1543 | endo-1,4-beta-xylanase D |
|  | BT1873 | endo-arabinase |  | BL1544 | possible extracellular exo-xylanase |
|  | BT2112 | conserved hypothetical protein |  |  |  |
|  | BT2852 | putative beta-xylosidase |  |  |  |
|  | BT2895 | endo-1,4-beta-xylanase |  |  |  |
|  | BT2898 | endo-1,4-beta-xylanase D precursor |  |  |  |
|  | BT2900 | arabinan endo-1,5-alpha-L-arabinosidase A precursor |  |  |  |
|  | BT2912 | putative glycosyslhydrolase |  |  |  |
|  | BT2959 | conserved hypothetical protein |  |  |  |
|  | BT3094 | putative secreted xylosidase |  |  |  |
|  | BT3108 | putative beta-xylosidase |  |  |  |
|  | BT3467 | putatuve glycosylhydrolase |  |  |  |
|  | BT3515 | putatuve glycosylhydrolase |  |  |  |
|  | BT3516 | arabinan endo-1,5-alpha-L-arabinosidase A precursor |  |  |  |
|  | BT3655 | arabinosidase |  |  |  |
|  | BT3656 | putative beta-xylosidase |  |  |  |
|  | BT3662 | putative endo-1,4-beta-xylanase |  |  |  |
|  | BT3663 | putative beta-xylosidase |  |  |  |
|  | BT3675 | endo-1,4-beta-xylanase D precursor |  |  |  |
|  | BT3683 | beta-glucanase precursor |  |  |  |
|  | BT3685 | conserved hypothetical protein |  |  |  |
|  | BT4095 | beta-xylosidase (1,4-beta-D-xylan xylosidase) |  |  |  |
|  | BT4152 | beta-galactosidase I |  |  |  |
|  | BT4185 | xylosidase/arabinosidase |  |  |  |
| **GH51** | **alpha-L-arabinofuranosidase (EC 3.2.1.55); endoglucanase (EC 3.2.1.4).** | | | | |
|  | BT0348 | alpha-L-arabinofuranosidase |  | BL0181 | similar to alpha-arabinofuranosidase I |
|  | BT0368 | alpha-L-arabinofuranosidase A precursor |  | BL0544 | alpha-L-arabinosidase |
|  | BT3096 | alpha-L-arabinofuranosidase A precursor |  | BL1138 | similar to alpha-L-arabinofuranosidase A |
|  | BT3657 | alpha-L-arabinofuranosidase A precursor |  | BL1166 | alpha-L-arabinofuranosidase |
|  |  |  |  | BL1611 | alpha-L-arabinosidase |
| **GH53** | **endo-1,4-beta-galactanase (EC 3.2.1.89).** | | | | |
|  | BT4668 | arabinogalactan endo-1,4-beta-galactosidase |  | BL0257 | possible arabinogalactan endo-beta-galactosidase or galactanase |
| **GH57** | **alpha-amylase (EC 3.2.1.1); 4-alpha-glucanotransferase (EC 2.4.1.-); alpha-galactosidase (EC 3.2.1.22); amylopullulanase (EC 3.2.1.41)** | | | | |
|  | BT4305 | putative alpha-amylase |  |  |  |
| **GH66** | **cycloisomaltooligosaccharide glucanotransferase (EC 2.4.1.-); dextranase (EC 3.2.1.11).** | | | | |
|  | BT3087 | cycloisomaltooligosaccharide glucanotransferase |  |  |  |
| **GH67** | **alpha-glucuronidase (EC 3.2.1.139).** | | | | |
|  | BT2622 | alpha-glucuronidase |  |  |  |
| **GH73** | **endo-beta-N-acetylglucosaminidase (EC 3.2.1.96); beta-1,4-N-acetylmuramoylhydrolase (EC 3.2.1.17).** | | | | |
|  | BT1538 | hemagglutinin |  |  |  |
| **GH76** | **alpha-1,6-mannanase (EC 3.2.1.101).** | | | | |
|  | BT1883 | putative alpha-1,6-mannanase |  |  |  |
|  | BT2623 | putative alpha-1,6-mannanase |  |  |  |
|  | BT2631 | putative hydrolase |  |  |  |
|  | BT2949 | putative alpha-1,6-mannanase |  |  |  |
|  | BT3301 | putative alpha-1,6-mannanase |  |  |  |
|  | BT3501 | putative alpha-1,6-mannanase |  |  |  |
|  | BT3521 | alpha-1,6-mannanase |  |  |  |
|  | BT3524 | alpha-1,6-mannanase |  |  |  |
|  | BT3782 | putative hydrolase |  |  |  |
|  | BT3792 | putative alpha-1,6-mannanase |  |  |  |
| **GH77** | **amylomaltase or 4-alpha-glucanotransferase (EC 2.4.1.25).** | | | | |
|  | BT2146 | 4-alpha-glucanotransferase |  | BL0527 | 4-alpha-glucanotransferase |
|  |  |  |  | BL1570 | 4-alpha-glucanotransferase |
| **GH78** | **alpha-L-rhamnosidase (EC 3.2.1.40)** | | | | |
|  | BT1001 | putative alpha-rhamnosidase |  |  |  |
|  | BT1013 | putative alpha-rhamnosidase |  |  |  |
|  | BT1019 | putative secreted hydrolase |  |  |  |
|  | BT2523 | alpha-rhamnosidase |  |  |  |
|  | BT2524 | alpha-rhamnosidase |  |  |  |
|  | BT4076 | alpha-rhamnosidase |  |  |  |
| **GH84** | **N-acetyl beta-glucosaminidase (EC 3.2.1.52); hyaluronidase (EC 3.2.1.35).** | | | | |
|  | BT4395 | hyaluronoglucosaminidase precursor |  |  |  |
| **GH85** | **endo-beta-N-acetylglucosaminidase (EC 3.2.1.96)** | | | | |
|  |  |  |  | BL1335 | probable endo-beta-N-acetylglucosaminidase; di-N-acetylchitobiosyl beta-N-acetylglucosaminidase |
| **GH88** | **Deltalpha-4,5 unsaturated glucuronyl hydrolases (EC 3.2.1.-)** | | | | |
|  | BT0146 | unsaturated glucuronyl hydrolase |  |  |  |
|  | BT2913 | unsaturated glucuronylhydrolase |  |  |  |
|  | BT3348 | putative unsaturated glucuronyl hydrolase |  |  |  |
|  | BT4658 | glucuronyl hydrolase |  |  |  |
| **GH89** | **alpha-N-acetylglucosaminidase (EC 3.2.1.50)** | | | | |
|  | BT0438 | alpha-N-acetylglucosaminidase precursor |  |  |  |
|  | BT3590 | alpha-N-acetylglucosaminidase precursor |  |  |  |
|  | BT4359 | alpha-N-acetylglucosaminidase precursor |  |  |  |
| **GH92** | **alpha-1,2-mannosidase (EC 3.2.1.-)** | | | | |
|  | BT1032 | conserved hypothetical protein |  |  |  |
|  | BT1769 | conserved hypothetical protein |  |  |  |
|  | BT1878 | conserved hypothetical protein |  |  |  |
|  | BT2105 | putative alpha-1,2-mannosidase |  |  |  |
|  | BT2111 | conserved hypothetical protein |  |  |  |
|  | BT2199 | alpha-1,2-mannosidase |  |  |  |
|  | BT2629 | putative alpha-1,2-mannosidase |  |  |  |
|  | BT2948 | conserved hypothetical protein |  |  |  |
|  | BT3130 | conserved hypothetical protein |  |  |  |
|  | BT3527 | alpha-1,2-mannosidase |  |  |  |
|  | BT3530 | putative alpha-1,2-mannosidase |  |  |  |
|  | BT3773 | conserved hypothetical protein |  |  |  |
|  | BT3784 | putative alpha-1,2-mannosidase |  |  |  |
|  | BT3858 | conserved hypothetical protein 5023713:5025980 forward MW:85814 (COG3537, GH92: Putative alpha-1,2-mannosidase) |  |  |  |
|  | BT3962 | putative sugar hydrolase |  |  |  |
|  | BT3963 | putative sugar hydrolase |  |  |  |
|  | BT3965 | conserved hypothetical protein |  |  |  |
|  | BT3990 | putative alpha-1,2-mannosidase |  |  |  |
|  | BT3991 | putative alpha-1,2-mannosidase |  |  |  |
|  | BT3994 | putative alpha-1,2-mannosidase |  |  |  |
|  | BT4073 | conserved hypothetical protein |  |  |  |
|  | BT4092 | putative alpha-1,2-mannosidase |  |  |  |
|  | BT4093 | conserved hypothetical protein |  |  |  |
| **GH93** | **exo-arabinanase (EC 3.2.1.55)** | | | | |
|  | BT2892 | conserved hypothetical protein |  |  |  |
| **GH95** | **alpha-L-fucosidase (EC 3.2.1.51)** | | | | |
|  | BT1010 | conserved hypothetical protein |  |  |  |
|  | BT1777 | conserved hypothetical protein |  |  |  |
|  | BT3155 | conserved hypothetical protein |  |  |  |
|  | BT3173 | conserved hypothetical protein |  |  |  |
|  | BT4682 | conserved hypothetical protein |  |  |  |
| **GH97** | **alpha-glucosidase (EC 3.2.1.20)** | | | | |
|  | BT0132 | glucosidase |  |  |  |
|  | BT0683 | alpha-glucosidase |  |  |  |
|  | BT1871 | putative alpha-glucosidase |  |  |  |
|  | BT2620 | alpha-glucosidase |  |  |  |
|  | BT3163 | alpha-glucosidase, putative |  |  |  |
|  | BT3294 | putative alpha-glucosidase |  |  |  |
|  | BT3661 | alpha-glucosidase |  |  |  |
|  | BT3664 | putative alpha-glucosidase |  |  |  |
|  | BT3703 | glucosidase |  |  |  |
|  | BT4581 | alpha-glucosidase |  |  |  |
| **PL1** | **pectate lyase (EC 4.2.2.2); pectin lyase (EC 4.2.2.10).** | | | | |
|  | BT0980 | conserved hypothetical protein |  |  |  |
|  | BT1023 | conserved hypothetical protein |  |  |  |
|  | BT4115 | conserved hypothetical protein |  |  |  |
|  | BT4116 | conserved hypothetical protein |  |  |  |
|  | BT4119 | conserved hypothetical protein |  |  |  |
| **PL8** | **Hyaluronate lyase (EC 4.2.2.1); chondroitin ABC lyase (EC 4.2.2.4); chondroitin AC lyase (EC 4.2.2.5); xanthan lyase (EC 4.2.2.12).** | | | | |
|  | BT2964 | chondroitinase AC precursor |  |  |  |
|  | BT3324 | chondroitinase (chondroitin lyase) precursor |  |  |  |
|  | BT3350 | putative chondroitinase (chondroitin lyase) |  |  |  |
| **PL9** | **pectate lyase (EC 4.2.2.2); exopolygalacturonate lyase (EC 4.2.2.9).** | | | | |
|  | BT4170 | putative pectate lyase L precursor |  |  |  |
|  | BT4183 | pectate lyase L precursor |  |  |  |
| **PL10** | **pectate lyase (EC 4.2.2.2).** | | | | |
|  | BT2254 | putative pectate lyase |  |  |  |
| **PL11** | **rhamnogalacturonan lyase (EC 4.2.2.-).** | | | | |
|  | BT4175 | conserved hypothetical protein |  |  |  |
| **PL12** | **Heparin-sulfate lyase (EC 4.2.2.8)** | | | | |
|  | BT4657 | heparinase III protein |  |  |  |
|  | BT4662 | heparinase III protein, heparitin sulfate lyase |  |  |  |
| **PL13** | **heparin lyase (EC 4.2.2.7)** | | | | |
|  | BT4675 | heparin lyase I precursor |  |  |  |
