# Supplementary material for: Genomic and Metabolic Studies of the Impact of Probiotics on a Model Gut Symbiont and Host
Source: PLoS Biol. 2006 Nov 28;4(12):e413. doi: 10.1371/journal.pbio.0040413 (PMC1661682; doi:10.1371/journal.pbio.0040413)
Supplement: Supplementary file 13 [file pbio.0040413.st004.doc]

Table S4. COG functional categorization of *B. longum* genes with increased (red) or decreased (green) expression upon co-colonization with *B. thetaiotaomicron* in NMRI mice.

| **COG function** | **Gene** | **Description** | **Fold difference in expression (co-colonization versus mono-association)** |
| --- | --- | --- | --- |
| Translation |  |  |  |
|  | BL1075 | narrowly conserved hypothetical transmembrane protein | 1.5 |
|  | BL1409 | tRNA delta(2)-isopentenylpyrophosphate transferase | 1.5 |
|  |  |  |  |
|  | BL0405 | narrowly conserved hypothetical protein with possible methylase domain | -1.6 |
| Transcription |  |  |  |
|  | BL0528 | probable LacI-type transcriptional regulator | 1.7 |
|  | BL1691 | glucokinase | 1.6 |
|  |  |  |  |
|  | BL1312 | hypothetical protein with high similarity to putative regulatory protein NrdR | -1.4 |
|  | BL1769 | conserved hypothetical protein with acetyl transferase (GnaT) domain | -9.7 |
| Replication, recombination and repair |  |  |  |
|  | BL1751 | exonuclease VII, large subunit | 2.3 |
| Signal transduction mechanisms |  |  |  |
|  | BL0094 | hypothetical protein | 1.6 |
| Cell wall/membrane biogenesis |  |  |  |
|  | BL0249 | undecaprenyl-phosphate sugar phosphotransferase | 2.6 |
|  |  |  |  |
|  | BL1311 | hypothetical protein with LysM domain of membrane-bound lytic murein transglycosylases | -1.8 |
| Cell motility |  |  |  |
|  | BL1802 | hypothetical protein | 1.7 |
| Intracellular trafficking and secretion |  |  |  |
|  | BL0421 | narrowly conserved hypothetical protein possibly involved in xylan degradation | 4.2 |
| Energy production and conversion |  |  |  |
|  | BL1074 | dihydrolipoamide dehydrogenase | 5.1 |
|  | BL1111 | possible Na(+)/H(+) antiporter | 1.5 |
|  |  |  |  |
|  | BL0046 | narrowly conserved hypothetical protein | -1.4 |
|  | BL1771 | C4-dicarboxylate transporter | -7.3 |
| Carbohydrate transport and metabolism |  |  |  |
|  | BL0140 | hypothetical protein in PgaM phosphoglycerate mutase family | 2.9 |
|  | BL0529 | probable alpha-1,4-glucosidase; maltase-like enzyme | 4.6 |
|  | BL1544 | possible extracellular exo-xylanase | 1.8 |
|  | BL1695 | ATP binding protein of ABC transporter for pentoses | 1.9 |
|  |  |  |  |
|  | BL1327 | alpha-mannosidase | -2.8 |
|  | BL1329 | alpha-mannosidase | -6.7 |
|  | BL1330 | probable solute-binding protein of ABC transporter system | -9.1 |
|  | BL1331 | probable sugar permease of ABC transporter system | -7.4 |
|  | BL1332 | probable sugar permease of ABC transporter system | -10.1 |
|  | BL1333 | narrowly conserved hypothetical protein | -8.8 |
|  | BL1335 | probable endo-beta-N-acetylglucosaminidase; di-N-acetylchitobiosyl beta-N-acetylglucosaminidase | -14.3 |
|  | BL1337 | narrowly conserved hypothetical protein with possible isomerase function | -4.3 |
| Amino acid transport and metabolism |  |  |  |
|  | BL0024 | permease protein of ABC transporter for glutamate | 2.1 |
|  | BL0067 | carbamoyl-phosphate synthase small chain | 1.8 |
|  | BL0077 | probable solute-binding protein for glutamate/aspartate ABC transporter system | 2.0 |
|  | BL0394 | probable solute binding protein of ABC transporter system | 1.9 |
|  | BL0852 | probable branched-chain amino acid aminotransferase | 1.7 |
|  | BL0878 | chorismate synthase | 1.8 |
|  | BL1280 | succinyl-diaminopimelate desuccinylase | 1.7 |
|  | BL1300 | phosphoribosylformimino-5-aminoimidazole carboxamide ribotide isomerase | 3.2 |
|  |  |  |  |
|  | BL0704 | probable shikimate 5-dehydrogenase | -3.6 |
| Nucleotide transport and metabolism |  |  |  |
|  | BL1055 | thiamine biosynthesis protein | 1.6 |
|  |  |  |  |
|  | BL1770 | widely conserved hypothetical protein in the inosine-uridine preferring nucleoside hydrolase family | -7.7 |
| Coenzyme transport and metabolism |  |  |  |
|  | BL1055a | hypothetical protein |  |
|  |  |  |  |
|  | BL1145 | conserved hypothetical protein in sno glutamine amidotransferase family | -5.5 |
| Lipid transport and metabolism |  |  |  |
|  | BL0098 | 1-hydroxy-2-methyl-2-(E)-butenyl 4-diphosphate synthase 1 | 1.7 |
|  |  |  |  |
|  | BL0656 |  | -1.8 |
| Inorganic ion transport and metabolism |  |  |  |
|  | BL0252 | H(+)-stimulated manganese uptake system protein | 1.6 |
|  | BL1516 | probable NhaP-type Na(+)/H(+) exchanger | 2.4 |
|  |  |  |  |
|  | BL0409 | copper-transporting ATPase | -2.6 |
|  | BL1309 | probable cation efflux protein | -1.7 |
| General function prediction only |  |  |  |
|  | BL0744 | probable hydrolase or phosphatase | 2.3 |
|  | BL1670 | widely conserved hypothetical protein with methyltransferase domain | 1.5 |
|  |  |  |  |
|  | BL0151 | narrowly conserved hypothetical protein | -1.6 |
| Function unknown |  |  |  |
|  | BL0027 | narrowly conserved hypothetical protein | 2.6 |
|  | BL0476 | narrowly conserved hypothetical membrane protein | 1.8 |
|  |  |  |  |
|  | BL1338 | narrowly conserved hypothetical protein | -5.0 |
|  | BL1407 | narrowly conserved hypothetical protein | -1.4 |
| not in COGs |  |  |  |
|  | BL0605 | hypothetical protein | 1.6 |
|  | BL1488 | hypothetical protein | 2.9 |
|  | BL1543 | endo-1,4-beta-xylanase D | 2.3 |
|  |  |  |  |
|  | BL0655 | hypothetical protein | -1.5 |
|  | BL1235 | hypothetical protein | -1.4 |
|  | BL1404 | hypothetical protein | -2.5 |
