# Supplementary material for: Genomic and Metabolic Studies of the Impact of Probiotics on a Model Gut Symbiont and Host
Source: PLoS Biol. 2006 Nov 28;4(12):e413. doi: 10.1371/journal.pbio.0040413 (PMC1661682; doi:10.1371/journal.pbio.0040413)
Supplement: Supplementary file 14 [file pbio.0040413.st005.doc]

**Table S5.** All *B. thetaiotaomicron* genes identified by ANOVA displaying significant host effect, condition effect, interaction effect, or some combination of the three effects. Refer to Figure S4 for a Venn diagram illustrating the relationship between set number and effect(s).

| **Set** | **Gene ID** | **Host Effect (NMRI/B6)** | | **Condition Effect (Bt/BtBl)** | | **Interaction Effect** | |
| --- | --- | --- | --- | --- | --- | --- | --- |
|  |  | **p-value** | **q-value** | **p-value** | **q-value** | **p-value** | **q-value** |
| set1 | BT0007 | 0.000192632 | 0.0000978 | 0.124268 | 0.05010333 | 0.0968163 | 0.06075642 |
| set1 | BT0010 | 0.00000371 | 0.00000551 | 0.0195903 | 0.01055962 | 0.0269321 | 0.0243287 |
| set1 | BT0012 | 0.000106536 | 0.0000611 | 0.16747 | 0.06440479 | 0.0225473 | 0.02172491 |
| set1 | BT0015 | 0.00121391 | 0.000457096 | 0.705717 | 0.2072626 | 0.0145374 | 0.01640481 |
| set1 | BT0017 | 0.0071689 | 0.002172954 | 0.0387662 | 0.01890498 | 0.694391 | 0.2566238 |
| set1 | BT0021 | 0.0328183 | 0.008451485 | 0.0468269 | 0.02218218 | 0.315551 | 0.1454784 |
| set1 | BT0027 | 0.000102662 | 0.0000593 | 0.220194 | 0.0807969 | 0.918402 | 0.3108595 |
| set1 | BT0041 | 0.0197214 | 0.005361484 | 0.0185976 | 0.01010116 | 0.162453 | 0.088551 |
| set1 | BT0047 | 0.000729279 | 0.000293441 | 0.471157 | 0.1507679 | 0.0218054 | 0.02131325 |
| set1 | BT0048 | 0.013836 | 0.003918239 | 0.0608297 | 0.02749972 | 0.690631 | 0.2556856 |
| set1 | BT0049 | 0.0174558 | 0.004814733 | 0.0704877 | 0.03122319 | 0.673544 | 0.2510686 |
| set1 | BT0069 | 0.00156807 | 0.000568669 | 0.12407 | 0.05005691 | 0.0229129 | 0.02192587 |
| set1 | BT0071 | 0.0123449 | 0.00353247 | 0.0301367 | 0.0152828 | 0.031547 | 0.02740145 |
| set1 | BT0076 | 8.01389E-05 | 0.0000495 | 0.159815 | 0.06185472 | 0.401843 | 0.1735616 |
| set1 | BT0080 | 0.0136278 | 0.003865952 | 0.466781 | 0.149681 | 0.529632 | 0.2114378 |
| set1 | BT0089 | 0.00744545 | 0.002244196 | 0.916975 | 0.2548103 | 0.739583 | 0.2683263 |
| set1 | BT0098 | 0.00000073 | 0.00000215 | 0.385135 | 0.1280198 | 0.00826681 | 0.01124582 |
| set1 | BT0100 | 5.64556E-05 | 0.0000371 | 0.186082 | 0.07030898 | 0.0833638 | 0.054305 |
| set1 | BT0104 | 0.00735362 | 0.002220939 | 0.0294451 | 0.0149824 | 0.486813 | 0.199841 |
| set1 | BT0106 | 6.61644E-05 | 0.0000422 | 0.0332166 | 0.01661449 | 0.980662 | 0.3245114 |
| set1 | BT0111 | 2.60179E-05 | 0.0000209 | 0.261549 | 0.09305985 | 0.0259089 | 0.02371083 |
| set1 | BT0114 | 2.85038E-05 | 0.0000225 | 0.338036 | 0.11525 | 0.0138075 | 0.01582224 |
| set1 | BT0115 | 0.00614024 | 0.001892999 | 0.204859 | 0.07595442 | 0.200405 | 0.1037121 |
| set1 | BT0116 | 0.0274262 | 0.007215178 | 0.0785749 | 0.03415345 | 0.603751 | 0.2320046 |
| set1 | BT0117 | 0.00897329 | 0.002646742 | 0.0566086 | 0.02595081 | 0.0125621 | 0.01489511 |
| set1 | BT0122 | 0.00997106 | 0.002911541 | 0.675811 | 0.2004287 | 0.63574 | 0.2404573 |
| set1 | BT0124 | 0.000699416 | 0.000283147 | 0.0498033 | 0.0233178 | 0.0192194 | 0.01956486 |
| set1 | BT0129 | 0.000872827 | 0.000345507 | 0.969316 | 0.2650262 | 0.025591 | 0.02350783 |
| set1 | BT0130 | 0.000298958 | 0.000139673 | 0.0682373 | 0.0303371 | 0.0635602 | 0.04498613 |
| set1 | BT0132 | 0.0288601 | 0.007545114 | 0.225331 | 0.08230682 | 0.861435 | 0.2976973 |
| set1 | BT0134 | 0.000043099 | 0.0000305 | 0.107929 | 0.04457192 | 0.158976 | 0.08710859 |
| set1 | BT0136 | 1.99836E-05 | 0.0000172 | 0.383335 | 0.1275619 | 0.0605813 | 0.04347655 |
| set1 | BT0137 | 0.000276881 | 0.000131847 | 0.80356 | 0.2296878 | 0.0631491 | 0.04478153 |
| set1 | BT0147 | 0.000401708 | 0.000177488 | 0.121788 | 0.04926787 | 0.645344 | 0.2430253 |
| set1 | BT0149 | 0.00000597 | 0.00000742 | 0.64891 | 0.1937827 | 0.0227748 | 0.0218507 |
| set1 | BT0152 | 1.00809E-05 | 0.0000106 | 0.573893 | 0.1763058 | 0.11873 | 0.07005066 |
| set1 | BT0158 | 0.015283 | 0.004274619 | 0.0534945 | 0.02469707 | 0.0171282 | 0.01814056 |
| set1 | BT0160 | 0.000362551 | 0.000163431 | 0.0192738 | 0.01042624 | 0.302161 | 0.1410327 |
| set1 | BT0163 | 1.92261E-05 | 0.0000168 | 0.808542 | 0.2308933 | 0.0179313 | 0.01862824 |
| set1 | BT0173 | 0.00000167 | 0.00000343 | 0.0682482 | 0.0303371 | 0.283122 | 0.1344549 |
| set1 | BT0174 | 0.00000213 | 0.0000039 | 0.0232969 | 0.01224608 | 0.0992833 | 0.06183278 |
| set1 | BT0185 | 0.00989061 | 0.002892513 | 0.456792 | 0.1471026 | 0.070419 | 0.0483771 |
| set1 | BT0200 | 0.0366824 | 0.009357433 | 0.549204 | 0.1708062 | 0.170774 | 0.09192608 |
| set1 | BT0204 | 0.016647 | 0.004611814 | 0.171737 | 0.0657944 | 0.279725 | 0.1335763 |
| set1 | BT0205 | 0.00196586 | 0.000689154 | 0.45915 | 0.1476259 | 0.846864 | 0.2946499 |
| set1 | BT0211 | 0.0133425 | 0.003793549 | 0.149743 | 0.05870889 | 0.0466088 | 0.03574605 |
| set1 | BT0216 | 0.000367758 | 0.000165341 | 0.473061 | 0.1510931 | 0.27874 | 0.1333663 |
| set1 | BT0220 | 0.00122516 | 0.000460217 | 0.592811 | 0.1808755 | 0.0554189 | 0.0406549 |
| set1 | BT0237 | 0.00000377 | 0.00000551 | 0.0944869 | 0.0396717 | 0.0309008 | 0.02696782 |
| set1 | BT0238 | 0.00769955 | 0.002315254 | 0.55808 | 0.17265 | 0.926794 | 0.3129896 |
| set1 | BT0241 | 0.00952905 | 0.002797585 | 0.472556 | 0.1509717 | 0.12692 | 0.07358147 |
| set1 | BT0242 | 6.46652E-05 | 0.0000415 | 0.190878 | 0.0718738 | 0.0851621 | 0.05511952 |
| set1 | BT0244 | 1.55662E-05 | 0.0000145 | 0.98859 | 0.2688961 | 0.030692 | 0.02681748 |
| set1 | BT0246 | 0.0108 | 0.003121079 | 0.0814206 | 0.0351634 | 0.0220619 | 0.02144708 |
| set1 | BT0262 | 0.0101769 | 0.002960225 | 0.107257 | 0.04435506 | 0.878757 | 0.3016002 |
| set1 | BT0269 | 0.000000262 | 0.00000124 | 0.452803 | 0.1460127 | 0.0785658 | 0.05221342 |
| set1 | BT0274 | 0.000000252 | 0.0000012 | 0.308228 | 0.1067443 | 0.0864211 | 0.05576228 |
| set1 | BT0283 | 0.0315242 | 0.008162662 | 0.0797605 | 0.03457005 | 0.00934448 | 0.01225821 |
| set1 | BT0291 | 0.013486 | 0.003828596 | 0.493266 | 0.1562247 | 0.104013 | 0.0639107 |
| set1 | BT0295 | 0.0000018 | 0.00000357 | 0.110517 | 0.04545422 | 0.047466 | 0.0362331 |
| set1 | BT0296 | 3.23406E-05 | 0.0000247 | 0.0181488 | 0.009917321 | 0.75693 | 0.2727325 |
| set1 | BT0298 | 0.00488808 | 0.001548598 | 0.480181 | 0.1531243 | 0.161993 | 0.08849744 |
| set1 | BT0303 | 0.00157027 | 0.000569194 | 0.0861598 | 0.03688049 | 0.06291 | 0.04465512 |
| set1 | BT0304 | 0.0118373 | 0.003394922 | 0.463825 | 0.1489231 | 0.111894 | 0.06723346 |
| set1 | BT0305 | 0.00457839 | 0.00146273 | 0.901602 | 0.2518128 | 0.0435655 | 0.03417992 |
| set1 | BT0314 | 0.00190564 | 0.000670528 | 0.581777 | 0.1783196 | 0.523364 | 0.209906 |
| set1 | BT0329 | 1.49648E-05 | 0.000014 | 0.584763 | 0.1789425 | 0.235019 | 0.117463 |
| set1 | BT0330 | 4.03419E-05 | 0.0000292 | 0.443663 | 0.1436791 | 0.112421 | 0.0674397 |
| set1 | BT0333 | 4.10851E-05 | 0.0000295 | 0.676796 | 0.2005731 | 0.0245377 | 0.02287726 |
| set1 | BT0340 | 0.0128842 | 0.003675672 | 0.0506409 | 0.02362754 | 0.667303 | 0.249312 |
| set1 | BT0347 | 0.000130445 | 0.0000712 | 0.276621 | 0.0975889 | 0.802363 | 0.2842877 |
| set1 | BT0350 | 0.000558771 | 0.000234171 | 0.378614 | 0.1262692 | 0.86584 | 0.2987736 |
| set1 | BT0351 | 0.000298228 | 0.000139424 | 0.249798 | 0.08983137 | 0.970893 | 0.3225147 |
| set1 | BT0352 | 0.000689145 | 0.000279672 | 0.462353 | 0.1485371 | 0.765462 | 0.2749294 |
| set1 | BT0353 | 0.0174482 | 0.004814391 | 0.320577 | 0.1102163 | 0.918865 | 0.3108595 |
| set1 | BT0355 | 0.0117371 | 0.003371295 | 0.193623 | 0.07265827 | 0.650442 | 0.2445766 |
| set1 | BT0368 | 0.00990109 | 0.002893342 | 0.981095 | 0.2673994 | 0.797504 | 0.2829759 |
| set1 | BT0369 | 0.00235614 | 0.000809474 | 0.889484 | 0.2490618 | 0.309632 | 0.1434253 |
| set1 | BT0370 | 0.00000246 | 0.00000416 | 0.150654 | 0.05897036 | 0.28525 | 0.1351594 |
| set1 | BT0371 | 0.00000658 | 0.00000788 | 0.052845 | 0.02445752 | 0.266198 | 0.1289686 |
| set1 | BT0372 | 0.000000758 | 0.0000022 | 0.0826681 | 0.03558765 | 0.0151863 | 0.01681569 |
| set1 | BT0373 | 0.000078652 | 0.0000488 | 0.843416 | 0.2388196 | 0.0957904 | 0.06029698 |
| set1 | BT0378 | 6.81767E-05 | 0.0000433 | 0.61594 | 0.1860527 | 0.045208 | 0.03498244 |
| set1 | BT0385 | 4.92643E-05 | 0.0000337 | 0.371792 | 0.1243373 | 0.0545602 | 0.04014534 |
| set1 | BT0390 | 1.27919E-05 | 0.0000126 | 0.327397 | 0.1120338 | 0.0387794 | 0.03153741 |
| set1 | BT0391 | 0.000399038 | 0.000176515 | 0.52463 | 0.1643909 | 0.170698 | 0.09191888 |
| set1 | BT0395 | 0.000698435 | 0.000282987 | 0.931646 | 0.2576424 | 0.606299 | 0.2326387 |
| set1 | BT0396 | 1.61687E-05 | 0.0000148 | 0.65305 | 0.1948084 | 0.0124172 | 0.01479098 |
| set1 | BT0403 | 0.0278925 | 0.007326175 | 0.254072 | 0.09107013 | 0.665738 | 0.2488555 |
| set1 | BT0404 | 0.00418032 | 0.001344063 | 0.0307772 | 0.0155684 | 0.0887878 | 0.05679011 |
| set1 | BT0406 | 0.00138897 | 0.000512559 | 0.180048 | 0.06852207 | 0.976055 | 0.3233513 |
| set1 | BT0407 | 0.0248876 | 0.006630572 | 0.0556494 | 0.02553046 | 0.00718284 | 0.01039825 |
| set1 | BT0416 | 9.58523E-05 | 0.0000566 | 0.526428 | 0.164698 | 0.0707614 | 0.04854577 |
| set1 | BT0417 | 7.08813E-05 | 0.0000446 | 0.515755 | 0.1621146 | 0.0216894 | 0.02129651 |
| set1 | BT0418 | 0.000143376 | 0.0000762 | 0.119652 | 0.04860071 | 0.433738 | 0.1836495 |
| set1 | BT0420 | 0.00556765 | 0.001738425 | 0.196251 | 0.07343911 | 0.90642 | 0.3080921 |
| set1 | BT0421 | 3.83505E-05 | 0.0000281 | 0.263957 | 0.0938061 | 0.0119559 | 0.01442095 |
| set1 | BT0424 | 0.000358815 | 0.000162073 | 0.432636 | 0.1408253 | 0.0111075 | 0.01378209 |
| set1 | BT0428 | 0.00157392 | 0.000570244 | 0.610963 | 0.1849999 | 0.0198023 | 0.01994387 |
| set1 | BT0429 | 0.000109533 | 0.0000625 | 0.518214 | 0.1627605 | 0.0417205 | 0.03303253 |
| set1 | BT0430 | 0.000224448 | 0.000110799 | 0.862725 | 0.2431472 | 0.0076675 | 0.01082237 |
| set1 | BT0439 | 0.00797215 | 0.00238585 | 0.0445114 | 0.02126704 | 0.44533 | 0.1872553 |
| set1 | BT0440 | 0.0376911 | 0.009587983 | 0.411629 | 0.1352264 | 0.109561 | 0.06640243 |
| set1 | BT0446 | 0.000139463 | 0.0000747 | 0.404575 | 0.1331988 | 0.0226086 | 0.02176236 |
| set1 | BT0451 | 0.0173727 | 0.004797056 | 0.837021 | 0.2374055 | 0.0127336 | 0.01498949 |
| set1 | BT0456 | 0.00283852 | 0.000958665 | 0.691682 | 0.2042313 | 0.0752695 | 0.05072823 |
| set1 | BT0459 | 0.000324949 | 0.000149185 | 0.0825375 | 0.0355441 | 0.0779593 | 0.05194641 |
| set1 | BT0460 | 0.00000186 | 0.0000036 | 0.293674 | 0.1024001 | 0.161088 | 0.0880358 |
| set1 | BT0461 | 0.00000602 | 0.00000743 | 0.2665 | 0.09462632 | 0.330785 | 0.1506564 |
| set1 | BT0463 | 5.28177E-05 | 0.0000355 | 0.520261 | 0.1632336 | 0.146817 | 0.08178956 |
| set1 | BT0464 | 0.00168567 | 0.000604078 | 0.105042 | 0.04360327 | 0.0669137 | 0.04666218 |
| set1 | BT0466 | 0.00395599 | 0.001279772 | 0.053327 | 0.02464283 | 0.433567 | 0.1836495 |
| set1 | BT0467 | 0.00370482 | 0.001207076 | 0.943034 | 0.2597807 | 0.194102 | 0.101289 |
| set1 | BT0468 | 0.00000148 | 0.00000325 | 0.0507215 | 0.0236421 | 0.00796041 | 0.01102381 |
| set1 | BT0472 | 0.00179923 | 0.000638948 | 0.824575 | 0.2347503 | 0.380136 | 0.1667689 |
| set1 | BT0474 | 0.000175744 | 0.0000903 | 0.251889 | 0.09052942 | 0.107915 | 0.06571663 |
| set1 | BT0479 | 0.00013376 | 0.0000725 | 0.0409567 | 0.01983309 | 0.0203013 | 0.02035565 |
| set1 | BT0488 | 8.14056E-05 | 0.0000499 | 0.600559 | 0.1828239 | 0.09635 | 0.06056605 |
| set1 | BT0492 | 0.0263034 | 0.006963287 | 0.0218348 | 0.0116191 | 0.0305482 | 0.02670773 |
| set1 | BT0496 | 0.00000133 | 0.00000303 | 0.834475 | 0.2370102 | 0.0738968 | 0.05003884 |
| set1 | BT0498 | 0.000205232 | 0.000102917 | 0.0916512 | 0.03869629 | 0.142701 | 0.08031132 |
| set1 | BT0506 | 2.58355E-05 | 0.0000209 | 0.623963 | 0.1881469 | 0.212474 | 0.1087878 |
| set1 | BT0515 | 0.0100165 | 0.002921428 | 0.337053 | 0.1149797 | 0.174531 | 0.0935027 |
| set1 | BT0518 | 0.000000735 | 0.00000215 | 0.485576 | 0.1544777 | 0.00997387 | 0.01283158 |
| set1 | BT0526 | 1.65084E-05 | 0.0000151 | 0.077691 | 0.03388054 | 0.0316755 | 0.02744809 |
| set1 | BT0535 | 0.000472037 | 0.000202877 | 0.173731 | 0.066474 | 0.367966 | 0.1629364 |
| set1 | BT0536 | 0.000018344 | 0.0000163 | 0.643206 | 0.1923171 | 0.683805 | 0.2537978 |
| set1 | BT0545 | 0.000389629 | 0.000173061 | 0.829753 | 0.236002 | 0.82551 | 0.2894153 |
| set1 | BT0551 | 0.00000149 | 0.00000325 | 0.295614 | 0.1029215 | 0.019251 | 0.01956988 |
| set1 | BT0557 | 0.000979163 | 0.000381024 | 0.0869641 | 0.03718524 | 0.232696 | 0.1166595 |
| set1 | BT0558 | 9.99449E-05 | 0.0000582 | 0.0644055 | 0.02888921 | 0.0321143 | 0.02770531 |
| set1 | BT0567 | 0.000372286 | 0.000166947 | 0.301172 | 0.1045705 | 0.0367976 | 0.03033186 |
| set1 | BT0569 | 0.0013675 | 0.00050601 | 0.71377 | 0.2089672 | 0.149446 | 0.08293937 |
| set1 | BT0572 | 0.00248783 | 0.000850856 | 0.310258 | 0.1072628 | 0.0670964 | 0.04674518 |
| set1 | BT0574 | 0.00168455 | 0.000603966 | 0.912028 | 0.2540785 | 0.00885396 | 0.01177255 |
| set1 | BT0575 | 0.00106188 | 0.00040859 | 0.0503964 | 0.02354074 | 0.0216509 | 0.02128168 |
| set1 | BT0576 | 0.00000838 | 0.00000939 | 0.0873775 | 0.037296 | 0.0326517 | 0.02799653 |
| set1 | BT0578 | 5.05592E-05 | 0.0000343 | 0.998174 | 0.270589 | 0.163186 | 0.08885158 |
| set1 | BT0584 | 0.000944808 | 0.000369364 | 0.115964 | 0.04743608 | 0.061453 | 0.04393962 |
| set1 | BT0614 | 0.00150458 | 0.000548007 | 0.580546 | 0.1779875 | 0.402672 | 0.1737989 |
| set1 | BT0617 | 2.99642E-05 | 0.0000234 | 0.0378787 | 0.01851703 | 0.0862585 | 0.05568184 |
| set1 | BT0618 | 0.00935552 | 0.002751976 | 0.0225125 | 0.01188548 | 0.394705 | 0.1714186 |
| set1 | BT0619 | 0.00540524 | 0.001689108 | 0.0768425 | 0.03354605 | 0.225855 | 0.1143625 |
| set1 | BT0621 | 0.00154783 | 0.000562677 | 0.0422891 | 0.0203762 | 0.587098 | 0.2273911 |
| set1 | BT0626 | 0.0131496 | 0.003742922 | 0.0359067 | 0.01770347 | 0.655603 | 0.2460052 |
| set1 | BT0627 | 1.56175E-05 | 0.0000145 | 0.579339 | 0.1777076 | 0.0302533 | 0.02656064 |
| set1 | BT0630 | 0.00000088 | 0.00000241 | 0.0205677 | 0.01102741 | 0.519519 | 0.208706 |
| set1 | BT0631 | 0.000000363 | 0.00000142 | 0.0526527 | 0.02439657 | 0.0112434 | 0.01384597 |
| set1 | BT0633 | 0.00789862 | 0.002366652 | 0.0583782 | 0.02657045 | 0.826775 | 0.2896513 |
| set1 | BT0634 | 9.16044E-05 | 0.0000545 | 0.387266 | 0.1286573 | 0.0452 | 0.03498244 |
| set1 | BT0644 | 5.59363E-05 | 0.0000369 | 0.0984207 | 0.04110903 | 0.0237946 | 0.02241968 |
| set1 | BT0645 | 0.0140869 | 0.003979782 | 0.0308206 | 0.0155773 | 0.00717523 | 0.01039749 |
| set1 | BT0654 | 2.10335E-05 | 0.0000179 | 0.93294 | 0.2578057 | 0.336022 | 0.1521447 |
| set1 | BT0655 | 0.000000351 | 0.00000139 | 0.868898 | 0.2444307 | 0.515331 | 0.207605 |
| set1 | BT0656 | 1.05545E-05 | 0.000011 | 0.0466286 | 0.02211426 | 0.00726088 | 0.01045889 |
| set1 | BT0659 | 0.000186753 | 0.0000952 | 0.363996 | 0.1219665 | 0.118037 | 0.0697823 |
| set1 | BT0668 | 0.0210995 | 0.005711518 | 0.642297 | 0.1920928 | 0.690633 | 0.2556856 |
| set1 | BT0669 | 5.42518E-05 | 0.0000363 | 0.105707 | 0.04381908 | 0.0776189 | 0.05185394 |
| set1 | BT0672 | 0.00174378 | 0.000621961 | 0.858592 | 0.2423963 | 0.25782 | 0.1258593 |
| set1 | BT0687 | 0.000113303 | 0.0000638 | 0.0781395 | 0.03403845 | 0.0217978 | 0.02131325 |
| set1 | BT0688 | 0.000000843 | 0.00000233 | 0.443925 | 0.1437254 | 0.391249 | 0.1702703 |
| set1 | BT0701 | 1.45875E-05 | 0.0000138 | 0.355306 | 0.1198191 | 0.0833857 | 0.054305 |
| set1 | BT0702 | 0.00518177 | 0.001628013 | 0.0301048 | 0.01527304 | 0.194633 | 0.1014219 |
| set1 | BT0704 | 3.68006E-05 | 0.0000272 | 0.0671233 | 0.02991941 | 0.01249 | 0.01483643 |
| set1 | BT0713 | 0.00197982 | 0.000693085 | 0.403326 | 0.1329325 | 0.352462 | 0.1577876 |
| set1 | BT0714 | 0.0147107 | 0.004132875 | 0.35258 | 0.1191993 | 0.612815 | 0.2343222 |
| set1 | BT0715 | 0.0231551 | 0.006203614 | 0.185791 | 0.07024297 | 0.885986 | 0.303372 |
| set1 | BT0716 | 6.18809E-05 | 0.00004 | 0.0737731 | 0.03248787 | 0.395126 | 0.1714636 |
| set1 | BT0718 | 0.000271548 | 0.000129832 | 0.471927 | 0.1509305 | 0.456844 | 0.1908938 |
| set1 | BT0719 | 0.00440722 | 0.001412 | 0.733099 | 0.2136938 | 0.6671 | 0.2492996 |
| set1 | BT0724 | 0.00562707 | 0.001754806 | 0.0394345 | 0.01916127 | 0.0133272 | 0.01548852 |
| set1 | BT0725 | 0.00000671 | 0.00000797 | 0.46839 | 0.150053 | 0.0241098 | 0.02259087 |
| set1 | BT0726 | 0.00168084 | 0.000602921 | 0.0504619 | 0.02356222 | 0.0701804 | 0.04827514 |
| set1 | BT0727 | 0.00193008 | 0.000678497 | 0.109376 | 0.04504629 | 0.931051 | 0.313897 |
| set1 | BT0735 | 0.00000481 | 0.00000644 | 0.362987 | 0.1216959 | 0.0354189 | 0.02954016 |
| set1 | BT0736 | 7.22494E-05 | 0.0000453 | 0.594551 | 0.1812233 | 0.163473 | 0.08897484 |
| set1 | BT0741 | 0.00000386 | 0.00000558 | 0.577802 | 0.1773713 | 0.00705448 | 0.01027536 |
| set1 | BT0753 | 1.82583E-05 | 0.0000163 | 0.370986 | 0.1241365 | 0.0149358 | 0.01664734 |
| set1 | BT0757 | 0.000146225 | 0.0000773 | 0.0839512 | 0.03605008 | 0.514372 | 0.2075471 |
| set1 | BT0758 | 3.54602E-05 | 0.0000265 | 0.819049 | 0.2334522 | 0.069523 | 0.04795641 |
| set1 | BT0766 | 5.41489E-05 | 0.0000362 | 0.609403 | 0.1848179 | 0.042415 | 0.03345609 |
| set1 | BT0770 | 0.00000622 | 0.0000076 | 0.69585 | 0.2050127 | 0.0151513 | 0.01680202 |
| set1 | BT0771 | 0.00000597 | 0.00000742 | 0.594889 | 0.1812349 | 0.204608 | 0.1054223 |
| set1 | BT0773 | 1.56666E-05 | 0.0000145 | 0.519234 | 0.1629961 | 0.0129109 | 0.01515629 |
| set1 | BT0775 | 0.00068271 | 0.000277209 | 0.0441204 | 0.02112376 | 0.00713363 | 0.01034742 |
| set1 | BT0779 | 0.0252598 | 0.006712843 | 0.150999 | 0.05908626 | 0.183673 | 0.09715436 |
| set1 | BT0780 | 0.000183055 | 0.0000935 | 0.663358 | 0.1976088 | 0.0197838 | 0.01994387 |
| set1 | BT0785 | 0.0010696 | 0.000411162 | 0.0300857 | 0.01526976 | 0.423264 | 0.1803453 |
| set1 | BT0786 | 0.0174959 | 0.004822279 | 0.293171 | 0.1023219 | 0.782072 | 0.2790067 |
| set1 | BT0794 | 2.81219E-05 | 0.0000222 | 0.902984 | 0.2521405 | 0.0627511 | 0.04460704 |
| set1 | BT0795 | 0.00000226 | 0.000004 | 0.350855 | 0.1186826 | 0.88389 | 0.3027956 |
| set1 | BT0796 | 0.0238089 | 0.006360764 | 0.4025 | 0.1327327 | 0.0539971 | 0.03981084 |
| set1 | BT0799 | 0.0161295 | 0.004484865 | 0.870261 | 0.2447002 | 0.337401 | 0.1525338 |
| set1 | BT0800 | 0.0253004 | 0.006721272 | 0.195149 | 0.07311734 | 0.281792 | 0.1340814 |
| set1 | BT0802 | 0.00000172 | 0.0000035 | 0.120227 | 0.04876704 | 0.179341 | 0.09534882 |
| set1 | BT0804 | 0.0010935 | 0.00041884 | 0.0524144 | 0.02430827 | 0.878249 | 0.3015544 |
| set1 | BT0814 | 0.0000015 | 0.00000325 | 0.77238 | 0.2228318 | 0.0260687 | 0.02382736 |
| set1 | BT0815 | 0.00143675 | 0.000527618 | 0.436257 | 0.1417503 | 0.0331342 | 0.02824525 |
| set1 | BT0829 | 0.00000646 | 0.00000778 | 0.569241 | 0.1754119 | 0.0216082 | 0.02128168 |
| set1 | BT0839 | 0.00814631 | 0.002430284 | 0.958749 | 0.2630898 | 0.119155 | 0.07013228 |
| set1 | BT0840 | 0.00299925 | 0.001001773 | 0.0496924 | 0.02328392 | 0.0174414 | 0.01830062 |
| set1 | BT0842 | 0.0031705 | 0.001051991 | 0.128636 | 0.05157342 | 0.0121542 | 0.01458821 |
| set1 | BT0843 | 0.00111166 | 0.000424936 | 0.7195 | 0.2103898 | 0.0102772 | 0.01309557 |
| set1 | BT0854 | 0.00574996 | 0.001789443 | 0.0222345 | 0.01178507 | 0.686648 | 0.2545691 |
| set1 | BT0856 | 0.000869506 | 0.000344914 | 0.941979 | 0.2596677 | 0.0682512 | 0.04730276 |
| set1 | BT0858 | 0.00734447 | 0.002219061 | 0.678017 | 0.2007872 | 0.607555 | 0.2329785 |
| set1 | BT0864 | 0.0210418 | 0.005697937 | 0.252416 | 0.09069185 | 0.0419671 | 0.03319198 |
| set1 | BT0871 | 1.42637E-05 | 0.0000136 | 0.0274946 | 0.01413883 | 0.873929 | 0.3005757 |
| set1 | BT0872 | 6.65163E-05 | 0.0000424 | 0.181511 | 0.06888353 | 0.0183529 | 0.01894555 |
| set1 | BT0875 | 6.48E-08 | 0.000000654 | 0.913383 | 0.2542215 | 0.515317 | 0.207605 |
| set1 | BT0880 | 0.0233943 | 0.006256626 | 0.049572 | 0.0232413 | 0.114772 | 0.06840277 |
| set1 | BT0885 | 0.00211952 | 0.000737556 | 0.0922411 | 0.03889098 | 0.0173054 | 0.01826235 |
| set1 | BT0889 | 1.38716E-05 | 0.0000134 | 0.609404 | 0.1848179 | 0.22304 | 0.1131713 |
| set1 | BT0892 | 1.47902E-05 | 0.0000139 | 0.0759296 | 0.03320752 | 0.00991746 | 0.01279264 |
| set1 | BT0896 | 0.000531353 | 0.000224671 | 0.429975 | 0.1401858 | 0.135299 | 0.07703956 |
| set1 | BT0897 | 0.000144246 | 0.0000765 | 0.608763 | 0.1847627 | 0.216129 | 0.1103727 |
| set1 | BT0898 | 0.0221313 | 0.005955781 | 0.64038 | 0.1917093 | 0.0278808 | 0.02498581 |
| set1 | BT0900 | 0.000352198 | 0.000159811 | 0.474013 | 0.1513172 | 0.134741 | 0.07684106 |
| set1 | BT0906 | 0.00000971 | 0.0000103 | 0.200093 | 0.07462254 | 0.0217463 | 0.02131325 |
| set1 | BT0909 | 0.0000018 | 0.00000357 | 0.350497 | 0.1185947 | 0.0244856 | 0.02286863 |
| set1 | BT0912 | 0.00483576 | 0.001535236 | 0.0542284 | 0.02496388 | 0.00787014 | 0.01096084 |
| set1 | BT0916 | 0.0274067 | 0.007214985 | 0.726675 | 0.2120259 | 0.00846749 | 0.01139284 |
| set1 | BT0924 | 0.00000122 | 0.00000289 | 0.942836 | 0.2597807 | 0.0286608 | 0.02554447 |
| set1 | BT0926 | 0.00000772 | 0.00000885 | 0.518109 | 0.1627605 | 0.0316517 | 0.02744809 |
| set1 | BT0930 | 0.00535436 | 0.001675283 | 0.456044 | 0.1469009 | 0.336609 | 0.1522625 |
| set1 | BT0948 | 0.000645575 | 0.000264992 | 0.0554206 | 0.02544482 | 0.584195 | 0.2266854 |
| set1 | BT0949 | 0.00000942 | 0.0000101 | 0.0469655 | 0.0222304 | 0.830983 | 0.2907092 |
| set1 | BT0956 | 4.70052E-05 | 0.0000326 | 0.431277 | 0.1405344 | 0.0169756 | 0.01805711 |
| set1 | BT0970 | 0.0351136 | 0.00898146 | 0.343817 | 0.1169631 | 0.0742953 | 0.05023468 |
| set1 | BT0972 | 0.00220502 | 0.000763447 | 0.0521104 | 0.02420099 | 0.819459 | 0.2879834 |
| set1 | BT0973 | 0.00415094 | 0.001335752 | 0.650283 | 0.1941447 | 0.67093 | 0.2503485 |
| set1 | BT0974 | 0.00149151 | 0.000544032 | 0.0421999 | 0.02034466 | 0.890566 | 0.3043723 |
| set1 | BT0978 | 0.00969392 | 0.002840479 | 0.65366 | 0.1949121 | 0.400241 | 0.1731059 |
| set1 | BT0984 | 0.00000641 | 0.00000773 | 0.624173 | 0.1881632 | 0.0133369 | 0.01548852 |
| set1 | BT0988 | 0.0283412 | 0.007427466 | 0.0370205 | 0.0181604 | 0.0690938 | 0.04775146 |
| set1 | BT0989 | 0.000611516 | 0.000252363 | 0.194282 | 0.07288167 | 0.0112022 | 0.01381842 |
| set1 | BT0990 | 0.000244134 | 0.000118681 | 0.268263 | 0.09519635 | 0.0211089 | 0.02096492 |
| set1 | BT0991 | 0.0185044 | 0.0050707 | 0.540757 | 0.1687003 | 0.00798864 | 0.01105248 |
| set1 | BT0992 | 0.00000392 | 0.00000564 | 0.0447077 | 0.02132044 | 0.0376873 | 0.03091515 |
| set1 | BT1009 | 0.0344471 | 0.008825894 | 0.0181775 | 0.009926545 | 0.0329564 | 0.02815223 |
| set1 | BT1011 | 0.0293842 | 0.007666245 | 0.0509184 | 0.02370208 | 0.171129 | 0.09204966 |
| set1 | BT1017 | 0.0248702 | 0.006630258 | 0.444195 | 0.1437742 | 0.23319 | 0.116792 |
| set1 | BT1018 | 0.0160314 | 0.004464148 | 0.26008 | 0.09270635 | 0.770853 | 0.2762573 |
| set1 | BT1020 | 0.00694606 | 0.002115576 | 0.0280825 | 0.01439214 | 0.937217 | 0.315324 |
| set1 | BT1023 | 0.000158394 | 0.0000828 | 0.0260057 | 0.01345336 | 0.0313787 | 0.02730373 |
| set1 | BT1026 | 0.00515236 | 0.001621465 | 0.513228 | 0.1614884 | 0.0250016 | 0.0231694 |
| set1 | BT1029 | 0.0194023 | 0.005289935 | 0.120106 | 0.04873432 | 0.272002 | 0.1309297 |
| set1 | BT1032 | 2.76834E-05 | 0.000022 | 0.458954 | 0.1476021 | 0.0673352 | 0.04684485 |
| set1 | BT1033 | 0.010634 | 0.003080164 | 0.0389475 | 0.01897042 | 0.176017 | 0.09402428 |
| set1 | BT1037 | 8.18E-09 | 0.000000225 | 0.0355735 | 0.01759665 | 0.00712411 | 0.01034742 |
| set1 | BT1038 | 0.0000016 | 0.00000337 | 0.361085 | 0.1212264 | 0.0240594 | 0.02259087 |
| set1 | BT1039 | 0.00000017 | 0.000001 | 0.130375 | 0.05216513 | 0.0129744 | 0.0151877 |
| set1 | BT1040 | 1.15143E-05 | 0.0000117 | 0.22105 | 0.08106175 | 0.0753017 | 0.05072823 |
| set1 | BT1041 | 0.0000027 | 0.00000442 | 0.616548 | 0.1861898 | 0.0836519 | 0.05435778 |
| set1 | BT1042 | 0.000254362 | 0.000122622 | 0.074689 | 0.03280806 | 0.079879 | 0.05280629 |
| set1 | BT1052 | 0.00000116 | 0.00000278 | 0.0195571 | 0.01054643 | 0.634546 | 0.2402533 |
| set1 | BT1058 | 0.000140999 | 0.0000753 | 0.718818 | 0.2102413 | 0.017456 | 0.01830285 |
| set1 | BT1059 | 0.000823492 | 0.000327692 | 0.630614 | 0.1896789 | 0.257539 | 0.1257639 |
| set1 | BT1062 | 0.000494342 | 0.000211026 | 0.12075 | 0.04894631 | 0.0458631 | 0.03541469 |
| set1 | BT1063 | 1.02778E-05 | 0.0000108 | 0.0366233 | 0.01803633 | 0.0163575 | 0.01768151 |
| set1 | BT1079 | 0.000405434 | 0.000178854 | 0.87374 | 0.2453928 | 0.155386 | 0.08558877 |
| set1 | BT1082 | 2.28092E-05 | 0.000019 | 0.268724 | 0.09527597 | 0.12715 | 0.07359855 |
| set1 | BT1083 | 0.00853977 | 0.002530214 | 0.0369482 | 0.01814292 | 0.148156 | 0.08237918 |
| set1 | BT1084 | 0.00803482 | 0.002403655 | 0.216192 | 0.07966723 | 0.215751 | 0.110235 |
| set1 | BT1098 | 4.19882E-05 | 0.00003 | 0.549626 | 0.1708494 | 0.0693486 | 0.04788247 |
| set1 | BT1101 | 4.67534E-05 | 0.0000325 | 0.361301 | 0.1212653 | 0.252285 | 0.1236095 |
| set1 | BT1102 | 9.14739E-05 | 0.0000545 | 0.432841 | 0.1408541 | 0.22677 | 0.1146676 |
| set1 | BT1103 | 7.44917E-05 | 0.0000464 | 0.0897691 | 0.03808803 | 0.636964 | 0.2407962 |
| set1 | BT1104 | 0.00000462 | 0.00000625 | 0.775643 | 0.2235062 | 0.889754 | 0.3042364 |
| set1 | BT1105 | 0.00000546 | 0.00000695 | 0.075899 | 0.03320616 | 0.442571 | 0.1863087 |
| set1 | BT1106 | 1.60785E-05 | 0.0000147 | 0.18053 | 0.06866224 | 0.232068 | 0.1165307 |
| set1 | BT1107 | 0.00000148 | 0.00000325 | 0.104107 | 0.04325975 | 0.768142 | 0.2754875 |
| set1 | BT1108 | 0.00516874 | 0.001625269 | 0.361072 | 0.1212264 | 0.946245 | 0.3174157 |
| set1 | BT1109 | 0.0220598 | 0.005939601 | 0.873097 | 0.2453263 | 0.968467 | 0.3220219 |
| set1 | BT1115 | 4.28732E-05 | 0.0000304 | 0.813999 | 0.2321772 | 0.187074 | 0.09846163 |
| set1 | BT1116 | 0.000749097 | 0.000300298 | 0.471343 | 0.1507836 | 0.496823 | 0.202077 |
| set1 | BT1129 | 0.000892225 | 0.000351348 | 0.0260906 | 0.0134915 | 0.102938 | 0.06357257 |
| set1 | BT1132 | 0.000995568 | 0.000386612 | 0.587839 | 0.1795853 | 0.0525415 | 0.03911089 |
| set1 | BT1137 | 1.98696E-05 | 0.0000172 | 0.945682 | 0.2602134 | 0.047429 | 0.0362331 |
| set1 | BT1151 | 0.00000129 | 0.00000297 | 0.179414 | 0.06830953 | 0.231846 | 0.1164921 |
| set1 | BT1152 | 5.59E-08 | 0.000000641 | 0.055284 | 0.02539176 | 0.3216 | 0.1475259 |
| set1 | BT1153 | 0.00000661 | 0.0000079 | 0.0978474 | 0.04092618 | 0.333575 | 0.1515335 |
| set1 | BT1156 | 1.89555E-05 | 0.0000166 | 0.0297232 | 0.01511117 | 0.0295336 | 0.02611623 |
| set1 | BT1157 | 0.0000018 | 0.00000357 | 0.147914 | 0.0580672 | 0.016055 | 0.0174374 |
| set1 | BT1158 | 0.00000102 | 0.00000259 | 0.0943133 | 0.03962636 | 0.00894319 | 0.01187671 |
| set1 | BT1159 | 1.03975E-05 | 0.0000108 | 0.0247901 | 0.01291439 | 0.0237955 | 0.02241968 |
| set1 | BT1161 | 2.58915E-05 | 0.0000209 | 0.781655 | 0.2247026 | 0.0265229 | 0.02412535 |
| set1 | BT1172 | 0.00465665 | 0.001483975 | 0.171967 | 0.06586163 | 0.0285283 | 0.02544182 |
| set1 | BT1182 | 0.000117439 | 0.0000655 | 0.119229 | 0.04849249 | 0.00746253 | 0.01065602 |
| set1 | BT1185 | 0.00000626 | 0.0000076 | 0.323419 | 0.1108806 | 0.491552 | 0.2007675 |
| set1 | BT1192 | 0.000000163 | 0.000001 | 0.743843 | 0.2162801 | 0.437516 | 0.1847635 |
| set1 | BT1193 | 1.18516E-05 | 0.0000119 | 0.0895248 | 0.03804716 | 0.162104 | 0.08851465 |
| set1 | BT1194 | 0.000500374 | 0.00021336 | 0.908395 | 0.2533001 | 0.694302 | 0.2566238 |
| set1 | BT1196 | 0.0034198 | 0.001122914 | 0.831624 | 0.2363672 | 0.0173353 | 0.01826538 |
| set1 | BT1199 | 0.00000535 | 0.00000684 | 0.378847 | 0.126312 | 0.0463474 | 0.03560368 |
| set1 | BT1202 | 1.85562E-05 | 0.0000164 | 0.155716 | 0.0605788 | 0.216171 | 0.1103727 |
| set1 | BT1203 | 0.000714626 | 0.000288466 | 0.236468 | 0.0858298 | 0.28638 | 0.1355199 |
| set1 | BT1205 | 0.00000515 | 0.00000669 | 0.0468613 | 0.02218978 | 0.00862193 | 0.01154772 |
| set1 | BT1206 | 0.00232122 | 0.000799291 | 0.211565 | 0.07817681 | 0.0556552 | 0.04080786 |
| set1 | BT1209 | 3.33422E-05 | 0.0000253 | 0.323568 | 0.1108806 | 0.933099 | 0.3143709 |
| set1 | BT1210 | 2.70634E-05 | 0.0000216 | 0.163393 | 0.06303757 | 0.842738 | 0.2937419 |
| set1 | BT1211 | 0.00182874 | 0.000646777 | 0.0384185 | 0.01874299 | 0.0718897 | 0.04903409 |
| set1 | BT1220 | 0.00792376 | 0.002373245 | 0.664489 | 0.1978969 | 0.54564 | 0.2157165 |
| set1 | BT1223 | 0.0195877 | 0.005328655 | 0.708166 | 0.2077798 | 0.907381 | 0.3083245 |
| set1 | BT1235 | 0.000389441 | 0.000173061 | 0.0183399 | 0.009997152 | 0.0177152 | 0.01846111 |
| set1 | BT1253 | 0.00891189 | 0.002629656 | 0.954875 | 0.262265 | 0.381778 | 0.1671895 |
| set1 | BT1267 | 0.000173931 | 0.0000896 | 0.90112 | 0.2517518 | 0.0195968 | 0.01981165 |
| set1 | BT1275 | 0.00298217 | 0.000998271 | 0.125588 | 0.05048388 | 0.912755 | 0.3095408 |
| set1 | BT1276 | 0.0329786 | 0.008489878 | 0.0799079 | 0.03462151 | 0.216649 | 0.1105188 |
| set1 | BT1277 | 2.31179E-05 | 0.0000191 | 0.912999 | 0.2542202 | 0.24743 | 0.1219398 |
| set1 | BT1290 | 0.00371886 | 0.001210712 | 0.373048 | 0.1246538 | 0.0178068 | 0.01852512 |
| set1 | BT1293 | 3.11256E-05 | 0.000024 | 0.0467119 | 0.02214508 | 0.0301463 | 0.02650587 |
| set1 | BT1295 | 1.87282E-05 | 0.0000165 | 0.194564 | 0.0729434 | 0.0914273 | 0.05804839 |
| set1 | BT1297 | 0.00000101 | 0.00000256 | 0.677662 | 0.2007312 | 0.0321866 | 0.02771166 |
| set1 | BT1300 | 0.00000108 | 0.00000267 | 0.41957 | 0.1374613 | 0.0103846 | 0.01318816 |
| set1 | BT1305 | 0.000119485 | 0.0000664 | 0.206348 | 0.0764361 | 0.176927 | 0.09433873 |
| set1 | BT1308 | 0.0100652 | 0.002931114 | 0.88443 | 0.2479915 | 0.02671 | 0.02425734 |
| set1 | BT1310 | 0.0104208 | 0.003026431 | 0.0566671 | 0.02596777 | 0.447911 | 0.18791 |
| set1 | BT1311 | 0.0164052 | 0.004549823 | 0.0319641 | 0.01606117 | 0.489283 | 0.2002895 |
| set1 | BT1314 | 0.000000956 | 0.00000254 | 0.066137 | 0.02955613 | 0.975617 | 0.3232792 |
| set1 | BT1316 | 0.0192938 | 0.005263066 | 0.0311828 | 0.01574061 | 0.0968514 | 0.06075642 |
| set1 | BT1321 | 0.00172919 | 0.000617279 | 0.177829 | 0.06780574 | 0.0216598 | 0.02128168 |
| set1 | BT1322 | 0.000000205 | 0.00000108 | 0.195706 | 0.07325786 | 0.0495639 | 0.03739407 |
| set1 | BT1324 | 0.000345575 | 0.000157183 | 0.0735978 | 0.03243433 | 0.0321422 | 0.02770531 |
| set1 | BT1325 | 0.000368385 | 0.00016547 | 0.138414 | 0.05490878 | 0.025547 | 0.02348275 |
| set1 | BT1329 | 0.00412824 | 0.001329012 | 0.0445253 | 0.02126704 | 0.0947627 | 0.05975257 |
| set1 | BT1331 | 0.00000621 | 0.00000759 | 0.646232 | 0.1930784 | 0.0123115 | 0.01472884 |
| set1 | BT1332 | 0.000386878 | 0.000172344 | 0.335248 | 0.114461 | 0.00748499 | 0.01066734 |
| set1 | BT1333 | 2.15139E-05 | 0.0000182 | 0.990714 | 0.2692313 | 0.0572411 | 0.04161729 |
| set1 | BT1337 | 0.000175578 | 0.0000903 | 0.033917 | 0.01690881 | 0.0472893 | 0.03615462 |
| set1 | BT1338 | 0.000141283 | 0.0000754 | 0.117514 | 0.04790789 | 0.0110045 | 0.01371283 |
| set1 | BT1339 | 9.64019E-05 | 0.0000567 | 0.0827768 | 0.03562175 | 0.00878505 | 0.01171275 |
| set1 | BT1340 | 0.000112174 | 0.0000634 | 0.633469 | 0.1901577 | 0.113576 | 0.06791054 |
| set1 | BT1342 | 3.85235E-05 | 0.0000282 | 0.471169 | 0.1507679 | 0.0185824 | 0.01912865 |
| set1 | BT1343 | 0.00181194 | 0.000642336 | 0.854207 | 0.2414225 | 0.00795113 | 0.01102136 |
| set1 | BT1349 | 0.000180251 | 0.0000924 | 0.0193418 | 0.01045366 | 0.0146847 | 0.01648009 |
| set1 | BT1350 | 0.000312157 | 0.000144413 | 0.0942435 | 0.03961081 | 0.0176387 | 0.0184449 |
| set1 | BT1356 | 0.000394533 | 0.00017482 | 0.137725 | 0.0546534 | 0.0337273 | 0.02860136 |
| set1 | BT1357 | 0.00000629 | 0.00000761 | 0.0392572 | 0.01909817 | 0.00838593 | 0.01133505 |
| set1 | BT1359 | 0.00196715 | 0.000689287 | 0.883328 | 0.24774 | 0.953607 | 0.3186507 |
| set1 | BT1360 | 0.0147233 | 0.00413488 | 0.0329034 | 0.01647693 | 0.814312 | 0.2868998 |
| set1 | BT1362 | 5.62486E-05 | 0.0000371 | 0.327499 | 0.112037 | 0.0579731 | 0.04196244 |
| set1 | BT1367 | 0.0124319 | 0.00355602 | 0.77759 | 0.2239071 | 0.0865001 | 0.05578873 |
| set1 | BT1368 | 0.00157873 | 0.000571713 | 0.0284834 | 0.01457286 | 0.0930795 | 0.05889361 |
| set1 | BT1369 | 0.00237983 | 0.000816872 | 0.314721 | 0.1086175 | 0.0240931 | 0.02259087 |
| set1 | BT1371 | 0.00149331 | 0.000544426 | 0.151821 | 0.05933103 | 0.912959 | 0.3095408 |
| set1 | BT1372 | 0.0000056 | 0.00000709 | 0.526261 | 0.1646884 | 0.0709707 | 0.04861374 |
| set1 | BT1374 | 1.25846E-05 | 0.0000124 | 0.752437 | 0.2178587 | 0.0381825 | 0.03122497 |
| set1 | BT1378 | 0.00758964 | 0.002284019 | 0.029908 | 0.01519234 | 0.955614 | 0.318955 |
| set1 | BT1382 | 0.00318396 | 0.001054625 | 0.376148 | 0.1255854 | 0.425685 | 0.1811221 |
| set1 | BT1394 | 0.00104365 | 0.000402394 | 0.49025 | 0.1555958 | 0.0800908 | 0.05291036 |
| set1 | BT1399 | 0.00495013 | 0.001564981 | 0.0797396 | 0.03457005 | 0.0855036 | 0.05526744 |
| set1 | BT1410 | 0.00000403 | 0.00000575 | 0.633388 | 0.1901577 | 0.0129503 | 0.01517158 |
| set1 | BT1414 | 1.57805E-05 | 0.0000146 | 0.0858944 | 0.03677992 | 0.461698 | 0.1924838 |
| set1 | BT1416 | 0.00013392 | 0.0000726 | 0.605337 | 0.183954 | 0.053806 | 0.03972981 |
| set1 | BT1417 | 0.00299012 | 0.000999164 | 0.660739 | 0.1968771 | 0.266966 | 0.1292069 |
| set1 | BT1418 | 0.000252924 | 0.000122007 | 0.441481 | 0.1430491 | 0.0665759 | 0.04651501 |
| set1 | BT1427 | 0.0219242 | 0.005913609 | 0.0499987 | 0.02338719 | 0.0463504 | 0.03560368 |
| set1 | BT1429 | 0.000114924 | 0.0000645 | 0.260095 | 0.09270635 | 0.690487 | 0.2556856 |
| set1 | BT1432 | 0.00475583 | 0.001511762 | 0.0647472 | 0.02902093 | 0.579536 | 0.2253542 |
| set1 | BT1433 | 0.00136788 | 0.00050601 | 0.86838 | 0.2443419 | 0.91328 | 0.3095408 |
| set1 | BT1438 | 0.000871335 | 0.000345294 | 0.190487 | 0.07177131 | 0.12077 | 0.07074245 |
| set1 | BT1439 | 1.22086E-05 | 0.0000122 | 0.291937 | 0.1020091 | 0.0657964 | 0.04619024 |
| set1 | BT1442 | 0.000323645 | 0.000148728 | 0.231552 | 0.08434961 | 0.0138746 | 0.01587435 |
| set1 | BT1445 | 0.00575903 | 0.001790499 | 0.0282238 | 0.01445842 | 0.083616 | 0.05435778 |
| set1 | BT1455 | 0.00000114 | 0.00000275 | 0.573023 | 0.1760833 | 0.27989 | 0.1336117 |
| set1 | BT1457 | 0.00136927 | 0.000506159 | 0.830798 | 0.2362436 | 0.0436476 | 0.03420486 |
| set1 | BT1466 | 0.00822641 | 0.002452247 | 0.0341077 | 0.01697586 | 0.788661 | 0.2808565 |
| set1 | BT1467 | 0.0158295 | 0.004414423 | 0.06695 | 0.02987524 | 0.0143474 | 0.01623811 |
| set1 | BT1468 | 0.000000365 | 0.00000142 | 0.503459 | 0.1588283 | 0.0126231 | 0.01490723 |
| set1 | BT1470 | 4.78988E-05 | 0.000033 | 0.389267 | 0.12918 | 0.199035 | 0.1031664 |
| set1 | BT1471 | 0.00973713 | 0.002850933 | 0.0975827 | 0.0408296 | 0.292498 | 0.1375799 |
| set1 | BT1475 | 0.00796132 | 0.002383552 | 0.0433977 | 0.02086043 | 0.865121 | 0.298666 |
| set1 | BT1478 | 0.000831498 | 0.000330704 | 0.780552 | 0.2244389 | 0.0292779 | 0.02593699 |
| set1 | BT1481 | 0.000357591 | 0.000161773 | 0.952914 | 0.2619647 | 0.0352589 | 0.02946399 |
| set1 | BT1487 | 0.00135624 | 0.000503426 | 0.48867 | 0.1551735 | 0.540776 | 0.2142552 |
| set1 | BT1488 | 3.67841E-05 | 0.0000272 | 0.726501 | 0.2120259 | 0.0835187 | 0.05433551 |
| set1 | BT1491 | 0.0003486 | 0.000158369 | 0.970604 | 0.2652582 | 0.0451321 | 0.03498244 |
| set1 | BT1492 | 0.0012201 | 0.000459198 | 0.977069 | 0.266844 | 0.546998 | 0.2161009 |
| set1 | BT1501 | 0.00027183 | 0.000129885 | 0.0709955 | 0.0314366 | 0.0455544 | 0.03519483 |
| set1 | BT1502 | 0.00000229 | 0.00000403 | 0.19487 | 0.07303546 | 0.832968 | 0.2911261 |
| set1 | BT1505 | 0.00225834 | 0.000779768 | 0.835654 | 0.2372335 | 0.565017 | 0.2211142 |
| set1 | BT1506 | 0.00533746 | 0.001671377 | 0.522169 | 0.1637897 | 0.720941 | 0.2632545 |
| set1 | BT1511 | 0.00000373 | 0.00000551 | 0.0383749 | 0.01872928 | 0.0125497 | 0.01489244 |
| set1 | BT1516 | 0.000126091 | 0.0000693 | 0.524918 | 0.1644385 | 0.11831 | 0.06988713 |
| set1 | BT1518 | 0.00000611 | 0.00000753 | 0.0216703 | 0.01154173 | 0.207768 | 0.1067507 |
| set1 | BT1525 | 0.0283414 | 0.007427466 | 0.192255 | 0.07225715 | 0.107839 | 0.06571147 |
| set1 | BT1526 | 0.000205328 | 0.000102917 | 0.156453 | 0.06078721 | 0.0398199 | 0.03218013 |
| set1 | BT1540 | 0.00395669 | 0.001279772 | 0.045507 | 0.02165886 | 0.134094 | 0.07656132 |
| set1 | BT1544 | 0.0138558 | 0.003921058 | 0.143526 | 0.0566391 | 0.663883 | 0.2484577 |
| set1 | BT1547 | 0.00442051 | 0.001415277 | 0.0581899 | 0.02651471 | 0.16585 | 0.08996836 |
| set1 | BT1548 | 0.000264254 | 0.000126826 | 0.327327 | 0.1120338 | 0.998516 | 0.3290103 |
| set1 | BT1549 | 0.00000327 | 0.00000508 | 0.803536 | 0.2296878 | 0.0870797 | 0.05594134 |
| set1 | BT1550 | 0.000143837 | 0.0000763 | 0.153702 | 0.05991106 | 0.0445881 | 0.03470399 |
| set1 | BT1560 | 0.00810812 | 0.002421835 | 0.0638199 | 0.02866912 | 0.330987 | 0.1506957 |
| set1 | BT1561 | 0.00172984 | 0.000617279 | 0.0310733 | 0.01569396 | 0.0146355 | 0.0164732 |
| set1 | BT1573 | 0.000578501 | 0.000240839 | 0.108708 | 0.04483232 | 0.0948896 | 0.0598069 |
| set1 | BT1578 | 0.000107431 | 0.0000615 | 0.132124 | 0.05274264 | 0.0135211 | 0.01559142 |
| set1 | BT1579 | 0.00000374 | 0.00000551 | 0.401076 | 0.1324439 | 0.0134808 | 0.01558317 |
| set1 | BT1580 | 2.48161E-05 | 0.0000202 | 0.913093 | 0.2542202 | 0.034804 | 0.02921461 |
| set1 | BT1582 | 0.000536539 | 0.000226232 | 0.293819 | 0.1024001 | 0.880233 | 0.3019656 |
| set1 | BT1589 | 0.0333987 | 0.008586344 | 0.0354955 | 0.01757246 | 0.242811 | 0.1200495 |
| set1 | BT1592 | 0.0134447 | 0.003818304 | 0.566236 | 0.1747088 | 0.0586908 | 0.04238072 |
| set1 | BT1599 | 1.90868E-05 | 0.0000167 | 0.227929 | 0.08318033 | 0.582525 | 0.2263366 |
| set1 | BT1601 | 0.000139011 | 0.0000746 | 0.411553 | 0.1352264 | 0.0111202 | 0.01378209 |
| set1 | BT1615 | 0.00123872 | 0.000464819 | 0.921255 | 0.2556467 | 0.0181513 | 0.01880356 |
| set1 | BT1616 | 0.0221435 | 0.005955781 | 0.414187 | 0.135845 | 0.425548 | 0.1811163 |
| set1 | BT1624 | 0.00528911 | 0.0016578 | 0.0461008 | 0.02188973 | 0.651726 | 0.244926 |
| set1 | BT1625 | 4.54965E-05 | 0.0000319 | 0.239936 | 0.08690578 | 0.0248226 | 0.02303696 |
| set1 | BT1629 | 0.00990096 | 0.002893342 | 0.320717 | 0.1102163 | 0.957985 | 0.3194196 |
| set1 | BT1631 | 0.00212739 | 0.000739614 | 0.278369 | 0.0980338 | 0.14085 | 0.07954033 |
| set1 | BT1636 | 0.00000211 | 0.00000389 | 0.379223 | 0.1264025 | 0.0406718 | 0.03255339 |
| set1 | BT1641 | 0.000000299 | 0.00000127 | 0.1434 | 0.05660787 | 0.029708 | 0.02623883 |
| set1 | BT1660 | 0.00712761 | 0.002163037 | 0.39649 | 0.1310729 | 0.502722 | 0.2039114 |
| set1 | BT1669 | 4.45055E-05 | 0.0000313 | 0.0228272 | 0.01202011 | 0.380997 | 0.1670468 |
| set1 | BT1670 | 0.000445641 | 0.000192847 | 0.310871 | 0.1074132 | 0.051841 | 0.03867676 |
| set1 | BT1677 | 0.000386037 | 0.000172071 | 0.278194 | 0.09800074 | 0.109287 | 0.0662911 |
| set1 | BT1679 | 8.80247E-05 | 0.0000529 | 0.961673 | 0.2635927 | 0.438899 | 0.1851878 |
| set1 | BT1685 | 0.000000538 | 0.00000179 | 0.333057 | 0.1137451 | 0.00836049 | 0.01132152 |
| set1 | BT1686 | 0.000000306 | 0.00000128 | 0.176675 | 0.06742958 | 0.0266749 | 0.02424557 |
| set1 | BT1687 | 0.000000145 | 0.000000949 | 0.0202281 | 0.01087429 | 0.0231649 | 0.02204913 |
| set1 | BT1688 | 0.00000082 | 0.0000023 | 0.167276 | 0.06435067 | 0.0346648 | 0.02917725 |
| set1 | BT1689 | 0.000000298 | 0.00000127 | 0.155861 | 0.06061569 | 0.0140171 | 0.01597513 |
| set1 | BT1692 | 0.000278755 | 0.000132441 | 0.646002 | 0.1930575 | 0.224442 | 0.113804 |
| set1 | BT1693 | 4.42189E-05 | 0.0000312 | 0.491565 | 0.1558874 | 0.192486 | 0.1006604 |
| set1 | BT1694 | 0.000000787 | 0.00000225 | 0.234569 | 0.08526864 | 0.183882 | 0.09716483 |
| set1 | BT1695 | 0.00000637 | 0.00000769 | 0.418114 | 0.1370214 | 0.387874 | 0.1690522 |
| set1 | BT1697 | 1.11598E-05 | 0.0000114 | 0.803357 | 0.2296878 | 0.0275992 | 0.024794 |
| set1 | BT1698 | 8.18395E-05 | 0.0000501 | 0.222522 | 0.0814885 | 0.27939 | 0.1334789 |
| set1 | BT1701 | 0.00290349 | 0.000976715 | 0.317994 | 0.1094674 | 0.139888 | 0.07910026 |
| set1 | BT1706 | 5.12957E-05 | 0.0000347 | 0.625255 | 0.1884424 | 0.039934 | 0.03218489 |
| set1 | BT1717 | 0.0308453 | 0.008006045 | 0.273245 | 0.09660315 | 0.0354386 | 0.02954016 |
| set1 | BT1719 | 0.0069912 | 0.002126757 | 0.08184 | 0.03529406 | 0.093269 | 0.05897406 |
| set1 | BT1722 | 0.00187139 | 0.000659396 | 0.309621 | 0.1071346 | 0.0331819 | 0.0282695 |
| set1 | BT1729 | 0.00424763 | 0.001364545 | 0.0550512 | 0.02530408 | 0.46494 | 0.1935772 |
| set1 | BT1735 | 0.000435844 | 0.000189801 | 0.415375 | 0.1361977 | 0.366365 | 0.1623775 |
| set1 | BT1736 | 0.000353208 | 0.000160072 | 0.279018 | 0.0982051 | 0.201127 | 0.1039937 |
| set1 | BT1744 | 0.000000992 | 0.00000256 | 0.0330992 | 0.01656263 | 0.0158549 | 0.01726084 |
| set1 | BT1747 | 9.72266E-05 | 0.000057 | 0.978756 | 0.2671235 | 0.0289089 | 0.02568965 |
| set1 | BT1754 | 0.00138046 | 0.000509667 | 0.402341 | 0.1327165 | 0.077825 | 0.05188047 |
| set1 | BT1755 | 0.00364365 | 0.001189193 | 0.291254 | 0.1018293 | 0.255402 | 0.1249282 |
| set1 | BT1760 | 0.000308871 | 0.000143419 | 0.22464 | 0.08210407 | 0.0225334 | 0.02172491 |
| set1 | BT1762 | 0.000440932 | 0.000191576 | 0.423272 | 0.138299 | 0.00953718 | 0.01242219 |
| set1 | BT1763 | 0.00613745 | 0.001892999 | 0.0598702 | 0.02714723 | 0.053255 | 0.03948183 |
| set1 | BT1766 | 0.000015316 | 0.0000143 | 0.217145 | 0.07992004 | 0.0283072 | 0.02527535 |
| set1 | BT1769 | 2.31604E-05 | 0.0000192 | 0.550906 | 0.1710273 | 0.017181 | 0.01818336 |
| set1 | BT1778 | 8.57405E-05 | 0.0000517 | 0.175946 | 0.06721506 | 0.0767725 | 0.05145928 |
| set1 | BT1779 | 0.00231049 | 0.000796321 | 0.0593384 | 0.0269162 | 0.236726 | 0.1180349 |
| set1 | BT1780 | 0.00490109 | 0.001551184 | 0.779487 | 0.2242395 | 0.0239707 | 0.02257027 |
| set1 | BT1781 | 0.00000234 | 0.00000409 | 0.457118 | 0.1471684 | 0.014616 | 0.0164732 |
| set1 | BT1797 | 4.07739E-05 | 0.0000294 | 0.388173 | 0.1289232 | 0.464096 | 0.1934286 |
| set1 | BT1799 | 0.0286821 | 0.007503762 | 0.290546 | 0.1016483 | 0.185893 | 0.09801576 |
| set1 | BT1800 | 1.94269E-05 | 0.0000169 | 0.236835 | 0.0859372 | 0.101221 | 0.06277943 |
| set1 | BT1805 | 2.41516E-05 | 0.0000198 | 0.162478 | 0.06274468 | 0.157106 | 0.08634178 |
| set1 | BT1806 | 3.71263E-05 | 0.0000274 | 0.181114 | 0.06879771 | 0.101999 | 0.063149 |
| set1 | BT1807 | 1.66811E-05 | 0.0000152 | 0.506004 | 0.1594646 | 0.0165129 | 0.01775913 |
| set1 | BT1809 | 0.000000854 | 0.00000234 | 0.553353 | 0.1715224 | 0.0995362 | 0.06196399 |
| set1 | BT1816 | 0.00000197 | 0.00000371 | 0.950817 | 0.2614477 | 0.00721179 | 0.01040937 |
| set1 | BT1817 | 0.000248008 | 0.000120018 | 0.344929 | 0.1172691 | 0.0561281 | 0.04102684 |
| set1 | BT1827 | 0.00590116 | 0.001830476 | 0.0949396 | 0.03984792 | 0.0272669 | 0.02455558 |
| set1 | BT1830 | 0.000122984 | 0.0000679 | 0.270226 | 0.09569615 | 0.730516 | 0.2657592 |
| set1 | BT1832 | 0.00000224 | 0.000004 | 0.293455 | 0.1023914 | 0.13006 | 0.07498481 |
| set1 | BT1833 | 0.00175134 | 0.000624363 | 0.652006 | 0.194611 | 0.0873195 | 0.05602184 |
| set1 | BT1842 | 0.00000161 | 0.00000338 | 0.0367691 | 0.01808438 | 0.132466 | 0.07586788 |
| set1 | BT1846 | 0.00120666 | 0.000455045 | 0.22335 | 0.08170675 | 0.936395 | 0.3151197 |
| set1 | BT1850 | 4.63618E-05 | 0.0000323 | 0.0458267 | 0.0217767 | 0.0298557 | 0.02633759 |
| set1 | BT1852 | 0.00923683 | 0.002722353 | 0.209624 | 0.077507 | 0.581665 | 0.2260623 |
| set1 | BT1874 | 0.00000846 | 0.00000943 | 0.844589 | 0.2390397 | 0.016932 | 0.01804029 |
| set1 | BT1880 | 0.000540163 | 0.00022738 | 0.461798 | 0.1484169 | 0.306393 | 0.1422841 |
| set1 | BT1881 | 0.00000894 | 0.00000974 | 0.04857 | 0.02287335 | 0.207359 | 0.1066525 |
| set1 | BT1882 | 0.00487578 | 0.001545996 | 0.0669824 | 0.02987866 | 0.459356 | 0.1917797 |
| set1 | BT1883 | 0.000205126 | 0.000102917 | 0.413871 | 0.1357832 | 0.70235 | 0.2583942 |
| set1 | BT1884 | 0.00211668 | 0.000737245 | 0.186027 | 0.07030898 | 0.0730564 | 0.04968427 |
| set1 | BT1889 | 0.0322079 | 0.00831126 | 0.968911 | 0.2649754 | 0.696531 | 0.2570911 |
| set1 | BT1890 | 0.00641689 | 0.001970263 | 0.797601 | 0.2283627 | 0.0999697 | 0.06218112 |
| set1 | BT1898 | 0.00625525 | 0.001923754 | 0.0616952 | 0.0278389 | 0.66417 | 0.2484577 |
| set1 | BT1899 | 0.0109391 | 0.003157661 | 0.491461 | 0.1558874 | 0.132579 | 0.07588215 |
| set1 | BT1900 | 0.0250114 | 0.006653839 | 0.258954 | 0.0924489 | 0.188041 | 0.09882885 |
| set1 | BT1902 | 0.00182104 | 0.000644656 | 0.632876 | 0.1901577 | 0.0730032 | 0.04968427 |
| set1 | BT1904 | 0.0166577 | 0.00461309 | 0.172999 | 0.06621489 | 0.0602195 | 0.0432896 |
| set1 | BT1908 | 0.00963375 | 0.002825036 | 0.0224834 | 0.01187531 | 0.528926 | 0.2112709 |
| set1 | BT1917 | 0.00156425 | 0.000567828 | 0.117424 | 0.04789103 | 0.0866293 | 0.05579851 |
| set1 | BT1921 | 0.000195999 | 0.0000992 | 0.640958 | 0.1917873 | 0.0602638 | 0.04330024 |
| set1 | BT1923 | 0.000000695 | 0.00000208 | 0.154986 | 0.06035312 | 0.0136981 | 0.01572143 |
| set1 | BT1941 | 0.00105775 | 0.000407622 | 0.197492 | 0.07383489 | 0.0708204 | 0.04854577 |
| set1 | BT1943 | 0.0137942 | 0.003908762 | 0.257979 | 0.09216972 | 0.104651 | 0.06424893 |
| set1 | BT1945 | 0.038102 | 0.009680411 | 0.705566 | 0.2072626 | 0.240353 | 0.119173 |
| set1 | BT1946 | 0.000405949 | 0.000178944 | 0.549421 | 0.1708297 | 0.578215 | 0.2249597 |
| set1 | BT1948 | 0.000711079 | 0.000287187 | 0.0271954 | 0.01399689 | 0.013199 | 0.01537704 |
| set1 | BT1950 | 0.00000667 | 0.00000794 | 0.504872 | 0.1591909 | 0.103532 | 0.06374857 |
| set1 | BT1952 | 0.0000003 | 0.00000127 | 0.640572 | 0.1917193 | 0.109672 | 0.06641486 |
| set1 | BT1954 | 0.000000294 | 0.00000127 | 0.772947 | 0.2229169 | 0.0194793 | 0.01975799 |
| set1 | BT1955 | 0.0000014 | 0.00000313 | 0.107179 | 0.04433798 | 0.678484 | 0.252404 |
| set1 | BT1956 | 0.000000925 | 0.00000249 | 0.699772 | 0.2058168 | 0.232479 | 0.1166 |
| set1 | BT1957 | 2.36E-08 | 0.000000385 | 0.0637028 | 0.02862716 | 0.525465 | 0.2104037 |
| set1 | BT1969 | 0.00184107 | 0.000650834 | 0.162245 | 0.06271485 | 0.12779 | 0.07385251 |
| set1 | BT1970 | 0.00711058 | 0.002158735 | 0.296888 | 0.1032314 | 0.0658316 | 0.04619286 |
| set1 | BT1976 | 0.000473727 | 0.000203488 | 0.357568 | 0.1203132 | 0.0619281 | 0.04417174 |
| set1 | BT1979 | 0.00128686 | 0.000481213 | 0.0610692 | 0.02759767 | 0.0698617 | 0.04812357 |
| set1 | BT1986 | 0.0385441 | 0.009786164 | 0.0190329 | 0.01030515 | 0.823891 | 0.2891239 |
| set1 | BT1990 | 0.00604259 | 0.001867458 | 0.0967802 | 0.04055001 | 0.0412873 | 0.03283121 |
| set1 | BT1995 | 0.00000117 | 0.00000278 | 0.340754 | 0.1161111 | 0.0148506 | 0.01660736 |
| set1 | BT1998 | 0.00000817 | 0.00000923 | 0.0205489 | 0.01102694 | 0.373198 | 0.1644131 |
| set1 | BT1999 | 0.000115837 | 0.0000648 | 0.273267 | 0.09660315 | 0.985812 | 0.3258483 |
| set1 | BT2009 | 4.94472E-05 | 0.0000338 | 0.437811 | 0.1421268 | 0.0349286 | 0.02928176 |
| set1 | BT2016 | 2.24687E-05 | 0.0000188 | 0.401445 | 0.1324933 | 0.0587242 | 0.04238072 |
| set1 | BT2017 | 4.82193E-05 | 0.0000332 | 0.496746 | 0.1569564 | 0.00823977 | 0.01122014 |
| set1 | BT2020 | 0.00528972 | 0.0016578 | 0.109235 | 0.04500356 | 0.260589 | 0.1269156 |
| set1 | BT2027 | 0.00000324 | 0.00000506 | 0.420022 | 0.1375348 | 0.0228904 | 0.02191863 |
| set1 | BT2028 | 0.0106309 | 0.003080164 | 0.140384 | 0.05558076 | 0.271386 | 0.1307855 |
| set1 | BT2031 | 0.0358825 | 0.009162649 | 0.0726594 | 0.03203246 | 0.0830183 | 0.05413778 |
| set1 | BT2035 | 0.0268685 | 0.007085623 | 0.708735 | 0.20781 | 0.0285136 | 0.02544182 |
| set1 | BT2046 | 0.000021697 | 0.0000183 | 0.128824 | 0.05163006 | 0.534942 | 0.2125749 |
| set1 | BT2052 | 3.03665E-05 | 0.0000236 | 0.33901 | 0.1155494 | 0.0503312 | 0.03775275 |
| set1 | BT2053 | 2.38102E-05 | 0.0000196 | 0.296003 | 0.1030127 | 0.307789 | 0.1427109 |
| set1 | BT2054 | 0.00000777 | 0.00000889 | 0.0775644 | 0.03384897 | 0.252049 | 0.1236095 |
| set1 | BT2055 | 0.0000087 | 0.00000958 | 0.0181462 | 0.009917321 | 0.209307 | 0.1074286 |
| set1 | BT2056 | 1.00006E-05 | 0.0000106 | 0.315655 | 0.1087862 | 0.00897715 | 0.01189326 |
| set1 | BT2059 | 0.000568356 | 0.000237345 | 0.468438 | 0.150053 | 0.0315043 | 0.02738056 |
| set1 | BT2066 | 0.000049848 | 0.000034 | 0.670963 | 0.1994807 | 0.734308 | 0.2667421 |
| set1 | BT2067 | 0.00259162 | 0.00088316 | 0.226096 | 0.08253633 | 0.0846978 | 0.05489162 |
| set1 | BT2070 | 0.0274265 | 0.007215178 | 0.543602 | 0.1694566 | 0.762746 | 0.2741551 |
| set1 | BT2074 | 0.000568885 | 0.000237358 | 0.702027 | 0.2063796 | 0.131309 | 0.07532278 |
| set1 | BT2076 | 0.000109719 | 0.0000625 | 0.961087 | 0.2634919 | 0.759568 | 0.2733475 |
| set1 | BT2081 | 0.00374566 | 0.001217761 | 0.138872 | 0.05505431 | 0.264217 | 0.1284269 |
| set1 | BT2082 | 9.07643E-05 | 0.0000542 | 0.594109 | 0.1811441 | 0.720652 | 0.2632545 |
| set1 | BT2086 | 0.000336382 | 0.000153834 | 0.0415639 | 0.02009089 | 0.0237257 | 0.02241968 |
| set1 | BT2090 | 0.00000242 | 0.00000414 | 0.0377025 | 0.01844583 | 0.197361 | 0.1024637 |
| set1 | BT2091 | 0.00000636 | 0.00000769 | 0.0211044 | 0.01128511 | 0.740988 | 0.2687626 |
| set1 | BT2094 | 0.021454 | 0.005795043 | 0.232595 | 0.08462747 | 0.126234 | 0.07324162 |
| set1 | BT2099 | 0.00300138 | 0.001001834 | 0.978665 | 0.2671235 | 0.032871 | 0.02815167 |
| set1 | BT2102 | 0.000652468 | 0.000267368 | 0.261293 | 0.09301573 | 0.22824 | 0.1151729 |
| set1 | BT2111 | 0.000360343 | 0.000162533 | 0.232588 | 0.08462747 | 0.0513837 | 0.03842441 |
| set1 | BT2118 | 0.0296744 | 0.00773495 | 0.0501129 | 0.02342176 | 0.0173378 | 0.01826538 |
| set1 | BT2123 | 0.000216571 | 0.000107414 | 0.358095 | 0.1204234 | 0.0261646 | 0.02387043 |
| set1 | BT2124 | 0.00000847 | 0.00000943 | 0.901109 | 0.2517518 | 0.170619 | 0.09191005 |
| set1 | BT2129 | 0.000545243 | 0.000229263 | 0.260021 | 0.09270635 | 0.0156245 | 0.01707781 |
| set1 | BT2136 | 0.00305953 | 0.001019208 | 0.932743 | 0.2578057 | 0.0858065 | 0.05541443 |
| set1 | BT2143 | 1.00436E-05 | 0.0000106 | 0.0982971 | 0.04107161 | 0.0103609 | 0.01317934 |
| set1 | BT2145 | 2.77541E-05 | 0.000022 | 0.889214 | 0.2490439 | 0.0096147 | 0.01250098 |
| set1 | BT2147 | 0.00065671 | 0.00026809 | 0.0631608 | 0.02840473 | 0.30606 | 0.1422195 |
| set1 | BT2154 | 0.00000205 | 0.0000038 | 0.169614 | 0.06514635 | 0.0109217 | 0.0136444 |
| set1 | BT2161 | 0.000536965 | 0.000226285 | 0.58505 | 0.1789596 | 0.0123683 | 0.01475587 |
| set1 | BT2162 | 0.00110267 | 0.000422139 | 0.473844 | 0.1513032 | 0.0101008 | 0.01294948 |
| set1 | BT2168 | 0.000084921 | 0.0000513 | 0.586775 | 0.179351 | 0.45011 | 0.1885744 |
| set1 | BT2169 | 0.00232828 | 0.000800994 | 0.0446473 | 0.02130005 | 0.0730524 | 0.04968427 |
| set1 | BT2179 | 0.00000037 | 0.00000142 | 0.0521838 | 0.02422576 | 0.0135644 | 0.01562908 |
| set1 | BT2180 | 2.30792E-05 | 0.0000191 | 0.355502 | 0.1198517 | 0.0276592 | 0.0248327 |
| set1 | BT2190 | 0.00165587 | 0.000595375 | 0.915018 | 0.2544421 | 0.024031 | 0.02259087 |
| set1 | BT2192 | 0.00244639 | 0.000837819 | 0.627716 | 0.1890426 | 0.133302 | 0.07619803 |
| set1 | BT2193 | 0.0040539 | 0.001307861 | 0.143786 | 0.05672317 | 0.0393065 | 0.03191303 |
| set1 | BT2202 | 0.000156886 | 0.0000823 | 0.827105 | 0.2353597 | 0.622064 | 0.2369945 |
| set1 | BT2207 | 0.000000805 | 0.00000227 | 0.358983 | 0.1206549 | 0.12712 | 0.07359855 |
| set1 | BT2211 | 1.70488E-05 | 0.0000154 | 0.129035 | 0.05169747 | 0.0187983 | 0.01924295 |
| set1 | BT2213 | 0.000000581 | 0.00000189 | 0.0725656 | 0.03200279 | 0.0186047 | 0.01913819 |
| set1 | BT2220 | 0.000000601 | 0.0000019 | 0.176221 | 0.06729883 | 0.0907697 | 0.05772775 |
| set1 | BT2233 | 0.000300542 | 0.00014024 | 0.214309 | 0.07906983 | 0.929701 | 0.3137692 |
| set1 | BT2238 | 0.0186021 | 0.005093783 | 0.653151 | 0.1948084 | 0.0791101 | 0.05242764 |
| set1 | BT2244 | 0.000060457 | 0.0000392 | 0.0279279 | 0.01433115 | 0.0112584 | 0.01385283 |
| set1 | BT2245 | 0.00847032 | 0.002515049 | 0.778343 | 0.2239637 | 0.12239 | 0.07152015 |
| set1 | BT2246 | 0.000477313 | 0.000204762 | 0.082371 | 0.03549771 | 0.119446 | 0.07024722 |
| set1 | BT2248 | 0.00128318 | 0.000480074 | 0.0367673 | 0.01808438 | 0.581433 | 0.2260319 |
| set1 | BT2249 | 0.00100439 | 0.000388841 | 0.0221687 | 0.01175535 | 0.943385 | 0.3168183 |
| set1 | BT2251 | 0.000055523 | 0.0000367 | 0.0500096 | 0.02338719 | 0.278604 | 0.1333447 |
| set1 | BT2257 | 0.0194831 | 0.00530623 | 0.968602 | 0.2649509 | 0.014785 | 0.01657997 |
| set1 | BT2266 | 0.0102453 | 0.002976689 | 0.060335 | 0.02731697 | 0.527875 | 0.2111959 |
| set1 | BT2272 | 0.0243057 | 0.006482049 | 0.342168 | 0.1165272 | 0.482083 | 0.1987248 |
| set1 | BT2284 | 0.0351829 | 0.008990071 | 0.179433 | 0.06830953 | 0.844004 | 0.2937941 |
| set1 | BT2290 | 0.000245961 | 0.000119332 | 0.765268 | 0.2210471 | 0.0268241 | 0.02429071 |
| set1 | BT2296 | 0.0161655 | 0.004493224 | 0.318142 | 0.1094871 | 0.530235 | 0.211506 |
| set1 | BT2297 | 1.62312E-05 | 0.0000148 | 0.0767098 | 0.03351234 | 0.0190669 | 0.01945008 |
| set1 | BT2299 | 0.00000757 | 0.00000871 | 0.0507504 | 0.0236421 | 0.119809 | 0.07032928 |
| set1 | BT2302 | 0.00100861 | 0.000390103 | 0.0224037 | 0.01184875 | 0.430783 | 0.1828671 |
| set1 | BT2303 | 0.00000137 | 0.00000308 | 0.253468 | 0.0908806 | 0.289645 | 0.1366684 |
| set1 | BT2309 | 0.00454695 | 0.001453913 | 0.268674 | 0.09527597 | 0.231511 | 0.1164232 |
| set1 | BT2319 | 3.94732E-05 | 0.0000288 | 0.748162 | 0.2171414 | 0.0672642 | 0.04683985 |
| set1 | BT2322 | 0.000534044 | 0.000225431 | 0.857369 | 0.2421462 | 0.046152 | 0.03554428 |
| set1 | BT2323 | 5.16432E-05 | 0.0000349 | 0.0935139 | 0.03931784 | 0.331205 | 0.1506957 |
| set1 | BT2333 | 0.0351309 | 0.00898285 | 0.328477 | 0.1123397 | 0.881914 | 0.3023978 |
| set1 | BT2334 | 1.45658E-05 | 0.0000138 | 0.0671227 | 0.02991941 | 0.315425 | 0.145466 |
| set1 | BT2337 | 0.00145802 | 0.000534392 | 0.159331 | 0.06170694 | 0.477712 | 0.1974219 |
| set1 | BT2338 | 4.09291E-05 | 0.0000294 | 0.177474 | 0.06771313 | 0.266399 | 0.1290175 |
| set1 | BT2339 | 5.46017E-05 | 0.0000364 | 0.116065 | 0.04745332 | 0.176229 | 0.09410328 |
| set1 | BT2342 | 0.0145454 | 0.004094034 | 0.107605 | 0.04446852 | 0.976591 | 0.3233829 |
| set1 | BT2345 | 0.00626255 | 0.001925217 | 0.0491339 | 0.02308491 | 0.642563 | 0.2424134 |
| set1 | BT2347 | 0.000320755 | 0.000147669 | 0.377357 | 0.1259195 | 0.226204 | 0.1144602 |
| set1 | BT2349 | 0.000095573 | 0.0000565 | 0.0389374 | 0.01897042 | 0.104713 | 0.06426012 |
| set1 | BT2356 | 0.000679483 | 0.000276492 | 0.0243314 | 0.01271792 | 0.489161 | 0.2002895 |
| set1 | BT2368 | 0.00000133 | 0.00000303 | 0.203267 | 0.07548 | 0.348033 | 0.156414 |
| set1 | BT2375 | 0.0101232 | 0.00294687 | 0.337327 | 0.1150407 | 0.753429 | 0.2720051 |
| set1 | BT2379 | 0.000511735 | 0.000217225 | 0.0511203 | 0.02376858 | 0.0230197 | 0.02197078 |
| set1 | BT2383 | 0.000000536 | 0.00000179 | 0.303412 | 0.1052877 | 0.433157 | 0.1836094 |
| set1 | BT2384 | 0.028005 | 0.007346937 | 0.193417 | 0.07262609 | 0.040189 | 0.03228996 |
| set1 | BT2387 | 0.000286694 | 0.000135195 | 0.913843 | 0.2542909 | 0.00950421 | 0.01242219 |
| set1 | BT2391 | 0.0298325 | 0.007769813 | 0.552123 | 0.171273 | 0.643719 | 0.2426009 |
| set1 | BT2410 | 0.0151131 | 0.004233356 | 0.0217499 | 0.01157902 | 0.439511 | 0.1853077 |
| set1 | BT2412 | 0.00179985 | 0.000638948 | 0.19358 | 0.07265827 | 0.0216515 | 0.02128168 |
| set1 | BT2415 | 4.40309E-05 | 0.0000311 | 0.488558 | 0.1551735 | 0.0267639 | 0.02426917 |
| set1 | BT2417 | 2.71E-08 | 0.000000402 | 0.201852 | 0.0751162 | 0.322968 | 0.1480146 |
| set1 | BT2419 | 1.02074E-05 | 0.0000107 | 0.124362 | 0.0501245 | 0.0303701 | 0.02661543 |
| set1 | BT2420 | 0.000000212 | 0.00000108 | 0.578304 | 0.1774803 | 0.0203893 | 0.02040207 |
| set1 | BT2431 | 0.00674623 | 0.002058856 | 0.431821 | 0.1406738 | 0.906487 | 0.3080921 |
| set1 | BT2434 | 0.000932856 | 0.000365069 | 0.179227 | 0.06827414 | 0.17775 | 0.09467439 |
| set1 | BT2437 | 0.000000117 | 0.000000902 | 0.0224641 | 0.0118703 | 0.479873 | 0.1979805 |
| set1 | BT2438 | 0.000300123 | 0.000140131 | 0.362315 | 0.1215043 | 0.131472 | 0.07538682 |
| set1 | BT2443 | 0.00000437 | 0.00000602 | 0.0975069 | 0.04081202 | 0.0294892 | 0.02609268 |
| set1 | BT2445 | 0.00458373 | 0.001463818 | 0.12864 | 0.05157342 | 0.218392 | 0.1112359 |
| set1 | BT2453 | 0.0159142 | 0.004433338 | 0.272436 | 0.09639401 | 0.472503 | 0.1957652 |
| set1 | BT2456 | 0.000000177 | 0.000001 | 0.088264 | 0.03759468 | 0.807115 | 0.2847343 |
| set1 | BT2457 | 0.0106491 | 0.003082178 | 0.974529 | 0.2662104 | 0.00807107 | 0.01111414 |
| set1 | BT2465 | 0.00009702 | 0.000057 | 0.844338 | 0.2390247 | 0.0111798 | 0.01380239 |
| set1 | BT2467 | 0.00205864 | 0.000718352 | 0.158143 | 0.06132548 | 0.177945 | 0.09474388 |
| set1 | BT2470 | 0.00233414 | 0.000802645 | 0.672114 | 0.1995881 | 0.726177 | 0.2645741 |
| set1 | BT2475 | 1.26098E-05 | 0.0000124 | 0.882181 | 0.2474758 | 0.0274427 | 0.02468362 |
| set1 | BT2478 | 0.00000123 | 0.00000289 | 0.568739 | 0.1753183 | 0.190523 | 0.09984732 |
| set1 | BT2480 | 0.00498416 | 0.001573767 | 0.116124 | 0.04745332 | 0.276663 | 0.1326752 |
| set1 | BT2481 | 0.000636763 | 0.000261783 | 0.225945 | 0.08250615 | 0.159232 | 0.08718377 |
| set1 | BT2482 | 0.00876135 | 0.002589274 | 0.0236098 | 0.01238899 | 0.821516 | 0.2886355 |
| set1 | BT2483 | 0.000139454 | 0.0000747 | 0.0211696 | 0.01131497 | 0.181956 | 0.09659916 |
| set1 | BT2493 | 0.00000959 | 0.0000102 | 0.17389 | 0.06651377 | 0.0206588 | 0.02060153 |
| set1 | BT2499 | 0.00265377 | 0.000902713 | 0.553625 | 0.1715317 | 0.827125 | 0.28967 |
| set1 | BT2510 | 0.00000225 | 0.000004 | 0.502544 | 0.1586132 | 0.0180732 | 0.01873587 |
| set1 | BT2515 | 0.0310204 | 0.008043218 | 0.120104 | 0.04873432 | 0.748884 | 0.2706972 |
| set1 | BT2517 | 0.00000148 | 0.00000325 | 0.039651 | 0.01925872 | 0.331137 | 0.1506957 |
| set1 | BT2518 | 0.00000305 | 0.00000486 | 0.471121 | 0.1507679 | 0.28599 | 0.1354226 |
| set1 | BT2519 | 0.000000238 | 0.00000115 | 0.888378 | 0.2489252 | 0.255137 | 0.1248402 |
| set1 | BT2523 | 0.000582115 | 0.000241679 | 0.207497 | 0.0768146 | 0.94757 | 0.3175699 |
| set1 | BT2531 | 0.00272318 | 0.000923005 | 0.0546174 | 0.0251238 | 0.198134 | 0.1028083 |
| set1 | BT2532 | 0.00000508 | 0.00000668 | 0.0506884 | 0.02364057 | 0.0322062 | 0.02771166 |
| set1 | BT2535 | 0.0138634 | 0.003921058 | 0.319656 | 0.1099455 | 0.025107 | 0.02323773 |
| set1 | BT2544 | 0.000126664 | 0.0000695 | 0.859705 | 0.2425791 | 0.0608002 | 0.04353648 |
| set1 | BT2552 | 0.0376517 | 0.009582088 | 0.684269 | 0.202341 | 0.0375894 | 0.03086029 |
| set1 | BT2555 | 5.93281E-05 | 0.0000387 | 0.137539 | 0.05459753 | 0.503799 | 0.2042918 |
| set1 | BT2563 | 0.000581743 | 0.000241679 | 0.102436 | 0.04258004 | 0.184803 | 0.09758124 |
| set1 | BT2568 | 0.000456926 | 0.000197279 | 0.917219 | 0.2548195 | 0.0223689 | 0.02164518 |
| set1 | BT2570 | 0.00000624 | 0.0000076 | 0.137384 | 0.05457187 | 0.12271 | 0.0716501 |
| set1 | BT2573 | 3.48972E-05 | 0.0000262 | 0.241168 | 0.08728979 | 0.026894 | 0.02432422 |
| set1 | BT2574 | 0.000156257 | 0.0000821 | 0.68339 | 0.2021305 | 0.843654 | 0.2937419 |
| set1 | BT2576 | 0.000273278 | 0.000130412 | 0.181627 | 0.0689059 | 0.958094 | 0.3194196 |
| set1 | BT2582 | 7.23E-08 | 0.000000684 | 0.430281 | 0.1402477 | 0.0466952 | 0.03577494 |
| set1 | BT2585 | 0.000139145 | 0.0000746 | 0.115348 | 0.04723209 | 0.0559298 | 0.04094784 |
| set1 | BT2589 | 0.0375219 | 0.009552267 | 0.633037 | 0.1901577 | 0.497154 | 0.2021556 |
| set1 | BT2596 | 0.00068242 | 0.000277209 | 0.0436831 | 0.02095592 | 0.137057 | 0.07776907 |
| set1 | BT2597 | 0.00462963 | 0.001476608 | 0.359306 | 0.1207298 | 0.551993 | 0.2172827 |
| set1 | BT2598 | 0.00297496 | 0.00099718 | 0.242788 | 0.08772834 | 0.181612 | 0.09645142 |
| set1 | BT2607 | 0.00405202 | 0.001307812 | 0.505361 | 0.1593035 | 0.0339711 | 0.02874169 |
| set1 | BT2608 | 8.73348E-05 | 0.0000526 | 0.2028 | 0.07532974 | 0.0167679 | 0.01791406 |
| set1 | BT2609 | 0.00926058 | 0.002728291 | 0.98473 | 0.2681481 | 0.162326 | 0.08851465 |
| set1 | BT2624 | 0.00169755 | 0.000607476 | 0.542526 | 0.1691648 | 0.0112878 | 0.01386103 |
| set1 | BT2625 | 0.000654479 | 0.000267612 | 0.085768 | 0.03673882 | 0.0218093 | 0.02131325 |
| set1 | BT2626 | 0.00122373 | 0.000460163 | 0.435939 | 0.1417477 | 0.0501157 | 0.03761963 |
| set1 | BT2630 | 0.000121637 | 0.0000673 | 0.163945 | 0.06323034 | 0.0548277 | 0.0402816 |
| set1 | BT2633 | 0.00270801 | 0.000919098 | 0.929489 | 0.2572226 | 0.11931 | 0.07019536 |
| set1 | BT2637 | 0.00000236 | 0.0000041 | 0.131667 | 0.05262978 | 0.346487 | 0.1558734 |
| set1 | BT2641 | 0.0303445 | 0.007895012 | 0.204934 | 0.07595891 | 0.486036 | 0.1996255 |
| set1 | BT2646 | 0.000452764 | 0.000195594 | 0.180497 | 0.06866224 | 0.0301548 | 0.02650587 |
| set1 | BT2649 | 0.000125596 | 0.0000691 | 0.186939 | 0.0706107 | 0.0324323 | 0.02786377 |
| set1 | BT2651 | 0.0321576 | 0.00830111 | 0.846943 | 0.2396498 | 0.13465 | 0.07681901 |
| set1 | BT2652 | 0.000224567 | 0.000110799 | 0.495775 | 0.1568055 | 0.0329956 | 0.02815223 |
| set1 | BT2655 | 0.000000248 | 0.00000119 | 0.0319474 | 0.01605946 | 0.0275838 | 0.024794 |
| set1 | BT2656 | 0.00666695 | 0.002035482 | 0.21843 | 0.08024714 | 0.00697365 | 0.01018582 |
| set1 | BT2657 | 0.000041671 | 0.0000298 | 0.195575 | 0.07323152 | 0.28868 | 0.1363007 |
| set1 | BT2682 | 0.0108276 | 0.003127861 | 0.109143 | 0.04499636 | 0.113789 | 0.0680102 |
| set1 | BT2692 | 0.00139753 | 0.000515241 | 0.0477242 | 0.02251893 | 0.0576218 | 0.04176995 |
| set1 | BT2700 | 0.000231323 | 0.000113467 | 0.512181 | 0.1612429 | 0.0304503 | 0.0266698 |
| set1 | BT2701 | 0.000111189 | 0.0000629 | 0.25558 | 0.091506 | 0.106471 | 0.06506695 |
| set1 | BT2702 | 7.47444E-05 | 0.0000465 | 0.584343 | 0.1789245 | 0.0229931 | 0.02195967 |
| set1 | BT2703 | 4.06838E-05 | 0.0000294 | 0.183361 | 0.06949827 | 0.0530638 | 0.03937986 |
| set1 | BT2704 | 3.99216E-05 | 0.000029 | 0.390442 | 0.1295343 | 0.0265262 | 0.02412535 |
| set1 | BT2705 | 8.38301E-05 | 0.000051 | 0.260713 | 0.09287182 | 0.0461105 | 0.03553096 |
| set1 | BT2706 | 4.92032E-05 | 0.0000337 | 0.371638 | 0.1243202 | 0.0617434 | 0.04409583 |
| set1 | BT2707 | 9.74129E-05 | 0.000057 | 0.147292 | 0.05787945 | 0.103269 | 0.06366675 |
| set1 | BT2708 | 0.000113217 | 0.0000638 | 0.280426 | 0.09855708 | 0.0888477 | 0.05679205 |
| set1 | BT2709 | 0.000141544 | 0.0000754 | 0.166361 | 0.0640803 | 0.117994 | 0.0697823 |
| set1 | BT2710 | 9.01541E-05 | 0.0000539 | 0.214083 | 0.07901058 | 0.110946 | 0.06688346 |
| set1 | BT2711 | 0.000100205 | 0.0000583 | 0.392558 | 0.1300221 | 0.0831553 | 0.05420304 |
| set1 | BT2713 | 4.11886E-05 | 0.0000295 | 0.402713 | 0.1327667 | 0.131924 | 0.07561646 |
| set1 | BT2714 | 0.000121467 | 0.0000673 | 0.241956 | 0.08750623 | 0.0499596 | 0.03760854 |
| set1 | BT2715 | 0.00014392 | 0.0000763 | 0.466239 | 0.1495469 | 0.0547904 | 0.0402816 |
| set1 | BT2716 | 0.000240883 | 0.000117699 | 0.083709 | 0.03595886 | 0.0825189 | 0.05402813 |
| set1 | BT2717 | 0.00013591 | 0.0000733 | 0.203868 | 0.07565666 | 0.0560624 | 0.04100402 |
| set1 | BT2718 | 0.000105507 | 0.0000606 | 0.277391 | 0.09780343 | 0.0394661 | 0.03201961 |
| set1 | BT2719 | 0.000116456 | 0.000065 | 0.188668 | 0.07121924 | 0.0780573 | 0.05198811 |
| set1 | BT2720 | 0.000221896 | 0.000109696 | 0.191002 | 0.07188772 | 0.146035 | 0.08157061 |
| set1 | BT2721 | 0.000326765 | 0.000149888 | 0.112351 | 0.04614567 | 0.186637 | 0.09830212 |
| set1 | BT2722 | 0.000244628 | 0.000118794 | 0.124534 | 0.05017708 | 0.0593405 | 0.04279179 |
| set1 | BT2723 | 0.0011812 | 0.000447003 | 0.19725 | 0.07376724 | 0.0950078 | 0.05985569 |
| set1 | BT2724 | 0.000427008 | 0.000186705 | 0.178862 | 0.0681566 | 0.107237 | 0.06548055 |
| set1 | BT2725 | 0.0006282 | 0.000258403 | 0.0741865 | 0.03263731 | 0.0771625 | 0.05164994 |
| set1 | BT2727 | 0.000261927 | 0.000126028 | 0.0315895 | 0.01592594 | 0.175427 | 0.09387993 |
| set1 | BT2728 | 0.00057674 | 0.000240238 | 0.0180202 | 0.00986741 | 0.136666 | 0.07761353 |
| set1 | BT2731 | 0.0121288 | 0.003471946 | 0.0505363 | 0.02358784 | 0.0435069 | 0.03417047 |
| set1 | BT2732 | 3.93036E-05 | 0.0000287 | 0.25592 | 0.0915695 | 0.30672 | 0.1423459 |
| set1 | BT2734 | 4.84557E-05 | 0.0000333 | 0.446713 | 0.1444246 | 0.0261062 | 0.02383196 |
| set1 | BT2735 | 0.000378315 | 0.000169027 | 0.611076 | 0.1849999 | 0.0250926 | 0.02323773 |
| set1 | BT2736 | 0.00372899 | 0.001213383 | 0.280083 | 0.09847713 | 0.0212277 | 0.02104021 |
| set1 | BT2737 | 0.00136962 | 0.000506159 | 0.39443 | 0.1304686 | 0.0338324 | 0.02865464 |
| set1 | BT2738 | 0.000887846 | 0.000350353 | 0.31662 | 0.1090565 | 0.036086 | 0.0298842 |
| set1 | BT2739 | 0.000463713 | 0.000199867 | 0.462598 | 0.1485763 | 0.0383422 | 0.03132579 |
| set1 | BT2740 | 1.05674E-05 | 0.000011 | 0.108499 | 0.0447767 | 0.176784 | 0.09433101 |
| set1 | BT2743 | 0.00000174 | 0.00000352 | 0.0635308 | 0.02856049 | 0.26617 | 0.1289686 |
| set1 | BT2750 | 0.000048339 | 0.0000332 | 0.901175 | 0.2517518 | 0.879945 | 0.3019374 |
| set1 | BT2751 | 0.00644103 | 0.001976873 | 0.0777209 | 0.03388054 | 0.402869 | 0.1738328 |
| set1 | BT2752 | 0.0162677 | 0.004513343 | 0.929835 | 0.2572594 | 0.953664 | 0.3186507 |
| set1 | BT2757 | 0.00000852 | 0.00000946 | 0.228485 | 0.08333289 | 0.0247918 | 0.02303314 |
| set1 | BT2759 | 0.00611481 | 0.001887466 | 0.0342972 | 0.01706314 | 0.778712 | 0.2783263 |
| set1 | BT2761 | 0.00000889 | 0.00000971 | 0.0640679 | 0.02875914 | 0.0741908 | 0.05018714 |
| set1 | BT2765 | 0.0226764 | 0.006079667 | 0.0426668 | 0.0205418 | 0.833697 | 0.2913115 |
| set1 | BT2768 | 0.00873 | 0.002584045 | 0.0818016 | 0.0352901 | 0.0982664 | 0.06135565 |
| set1 | BT2770 | 0.000217351 | 0.00010766 | 0.0757214 | 0.03314401 | 0.119854 | 0.07032928 |
| set1 | BT2782 | 0.000000615 | 0.00000194 | 0.0290587 | 0.01481074 | 0.00863765 | 0.01155822 |
| set1 | BT2784 | 0.00541684 | 0.001692034 | 0.067342 | 0.03000582 | 0.964178 | 0.3209966 |
| set1 | BT2787 | 0.00120741 | 0.000455101 | 0.0842141 | 0.03612445 | 0.0161995 | 0.01756245 |
| set1 | BT2791 | 3.67179E-05 | 0.0000272 | 0.954558 | 0.2622376 | 0.00736967 | 0.01056453 |
| set1 | BT2796 | 0.000125547 | 0.0000691 | 0.0801561 | 0.0347166 | 0.124842 | 0.07263494 |
| set1 | BT2800 | 0.0148026 | 0.004152528 | 0.256164 | 0.09162967 | 0.142818 | 0.08032386 |
| set1 | BT2804 | 0.000507357 | 0.00021573 | 0.861892 | 0.2430826 | 0.37161 | 0.1639829 |
| set1 | BT2805 | 0.000200189 | 0.000101038 | 0.584846 | 0.1789425 | 0.342294 | 0.1542708 |
| set1 | BT2808 | 0.00000144 | 0.00000319 | 0.189147 | 0.07137775 | 0.526103 | 0.2106018 |
| set1 | BT2810 | 6.56893E-05 | 0.000042 | 0.275432 | 0.09725465 | 0.0494107 | 0.03733221 |
| set1 | BT2811 | 1.83011E-05 | 0.0000163 | 0.31001 | 0.1072385 | 0.702323 | 0.2583942 |
| set1 | BT2812 | 0.017148 | 0.004740199 | 0.314806 | 0.1086175 | 0.05298 | 0.03933756 |
| set1 | BT2817 | 0.00000382 | 0.00000556 | 0.839387 | 0.2378461 | 0.611891 | 0.2340298 |
| set1 | BT2818 | 1.03944E-05 | 0.0000108 | 0.0196081 | 0.0105645 | 0.153283 | 0.08458911 |
| set1 | BT2819 | 0.000000495 | 0.00000171 | 0.0842676 | 0.03613457 | 0.0296164 | 0.02617368 |
| set1 | BT2825 | 0.000989226 | 0.000384347 | 0.464167 | 0.1489231 | 0.878404 | 0.3015544 |
| set1 | BT2826 | 0.00058925 | 0.000244373 | 0.404075 | 0.1330705 | 0.0151536 | 0.01680202 |
| set1 | BT2829 | 0.0157339 | 0.004390998 | 0.372979 | 0.1246538 | 0.0526942 | 0.03920467 |
| set1 | BT2834 | 0.00369073 | 0.001203521 | 0.0268075 | 0.0138149 | 0.547801 | 0.2162796 |
| set1 | BT2839 | 0.00471879 | 0.001501249 | 0.198587 | 0.07412955 | 0.143162 | 0.0804865 |
| set1 | BT2844 | 0.00000415 | 0.00000584 | 0.167015 | 0.06427073 | 0.27943 | 0.1334789 |
| set1 | BT2846 | 0.00119157 | 0.000450027 | 0.0220964 | 0.01172731 | 0.106333 | 0.06503536 |
| set1 | BT2853 | 0.00827913 | 0.00246602 | 0.076787 | 0.03353394 | 0.173373 | 0.09298412 |
| set1 | BT2854 | 0.0000017 | 0.00000347 | 0.0679955 | 0.03025237 | 0.0138688 | 0.01587435 |
| set1 | BT2864 | 0.00324594 | 0.001073276 | 0.0650228 | 0.02912284 | 0.0868449 | 0.05588833 |
| set1 | BT2869 | 0.0320299 | 0.00827379 | 0.622992 | 0.1879479 | 0.616287 | 0.2352209 |
| set1 | BT2870 | 0.00129735 | 0.000484657 | 0.550538 | 0.170957 | 0.538947 | 0.2137613 |
| set1 | BT2878 | 0.000783099 | 0.000312934 | 0.56501 | 0.1743751 | 0.112124 | 0.06734409 |
| set1 | BT2889 | 0.0249689 | 0.006649544 | 0.242228 | 0.08757839 | 0.407211 | 0.17514 |
| set1 | BT2897 | 0.0275965 | 0.007254857 | 0.290475 | 0.1016483 | 0.398038 | 0.1723054 |
| set1 | BT2909 | 0.000425292 | 0.000186062 | 0.0435419 | 0.02090479 | 0.436406 | 0.1844229 |
| set1 | BT2911 | 0.000908676 | 0.000357083 | 0.133118 | 0.0530868 | 0.157306 | 0.08638701 |
| set1 | BT2916 | 0.000020433 | 0.0000175 | 0.286567 | 0.1004523 | 0.30988 | 0.1434949 |
| set1 | BT2917 | 0.000372777 | 0.000166947 | 0.686058 | 0.2028204 | 0.100808 | 0.06259644 |
| set1 | BT2918 | 2.02824E-05 | 0.0000174 | 0.164354 | 0.06336785 | 0.0383698 | 0.03132579 |
| set1 | BT2919 | 2.53067E-05 | 0.0000205 | 0.0181893 | 0.0099285 | 0.874837 | 0.3007471 |
| set1 | BT2921 | 0.00326341 | 0.00107792 | 0.019341 | 0.01045366 | 0.528796 | 0.2112709 |
| set1 | BT2928 | 0.00019033 | 0.0000968 | 0.0357241 | 0.01763504 | 0.0835436 | 0.05433551 |
| set1 | BT2931 | 0.0257641 | 0.006832467 | 0.348025 | 0.1180227 | 0.869562 | 0.2994948 |
| set1 | BT2935 | 0.00000184 | 0.00000357 | 0.994552 | 0.2699707 | 0.0206337 | 0.02059048 |
| set1 | BT2936 | 0.000155049 | 0.0000816 | 0.933726 | 0.2579224 | 0.0981537 | 0.06133746 |
| set1 | BT2938 | 0.0211441 | 0.005721545 | 0.0345704 | 0.01716372 | 0.384356 | 0.1681179 |
| set1 | BT2944 | 8.94294E-05 | 0.0000535 | 0.463975 | 0.1489231 | 0.0569299 | 0.04143207 |
| set1 | BT2947 | 0.000172902 | 0.0000891 | 0.268066 | 0.0951544 | 0.542384 | 0.2146605 |
| set1 | BT2950 | 0.00113596 | 0.000432695 | 0.890487 | 0.2492271 | 0.340588 | 0.1535962 |
| set1 | BT2952 | 0.000000269 | 0.00000126 | 0.343873 | 0.1169631 | 0.336603 | 0.1522625 |
| set1 | BT2953 | 0.000387592 | 0.000172527 | 0.143199 | 0.056547 | 0.013657 | 0.01569755 |
| set1 | BT2956 | 0.000889006 | 0.000350445 | 0.938055 | 0.2588223 | 0.252149 | 0.1236095 |
| set1 | BT2963 | 0.000121742 | 0.0000673 | 0.386579 | 0.1284644 | 0.0213424 | 0.02108272 |
| set1 | BT2964 | 0.00036397 | 0.000163973 | 0.104269 | 0.04331216 | 0.148521 | 0.08251961 |
| set1 | BT2969 | 0.00139813 | 0.000515241 | 0.27303 | 0.0965759 | 0.135372 | 0.07705124 |
| set1 | BT2975 | 0.000196582 | 0.0000994 | 0.981967 | 0.2675487 | 0.0536871 | 0.03968193 |
| set1 | BT2977 | 9.68945E-05 | 0.0000569 | 0.547483 | 0.1704904 | 0.0566241 | 0.0412709 |
| set1 | BT2978 | 0.0144479 | 0.004071133 | 0.372406 | 0.1245081 | 0.041404 | 0.03288838 |
| set1 | BT2979 | 1.41448E-05 | 0.0000136 | 0.570313 | 0.1756527 | 0.016645 | 0.01783468 |
| set1 | BT2980 | 0.00000009 | 0.000000783 | 0.55114 | 0.1710559 | 0.381428 | 0.1670861 |
| set1 | BT2982 | 0.00707741 | 0.002149527 | 0.807355 | 0.2306088 | 0.553196 | 0.2175903 |
| set1 | BT2983 | 0.000538417 | 0.000226771 | 0.0507359 | 0.0236421 | 0.246255 | 0.1215067 |
| set1 | BT2985 | 0.000000497 | 0.00000171 | 0.755224 | 0.2185609 | 0.00758242 | 0.01075958 |
| set1 | BT2989 | 0.000123181 | 0.000068 | 0.353452 | 0.1194053 | 0.766447 | 0.275014 |
| set1 | BT2992 | 0.00331055 | 0.001090827 | 0.147236 | 0.05787627 | 0.627091 | 0.2384764 |
| set1 | BT2994 | 0.00624522 | 0.00192145 | 0.0371919 | 0.01822554 | 0.496079 | 0.2019512 |
| set1 | BT2995 | 0.0231958 | 0.006212319 | 0.018858 | 0.01021962 | 0.361918 | 0.1608658 |
| set1 | BT3006 | 0.00363454 | 0.001186732 | 0.202192 | 0.07521955 | 0.224851 | 0.1139327 |
| set1 | BT3020 | 6.73489E-05 | 0.0000429 | 0.200892 | 0.07487428 | 0.0170533 | 0.01811007 |
| set1 | BT3027 | 0.00136149 | 0.00050488 | 0.0416819 | 0.02012377 | 0.146703 | 0.08175708 |
| set1 | BT3032 | 0.00718801 | 0.002177874 | 0.550279 | 0.1709326 | 0.0268404 | 0.02429071 |
| set1 | BT3036 | 1.11289E-05 | 0.0000114 | 0.348017 | 0.1180227 | 0.174753 | 0.09355334 |
| set1 | BT3040 | 0.000284704 | 0.000134592 | 0.801259 | 0.2292471 | 0.133696 | 0.07636378 |
| set1 | BT3041 | 5.51728E-05 | 0.0000366 | 0.743894 | 0.2162801 | 0.0263585 | 0.02403239 |
| set1 | BT3043 | 0.0219826 | 0.005925138 | 0.122593 | 0.04954374 | 0.528901 | 0.2112709 |
| set1 | BT3044 | 0.00271192 | 0.000919743 | 0.571239 | 0.1757808 | 0.174723 | 0.09355334 |
| set1 | BT3045 | 0.000339182 | 0.000154647 | 0.397752 | 0.1314541 | 0.678222 | 0.252404 |
| set1 | BT3046 | 0.0000096 | 0.0000102 | 0.421327 | 0.1378849 | 0.589641 | 0.2281353 |
| set1 | BT3053 | 0.000000453 | 0.00000159 | 0.092814 | 0.0390916 | 0.206188 | 0.1061246 |
| set1 | BT3054 | 0.00000347 | 0.00000529 | 0.109586 | 0.0451174 | 0.187385 | 0.09855465 |
| set1 | BT3058 | 0.00000265 | 0.00000437 | 0.986783 | 0.2684651 | 0.3999 | 0.1730094 |
| set1 | BT3061 | 0.000276734 | 0.000131847 | 0.181279 | 0.0688171 | 0.259984 | 0.1267892 |
| set1 | BT3071 | 0.00000791 | 0.00000903 | 0.37337 | 0.1247269 | 0.172535 | 0.09267011 |
| set1 | BT3073 | 0.000126872 | 0.0000695 | 0.286506 | 0.1004523 | 0.254536 | 0.1245876 |
| set1 | BT3077 | 2.59862E-05 | 0.0000209 | 0.467268 | 0.1497974 | 0.0397351 | 0.03217204 |
| set1 | BT3079 | 0.000000125 | 0.000000919 | 0.10673 | 0.0441825 | 0.101231 | 0.06277943 |
| set1 | BT3083 | 0.00654573 | 0.002004131 | 0.0338052 | 0.01686003 | 0.621038 | 0.2367264 |
| set1 | BT3088 | 0.00000314 | 0.00000499 | 0.262188 | 0.09325974 | 0.0127082 | 0.0149716 |
| set1 | BT3090 | 0.00558121 | 0.00174194 | 0.591608 | 0.1805997 | 0.074807 | 0.05053411 |
| set1 | BT3091 | 3.57878E-05 | 0.0000266 | 0.0925515 | 0.03900824 | 0.103656 | 0.06377573 |
| set1 | BT3092 | 0.00065809 | 0.000268508 | 0.247753 | 0.08922878 | 0.0155828 | 0.01707037 |
| set1 | BT3101 | 0.0170982 | 0.004729888 | 0.16615 | 0.06401944 | 0.275547 | 0.1321832 |
| set1 | BT3104 | 0.00736414 | 0.002223229 | 0.929033 | 0.2571805 | 0.970394 | 0.3224219 |
| set1 | BT3106 | 0.0117194 | 0.003367489 | 0.823404 | 0.2344722 | 0.767977 | 0.2754875 |
| set1 | BT3116 | 0.000113952 | 0.0000641 | 0.0628157 | 0.02826005 | 0.00790318 | 0.01098828 |
| set1 | BT3117 | 1.13782E-05 | 0.0000116 | 0.841887 | 0.2384985 | 0.0413754 | 0.03288345 |
| set1 | BT3118 | 0.00000383 | 0.00000556 | 0.994117 | 0.2699132 | 0.137304 | 0.07784902 |
| set1 | BT3119 | 0.00118489 | 0.000447951 | 0.631053 | 0.1897637 | 0.108627 | 0.06605454 |
| set1 | BT3120 | 0.00176354 | 0.000627825 | 0.79799 | 0.22842 | 0.0500908 | 0.03761963 |
| set1 | BT3123 | 0.00503761 | 0.001587994 | 0.568792 | 0.1753183 | 0.896915 | 0.3057133 |
| set1 | BT3129 | 0.00171544 | 0.000613298 | 0.0234731 | 0.01232797 | 0.0566169 | 0.0412709 |
| set1 | BT3145 | 0.00000505 | 0.00000666 | 0.0793596 | 0.03442099 | 0.720868 | 0.2632545 |
| set1 | BT3146 | 0.00000663 | 0.0000079 | 0.939043 | 0.2589765 | 0.358627 | 0.159671 |
| set1 | BT3147 | 0.00000889 | 0.00000971 | 0.185518 | 0.07016512 | 0.336453 | 0.1522625 |
| set1 | BT3168 | 0.0155598 | 0.004347219 | 0.557358 | 0.1724983 | 0.0443756 | 0.03468572 |
| set1 | BT3177 | 0.000393207 | 0.000174445 | 0.201424 | 0.07500314 | 0.00792643 | 0.0109975 |
| set1 | BT3178 | 0.000805709 | 0.000321122 | 0.0525483 | 0.02435753 | 0.100316 | 0.0623173 |
| set1 | BT3179 | 0.000967087 | 0.000376783 | 0.994949 | 0.2700177 | 0.0702667 | 0.04831186 |
| set1 | BT3182 | 0.000000573 | 0.00000189 | 0.356033 | 0.1199638 | 0.803224 | 0.2843674 |
| set1 | BT3184 | 0.0318432 | 0.008233994 | 0.446802 | 0.1444246 | 0.385657 | 0.1684861 |
| set1 | BT3186 | 3.29966E-05 | 0.0000251 | 0.15624 | 0.06072399 | 0.282623 | 0.1342904 |
| set1 | BT3187 | 2.39299E-05 | 0.0000197 | 0.426358 | 0.1392697 | 0.134861 | 0.07684978 |
| set1 | BT3192 | 0.00338159 | 0.001110849 | 0.019699 | 0.01060874 | 0.643185 | 0.2425637 |
| set1 | BT3208 | 0.026323 | 0.006966039 | 0.0306079 | 0.01550223 | 0.361248 | 0.1606431 |
| set1 | BT3210 | 3.52545E-05 | 0.0000264 | 0.757161 | 0.2190689 | 0.030534 | 0.02670773 |
| set1 | BT3211 | 0.000319303 | 0.000147179 | 0.504032 | 0.1589675 | 0.123704 | 0.07211575 |
| set1 | BT3212 | 0.000170515 | 0.000088 | 0.983929 | 0.2679904 | 0.0166759 | 0.0178404 |
| set1 | BT3214 | 1.94776E-05 | 0.0000169 | 0.932243 | 0.2577485 | 0.0826449 | 0.0540865 |
| set1 | BT3226 | 0.00961923 | 0.002821871 | 0.535802 | 0.1672409 | 0.0269897 | 0.02436574 |
| set1 | BT3230 | 0.000000222 | 0.00000111 | 0.738075 | 0.2149368 | 0.0643324 | 0.04542316 |
| set1 | BT3233 | 0.00000792 | 0.00000903 | 0.0876903 | 0.03738988 | 0.947109 | 0.3175604 |
| set1 | BT3238 | 0.0171468 | 0.004740199 | 0.253126 | 0.09082803 | 0.94503 | 0.3172041 |
| set1 | BT3240 | 8.07999E-05 | 0.0000496 | 0.785149 | 0.2256533 | 0.0228376 | 0.02189664 |
| set1 | BT3242 | 7.03032E-05 | 0.0000443 | 0.3476 | 0.117978 | 0.117433 | 0.06953697 |
| set1 | BT3248 | 0.000280155 | 0.000132897 | 0.0666768 | 0.02976432 | 0.150366 | 0.0832925 |
| set1 | BT3257 | 4.09215E-05 | 0.0000294 | 0.514733 | 0.1618776 | 0.0104618 | 0.01322746 |
| set1 | BT3258 | 0.000464664 | 0.000200163 | 0.257614 | 0.09206654 | 0.221133 | 0.1123591 |
| set1 | BT3261 | 0.0325049 | 0.008385042 | 0.273507 | 0.0966597 | 0.0105394 | 0.01325744 |
| set1 | BT3263 | 0.00000115 | 0.00000276 | 0.289949 | 0.10152 | 0.0678778 | 0.04706619 |
| set1 | BT3268 | 7.38134E-05 | 0.0000462 | 0.849789 | 0.2403239 | 0.0299995 | 0.02643268 |
| set1 | BT3272 | 0.00060914 | 0.000251711 | 0.0356546 | 0.01762233 | 0.0845106 | 0.0548187 |
| set1 | BT3273 | 0.000297483 | 0.000139242 | 0.0317844 | 0.01599749 | 0.00842161 | 0.01135192 |
| set1 | BT3275 | 0.00000376 | 0.00000551 | 0.0764555 | 0.03342542 | 0.0095304 | 0.01242219 |
| set1 | BT3284 | 0.00102393 | 0.000395596 | 0.207946 | 0.07695724 | 0.116798 | 0.06927273 |
| set1 | BT3287 | 0.00136353 | 0.000505141 | 0.56864 | 0.1753183 | 0.0415802 | 0.03299263 |
| set1 | BT3288 | 0.00848945 | 0.002518753 | 0.0619779 | 0.02793516 | 0.990296 | 0.3269623 |
| set1 | BT3293 | 0.00000271 | 0.00000442 | 0.630012 | 0.1896395 | 0.949006 | 0.3178469 |
| set1 | BT3294 | 0.000877173 | 0.000346683 | 0.0753853 | 0.03304125 | 0.109152 | 0.06626398 |
| set1 | BT3300 | 0.00788355 | 0.002364008 | 0.238244 | 0.0864225 | 0.63924 | 0.2414079 |
| set1 | BT3301 | 0.000133667 | 0.0000725 | 0.630378 | 0.1896789 | 0.411981 | 0.1767255 |
| set1 | BT3302 | 0.00000191 | 0.00000366 | 0.810998 | 0.2314852 | 0.131123 | 0.07530172 |
| set1 | BT3305 | 0.000158932 | 0.0000831 | 0.283688 | 0.09967452 | 0.28197 | 0.1340814 |
| set1 | BT3318 | 0.000000224 | 0.00000111 | 0.0511192 | 0.02376858 | 0.0338487 | 0.02865464 |
| set1 | BT3323 | 0.000190235 | 0.0000968 | 0.0619482 | 0.02793219 | 0.255824 | 0.1250513 |
| set1 | BT3329 | 0.000302112 | 0.000140799 | 0.836555 | 0.2373777 | 0.062533 | 0.04451668 |
| set1 | BT3332 | 0.0269541 | 0.007105722 | 0.0474376 | 0.02240996 | 0.11455 | 0.06829819 |
| set1 | BT3336 | 0.00596317 | 0.00184744 | 0.363237 | 0.1217459 | 0.9303 | 0.3138264 |
| set1 | BT3337 | 8.22416E-05 | 0.0000502 | 0.345053 | 0.1172782 | 0.366231 | 0.1623738 |
| set1 | BT3338 | 1.77284E-05 | 0.0000159 | 0.704869 | 0.2071143 | 0.100057 | 0.06218272 |
| set1 | BT3345 | 0.034382 | 0.008815183 | 0.464171 | 0.1489231 | 0.782136 | 0.2790067 |
| set1 | BT3348 | 0.00180744 | 0.000641041 | 0.422855 | 0.138208 | 0.109726 | 0.06642016 |
| set1 | BT3349 | 0.0139199 | 0.00393554 | 0.63627 | 0.190668 | 0.0716644 | 0.04895186 |
| set1 | BT3351 | 0.00000256 | 0.0000043 | 0.295656 | 0.1029215 | 0.0192483 | 0.01956988 |
| set1 | BT3353 | 0.00115558 | 0.000438184 | 0.115787 | 0.04737972 | 0.0581597 | 0.04207675 |
| set1 | BT3358 | 0.000200778 | 0.000101038 | 0.0908982 | 0.03848598 | 0.0644058 | 0.04544215 |
| set1 | BT3364 | 0.00229586 | 0.000792361 | 0.292792 | 0.1022782 | 0.0684027 | 0.04738538 |
| set1 | BT3367 | 0.0254545 | 0.006757466 | 0.480814 | 0.1532452 | 0.113189 | 0.06770672 |
| set1 | BT3373 | 0.00829304 | 0.002469191 | 0.421962 | 0.137983 | 0.0568717 | 0.04141025 |
| set1 | BT3375 | 0.00392921 | 0.00127306 | 0.737196 | 0.214774 | 0.100017 | 0.06218272 |
| set1 | BT3377 | 0.00722586 | 0.002188466 | 0.399245 | 0.1319114 | 0.992542 | 0.3274829 |
| set1 | BT3385 | 1.45738E-05 | 0.0000138 | 0.353723 | 0.119452 | 0.111452 | 0.0670777 |
| set1 | BT3387 | 0.00292805 | 0.000982762 | 0.0498022 | 0.0233178 | 0.0241157 | 0.02259087 |
| set1 | BT3404 | 0.000311202 | 0.000144147 | 0.587335 | 0.1794767 | 0.299786 | 0.1401295 |
| set1 | BT3405 | 0.0320993 | 0.008288888 | 0.0565784 | 0.02594681 | 0.232498 | 0.1166 |
| set1 | BT3409 | 9.73593E-05 | 0.000057 | 0.837046 | 0.2374055 | 0.0216228 | 0.02128168 |
| set1 | BT3411 | 0.012025 | 0.003447448 | 0.42735 | 0.1395183 | 0.363612 | 0.1614498 |
| set1 | BT3415 | 0.000385658 | 0.000172003 | 0.322674 | 0.1107628 | 0.0817217 | 0.05367376 |
| set1 | BT3420 | 5.38E-08 | 0.000000626 | 0.141077 | 0.05583683 | 0.00896243 | 0.01188448 |
| set1 | BT3434 | 0.00440841 | 0.001412 | 0.0318521 | 0.01601822 | 0.796906 | 0.2828321 |
| set1 | BT3443 | 0.00000142 | 0.00000316 | 0.672201 | 0.1995881 | 0.0380778 | 0.03115671 |
| set1 | BT3444 | 0.00000113 | 0.00000273 | 0.0767074 | 0.03351234 | 0.188976 | 0.0992137 |
| set1 | BT3446 | 0.000613048 | 0.000252719 | 0.136527 | 0.05426714 | 0.0714871 | 0.04887528 |
| set1 | BT3448 | 5.51562E-05 | 0.0000366 | 0.0208786 | 0.01117426 | 0.0251501 | 0.02325705 |
| set1 | BT3452 | 0.000000446 | 0.00000158 | 0.153607 | 0.05989336 | 0.18705 | 0.09846163 |
| set1 | BT3464 | 0.000000932 | 0.0000025 | 0.548217 | 0.170631 | 0.00873026 | 0.01165029 |
| set1 | BT3466 | 0.000281234 | 0.000133284 | 0.0214499 | 0.01143948 | 0.0288495 | 0.0256659 |
| set1 | BT3469 | 0.0000016 | 0.00000337 | 0.287097 | 0.1006089 | 0.0598409 | 0.04303851 |
| set1 | BT3470 | 0.000000576 | 0.00000189 | 0.795766 | 0.2279995 | 0.0148473 | 0.01660736 |
| set1 | BT3471 | 0.000000654 | 0.00000202 | 0.089656 | 0.03805341 | 0.00748332 | 0.01066734 |
| set1 | BT3476 | 0.00505699 | 0.00159344 | 0.0542165 | 0.02496388 | 0.053379 | 0.0395445 |
| set1 | BT3484 | 0.00568979 | 0.001772087 | 0.522368 | 0.1638096 | 0.230801 | 0.1162255 |
| set1 | BT3486 | 0.00938745 | 0.002759224 | 0.272019 | 0.09627467 | 0.260526 | 0.1269156 |
| set1 | BT3488 | 0.011819 | 0.003390959 | 0.998927 | 0.2707324 | 0.676113 | 0.2518344 |
| set1 | BT3497 | 0.000567951 | 0.000237345 | 0.83815 | 0.2376628 | 0.0355219 | 0.02956159 |
| set1 | BT3498 | 0.000737374 | 0.000296226 | 0.163316 | 0.06302799 | 0.182537 | 0.09680258 |
| set1 | BT3500 | 5.32204E-05 | 0.0000356 | 0.190349 | 0.0717417 | 0.022171 | 0.02152463 |
| set1 | BT3501 | 0.000229918 | 0.000113071 | 0.0757295 | 0.03314401 | 0.102271 | 0.0632907 |
| set1 | BT3503 | 0.0200293 | 0.005441282 | 0.0815118 | 0.03517764 | 0.0320441 | 0.02770209 |
| set1 | BT3518 | 0.000000666 | 0.00000202 | 0.0213875 | 0.01141124 | 0.277079 | 0.132788 |
| set1 | BT3519 | 2.89038E-05 | 0.0000227 | 0.0412112 | 0.01993636 | 0.620399 | 0.2366058 |
| set1 | BT3522 | 0.000292635 | 0.000137483 | 0.324707 | 0.1112393 | 0.0803436 | 0.05302963 |
| set1 | BT3524 | 0.0142901 | 0.00403117 | 0.436248 | 0.1417503 | 0.238239 | 0.1184673 |
| set1 | BT3529 | 0.00000837 | 0.00000939 | 0.115064 | 0.04714776 | 0.0731981 | 0.04974486 |
| set1 | BT3533 | 0.0005334 | 0.00022541 | 0.0280548 | 0.01438404 | 0.038761 | 0.03153741 |
| set1 | BT3540 | 0.00000329 | 0.00000509 | 0.403748 | 0.1330353 | 0.0169414 | 0.01804029 |
| set1 | BT3541 | 0.0129338 | 0.003687043 | 0.550318 | 0.1709326 | 0.0301283 | 0.02650587 |
| set1 | BT3544 | 7.18093E-05 | 0.0000451 | 0.472332 | 0.1509717 | 0.0101441 | 0.01297101 |
| set1 | BT3545 | 1.21746E-05 | 0.0000122 | 0.0246319 | 0.0128583 | 0.281137 | 0.1339021 |
| set1 | BT3546 | 2.98803E-05 | 0.0000234 | 0.411954 | 0.1352963 | 0.0384175 | 0.03134729 |
| set1 | BT3548 | 0.000744226 | 0.000298504 | 0.12034 | 0.04879649 | 0.105794 | 0.06478808 |
| set1 | BT3559 | 0.00577415 | 0.00179402 | 0.0615203 | 0.02777035 | 0.03089 | 0.02696782 |
| set1 | BT3560 | 0.00000021 | 0.00000108 | 0.0955744 | 0.04007259 | 0.947439 | 0.3175699 |
| set1 | BT3561 | 0.00000225 | 0.000004 | 0.730947 | 0.2132209 | 0.799935 | 0.2836328 |
| set1 | BT3562 | 0.00000125 | 0.00000291 | 0.122576 | 0.04954374 | 0.729063 | 0.2652964 |
| set1 | BT3568 | 4.05441E-05 | 0.0000293 | 0.0462458 | 0.02194995 | 0.0400603 | 0.03223974 |
| set1 | BT3577 | 0.000581966 | 0.000241679 | 0.158136 | 0.06132548 | 0.0146322 | 0.0164732 |
| set1 | BT3581 | 0.000216927 | 0.000107521 | 0.52799 | 0.1651439 | 0.487779 | 0.2000056 |
| set1 | BT3583 | 0.00050508 | 0.000214882 | 0.394446 | 0.1304686 | 0.0336618 | 0.02857232 |
| set1 | BT3587 | 1.16875E-05 | 0.0000118 | 0.075051 | 0.03293058 | 0.0164443 | 0.01773798 |
| set1 | BT3590 | 0.0183001 | 0.005027457 | 0.0841603 | 0.0361142 | 0.286209 | 0.1354826 |
| set1 | BT3598 | 0.00035327 | 0.000160072 | 0.451853 | 0.1458621 | 0.0510666 | 0.03824573 |
| set1 | BT3599 | 0.000289618 | 0.000136319 | 0.0592354 | 0.02687957 | 0.202202 | 0.1044025 |
| set1 | BT3600 | 0.00481814 | 0.001530284 | 0.936508 | 0.2585135 | 0.143459 | 0.08062259 |
| set1 | BT3602 | 0.000516523 | 0.000219012 | 0.831353 | 0.2363458 | 0.00768914 | 0.01083352 |
| set1 | BT3604 | 0.0382562 | 0.009716327 | 0.166997 | 0.06427073 | 0.781959 | 0.2790067 |
| set1 | BT3605 | 0.00167025 | 0.000599407 | 0.0818772 | 0.0352975 | 0.726043 | 0.2645741 |
| set1 | BT3609 | 0.0142568 | 0.004023276 | 0.429217 | 0.1400143 | 0.212655 | 0.1088425 |
| set1 | BT3611 | 0.000561334 | 0.000235115 | 0.149054 | 0.05847672 | 0.012944 | 0.01517158 |
| set1 | BT3618 | 0.000175176 | 0.0000902 | 0.872377 | 0.2452381 | 0.803687 | 0.2843674 |
| set1 | BT3619 | 0.00503757 | 0.001587994 | 0.813873 | 0.2321772 | 0.490444 | 0.2004265 |
| set1 | BT3623 | 0.000110323 | 0.0000626 | 0.0871011 | 0.03721747 | 0.381271 | 0.1670672 |
| set1 | BT3630 | 0.000000335 | 0.00000133 | 0.185162 | 0.07007096 | 0.0222072 | 0.02152745 |
| set1 | BT3637 | 6.53E-08 | 0.000000654 | 0.0903956 | 0.03831347 | 0.00899623 | 0.01190779 |
| set1 | BT3645 | 0.0000068 | 0.00000804 | 0.21609 | 0.07965395 | 0.832312 | 0.2910354 |
| set1 | BT3646 | 5.30602E-05 | 0.0000356 | 0.270108 | 0.09568242 | 0.0869443 | 0.05588864 |
| set1 | BT3648 | 0.00000373 | 0.00000551 | 0.343682 | 0.1169631 | 0.131221 | 0.07530172 |
| set1 | BT3649 | 0.00000257 | 0.0000043 | 0.966553 | 0.2645701 | 0.146671 | 0.08175708 |
| set1 | BT3652 | 0.000000424 | 0.00000154 | 0.0310774 | 0.01569396 | 0.897509 | 0.3058195 |
| set1 | BT3653 | 0.00000403 | 0.00000575 | 0.0378239 | 0.01849773 | 0.101961 | 0.063149 |
| set1 | BT3655 | 1.83362E-05 | 0.0000163 | 0.0289986 | 0.01478635 | 0.0231115 | 0.02203204 |
| set1 | BT3657 | 0.00000167 | 0.00000343 | 0.309533 | 0.1071346 | 0.644837 | 0.2428967 |
| set1 | BT3659 | 0.000159704 | 0.0000834 | 0.0682689 | 0.0303371 | 0.0844124 | 0.05477921 |
| set1 | BT3661 | 0.00254 | 0.000868306 | 0.30614 | 0.1061125 | 0.010398 | 0.01319224 |
| set1 | BT3680 | 8.67226E-05 | 0.0000523 | 0.0649416 | 0.02909726 | 0.0111766 | 0.01380239 |
| set1 | BT3685 | 0.00309067 | 0.001028447 | 0.0290956 | 0.0148233 | 0.0834916 | 0.05433551 |
| set1 | BT3687 | 0.00037016 | 0.000166168 | 0.0409649 | 0.01983309 | 0.0153216 | 0.01691043 |
| set1 | BT3689 | 0.0028783 | 0.000970368 | 0.791339 | 0.2268802 | 0.817378 | 0.2874568 |
| set1 | BT3692 | 0.00602054 | 0.001861404 | 0.990357 | 0.2692036 | 0.0170391 | 0.01811007 |
| set1 | BT3708 | 0.00000416 | 0.00000584 | 0.0569335 | 0.02605032 | 0.0566603 | 0.04127679 |
| set1 | BT3709 | 0.00187481 | 0.000660294 | 0.960688 | 0.2634423 | 0.0134236 | 0.0155523 |
| set1 | BT3711 | 0.0104823 | 0.003042044 | 0.873397 | 0.2453535 | 0.124611 | 0.07258685 |
| set1 | BT3712 | 2.67946E-05 | 0.0000214 | 0.519833 | 0.1631417 | 0.231886 | 0.1164921 |
| set1 | BT3713 | 0.000977447 | 0.000380552 | 0.567089 | 0.1749273 | 0.0788924 | 0.05233062 |
| set1 | BT3723 | 3.61787E-05 | 0.0000268 | 0.0872515 | 0.03725538 | 0.0496146 | 0.03740638 |
| set1 | BT3725 | 1.76155E-05 | 0.0000159 | 0.0407338 | 0.01974495 | 0.0877135 | 0.05622547 |
| set1 | BT3726 | 0.000864557 | 0.000343131 | 0.0346184 | 0.01718049 | 0.0699763 | 0.04817142 |
| set1 | BT3730 | 0.000932073 | 0.000364951 | 0.470421 | 0.1506482 | 0.0735048 | 0.04986095 |
| set1 | BT3733 | 0.00000452 | 0.00000615 | 0.879192 | 0.2467519 | 0.140315 | 0.07928063 |
| set1 | BT3737 | 0.0160608 | 0.00447069 | 0.0357112 | 0.01763504 | 0.44594 | 0.1873508 |
| set1 | BT3740 | 0.00831225 | 0.002472965 | 0.292908 | 0.1022892 | 0.109543 | 0.06640243 |
| set1 | BT3743 | 5.10281E-05 | 0.0000346 | 0.148075 | 0.05811151 | 0.0451545 | 0.03498244 |
| set1 | BT3745 | 0.017162 | 0.004742337 | 0.171329 | 0.06565892 | 0.049408 | 0.03733221 |
| set1 | BT3757 | 0.00107387 | 0.000412156 | 0.0472847 | 0.02235521 | 0.875332 | 0.3008468 |
| set1 | BT3763 | 0.000241353 | 0.000117852 | 0.0832777 | 0.03579905 | 0.656172 | 0.24603 |
| set1 | BT3765 | 1.86496E-05 | 0.0000165 | 0.102227 | 0.04250779 | 0.24047 | 0.119173 |
| set1 | BT3766 | 0.000019948 | 0.0000172 | 0.502646 | 0.1586132 | 0.207708 | 0.1067507 |
| set1 | BT3768 | 0.00000656 | 0.00000787 | 0.0512916 | 0.02383161 | 0.0365536 | 0.0301787 |
| set1 | BT3780 | 0.000191172 | 0.0000972 | 0.159919 | 0.06187515 | 0.131201 | 0.07530172 |
| set1 | BT3781 | 0.00000514 | 0.00000669 | 0.0263772 | 0.01362221 | 0.0212895 | 0.021073 |
| set1 | BT3784 | 0.000445535 | 0.000192847 | 0.451151 | 0.1457523 | 0.67798 | 0.2524017 |
| set1 | BT3786 | 6.83664E-05 | 0.0000433 | 0.218365 | 0.08024714 | 0.011156 | 0.01379621 |
| set1 | BT3790 | 0.00115464 | 0.000438047 | 0.954297 | 0.2622255 | 0.0166868 | 0.0178404 |
| set1 | BT3792 | 4.15774E-05 | 0.0000298 | 0.113223 | 0.04640915 | 0.917195 | 0.3105809 |
| set1 | BT3806 | 0.0072938 | 0.002206394 | 0.695567 | 0.2049794 | 0.952659 | 0.3186207 |
| set1 | BT3808 | 0.00000295 | 0.00000472 | 0.0181172 | 0.00991603 | 0.136576 | 0.07761353 |
| set1 | BT3811 | 0.000101046 | 0.0000587 | 0.258349 | 0.09227462 | 0.997475 | 0.3288149 |
| set1 | BT3814 | 0.00304452 | 0.001014655 | 0.109198 | 0.04500356 | 0.0734699 | 0.04986032 |
| set1 | BT3815 | 0.000878012 | 0.000346834 | 0.0184258 | 0.01002588 | 0.334885 | 0.1517703 |
| set1 | BT3817 | 0.00371918 | 0.001210712 | 0.0344488 | 0.0171143 | 0.161049 | 0.0880358 |
| set1 | BT3832 | 5.87687E-05 | 0.0000384 | 0.254919 | 0.09131954 | 0.0164181 | 0.01773395 |
| set1 | BT3833 | 0.0179435 | 0.004936659 | 0.0679417 | 0.03023957 | 0.01173 | 0.01423032 |
| set1 | BT3835 | 0.000895046 | 0.000352276 | 0.991371 | 0.2693493 | 0.0176543 | 0.0184449 |
| set1 | BT3838 | 0.000000116 | 0.000000902 | 0.0717247 | 0.03170138 | 0.00752535 | 0.01069371 |
| set1 | BT3842 | 0.00000415 | 0.00000584 | 0.159054 | 0.0616481 | 0.0449507 | 0.03491216 |
| set1 | BT3843 | 5.90758E-05 | 0.0000386 | 0.713566 | 0.2089581 | 0.0254958 | 0.02346443 |
| set1 | BT3844 | 0.00164035 | 0.000591058 | 0.124212 | 0.05009747 | 0.859961 | 0.2975143 |
| set1 | BT3848 | 0.00220838 | 0.000764261 | 0.031851 | 0.01601822 | 0.0224758 | 0.02170985 |
| set1 | BT3850 | 1.11981E-05 | 0.0000114 | 0.112485 | 0.04618501 | 0.137357 | 0.07784902 |
| set1 | BT3851 | 1.24565E-05 | 0.0000124 | 0.0397125 | 0.01927309 | 0.530091 | 0.211506 |
| set1 | BT3852 | 0.000000185 | 0.00000103 | 0.230909 | 0.08416614 | 0.0119136 | 0.01439358 |
| set1 | BT3863 | 3.20686E-05 | 0.0000245 | 0.737338 | 0.214774 | 0.0104575 | 0.01322746 |
| set1 | BT3864 | 0.00788669 | 0.002364013 | 0.0240946 | 0.01261052 | 0.33977 | 0.1533215 |
| set1 | BT3871 | 2.29889E-05 | 0.0000191 | 0.607288 | 0.184415 | 0.0177327 | 0.01846111 |
| set1 | BT3873 | 0.000104638 | 0.0000603 | 0.108606 | 0.04480555 | 0.0329986 | 0.02815223 |
| set1 | BT3875 | 0.0038527 | 0.001250412 | 0.216714 | 0.0798109 | 0.0758807 | 0.05097791 |
| set1 | BT3878 | 0.00014568 | 0.0000772 | 0.291404 | 0.1018523 | 0.106046 | 0.06491532 |
| set1 | BT3884 | 0.00477685 | 0.001517806 | 0.0186777 | 0.0101401 | 0.483869 | 0.1990697 |
| set1 | BT3887 | 0.00387969 | 0.001258093 | 0.0805115 | 0.03485803 | 0.0495728 | 0.03739407 |
| set1 | BT3889 | 0.000280241 | 0.000132897 | 0.279306 | 0.0982492 | 0.0140659 | 0.01598112 |
| set1 | BT3892 | 1.80244E-05 | 0.0000162 | 0.548559 | 0.1706744 | 0.123539 | 0.07207681 |
| set1 | BT3893 | 2.59109E-05 | 0.0000209 | 0.995969 | 0.2702338 | 0.0173983 | 0.01829463 |
| set1 | BT3894 | 0.000120323 | 0.0000668 | 0.914335 | 0.2543692 | 0.236912 | 0.1180875 |
| set1 | BT3898 | 0.00134662 | 0.000500346 | 0.778035 | 0.2239313 | 0.0100597 | 0.01290806 |
| set1 | BT3900 | 0.0120368 | 0.003449524 | 0.296773 | 0.1032211 | 0.200737 | 0.1038286 |
| set1 | BT3901 | 0.017798 | 0.004898409 | 0.560224 | 0.1732078 | 0.187755 | 0.09871388 |
| set1 | BT3902 | 0.000372527 | 0.000166947 | 0.0243116 | 0.01271308 | 0.186402 | 0.09821359 |
| set1 | BT3910 | 0.000612741 | 0.000252719 | 0.213449 | 0.07880067 | 0.403847 | 0.1741012 |
| set1 | BT3912 | 1.25608E-05 | 0.0000124 | 0.905777 | 0.2528619 | 0.883176 | 0.3026923 |
| set1 | BT3917 | 0.034402 | 0.008817324 | 0.491685 | 0.1558874 | 0.292448 | 0.1375799 |
| set1 | BT3918 | 0.000284206 | 0.00013444 | 0.116106 | 0.04745332 | 0.0163443 | 0.01768026 |
| set1 | BT3919 | 0.000000099 | 0.000000833 | 0.0552654 | 0.02539176 | 0.0428581 | 0.0337331 |
| set1 | BT3920 | 1.73397E-05 | 0.0000157 | 0.923607 | 0.2561229 | 0.0251691 | 0.02325705 |
| set1 | BT3925 | 0.00118433 | 0.000447951 | 0.690486 | 0.2040544 | 0.0302175 | 0.02654509 |
| set1 | BT3932 | 0.00056854 | 0.000237345 | 0.716196 | 0.2095251 | 0.0490819 | 0.03721501 |
| set1 | BT3934 | 0.000467795 | 0.000201168 | 0.136187 | 0.05418747 | 0.1501 | 0.08317654 |
| set1 | BT3937 | 0.00000973 | 0.0000103 | 0.0896213 | 0.03805205 | 0.0179667 | 0.01865181 |
| set1 | BT3938 | 0.0346196 | 0.00886709 | 0.0251018 | 0.0130416 | 0.17587 | 0.09400453 |
| set1 | BT3953 | 0.000165786 | 0.000086 | 0.0828308 | 0.0356323 | 0.167672 | 0.0907555 |
| set1 | BT3960 | 0.00000314 | 0.00000499 | 0.0969483 | 0.04060636 | 0.0357168 | 0.02966633 |
| set1 | BT3961 | 0.0017582 | 0.000626219 | 0.911572 | 0.2540101 | 0.103133 | 0.06362504 |
| set1 | BT3981 | 0.00165741 | 0.000595646 | 0.480989 | 0.1532606 | 0.518333 | 0.2082866 |
| set1 | BT3984 | 0.000000396 | 0.00000148 | 0.0434667 | 0.02088529 | 0.0170853 | 0.01812127 |
| set1 | BT3985 | 6.95E-09 | 0.000000225 | 0.191525 | 0.07202761 | 0.00800498 | 0.01105505 |
| set1 | BT3987 | 2.37E-08 | 0.000000385 | 0.67225 | 0.1995881 | 0.0436103 | 0.03419679 |
| set1 | BT3988 | 1.15E-08 | 0.000000264 | 0.532431 | 0.1663606 | 0.0407462 | 0.03257741 |
| set1 | BT3993 | 5.01636E-05 | 0.0000342 | 0.0895856 | 0.03805026 | 0.0486381 | 0.03693575 |
| set1 | BT3994 | 0.00290487 | 0.000976715 | 0.996967 | 0.2703225 | 0.231113 | 0.1163028 |
| set1 | BT3995 | 0.00275888 | 0.000933434 | 0.75219 | 0.2178394 | 0.0351752 | 0.02943805 |
| set1 | BT3999 | 0.000135368 | 0.0000732 | 0.711726 | 0.2085204 | 0.161013 | 0.0880358 |
| set1 | BT4000 | 0.000794317 | 0.000316915 | 0.0220587 | 0.01171245 | 0.030327 | 0.02659354 |
| set1 | BT4001 | 0.00000794 | 0.00000904 | 0.184213 | 0.06975553 | 0.0243538 | 0.02278484 |
| set1 | BT4004 | 1.47239E-05 | 0.0000139 | 0.0221253 | 0.01173749 | 0.124766 | 0.0726195 |
| set1 | BT4009 | 1.67771E-05 | 0.0000152 | 0.0246643 | 0.01286965 | 0.0134821 | 0.01558317 |
| set1 | BT4020 | 0.00136563 | 0.000505672 | 0.969846 | 0.2651111 | 0.0245092 | 0.02287192 |
| set1 | BT4023 | 0.000750726 | 0.000300792 | 0.413392 | 0.1356948 | 0.702572 | 0.2584111 |
| set1 | BT4025 | 0.0035311 | 0.001155949 | 0.918099 | 0.2549225 | 0.369601 | 0.163417 |
| set1 | BT4029 | 0.00000511 | 0.00000668 | 0.0215736 | 0.0114953 | 0.0785994 | 0.05221342 |
| set1 | BT4034 | 1.79E-08 | 0.00000033 | 0.48437 | 0.1541346 | 0.014139 | 0.01605175 |
| set1 | BT4041 | 0.0000015 | 0.00000325 | 0.900477 | 0.2517353 | 0.023153 | 0.02204913 |
| set1 | BT4049 | 0.000394746 | 0.00017482 | 0.241534 | 0.08737978 | 0.102943 | 0.06357257 |
| set1 | BT4054 | 0.00182528 | 0.000645855 | 0.35619 | 0.1199832 | 0.233385 | 0.1168453 |
| set1 | BT4055 | 0.00245222 | 0.000839435 | 0.222096 | 0.08139591 | 0.11281 | 0.06756261 |
| set1 | BT4061 | 0.00200628 | 0.000702023 | 0.0582606 | 0.02653692 | 0.298013 | 0.1395403 |
| set1 | BT4063 | 0.00122388 | 0.000460163 | 0.509724 | 0.1605113 | 0.460207 | 0.1920258 |
| set1 | BT4064 | 0.0242648 | 0.006473422 | 0.0656492 | 0.02934899 | 0.507821 | 0.2055334 |
| set1 | BT4066 | 0.00176877 | 0.000629391 | 0.019397 | 0.01047412 | 0.905676 | 0.3080039 |
| set1 | BT4069 | 0.0250092 | 0.006653839 | 0.0209043 | 0.01118306 | 0.00938187 | 0.01229627 |
| set1 | BT4076 | 3.52206E-05 | 0.0000264 | 0.0306415 | 0.01550625 | 0.0257182 | 0.02358041 |
| set1 | BT4082 | 0.00000854 | 0.00000946 | 0.726212 | 0.2119932 | 0.0126613 | 0.01494032 |
| set1 | BT4083 | 2.45858E-05 | 0.0000201 | 0.0530378 | 0.02452578 | 0.191948 | 0.1004886 |
| set1 | BT4085 | 0.000140309 | 0.000075 | 0.0585869 | 0.02662549 | 0.142601 | 0.0802941 |
| set1 | BT4087 | 0.00289402 | 0.000974365 | 0.865447 | 0.2437438 | 0.0290129 | 0.02574885 |
| set1 | BT4091 | 0.0106448 | 0.003082112 | 0.934806 | 0.2581617 | 0.065796 | 0.04619024 |
| set1 | BT4092 | 4.08093E-05 | 0.0000294 | 0.145759 | 0.05741919 | 0.0148773 | 0.01660736 |
| set1 | BT4104 | 0.0216426 | 0.005841817 | 0.221668 | 0.08126371 | 0.130364 | 0.07501228 |
| set1 | BT4106 | 0.0000039 | 0.00000563 | 0.0527854 | 0.02444867 | 0.554005 | 0.2177918 |
| set1 | BT4107 | 0.00000826 | 0.00000929 | 0.0256324 | 0.01329814 | 0.777527 | 0.2782694 |
| set1 | BT4117 | 0.0214165 | 0.005786979 | 0.0581283 | 0.02650664 | 0.899184 | 0.3063192 |
| set1 | BT4122 | 0.00000599 | 0.00000742 | 0.153257 | 0.05977618 | 0.00838124 | 0.01133505 |
| set1 | BT4123 | 0.000183619 | 0.0000938 | 0.0571445 | 0.02612707 | 0.00851062 | 0.01142992 |
| set1 | BT4126 | 0.000553326 | 0.000232404 | 0.531174 | 0.1660538 | 0.849597 | 0.2953208 |
| set1 | BT4127 | 8.38852E-05 | 0.000051 | 0.166607 | 0.0641546 | 0.76518 | 0.2748953 |
| set1 | BT4128 | 0.00093001 | 0.000364332 | 0.461856 | 0.1484169 | 0.289156 | 0.1364815 |
| set1 | BT4143 | 4.32756E-05 | 0.0000306 | 0.429846 | 0.1401816 | 0.720735 | 0.2632545 |
| set1 | BT4147 | 0.000563039 | 0.000235698 | 0.136387 | 0.05424365 | 0.023541 | 0.02232318 |
| set1 | BT4154 | 5.11512E-05 | 0.0000346 | 0.87651 | 0.2460564 | 0.0793132 | 0.05253852 |
| set1 | BT4163 | 0.00000804 | 0.00000913 | 0.268661 | 0.09527597 | 0.0387016 | 0.03150905 |
| set1 | BT4165 | 0.0000024 | 0.00000414 | 0.38402 | 0.1277546 | 0.0606869 | 0.04347655 |
| set1 | BT4166 | 0.00000481 | 0.00000644 | 0.202606 | 0.07528938 | 0.0405446 | 0.03250475 |
| set1 | BT4167 | 6.01356E-05 | 0.0000391 | 0.80995 | 0.2312407 | 0.235766 | 0.1177562 |
| set1 | BT4168 | 0.00000403 | 0.00000575 | 0.239858 | 0.08690358 | 0.0153747 | 0.01693609 |
| set1 | BT4169 | 3.60838E-05 | 0.0000268 | 0.522824 | 0.1638713 | 0.183754 | 0.09715436 |
| set1 | BT4170 | 0.000849763 | 0.000337613 | 0.534578 | 0.1669073 | 0.700388 | 0.2578663 |
| set1 | BT4171 | 0.00000959 | 0.0000102 | 0.030791 | 0.01556886 | 0.0161981 | 0.01756245 |
| set1 | BT4173 | 0.0014287 | 0.000525172 | 0.241213 | 0.08728979 | 0.292514 | 0.1375799 |
| set1 | BT4174 | 0.00273763 | 0.000927073 | 0.362144 | 0.1214807 | 0.232159 | 0.1165307 |
| set1 | BT4186 | 0.00000228 | 0.00000402 | 0.126594 | 0.05083752 | 0.00869198 | 0.01162033 |
| set1 | BT4189 | 0.00376339 | 0.001222475 | 0.368222 | 0.1232457 | 0.194797 | 0.1014259 |
| set1 | BT4190 | 0.0185452 | 0.005080041 | 0.172271 | 0.06595715 | 0.684638 | 0.2540428 |
| set1 | BT4192 | 0.0311915 | 0.008082045 | 0.133191 | 0.05309839 | 0.227455 | 0.1148557 |
| set1 | BT4203 | 0.00000116 | 0.00000277 | 0.0416138 | 0.02009892 | 0.0165666 | 0.01776364 |
| set1 | BT4204 | 0.000565927 | 0.000236646 | 0.778045 | 0.2239313 | 0.130832 | 0.07516665 |
| set1 | BT4205 | 5.59211E-05 | 0.0000369 | 0.275746 | 0.09730864 | 0.00785296 | 0.0109473 |
| set1 | BT4208 | 0.000225562 | 0.000111178 | 0.452407 | 0.1459614 | 0.0129111 | 0.01515629 |
| set1 | BT4209 | 8.40564E-05 | 0.0000511 | 0.56488 | 0.1743751 | 0.00720464 | 0.01040937 |
| set1 | BT4215 | 0.000127198 | 0.0000697 | 0.118215 | 0.04813318 | 0.13326 | 0.07619803 |
| set1 | BT4216 | 0.000101188 | 0.0000587 | 0.29376 | 0.1024001 | 0.234128 | 0.1170974 |
| set1 | BT4217 | 6.50428E-05 | 0.0000416 | 0.0893672 | 0.03799758 | 0.0626238 | 0.04454153 |
| set1 | BT4218 | 0.00332677 | 0.001095217 | 0.417227 | 0.1367678 | 0.0638389 | 0.04513986 |
| set1 | BT4228 | 0.0000011 | 0.00000272 | 0.452523 | 0.1459614 | 0.0237768 | 0.02241968 |
| set1 | BT4235 | 0.00000359 | 0.00000543 | 0.0537917 | 0.02481012 | 0.0233963 | 0.02222682 |
| set1 | BT4236 | 0.000617345 | 0.000254352 | 0.802663 | 0.2295944 | 0.0805157 | 0.05309976 |
| set1 | BT4238 | 0.0011043 | 0.000422549 | 0.73287 | 0.2136786 | 0.0188754 | 0.01929496 |
| set1 | BT4240 | 0.00000184 | 0.00000357 | 0.44677 | 0.1444246 | 0.0730751 | 0.04968427 |
| set1 | BT4241 | 0.000000358 | 0.00000141 | 0.711914 | 0.2085249 | 0.0409106 | 0.03265549 |
| set1 | BT4242 | 0.000000225 | 0.00000111 | 0.495879 | 0.1568055 | 0.114022 | 0.06812172 |
| set1 | BT4243 | 0.00000219 | 0.00000398 | 0.34157 | 0.1163563 | 0.156681 | 0.08614046 |
| set1 | BT4254 | 0.00057545 | 0.000239833 | 0.981043 | 0.2673994 | 0.0347555 | 0.02920336 |
| set1 | BT4260 | 0.00133021 | 0.000494978 | 0.479432 | 0.1529258 | 0.00739789 | 0.01057458 |
| set1 | BT4284 | 0.0029805 | 0.000998271 | 0.100702 | 0.0419893 | 0.00815085 | 0.0111716 |
| set1 | BT4287 | 0.0091181 | 0.002688407 | 0.124745 | 0.05024534 | 0.0111258 | 0.01378209 |
| set1 | BT4293 | 0.000057896 | 0.000038 | 0.0353901 | 0.01753465 | 0.0357309 | 0.02966633 |
| set1 | BT4294 | 0.00000335 | 0.00000516 | 0.544062 | 0.1694977 | 0.38467 | 0.1682051 |
| set1 | BT4295 | 0.00000731 | 0.0000085 | 0.979278 | 0.2672056 | 0.1092 | 0.06626572 |
| set1 | BT4296 | 0.00000411 | 0.00000583 | 0.600971 | 0.1829033 | 0.378989 | 0.1663155 |
| set1 | BT4297 | 1.11313E-05 | 0.0000114 | 0.134159 | 0.05344901 | 0.310102 | 0.1435524 |
| set1 | BT4298 | 0.00000177 | 0.00000355 | 0.895015 | 0.2504363 | 0.119873 | 0.07032928 |
| set1 | BT4304 | 0.00275525 | 0.000932622 | 0.433359 | 0.1409847 | 0.678642 | 0.252404 |
| set1 | BT4305 | 0.00239809 | 0.000822394 | 0.676384 | 0.2005494 | 0.758907 | 0.2732436 |
| set1 | BT4314 | 0.00349064 | 0.001144684 | 0.202579 | 0.07528938 | 0.125389 | 0.07286655 |
| set1 | BT4316 | 5.98154E-05 | 0.0000389 | 0.115454 | 0.04725947 | 0.142741 | 0.08031132 |
| set1 | BT4317 | 0.00975355 | 0.002854636 | 0.0445542 | 0.02127243 | 0.0146831 | 0.01648009 |
| set1 | BT4320 | 0.00287819 | 0.000970368 | 0.0543588 | 0.02501437 | 0.488281 | 0.2000721 |
| set1 | BT4327 | 0.000000287 | 0.00000127 | 0.673005 | 0.1996946 | 0.015391 | 0.01693609 |
| set1 | BT4329 | 6.17E-08 | 0.000000654 | 0.204031 | 0.07569389 | 0.0445413 | 0.03469296 |
| set1 | BT4332 | 0.0277718 | 0.007298407 | 0.381463 | 0.127114 | 0.0612827 | 0.0438392 |
| set1 | BT4333 | 0.000434614 | 0.000189374 | 0.763977 | 0.2208823 | 0.016924 | 0.01804029 |
| set1 | BT4336 | 0.0258217 | 0.006845344 | 0.721533 | 0.2109332 | 0.422791 | 0.1802565 |
| set1 | BT4347 | 0.00000197 | 0.00000371 | 0.315462 | 0.1087507 | 0.0440405 | 0.03447888 |
| set1 | BT4351 | 0.000230918 | 0.000113415 | 0.358697 | 0.1205923 | 0.936133 | 0.3151038 |
| set1 | BT4353 | 0.000482195 | 0.000206539 | 0.634994 | 0.1904735 | 0.00994118 | 0.01281198 |
| set1 | BT4362 | 0.0368422 | 0.009388704 | 0.0483966 | 0.02280948 | 0.219259 | 0.1116 |
| set1 | BT4365 | 0.000579825 | 0.000241258 | 0.232356 | 0.08459147 | 0.111101 | 0.0669288 |
| set1 | BT4367 | 0.00139828 | 0.000515241 | 0.0915908 | 0.03869629 | 0.0173926 | 0.01829463 |
| set1 | BT4368 | 2.10162E-05 | 0.0000179 | 0.0208515 | 0.01116471 | 0.162147 | 0.08851465 |
| set1 | BT4369 | 0.000161737 | 0.0000841 | 0.0223375 | 0.01183447 | 0.129437 | 0.07465738 |
| set1 | BT4371 | 0.000106681 | 0.0000611 | 0.0585635 | 0.02662549 | 0.0157628 | 0.01720336 |
| set1 | BT4372 | 0.025568 | 0.006785218 | 0.498844 | 0.1575369 | 0.614375 | 0.2346998 |
| set1 | BT4373 | 0.0195164 | 0.005313387 | 0.549947 | 0.1709052 | 0.252102 | 0.1236095 |
| set1 | BT4385 | 0.000000137 | 0.000000919 | 0.018907 | 0.01024158 | 0.251833 | 0.1236095 |
| set1 | BT4387 | 8.19801E-05 | 0.0000501 | 0.48589 | 0.1545175 | 0.026767 | 0.02426917 |
| set1 | BT4389 | 0.0240584 | 0.006420621 | 0.285349 | 0.1001416 | 0.237674 | 0.1182879 |
| set1 | BT4395 | 0.000000101 | 0.000000835 | 0.335544 | 0.1145297 | 0.105651 | 0.06472751 |
| set1 | BT4410 | 0.000325035 | 0.000149185 | 0.162537 | 0.0627474 | 0.0151978 | 0.01681569 |
| set1 | BT4417 | 0.000291085 | 0.000136839 | 0.714563 | 0.2091487 | 0.00923275 | 0.01216601 |
| set1 | BT4431 | 0.00000187 | 0.0000036 | 0.162432 | 0.06274468 | 0.884921 | 0.303078 |
| set1 | BT4432 | 0.00931541 | 0.002742309 | 0.0524219 | 0.02430827 | 0.156319 | 0.08600586 |
| set1 | BT4438 | 0.000000969 | 0.00000256 | 0.814294 | 0.2322065 | 0.00758636 | 0.01075958 |
| set1 | BT4444 | 0.00559265 | 0.001744791 | 0.343932 | 0.1169631 | 0.359345 | 0.1599422 |
| set1 | BT4446 | 0.000000791 | 0.00000225 | 0.796534 | 0.2281113 | 0.0657617 | 0.04619024 |
| set1 | BT4454 | 0.000212326 | 0.000105864 | 0.755134 | 0.2185609 | 0.0894842 | 0.0570863 |
| set1 | BT4455 | 0.022543 | 0.006052477 | 0.679364 | 0.2010875 | 0.825314 | 0.2894153 |
| set1 | BT4456 | 0.00146851 | 0.000537196 | 0.6147 | 0.1857645 | 0.4081 | 0.1754709 |
| set1 | BT4460 | 0.00000235 | 0.0000041 | 0.0583219 | 0.02655483 | 0.627447 | 0.2384882 |
| set1 | BT4463 | 0.0249959 | 0.006653839 | 0.0619231 | 0.0279313 | 0.465009 | 0.1935772 |
| set1 | BT4469 | 0.00488354 | 0.001547808 | 0.83876 | 0.23778 | 0.270361 | 0.1304629 |
| set1 | BT4471 | 0.00823909 | 0.00245506 | 0.0249252 | 0.012961 | 0.470278 | 0.1950636 |
| set1 | BT4474 | 2.78762E-05 | 0.0000221 | 0.0710951 | 0.03146916 | 0.283762 | 0.1347153 |
| set1 | BT4491 | 0.00814192 | 0.002429932 | 0.0440699 | 0.02110795 | 0.676499 | 0.2519142 |
| set1 | BT4492 | 0.00114539 | 0.000435192 | 0.722867 | 0.211221 | 0.529845 | 0.2114654 |
| set1 | BT4500 | 0.000609271 | 0.000251711 | 0.0247928 | 0.01291439 | 0.087206 | 0.05597348 |
| set1 | BT4504 | 9.55398E-05 | 0.0000565 | 0.0185411 | 0.01007954 | 0.00751353 | 0.01068726 |
| set1 | BT4507 | 0.0189044 | 0.005167214 | 0.786767 | 0.2259034 | 0.0344947 | 0.02910082 |
| set1 | BT4509 | 0.00000128 | 0.00000297 | 0.0193842 | 0.01047189 | 0.00790481 | 0.01098828 |
| set1 | BT4518 | 0.000034845 | 0.0000261 | 0.153967 | 0.059995 | 0.485965 | 0.1996255 |
| set1 | BT4520 | 9.61826E-05 | 0.0000567 | 0.0751854 | 0.03297757 | 0.0463122 | 0.03560368 |
| set1 | BT4527 | 0.000021562 | 0.0000182 | 0.074038 | 0.03259265 | 0.0151201 | 0.01680173 |
| set1 | BT4528 | 0.00000165 | 0.00000342 | 0.203597 | 0.0755793 | 0.538163 | 0.2135346 |
| set1 | BT4541 | 0.00134544 | 0.000500153 | 0.0390029 | 0.01898975 | 0.553495 | 0.2176496 |
| set1 | BT4542 | 2.58885E-05 | 0.0000209 | 0.78833 | 0.2262446 | 0.563689 | 0.22083 |
| set1 | BT4548 | 0.0160702 | 0.004471662 | 0.284321 | 0.09983885 | 0.122261 | 0.0715017 |
| set1 | BT4550 | 0.000000947 | 0.00000253 | 0.0501223 | 0.02342176 | 0.0123099 | 0.01472884 |
| set1 | BT4554 | 0.000501843 | 0.000213866 | 0.197581 | 0.0738389 | 0.492717 | 0.2010323 |
| set1 | BT4555 | 0.00000244 | 0.00000414 | 0.295041 | 0.1027963 | 0.0829455 | 0.05413778 |
| set1 | BT4560 | 0.000388717 | 0.000172858 | 0.111936 | 0.04599086 | 0.189216 | 0.09926868 |
| set1 | BT4561 | 0.0367323 | 0.009367005 | 0.40397 | 0.1330705 | 0.521994 | 0.2095283 |
| set1 | BT4564 | 0.000154323 | 0.0000812 | 0.329805 | 0.1127301 | 0.0384835 | 0.0313837 |
| set1 | BT4572 | 0.00030747 | 0.000142856 | 0.746519 | 0.2167166 | 0.754246 | 0.2722331 |
| set1 | BT4574 | 0.0183334 | 0.005032952 | 0.310118 | 0.1072451 | 0.786512 | 0.2802272 |
| set1 | BT4576 | 0.000486085 | 0.00020797 | 0.244591 | 0.08819516 | 0.0194537 | 0.01974859 |
| set1 | BT4582 | 0.0121006 | 0.003465184 | 0.243785 | 0.08803457 | 0.0140022 | 0.01597513 |
| set1 | BT4583 | 0.00423898 | 0.001362345 | 0.776027 | 0.2235636 | 0.830944 | 0.2907092 |
| set1 | BT4584 | 0.000231177 | 0.000113467 | 0.113005 | 0.04633551 | 0.113147 | 0.06770672 |
| set1 | BT4588 | 0.00116922 | 0.000442912 | 0.176662 | 0.06742958 | 0.0112934 | 0.01386103 |
| set1 | BT4589 | 0.000656289 | 0.000268063 | 0.44094 | 0.1429122 | 0.0165054 | 0.01775913 |
| set1 | BT4590 | 0.000112382 | 0.0000634 | 0.269734 | 0.09557796 | 0.0116257 | 0.01412714 |
| set1 | BT4594 | 0.0132223 | 0.003760786 | 0.0184649 | 0.01004263 | 0.303029 | 0.141348 |
| set1 | BT4601 | 0.00000158 | 0.00000336 | 0.0607749 | 0.02748523 | 0.0102297 | 0.01304637 |
| set1 | BT4605 | 0.00000285 | 0.00000459 | 0.829129 | 0.23588 | 0.024617 | 0.02291425 |
| set1 | BT4610 | 0.00164861 | 0.000593329 | 0.100067 | 0.04176778 | 0.0714046 | 0.04886535 |
| set1 | BT4614 | 0.0133617 | 0.003797581 | 0.888889 | 0.2490106 | 0.0908043 | 0.05772775 |
| set1 | BT4615 | 0.0011302 | 0.000430935 | 0.480338 | 0.1531339 | 0.432071 | 0.1833079 |
| set1 | BT4617 | 2.72E-09 | 0.000000196 | 0.136192 | 0.05418747 | 0.154718 | 0.08530468 |
| set1 | BT4620 | 0.00000277 | 0.00000451 | 0.0951675 | 0.03991584 | 0.0221439 | 0.02151254 |
| set1 | BT4623 | 0.000727408 | 0.000292844 | 0.0914777 | 0.03866358 | 0.822213 | 0.2887421 |
| set1 | BT4628 | 0.000082713 | 0.0000504 | 0.0266356 | 0.01373804 | 0.644012 | 0.2426482 |
| set1 | BT4633 | 0.00601006 | 0.001859266 | 0.116304 | 0.04751081 | 0.293975 | 0.1381343 |
| set1 | BT4637 | 5.14035E-05 | 0.0000347 | 0.105686 | 0.04381908 | 0.572034 | 0.2232056 |
| set1 | BT4638 | 0.000177348 | 0.000091 | 0.0358893 | 0.01770212 | 0.45548 | 0.1904866 |
| set1 | BT4654 | 0.000021096 | 0.0000179 | 0.125169 | 0.0503766 | 0.0115364 | 0.01407689 |
| set1 | BT4658 | 0.0165003 | 0.004574522 | 0.0913509 | 0.0386235 | 0.273894 | 0.1316915 |
| set1 | BT4662 | 0.000263321 | 0.000126538 | 0.123238 | 0.04975447 | 0.417135 | 0.178467 |
| set1 | BT4665 | 0.00695548 | 0.002116742 | 0.026357 | 0.0136176 | 0.398868 | 0.1726138 |
| set1 | BT4666 | 0.00291287 | 0.000978535 | 0.401825 | 0.1325825 | 0.464389 | 0.1934408 |
| set1 | BT4673 | 4.47351E-05 | 0.0000314 | 0.609839 | 0.1848691 | 0.0734311 | 0.04985704 |
| set1 | BT4679 | 0.00143094 | 0.00052574 | 0.0256396 | 0.01329814 | 0.755196 | 0.2723751 |
| set1 | BT4680 | 3.11483E-05 | 0.000024 | 0.360593 | 0.1211286 | 0.056276 | 0.04107838 |
| set1 | BT4681 | 5.53374E-05 | 0.0000366 | 0.953612 | 0.2620969 | 0.0475641 | 0.03628911 |
| set1 | BT4683 | 0.00012236 | 0.0000676 | 0.169875 | 0.06520513 | 0.0114534 | 0.01402224 |
| set1 | BT4692 | 0.00601117 | 0.001859266 | 0.0288998 | 0.01474843 | 0.450463 | 0.1886571 |
| set1 | BT4698 | 0.000050485 | 0.0000343 | 0.152343 | 0.0594773 | 0.031718 | 0.02746871 |
| set1 | BT4699 | 0.00000926 | 0.00000999 | 0.122885 | 0.04964514 | 0.169921 | 0.0916686 |
| set1 | BT4703 | 0.000000536 | 0.00000179 | 0.197825 | 0.07386831 | 0.0104723 | 0.01322934 |
| set1 | BT4704 | 0.00107129 | 0.000411547 | 0.189233 | 0.0713879 | 0.816467 | 0.2873472 |
| set1 | BT4706 | 0.00000184 | 0.00000357 | 0.436299 | 0.1417503 | 0.251159 | 0.1234286 |
| set1 | BT4707 | 0.000653769 | 0.000267605 | 0.917852 | 0.2549225 | 0.0540927 | 0.0398413 |
| set1 | BT4708 | 0.000000176 | 0.000001 | 0.668144 | 0.1987853 | 0.105251 | 0.06450938 |
| set1 | BT4709 | 0.00000422 | 0.00000589 | 0.996478 | 0.2702549 | 0.911821 | 0.309475 |
| set1 | BT4711 | 0.00000051 | 0.00000175 | 0.347829 | 0.1180225 | 0.142005 | 0.0799892 |
| set1 | BT4712 | 0.00073586 | 0.000295775 | 0.0716179 | 0.03166576 | 0.48114 | 0.1983917 |
| set1 | BT4717 | 0.00180545 | 0.000640636 | 0.0786014 | 0.03415345 | 0.708582 | 0.2601553 |
| set1 | BT4725 | 0.00471595 | 0.001500976 | 0.544155 | 0.1694977 | 0.613873 | 0.2346045 |
| set1 | BT4726 | 0.0064985 | 0.001992088 | 0.0487737 | 0.02294245 | 0.741918 | 0.2688955 |
| set1 | BT4738 | 0.00224668 | 0.00077645 | 0.742685 | 0.2160709 | 0.0235266 | 0.02232318 |
| set1 | BT4739 | 0.00130201 | 0.000486158 | 0.698623 | 0.2056408 | 0.523603 | 0.2099445 |
| set1 | BT4743 | 0.0026846 | 0.000911971 | 0.190261 | 0.07173091 | 0.528659 | 0.2112709 |
| set1 | BT4747 | 9.13073E-05 | 0.0000545 | 0.135106 | 0.05380855 | 0.0145394 | 0.01640481 |
| set1 | BT4750 | 6.17794E-05 | 0.00004 | 0.0556272 | 0.02552997 | 0.0158625 | 0.01726084 |
| set1 | BT4754 | 0.000792013 | 0.00031633 | 0.0695812 | 0.03086692 | 0.049549 | 0.03739407 |
| set1 | BT4756 | 0.00166837 | 0.000599016 | 0.0934623 | 0.03930982 | 0.0353315 | 0.02948481 |
| set1 | BT4762 | 0.0029812 | 0.000998271 | 0.428438 | 0.1397979 | 0.115569 | 0.06868257 |
| set1 | BT4764 | 0.000288298 | 0.000135782 | 0.274388 | 0.09691435 | 0.16312 | 0.0888486 |
| set1 | BT4778 | 0.00288955 | 0.000973388 | 0.392408 | 0.1300081 | 0.288144 | 0.1360914 |
| set1 | BT4780 | 0.00629558 | 0.001934585 | 0.118787 | 0.04834528 | 0.611197 | 0.2339473 |
| set1 | tRNA_55 | 0.000272487 | 0.000130117 | 0.382797 | 0.1274807 | 0.0857064 | 0.05537415 |
| set2 | BT0023 | 0.15772 | 0.03414538 | 0.00284796 | 0.00199663 | 0.759118 | 0.2732525 |
| set2 | BT0029 | 0.418866 | 0.08091466 | 0.00492395 | 0.003225535 | 0.0316659 | 0.02744809 |
| set2 | BT0032 | 0.323075 | 0.06455203 | 3.59174E-05 | 0.0000484 | 0.177385 | 0.0945486 |
| set2 | BT0042 | 0.0519213 | 0.01279624 | 0.000551527 | 0.000482417 | 0.577947 | 0.224915 |
| set2 | BT0060 | 0.240744 | 0.04985224 | 0.00381207 | 0.002562488 | 0.950072 | 0.3180453 |
| set2 | BT0064 | 0.350477 | 0.0691148 | 0.0122671 | 0.007125281 | 0.877732 | 0.3015304 |
| set2 | BT0077 | 0.156498 | 0.03392935 | 0.00583 | 0.00374195 | 0.136278 | 0.07750681 |
| set2 | BT0082 | 0.661529 | 0.1198961 | 1.26926E-05 | 0.0000205 | 0.0352232 | 0.02946143 |
| set2 | BT0083 | 0.497192 | 0.09426493 | 4.14591E-05 | 0.0000548 | 0.243036 | 0.1201203 |
| set2 | BT0094 | 0.0615043 | 0.01491039 | 0.000230162 | 0.000229962 | 0.727482 | 0.2649574 |
| set2 | BT0126 | 0.773811 | 0.1370946 | 1.96992E-05 | 0.0000293 | 0.071107 | 0.04868439 |
| set2 | BT0139 | 0.328795 | 0.0655392 | 0.0168651 | 0.009315136 | 0.165053 | 0.08970176 |
| set2 | BT0144 | 0.930605 | 0.1599157 | 1.89744E-05 | 0.0000284 | 0.352009 | 0.1577292 |
| set2 | BT0148 | 0.179164 | 0.03829575 | 0.000000682 | 0.00000172 | 0.0309799 | 0.02700474 |
| set2 | BT0150 | 0.315425 | 0.06317362 | 2.76372E-05 | 0.000039 | 0.0492933 | 0.03731747 |
| set2 | BT0156 | 0.0839546 | 0.01953941 | 0.000628818 | 0.000539092 | 0.0902764 | 0.0574668 |
| set2 | BT0166 | 0.0655435 | 0.01574337 | 0.00311291 | 0.002152399 | 0.891112 | 0.304488 |
| set2 | BT0170 | 0.687229 | 0.1240041 | 0.00599408 | 0.003830979 | 0.0854077 | 0.05522978 |
| set2 | BT0176 | 0.0795003 | 0.01863448 | 7.65613E-05 | 0.0000919 | 0.0963829 | 0.06056605 |
| set2 | BT0177 | 0.843947 | 0.1473474 | 0.012721 | 0.007345285 | 0.546924 | 0.2161009 |
| set2 | BT0199 | 0.0404549 | 0.01022329 | 0.000466682 | 0.000417577 | 0.684901 | 0.2540761 |
| set2 | BT0202 | 0.0901906 | 0.02077353 | 0.00289662 | 0.002021359 | 0.716635 | 0.2624412 |
| set2 | BT0203 | 0.0700774 | 0.01665795 | 0.00561761 | 0.003619754 | 0.464339 | 0.1934408 |
| set2 | BT0206 | 0.652861 | 0.1186376 | 0.00374174 | 0.00252223 | 0.144746 | 0.08115944 |
| set2 | BT0207 | 0.958282 | 0.1639808 | 0.000103804 | 0.000118853 | 0.754751 | 0.2723484 |
| set2 | BT0213 | 0.0411355 | 0.01038142 | 0.00210692 | 0.001543393 | 0.728232 | 0.2650597 |
| set2 | BT0214 | 0.150914 | 0.03285045 | 0.00834746 | 0.005118435 | 0.0164731 | 0.01775417 |
| set2 | BT0217 | 0.841636 | 0.1470457 | 0.00000014 | 0.00000048 | 0.218912 | 0.1114621 |
| set2 | BT0219 | 0.232376 | 0.04823821 | 0.00996103 | 0.005947815 | 0.731526 | 0.2660607 |
| set2 | BT0225 | 0.530431 | 0.09954449 | 0.000000112 | 0.000000394 | 0.0509618 | 0.03818673 |
| set2 | BT0243 | 0.768061 | 0.1362992 | 0.0000063 | 0.0000114 | 0.260397 | 0.1269063 |
| set2 | BT0250 | 0.747593 | 0.1332292 | 2.21E-10 | 2.88E-09 | 0.0409664 | 0.03268226 |
| set2 | BT0259 | 0.653506 | 0.1187241 | 4.95E-08 | 0.000000205 | 0.0708384 | 0.04854577 |
| set2 | BT0260 | 0.0664867 | 0.0159598 | 0.00367266 | 0.00248438 | 0.650463 | 0.2445766 |
| set2 | BT0278 | 0.16051 | 0.03472952 | 0.00406303 | 0.002708575 | 0.397224 | 0.1720037 |
| set2 | BT0279 | 0.622487 | 0.1141319 | 0.0109571 | 0.006456406 | 0.744816 | 0.2695586 |
| set2 | BT0289 | 0.918277 | 0.1582463 | 0.00855593 | 0.005219767 | 0.777605 | 0.2782694 |
| set2 | BT0292 | 0.0832975 | 0.01941031 | 0.000000687 | 0.00000173 | 0.867733 | 0.2991456 |
| set2 | BT0293 | 0.0534152 | 0.01312443 | 5.48E-12 | 1.74E-10 | 0.183057 | 0.09693826 |
| set2 | BT0297 | 0.225633 | 0.04698038 | 0.00130513 | 0.00100995 | 0.2954 | 0.1384935 |
| set2 | BT0307 | 0.188976 | 0.04010264 | 0.000447706 | 0.000403286 | 0.409438 | 0.1758386 |
| set2 | BT0331 | 0.861936 | 0.1501073 | 0.00000396 | 0.00000777 | 0.0609338 | 0.04361087 |
| set2 | BT0335 | 0.0841111 | 0.01956977 | 0.00919474 | 0.005550619 | 0.0809644 | 0.05331232 |
| set2 | BT0354 | 0.96343 | 0.1646384 | 0.000583896 | 0.00050633 | 0.0158616 | 0.01726084 |
| set2 | BT0361 | 0.382944 | 0.0746422 | 0.0103207 | 0.006117219 | 0.523838 | 0.2099477 |
| set2 | BT0379 | 0.370254 | 0.07237373 | 0.00377345 | 0.002539354 | 0.51824 | 0.2082866 |
| set2 | BT0381 | 0.292294 | 0.0590724 | 9.45481E-05 | 0.000109396 | 0.658231 | 0.246739 |
| set2 | BT0384 | 0.0782906 | 0.01837369 | 0.00550227 | 0.003556163 | 0.118085 | 0.0697823 |
| set2 | BT0387 | 0.110629 | 0.02492732 | 0.000157976 | 0.000169023 | 0.0533934 | 0.0395445 |
| set2 | BT0388 | 0.967656 | 0.1652486 | 2.08765E-05 | 0.0000308 | 0.84518 | 0.2941337 |
| set2 | BT0397 | 0.0437752 | 0.01097806 | 7.70929E-05 | 0.0000924 | 0.778562 | 0.2783263 |
| set2 | BT0400 | 0.197255 | 0.04170245 | 0.00000088 | 0.00000215 | 0.0345406 | 0.02910794 |
| set2 | BT0409 | 0.0719633 | 0.01705268 | 0.000268178 | 0.000263008 | 0.942947 | 0.3167437 |
| set2 | BT0412 | 0.619192 | 0.1135828 | 0.00333123 | 0.002282451 | 0.0325684 | 0.02794142 |
| set2 | BT0415 | 0.341568 | 0.06765761 | 0.000000713 | 0.00000179 | 0.0124892 | 0.01483643 |
| set2 | BT0437 | 0.979049 | 0.1668554 | 0.00135661 | 0.001043105 | 0.0451663 | 0.03498244 |
| set2 | BT0444 | 0.576897 | 0.1069912 | 0.00119251 | 0.000934778 | 0.0827933 | 0.05413654 |
| set2 | BT0445 | 0.41998 | 0.08106779 | 0.00000223 | 0.00000468 | 0.950966 | 0.3182176 |
| set2 | BT0477 | 0.268881 | 0.05486809 | 0.00066354 | 0.000564056 | 0.485193 | 0.1995025 |
| set2 | BT0484 | 0.144111 | 0.03155108 | 0.00752195 | 0.004697591 | 0.828021 | 0.2898111 |
| set2 | BT0487 | 0.870689 | 0.1512488 | 0.0109429 | 0.006451186 | 0.854799 | 0.2964309 |
| set2 | BT0490 | 0.080707 | 0.01888808 | 2.02024E-05 | 0.0000299 | 0.134191 | 0.07658691 |
| set2 | BT0503 | 0.625268 | 0.1145586 | 0.000347554 | 0.000329277 | 0.673235 | 0.2510171 |
| set2 | BT0519 | 0.0651544 | 0.01565985 | 0.00745471 | 0.004665691 | 0.0848677 | 0.05497746 |
| set2 | BT0521 | 0.205287 | 0.04322365 | 4.08E-08 | 0.000000175 | 0.202871 | 0.104711 |
| set2 | BT0547 | 0.17906 | 0.03829575 | 4.52E-09 | 2.88E-08 | 0.0139102 | 0.01590269 |
| set2 | BT0570 | 0.0695704 | 0.01656344 | 0.000600611 | 0.000519333 | 0.064635 | 0.04557106 |
| set2 | BT0586 | 0.670002 | 0.1212575 | 0.000000193 | 0.000000615 | 0.724657 | 0.2641784 |
| set2 | BT0592 | 0.538818 | 0.100851 | 0.00100928 | 0.000810073 | 0.290096 | 0.1368372 |
| set2 | BT0595 | 0.762538 | 0.1354756 | 0.000011158 | 0.0000184 | 0.94146 | 0.3164614 |
| set2 | BT0596 | 0.538087 | 0.1007564 | 0.00101986 | 0.000818021 | 0.225971 | 0.1143818 |
| set2 | BT0599 | 0.753747 | 0.1341995 | 0.000605877 | 0.000523512 | 0.708732 | 0.2601553 |
| set2 | BT0602 | 0.488111 | 0.09279893 | 0.00158513 | 0.001198972 | 0.0146692 | 0.01648009 |
| set2 | BT0603 | 0.0505062 | 0.01249631 | 1.85646E-05 | 0.000028 | 0.0135049 | 0.01559142 |
| set2 | BT0604 | 0.209034 | 0.04386189 | 0.00200799 | 0.001481128 | 0.466012 | 0.1937869 |
| set2 | BT0608 | 0.675119 | 0.1220085 | 4.43511E-05 | 0.000058 | 0.733216 | 0.2664773 |
| set2 | BT0611 | 0.0477464 | 0.01185807 | 9.7E-11 | 1.5E-09 | 0.0907383 | 0.05772775 |
| set2 | BT0613 | 0.584522 | 0.1081404 | 0.000236519 | 0.000235729 | 0.251514 | 0.1235616 |
| set2 | BT0615 | 0.366586 | 0.07184229 | 0.00000562 | 0.0000103 | 0.615497 | 0.2350416 |
| set2 | BT0623 | 0.379508 | 0.07404874 | 0.00796116 | 0.004916506 | 0.564312 | 0.221015 |
| set2 | BT0641 | 0.869551 | 0.1511552 | 0.00000541 | 0.00001 | 0.0187646 | 0.01923528 |
| set2 | BT0642 | 0.900543 | 0.1556134 | 0.00112133 | 0.000887041 | 0.194855 | 0.1014259 |
| set2 | BT0646 | 0.0990834 | 0.02260851 | 0.000737358 | 0.000616813 | 0.486833 | 0.199841 |
| set2 | BT0660 | 0.897405 | 0.155142 | 0.00000915 | 0.0000154 | 0.195726 | 0.1017747 |
| set2 | BT0674 | 0.163675 | 0.03536379 | 5.82311E-05 | 0.0000733 | 0.0574082 | 0.04165625 |
| set2 | BT0676 | 0.216844 | 0.04537488 | 0.0158826 | 0.008837845 | 0.0410016 | 0.03269258 |
| set2 | BT0689 | 0.198235 | 0.04188623 | 0.00731176 | 0.00459534 | 0.758437 | 0.2732084 |
| set2 | BT0707 | 0.115551 | 0.02591293 | 0.00000061 | 0.00000158 | 0.453779 | 0.1899375 |
| set2 | BT0710 | 0.149286 | 0.03258981 | 0.000703128 | 0.00059229 | 0.284348 | 0.1349062 |
| set2 | BT0712 | 0.587532 | 0.1085381 | 8.83967E-05 | 0.000103557 | 0.697351 | 0.2572532 |
| set2 | BT0729 | 0.0879619 | 0.02033445 | 0.00000178 | 0.00000387 | 0.290634 | 0.137047 |
| set2 | BT0738 | 0.0458666 | 0.01144093 | 0.0072262 | 0.004551038 | 0.79642 | 0.282728 |
| set2 | BT0743 | 0.851322 | 0.148498 | 3.72538E-05 | 0.0000499 | 0.380988 | 0.1670468 |
| set2 | BT0746 | 0.137498 | 0.03025357 | 0.00000626 | 0.0000113 | 0.883828 | 0.3027956 |
| set2 | BT0807 | 0.643203 | 0.117305 | 6.00735E-05 | 0.0000751 | 0.0463172 | 0.03560368 |
| set2 | BT0811 | 0.0451929 | 0.01128126 | 0.00138737 | 0.001064047 | 0.0596732 | 0.04298107 |
| set2 | BT0812 | 0.428553 | 0.08253317 | 0.000000149 | 0.000000506 | 0.0400244 | 0.03222851 |
| set2 | BT0813 | 0.939401 | 0.1612157 | 0.00018366 | 0.000191087 | 0.93442 | 0.3146715 |
| set2 | BT0818 | 0.0553675 | 0.01355728 | 0.000737857 | 0.000616813 | 0.572521 | 0.2233019 |
| set2 | BT0823 | 0.467025 | 0.0890361 | 0.00860789 | 0.005246167 | 0.664114 | 0.2484577 |
| set2 | BT0825 | 0.266265 | 0.05443068 | 0.000545204 | 0.000477578 | 0.444892 | 0.1871248 |
| set2 | BT0827 | 0.112695 | 0.0253325 | 0.000418526 | 0.000381842 | 0.02731 | 0.02457932 |
| set2 | BT0828 | 0.732066 | 0.1308627 | 0.00335933 | 0.002299096 | 0.467813 | 0.1942607 |
| set2 | BT0831 | 0.0830313 | 0.01936617 | 0.00977762 | 0.005852765 | 0.407042 | 0.1751186 |
| set2 | BT0835 | 0.276012 | 0.05612652 | 0.00971008 | 0.005815218 | 0.381248 | 0.1670672 |
| set2 | BT0855 | 0.540671 | 0.1010902 | 0.00085076 | 0.000697202 | 0.334792 | 0.1517703 |
| set2 | BT0862 | 0.155967 | 0.03384331 | 3.63623E-05 | 0.0000489 | 0.0675044 | 0.04685154 |
| set2 | BT0868 | 0.320332 | 0.06408856 | 7.48026E-05 | 0.0000902 | 0.816479 | 0.2873472 |
| set2 | BT0878 | 0.0643762 | 0.01550235 | 0.000000479 | 0.00000129 | 0.133398 | 0.07622323 |
| set2 | BT0879 | 0.0817345 | 0.01911082 | 0.00155903 | 0.001182191 | 0.0267209 | 0.02425734 |
| set2 | BT0886 | 0.540851 | 0.1010989 | 0.00113046 | 0.00089251 | 0.396699 | 0.1719207 |
| set2 | BT0890 | 0.341021 | 0.06758464 | 6.07E-09 | 3.59E-08 | 0.0518529 | 0.03867676 |
| set2 | BT0895 | 0.979764 | 0.1669363 | 0.00430288 | 0.002854292 | 0.566815 | 0.2217587 |
| set2 | BT0899 | 0.0940089 | 0.02156767 | 0.000441137 | 0.00039826 | 0.853957 | 0.2962751 |
| set2 | BT0901 | 0.0699369 | 0.01664023 | 0.0100001 | 0.005965246 | 0.0818466 | 0.05370812 |
| set2 | BT0957 | 0.465206 | 0.08871167 | 0.000166901 | 0.000176077 | 0.335075 | 0.1518096 |
| set2 | BT0962 | 0.850374 | 0.1483918 | 0.00571916 | 0.003674713 | 0.32262 | 0.1479013 |
| set2 | BT0965 | 0.0403689 | 0.01020497 | 0.00108272 | 0.000861576 | 0.949046 | 0.3178469 |
| set2 | BT0969 | 0.336059 | 0.0667502 | 0.000204515 | 0.000209182 | 0.0935927 | 0.05914185 |
| set2 | BT0971 | 0.189388 | 0.04017391 | 0.000053649 | 0.0000683 | 0.0174291 | 0.01830062 |
| set2 | BT0994 | 0.464397 | 0.08857972 | 0.00249331 | 0.001778969 | 0.85121 | 0.2956288 |
| set2 | BT0997 | 0.503409 | 0.09522894 | 0.0164686 | 0.009112804 | 0.152505 | 0.08419143 |
| set2 | BT1001 | 0.246691 | 0.05090267 | 0.00220909 | 0.001603648 | 0.436237 | 0.1844229 |
| set2 | BT1004 | 0.573667 | 0.1065227 | 0.000215893 | 0.000218966 | 0.0619053 | 0.04417174 |
| set2 | BT1015 | 0.0845371 | 0.01965414 | 0.000038507 | 0.0000513 | 0.0512833 | 0.03836888 |
| set2 | BT1016 | 0.0404948 | 0.01022996 | 9.44324E-05 | 0.000109367 | 0.55631 | 0.2184369 |
| set2 | BT1035 | 0.865823 | 0.150611 | 2.58174E-05 | 0.0000368 | 0.224558 | 0.1138235 |
| set2 | BT1054 | 0.826351 | 0.1448394 | 7.94E-10 | 7.61E-09 | 0.0280484 | 0.02509006 |
| set2 | BT1055 | 0.150733 | 0.03282049 | 5.55E-08 | 0.000000228 | 0.173201 | 0.0929598 |
| set2 | BT1061 | 0.119866 | 0.02673799 | 0.00000777 | 0.0000135 | 0.0480145 | 0.03653779 |
| set2 | BT1065 | 0.205307 | 0.04322365 | 0.000243641 | 0.000242427 | 0.772521 | 0.2767875 |
| set2 | BT1072 | 0.0778198 | 0.01827454 | 5.46E-08 | 0.000000225 | 0.188531 | 0.09901548 |
| set2 | BT1073 | 0.186476 | 0.03961172 | 6.41E-08 | 0.000000253 | 0.0379782 | 0.03109254 |
| set2 | BT1081 | 0.503311 | 0.09522894 | 0.00431509 | 0.00286082 | 0.0677376 | 0.04699117 |
| set2 | BT1090 | 0.49249 | 0.09354545 | 0.000138906 | 0.000151985 | 0.0313134 | 0.02727204 |
| set2 | BT1111 | 0.164081 | 0.03544139 | 0.00884535 | 0.005369218 | 0.436217 | 0.1844229 |
| set2 | BT1113 | 0.165369 | 0.03565854 | 0.00121145 | 0.000949009 | 0.547054 | 0.2161009 |
| set2 | BT1117 | 0.179208 | 0.03829575 | 0.0160707 | 0.008937664 | 0.630871 | 0.239344 |
| set2 | BT1118 | 0.0513127 | 0.01265861 | 1.52E-09 | 1.23E-08 | 0.0869583 | 0.05588864 |
| set2 | BT1123 | 0.153885 | 0.03341069 | 5.69E-10 | 6.03E-09 | 0.0148608 | 0.01660736 |
| set2 | BT1124 | 0.119458 | 0.02667056 | 0.00827779 | 0.005080873 | 0.23097 | 0.1162707 |
| set2 | BT1125 | 0.0921949 | 0.02121581 | 1.41752E-05 | 0.0000223 | 0.626177 | 0.2382522 |
| set2 | BT1127 | 0.111324 | 0.02504776 | 1.54E-13 | 1.04E-11 | 0.105105 | 0.0644468 |
| set2 | BT1135 | 0.575523 | 0.1067626 | 0.000698463 | 0.000588771 | 0.0433775 | 0.03408708 |
| set2 | BT1142 | 0.520467 | 0.09795977 | 0.000000616 | 0.00000159 | 0.103374 | 0.06370473 |
| set2 | BT1164 | 0.180475 | 0.03849204 | 2.74349E-05 | 0.0000388 | 0.0420083 | 0.03320667 |
| set2 | BT1165 | 0.164171 | 0.03544492 | 8.99841E-05 | 0.000105021 | 0.719078 | 0.2630321 |
| set2 | BT1166 | 0.445052 | 0.0853631 | 0.00000568 | 0.0000104 | 0.343996 | 0.1548952 |
| set2 | BT1169 | 0.650668 | 0.1184012 | 0.00216711 | 0.001581729 | 0.103525 | 0.06374857 |
| set2 | BT1173 | 0.111079 | 0.02501382 | 0.000219549 | 0.000221743 | 0.320101 | 0.147085 |
| set2 | BT1176 | 0.947242 | 0.1623595 | 0.000826298 | 0.000680851 | 0.0597161 | 0.04299088 |
| set2 | BT1180 | 0.0732512 | 0.01732532 | 0.00989024 | 0.005914316 | 0.44743 | 0.1878659 |
| set2 | BT1184 | 0.107619 | 0.02430698 | 4.55E-09 | 2.88E-08 | 0.0532278 | 0.03948161 |
| set2 | BT1213 | 0.391168 | 0.07601029 | 0.00000191 | 0.00000412 | 0.0323812 | 0.02784592 |
| set2 | BT1215 | 0.556722 | 0.103707 | 0.00000303 | 0.00000613 | 0.0196472 | 0.01984893 |
| set2 | BT1216 | 0.417974 | 0.08076296 | 0.00000415 | 0.00000806 | 0.330391 | 0.1505704 |
| set2 | BT1224 | 0.471078 | 0.08974098 | 0.000746295 | 0.000622761 | 0.056241 | 0.04107325 |
| set2 | BT1225 | 0.128001 | 0.02834379 | 0.00474021 | 0.003120402 | 0.0869596 | 0.05588864 |
| set2 | BT1230 | 0.471511 | 0.08980086 | 1.16E-09 | 1.02E-08 | 0.119729 | 0.07032928 |
| set2 | BT1247 | 0.344392 | 0.06807446 | 0.000403205 | 0.000370663 | 0.601306 | 0.2314286 |
| set2 | BT1255 | 0.860705 | 0.1499274 | 0.00000313 | 0.00000628 | 0.085267 | 0.05516309 |
| set2 | BT1257 | 0.280775 | 0.05704909 | 0.0018569 | 0.001382887 | 0.741175 | 0.2687626 |
| set2 | BT1258 | 0.0670625 | 0.01606235 | 0.000225306 | 0.000226619 | 0.0102127 | 0.01303602 |
| set2 | BT1261 | 0.703313 | 0.1264372 | 1.04703E-05 | 0.0000173 | 0.185311 | 0.0978143 |
| set2 | BT1264 | 0.348835 | 0.06888074 | 0.00000825 | 0.0000142 | 0.127132 | 0.07359855 |
| set2 | BT1285 | 0.0493479 | 0.01222971 | 3.62083E-05 | 0.0000488 | 0.00872997 | 0.01165029 |
| set2 | BT1292 | 0.99447 | 0.1689509 | 0.0092783 | 0.005598264 | 0.531201 | 0.2116218 |
| set2 | BT1304 | 0.122689 | 0.0272953 | 0.000923176 | 0.000749429 | 0.0208536 | 0.02073943 |
| set2 | BT1319 | 0.317839 | 0.06364026 | 0.000163131 | 0.000173311 | 0.13041 | 0.07501228 |
| set2 | BT1334 | 0.0592867 | 0.01442824 | 0.000728974 | 0.000610655 | 0.0183437 | 0.01894555 |
| set2 | BT1336 | 0.120878 | 0.02692192 | 0.0075429 | 0.004706327 | 0.318406 | 0.1464727 |
| set2 | BT1381 | 0.782514 | 0.1382804 | 0.00241907 | 0.001732138 | 0.138846 | 0.07860189 |
| set2 | BT1405 | 0.369548 | 0.07225439 | 0.00231065 | 0.001669348 | 0.287313 | 0.13583 |
| set2 | BT1411 | 0.139909 | 0.03072889 | 2.15699E-05 | 0.0000317 | 0.329704 | 0.1503974 |
| set2 | BT1412 | 0.0859669 | 0.01992804 | 1.09387E-05 | 0.0000181 | 0.550898 | 0.2169771 |
| set2 | BT1415 | 0.434027 | 0.08352363 | 0.0150079 | 0.008451654 | 0.125518 | 0.07291265 |
| set2 | BT1420 | 0.909054 | 0.1569407 | 0.000470505 | 0.000419754 | 0.620059 | 0.2365375 |
| set2 | BT1421 | 0.745365 | 0.1328635 | 0.000774659 | 0.000642685 | 0.051463 | 0.03846411 |
| set2 | BT1459 | 0.17257 | 0.03705294 | 0.0101776 | 0.006053193 | 0.117182 | 0.06947241 |
| set2 | BT1469 | 0.0718798 | 0.01704357 | 4.31E-09 | 2.77E-08 | 0.0593521 | 0.04279179 |
| set2 | BT1474 | 0.143676 | 0.03146495 | 0.000875244 | 0.000714842 | 0.713818 | 0.2614991 |
| set2 | BT1482 | 0.979965 | 0.1669363 | 0.00696054 | 0.004399784 | 0.404376 | 0.1742709 |
| set2 | BT1494 | 0.450392 | 0.08621256 | 0.00587115 | 0.003758377 | 0.420096 | 0.1793679 |
| set2 | BT1497 | 0.134137 | 0.02958134 | 5.16131E-05 | 0.0000662 | 0.00762061 | 0.01077692 |
| set2 | BT1512 | 0.332992 | 0.06625365 | 0.000329363 | 0.000314758 | 0.00691885 | 0.01012593 |
| set2 | BT1513 | 0.222572 | 0.04644506 | 6.01653E-05 | 0.0000752 | 0.0619597 | 0.04417282 |
| set2 | BT1522 | 0.23469 | 0.04867851 | 0.000614128 | 0.000529505 | 0.855075 | 0.2964358 |
| set2 | BT1523 | 0.56496 | 0.1050398 | 1.64408E-05 | 0.0000254 | 0.822888 | 0.288841 |
| set2 | BT1527 | 0.368821 | 0.07215617 | 0.00263664 | 0.001866899 | 0.0111448 | 0.01379398 |
| set2 | BT1531 | 0.0427163 | 0.01073384 | 0.00019413 | 0.000200256 | 0.604174 | 0.2321064 |
| set2 | BT1535 | 0.0980309 | 0.02240889 | 3.42575E-05 | 0.0000465 | 0.89677 | 0.3057133 |
| set2 | BT1536 | 0.193766 | 0.0410336 | 1.28446E-05 | 0.0000207 | 0.0109398 | 0.01365053 |
| set2 | BT1552 | 0.0671091 | 0.01606843 | 0.00782698 | 0.00484788 | 0.575609 | 0.2241835 |
| set2 | BT1556 | 0.805854 | 0.1416771 | 7.63E-10 | 7.38E-09 | 0.0749621 | 0.05061559 |
| set2 | BT1557 | 0.165462 | 0.03566843 | 0.000448514 | 0.000403713 | 0.204423 | 0.1053954 |
| set2 | BT1558 | 0.363156 | 0.07120853 | 0.00000546 | 0.0000101 | 0.856633 | 0.2967126 |
| set2 | BT1565 | 0.144632 | 0.03164684 | 0.000778843 | 0.000645713 | 0.515364 | 0.207605 |
| set2 | BT1567 | 0.442644 | 0.0849874 | 0.000154255 | 0.000165607 | 0.0236902 | 0.02241968 |
| set2 | BT1569 | 0.0687714 | 0.01640417 | 1.16536E-05 | 0.0000191 | 0.170058 | 0.09167513 |
| set2 | BT1585 | 0.156213 | 0.03387726 | 0.0107206 | 0.0063294 | 0.0171078 | 0.01813203 |
| set2 | BT1587 | 0.0596693 | 0.01450736 | 0.00126191 | 0.000982801 | 0.311689 | 0.1440146 |
| set2 | BT1593 | 0.786754 | 0.1388675 | 0.0149944 | 0.00844799 | 0.445631 | 0.1872839 |
| set2 | BT1598 | 0.505601 | 0.09554808 | 0.00336338 | 0.002300564 | 0.0125807 | 0.01490328 |
| set2 | BT1618 | 0.321163 | 0.06422087 | 7.29209E-05 | 0.0000882 | 0.635365 | 0.2404274 |
| set2 | BT1621 | 0.589789 | 0.1089284 | 0.000123843 | 0.00013775 | 0.541933 | 0.2145561 |
| set2 | BT1623 | 0.453774 | 0.08679408 | 0.00073919 | 0.0006175 | 0.85481 | 0.2964309 |
| set2 | BT1645 | 0.0447151 | 0.01118043 | 7.20014E-05 | 0.0000872 | 0.00907617 | 0.01199197 |
| set2 | BT1663 | 0.551478 | 0.1028567 | 9.10097E-05 | 0.000105911 | 0.1472 | 0.08194072 |
| set2 | BT1665 | 0.31483 | 0.06308784 | 7.75065E-05 | 0.0000928 | 0.0606825 | 0.04347655 |
| set2 | BT1674 | 0.081506 | 0.01906917 | 3.36528E-05 | 0.0000459 | 0.733077 | 0.2664773 |
| set2 | BT1690 | 0.062115 | 0.01502956 | 0.000758192 | 0.000630756 | 0.113154 | 0.06770672 |
| set2 | BT1702 | 0.119738 | 0.02671731 | 4.37546E-05 | 0.0000573 | 0.870136 | 0.2996222 |
| set2 | BT1708 | 0.764097 | 0.1356593 | 8.96372E-05 | 0.000104717 | 0.00954809 | 0.01242538 |
| set2 | BT1716 | 0.200177 | 0.04227297 | 0.000397113 | 0.000366178 | 0.329602 | 0.1503974 |
| set2 | BT1732 | 0.234301 | 0.04861115 | 0.00214597 | 0.001568194 | 0.0290893 | 0.02578547 |
| set2 | BT1738 | 0.14957 | 0.03264239 | 0.00307318 | 0.002127363 | 0.337946 | 0.1527332 |
| set2 | BT1753 | 0.532334 | 0.09987685 | 9.20877E-05 | 0.000106959 | 0.49367 | 0.2011848 |
| set2 | BT1761 | 0.0409373 | 0.01033485 | 0.00456046 | 0.003008634 | 0.377833 | 0.1658577 |
| set2 | BT1768 | 0.960512 | 0.1643252 | 0.0000011 | 0.00000259 | 0.123668 | 0.07211575 |
| set2 | BT1783 | 0.560031 | 0.1042208 | 0.0105775 | 0.006247968 | 0.312308 | 0.1442552 |
| set2 | BT1790 | 0.434426 | 0.08355792 | 0.00000304 | 0.00000616 | 0.370189 | 0.1635464 |
| set2 | BT1818 | 0.0665565 | 0.01597149 | 0.000882951 | 0.000719677 | 0.0131689 | 0.01535415 |
| set2 | BT1831 | 0.0755607 | 0.0178048 | 2.36064E-05 | 0.0000343 | 0.0410264 | 0.03269459 |
| set2 | BT1836 | 0.443944 | 0.08519377 | 0.00000172 | 0.00000377 | 0.469255 | 0.1947493 |
| set2 | BT1840 | 0.372694 | 0.0728285 | 0.00435439 | 0.00288213 | 0.00693423 | 0.01013834 |
| set2 | BT1845 | 0.688166 | 0.1241293 | 0.0026866 | 0.001897237 | 0.558814 | 0.2192127 |
| set2 | BT1855 | 0.0528769 | 0.01300634 | 0.00285504 | 0.001998347 | 0.101312 | 0.06280313 |
| set2 | BT1856 | 0.737484 | 0.131769 | 0.000000479 | 0.00000129 | 0.271795 | 0.1308967 |
| set2 | BT1857 | 0.0509328 | 0.01259774 | 1.85515E-05 | 0.000028 | 0.145989 | 0.08157061 |
| set2 | BT1858 | 0.633468 | 0.1158086 | 0.00849915 | 0.00519037 | 0.835366 | 0.2916864 |
| set2 | BT1878 | 0.0539144 | 0.01323144 | 0.00483214 | 0.003173999 | 0.0522849 | 0.03895939 |
| set2 | BT1897 | 0.176171 | 0.03772979 | 3.33338E-05 | 0.0000456 | 0.0481394 | 0.03659199 |
| set2 | BT1909 | 0.117428 | 0.02625606 | 0.000000152 | 0.000000512 | 0.202061 | 0.1043664 |
| set2 | BT1949 | 0.137783 | 0.03030592 | 9.51E-09 | 5.16E-08 | 0.0129166 | 0.01515629 |
| set2 | BT1960 | 0.537527 | 0.1007263 | 0.000822211 | 0.000677945 | 0.643292 | 0.2425637 |
| set2 | BT1961 | 0.0690877 | 0.01646924 | 1.66485E-05 | 0.0000257 | 0.0657099 | 0.04619024 |
| set2 | BT1964 | 0.776923 | 0.137485 | 0.00391558 | 0.002623309 | 0.313642 | 0.1447347 |
| set2 | BT1975 | 0.428377 | 0.08252027 | 0.00177016 | 0.001327291 | 0.449447 | 0.1885005 |
| set2 | BT1980 | 0.0451912 | 0.01128126 | 0.0103081 | 0.006115753 | 0.974045 | 0.3229041 |
| set2 | BT1981 | 0.690206 | 0.1244126 | 0.00290056 | 0.00202294 | 0.0172065 | 0.01819258 |
| set2 | BT1982 | 0.0628628 | 0.01518623 | 0.00146003 | 0.001114819 | 0.1952 | 0.1015371 |
| set2 | BT1984 | 0.206262 | 0.0433885 | 4.27312E-05 | 0.0000564 | 0.049238 | 0.03729484 |
| set2 | BT1985 | 0.366681 | 0.0718423 | 1.08E-09 | 9.76E-09 | 0.00966516 | 0.01255095 |
| set2 | BT1987 | 0.06996 | 0.01664049 | 2.48E-09 | 1.77E-08 | 0.0214961 | 0.02120601 |
| set2 | BT2001 | 0.876549 | 0.1520922 | 0.000677527 | 0.000574329 | 0.52221 | 0.2095577 |
| set2 | BT2004 | 0.142202 | 0.03117823 | 0.00761064 | 0.004743692 | 0.751133 | 0.2712429 |
| set2 | BT2012 | 0.212507 | 0.04455358 | 0.00071036 | 0.000597549 | 0.145426 | 0.08137081 |
| set2 | BT2019 | 0.106589 | 0.02411032 | 0.000453148 | 0.000407277 | 0.973769 | 0.3229041 |
| set2 | BT2022 | 0.320925 | 0.06419023 | 0.00291567 | 0.002031133 | 0.850564 | 0.295517 |
| set2 | BT2025 | 0.0546096 | 0.01339336 | 6.67859E-05 | 0.0000822 | 0.198621 | 0.1029882 |
| set2 | BT2037 | 0.261679 | 0.05361527 | 0.000000846 | 0.00000207 | 0.456561 | 0.1908299 |
| set2 | BT2043 | 0.230717 | 0.04795959 | 2.54E-09 | 0.000000018 | 0.280168 | 0.1337009 |
| set2 | BT2044 | 0.253306 | 0.05209718 | 4.73E-08 | 0.000000199 | 0.0809898 | 0.05331232 |
| set2 | BT2058 | 0.685182 | 0.1236977 | 0.000103458 | 0.000118569 | 0.698525 | 0.2574387 |
| set2 | BT2078 | 0.980728 | 0.1670287 | 0.000219745 | 0.000221755 | 0.0415344 | 0.03297412 |
| set2 | BT2080 | 0.135413 | 0.02983666 | 0.00000411 | 0.00000803 | 0.285013 | 0.1350907 |
| set2 | BT2095 | 0.103649 | 0.02353014 | 2.32886E-05 | 0.0000339 | 0.192486 | 0.1006604 |
| set2 | BT2096 | 0.642513 | 0.1172357 | 0.000307982 | 0.000296909 | 0.791265 | 0.2811937 |
| set2 | BT2100 | 0.0959304 | 0.02196701 | 0.0117081 | 0.006842188 | 0.940047 | 0.3161313 |
| set2 | BT2107 | 0.945545 | 0.1621679 | 0.000000164 | 0.000000542 | 0.924403 | 0.3123012 |
| set2 | BT2109 | 0.226423 | 0.0471058 | 0.00116097 | 0.000914804 | 0.231497 | 0.1164232 |
| set2 | BT2116 | 0.482489 | 0.09184544 | 0.000393696 | 0.000363304 | 0.00968564 | 0.01255684 |
| set2 | BT2117 | 0.28488 | 0.05779007 | 0.000459341 | 0.000412078 | 0.0191493 | 0.01950703 |
| set2 | BT2156 | 0.0569447 | 0.01390299 | 0.00000827 | 0.0000142 | 0.599495 | 0.2309738 |
| set2 | BT2157 | 0.041679 | 0.01049758 | 0.00117811 | 0.000925894 | 0.873407 | 0.3004666 |
| set2 | BT2159 | 0.661117 | 0.1198501 | 0.000582881 | 0.000506176 | 0.894975 | 0.3053814 |
| set2 | BT2166 | 0.0836557 | 0.01948778 | 0.000562647 | 0.000491076 | 0.85149 | 0.2956288 |
| set2 | BT2173 | 0.502681 | 0.09518696 | 5.54148E-05 | 0.0000703 | 0.914533 | 0.3098939 |
| set2 | BT2174 | 0.953544 | 0.1632438 | 0.00606519 | 0.003873179 | 0.210342 | 0.1078844 |
| set2 | BT2177 | 0.635686 | 0.116158 | 1.22563E-05 | 0.00002 | 0.0894127 | 0.05706548 |
| set2 | BT2197 | 0.248492 | 0.05123238 | 0.000000137 | 0.00000047 | 0.141212 | 0.07963419 |
| set2 | BT2198 | 0.0931373 | 0.02140014 | 2.74E-11 | 5.42E-10 | 0.365444 | 0.1622142 |
| set2 | BT2231 | 0.147539 | 0.03224563 | 0.00000695 | 0.0000123 | 0.0545501 | 0.04014534 |
| set2 | BT2235 | 0.584198 | 0.1081069 | 0.000446731 | 0.000402708 | 0.118662 | 0.07003869 |
| set2 | BT2241 | 0.252209 | 0.05188669 | 0.000000214 | 0.000000671 | 0.200441 | 0.1037121 |
| set2 | BT2247 | 0.148038 | 0.03233798 | 1.35165E-05 | 0.0000215 | 0.510493 | 0.2063791 |
| set2 | BT2261 | 0.251723 | 0.05181384 | 0.00111926 | 0.000885983 | 0.0743357 | 0.05023886 |
| set2 | BT2262 | 0.322444 | 0.06445999 | 0.0095103 | 0.00570689 | 0.536975 | 0.2132096 |
| set2 | BT2265 | 0.332655 | 0.06622142 | 0.0126462 | 0.007309082 | 0.188431 | 0.09899838 |
| set2 | BT2267 | 0.196089 | 0.04146752 | 0.000427352 | 0.000387738 | 0.0182058 | 0.01884671 |
| set2 | BT2271 | 0.552222 | 0.1029701 | 0.0149203 | 0.008410164 | 0.790573 | 0.281135 |
| set2 | BT2282 | 0.938015 | 0.1610589 | 3.58E-09 | 2.38E-08 | 0.762182 | 0.2740799 |
| set2 | BT2310 | 0.12322 | 0.02740538 | 0.00015309 | 0.000164641 | 0.621683 | 0.2369108 |
| set2 | BT2314 | 0.490922 | 0.09328649 | 0.00154937 | 0.001176343 | 0.0706634 | 0.04851644 |
| set2 | BT2317 | 0.40213 | 0.07791046 | 0.00000178 | 0.00000387 | 0.119077 | 0.07013228 |
| set2 | BT2321 | 0.802846 | 0.1412139 | 7.37302E-05 | 0.000089 | 0.0985149 | 0.06148465 |
| set2 | BT2326 | 0.185535 | 0.03944504 | 9.81E-08 | 0.000000354 | 0.942461 | 0.3166529 |
| set2 | BT2343 | 0.859524 | 0.1497645 | 0.000078935 | 0.000094 | 0.264118 | 0.1284269 |
| set2 | BT2360 | 0.102481 | 0.02329947 | 0.00000462 | 0.00000884 | 0.00904159 | 0.01195705 |
| set2 | BT2369 | 0.0727278 | 0.01721229 | 0.000445307 | 0.000401724 | 0.994779 | 0.3281472 |
| set2 | BT2371 | 0.329862 | 0.06573458 | 0.00363444 | 0.002460887 | 0.675126 | 0.2515305 |
| set2 | BT2382 | 0.150615 | 0.03280424 | 0.000349762 | 0.000330332 | 0.792923 | 0.2816874 |
| set2 | BT2388 | 0.507992 | 0.09590414 | 0.00481536 | 0.003166421 | 0.268461 | 0.1297862 |
| set2 | BT2400 | 0.707913 | 0.1271156 | 0.0103512 | 0.006129282 | 0.886828 | 0.3035187 |
| set2 | BT2404 | 0.165059 | 0.03560184 | 0.00204772 | 0.001506421 | 0.957823 | 0.3194196 |
| set2 | BT2414 | 0.932903 | 0.1602175 | 0.00563378 | 0.003627573 | 0.238924 | 0.1186096 |
| set2 | BT2416 | 0.731893 | 0.1308627 | 7.92054E-05 | 0.0000943 | 0.00760597 | 0.01077692 |
| set2 | BT2418 | 0.16811 | 0.03618772 | 0.00875899 | 0.005324824 | 0.521819 | 0.2095153 |
| set2 | BT2449 | 0.652624 | 0.1186355 | 0.000219264 | 0.00022164 | 0.320138 | 0.147085 |
| set2 | BT2464 | 0.149834 | 0.03269058 | 0.011427 | 0.006690862 | 0.344895 | 0.1552048 |
| set2 | BT2472 | 0.143621 | 0.03146201 | 0.000169092 | 0.000178233 | 0.0587116 | 0.04238072 |
| set2 | BT2477 | 0.926794 | 0.1593491 | 1.26999E-05 | 0.0000205 | 0.768952 | 0.2757106 |
| set2 | BT2479 | 0.33292 | 0.06625365 | 0.00566675 | 0.003644916 | 0.347835 | 0.156384 |
| set2 | BT2484 | 0.0639227 | 0.01540294 | 3.94977E-05 | 0.0000524 | 0.926867 | 0.3129896 |
| set2 | BT2498 | 0.20752 | 0.04360465 | 0.00547203 | 0.003538512 | 0.441315 | 0.1859682 |
| set2 | BT2500 | 0.135904 | 0.02993613 | 8.19E-08 | 0.000000312 | 0.916904 | 0.310554 |
| set2 | BT2502 | 0.443554 | 0.08514051 | 1.92185E-05 | 0.0000287 | 0.308809 | 0.1431344 |
| set2 | BT2503 | 0.86614 | 0.1506315 | 0.000000238 | 0.000000726 | 0.165428 | 0.08983904 |
| set2 | BT2504 | 0.361754 | 0.07100564 | 0.000000325 | 0.000000938 | 0.0988976 | 0.06164487 |
| set2 | BT2507 | 0.104622 | 0.02372279 | 2.11782E-05 | 0.0000312 | 0.411203 | 0.1764434 |
| set2 | BT2521 | 0.0852403 | 0.01978991 | 0.00487085 | 0.00319595 | 0.219697 | 0.1117455 |
| set2 | BT2522 | 0.0487704 | 0.01209848 | 0.000806111 | 0.00066649 | 0.601235 | 0.2314286 |
| set2 | BT2525 | 0.920254 | 0.1585488 | 4.26E-08 | 0.000000181 | 0.0810526 | 0.05332974 |
| set2 | BT2534 | 0.726867 | 0.1301177 | 0.00871741 | 0.005302215 | 0.972249 | 0.3227103 |
| set2 | BT2536 | 0.107389 | 0.02427677 | 0.000000232 | 0.000000714 | 0.0523622 | 0.0389972 |
| set2 | BT2539 | 0.247046 | 0.05096202 | 0.00126737 | 0.000985085 | 0.0412688 | 0.03283121 |
| set2 | BT2543 | 0.123956 | 0.02754479 | 0.00584927 | 0.003750333 | 0.943902 | 0.3169194 |
| set2 | BT2554 | 0.889523 | 0.1540256 | 0.000191637 | 0.000198192 | 0.34057 | 0.1535962 |
| set2 | BT2565 | 0.0551954 | 0.01352389 | 0.000228884 | 0.000229254 | 0.589117 | 0.2280528 |
| set2 | BT2575 | 0.0772743 | 0.01816334 | 0.00000779 | 0.0000135 | 0.874257 | 0.3006181 |
| set2 | BT2605 | 0.877088 | 0.1521509 | 0.000233758 | 0.000233169 | 0.722989 | 0.2637399 |
| set2 | BT2606 | 0.292461 | 0.05909037 | 0.000000304 | 0.000000881 | 0.0774525 | 0.05177323 |
| set2 | BT2617 | 0.30243 | 0.06092566 | 0.000140382 | 0.000153322 | 0.168411 | 0.09108833 |
| set2 | BT2619 | 0.0470544 | 0.01169963 | 0.00250083 | 0.001781185 | 0.0850937 | 0.05509954 |
| set2 | BT2632 | 0.387137 | 0.07530434 | 0.000000637 | 0.00000163 | 0.0355242 | 0.02956159 |
| set2 | BT2638 | 0.189895 | 0.0402589 | 0.000199003 | 0.000204236 | 0.40619 | 0.1748546 |
| set2 | BT2664 | 0.522972 | 0.0982665 | 3.84E-08 | 0.000000168 | 0.46648 | 0.1939266 |
| set2 | BT2665 | 0.112671 | 0.0253325 | 0.000669299 | 0.000568551 | 0.0229929 | 0.02195967 |
| set2 | BT2669 | 0.0628338 | 0.01518407 | 0.00225918 | 0.001636077 | 0.832884 | 0.2911261 |
| set2 | BT2683 | 0.309083 | 0.06208415 | 0.000269245 | 0.000263348 | 0.0925924 | 0.0586866 |
| set2 | BT2685 | 0.126574 | 0.02807709 | 0.00310207 | 0.002146132 | 0.710966 | 0.2608449 |
| set2 | BT2696 | 0.648861 | 0.118166 | 0.000109011 | 0.000123759 | 0.330234 | 0.1505456 |
| set2 | BT2698 | 0.541533 | 0.1011764 | 0.00136399 | 0.001048112 | 0.127846 | 0.0738558 |
| set2 | BT2699 | 0.214818 | 0.0449758 | 0.000138861 | 0.000151985 | 0.234801 | 0.117394 |
| set2 | BT2746 | 0.0682836 | 0.01631096 | 1.70183E-05 | 0.0000261 | 0.414803 | 0.1777283 |
| set2 | BT2754 | 0.712284 | 0.1278095 | 0.00000558 | 0.0000103 | 0.0172145 | 0.01819258 |
| set2 | BT2758 | 0.0416642 | 0.01049758 | 0.0119283 | 0.006954051 | 0.893573 | 0.3050568 |
| set2 | BT2760 | 0.607812 | 0.1116848 | 0.0116563 | 0.006818514 | 0.958773 | 0.3195734 |
| set2 | BT2766 | 0.916538 | 0.1580885 | 6.24468E-05 | 0.0000774 | 0.0176868 | 0.01845259 |
| set2 | BT2776 | 0.554554 | 0.1033286 | 0.000160308 | 0.000170762 | 0.765738 | 0.2749531 |
| set2 | BT2779 | 0.089445 | 0.02063319 | 0.00103697 | 0.000830642 | 0.412829 | 0.1769859 |
| set2 | BT2785 | 0.0553076 | 0.01354699 | 0.00000226 | 0.00000474 | 0.0110357 | 0.01374005 |
| set2 | BT2788 | 0.202763 | 0.04274751 | 0.0105288 | 0.006222244 | 0.86946 | 0.2994948 |
| set2 | BT2789 | 0.45345 | 0.08675403 | 0.000388325 | 0.000359171 | 0.0770777 | 0.05161671 |
| set2 | BT2792 | 0.384608 | 0.0748894 | 4.07E-08 | 0.000000175 | 0.595511 | 0.2296196 |
| set2 | BT2799 | 0.669097 | 0.1211227 | 0.0146842 | 0.00830032 | 0.372855 | 0.1643607 |
| set2 | BT2830 | 0.291582 | 0.05899151 | 0.00143345 | 0.001096603 | 0.291877 | 0.1374565 |
| set2 | BT2836 | 0.354313 | 0.06976221 | 5.93328E-05 | 0.0000743 | 0.616754 | 0.2353379 |
| set2 | BT2840 | 0.0926739 | 0.02130013 | 0.000118413 | 0.000132564 | 0.868464 | 0.2993273 |
| set2 | BT2843 | 0.0752845 | 0.01774524 | 0.00328144 | 0.002252168 | 0.594168 | 0.2292825 |
| set2 | BT2852 | 0.226048 | 0.04704071 | 7.17551E-05 | 0.000087 | 0.949995 | 0.3180453 |
| set2 | BT2874 | 0.647691 | 0.1180098 | 2.46509E-05 | 0.0000356 | 0.291505 | 0.1373694 |
| set2 | BT2877 | 0.120302 | 0.02681154 | 0.000719194 | 0.000603718 | 0.0763075 | 0.05121776 |
| set2 | BT2879 | 0.774012 | 0.1370981 | 0.00507587 | 0.003308904 | 0.970287 | 0.3224219 |
| set2 | BT2883 | 0.153133 | 0.03326651 | 0.000136618 | 0.000150299 | 0.514905 | 0.207591 |
| set2 | BT2888 | 0.258824 | 0.053131 | 2.64055E-05 | 0.0000376 | 0.0377018 | 0.03091515 |
| set2 | BT2899 | 0.0865521 | 0.02005141 | 4.95926E-05 | 0.0000637 | 0.224368 | 0.113804 |
| set2 | BT2926 | 0.495525 | 0.09397241 | 4.66E-08 | 0.000000197 | 0.0153837 | 0.01693609 |
| set2 | BT2939 | 0.176009 | 0.0377062 | 0.00265656 | 0.001877702 | 0.0776439 | 0.05185394 |
| set2 | BT2943 | 0.869782 | 0.1511607 | 0.00000483 | 0.00000915 | 0.0258505 | 0.02368692 |
| set2 | BT2949 | 0.0922539 | 0.02122293 | 0.00000214 | 0.00000453 | 0.514628 | 0.2075714 |
| set2 | BT2951 | 0.924665 | 0.1591277 | 0.00000148 | 0.00000332 | 0.831859 | 0.2909463 |
| set2 | BT2960 | 0.75287 | 0.1340749 | 0.000746519 | 0.000622761 | 0.369577 | 0.163417 |
| set2 | BT2966 | 0.284034 | 0.05765728 | 0.00648442 | 0.004122556 | 0.280635 | 0.133808 |
| set2 | BT2968 | 0.102908 | 0.02337549 | 0.000656109 | 0.000558919 | 0.368717 | 0.1631736 |
| set2 | BT2970 | 0.118349 | 0.02643075 | 0.00131102 | 0.001013859 | 0.310231 | 0.1435668 |
| set2 | BT2971 | 0.644325 | 0.1174247 | 0.00000631 | 0.0000114 | 0.0972808 | 0.06097367 |
| set2 | BT2987 | 0.580253 | 0.1074875 | 0.00000468 | 0.0000089 | 0.0386805 | 0.03150905 |
| set2 | BT2997 | 0.249902 | 0.05147978 | 0.00000359 | 0.00000708 | 0.90322 | 0.3074087 |
| set2 | BT2998 | 0.310931 | 0.06237259 | 4.54972E-05 | 0.0000592 | 0.194289 | 0.1013146 |
| set2 | BT2999 | 0.427976 | 0.08246402 | 0.00061794 | 0.000531275 | 0.2942 | 0.1381516 |
| set2 | BT3005 | 0.395929 | 0.07687622 | 2.29139E-05 | 0.0000334 | 0.0101425 | 0.01297101 |
| set2 | BT3009 | 0.0575753 | 0.01404789 | 0.00000718 | 0.0000126 | 0.506196 | 0.2050372 |
| set2 | BT3010 | 0.326865 | 0.06518883 | 0.00034325 | 0.000325966 | 0.356895 | 0.1591412 |
| set2 | BT3013 | 0.0558759 | 0.01366408 | 0.00153473 | 0.001165961 | 0.268605 | 0.1297862 |
| set2 | BT3026 | 0.744314 | 0.1327386 | 0.000266898 | 0.000262113 | 0.0377194 | 0.03091515 |
| set2 | BT3033 | 0.961799 | 0.1645082 | 0.0161799 | 0.008981854 | 0.905661 | 0.3080039 |
| set2 | BT3039 | 0.210226 | 0.04409978 | 0.0132082 | 0.007586709 | 0.0211237 | 0.02096544 |
| set2 | BT3070 | 0.362382 | 0.07110263 | 0.000270331 | 0.000263983 | 0.039145 | 0.0317995 |
| set2 | BT3072 | 0.671291 | 0.1214327 | 0.000182161 | 0.000190019 | 0.333743 | 0.1515335 |
| set2 | BT3080 | 0.0400799 | 0.01014207 | 0.0173447 | 0.009540772 | 0.160849 | 0.08800347 |
| set2 | BT3086 | 0.217337 | 0.0454529 | 4.91E-08 | 0.000000204 | 0.775687 | 0.2777862 |
| set2 | BT3097 | 0.0630533 | 0.01522253 | 0.00495247 | 0.003240703 | 0.755126 | 0.2723751 |
| set2 | BT3098 | 0.592521 | 0.1092997 | 0.00317635 | 0.002189996 | 0.988997 | 0.3266069 |
| set2 | BT3100 | 0.124021 | 0.02755114 | 0.000885866 | 0.000721566 | 0.135097 | 0.07695439 |
| set2 | BT3127 | 0.580942 | 0.1075833 | 0.00000535 | 0.00000994 | 0.35105 | 0.1573955 |
| set2 | BT3133 | 0.358067 | 0.07035493 | 5.63E-08 | 0.000000231 | 0.201719 | 0.1042264 |
| set2 | BT3135 | 0.0863947 | 0.02002108 | 1.66E-08 | 8.24E-08 | 0.0664126 | 0.04646727 |
| set2 | BT3137 | 0.0565369 | 0.01381234 | 0.000832402 | 0.000685413 | 0.0359037 | 0.0297593 |
| set2 | BT3141 | 0.799187 | 0.1406376 | 0.000940208 | 0.00076121 | 0.930414 | 0.3138264 |
| set2 | BT3153 | 0.397127 | 0.07706929 | 0.000000919 | 0.00000223 | 0.445653 | 0.1872839 |
| set2 | BT3160 | 0.343493 | 0.0679145 | 1.75963E-05 | 0.0000268 | 0.405351 | 0.1745958 |
| set2 | BT3161 | 0.793602 | 0.1399374 | 0.00272514 | 0.001919437 | 0.406019 | 0.1748322 |
| set2 | BT3167 | 0.046364 | 0.01154694 | 3.08E-10 | 3.58E-09 | 0.0340553 | 0.02879633 |
| set2 | BT3170 | 0.859572 | 0.1497645 | 0.000153199 | 0.000164641 | 0.0281838 | 0.02519582 |
| set2 | BT3183 | 0.757494 | 0.134645 | 0.00000448 | 0.00000863 | 0.343431 | 0.1546882 |
| set2 | BT3188 | 0.0565111 | 0.01381049 | 0.00000151 | 0.00000337 | 0.0363592 | 0.03006894 |
| set2 | BT3201 | 0.115176 | 0.02585181 | 0.0158469 | 0.00882946 | 0.627432 | 0.2384882 |
| set2 | BT3205 | 0.795868 | 0.140182 | 0.00000213 | 0.00000452 | 0.127334 | 0.073676 |
| set2 | BT3207 | 0.0447631 | 0.01118873 | 1.81135E-05 | 0.0000275 | 0.465936 | 0.1937869 |
| set2 | BT3219 | 0.440538 | 0.0846045 | 0.00111876 | 0.000885983 | 0.282514 | 0.1342904 |
| set2 | BT3220 | 0.169356 | 0.03642486 | 0.000851338 | 0.000697203 | 0.801857 | 0.284177 |
| set2 | BT3221 | 0.295542 | 0.05964918 | 4.32E-11 | 7.91E-10 | 0.683145 | 0.253617 |
| set2 | BT3223 | 0.0491011 | 0.01217652 | 4.25E-18 | 2.57E-15 | 0.716747 | 0.2624412 |
| set2 | BT3234 | 0.590431 | 0.1089938 | 0.00000711 | 0.0000125 | 0.0115672 | 0.01410275 |
| set2 | BT3250 | 0.33715 | 0.06694017 | 0.00000113 | 0.00000263 | 0.4934 | 0.2011306 |
| set2 | BT3251 | 0.651619 | 0.1185238 | 0.00000861 | 0.0000146 | 0.24072 | 0.1192164 |
| set2 | BT3253 | 0.0438249 | 0.01098688 | 0.000344049 | 0.000326468 | 0.146602 | 0.08175708 |
| set2 | BT3269 | 0.0413571 | 0.01042692 | 0.00342594 | 0.002335893 | 0.299844 | 0.1401295 |
| set2 | BT3274 | 0.157361 | 0.03408715 | 0.000076576 | 0.0000919 | 0.110947 | 0.06688346 |
| set2 | BT3281 | 0.306498 | 0.06164672 | 0.00564808 | 0.003634843 | 0.459786 | 0.1919046 |
| set2 | BT3282 | 0.638389 | 0.1166238 | 7.7E-09 | 4.32E-08 | 0.614442 | 0.2346998 |
| set2 | BT3283 | 0.823511 | 0.1444866 | 0.000063853 | 0.000079 | 0.0141945 | 0.01608987 |
| set2 | BT3310 | 0.273822 | 0.05578352 | 0.00000165 | 0.00000364 | 0.00681898 | 0.009999711 |
| set2 | BT3324 | 0.703149 | 0.1264372 | 2.57256E-05 | 0.0000368 | 0.154996 | 0.085406 |
| set2 | BT3330 | 0.834864 | 0.1462 | 0.00146246 | 0.001115969 | 0.986659 | 0.3260548 |
| set2 | BT3331 | 0.970316 | 0.1656281 | 0.00329793 | 0.002260917 | 0.952085 | 0.3185013 |
| set2 | BT3335 | 0.162623 | 0.03515656 | 1.37427E-05 | 0.0000219 | 0.861504 | 0.2976973 |
| set2 | BT3344 | 0.0684685 | 0.01634222 | 0.00240401 | 0.001724421 | 0.182688 | 0.09682278 |
| set2 | BT3352 | 0.401376 | 0.07779414 | 0.00000291 | 0.0000059 | 0.0251754 | 0.02325705 |
| set2 | BT3360 | 0.115319 | 0.02586856 | 1.76E-10 | 2.47E-09 | 0.0210879 | 0.02095825 |
| set2 | BT3368 | 0.342898 | 0.06781457 | 0.00000135 | 0.00000306 | 0.0447656 | 0.03480523 |
| set2 | BT3379 | 0.180962 | 0.03857032 | 0.00118488 | 0.000930004 | 0.603308 | 0.2318951 |
| set2 | BT3380 | 0.607338 | 0.1116248 | 0.00000936 | 0.0000157 | 0.0623011 | 0.04439465 |
| set2 | BT3384 | 0.113022 | 0.02539846 | 0.000108456 | 0.000123361 | 0.793909 | 0.2819047 |
| set2 | BT3386 | 0.780549 | 0.1380298 | 0.0127532 | 0.007360359 | 0.155632 | 0.08569212 |
| set2 | BT3398 | 0.572461 | 0.106351 | 0.000000403 | 0.00000112 | 0.271592 | 0.1308418 |
| set2 | BT3400 | 0.0438935 | 0.01100043 | 0.00000285 | 0.00000581 | 0.528701 | 0.2112709 |
| set2 | BT3413 | 0.862313 | 0.1501075 | 8.25E-09 | 4.55E-08 | 0.0231139 | 0.02203204 |
| set2 | BT3422 | 0.469992 | 0.08957919 | 2.52377E-05 | 0.0000362 | 0.896989 | 0.3057133 |
| set2 | BT3424 | 0.0938826 | 0.02154522 | 8.44954E-05 | 0.0000996 | 0.0162341 | 0.017574 |
| set2 | BT3427 | 0.459642 | 0.08778336 | 0.000622832 | 0.0005351 | 0.13813 | 0.07825691 |
| set2 | BT3428 | 0.330507 | 0.06584577 | 0.00000329 | 0.00000657 | 0.0737428 | 0.04999929 |
| set2 | BT3435 | 0.90302 | 0.1559346 | 0.000106967 | 0.000121782 | 0.115697 | 0.06870301 |
| set2 | BT3436 | 0.91524 | 0.1579366 | 0.00177763 | 0.001332065 | 0.252116 | 0.1236095 |
| set2 | BT3437 | 0.631359 | 0.1154959 | 0.00256056 | 0.001819429 | 0.352732 | 0.157825 |
| set2 | BT3438 | 0.657952 | 0.1193907 | 0.00518456 | 0.003374295 | 0.656163 | 0.24603 |
| set2 | BT3468 | 0.262709 | 0.05379718 | 0.00151013 | 0.00114944 | 0.511053 | 0.2065313 |
| set2 | BT3475 | 0.0826761 | 0.01929521 | 0.0132331 | 0.007597399 | 0.0374308 | 0.03076449 |
| set2 | BT3478 | 0.379361 | 0.07403915 | 0.000225519 | 0.000226635 | 0.0416961 | 0.03303103 |
| set2 | BT3491 | 0.620626 | 0.1138183 | 0.00112899 | 0.000891932 | 0.8044 | 0.2843674 |
| set2 | BT3492 | 0.952242 | 0.1631316 | 1.39806E-05 | 0.000022 | 0.836002 | 0.2918391 |
| set2 | BT3502 | 0.132117 | 0.02914436 | 0.000116937 | 0.000131072 | 0.366479 | 0.162379 |
| set2 | BT3509 | 0.606944 | 0.1115795 | 0.00147979 | 0.001127768 | 0.422241 | 0.1800892 |
| set2 | BT3511 | 0.110318 | 0.02487205 | 0.00886524 | 0.005375888 | 0.114219 | 0.0681562 |
| set2 | BT3513 | 0.220432 | 0.04606199 | 0.0000025 | 0.00000516 | 0.515608 | 0.2076463 |
| set2 | BT3516 | 0.436204 | 0.08385728 | 0.00000688 | 0.0000122 | 0.0120971 | 0.01453156 |
| set2 | BT3517 | 0.0896561 | 0.02066929 | 3.04E-10 | 3.56E-09 | 0.0329749 | 0.02815223 |
| set2 | BT3521 | 0.224551 | 0.04679352 | 3.08196E-05 | 0.0000426 | 0.600414 | 0.2312065 |
| set2 | BT3535 | 0.272041 | 0.05543819 | 0.000000129 | 0.000000445 | 0.151781 | 0.08391798 |
| set2 | BT3538 | 0.074862 | 0.01765115 | 0.000629383 | 0.000539194 | 0.0229674 | 0.02195967 |
| set2 | BT3549 | 0.649725 | 0.1182949 | 0.0146176 | 0.008266542 | 0.512341 | 0.2068983 |
| set2 | BT3554 | 0.167112 | 0.03598851 | 0.00115196 | 0.000908297 | 0.498734 | 0.2026057 |
| set2 | BT3578 | 0.125882 | 0.02793177 | 0.00136984 | 0.001051938 | 0.35058 | 0.1572328 |
| set2 | BT3579 | 0.409374 | 0.07920233 | 0.0101328 | 0.006029517 | 0.891789 | 0.3045871 |
| set2 | BT3582 | 0.228853 | 0.04758519 | 0.00201306 | 0.00148273 | 0.784853 | 0.2797719 |
| set2 | BT3588 | 0.0429072 | 0.01077501 | 0.00037241 | 0.000348183 | 0.0337666 | 0.02861815 |
| set2 | BT3594 | 0.0885731 | 0.02046141 | 0.00271094 | 0.001911664 | 0.814776 | 0.2868998 |
| set2 | BT3596 | 0.153321 | 0.03329779 | 0.0151816 | 0.00851375 | 0.778351 | 0.2783263 |
| set2 | BT3608 | 0.268699 | 0.05484574 | 0.00511952 | 0.003333761 | 0.933754 | 0.3145194 |
| set2 | BT3620 | 0.798115 | 0.1405451 | 0.0042056 | 0.002792829 | 0.0333814 | 0.02840649 |
| set2 | BT3626 | 0.27519 | 0.05597441 | 0.000421431 | 0.000383182 | 0.416609 | 0.178346 |
| set2 | BT3632 | 0.870042 | 0.1511711 | 0.00000846 | 0.0000144 | 0.743254 | 0.2692588 |
| set2 | BT3633 | 0.347089 | 0.06857175 | 0.00270082 | 0.00190564 | 0.149327 | 0.08290467 |
| set2 | BT3643 | 0.881262 | 0.1527699 | 2.22423E-05 | 0.0000325 | 0.172731 | 0.09274145 |
| set2 | BT3656 | 0.142438 | 0.03122093 | 0.000000643 | 0.00000165 | 0.00812935 | 0.01115255 |
| set2 | BT3668 | 0.336105 | 0.0667502 | 7.58E-09 | 4.28E-08 | 0.433033 | 0.1836094 |
| set2 | BT3669 | 0.862334 | 0.1501075 | 0.0012536 | 0.000978221 | 0.076625 | 0.05138387 |
| set2 | BT3671 | 0.640488 | 0.1169507 | 0.0152472 | 0.008542607 | 0.175848 | 0.09400453 |
| set2 | BT3690 | 0.0396589 | 0.01004562 | 2.84872E-05 | 0.00004 | 0.518109 | 0.2082866 |
| set2 | BT3714 | 0.794751 | 0.1400504 | 1.38471E-05 | 0.000022 | 0.0112427 | 0.01384597 |
| set2 | BT3718 | 0.717351 | 0.1286521 | 0.000245752 | 0.000243957 | 0.0212545 | 0.02105256 |
| set2 | BT3742 | 0.140621 | 0.03086736 | 0.0163437 | 0.009056413 | 0.010005 | 0.01284911 |
| set2 | BT3746 | 0.335294 | 0.0666416 | 2.66E-08 | 0.000000123 | 0.0199801 | 0.02008847 |
| set2 | BT3750 | 0.0830789 | 0.0193713 | 1.39785E-05 | 0.000022 | 0.851758 | 0.2956519 |
| set2 | BT3752 | 0.208485 | 0.043771 | 0.00000327 | 0.00000654 | 0.0178646 | 0.01857209 |
| set2 | BT3758 | 0.0870105 | 0.02013912 | 0.000583839 | 0.00050633 | 0.632874 | 0.2398058 |
| set2 | BT3760 | 0.871648 | 0.151346 | 0.00367325 | 0.00248438 | 0.672015 | 0.2506896 |
| set2 | BT3767 | 0.602501 | 0.1108974 | 0.000330471 | 0.000315567 | 0.101893 | 0.06313663 |
| set2 | BT3769 | 0.182796 | 0.0388955 | 0.00412928 | 0.002749705 | 0.00812085 | 0.01115255 |
| set2 | BT3770 | 0.533447 | 0.100041 | 0.000111561 | 0.000125944 | 0.0669903 | 0.04669342 |
| set2 | BT3772 | 0.22089 | 0.04613223 | 0.000000498 | 0.00000133 | 0.475839 | 0.1967586 |
| set2 | BT3773 | 0.0839548 | 0.01953941 | 0.00561864 | 0.003619754 | 0.550209 | 0.2168573 |
| set2 | BT3775 | 0.047471 | 0.01179934 | 0.000468344 | 0.000418754 | 0.71806 | 0.2627908 |
| set2 | BT3776 | 0.164798 | 0.03556581 | 0.0111144 | 0.006539524 | 0.303546 | 0.1414992 |
| set2 | BT3789 | 0.340996 | 0.06758464 | 0.0156875 | 0.008756802 | 0.805942 | 0.2844879 |
| set2 | BT3818 | 0.0669528 | 0.01604116 | 0.00140648 | 0.001076653 | 0.0270838 | 0.02442066 |
| set2 | BT3840 | 0.0694805 | 0.01654724 | 0.00399262 | 0.002666051 | 0.168155 | 0.09098339 |
| set2 | BT3841 | 0.548467 | 0.1023204 | 0.0170569 | 0.009408168 | 0.198415 | 0.1029177 |
| set2 | BT3856 | 0.369036 | 0.07217293 | 0.00000163 | 0.00000361 | 0.0787029 | 0.05222852 |
| set2 | BT3867 | 0.525288 | 0.09864569 | 0.00254871 | 0.001812075 | 0.183199 | 0.09697848 |
| set2 | BT3872 | 0.341921 | 0.06770666 | 0.000000361 | 0.00000102 | 0.320493 | 0.147156 |
| set2 | BT3879 | 0.455026 | 0.08696762 | 0.00000843 | 0.0000144 | 0.181412 | 0.09638007 |
| set2 | BT3890 | 0.131146 | 0.02894704 | 0.00323043 | 0.002219681 | 0.115274 | 0.06859056 |
| set2 | BT3899 | 0.142732 | 0.03127631 | 0.00000508 | 0.00000955 | 0.476983 | 0.1971761 |
| set2 | BT3906 | 0.279564 | 0.05681828 | 0.000399607 | 0.000367636 | 0.998317 | 0.3290103 |
| set2 | BT3924 | 0.179878 | 0.03838251 | 0.00000465 | 0.00000887 | 0.173271 | 0.09296338 |
| set2 | BT3933 | 0.292689 | 0.05912066 | 0.000011432 | 0.0000188 | 0.659528 | 0.247162 |
| set2 | BT3939 | 0.0421452 | 0.01060089 | 0.00213702 | 0.001563133 | 0.255705 | 0.1250347 |
| set2 | BT3942 | 0.564987 | 0.1050398 | 0.00061672 | 0.000530982 | 0.468198 | 0.1943656 |
| set2 | BT3943 | 0.0926127 | 0.02129253 | 0.00387728 | 0.002599091 | 0.756713 | 0.2727212 |
| set2 | BT3944 | 0.880602 | 0.1526905 | 0.000000833 | 0.00000204 | 0.101121 | 0.06276425 |
| set2 | BT3963 | 0.239576 | 0.04963753 | 2.72794E-05 | 0.0000387 | 0.152176 | 0.0840414 |
| set2 | BT4011 | 0.0760595 | 0.01791118 | 2.89078E-05 | 0.0000405 | 0.00705595 | 0.01027536 |
| set2 | BT4028 | 0.70675 | 0.1269369 | 8.84732E-05 | 0.000103557 | 0.607022 | 0.2328349 |
| set2 | BT4046 | 0.292808 | 0.05912891 | 7.14437E-05 | 0.0000868 | 0.357775 | 0.1594321 |
| set2 | BT4051 | 0.602089 | 0.1108485 | 0.00461356 | 0.003039009 | 0.402004 | 0.1735616 |
| set2 | BT4057 | 0.444657 | 0.08530896 | 0.00138365 | 0.001061868 | 0.00882091 | 0.01174989 |
| set2 | BT4079 | 0.0667138 | 0.01599402 | 0.000000108 | 0.000000382 | 0.354535 | 0.1583776 |
| set2 | BT4088 | 0.615583 | 0.113003 | 0.000000753 | 0.00000187 | 0.791332 | 0.2811937 |
| set2 | BT4090 | 0.998649 | 0.1695847 | 0.000116061 | 0.000130536 | 0.539684 | 0.2139761 |
| set2 | BT4093 | 0.397384 | 0.0770994 | 0.00663987 | 0.004212517 | 0.682368 | 0.2534127 |
| set2 | BT4095 | 0.612598 | 0.1124823 | 0.000127768 | 0.000141724 | 0.507841 | 0.2055334 |
| set2 | BT4096 | 0.0597916 | 0.01453243 | 0.0137414 | 0.007855622 | 0.450137 | 0.1885744 |
| set2 | BT4100 | 0.892844 | 0.1544622 | 0.00191299 | 0.001416798 | 0.0674993 | 0.04685154 |
| set2 | BT4103 | 0.14084 | 0.03090647 | 0.0157038 | 0.00875803 | 0.145042 | 0.08126333 |
| set2 | BT4111 | 0.358303 | 0.07038303 | 0.0168952 | 0.009327496 | 0.182868 | 0.09687313 |
| set2 | BT4116 | 0.447418 | 0.08576859 | 0.000287236 | 0.000278688 | 0.0279619 | 0.02503715 |
| set2 | BT4119 | 0.502684 | 0.09518696 | 0.00036254 | 0.000340802 | 0.891817 | 0.3045871 |
| set2 | BT4124 | 0.258903 | 0.0531328 | 0.000000676 | 0.00000172 | 0.295046 | 0.138416 |
| set2 | BT4150 | 0.267626 | 0.0546562 | 0.00416002 | 0.00276408 | 0.1247 | 0.07260988 |
| set2 | BT4155 | 0.286429 | 0.05805762 | 9.34131E-05 | 0.00010829 | 0.00809251 | 0.01113322 |
| set2 | BT4156 | 0.124632 | 0.02767063 | 1.26802E-05 | 0.0000205 | 0.586138 | 0.2272785 |
| set2 | BT4160 | 0.113747 | 0.0255538 | 0.000547993 | 0.000479673 | 0.450026 | 0.1885744 |
| set2 | BT4177 | 0.162098 | 0.03505308 | 7.76155E-05 | 0.0000928 | 0.0444759 | 0.03469296 |
| set2 | BT4178 | 0.252214 | 0.05188669 | 5.44442E-05 | 0.0000692 | 0.118954 | 0.07013228 |
| set2 | BT4182 | 0.0815465 | 0.01907275 | 0.000946321 | 0.000765646 | 0.386781 | 0.1687483 |
| set2 | BT4191 | 0.28854 | 0.0584542 | 0.0138405 | 0.007904793 | 0.193977 | 0.1012598 |
| set2 | BT4194 | 0.598092 | 0.1102199 | 0.00000346 | 0.00000685 | 0.32246 | 0.1478742 |
| set2 | BT4199 | 0.292072 | 0.0590433 | 0.0133022 | 0.007629818 | 0.274465 | 0.1318365 |
| set2 | BT4202 | 0.171275 | 0.03682713 | 0.000359719 | 0.000338413 | 0.23169 | 0.1164733 |
| set2 | BT4226 | 0.779521 | 0.1378803 | 0.000181513 | 0.00018967 | 0.240679 | 0.1192164 |
| set2 | BT4227 | 0.135168 | 0.02979135 | 0.00525346 | 0.003411784 | 0.665742 | 0.2488555 |
| set2 | BT4253 | 0.703518 | 0.1264372 | 0.00000159 | 0.00000354 | 0.0573125 | 0.04162796 |
| set2 | BT4255 | 0.211171 | 0.04428574 | 0.00196185 | 0.00144943 | 0.259132 | 0.1264577 |
| set2 | BT4256 | 0.493242 | 0.0936568 | 0.000044544 | 0.000058 | 0.498951 | 0.2026057 |
| set2 | BT4266 | 0.0769498 | 0.01809831 | 4.87E-09 | 3.05E-08 | 0.423325 | 0.1803453 |
| set2 | BT4300 | 0.25929 | 0.0531978 | 0.000419592 | 0.000382526 | 0.0176478 | 0.0184449 |
| set2 | BT4301 | 0.123303 | 0.02741578 | 0.000611523 | 0.000527635 | 0.504031 | 0.2043294 |
| set2 | BT4335 | 0.448505 | 0.08591654 | 0.000116183 | 0.000130552 | 0.959274 | 0.3196678 |
| set2 | BT4339 | 0.260132 | 0.05334409 | 7.04627E-05 | 0.0000858 | 0.0200729 | 0.02015417 |
| set2 | BT4344 | 0.20706 | 0.04354426 | 6.98E-12 | 2.06E-10 | 0.0690924 | 0.04775146 |
| set2 | BT4350 | 0.131578 | 0.02903392 | 0.000572621 | 0.000498341 | 0.682 | 0.2534127 |
| set2 | BT4355 | 0.24894 | 0.0512968 | 0.00000501 | 0.00000944 | 0.0106577 | 0.01337149 |
| set2 | BT4377 | 0.506055 | 0.09561 | 0.00611756 | 0.003901641 | 0.316198 | 0.145731 |
| set2 | BT4381 | 0.660821 | 0.1198251 | 0.00013228 | 0.000146058 | 0.118477 | 0.06995762 |
| set2 | BT4390 | 0.244007 | 0.05043134 | 0.000000989 | 0.00000236 | 0.0157597 | 0.01720336 |
| set2 | BT4396 | 0.56448 | 0.1049971 | 0.00000308 | 0.00000621 | 0.115139 | 0.06856123 |
| set2 | BT4397 | 0.771773 | 0.1368616 | 0.00179101 | 0.001339598 | 0.785626 | 0.2799795 |
| set2 | BT4400 | 0.0965492 | 0.0220902 | 0.0014833 | 0.001129731 | 0.06652 | 0.04649809 |
| set2 | BT4404 | 0.308152 | 0.06191358 | 0.00084112 | 0.000691178 | 0.0198091 | 0.01994387 |
| set2 | BT4408 | 0.058154 | 0.01417994 | 0.00000339 | 0.00000673 | 0.0805222 | 0.05309976 |
| set2 | BT4409 | 0.590416 | 0.1089938 | 0.00228748 | 0.001654588 | 0.841477 | 0.2934712 |
| set2 | BT4413 | 0.0440926 | 0.01104302 | 0.00277265 | 0.00194949 | 0.0465511 | 0.03572046 |
| set2 | BT4419 | 0.0766242 | 0.01802733 | 0.000189426 | 0.000196073 | 0.905808 | 0.3080039 |
| set2 | BT4421 | 0.600064 | 0.1105294 | 4.92768E-05 | 0.0000633 | 0.555132 | 0.218108 |
| set2 | BT4427 | 0.533473 | 0.100041 | 0.0081066 | 0.004985932 | 0.562023 | 0.2203537 |
| set2 | BT4429 | 0.130975 | 0.02891773 | 0.00329168 | 0.002257914 | 0.183796 | 0.09715436 |
| set2 | BT4478 | 0.0398738 | 0.01009667 | 0.00237109 | 0.001704475 | 0.019797 | 0.01994387 |
| set2 | BT4488 | 0.991736 | 0.1685621 | 5.91858E-05 | 0.0000743 | 0.0293891 | 0.0260198 |
| set2 | BT4513 | 0.0440231 | 0.01102926 | 0.00480578 | 0.003161843 | 0.0784965 | 0.05220957 |
| set2 | BT4522 | 0.666537 | 0.120717 | 6.79436E-05 | 0.0000833 | 0.127443 | 0.07371001 |
| set2 | BT4523 | 0.0502804 | 0.01244858 | 0.0000012 | 0.00000275 | 0.540129 | 0.2140566 |
| set2 | BT4534 | 0.967506 | 0.1652486 | 0.000000996 | 0.00000237 | 0.039813 | 0.03218013 |
| set2 | BT4535 | 0.75427 | 0.1342295 | 0.000000164 | 0.000000542 | 0.151848 | 0.08392341 |
| set2 | BT4540 | 0.220875 | 0.04613223 | 4.26E-08 | 0.000000181 | 0.385953 | 0.1685652 |
| set2 | BT4559 | 0.962665 | 0.1645513 | 0.000052746 | 0.0000674 | 0.53323 | 0.2121818 |
| set2 | BT4585 | 0.52087 | 0.09799318 | 0.000101998 | 0.000117007 | 0.0175198 | 0.01835664 |
| set2 | BT4586 | 0.27444 | 0.0558669 | 0.000693761 | 0.000586034 | 0.275183 | 0.1321281 |
| set2 | BT4596 | 0.463151 | 0.0883866 | 6.89344E-05 | 0.0000843 | 0.261869 | 0.1274617 |
| set2 | BT4602 | 0.284073 | 0.05765728 | 0.00000105 | 0.00000248 | 0.136689 | 0.07761353 |
| set2 | BT4606 | 0.362436 | 0.07110263 | 7.59555E-05 | 0.0000913 | 0.252272 | 0.1236095 |
| set2 | BT4607 | 0.0467183 | 0.01162752 | 6.05E-08 | 0.000000241 | 0.330755 | 0.1506564 |
| set2 | BT4631 | 0.264584 | 0.05414006 | 0.0092954 | 0.005602985 | 0.300204 | 0.1402085 |
| set2 | BT4634 | 0.274606 | 0.05587064 | 0.000584999 | 0.000506922 | 0.274412 | 0.1318365 |
| set2 | BT4641 | 0.320232 | 0.0640855 | 0.000211815 | 0.000215735 | 0.00834302 | 0.01131874 |
| set2 | BT4642 | 0.890119 | 0.1540936 | 0.00823301 | 0.005055957 | 0.305903 | 0.142198 |
| set2 | BT4643 | 0.432212 | 0.0831955 | 0.000105326 | 0.00012014 | 0.317034 | 0.145933 |
| set2 | BT4649 | 0.150181 | 0.03273857 | 0.0120678 | 0.007021822 | 0.0141786 | 0.01608426 |
| set2 | BT4653 | 0.458037 | 0.0874989 | 0.0161119 | 0.008952945 | 0.441547 | 0.1859843 |
| set2 | BT4655 | 0.77487 | 0.1371858 | 1.66E-09 | 1.33E-08 | 0.961556 | 0.3203555 |
| set2 | BT4657 | 0.10866 | 0.02451285 | 0.0124934 | 0.007231206 | 0.0347262 | 0.02920336 |
| set2 | BT4669 | 0.782297 | 0.1382743 | 0.00117058 | 0.000921174 | 0.00732274 | 0.01052066 |
| set2 | BT4684 | 0.151436 | 0.03294632 | 0.00014636 | 0.000158561 | 0.0334374 | 0.02843766 |
| set2 | BT4685 | 0.616792 | 0.1131841 | 0.0033667 | 0.002301532 | 0.839877 | 0.2929828 |
| set2 | BT4688 | 0.749438 | 0.1334952 | 0.0154557 | 0.008647391 | 0.678674 | 0.252404 |
| set2 | BT4710 | 0.222686 | 0.04644845 | 0.00745846 | 0.004665691 | 0.0777146 | 0.05186378 |
| set2 | BT4758 | 0.146099 | 0.03194936 | 0.000000214 | 0.000000671 | 0.396798 | 0.1719207 |
| set2 | BT4759 | 0.0411558 | 0.01038308 | 0.00741322 | 0.004644607 | 0.0187488 | 0.01923251 |
| set2 | BT4770 | 0.195688 | 0.04141744 | 3.45E-09 | 0.000000023 | 0.016446 | 0.01773798 |
| set2 | BT4774 | 0.754039 | 0.13422 | 0.0000014 | 0.00000316 | 0.865573 | 0.2987517 |
| set2 | BT4779 | 0.208505 | 0.043771 | 0.000000375 | 0.00000105 | 0.839599 | 0.2929554 |
| set2 | tRNA_58 | 0.188999 | 0.04010264 | 0.010317 | 0.006117219 | 0.527164 | 0.2109689 |
| set3 | BT0001 | 0.625745 | 0.1145905 | 0.0167424 | 0.009251595 | 0.00160074 | 0.003894256 |
| set3 | BT0014 | 0.00623776 | 0.001919936 | 2.56984E-05 | 0.0000368 | 0.00505921 | 0.008272768 |
| set3 | BT0028 | 0.0401225 | 0.01014946 | 0.822585 | 0.2343495 | 0.00101381 | 0.003032208 |
| set3 | BT0030 | 1.15851E-05 | 0.0000117 | 0.00586235 | 0.003754734 | 0.00424483 | 0.007339302 |
| set3 | BT0033 | 0.000040873 | 0.0000294 | 0.00181501 | 0.001356709 | 0.00129331 | 0.003433763 |
| set3 | BT0037 | 0.00038283 | 0.000170843 | 0.0813163 | 0.03514349 | 0.00503006 | 0.00824076 |
| set3 | BT0044 | 0.000310434 | 0.000143968 | 0.019973 | 0.01074672 | 0.00220543 | 0.004680201 |
| set3 | BT0046 | 0.000014841 | 0.0000139 | 0.0489994 | 0.02303068 | 0.00352981 | 0.006501224 |
| set3 | BT0061 | 0.00000592 | 0.00000741 | 0.000014028 | 0.0000221 | 0.000633369 | 0.002256637 |
| set3 | BT0066 | 0.000176511 | 0.0000906 | 0.00494346 | 0.00323656 | 0.00092818 | 0.002880535 |
| set3 | BT0073 | 0.000012963 | 0.0000127 | 0.008005 | 0.004933805 | 0.00132195 | 0.003475991 |
| set3 | BT0096 | 6.28177E-05 | 0.0000405 | 0.0720736 | 0.03179742 | 0.00602379 | 0.009259096 |
| set3 | BT0128 | 0.147063 | 0.03215088 | 0.0433532 | 0.02084734 | 0.000111793 | 0.000948411 |
| set3 | BT0146 | 0.000108092 | 0.0000618 | 0.129893 | 0.05198949 | 0.00266118 | 0.005336608 |
| set3 | BT0171 | 0.09052 | 0.02083672 | 6.14405E-05 | 0.0000764 | 0.00289706 | 0.005685361 |
| set3 | BT0172 | 0.000293076 | 0.000137604 | 0.000216174 | 0.000219067 | 0.00576203 | 0.009030287 |
| set3 | BT0178 | 0.00189685 | 0.000667746 | 0.00219501 | 0.001596305 | 0.00001446 | 0.000402443 |
| set3 | BT0179 | 0.000200411 | 0.000101038 | 0.0142924 | 0.00810921 | 4.25211E-05 | 0.000625552 |
| set3 | BT0180 | 0.000928914 | 0.000364091 | 0.00375833 | 0.002530589 | 1.56786E-05 | 0.000418453 |
| set3 | BT0182 | 0.17596 | 0.0377062 | 0.155702 | 0.0605788 | 0.00420388 | 0.007290237 |
| set3 | BT0192 | 2.94949E-05 | 0.0000231 | 0.187142 | 0.07066528 | 0.00201546 | 0.004448921 |
| set3 | BT0193 | 1.55031E-05 | 0.0000145 | 0.95024 | 0.2613485 | 0.00229553 | 0.004813789 |
| set3 | BT0197 | 0.0316918 | 0.008197645 | 8.44218E-05 | 0.0000996 | 0.000449193 | 0.001855339 |
| set3 | BT0212 | 0.0212464 | 0.005747172 | 1.98899E-05 | 0.0000295 | 0.000111086 | 0.000948054 |
| set3 | BT0221 | 0.000908159 | 0.000357065 | 0.0444739 | 0.0212593 | 0.000328562 | 0.001583476 |
| set3 | BT0224 | 0.00000524 | 0.00000675 | 0.00233179 | 0.001682608 | 0.00188435 | 0.004274861 |
| set3 | BT0227 | 0.179664 | 0.03835848 | 0.0109673 | 0.006459265 | 0.000654965 | 0.002302989 |
| set3 | BT0230 | 0.000110315 | 0.0000626 | 0.0320245 | 0.01608483 | 0.000840416 | 0.002733973 |
| set3 | BT0245 | 0.0399914 | 0.01012306 | 2.86398E-05 | 0.0000402 | 0.000986926 | 0.002986406 |
| set3 | BT0247 | 0.463312 | 0.08839504 | 0.00131501 | 0.001016293 | 0.00175351 | 0.004098743 |
| set3 | BT0254 | 0.854759 | 0.1490289 | 2.03562E-05 | 0.0000301 | 0.00000351 | 0.000183781 |
| set3 | BT0285 | 0.711112 | 0.1276295 | 0.00000133 | 0.00000304 | 0.000156277 | 0.001113602 |
| set3 | BT0287 | 0.00182011 | 0.000644629 | 0.775039 | 0.2234388 | 0.00139808 | 0.003562966 |
| set3 | BT0288 | 0.000212655 | 0.000105888 | 0.253171 | 0.09082803 | 0.00431296 | 0.007423403 |
| set3 | BT0306 | 0.00238906 | 0.000819668 | 0.69138 | 0.2041938 | 0.00000974 | 0.00031786 |
| set3 | BT0318 | 0.368208 | 0.0720855 | 0.488791 | 0.1551735 | 0.00445025 | 0.007596055 |
| set3 | BT0320 | 0.000013897 | 0.0000134 | 0.00164085 | 0.001237244 | 0.0039754 | 0.007047785 |
| set3 | BT0321 | 0.000230903 | 0.000113415 | 3.52526E-05 | 0.0000477 | 0.000967168 | 0.002963934 |
| set3 | BT0322 | 0.12667 | 0.02809015 | 0.00400182 | 0.002670718 | 0.00391388 | 0.006980874 |
| set3 | BT0334 | 6.21253E-05 | 0.0000402 | 0.00322409 | 0.002216585 | 0.0013617 | 0.003515318 |
| set3 | BT0346 | 0.69023 | 0.1244126 | 0.0118767 | 0.006927312 | 0.00588181 | 0.009133231 |
| set3 | BT0363 | 0.00102489 | 0.000395765 | 0.0121419 | 0.007058139 | 0.00354044 | 0.006512631 |
| set3 | BT0383 | 0.000443452 | 0.000192119 | 0.0162748 | 0.00902624 | 0.000444187 | 0.001855339 |
| set3 | BT0408 | 0.487246 | 0.09265775 | 0.980796 | 0.2673994 | 0.000421661 | 0.001794099 |
| set3 | BT0413 | 1.34091E-05 | 0.000013 | 0.12134 | 0.04915248 | 0.00136262 | 0.003515318 |
| set3 | BT0425 | 0.000208719 | 0.000104409 | 0.0256394 | 0.01329814 | 0.00204128 | 0.0044874 |
| set3 | BT0426 | 0.00184776 | 0.00065185 | 0.0491918 | 0.02310313 | 3.72658E-05 | 0.000612068 |
| set3 | BT0478 | 0.0000074 | 0.00000856 | 0.246606 | 0.08886867 | 0.000503617 | 0.00196062 |
| set3 | BT0508 | 1.94961E-05 | 0.0000169 | 0.000299818 | 0.000289732 | 1.98167E-05 | 0.000448892 |
| set3 | BT0510 | 0.34145 | 0.06765195 | 4.35322E-05 | 0.0000572 | 0.00663613 | 0.00982977 |
| set3 | BT0512 | 0.0760994 | 0.017915 | 0.00190437 | 0.001413012 | 0.000148718 | 0.001080723 |
| set3 | BT0513 | 0.502939 | 0.09520806 | 0.00218787 | 0.001593992 | 0.00181989 | 0.004167631 |
| set3 | BT0522 | 0.00160557 | 0.000580045 | 0.00936676 | 0.005637559 | 0.0016092 | 0.003897979 |
| set3 | BT0523 | 0.00255225 | 0.000871706 | 0.126989 | 0.05096226 | 1.44955E-05 | 0.000402443 |
| set3 | BT0524 | 0.420261 | 0.08110134 | 0.980053 | 0.2672963 | 0.000476759 | 0.001906264 |
| set3 | BT0540 | 0.102795 | 0.02335683 | 0.00735547 | 0.00461321 | 0.00380065 | 0.00682035 |
| set3 | BT0548 | 0.00000595 | 0.00000742 | 0.00190611 | 0.001413435 | 0.000297667 | 0.001476861 |
| set3 | BT0550 | 0.00490239 | 0.001551184 | 0.000912971 | 0.000742142 | 0.000988742 | 0.002986406 |
| set3 | BT0555 | 2.46782E-05 | 0.0000201 | 0.07934 | 0.03442099 | 0.000634981 | 0.002256903 |
| set3 | BT0562 | 3.04586E-05 | 0.0000236 | 0.0928793 | 0.03910546 | 0.00557566 | 0.008800664 |
| set3 | BT0568 | 0.0763493 | 0.01796824 | 0.00000635 | 0.0000114 | 0.00146683 | 0.003686965 |
| set3 | BT0571 | 0.00387924 | 0.001258093 | 0.0130367 | 0.007498898 | 0.000406866 | 0.001761789 |
| set3 | BT0573 | 0.000881502 | 0.000348031 | 0.0340972 | 0.01697586 | 0.000613075 | 0.002227585 |
| set3 | BT0577 | 0.000860814 | 0.000341824 | 0.13196 | 0.05269459 | 0.00126881 | 0.003404313 |
| set3 | BT0590 | 4.69259E-05 | 0.0000326 | 0.306682 | 0.1062394 | 0.00241604 | 0.004983029 |
| set3 | BT0609 | 0.00000531 | 0.0000068 | 1.50333E-05 | 0.0000235 | 6.37832E-05 | 0.00072504 |
| set3 | BT0612 | 0.000364335 | 0.00016404 | 0.785917 | 0.2257666 | 0.000371116 | 0.001695937 |
| set3 | BT0616 | 0.0959527 | 0.02196701 | 0.279005 | 0.0982051 | 0.00184811 | 0.004219092 |
| set3 | BT0625 | 0.00656615 | 0.00200957 | 0.00111604 | 0.000884594 | 0.00643303 | 0.009645728 |
| set3 | BT0638 | 0.00279545 | 0.000944634 | 0.0638893 | 0.02868963 | 0.00644398 | 0.009652287 |
| set3 | BT0643 | 0.000054273 | 0.0000363 | 0.0926429 | 0.03903314 | 0.000691047 | 0.002364571 |
| set3 | BT0647 | 0.00222793 | 0.000770674 | 0.000639473 | 0.000546675 | 0.000248607 | 0.001351611 |
| set3 | BT0653 | 0.111738 | 0.02513231 | 5.17083E-05 | 0.0000662 | 0.000162185 | 0.001124178 |
| set3 | BT0661 | 0.220143 | 0.04601431 | 0.000225315 | 0.000226619 | 0.00036598 | 0.001695937 |
| set3 | BT0670 | 0.00130779 | 0.000487354 | 0.247397 | 0.08912713 | 0.00528664 | 0.008501754 |
| set3 | BT0671 | 0.270828 | 0.05522073 | 0.121769 | 0.04926787 | 0.00452653 | 0.007655046 |
| set3 | BT0679 | 0.000098933 | 0.0000577 | 0.000511819 | 0.000451599 | 0.00130879 | 0.003461618 |
| set3 | BT0692 | 0.000209642 | 0.000104802 | 0.000357763 | 0.000336836 | 0.00677472 | 0.009971554 |
| set3 | BT0697 | 0.100287 | 0.0228487 | 0.0141341 | 0.008042059 | 0.0013308 | 0.003475991 |
| set3 | BT0730 | 0.00662927 | 0.00202643 | 0.00803336 | 0.004945922 | 0.000334533 | 0.001589215 |
| set3 | BT0732 | 0.000141832 | 0.0000755 | 0.571054 | 0.1757808 | 0.000908738 | 0.002855197 |
| set3 | BT0734 | 0.0238634 | 0.006373075 | 0.0895468 | 0.03804716 | 0.000394737 | 0.001729678 |
| set3 | BT0747 | 0.0644316 | 0.01551075 | 0.0138795 | 0.007919578 | 0.00098853 | 0.002986406 |
| set3 | BT0751 | 0.168176 | 0.03619163 | 0.0021978 | 0.001596412 | 0.000669054 | 0.002338374 |
| set3 | BT0754 | 0.110669 | 0.02492891 | 2.46133E-05 | 0.0000356 | 9.73912E-05 | 0.00091342 |
| set3 | BT0769 | 0.0885922 | 0.02046141 | 0.000567494 | 0.000494949 | 1.20979E-05 | 0.000359358 |
| set3 | BT0774 | 0.000244148 | 0.000118681 | 0.00104128 | 0.000833542 | 0.000588274 | 0.002148106 |
| set3 | BT0784 | 0.0206136 | 0.005583982 | 0.108464 | 0.0447767 | 0.000255741 | 0.001364989 |
| set3 | BT0789 | 0.00136025 | 0.000504667 | 0.725731 | 0.211904 | 0.00458968 | 0.007735108 |
| set3 | BT0791 | 3.19508E-05 | 0.0000245 | 0.000998806 | 0.000803268 | 6.14977E-05 | 0.000722189 |
| set3 | BT0792 | 0.017564 | 0.004839286 | 0.00000334 | 0.00000665 | 6.07208E-05 | 0.000722189 |
| set3 | BT0808 | 0.000163864 | 0.0000851 | 0.0138791 | 0.007919578 | 0.00126176 | 0.003398462 |
| set3 | BT0820 | 0.00164301 | 0.000591594 | 0.000322447 | 0.000308637 | 4.86955E-05 | 0.000643974 |
| set3 | BT0824 | 0.00317184 | 0.001051991 | 0.00224054 | 0.001624526 | 0.000683743 | 0.002356055 |
| set3 | BT0830 | 0.962703 | 0.1645513 | 1.53865E-05 | 0.000024 | 0.00212446 | 0.004605326 |
| set3 | BT0836 | 0.00389876 | 0.001263735 | 0.925423 | 0.2563911 | 0.00195302 | 0.004356953 |
| set3 | BT0837 | 0.000242576 | 0.000118373 | 0.62686 | 0.188879 | 0.00125847 | 0.003398462 |
| set3 | BT0844 | 0.00014037 | 0.000075 | 0.915667 | 0.2545639 | 0.00324997 | 0.0061478 |
| set3 | BT0845 | 0.000758361 | 0.00030369 | 0.457799 | 0.1473484 | 0.00310783 | 0.005956053 |
| set3 | BT0846 | 9.60806E-05 | 0.0000567 | 0.00662112 | 0.004202829 | 0.00048444 | 0.001927151 |
| set3 | BT0849 | 0.0183301 | 0.005032952 | 0.000917906 | 0.000745652 | 0.000486085 | 0.001928469 |
| set3 | BT0863 | 3.74146E-05 | 0.0000275 | 0.00611334 | 0.003901009 | 3.34483E-05 | 0.00057764 |
| set3 | BT0876 | 0.0739643 | 0.01747214 | 2.71658E-05 | 0.0000386 | 0.00583436 | 0.009080572 |
| set3 | BT0877 | 8.91591E-05 | 0.0000534 | 0.00147566 | 0.001125331 | 0.000334224 | 0.001589215 |
| set3 | BT0887 | 0.00516062 | 0.00162339 | 0.0443028 | 0.02119429 | 0.00647215 | 0.0096846 |
| set3 | BT0903 | 5.84106E-05 | 0.0000382 | 0.000266337 | 0.000261825 | 0.00197969 | 0.004409749 |
| set3 | BT0911 | 2.61211E-05 | 0.0000209 | 0.11131 | 0.04574922 | 0.000379242 | 0.001695937 |
| set3 | BT0929 | 0.0113762 | 0.00327509 | 0.937985 | 0.2588223 | 0.00488281 | 0.00805345 |
| set3 | BT0931 | 0.0703777 | 0.01671883 | 0.000000252 | 0.00000076 | 2.19334E-05 | 0.00046405 |
| set3 | BT0934 | 0.0268628 | 0.007085623 | 0.0050532 | 0.003295904 | 0.000534001 | 0.002015091 |
| set3 | BT0937 | 0.0272263 | 0.007174011 | 0.0825269 | 0.0355441 | 0.00310803 | 0.005956053 |
| set3 | BT0942 | 0.000063908 | 0.0000411 | 0.000370769 | 0.000346918 | 0.00119748 | 0.003310365 |
| set3 | BT0943 | 0.104759 | 0.02373895 | 0.00132489 | 0.001022083 | 0.00636515 | 0.009573284 |
| set3 | BT0947 | 0.000032832 | 0.000025 | 0.000329198 | 0.000314758 | 0.00623221 | 0.009450806 |
| set3 | BT0951 | 8.41391E-05 | 0.0000511 | 0.0268225 | 0.01381674 | 0.00126106 | 0.003398462 |
| set3 | BT0952 | 0.000600469 | 0.000248617 | 0.000421502 | 0.000383182 | 0.000267212 | 0.001379763 |
| set3 | BT0982 | 1.06649E-05 | 0.000011 | 0.000172389 | 0.000181234 | 0.00215091 | 0.00462474 |
| set3 | BT0996 | 2.87887E-05 | 0.0000227 | 0.000269841 | 0.000263717 | 0.0018871 | 0.004274861 |
| set3 | BT0999 | 0.23298 | 0.04835033 | 0.0237874 | 0.01247135 | 0.00128865 | 0.003433763 |
| set3 | BT1002 | 0.0000036 | 0.00000544 | 0.444879 | 0.1439571 | 0.000267479 | 0.001379763 |
| set3 | BT1014 | 0.243308 | 0.05032345 | 3.32511E-05 | 0.0000456 | 0.00091029 | 0.002855197 |
| set3 | BT1022 | 1.12071E-05 | 0.0000114 | 0.00033391 | 0.000318348 | 0.000171904 | 0.001147005 |
| set3 | BT1045 | 0.000000978 | 0.00000256 | 0.0156165 | 0.008725234 | 0.000184668 | 0.001178599 |
| set3 | BT1046 | 0.00831118 | 0.002472965 | 0.00240721 | 0.001725691 | 5.48654E-05 | 0.00068507 |
| set3 | BT1047 | 9.71407E-05 | 0.000057 | 0.0135629 | 0.007771968 | 0.00404595 | 0.007138374 |
| set3 | BT1048 | 1.42301E-05 | 0.0000136 | 0.0258925 | 0.01341716 | 0.000390046 | 0.001719387 |
| set3 | BT1053 | 0.00000572 | 0.00000721 | 0.000133243 | 0.000146987 | 0.000563024 | 0.002084685 |
| set3 | BT1070 | 0.00164074 | 0.000591058 | 0.604324 | 0.1837386 | 0.00317363 | 0.006042324 |
| set3 | BT1071 | 0.00394386 | 0.001276714 | 0.244529 | 0.08819516 | 0.000693713 | 0.002368173 |
| set3 | BT1077 | 0.000319299 | 0.000147179 | 1.23377E-05 | 0.00002 | 0.00337886 | 0.006326394 |
| set3 | BT1080 | 0.000189402 | 0.0000965 | 0.399937 | 0.1321039 | 0.000130219 | 0.001027693 |
| set3 | BT1085 | 0.00854145 | 0.002530214 | 0.000607262 | 0.000524333 | 0.000136413 | 0.001059488 |
| set3 | BT1094 | 0.000767054 | 0.000306685 | 0.000255093 | 0.000252574 | 0.00011391 | 0.000952107 |
| set3 | BT1126 | 1.31899E-05 | 0.0000128 | 0.0777206 | 0.03388054 | 0.00138526 | 0.003548778 |
| set3 | BT1128 | 0.653651 | 0.1187241 | 0.000512182 | 0.000451599 | 0.000351269 | 0.001647394 |
| set3 | BT1134 | 0.000101941 | 0.000059 | 0.0113908 | 0.0066729 | 4.85887E-05 | 0.000643974 |
| set3 | BT1149 | 8.91674E-05 | 0.0000534 | 0.000260658 | 0.00025703 | 4.99841E-05 | 0.000655113 |
| set3 | BT1160 | 0.00000935 | 0.0000101 | 0.0622309 | 0.02803873 | 0.00296034 | 0.005763323 |
| set3 | BT1162 | 7.13079E-05 | 0.0000448 | 0.00395871 | 0.002646334 | 0.000429532 | 0.001822307 |
| set3 | BT1168 | 0.000031864 | 0.0000244 | 0.180759 | 0.06868446 | 0.000585083 | 0.002141782 |
| set3 | BT1171 | 0.0666539 | 0.01598979 | 0.0369711 | 0.01814679 | 0.000512189 | 0.001981583 |
| set3 | BT1204 | 0.00044729 | 0.00019345 | 0.00780124 | 0.004840016 | 0.000540222 | 0.002028139 |
| set3 | BT1217 | 0.0146093 | 0.004110491 | 0.0916406 | 0.03869629 | 0.000205425 | 0.001251234 |
| set3 | BT1219 | 0.0100521 | 0.002928426 | 0.635733 | 0.1906016 | 0.00601538 | 0.00925586 |
| set3 | BT1237 | 3.07622E-05 | 0.0000238 | 0.000163729 | 0.000173793 | 0.00166608 | 0.003995083 |
| set3 | BT1242 | 0.00714266 | 0.002166736 | 0.918479 | 0.254935 | 0.000499822 | 0.001956529 |
| set3 | BT1271 | 0.0214128 | 0.005786979 | 0.898552 | 0.2513096 | 0.00284516 | 0.005604444 |
| set3 | BT1272 | 0.00184674 | 0.00065185 | 0.000259539 | 0.000256136 | 0.0028508 | 0.005604444 |
| set3 | BT1278 | 0.0000054 | 0.00000689 | 3.87553E-05 | 0.0000516 | 0.00000268 | 0.000154091 |
| set3 | BT1281 | 0.931292 | 0.1599771 | 0.000136183 | 0.000149957 | 0.00000509 | 0.000223749 |
| set3 | BT1294 | 0.000212549 | 0.000105888 | 0.872794 | 0.2452982 | 0.00241697 | 0.004983029 |
| set3 | BT1307 | 0.00290444 | 0.000976715 | 0.725574 | 0.211904 | 0.00351643 | 0.006497084 |
| set3 | BT1309 | 0.000508214 | 0.000215973 | 0.00104562 | 0.000835908 | 0.000417187 | 0.001790729 |
| set3 | BT1344 | 3.06396E-05 | 0.0000237 | 0.000939381 | 0.00076105 | 0.00213374 | 0.004612898 |
| set3 | BT1345 | 4.78866E-05 | 0.000033 | 0.000697619 | 0.00058847 | 0.000652204 | 0.002302989 |
| set3 | BT1351 | 0.000032495 | 0.0000248 | 0.0104793 | 0.006197796 | 0.00376113 | 0.006782598 |
| set3 | BT1352 | 6.83725E-05 | 0.0000433 | 0.00260261 | 0.001844967 | 0.00653649 | 0.009735296 |
| set3 | BT1364 | 0.000102486 | 0.0000593 | 0.0110354 | 0.006496206 | 0.00471081 | 0.007859589 |
| set3 | BT1370 | 2.50166E-05 | 0.0000203 | 0.982086 | 0.2675487 | 0.00473649 | 0.00787765 |
| set3 | BT1375 | 1.60449E-05 | 0.0000147 | 0.117665 | 0.04795326 | 0.00340749 | 0.006361529 |
| set3 | BT1377 | 0.360543 | 0.07080467 | 0.000433063 | 0.000392143 | 0.00627259 | 0.009492427 |
| set3 | BT1390 | 0.0308298 | 0.008004767 | 2.17964E-05 | 0.000032 | 0.00579337 | 0.00904702 |
| set3 | BT1404 | 3.06367E-05 | 0.0000237 | 0.00127153 | 0.00098603 | 0.00610592 | 0.009302496 |
| set3 | BT1406 | 0.325203 | 0.06489156 | 0.000833397 | 0.000685764 | 0.00215698 | 0.004629053 |
| set3 | BT1407 | 0.000000772 | 0.00000221 | 0.244296 | 0.0881414 | 5.31211E-05 | 0.000683798 |
| set3 | BT1426 | 0.000664486 | 0.000270972 | 0.00285868 | 0.001998347 | 0.0010989 | 0.003168431 |
| set3 | BT1430 | 0.00155654 | 0.0005653 | 0.00122374 | 0.000958015 | 0.00373232 | 0.006759334 |
| set3 | BT1437 | 0.000402488 | 0.000177729 | 0.0329143 | 0.01647693 | 0.0026592 | 0.005336608 |
| set3 | BT1443 | 0.0393702 | 0.009982512 | 0.0787209 | 0.03419307 | 0.00447921 | 0.007618908 |
| set3 | BT1458 | 0.00197084 | 0.000690261 | 1.83769E-05 | 0.0000278 | 9.83163E-05 | 0.00091342 |
| set3 | BT1460 | 0.166395 | 0.03584913 | 0.00019401 | 0.000200256 | 0.00210899 | 0.004579625 |
| set3 | BT1461 | 0.635012 | 0.1160629 | 0.000000811 | 0.000002 | 0.00011242 | 0.000948411 |
| set3 | BT1485 | 3.95977E-05 | 0.0000288 | 0.964324 | 0.2641065 | 0.000470688 | 0.001906264 |
| set3 | BT1486 | 3.72136E-05 | 0.0000274 | 0.0457701 | 0.02176693 | 0.00114724 | 0.003244808 |
| set3 | BT1509 | 0.00619165 | 0.001907295 | 0.309128 | 0.1070253 | 0.00168403 | 0.004006515 |
| set3 | BT1514 | 0.230823 | 0.04796845 | 0.583516 | 0.1787166 | 0.000106929 | 0.000939899 |
| set3 | BT1528 | 0.102465 | 0.02329947 | 0.136423 | 0.05424365 | 4.59409E-05 | 0.00064255 |
| set3 | BT1563 | 0.144842 | 0.03168363 | 5.29243E-05 | 0.0000675 | 0.00011879 | 0.000968745 |
| set3 | BT1564 | 0.595393 | 0.1097492 | 0.619023 | 0.1867972 | 0.00442214 | 0.007556861 |
| set3 | BT1566 | 0.00000694 | 0.00000819 | 0.000000253 | 0.00000076 | 2.39377E-05 | 0.000474846 |
| set3 | BT1571 | 0.0129176 | 0.003683812 | 0.00832453 | 0.005106967 | 0.000243209 | 0.0013492 |
| set3 | BT1583 | 0.00000429 | 0.00000593 | 0.000024944 | 0.0000359 | 0.000165714 | 0.001126179 |
| set3 | BT1595 | 0.000130455 | 0.0000712 | 0.965588 | 0.2643659 | 0.00240774 | 0.004977982 |
| set3 | BT1600 | 0.000228754 | 0.000112584 | 0.00588385 | 0.003764512 | 0.000873228 | 0.002798751 |
| set3 | BT1605 | 3.10777E-05 | 0.000024 | 0.5006 | 0.1580502 | 0.00281004 | 0.00555918 |
| set3 | BT1613 | 0.0512863 | 0.01265622 | 0.451566 | 0.1458084 | 0.00373185 | 0.006759334 |
| set3 | BT1614 | 0.134819 | 0.02972308 | 0.112599 | 0.0462161 | 0.00636351 | 0.009573284 |
| set3 | BT1620 | 0.685283 | 0.1236977 | 0.594141 | 0.1811441 | 0.000239624 | 0.0013492 |
| set3 | BT1627 | 0.00255999 | 0.000873167 | 0.000298293 | 0.000288489 | 0.00125681 | 0.003398462 |
| set3 | BT1630 | 0.221954 | 0.04632887 | 0.000427877 | 0.000387738 | 0.00125914 | 0.003398462 |
| set3 | BT1633 | 0.357289 | 0.07023854 | 0.183526 | 0.06953899 | 0.000450768 | 0.001855339 |
| set3 | BT1647 | 0.000668771 | 0.000272573 | 0.000308525 | 0.000297195 | 0.0011793 | 0.003291097 |
| set3 | BT1648 | 0.000146028 | 0.0000773 | 0.00000506 | 0.00000952 | 0.000737615 | 0.002483393 |
| set3 | BT1653 | 0.022401 | 0.006016487 | 0.0000082 | 0.0000141 | 0.0021271 | 0.004605326 |
| set3 | BT1678 | 0.00000613 | 0.00000753 | 0.0599735 | 0.02717367 | 0.00233613 | 0.004878023 |
| set3 | BT1684 | 0.0173128 | 0.004782261 | 0.000200355 | 0.000205275 | 0.000233417 | 0.001331073 |
| set3 | BT1718 | 0.440306 | 0.08458141 | 0.0018197 | 0.001359374 | 0.00265589 | 0.005336608 |
| set3 | BT1721 | 0.00000648 | 0.00000779 | 9.08863E-05 | 0.000105869 | 0.00044196 | 0.001855339 |
| set3 | BT1728 | 0.519423 | 0.09779388 | 0.9268 | 0.2567137 | 0.000374691 | 0.001695937 |
| set3 | BT1730 | 9.46266E-05 | 0.000056 | 0.0157483 | 0.008778571 | 0.00448681 | 0.007623002 |
| set3 | BT1733 | 4.89781E-05 | 0.0000336 | 0.286394 | 0.10045 | 0.00480002 | 0.007934731 |
| set3 | BT1734 | 0.027395 | 0.007214414 | 0.31423 | 0.1084808 | 0.00109655 | 0.003168431 |
| set3 | BT1745 | 0.00000135 | 0.00000305 | 0.00000225 | 0.00000472 | 2.93984E-05 | 0.000532771 |
| set3 | BT1757 | 8.10652E-05 | 0.0000497 | 0.805637 | 0.230227 | 0.00598274 | 0.009224977 |
| set3 | BT1758 | 0.0000088 | 0.00000964 | 0.964422 | 0.2641065 | 0.00190908 | 0.004311346 |
| set3 | BT1772 | 0.0166039 | 0.004601559 | 0.0512953 | 0.02383161 | 0.00653684 | 0.009735296 |
| set3 | BT1774 | 0.00620914 | 0.001911904 | 0.0404951 | 0.01963712 | 0.00357104 | 0.006547011 |
| set3 | BT1782 | 0.0203019 | 0.005511381 | 0.000150862 | 0.000162564 | 0.00363951 | 0.00662842 |
| set3 | BT1794 | 0.00000073 | 0.00000215 | 0.00000741 | 0.0000129 | 0.000135585 | 0.001058659 |
| set3 | BT1796 | 0.000619172 | 0.000254966 | 0.00000643 | 0.0000115 | 0.00176256 | 0.004106358 |
| set3 | BT1810 | 0.00647078 | 0.001985199 | 0.00338922 | 0.002314307 | 0.00340844 | 0.006361529 |
| set3 | BT1819 | 0.0283782 | 0.007434536 | 0.0112569 | 0.006610489 | 0.00527868 | 0.008501754 |
| set3 | BT1824 | 0.0143159 | 0.004036944 | 0.00384066 | 0.002577404 | 0.00170171 | 0.004035497 |
| set3 | BT1825 | 0.696312 | 0.1253895 | 7.79075E-05 | 0.000093 | 0.00172681 | 0.004055724 |
| set3 | BT1838 | 0.000984572 | 0.000382735 | 0.779694 | 0.2242456 | 0.000815368 | 0.002677616 |
| set3 | BT1839 | 4.69898E-05 | 0.0000326 | 0.00237105 | 0.001704475 | 0.00657436 | 0.009761484 |
| set3 | BT1851 | 0.00000259 | 0.00000431 | 0.00000519 | 0.00000971 | 6.35377E-05 | 0.00072504 |
| set3 | BT1862 | 0.0117432 | 0.003371767 | 0.000568939 | 0.000495851 | 0.00216158 | 0.004632153 |
| set3 | BT1887 | 0.187111 | 0.03973545 | 0.144416 | 0.05693451 | 0.000402283 | 0.001757497 |
| set3 | BT1907 | 0.000280069 | 0.000132897 | 1.83441E-05 | 0.0000278 | 0.00121856 | 0.003354401 |
| set3 | BT1910 | 2.81112E-05 | 0.0000222 | 0.017887 | 0.00980781 | 0.00163112 | 0.003944567 |
| set3 | BT1913 | 0.00159625 | 0.000577505 | 0.00000257 | 0.00000528 | 0.000847471 | 0.00274013 |
| set3 | BT1920 | 7.53705E-05 | 0.0000468 | 9.11295E-05 | 0.000105948 | 0.000270993 | 0.001379763 |
| set3 | BT1922 | 0.000259089 | 0.000124822 | 0.0414532 | 0.0200454 | 0.00439961 | 0.007542244 |
| set3 | BT1939 | 0.00000469 | 0.00000632 | 0.0055682 | 0.003591089 | 0.000453044 | 0.001857631 |
| set3 | BT1940 | 0.000214391 | 0.000106543 | 4.47E-08 | 0.00000019 | 0.00000104 | 0.000116079 |
| set3 | BT1966 | 0.00000405 | 0.00000575 | 0.000845315 | 0.000693681 | 0.000221129 | 0.001303612 |
| set3 | BT1989 | 0.917801 | 0.1582463 | 0.57187 | 0.1758632 | 0.00122186 | 0.003354401 |
| set3 | BT1991 | 0.00267049 | 0.000907993 | 4.07682E-05 | 0.000054 | 0.000139898 | 0.001068886 |
| set3 | BT2006 | 0.00000267 | 0.00000439 | 0.00489926 | 0.003211103 | 0.000468517 | 0.001905111 |
| set3 | BT2007 | 2.13129E-05 | 0.0000181 | 0.0247403 | 0.01289817 | 0.000404664 | 0.001759688 |
| set3 | BT2008 | 4.23236E-05 | 0.0000301 | 0.100481 | 0.0419261 | 0.0031294 | 0.00597361 |
| set3 | BT2010 | 6.30504E-05 | 0.0000406 | 0.601663 | 0.1830678 | 0.00284449 | 0.005604444 |
| set3 | BT2011 | 0.00113151 | 0.000431217 | 0.301759 | 0.1047442 | 0.0043137 | 0.007423403 |
| set3 | BT2042 | 0.000000444 | 0.00000158 | 0.00000309 | 0.00000622 | 4.86764E-05 | 0.000643974 |
| set3 | BT2047 | 0.0000038 | 0.00000554 | 0.0095052 | 0.005706665 | 0.000279733 | 0.001411084 |
| set3 | BT2069 | 0.0461768 | 0.01150789 | 2.83202E-05 | 0.0000399 | 3.31556E-05 | 0.00057764 |
| set3 | BT2098 | 0.000000268 | 0.00000126 | 0.000000165 | 0.000000543 | 2.05893E-05 | 0.000454921 |
| set3 | BT2113 | 0.000029271 | 0.000023 | 0.0259024 | 0.01341716 | 0.000448693 | 0.001855339 |
| set3 | BT2119 | 0.150018 | 0.03272129 | 0.00585881 | 0.003754457 | 0.000216708 | 0.001298142 |
| set3 | BT2132 | 0.534241 | 0.1001602 | 6.16333E-05 | 0.0000766 | 8.75885E-05 | 0.000863083 |
| set3 | BT2134 | 0.000133118 | 0.0000723 | 0.0239907 | 0.01256158 | 0.0037685 | 0.00678755 |
| set3 | BT2135 | 0.00000709 | 0.00000834 | 0.00000819 | 0.0000141 | 0.00208182 | 0.004534041 |
| set3 | BT2139 | 3.99177E-05 | 0.000029 | 7.17227E-05 | 0.000087 | 0.000827814 | 0.002709622 |
| set3 | BT2141 | 0.00000418 | 0.00000585 | 7.08875E-05 | 0.0000862 | 0.000103633 | 0.0009299 |
| set3 | BT2148 | 0.00000749 | 0.00000864 | 0.0028766 | 0.002009711 | 4.07066E-05 | 0.00061981 |
| set3 | BT2153 | 0.010854 | 0.003134291 | 0.157615 | 0.06119932 | 0.00312283 | 0.00597034 |
| set3 | BT2155 | 0.0679588 | 0.01624105 | 0.159779 | 0.06185472 | 0.00417677 | 0.00727609 |
| set3 | BT2160 | 0.0144279 | 0.004067011 | 0.00689679 | 0.004361769 | 0.000551837 | 0.002061201 |
| set3 | BT2175 | 0.128332 | 0.02840046 | 2.42857E-05 | 0.0000351 | 0.00323335 | 0.006132166 |
| set3 | BT2183 | 0.00000214 | 0.00000391 | 0.0249497 | 0.01296816 | 0.000253621 | 0.001364989 |
| set3 | BT2184 | 0.123456 | 0.02744174 | 3.46865E-05 | 0.0000471 | 3.68886E-05 | 0.000612068 |
| set3 | BT2196 | 0.0377002 | 0.009587983 | 6.91997E-05 | 0.0000844 | 0.000184326 | 0.001178599 |
| set3 | BT2200 | 0.152572 | 0.03316367 | 0.633674 | 0.1901577 | 0.00181416 | 0.004161 |
| set3 | BT2205 | 0.0462843 | 0.01153088 | 0.0003996 | 0.000367636 | 0.00220632 | 0.004680201 |
| set3 | BT2216 | 0.00024883 | 0.000120262 | 0.000035907 | 0.0000484 | 0.000628311 | 0.002255036 |
| set3 | BT2219 | 2.20959E-05 | 0.0000185 | 0.00000953 | 0.0000159 | 7.71309E-05 | 0.000797338 |
| set3 | BT2224 | 4.21851E-05 | 0.0000301 | 0.00000063 | 0.00000162 | 0.0000059 | 0.00023406 |
| set3 | BT2239 | 0.00000352 | 0.00000534 | 0.00183288 | 0.001368371 | 0.000247364 | 0.001351611 |
| set3 | BT2250 | 0.000448443 | 0.000193837 | 0.000181202 | 0.000189509 | 0.00363389 | 0.006626406 |
| set3 | BT2258 | 0.000136341 | 0.0000734 | 0.000727773 | 0.000610496 | 0.000271644 | 0.001379763 |
| set3 | BT2275 | 5.57988E-05 | 0.0000369 | 0.00185924 | 0.001383777 | 0.000138438 | 0.001063957 |
| set3 | BT2283 | 0.000305292 | 0.000142018 | 0.0736688 | 0.03245377 | 0.00609064 | 0.009302496 |
| set3 | BT2285 | 7.08201E-05 | 0.0000446 | 0.000166125 | 0.000175565 | 0.000102268 | 0.0009299 |
| set3 | BT2286 | 0.00028495 | 0.000134624 | 0.0166029 | 0.009182911 | 0.00257131 | 0.005220574 |
| set3 | BT2291 | 0.00184716 | 0.00065185 | 0.00000533 | 0.00000992 | 7.80288E-05 | 0.000800979 |
| set3 | BT2304 | 0.460469 | 0.08791911 | 0.00000425 | 0.0000082 | 0.000841842 | 0.002733973 |
| set3 | BT2313 | 9.72876E-05 | 0.000057 | 0.765279 | 0.2210471 | 0.00131338 | 0.00346751 |
| set3 | BT2324 | 5.79861E-05 | 0.0000381 | 8.65638E-05 | 0.000101717 | 0.00118398 | 0.003292884 |
| set3 | BT2330 | 0.000214243 | 0.000106539 | 0.00820172 | 0.005041868 | 0.000040957 | 0.00061981 |
| set3 | BT2331 | 0.126958 | 0.02813753 | 0.00219059 | 0.001594051 | 0.0015404 | 0.003813124 |
| set3 | BT2353 | 0.000124289 | 0.0000685 | 0.0810967 | 0.03507368 | 0.0054201 | 0.008638728 |
| set3 | BT2355 | 0.00130763 | 0.000487354 | 0.000468888 | 0.00041893 | 0.00068954 | 0.002364571 |
| set3 | BT2364 | 0.547442 | 0.1021543 | 6.82536E-05 | 0.0000836 | 0.00000111 | 0.000116079 |
| set3 | BT2365 | 0.205761 | 0.04330718 | 0.000215375 | 0.000218624 | 0.00176516 | 0.004106358 |
| set3 | BT2370 | 0.0951648 | 0.02181302 | 0.000791516 | 0.000654871 | 0.00611744 | 0.009305603 |
| set3 | BT2373 | 0.0192968 | 0.005263066 | 0.0003107 | 0.000298823 | 0.00334746 | 0.006291678 |
| set3 | BT2374 | 9.62725E-05 | 0.0000567 | 0.00000259 | 0.00000532 | 0.000594799 | 0.002166543 |
| set3 | BT2376 | 5.02407E-05 | 0.0000342 | 0.000170745 | 0.000179662 | 0.00357251 | 0.006547011 |
| set3 | BT2377 | 0.000585088 | 0.00024278 | 0.107046 | 0.04429813 | 0.00578504 | 0.00904532 |
| set3 | BT2378 | 0.0024878 | 0.000850856 | 0.0002231 | 0.000224953 | 0.0017068 | 0.004041039 |
| set3 | BT2381 | 0.00044173 | 0.000191795 | 3.70397E-05 | 0.0000497 | 0.00134689 | 0.003499337 |
| set3 | BT2436 | 0.0342757 | 0.008793887 | 0.306421 | 0.1061794 | 0.00028308 | 0.001423077 |
| set3 | BT2451 | 0.000167673 | 0.0000867 | 0.0334439 | 0.01671434 | 0.00477908 | 0.007909022 |
| set3 | BT2458 | 0.0284687 | 0.007453085 | 0.0540501 | 0.02490078 | 0.000310941 | 0.001520003 |
| set3 | BT2459 | 0.624722 | 0.1144862 | 0.00000456 | 0.00000875 | 0.000489971 | 0.001938646 |
| set3 | BT2460 | 0.351858 | 0.06933295 | 0.0981047 | 0.04101959 | 3.75267E-05 | 0.000612068 |
| set3 | BT2492 | 0.000000768 | 0.00000221 | 1.84189E-05 | 0.0000278 | 0.000016516 | 0.000428224 |
| set3 | BT2501 | 0.000013841 | 0.0000134 | 0.00000167 | 0.00000369 | 0.000247535 | 0.001351611 |
| set3 | BT2516 | 0.41918 | 0.080934 | 0.629172 | 0.1894338 | 0.00551752 | 0.00873708 |
| set3 | BT2529 | 8.24558E-05 | 0.0000503 | 0.00200943 | 0.001481128 | 0.00420652 | 0.007290237 |
| set3 | BT2538 | 0.00994919 | 0.002906276 | 0.0457897 | 0.02176768 | 0.0013907 | 0.003550322 |
| set3 | BT2540 | 0.0299924 | 0.007808772 | 0.00579892 | 0.00372398 | 0.00600746 | 0.009253374 |
| set3 | BT2542 | 2.40232E-05 | 0.0000197 | 0.208133 | 0.07700285 | 0.00158056 | 0.003873346 |
| set3 | BT2549 | 0.835556 | 0.1462874 | 0.159077 | 0.0616481 | 0.0010975 | 0.003168431 |
| set3 | BT2571 | 0.0001606 | 0.0000837 | 0.0606957 | 0.0274597 | 0.00441499 | 0.007553436 |
| set3 | BT2578 | 8.95509E-05 | 0.0000536 | 1.38049E-05 | 0.0000219 | 1.22103E-05 | 0.000359358 |
| set3 | BT2586 | 0.00695103 | 0.002116238 | 0.0627106 | 0.0282438 | 0.000688781 | 0.002364571 |
| set3 | BT2593 | 5.18359E-05 | 0.000035 | 0.00897264 | 0.005432834 | 0.000616302 | 0.002233781 |
| set3 | BT2599 | 0.00011554 | 0.0000647 | 0.132518 | 0.05286498 | 0.000546648 | 0.002047028 |
| set3 | BT2610 | 7.38417E-05 | 0.0000462 | 0.09086 | 0.0384833 | 0.0010934 | 0.003168431 |
| set3 | BT2627 | 0.000247865 | 0.000120018 | 0.438275 | 0.1422392 | 0.00418792 | 0.00727697 |
| set3 | BT2628 | 0.000161559 | 0.0000841 | 1.95163E-05 | 0.0000291 | 0.00556982 | 0.008800664 |
| set3 | BT2643 | 1.31438E-05 | 0.0000128 | 0.152602 | 0.05953993 | 0.00152926 | 0.003798359 |
| set3 | BT2650 | 0.00327386 | 0.001080619 | 0.764709 | 0.221041 | 0.00129627 | 0.003434692 |
| set3 | BT2654 | 0.20473 | 0.04312616 | 0.0151974 | 0.008518656 | 0.00104827 | 0.003089911 |
| set3 | BT2666 | 0.482739 | 0.09186992 | 0.000142901 | 0.000155792 | 0.000060809 | 0.000722189 |
| set3 | BT2676 | 0.000109695 | 0.0000625 | 0.131626 | 0.05262978 | 0.00195053 | 0.004356953 |
| set3 | BT2679 | 2.19269E-05 | 0.0000184 | 0.00012525 | 0.000139059 | 3.84981E-05 | 0.000614262 |
| set3 | BT2686 | 0.00000485 | 0.00000646 | 0.018389 | 0.01001488 | 0.000385353 | 0.001708964 |
| set3 | BT2771 | 0.00000522 | 0.00000674 | 0.786255 | 0.22581 | 0.00104145 | 0.003086115 |
| set3 | BT2772 | 0.011368 | 0.003273974 | 0.0868881 | 0.0371659 | 0.00110081 | 0.003168431 |
| set3 | BT2773 | 0.000359751 | 0.000162362 | 0.000887138 | 0.000721629 | 0.00180632 | 0.004149502 |
| set3 | BT2774 | 0.00321614 | 0.001064352 | 0.354981 | 0.1197764 | 0.00405397 | 0.007143938 |
| set3 | BT2777 | 0.000041082 | 0.0000295 | 3.88293E-05 | 0.0000517 | 0.000720185 | 0.002435883 |
| set3 | BT2781 | 0.00106965 | 0.000411162 | 0.000166632 | 0.000175947 | 0.000375821 | 0.001695937 |
| set3 | BT2802 | 6.60487E-05 | 0.0000422 | 2.98269E-05 | 0.0000416 | 0.00125785 | 0.003398462 |
| set3 | BT2806 | 0.305846 | 0.06153193 | 0.00000118 | 0.00000272 | 7.25759E-05 | 0.000782832 |
| set3 | BT2807 | 0.526232 | 0.09878097 | 1.77217E-05 | 0.000027 | 0.00136052 | 0.003515318 |
| set3 | BT2828 | 0.000941879 | 0.000368409 | 0.000000273 | 0.000000806 | 1.75033E-05 | 0.000428224 |
| set3 | BT2831 | 9.18448E-05 | 0.0000546 | 0.00110868 | 0.00088001 | 0.00086452 | 0.002789111 |
| set3 | BT2841 | 0.000194874 | 0.0000988 | 0.00885769 | 0.005374008 | 0.00593843 | 0.009175931 |
| set3 | BT2850 | 0.00000261 | 0.00000431 | 0.00294489 | 0.002046767 | 0.000301185 | 0.001488603 |
| set3 | BT2881 | 0.0512068 | 0.01264073 | 0.1681 | 0.0646265 | 0.00630388 | 0.00952015 |
| set3 | BT2887 | 8.50029E-05 | 0.0000513 | 0.00000778 | 0.0000135 | 0.000251447 | 0.001362007 |
| set3 | BT2903 | 4.00613E-05 | 0.000029 | 0.000640113 | 0.000546835 | 0.000021729 | 0.00046405 |
| set3 | BT2904 | 0.96826 | 0.1653144 | 0.0604346 | 0.02735181 | 0.00144191 | 0.003646579 |
| set3 | BT2923 | 0.000009 | 0.0000098 | 0.000811738 | 0.000670683 | 0.00341496 | 0.006361529 |
| set3 | BT2946 | 0.0153942 | 0.00430413 | 0.0884115 | 0.03763097 | 0.00231573 | 0.004842314 |
| set3 | BT2954 | 0.000121715 | 0.0000673 | 0.00768961 | 0.004787973 | 0.000889158 | 0.002821065 |
| set3 | BT2976 | 0.000149556 | 0.0000789 | 0.613673 | 0.1856 | 0.00244213 | 0.005013775 |
| set3 | BT3000 | 3.06634E-05 | 0.0000237 | 0.571311 | 0.1757808 | 0.00580276 | 0.009052053 |
| set3 | BT3002 | 0.892692 | 0.1544622 | 0.00000465 | 0.00000887 | 0.00150653 | 0.003760993 |
| set3 | BT3003 | 0.00160408 | 0.000579784 | 0.000571657 | 0.000497861 | 0.000171149 | 0.001147005 |
| set3 | BT3017 | 1.88837E-05 | 0.0000166 | 0.350337 | 0.1185737 | 0.00201301 | 0.004448921 |
| set3 | BT3019 | 0.00000907 | 0.00000986 | 0.0374545 | 0.01833192 | 0.000517022 | 0.001981583 |
| set3 | BT3037 | 0.00000333 | 0.00000514 | 0.030226 | 0.01532166 | 0.000256586 | 0.001364989 |
| set3 | BT3038 | 0.00000176 | 0.00000355 | 0.00781812 | 0.004845511 | 0.000102771 | 0.0009299 |
| set3 | BT3049 | 5.81216E-05 | 0.0000381 | 0.00000935 | 0.0000157 | 0.00188694 | 0.004274861 |
| set3 | BT3050 | 0.00000589 | 0.0000074 | 0.00583655 | 0.003744165 | 0.000157914 | 0.001118985 |
| set3 | BT3057 | 0.000141659 | 0.0000755 | 0.000000169 | 0.000000552 | 1.22404E-05 | 0.000359358 |
| set3 | BT3062 | 0.00262709 | 0.00089404 | 0.216995 | 0.07989003 | 0.00172958 | 0.004055724 |
| set3 | BT3064 | 1.60675E-05 | 0.0000147 | 3.72402E-05 | 0.0000499 | 0.000915689 | 0.002859909 |
| set3 | BT3065 | 0.24856 | 0.05123245 | 0.0435968 | 0.02092283 | 0.00168392 | 0.004006515 |
| set3 | BT3078 | 0.454374 | 0.08686494 | 0.000024246 | 0.0000351 | 0.000187038 | 0.001188557 |
| set3 | BT3082 | 0.0011858 | 0.000448071 | 0.593259 | 0.1809665 | 0.00538893 | 0.00860774 |
| set3 | BT3105 | 0.334912 | 0.06660781 | 0.000214877 | 0.000218302 | 0.0022755 | 0.004778612 |
| set3 | BT3131 | 8.03713E-05 | 0.0000495 | 0.0223875 | 0.01184875 | 0.000165253 | 0.001126179 |
| set3 | BT3136 | 0.00000625 | 0.0000076 | 0.141388 | 0.05594159 | 3.91865E-05 | 0.000618523 |
| set3 | BT3143 | 0.000033941 | 0.0000256 | 0.0875473 | 0.03734209 | 0.00524821 | 0.00847519 |
| set3 | BT3154 | 0.00485912 | 0.001541359 | 5.62332E-05 | 0.0000711 | 4.08886E-05 | 0.00061981 |
| set3 | BT3156 | 0.00936129 | 0.002752604 | 1.23461E-05 | 0.00002 | 0.000405182 | 0.001759688 |
| set3 | BT3158 | 0.567655 | 0.105484 | 0.000352101 | 0.000332282 | 0.00022987 | 0.001324944 |
| set3 | BT3169 | 0.0279053 | 0.007326175 | 0.0382831 | 0.01869203 | 0.00199924 | 0.004431522 |
| set3 | BT3171 | 0.000213704 | 0.000106341 | 0.00000567 | 0.0000104 | 0.00121086 | 0.003341061 |
| set3 | BT3190 | 0.0290571 | 0.007591373 | 1.23187E-05 | 0.00002 | 0.0020956 | 0.004557291 |
| set3 | BT3191 | 0.0524017 | 0.01290203 | 0.00002974 | 0.0000415 | 0.00142421 | 0.003610751 |
| set3 | BT3193 | 0.0522731 | 0.01287875 | 0.796025 | 0.2280196 | 0.00546507 | 0.008672736 |
| set3 | BT3196 | 0.0000132 | 0.0000128 | 0.0155721 | 0.008708422 | 0.00137685 | 0.003534503 |
| set3 | BT3197 | 0.0111288 | 0.003209971 | 4.84997E-05 | 0.0000625 | 0.00183782 | 0.004202136 |
| set3 | BT3209 | 0.00000957 | 0.0000102 | 0.0140168 | 0.007994144 | 0.000915587 | 0.002859909 |
| set3 | BT3227 | 0.0797361 | 0.01868396 | 0.632203 | 0.1900622 | 0.00460539 | 0.007743803 |
| set3 | BT3260 | 3.96407E-05 | 0.0000288 | 0.751807 | 0.2177807 | 0.00536943 | 0.008597049 |
| set3 | BT3265 | 0.00000679 | 0.00000804 | 0.00415042 | 0.002759219 | 0.0007721 | 0.002575864 |
| set3 | BT3278 | 0.00214465 | 0.000744641 | 0.00285137 | 0.001997862 | 0.000498361 | 0.001956026 |
| set3 | BT3279 | 0.00000131 | 0.00000299 | 0.00836665 | 0.005127599 | 0.000307066 | 0.001507519 |
| set3 | BT3280 | 0.00000766 | 0.0000088 | 0.0053751 | 0.003483291 | 0.00256484 | 0.005220574 |
| set3 | BT3285 | 0.00839909 | 0.002495858 | 0.000109548 | 0.000124252 | 9.72385E-05 | 0.00091342 |
| set3 | BT3289 | 0.00000247 | 0.00000416 | 0.00206248 | 0.001515434 | 0.000140956 | 0.001068886 |
| set3 | BT3292 | 8.06742E-05 | 0.0000496 | 0.765079 | 0.2210471 | 0.00470314 | 0.007859589 |
| set3 | BT3308 | 1.03514E-05 | 0.0000108 | 0.648064 | 0.1935779 | 0.000967087 | 0.002963934 |
| set3 | BT3322 | 6.28296E-05 | 0.0000405 | 1.71879E-05 | 0.0000263 | 0.000711634 | 0.00241252 |
| set3 | BT3346 | 0.00000369 | 0.00000551 | 3.83051E-05 | 0.0000511 | 0.00114653 | 0.003244808 |
| set3 | BT3354 | 4.78358E-05 | 0.000033 | 0.000150342 | 0.000162293 | 0.00022106 | 0.001303612 |
| set3 | BT3355 | 0.00000955 | 0.0000102 | 0.0214703 | 0.0114453 | 0.00117511 | 0.00328565 |
| set3 | BT3369 | 0.000192012 | 0.0000975 | 0.000186476 | 0.000193683 | 0.00123047 | 0.003369833 |
| set3 | BT3370 | 0.00160125 | 0.000579037 | 0.00000565 | 0.0000104 | 0.000203125 | 0.001242379 |
| set3 | BT3371 | 0.0113537 | 0.0032711 | 0.000154962 | 0.000166093 | 0.000243062 | 0.0013492 |
| set3 | BT3376 | 0.00000695 | 0.00000819 | 0.0747999 | 0.03283702 | 0.000931335 | 0.002884229 |
| set3 | BT3396 | 0.00000337 | 0.00000518 | 0.000125082 | 0.000139 | 0.000265436 | 0.001379763 |
| set3 | BT3397 | 0.00000366 | 0.0000055 | 0.000196512 | 0.000201851 | 0.000287162 | 0.001438671 |
| set3 | BT3399 | 0.246611 | 0.05090003 | 0.0000073 | 0.0000128 | 0.00276562 | 0.005515905 |
| set3 | BT3402 | 0.0897492 | 0.02067815 | 0.131444 | 0.05257544 | 0.00313402 | 0.00597466 |
| set3 | BT3414 | 0.179249 | 0.03829575 | 0.00000583 | 0.0000107 | 0.00119597 | 0.003310365 |
| set3 | BT3429 | 0.000113706 | 0.000064 | 0.170519 | 0.06538999 | 4.62346E-05 | 0.00064255 |
| set3 | BT3430 | 0.0976024 | 0.02231768 | 0.422879 | 0.138208 | 0.00466444 | 0.007816234 |
| set3 | BT3447 | 1.97794E-05 | 0.0000171 | 0.0343454 | 0.01708009 | 0.00350569 | 0.006493145 |
| set3 | BT3458 | 0.0116282 | 0.003343822 | 0.00000971 | 0.0000162 | 0.000442285 | 0.001855339 |
| set3 | BT3460 | 0.00000112 | 0.00000273 | 1.34465E-05 | 0.0000215 | 0.000000274 | 0.0000534 |
| set3 | BT3467 | 4.35042E-05 | 0.0000308 | 0.451457 | 0.1458084 | 0.00638454 | 0.009592618 |
| set3 | BT3485 | 0.0328016 | 0.00845006 | 0.312112 | 0.1078112 | 0.00297096 | 0.005768689 |
| set3 | BT3490 | 0.00027065 | 0.000129485 | 2.69242E-05 | 0.0000383 | 0.00649777 | 0.009713035 |
| set3 | BT3499 | 0.000110865 | 0.0000628 | 3.01252E-05 | 0.0000419 | 0.00216679 | 0.004636549 |
| set3 | BT3504 | 0.926731 | 0.1593491 | 0.000000153 | 0.000000514 | 1.88125E-05 | 0.000445407 |
| set3 | BT3505 | 0.172334 | 0.03703379 | 0.000276257 | 0.0002689 | 0.00179967 | 0.004140705 |
| set3 | BT3507 | 0.0315224 | 0.008162662 | 0.110755 | 0.0455366 | 0.00207158 | 0.004524179 |
| set3 | BT3512 | 5.01341E-05 | 0.0000342 | 0.00000612 | 0.0000111 | 0.000419649 | 0.001790729 |
| set3 | BT3526 | 0.000000276 | 0.00000127 | 0.000865271 | 0.000707175 | 2.92723E-05 | 0.000532771 |
| set3 | BT3528 | 1.97318E-05 | 0.0000171 | 0.107303 | 0.0443589 | 0.00179744 | 0.004140705 |
| set3 | BT3536 | 0.00492744 | 0.001558458 | 0.825873 | 0.2350645 | 0.000626095 | 0.002255036 |
| set3 | BT3555 | 0.00232395 | 0.000799868 | 0.000990416 | 0.000797051 | 0.00203014 | 0.004474597 |
| set3 | BT3558 | 1.06692E-05 | 0.000011 | 0.11289 | 0.04630408 | 0.00418895 | 0.00727697 |
| set3 | BT3565 | 0.0790998 | 0.01854635 | 0.147716 | 0.05800832 | 0.000267819 | 0.001379763 |
| set3 | BT3569 | 3.67933E-05 | 0.0000272 | 0.351233 | 0.1187772 | 0.00571416 | 0.008990273 |
| set3 | BT3572 | 0.0726808 | 0.01720654 | 0.185527 | 0.07016512 | 0.00603788 | 0.009271045 |
| set3 | BT3574 | 0.0133871 | 0.003803372 | 0.277869 | 0.0979148 | 0.000927972 | 0.002880535 |
| set3 | BT3591 | 7.07583E-05 | 0.0000446 | 0.39559 | 0.1308111 | 0.000233948 | 0.001331073 |
| set3 | BT3593 | 0.000919599 | 0.000360986 | 0.0356728 | 0.01762412 | 0.000268885 | 0.001379763 |
| set3 | BT3601 | 0.000491166 | 0.000209907 | 2.57519E-05 | 0.0000368 | 0.00164114 | 0.003955765 |
| set3 | BT3603 | 0.0139315 | 0.003937348 | 0.029389 | 0.01496016 | 0.000682078 | 0.002355848 |
| set3 | BT3638 | 0.886308 | 0.1535743 | 0.636709 | 0.1907522 | 0.00507134 | 0.008272768 |
| set3 | BT3639 | 0.089685 | 0.02066965 | 0.000523226 | 0.000460664 | 0.00171658 | 0.004051126 |
| set3 | BT3660 | 0.00000509 | 0.00000668 | 0.020473 | 0.01099127 | 0.00000055 | 0.0000734 |
| set3 | BT3662 | 0.0141529 | 0.003996936 | 0.00448855 | 0.002964432 | 0.00126064 | 0.003398462 |
| set3 | BT3665 | 0.000529448 | 0.000223991 | 9.26129E-05 | 0.000107466 | 0.000195163 | 0.001213913 |
| set3 | BT3666 | 0.00664321 | 0.002029871 | 0.478295 | 0.1526034 | 0.00303053 | 0.005845694 |
| set3 | BT3691 | 0.00000917 | 0.00000996 | 0.0289394 | 0.0147624 | 0.00158999 | 0.003883489 |
| set3 | BT3693 | 0.00155185 | 0.000563867 | 0.659618 | 0.1965916 | 0.0065103 | 0.009721865 |
| set3 | BT3694 | 9.67281E-05 | 0.0000569 | 0.105395 | 0.04373477 | 0.00574683 | 0.009030287 |
| set3 | BT3717 | 0.000610614 | 0.000252128 | 0.0972725 | 0.04072802 | 0.000477589 | 0.001906264 |
| set3 | BT3727 | 0.000119572 | 0.0000664 | 0.04447 | 0.0212593 | 0.0044641 | 0.007610845 |
| set3 | BT3738 | 0.0569749 | 0.01390588 | 1.96482E-05 | 0.0000292 | 0.00100895 | 0.003028746 |
| set3 | BT3739 | 0.00000211 | 0.00000389 | 0.678901 | 0.2009997 | 0.000473693 | 0.001906264 |
| set3 | BT3753 | 5.19125E-05 | 0.000035 | 0.256939 | 0.09185248 | 0.000162675 | 0.001124178 |
| set3 | BT3754 | 0.127868 | 0.02832262 | 0.0137307 | 0.007853221 | 0.000171691 | 0.001147005 |
| set3 | BT3755 | 0.975354 | 0.1663755 | 8.04804E-05 | 0.0000954 | 0.00264182 | 0.005326893 |
| set3 | BT3778 | 0.000377925 | 0.000168953 | 0.000145444 | 0.000157994 | 0.00344038 | 0.00640076 |
| set3 | BT3787 | 0.000405507 | 0.000178854 | 8.88302E-05 | 0.000103875 | 0.00373595 | 0.006759334 |
| set3 | BT3796 | 1.45589E-05 | 0.0000138 | 0.0188277 | 0.01020778 | 0.00032901 | 0.001583476 |
| set3 | BT3797 | 0.000580779 | 0.000241522 | 0.239531 | 0.08681114 | 0.00455223 | 0.00768081 |
| set3 | BT3799 | 1.58551E-05 | 0.0000147 | 0.00301394 | 0.002091149 | 0.00132562 | 0.003475991 |
| set3 | BT3809 | 0.000132424 | 0.0000721 | 0.00032149 | 0.00030821 | 0.00257571 | 0.005222284 |
| set3 | BT3845 | 5.97932E-05 | 0.0000389 | 0.00151493 | 0.001151643 | 0.000447982 | 0.001855339 |
| set3 | BT3847 | 0.334948 | 0.06660781 | 1.10885E-05 | 0.0000183 | 0.00118602 | 0.003292884 |
| set3 | BT3853 | 0.495103 | 0.09391592 | 0.864869 | 0.2436378 | 0.000655792 | 0.002302989 |
| set3 | BT3854 | 0.00033891 | 0.000154616 | 0.0225439 | 0.01189686 | 7.35945E-05 | 0.000782832 |
| set3 | BT3855 | 0.00977281 | 0.002859167 | 0.35428 | 0.1196067 | 0.00239762 | 0.004964051 |
| set3 | BT3857 | 1.81078E-05 | 0.0000162 | 8.43E-08 | 0.000000318 | 0.00000145 | 0.000119201 |
| set3 | BT3861 | 0.138081 | 0.03036264 | 0.000886918 | 0.000721629 | 0.00290308 | 0.005689568 |
| set3 | BT3862 | 0.000147025 | 0.0000777 | 0.00998315 | 0.005958078 | 0.000525724 | 0.001999275 |
| set3 | BT3870 | 5.12E-08 | 0.000000614 | 0.00000118 | 0.00000273 | 0.00000174 | 0.000126467 |
| set3 | BT3882 | 1.60837E-05 | 0.0000147 | 0.00000597 | 0.0000109 | 0.000516848 | 0.001981583 |
| set3 | BT3883 | 1.46823E-05 | 0.0000138 | 0.0354207 | 0.01754262 | 0.00109819 | 0.003168431 |
| set3 | BT3907 | 0.00000339 | 0.00000519 | 0.000000342 | 0.000000975 | 0.000064338 | 0.00072504 |
| set3 | BT3923 | 0.518161 | 0.09758055 | 0.000000408 | 0.00000113 | 6.62395E-05 | 0.000731084 |
| set3 | BT3926 | 0.00052501 | 0.000222362 | 0.022601 | 0.01192169 | 0.00393327 | 0.006998451 |
| set3 | BT3927 | 0.00260516 | 0.000887374 | 0.0484988 | 0.02284873 | 0.00134342 | 0.00349651 |
| set3 | BT3931 | 3.00368E-05 | 0.0000234 | 0.907615 | 0.253141 | 0.00322466 | 0.006123596 |
| set3 | BT3941 | 0.00000934 | 0.0000101 | 0.000353199 | 0.000333058 | 0.000379626 | 0.001695937 |
| set3 | BT3951 | 0.000190104 | 0.0000968 | 0.000756031 | 0.000629826 | 0.00051579 | 0.001981583 |
| set3 | BT3973 | 0.0050153 | 0.00158228 | 0.0171971 | 0.009476844 | 0.000394239 | 0.001729678 |
| set3 | BT3980 | 3.53277E-05 | 0.0000264 | 0.119656 | 0.04860071 | 0.00148585 | 0.003722027 |
| set3 | BT3989 | 0.000164459 | 0.0000854 | 0.0572346 | 0.02614132 | 0.00047287 | 0.001906264 |
| set3 | BT3992 | 0.0186748 | 0.005111841 | 0.0131833 | 0.007579615 | 0.000273271 | 0.00138324 |
| set3 | BT4002 | 0.000310879 | 0.000144086 | 0.0495822 | 0.0232413 | 0.00335702 | 0.006301578 |
| set3 | BT4005 | 0.00028297 | 0.000134023 | 3.19594E-05 | 0.000044 | 0.000220956 | 0.001303612 |
| set3 | BT4006 | 0.000000836 | 0.00000232 | 0.0129691 | 0.007466157 | 9.76881E-05 | 0.00091342 |
| set3 | BT4008 | 0.00121364 | 0.000457096 | 0.00282064 | 0.001979775 | 0.000064704 | 0.00072504 |
| set3 | BT4012 | 0.000112124 | 0.0000634 | 7.99924E-05 | 0.000095 | 0.000880155 | 0.002814807 |
| set3 | BT4015 | 5.02589E-05 | 0.0000342 | 0.271505 | 0.09612091 | 0.000978089 | 0.002978746 |
| set3 | BT4019 | 0.000199313 | 0.000100703 | 0.00341159 | 0.002328266 | 0.00142211 | 0.003610751 |
| set3 | BT4030 | 2.01326E-05 | 0.0000173 | 0.171158 | 0.0656142 | 0.00608669 | 0.009302496 |
| set3 | BT4045 | 0.972367 | 0.1659408 | 0.000339555 | 0.000322965 | 0.00462653 | 0.007770448 |
| set3 | BT4052 | 0.00032735 | 0.000149975 | 6.86691E-05 | 0.000084 | 0.00204309 | 0.0044874 |
| set3 | BT4062 | 0.000014278 | 0.0000136 | 0.0102919 | 0.006109142 | 0.00489513 | 0.008059748 |
| set3 | BT4068 | 0.0894743 | 0.02063366 | 0.00272815 | 0.001920437 | 0.00105466 | 0.003102516 |
| set3 | BT4077 | 1.31895E-05 | 0.0000128 | 0.00369244 | 0.002495962 | 0.00300141 | 0.005811045 |
| set3 | BT4081 | 0.011205 | 0.003229488 | 0.450124 | 0.1454594 | 2.90283E-05 | 0.000532771 |
| set3 | BT4084 | 0.0721039 | 0.01708065 | 0.553667 | 0.1715317 | 3.26453E-05 | 0.000577357 |
| set3 | BT4099 | 1.25315E-05 | 0.0000124 | 0.0367593 | 0.01808438 | 0.00217884 | 0.004655547 |
| set3 | BT4105 | 0.00130473 | 0.000486693 | 0.592724 | 0.1808755 | 0.00409305 | 0.007195528 |
| set3 | BT4118 | 3.19907E-05 | 0.0000245 | 0.000025879 | 0.0000369 | 0.000443392 | 0.001855339 |
| set3 | BT4120 | 3.01363E-05 | 0.0000235 | 0.00504772 | 0.003294109 | 0.00597082 | 0.009216278 |
| set3 | BT4138 | 0.000158039 | 0.0000828 | 0.879735 | 0.246847 | 0.000372349 | 0.001695937 |
| set3 | BT4139 | 0.000545045 | 0.000229263 | 0.000379106 | 0.000352534 | 0.0056667 | 0.008934755 |
| set3 | BT4148 | 0.00162826 | 0.000587121 | 0.236125 | 0.08574959 | 0.00537547 | 0.008597049 |
| set3 | BT4159 | 2.86931E-05 | 0.0000226 | 0.15608 | 0.06068133 | 0.000378995 | 0.001695937 |
| set3 | BT4175 | 0.000000999 | 0.00000256 | 0.00000537 | 0.00000997 | 0.000019545 | 0.000448892 |
| set3 | BT4184 | 0.000160927 | 0.0000838 | 0.776287 | 0.2235852 | 0.00414228 | 0.007238728 |
| set3 | BT4188 | 0.00000449 | 0.00000612 | 0.0471999 | 0.02232551 | 0.000763214 | 0.002557846 |
| set3 | BT4207 | 2.63561E-05 | 0.0000211 | 0.110125 | 0.04530842 | 0.00194825 | 0.004356953 |
| set3 | BT4213 | 2.25679E-05 | 0.0000188 | 0.00000659 | 0.0000118 | 9.82448E-05 | 0.00091342 |
| set3 | BT4219 | 6.37995E-05 | 0.000041 | 0.357236 | 0.120235 | 0.00146359 | 0.003685132 |
| set3 | BT4220 | 1.46492E-05 | 0.0000138 | 0.494025 | 0.1563633 | 0.00168134 | 0.004006515 |
| set3 | BT4221 | 6.61401E-05 | 0.0000422 | 3.00846E-05 | 0.0000419 | 5.72579E-05 | 0.000706302 |
| set3 | BT4229 | 0.00162739 | 0.000587087 | 0.0814152 | 0.0351634 | 0.000728986 | 0.002459983 |
| set3 | BT4234 | 0.00064562 | 0.000264992 | 0.244782 | 0.08823769 | 0.000495851 | 0.001951392 |
| set3 | BT4251 | 0.0319687 | 0.0082608 | 0.0901414 | 0.03821914 | 0.000418971 | 0.001790729 |
| set3 | BT4262 | 0.00100868 | 0.000390103 | 0.000846265 | 0.000693989 | 0.000114724 | 0.000952107 |
| set3 | BT4267 | 0.012729 | 0.003634135 | 0.000169461 | 0.000178466 | 8.01809E-05 | 0.000817355 |
| set3 | BT4272 | 0.0417076 | 0.01050129 | 0.0145921 | 0.008255987 | 0.00114184 | 0.003244808 |
| set3 | BT4279 | 0.0306099 | 0.007958592 | 0.0933162 | 0.03926205 | 0.00245312 | 0.00502229 |
| set3 | BT4282 | 0.213885 | 0.04480525 | 0.00918065 | 0.005547658 | 0.00160501 | 0.003894256 |
| set3 | BT4283 | 0.00566746 | 0.001765946 | 0.290568 | 0.1016483 | 0.00610906 | 0.009302496 |
| set3 | BT4285 | 0.0275528 | 0.007245886 | 0.0231293 | 0.01216328 | 0.00540067 | 0.008617116 |
| set3 | BT4306 | 0.0511723 | 0.01263636 | 0.540282 | 0.1685957 | 0.00529795 | 0.008501754 |
| set3 | BT4307 | 0.00725075 | 0.002195126 | 0.00355998 | 0.002414531 | 0.000202762 | 0.001242379 |
| set3 | BT4308 | 0.0591295 | 0.01440388 | 0.00752501 | 0.004697591 | 0.00657813 | 0.009761484 |
| set3 | BT4312 | 0.00000956 | 0.0000102 | 0.201775 | 0.07511069 | 0.0029246 | 0.005724102 |
| set3 | BT4330 | 0.000431604 | 0.000188171 | 3.78727E-05 | 0.0000507 | 0.00362579 | 0.006626406 |
| set3 | BT4338 | 4.19335E-05 | 0.0000299 | 6.02518E-05 | 0.0000752 | 0.000798115 | 0.002644624 |
| set3 | BT4348 | 0.00130441 | 0.000486693 | 0.00291433 | 0.002031133 | 0.00546038 | 0.008672736 |
| set3 | BT4349 | 0.000146958 | 0.0000777 | 0.000498837 | 0.00044112 | 0.00103418 | 0.003079295 |
| set3 | BT4359 | 0.000247359 | 0.000119857 | 0.488004 | 0.1550867 | 0.00177347 | 0.004119162 |
| set3 | BT4366 | 0.000476355 | 0.000204501 | 0.235783 | 0.08565839 | 0.00155501 | 0.003836351 |
| set3 | BT4378 | 0.00356253 | 0.00116523 | 0.00487457 | 0.003196655 | 0.000331448 | 0.001584817 |
| set3 | BT4380 | 0.00229841 | 0.00079288 | 0.0095004 | 0.005706619 | 0.0022751 | 0.004778612 |
| set3 | BT4407 | 0.00255889 | 0.000873167 | 0.0388314 | 0.01892914 | 0.000532797 | 0.002015091 |
| set3 | BT4412 | 0.00000376 | 0.00000551 | 0.0943629 | 0.03963341 | 0.00116909 | 0.003275056 |
| set3 | BT4422 | 0.000891672 | 0.000351313 | 0.00000102 | 0.00000243 | 4.16585E-05 | 0.000623993 |
| set3 | BT4423 | 4.47928E-05 | 0.0000314 | 0.0140344 | 0.007996631 | 0.00154598 | 0.003820494 |
| set3 | BT4424 | 0.000904154 | 0.000355675 | 0.69963 | 0.2058168 | 0.00410436 | 0.00719817 |
| set3 | BT4425 | 0.00166088 | 0.00059661 | 0.000377819 | 0.000351879 | 0.00513201 | 0.008314984 |
| set3 | BT4435 | 0.000250627 | 0.000121053 | 0.0148858 | 0.008394634 | 0.0041698 | 0.00727609 |
| set3 | BT4436 | 0.300876 | 0.06064488 | 0.00534819 | 0.003467712 | 3.17551E-05 | 0.000568462 |
| set3 | BT4447 | 1.01489E-05 | 0.0000107 | 0.00000838 | 0.0000143 | 0.00128579 | 0.003433763 |
| set3 | BT4449 | 0.628363 | 0.1149864 | 0.000143995 | 0.000156843 | 0.000215788 | 0.001298142 |
| set3 | BT4461 | 0.983038 | 0.1673223 | 6.02E-09 | 3.58E-08 | 0.00000034 | 0.0000555 |
| set3 | BT4470 | 0.000000769 | 0.00000221 | 0.00000198 | 0.00000423 | 1.68226E-05 | 0.000428224 |
| set3 | BT4480 | 0.181244 | 0.03859782 | 0.000000924 | 0.00000224 | 0.00000186 | 0.000126467 |
| set3 | BT4481 | 0.245045 | 0.05060442 | 1.56554E-05 | 0.0000244 | 0.000675997 | 0.002350656 |
| set3 | BT4483 | 0.372773 | 0.0728285 | 0.0144235 | 0.008172078 | 8.40236E-05 | 0.000844794 |
| set3 | BT4503 | 0.0000062 | 0.00000759 | 0.00000864 | 0.0000146 | 0.00037871 | 0.001695937 |
| set3 | BT4505 | 0.000407604 | 0.000179568 | 0.125374 | 0.05043142 | 0.00144331 | 0.003646579 |
| set3 | BT4511 | 0.742383 | 0.1324879 | 0.00190416 | 0.001413012 | 0.000230163 | 0.001324944 |
| set3 | BT4517 | 0.0469784 | 0.01168457 | 0.23119 | 0.08424315 | 0.000891532 | 0.002821065 |
| set3 | BT4519 | 1.91995E-05 | 0.0000168 | 0.0287455 | 0.01468209 | 0.000972582 | 0.002974316 |
| set3 | BT4524 | 0.164872 | 0.03557164 | 0.25899 | 0.0924489 | 0.000868767 | 0.002790546 |
| set3 | BT4530 | 0.000557938 | 0.00023407 | 3.38904E-05 | 0.0000462 | 0.000270469 | 0.001379763 |
| set3 | BT4531 | 2.99089E-05 | 0.0000234 | 1.84378E-05 | 0.0000278 | 0.000448701 | 0.001855339 |
| set3 | BT4533 | 4.76215E-05 | 0.0000329 | 0.000957251 | 0.000772419 | 0.00116034 | 0.003260829 |
| set3 | BT4536 | 1.69575E-05 | 0.0000154 | 0.000131393 | 0.000145345 | 0.00589213 | 0.009133231 |
| set3 | BT4565 | 1.81716E-05 | 0.0000162 | 0.355627 | 0.1198604 | 0.000936242 | 0.002893321 |
| set3 | BT4573 | 1.73837E-05 | 0.0000157 | 0.986152 | 0.2684143 | 0.00542678 | 0.008639994 |
| set3 | BT4581 | 0.00000184 | 0.00000357 | 0.789694 | 0.2265822 | 0.000244487 | 0.0013492 |
| set3 | BT4591 | 0.000276981 | 0.000131847 | 0.667397 | 0.198616 | 0.00417853 | 0.00727609 |
| set3 | BT4592 | 0.000100998 | 0.0000587 | 0.0480709 | 0.02266482 | 0.00111406 | 0.003195075 |
| set3 | BT4595 | 5.07872E-05 | 0.0000344 | 0.117414 | 0.04789103 | 0.00114502 | 0.003244808 |
| set3 | BT4598 | 0.000046544 | 0.0000324 | 0.000561925 | 0.000490801 | 0.00279488 | 0.005541167 |
| set3 | BT4609 | 0.864981 | 0.1505337 | 0.361925 | 0.1214409 | 0.000779328 | 0.002594083 |
| set3 | BT4627 | 0.000005 | 0.00000661 | 0.150157 | 0.05883302 | 0.00111442 | 0.003195075 |
| set3 | BT4629 | 0.000269273 | 0.000128907 | 0.000119753 | 0.000133693 | 0.000122046 | 0.000984361 |
| set3 | BT4630 | 0.00000795 | 0.00000904 | 0.45477 | 0.1465296 | 0.000765808 | 0.002560693 |
| set3 | BT4636 | 0.000000233 | 0.00000114 | 2.33566E-05 | 0.000034 | 3.83605E-05 | 0.000614262 |
| set3 | BT4640 | 0.000000229 | 0.00000113 | 1.68369E-05 | 0.0000259 | 0.00000464 | 0.000212801 |
| set3 | BT4645 | 0.00207942 | 0.000724935 | 0.069921 | 0.0309949 | 0.00370806 | 0.00673655 |
| set3 | BT4647 | 2.40034E-05 | 0.0000197 | 1.89056E-05 | 0.0000284 | 0.000360415 | 0.001681956 |
| set3 | BT4648 | 8.01286E-05 | 0.0000495 | 0.000022292 | 0.0000326 | 0.00262876 | 0.00530785 |
| set3 | BT4670 | 0.02642 | 0.006989265 | 0.000628133 | 0.000538887 | 0.00392094 | 0.006984979 |
| set3 | BT4691 | 1.74567E-05 | 0.0000157 | 0.00797495 | 0.004922506 | 0.000211044 | 0.001274879 |
| set3 | BT4705 | 0.000067626 | 0.000043 | 0.000160874 | 0.000171214 | 0.000192445 | 0.001210356 |
| set3 | BT4719 | 0.00000254 | 0.00000427 | 0.00000605 | 0.000011 | 0.000370077 | 0.001695937 |
| set3 | BT4722 | 0.354075 | 0.0697335 | 0.00305493 | 0.002117156 | 0.00464931 | 0.007799784 |
| set3 | BT4730 | 9.36373E-05 | 0.0000556 | 0.0112898 | 0.006626588 | 0.00106899 | 0.003125878 |
| set3 | BT4745 | 2.21071E-05 | 0.0000185 | 0.00000414 | 0.00000805 | 6.28563E-05 | 0.00072504 |
| set3 | BT4753 | 0.0327756 | 0.008446237 | 0.00000269 | 0.00000549 | 0.000194483 | 0.001213913 |
| set3 | BT4757 | 0.00175476 | 0.000625288 | 0.00809738 | 0.004982798 | 0.00160374 | 0.003894256 |
| set3 | SusB | 4.57926E-05 | 0.000032 | 0.117433 | 0.04789103 | 0.00204512 | 0.0044874 |
| set3 | tRNA_54 | 0.27127 | 0.05529595 | 0.00554598 | 0.003582496 | 0.00410087 | 0.00719817 |
| set4 | BT0003 | 0.00000554 | 0.00000703 | 2.09E-08 | 0.000000101 | 0.24115 | 0.1193489 |
| set4 | BT0005 | 8.01848E-05 | 0.0000495 | 0.000523711 | 0.000460755 | 0.0244901 | 0.02286863 |
| set4 | BT0013 | 2.50207E-05 | 0.0000203 | 2.00005E-05 | 0.0000296 | 0.0781182 | 0.05200508 |
| set4 | BT0018 | 0.00000541 | 0.00000689 | 0.00122867 | 0.000960629 | 0.226603 | 0.1146226 |
| set4 | BT0025 | 0.0184969 | 0.00507048 | 2.47718E-05 | 0.0000357 | 0.988123 | 0.3264128 |
| set4 | BT0031 | 0.00000492 | 0.00000654 | 0.0101172 | 0.006026171 | 0.0116241 | 0.01412714 |
| set4 | BT0034 | 0.00124049 | 0.000465252 | 0.000002 | 0.00000426 | 0.810236 | 0.285698 |
| set4 | BT0035 | 0.00874245 | 0.002586718 | 0.00000526 | 0.00000981 | 0.0647757 | 0.04564834 |
| set4 | BT0036 | 0.00290849 | 0.000977498 | 0.00951852 | 0.005708987 | 0.0258835 | 0.02370236 |
| set4 | BT0058 | 0.00243128 | 0.000833021 | 2.34E-09 | 1.69E-08 | 0.300054 | 0.140183 |
| set4 | BT0062 | 0.00404311 | 0.001305493 | 2.20314E-05 | 0.0000323 | 0.0632919 | 0.04486112 |
| set4 | BT0063 | 0.000416752 | 0.000183067 | 0.00854271 | 0.005214335 | 0.00769505 | 0.01083352 |
| set4 | BT0065 | 0.00728046 | 0.002203239 | 0.000845282 | 0.000693681 | 0.018527 | 0.01908501 |
| set4 | BT0067 | 0.0284347 | 0.00744676 | 0.000000292 | 0.000000851 | 0.0406386 | 0.03255339 |
| set4 | BT0074 | 0.00000029 | 0.00000127 | 0.0178317 | 0.009781929 | 0.00751144 | 0.01068726 |
| set4 | BT0078 | 0.000116248 | 0.000065 | 0.00000172 | 0.00000377 | 0.719381 | 0.2630321 |
| set4 | BT0084 | 0.000419312 | 0.000183978 | 0.000256064 | 0.00025312 | 0.348115 | 0.156414 |
| set4 | BT0085 | 4.54563E-05 | 0.0000319 | 4.70031E-05 | 0.0000608 | 0.0603012 | 0.04330591 |
| set4 | BT0091 | 0.000000801 | 0.00000226 | 0.000968458 | 0.000780941 | 0.284061 | 0.1348136 |
| set4 | BT0092 | 0.00000501 | 0.00000661 | 6.19E-12 | 1.92E-10 | 0.00991644 | 0.01279264 |
| set4 | BT0099 | 0.00470397 | 0.001497793 | 0.000387457 | 0.000358643 | 0.423368 | 0.1803453 |
| set4 | BT0102 | 1.19768E-05 | 0.000012 | 0.00730456 | 0.004593204 | 0.0165593 | 0.01776364 |
| set4 | BT0105 | 0.00777006 | 0.002332749 | 0.00312699 | 0.002160897 | 0.0795513 | 0.05264729 |
| set4 | BT0107 | 0.00342172 | 0.001123057 | 2.73436E-05 | 0.0000387 | 0.0255477 | 0.02348275 |
| set4 | BT0110 | 0.000286252 | 0.000135071 | 3.02E-08 | 0.000000136 | 0.0930318 | 0.05888881 |
| set4 | BT0121 | 0.0174268 | 0.00481024 | 1.45639E-05 | 0.0000228 | 0.0757629 | 0.05092369 |
| set4 | BT0125 | 0.000266534 | 0.000127758 | 0.00281207 | 0.001974907 | 0.0219214 | 0.02136709 |
| set4 | BT0127 | 0.00636764 | 0.001955935 | 0.000617321 | 0.00053112 | 0.514715 | 0.2075714 |
| set4 | BT0141 | 0.0233549 | 0.006248296 | 0.00117151 | 0.000921306 | 0.913137 | 0.3095408 |
| set4 | BT0151 | 1.06711E-05 | 0.000011 | 0.000000555 | 0.00000146 | 0.0118403 | 0.01431681 |
| set4 | BT0153 | 5.63146E-05 | 0.0000371 | 0.00456637 | 0.003010889 | 0.00967024 | 0.01255095 |
| set4 | BT0157 | 0.00049625 | 0.000211721 | 0.00293876 | 0.002044859 | 0.533164 | 0.2121818 |
| set4 | BT0164 | 0.00242775 | 0.000832188 | 0.000420126 | 0.000382724 | 0.16518 | 0.08973756 |
| set4 | BT0165 | 0.00810839 | 0.002421835 | 3.1E-09 | 2.14E-08 | 0.209443 | 0.1074608 |
| set4 | BT0167 | 6.15846E-05 | 0.0000399 | 0.000646674 | 0.00055166 | 0.160709 | 0.08795966 |
| set4 | BT0168 | 0.00000527 | 0.00000678 | 0.000587692 | 0.000508891 | 0.857774 | 0.2968976 |
| set4 | BT0175 | 0.000380992 | 0.000170123 | 0.000404977 | 0.000371448 | 0.780392 | 0.2786554 |
| set4 | BT0181 | 3.39995E-05 | 0.0000256 | 0.00526552 | 0.003417779 | 0.212084 | 0.108626 |
| set4 | BT0184 | 0.000074909 | 0.0000466 | 0.00126653 | 0.000985085 | 0.0321324 | 0.02770531 |
| set4 | BT0189 | 0.000110838 | 0.0000628 | 0.000000084 | 0.000000318 | 0.00776389 | 0.01087477 |
| set4 | BT0190 | 1.65937E-05 | 0.0000151 | 0.000498527 | 0.00044112 | 0.0840528 | 0.05459412 |
| set4 | BT0194 | 0.000034025 | 0.0000256 | 0.0142647 | 0.008097297 | 0.0154805 | 0.01698365 |
| set4 | BT0196 | 0.00000369 | 0.00000551 | 0.00000897 | 0.0000152 | 0.0107499 | 0.01347565 |
| set4 | BT0198 | 0.000486779 | 0.000208149 | 1.30033E-05 | 0.0000208 | 0.691811 | 0.2560571 |
| set4 | BT0201 | 0.000158304 | 0.0000828 | 0.000839485 | 0.000690304 | 0.0189804 | 0.01938879 |
| set4 | BT0210 | 0.000105403 | 0.0000606 | 0.00140175 | 0.001073712 | 0.169438 | 0.09157632 |
| set4 | BT0215 | 0.000110923 | 0.0000628 | 0.000304636 | 0.000293918 | 0.111669 | 0.06714437 |
| set4 | BT0223 | 0.0331009 | 0.008518465 | 5.98E-08 | 0.000000239 | 0.143991 | 0.08081972 |
| set4 | BT0231 | 1.01481E-05 | 0.0000107 | 0.00726881 | 0.004575488 | 0.573094 | 0.2234411 |
| set4 | BT0232 | 0.00334134 | 0.001099536 | 0.00374167 | 0.00252223 | 0.114152 | 0.06814452 |
| set4 | BT0236 | 0.000046383 | 0.0000323 | 0.0161712 | 0.008981152 | 0.354285 | 0.1583141 |
| set4 | BT0239 | 1.94963E-05 | 0.0000169 | 0.0141706 | 0.008057017 | 0.576463 | 0.2244565 |
| set4 | BT0248 | 0.00000496 | 0.00000657 | 0.000000372 | 0.00000104 | 0.389829 | 0.169753 |
| set4 | BT0249 | 0.00000418 | 0.00000585 | 0.00279063 | 0.00196099 | 0.0401104 | 0.03226237 |
| set4 | BT0251 | 0.00000193 | 0.00000368 | 0.00215511 | 0.001573921 | 0.122523 | 0.07156938 |
| set4 | BT0252 | 0.00417472 | 0.001342833 | 0.000000096 | 0.000000348 | 0.60892 | 0.2334007 |
| set4 | BT0253 | 0.000000409 | 0.00000152 | 9.97384E-05 | 0.000115071 | 0.141817 | 0.07991397 |
| set4 | BT0255 | 0.000135973 | 0.0000733 | 0.00245211 | 0.001752679 | 0.0148044 | 0.01658905 |
| set4 | BT0258 | 0.000000139 | 0.000000919 | 0.000150196 | 0.00016228 | 0.805572 | 0.2844634 |
| set4 | BT0263 | 0.00127761 | 0.000478436 | 0.00784937 | 0.004857402 | 0.593352 | 0.2291912 |
| set4 | BT0264 | 3.57671E-05 | 0.0000266 | 6.64E-08 | 0.000000261 | 0.0105014 | 0.0132433 |
| set4 | BT0267 | 0.000000396 | 0.00000148 | 1.85E-08 | 9.04E-08 | 0.976535 | 0.3233829 |
| set4 | BT0273 | 0.0161949 | 0.004499743 | 0.000000017 | 0.000000084 | 0.26416 | 0.1284269 |
| set4 | BT0276 | 0.00849445 | 0.002519249 | 0.000000616 | 0.00000159 | 0.388499 | 0.169272 |
| set4 | BT0284 | 0.0369347 | 0.009409108 | 0.000000368 | 0.00000103 | 0.0374845 | 0.03079139 |
| set4 | BT0290 | 0.0334796 | 0.00860422 | 0.000106842 | 0.000121755 | 0.0114886 | 0.01405361 |
| set4 | BT0294 | 0.00029365 | 0.000137788 | 0.000000266 | 0.000000792 | 0.357873 | 0.1594321 |
| set4 | BT0299 | 0.00000847 | 0.00000943 | 0.00000694 | 0.0000123 | 0.02253 | 0.02172491 |
| set4 | BT0301 | 0.00805646 | 0.002409177 | 0.0021048 | 0.001542775 | 0.40194 | 0.1735616 |
| set4 | BT0310 | 0.0169748 | 0.004697469 | 0.000348821 | 0.000329701 | 0.0915043 | 0.05807216 |
| set4 | BT0316 | 0.0137373 | 0.003894096 | 0.0157042 | 0.00875803 | 0.0115026 | 0.01405902 |
| set4 | BT0324 | 0.025749 | 0.006830856 | 1.12E-11 | 2.76E-10 | 0.415502 | 0.1779759 |
| set4 | BT0325 | 6.57121E-05 | 0.000042 | 0.00000752 | 0.0000131 | 0.332835 | 0.1513084 |
| set4 | BT0326 | 0.00000197 | 0.00000371 | 0.000000151 | 0.000000511 | 0.106375 | 0.06503536 |
| set4 | BT0332 | 0.00952635 | 0.002797585 | 0.0153735 | 0.008609376 | 0.971999 | 0.3227103 |
| set4 | BT0348 | 0.000115248 | 0.0000646 | 0.00166873 | 0.001255915 | 0.02703 | 0.02438713 |
| set4 | BT0376 | 0.0030662 | 0.001020981 | 0.00000411 | 0.00000803 | 0.170048 | 0.09167513 |
| set4 | BT0377 | 0.0311575 | 0.008076 | 0.0122814 | 0.007128938 | 0.478455 | 0.1976177 |
| set4 | BT0382 | 1.60167E-05 | 0.0000147 | 0.00322117 | 0.002216585 | 0.531246 | 0.2116218 |
| set4 | BT0411 | 0.000000678 | 0.00000204 | 0.000876774 | 0.000715608 | 0.611519 | 0.2339485 |
| set4 | BT0414 | 0.0203903 | 0.005533395 | 0.00000192 | 0.00000413 | 0.0557016 | 0.04082149 |
| set4 | BT0419 | 0.000117977 | 0.0000657 | 1.29684E-05 | 0.0000208 | 0.091051 | 0.05785952 |
| set4 | BT0423 | 0.00569185 | 0.001772087 | 0.00935552 | 0.005633601 | 0.0095233 | 0.01242219 |
| set4 | BT0443 | 0.00971829 | 0.002846518 | 0.000013856 | 0.000022 | 0.0927167 | 0.05874002 |
| set4 | BT0450 | 0.000675847 | 0.000275308 | 0.0111497 | 0.0065571 | 0.681906 | 0.2534127 |
| set4 | BT0457 | 0.0183845 | 0.00504515 | 6.4E-09 | 3.73E-08 | 0.0279722 | 0.02503715 |
| set4 | BT0469 | 0.034946 | 0.008941613 | 0.00497796 | 0.003255619 | 0.945996 | 0.3174047 |
| set4 | BT0471 | 0.00107174 | 0.000411547 | 0.0145272 | 0.008223119 | 0.110681 | 0.06677795 |
| set4 | BT0476 | 0.00129725 | 0.000484657 | 0.00000019 | 0.000000611 | 0.267707 | 0.1294801 |
| set4 | BT0481 | 0.0041204 | 0.001327052 | 0.00107656 | 0.000858003 | 0.592526 | 0.2290101 |
| set4 | BT0486 | 0.0286367 | 0.007494475 | 0.0001886 | 0.000195385 | 0.095971 | 0.0603589 |
| set4 | BT0493 | 0.000375777 | 0.000168092 | 3.78E-10 | 4.18E-09 | 0.0394814 | 0.03201961 |
| set4 | BT0495 | 0.000001 | 0.00000256 | 0.00397124 | 0.002653241 | 0.470479 | 0.1950918 |
| set4 | BT0499 | 0.000358896 | 0.000162073 | 0.000405289 | 0.000371448 | 0.665168 | 0.2487676 |
| set4 | BT0501 | 0.00000335 | 0.00000516 | 9.11E-08 | 0.000000336 | 0.0152521 | 0.01685907 |
| set4 | BT0504 | 0.00034682 | 0.000157655 | 2.55349E-05 | 0.0000366 | 0.019006 | 0.01940144 |
| set4 | BT0507 | 0.00000818 | 0.00000923 | 0.00063286 | 0.000541788 | 0.413409 | 0.1771828 |
| set4 | BT0511 | 0.0343725 | 0.008815183 | 0.00000329 | 0.00000657 | 0.0193321 | 0.01963873 |
| set4 | BT0520 | 0.0234833 | 0.00627821 | 0.000497307 | 0.000440735 | 0.953332 | 0.3186507 |
| set4 | BT0528 | 0.00326426 | 0.00107792 | 0.000641791 | 0.000547881 | 0.216463 | 0.1104834 |
| set4 | BT0530 | 0.002988 | 0.000999151 | 0.000927112 | 0.000752119 | 0.647813 | 0.2438917 |
| set4 | BT0532 | 0.00000954 | 0.0000102 | 8.40134E-05 | 0.0000992 | 0.0515671 | 0.03852231 |
| set4 | BT0533 | 1.42781E-05 | 0.0000136 | 0.000788849 | 0.000653112 | 0.00773127 | 0.01084768 |
| set4 | BT0554 | 0.0014851 | 0.000542217 | 1.19925E-05 | 0.0000196 | 0.0448565 | 0.03485744 |
| set4 | BT0556 | 0.00134868 | 0.000500866 | 0.0131923 | 0.007581181 | 0.0988106 | 0.06161681 |
| set4 | BT0559 | 2.86664E-05 | 0.0000226 | 2.37108E-05 | 0.0000344 | 0.0186659 | 0.0191877 |
| set4 | BT0560 | 0.000844366 | 0.000335645 | 0.000882759 | 0.000719677 | 0.217055 | 0.1106607 |
| set4 | BT0561 | 0.000140292 | 0.000075 | 0.00000578 | 0.0000106 | 0.0623467 | 0.04440559 |
| set4 | BT0582 | 8.08471E-05 | 0.0000496 | 8.53E-08 | 0.000000318 | 0.227708 | 0.1149439 |
| set4 | BT0583 | 0.00000136 | 0.00000308 | 0.00133359 | 0.001027366 | 0.0481605 | 0.03659199 |
| set4 | BT0587 | 0.00000401 | 0.00000575 | 0.00384344 | 0.002577837 | 0.0871741 | 0.05597348 |
| set4 | BT0588 | 0.00045944 | 0.000198138 | 0.000000442 | 0.0000012 | 0.00885289 | 0.01177255 |
| set4 | BT0594 | 0.00836651 | 0.00248813 | 0.00240094 | 0.001723242 | 0.643721 | 0.2426009 |
| set4 | BT0598 | 0.0315385 | 0.008163572 | 0.000421581 | 0.000383182 | 0.279324 | 0.1334789 |
| set4 | BT0600 | 0.00352788 | 0.001155706 | 0.00742408 | 0.004648999 | 0.487475 | 0.2000056 |
| set4 | BT0601 | 0.0221635 | 0.005959043 | 1.87318E-05 | 0.0000282 | 0.0158548 | 0.01726084 |
| set4 | BT0610 | 0.000948368 | 0.000369992 | 0.0000024 | 0.00000498 | 0.0699968 | 0.04817142 |
| set4 | BT0622 | 0.0295255 | 0.007700455 | 0.00282817 | 0.001983908 | 0.738235 | 0.2679034 |
| set4 | BT0632 | 0.000313213 | 0.000144813 | 0.0055546 | 0.003586147 | 0.567228 | 0.221802 |
| set4 | BT0636 | 0.00700067 | 0.002127927 | 0.00000178 | 0.00000387 | 0.0662106 | 0.04639227 |
| set4 | BT0637 | 0.00000196 | 0.00000371 | 0.00111516 | 0.000884477 | 0.0222892 | 0.02158228 |
| set4 | BT0648 | 4.63047E-05 | 0.0000323 | 0.000348709 | 0.000329701 | 0.834188 | 0.2914137 |
| set4 | BT0657 | 1.07691E-05 | 0.0000111 | 0.0151191 | 0.00848658 | 0.103663 | 0.06377573 |
| set4 | BT0658 | 0.00820503 | 0.002446837 | 8.33333E-05 | 0.0000985 | 0.386832 | 0.1687483 |
| set4 | BT0663 | 0.000983146 | 0.000382377 | 0.00000168 | 0.00000369 | 0.46115 | 0.1923099 |
| set4 | BT0664 | 0.0279297 | 0.007329723 | 0.00000235 | 0.0000049 | 0.336697 | 0.1522625 |
| set4 | BT0675 | 0.0125811 | 0.003594622 | 0.00915658 | 0.005535883 | 0.492748 | 0.2010323 |
| set4 | BT0678 | 0.00651291 | 0.001995697 | 0.000025737 | 0.0000368 | 0.93153 | 0.3139864 |
| set4 | BT0680 | 0.00000517 | 0.00000669 | 0.000000961 | 0.00000231 | 0.326851 | 0.1494589 |
| set4 | BT0682 | 2.24154E-05 | 0.0000187 | 4.57405E-05 | 0.0000594 | 0.0265208 | 0.02412535 |
| set4 | BT0683 | 3.46831E-05 | 0.000026 | 0.00773766 | 0.004812929 | 0.415828 | 0.1780636 |
| set4 | BT0690 | 8.45181E-05 | 0.0000513 | 0.000232114 | 0.000231721 | 0.121942 | 0.071372 |
| set4 | BT0694 | 0.0374028 | 0.00952515 | 0.00000984 | 0.0000164 | 0.954941 | 0.3188754 |
| set4 | BT0695 | 0.00127817 | 0.000478436 | 0.000265567 | 0.000261444 | 0.466931 | 0.1940042 |
| set4 | BT0700 | 0.00960035 | 0.002817424 | 0.000160155 | 0.00017075 | 0.0282214 | 0.02521408 |
| set4 | BT0706 | 0.000118032 | 0.0000657 | 1.3E-11 | 3.08E-10 | 0.742068 | 0.2688955 |
| set4 | BT0708 | 1.55E-08 | 0.000000297 | 1.15479E-05 | 0.0000189 | 0.531052 | 0.2116218 |
| set4 | BT0709 | 0.0000025 | 0.00000422 | 2.73E-11 | 5.42E-10 | 0.0111118 | 0.01378209 |
| set4 | BT0728 | 0.000054323 | 0.0000363 | 0.000999863 | 0.000803583 | 0.339679 | 0.1533215 |
| set4 | BT0731 | 0.000121013 | 0.0000671 | 3.33383E-05 | 0.0000456 | 0.0583818 | 0.04219584 |
| set4 | BT0733 | 0.0339085 | 0.008711489 | 0.000248327 | 0.000246278 | 0.489977 | 0.2003959 |
| set4 | BT0739 | 0.00432459 | 0.001386914 | 0.00000947 | 0.0000159 | 0.5939 | 0.2292825 |
| set4 | BT0740 | 0.00461723 | 0.001473273 | 5.91948E-05 | 0.0000743 | 0.744664 | 0.2695586 |
| set4 | BT0748 | 0.000267187 | 0.00012799 | 0.0140835 | 0.008020824 | 0.150689 | 0.08343993 |
| set4 | BT0759 | 0.000517472 | 0.000219292 | 0.00429282 | 0.002849183 | 0.237899 | 0.1183384 |
| set4 | BT0761 | 0.0011499 | 0.000436686 | 0.000000798 | 0.00000198 | 0.0299114 | 0.02637088 |
| set4 | BT0763 | 0.00011839 | 0.0000658 | 3.59005E-05 | 0.0000484 | 0.240441 | 0.119173 |
| set4 | BT0764 | 0.00000427 | 0.00000592 | 0.00204579 | 0.001505919 | 0.158969 | 0.08710859 |
| set4 | BT0776 | 0.000236501 | 0.000115707 | 0.00705169 | 0.00445274 | 0.260356 | 0.1269063 |
| set4 | BT0777 | 0.00216602 | 0.000750975 | 0.00706724 | 0.004460227 | 0.0698203 | 0.04811763 |
| set4 | BT0781 | 0.0220145 | 0.005930267 | 0.00606652 | 0.003873179 | 0.44782 | 0.18791 |
| set4 | BT0790 | 0.00000728 | 0.00000848 | 0.00000096 | 0.00000231 | 0.0770073 | 0.0515931 |
| set4 | BT0810 | 0.000084054 | 0.0000511 | 3.02493E-05 | 0.000042 | 0.14516 | 0.08129841 |
| set4 | BT0817 | 0.00699416 | 0.002126803 | 0.00126892 | 0.000985085 | 0.0416034 | 0.03299321 |
| set4 | BT0833 | 0.000180983 | 0.0000926 | 0.00315305 | 0.002176416 | 0.552118 | 0.2172827 |
| set4 | BT0841 | 0.0100151 | 0.002921428 | 0.00394866 | 0.00264254 | 0.2067 | 0.1063508 |
| set4 | BT0848 | 0.0157328 | 0.004390998 | 0.00285839 | 0.001998347 | 0.0194919 | 0.01975799 |
| set4 | BT0859 | 0.000430506 | 0.0001878 | 0.0107982 | 0.0063721 | 0.0399122 | 0.03218489 |
| set4 | BT0866 | 0.00000212 | 0.0000039 | 3.43E-09 | 0.000000023 | 0.0134204 | 0.0155523 |
| set4 | BT0870 | 0.0142285 | 0.004016787 | 1.80682E-05 | 0.0000275 | 0.375733 | 0.1651752 |
| set4 | BT0874 | 0.00255125 | 0.000871706 | 8.77E-09 | 4.79E-08 | 0.309123 | 0.1432347 |
| set4 | BT0881 | 0.000000659 | 0.00000202 | 0.0123818 | 0.007180316 | 0.0397718 | 0.03218013 |
| set4 | BT0882 | 0.00739501 | 0.00222988 | 0.000522056 | 0.000459969 | 0.0150949 | 0.01678643 |
| set4 | BT0888 | 0.0353044 | 0.009018073 | 0.00000242 | 0.00000501 | 0.887864 | 0.3038025 |
| set4 | BT0893 | 0.00015415 | 0.0000812 | 0.011665 | 0.0068203 | 0.0427053 | 0.03366698 |
| set4 | BT0904 | 2.40174E-05 | 0.0000197 | 0.00782997 | 0.00484788 | 0.00982782 | 0.01270798 |
| set4 | BT0910 | 0.00013898 | 0.0000746 | 0.00520516 | 0.003385878 | 0.0615813 | 0.04400992 |
| set4 | BT0917 | 2.26611E-05 | 0.0000189 | 0.00271808 | 0.001915581 | 0.272854 | 0.1312345 |
| set4 | BT0921 | 5.82783E-05 | 0.0000382 | 0.00134412 | 0.001034252 | 0.419269 | 0.1791712 |
| set4 | BT0927 | 0.0364934 | 0.00931236 | 7.95E-09 | 4.44E-08 | 0.0351136 | 0.02940327 |
| set4 | BT0932 | 1.29488E-05 | 0.0000127 | 0.000000141 | 0.000000483 | 0.426587 | 0.1813482 |
| set4 | BT0933 | 0.00000872 | 0.0000096 | 0.000553169 | 0.000483503 | 0.0550436 | 0.04041999 |
| set4 | BT0941 | 0.000441944 | 0.000191795 | 0.000478254 | 0.00042553 | 0.53491 | 0.2125749 |
| set4 | BT0946 | 0.000157613 | 0.0000826 | 0.000459513 | 0.000412078 | 0.0150255 | 0.01672192 |
| set4 | BT0950 | 0.0115898 | 0.003334046 | 0.00263387 | 0.001866032 | 0.169399 | 0.09157632 |
| set4 | BT0953 | 0.000372701 | 0.000166947 | 0.000164285 | 0.000174184 | 0.103843 | 0.06383727 |
| set4 | BT0954 | 0.00292655 | 0.000982695 | 0.000947674 | 0.000766227 | 0.834945 | 0.2916088 |
| set4 | BT0959 | 0.0221402 | 0.005955781 | 1.15412E-05 | 0.0000189 | 0.803823 | 0.2843674 |
| set4 | BT0968 | 4.14844E-05 | 0.0000297 | 4.65271E-05 | 0.0000603 | 0.366246 | 0.1623738 |
| set4 | BT0975 | 0.000112439 | 0.0000634 | 0.0156326 | 0.008730191 | 0.0469541 | 0.03591705 |
| set4 | BT0977 | 0.000598406 | 0.000247899 | 0.000415916 | 0.000380322 | 0.593463 | 0.2291912 |
| set4 | BT0979 | 0.00000065 | 0.00000202 | 5.54368E-05 | 0.0000703 | 0.103156 | 0.06362504 |
| set4 | BT0983 | 0.000373315 | 0.000167089 | 0.0102692 | 0.006098664 | 0.149622 | 0.082979 |
| set4 | BT0993 | 0.00032783 | 0.000150096 | 1.23873E-05 | 0.0000201 | 0.00805738 | 0.01111414 |
| set4 | BT1000 | 0.00010433 | 0.0000601 | 0.00800551 | 0.004933805 | 0.286823 | 0.1356858 |
| set4 | BT1003 | 0.0152011 | 0.004253283 | 0.000245785 | 0.000243957 | 0.0432946 | 0.03404016 |
| set4 | BT1010 | 0.00090976 | 0.000357324 | 0.0162033 | 0.008990713 | 0.278045 | 0.133164 |
| set4 | BT1025 | 0.022395 | 0.006016487 | 0.000758942 | 0.000630946 | 0.151067 | 0.08358345 |
| set4 | BT1030 | 0.0223873 | 0.006016487 | 0.00100803 | 0.000809608 | 0.121748 | 0.07128688 |
| set4 | BT1036 | 1.24E-08 | 0.000000276 | 0.00196167 | 0.00144943 | 0.167027 | 0.0905065 |
| set4 | BT1043 | 6.74E-08 | 0.000000654 | 0.0178995 | 0.009810212 | 0.0922676 | 0.05850599 |
| set4 | BT1044 | 1.19E-09 | 0.000000165 | 0.000256061 | 0.00025312 | 0.217886 | 0.1110167 |
| set4 | BT1049 | 2.03991E-05 | 0.0000175 | 9.93601E-05 | 0.000114744 | 0.00924863 | 0.012176 |
| set4 | BT1050 | 0.000000222 | 0.00000111 | 0.000000203 | 0.000000641 | 0.055362 | 0.04063347 |
| set4 | BT1051 | 0.000169206 | 0.0000874 | 2.96361E-05 | 0.0000414 | 0.11674 | 0.06926632 |
| set4 | BT1057 | 0.00000485 | 0.00000646 | 0.000270672 | 0.000264102 | 0.173685 | 0.09311743 |
| set4 | BT1066 | 8.19569E-05 | 0.0000501 | 0.000228791 | 0.000229254 | 0.331282 | 0.1506957 |
| set4 | BT1074 | 0.00614005 | 0.001892999 | 0.000000021 | 0.000000101 | 0.0218226 | 0.02131325 |
| set4 | BT1075 | 0.00120609 | 0.000455045 | 2.17E-09 | 1.59E-08 | 0.334111 | 0.1515744 |
| set4 | BT1078 | 0.01177 | 0.003378181 | 0.00799074 | 0.004929733 | 0.349122 | 0.1568185 |
| set4 | BT1086 | 0.000210802 | 0.000105243 | 0.00114614 | 0.000904298 | 0.0277917 | 0.02492118 |
| set4 | BT1089 | 0.00537018 | 0.001679539 | 0.00183401 | 0.001368371 | 0.0968024 | 0.06075642 |
| set4 | BT1097 | 0.0074954 | 0.002258352 | 0.00000266 | 0.00000545 | 0.320305 | 0.1471157 |
| set4 | BT1099 | 0.000793257 | 0.000316659 | 6.26E-08 | 0.000000248 | 0.0644212 | 0.04544215 |
| set4 | BT1112 | 0.00212917 | 0.000739893 | 0.00109741 | 0.000872116 | 0.498819 | 0.2026057 |
| set4 | BT1120 | 1.17558E-05 | 0.0000119 | 5.81142E-05 | 0.0000732 | 0.208518 | 0.1070611 |
| set4 | BT1130 | 0.000000059 | 0.000000654 | 0.000335047 | 0.00031918 | 0.193364 | 0.1010476 |
| set4 | BT1133 | 0.000036137 | 0.0000268 | 3.09E-08 | 0.000000138 | 0.814624 | 0.2868998 |
| set4 | BT1140 | 0.0351806 | 0.008990071 | 0.00322237 | 0.002216585 | 0.0176797 | 0.01845259 |
| set4 | BT1143 | 0.00136321 | 0.000505141 | 0.00000664 | 0.0000118 | 0.129216 | 0.07455921 |
| set4 | BT1144 | 0.0198422 | 0.005392388 | 0.000208012 | 0.000212399 | 0.280454 | 0.1337939 |
| set4 | BT1146 | 0.00296998 | 0.000995952 | 0.00000193 | 0.00000416 | 0.0664695 | 0.04648492 |
| set4 | BT1147 | 0.0189266 | 0.005171415 | 0.000405052 | 0.000371448 | 0.0128162 | 0.01507464 |
| set4 | BT1148 | 1.03338E-05 | 0.0000108 | 0.00006094 | 0.0000759 | 0.0474453 | 0.0362331 |
| set4 | BT1154 | 1.66381E-05 | 0.0000151 | 0.00000455 | 0.00000875 | 0.0277146 | 0.02486723 |
| set4 | BT1155 | 0.00111742 | 0.000426707 | 0.000158456 | 0.000169387 | 0.816935 | 0.2873699 |
| set4 | BT1163 | 0.0127895 | 0.003650031 | 0.00678043 | 0.004294925 | 0.0422324 | 0.0333479 |
| set4 | BT1167 | 1.25875E-05 | 0.0000124 | 1.28E-08 | 6.56E-08 | 0.342988 | 0.1545361 |
| set4 | BT1170 | 0.0031466 | 0.001044994 | 6.96714E-05 | 0.0000849 | 0.812938 | 0.2865131 |
| set4 | BT1175 | 0.0307996 | 0.00799967 | 0.00000673 | 0.0000119 | 0.266096 | 0.1289686 |
| set4 | BT1177 | 0.00195053 | 0.000685049 | 3.10777E-05 | 0.000043 | 0.371663 | 0.1639829 |
| set4 | BT1178 | 0.00309134 | 0.001028447 | 0.00200966 | 0.001481128 | 0.066791 | 0.04662213 |
| set4 | BT1183 | 0.000137309 | 0.0000739 | 2.99E-08 | 0.000000135 | 0.13673 | 0.07761353 |
| set4 | BT1187 | 0.00168646 | 0.000604078 | 1.16E-08 | 6.02E-08 | 0.0197954 | 0.01994387 |
| set4 | BT1188 | 0.000195962 | 0.0000992 | 0.0038184 | 0.002565316 | 0.826172 | 0.2895782 |
| set4 | BT1189 | 0.00318043 | 0.001054378 | 0.000142172 | 0.000155137 | 0.548345 | 0.216378 |
| set4 | BT1190 | 5.47228E-05 | 0.0000364 | 0.00172455 | 0.001294699 | 0.705787 | 0.2593337 |
| set4 | BT1191 | 0.00716057 | 0.002171299 | 0.000488022 | 0.000433142 | 0.0395313 | 0.03204238 |
| set4 | BT1201 | 0.00378555 | 0.001229145 | 3.95939E-05 | 0.0000525 | 0.078609 | 0.05221342 |
| set4 | BT1212 | 0.00950686 | 0.002793237 | 0.00000112 | 0.00000261 | 0.0186919 | 0.01920028 |
| set4 | BT1227 | 0.00000174 | 0.00000352 | 0.000707523 | 0.000595577 | 0.0127032 | 0.0149716 |
| set4 | BT1231 | 0.000131769 | 0.0000718 | 0.000000409 | 0.00000113 | 0.295858 | 0.1386197 |
| set4 | BT1233 | 0.000000726 | 0.00000215 | 0.000000832 | 0.00000204 | 0.00930752 | 0.01222065 |
| set4 | BT1234 | 0.0101344 | 0.002948996 | 3.33752E-05 | 0.0000456 | 0.422276 | 0.1800892 |
| set4 | BT1244 | 0.00000203 | 0.0000038 | 4.18E-13 | 2.29E-11 | 0.0146449 | 0.0164732 |
| set4 | BT1250 | 0.00000165 | 0.00000342 | 0.00520947 | 0.003386857 | 0.0478654 | 0.03646214 |
| set4 | BT1256 | 0.000000151 | 0.000000979 | 7.29E-08 | 0.000000284 | 0.0497283 | 0.03747285 |
| set4 | BT1260 | 0.0202108 | 0.005488619 | 0.000575555 | 0.000500534 | 0.488045 | 0.2000588 |
| set4 | BT1265 | 0.000739749 | 0.000296865 | 4.37012E-05 | 0.0000573 | 0.0112834 | 0.01386103 |
| set4 | BT1268 | 0.000243084 | 0.000118468 | 0.000268371 | 0.000263008 | 0.0256899 | 0.02356937 |
| set4 | BT1269 | 0.000234315 | 0.000114712 | 1.77E-13 | 1.12E-11 | 0.018458 | 0.01904065 |
| set4 | BT1270 | 0.0248889 | 0.006630572 | 0.00685814 | 0.004339598 | 0.334143 | 0.1515744 |
| set4 | BT1279 | 3.12353E-05 | 0.000024 | 0.00213775 | 0.001563133 | 0.459109 | 0.1917311 |
| set4 | BT1282 | 0.000000064 | 0.000000654 | 0.000736382 | 0.000616433 | 0.305917 | 0.142198 |
| set4 | BT1283 | 3.37E-09 | 0.000000196 | 1.58673E-05 | 0.0000246 | 0.674508 | 0.2513641 |
| set4 | BT1284 | 1.15E-08 | 0.000000264 | 6.51731E-05 | 0.0000804 | 0.369946 | 0.1635203 |
| set4 | BT1286 | 1.67657E-05 | 0.0000152 | 3.61E-08 | 0.000000158 | 0.214051 | 0.1094044 |
| set4 | BT1296 | 0.000214887 | 0.000106719 | 0.000398063 | 0.000366774 | 0.93074 | 0.3138642 |
| set4 | BT1299 | 1.31716E-05 | 0.0000128 | 0.00107112 | 0.000854597 | 0.196796 | 0.1022224 |
| set4 | BT1302 | 0.000289998 | 0.000136413 | 0.00104272 | 0.000834142 | 0.0243411 | 0.02278484 |
| set4 | BT1303 | 0.00517274 | 0.001625851 | 0.000115364 | 0.000129994 | 0.0324492 | 0.02786377 |
| set4 | BT1317 | 0.0266841 | 0.007044675 | 0.00438082 | 0.002898036 | 0.656061 | 0.24603 |
| set4 | BT1318 | 0.00134191 | 0.000499086 | 0.000122693 | 0.000136596 | 0.814817 | 0.2868998 |
| set4 | BT1323 | 0.00284856 | 0.000961626 | 0.000183995 | 0.000191271 | 0.0217687 | 0.02131325 |
| set4 | BT1330 | 0.00430146 | 0.001381252 | 0.0136422 | 0.007813703 | 0.60933 | 0.2334762 |
| set4 | BT1346 | 0.00111539 | 0.000426146 | 0.000817735 | 0.000674715 | 0.0207357 | 0.02066418 |
| set4 | BT1347 | 0.000390701 | 0.000173435 | 0.0101301 | 0.006029517 | 0.0360951 | 0.0298842 |
| set4 | BT1348 | 0.00013429 | 0.0000727 | 0.0172893 | 0.009514631 | 0.0108159 | 0.01353527 |
| set4 | BT1353 | 0.000960248 | 0.000374433 | 0.00249031 | 0.001777879 | 0.024586 | 0.02289991 |
| set4 | BT1354 | 0.000871995 | 0.000345358 | 0.00847965 | 0.005183702 | 0.0248273 | 0.02303696 |
| set4 | BT1361 | 0.00000613 | 0.00000753 | 2.02E-09 | 1.52E-08 | 0.044525 | 0.03469296 |
| set4 | BT1384 | 0.00875649 | 0.002588848 | 0.0086216 | 0.005251873 | 0.0529735 | 0.03933756 |
| set4 | BT1388 | 0.00271234 | 0.000919743 | 0.0102589 | 0.006095544 | 0.125616 | 0.07294071 |
| set4 | BT1393 | 0.00812681 | 0.002426379 | 3.83142E-05 | 0.0000511 | 0.824654 | 0.2893 |
| set4 | BT1396 | 0.00235795 | 0.000809729 | 0.000439351 | 0.000396944 | 0.426558 | 0.1813482 |
| set4 | BT1398 | 0.000467249 | 0.000201048 | 7.50612E-05 | 0.0000903 | 0.0557669 | 0.04084895 |
| set4 | BT1402 | 0.000601402 | 0.000248867 | 0.00000831 | 0.0000142 | 0.0324589 | 0.02786377 |
| set4 | BT1408 | 0.000148523 | 0.0000784 | 0.00795761 | 0.004916506 | 0.696269 | 0.257059 |
| set4 | BT1413 | 0.0043935 | 0.001408417 | 6.09466E-05 | 0.0000759 | 0.0416791 | 0.03303103 |
| set4 | BT1422 | 1.17148E-05 | 0.0000118 | 1.47E-08 | 7.44E-08 | 0.016515 | 0.01775913 |
| set4 | BT1423 | 3.33E-10 | 0.000000111 | 0.00000605 | 0.000011 | 0.312661 | 0.1443728 |
| set4 | BT1424 | 0.000136331 | 0.0000734 | 0.00102364 | 0.000820509 | 0.927458 | 0.3131171 |
| set4 | BT1431 | 0.000000375 | 0.00000143 | 0.00298832 | 0.002074564 | 0.0321968 | 0.02771166 |
| set4 | BT1440 | 0.0147623 | 0.004142759 | 5.23275E-05 | 0.0000669 | 0.192375 | 0.1006604 |
| set4 | BT1446 | 0.0195382 | 0.00531741 | 0.00000513 | 0.0000096 | 0.108569 | 0.06604663 |
| set4 | BT1451 | 0.000387743 | 0.000172527 | 7.93003E-05 | 0.0000943 | 0.952938 | 0.3186414 |
| set4 | BT1452 | 0.00000921 | 0.00000996 | 0.000112229 | 0.00012658 | 0.352868 | 0.157825 |
| set4 | BT1454 | 0.000000002 | 0.000000196 | 0.000000451 | 0.00000122 | 0.0226928 | 0.02180475 |
| set4 | BT1463 | 0.00000876 | 0.00000962 | 0.000194445 | 0.00020041 | 0.00779281 | 0.01089925 |
| set4 | BT1464 | 0.00576045 | 0.001790499 | 0.00942583 | 0.005664644 | 0.0102009 | 0.01303229 |
| set4 | BT1473 | 0.00013309 | 0.0000723 | 0.000000159 | 0.000000531 | 0.0132381 | 0.01540885 |
| set4 | BT1479 | 0.0147378 | 0.004137417 | 0.00172393 | 0.001294699 | 0.929817 | 0.3137692 |
| set4 | BT1480 | 7.92012E-05 | 0.000049 | 0.0103304 | 0.006119966 | 0.0135155 | 0.01559142 |
| set4 | BT1484 | 0.00000581 | 0.00000731 | 4.66111E-05 | 0.0000603 | 0.908688 | 0.3086972 |
| set4 | BT1500 | 0.000442819 | 0.000191955 | 0.00230872 | 0.001668952 | 0.577101 | 0.2246453 |
| set4 | BT1503 | 0.0037034 | 0.001207076 | 3.65004E-05 | 0.000049 | 0.0126096 | 0.01490328 |
| set4 | BT1507 | 0.00321285 | 0.001063728 | 0.00795998 | 0.004916506 | 0.957736 | 0.3194196 |
| set4 | BT1510 | 0.0233393 | 0.00624633 | 0.000461259 | 0.000413337 | 0.403442 | 0.1740289 |
| set4 | BT1517 | 6.89208E-05 | 0.0000436 | 0.00104879 | 0.000837888 | 0.328855 | 0.1501501 |
| set4 | BT1519 | 0.000069784 | 0.0000441 | 0.00000113 | 0.00000263 | 0.896407 | 0.3057133 |
| set4 | BT1521 | 0.00575351 | 0.001789812 | 0.000010718 | 0.0000177 | 0.964355 | 0.3209966 |
| set4 | BT1529 | 0.000694997 | 0.000281744 | 0.000182742 | 0.000190303 | 0.317651 | 0.1461712 |
| set4 | BT1532 | 0.0327116 | 0.008432615 | 0.000278336 | 0.000270705 | 0.551703 | 0.2172359 |
| set4 | BT1533 | 0.0306515 | 0.007966671 | 0.000182749 | 0.000190303 | 0.699167 | 0.2575543 |
| set4 | BT1534 | 0.0238722 | 0.006373176 | 0.00000183 | 0.00000395 | 0.924334 | 0.3123012 |
| set4 | BT1539 | 0.0107161 | 0.003100039 | 0.00212636 | 0.001556689 | 0.306671 | 0.1423459 |
| set4 | BT1545 | 0.000105028 | 0.0000604 | 0.00000116 | 0.00000269 | 0.0097662 | 0.01264197 |
| set4 | BT1546 | 0.0111587 | 0.003217369 | 0.0063962 | 0.004075048 | 0.422169 | 0.1800892 |
| set4 | BT1559 | 0.0300799 | 0.00782886 | 4.78E-08 | 0.0000002 | 0.558383 | 0.2191021 |
| set4 | BT1562 | 0.000342101 | 0.000155754 | 0.00714651 | 0.004505547 | 0.0561495 | 0.04102684 |
| set4 | BT1568 | 0.00758157 | 0.002282498 | 0.00338181 | 0.002310553 | 0.569234 | 0.2223495 |
| set4 | BT1572 | 0.00000112 | 0.00000273 | 0.00543911 | 0.003521926 | 0.011758 | 0.0142519 |
| set4 | BT1574 | 0.00179044 | 0.000636503 | 0.000853761 | 0.000698713 | 0.395159 | 0.1714636 |
| set4 | BT1575 | 0.0006551 | 0.000267721 | 0.0012605 | 0.000982336 | 0.0650827 | 0.04582069 |
| set4 | BT1586 | 0.00000713 | 0.00000838 | 0.0150661 | 0.008468637 | 0.064145 | 0.04531264 |
| set4 | BT1588 | 0.000110091 | 0.0000626 | 0.000000337 | 0.000000964 | 0.0354582 | 0.02954016 |
| set4 | BT1590 | 0.0232853 | 0.006234083 | 0.000418295 | 0.000381842 | 0.29984 | 0.1401295 |
| set4 | BT1594 | 0.00000678 | 0.00000804 | 0.00206489 | 0.001516282 | 0.028693 | 0.02555765 |
| set4 | BT1611 | 0.00100061 | 0.000388172 | 0.000293564 | 0.000284143 | 0.175916 | 0.09400453 |
| set4 | BT1637 | 0.000000154 | 0.000000982 | 0.000000413 | 0.00000114 | 0.00847804 | 0.01139659 |
| set4 | BT1649 | 0.00216958 | 0.000751865 | 4.09E-08 | 0.000000175 | 0.0627828 | 0.04460797 |
| set4 | BT1650 | 0.00000662 | 0.0000079 | 0.00000482 | 0.00000914 | 0.026103 | 0.02383196 |
| set4 | BT1656 | 0.000762257 | 0.000305089 | 4.9E-09 | 3.05E-08 | 0.096686 | 0.06073052 |
| set4 | BT1666 | 0.00000351 | 0.00000534 | 0.00297916 | 0.002069395 | 0.0218661 | 0.02134153 |
| set4 | BT1667 | 0.00170413 | 0.000609542 | 0.0158839 | 0.008837845 | 0.44755 | 0.1878659 |
| set4 | BT1672 | 7.13509E-05 | 0.0000448 | 0.000931041 | 0.0007548 | 0.236435 | 0.1179298 |
| set4 | BT1676 | 0.000000107 | 0.000000849 | 0.00373809 | 0.00252223 | 0.118971 | 0.07013228 |
| set4 | BT1680 | 0.00539533 | 0.001686707 | 0.00127177 | 0.00098603 | 0.00784331 | 0.01094424 |
| set4 | BT1691 | 1.42115E-05 | 0.0000136 | 0.00881536 | 0.005356397 | 0.651562 | 0.244926 |
| set4 | BT1700 | 0.0342515 | 0.008790658 | 5.30169E-05 | 0.0000676 | 0.120537 | 0.07066237 |
| set4 | BT1707 | 5.28344E-05 | 0.0000355 | 0.000108902 | 0.000123752 | 0.583771 | 0.2265808 |
| set4 | BT1713 | 0.00608886 | 0.001880222 | 0.000728566 | 0.000610655 | 0.444712 | 0.1871027 |
| set4 | BT1740 | 0.000647261 | 0.000265522 | 0.000697233 | 0.00058847 | 0.135757 | 0.07724042 |
| set4 | BT1741 | 0.000998232 | 0.000387448 | 0.000278903 | 0.000271039 | 0.485831 | 0.1996255 |
| set4 | BT1742 | 1.60382E-05 | 0.0000147 | 0.00000598 | 0.0000109 | 0.589573 | 0.2281353 |
| set4 | BT1743 | 0.000322078 | 0.000148188 | 0.00642683 | 0.004089475 | 0.0570225 | 0.0414789 |
| set4 | BT1749 | 0.00000045 | 0.00000159 | 5.82E-10 | 6.12E-09 | 0.00969188 | 0.01255684 |
| set4 | BT1750 | 0.000000446 | 0.00000158 | 0.00075987 | 0.000631283 | 0.0365155 | 0.03017817 |
| set4 | BT1775 | 0.00459253 | 0.00146601 | 1.39252E-05 | 0.000022 | 0.0105307 | 0.01325744 |
| set4 | BT1777 | 0.00580813 | 0.001803097 | 0.00000856 | 0.0000146 | 0.600234 | 0.2311978 |
| set4 | BT1788 | 2.45017E-05 | 0.00002 | 0.000000274 | 0.000000806 | 0.0219442 | 0.02137117 |
| set4 | BT1791 | 0.00931104 | 0.002742089 | 0.00021345 | 0.000217217 | 0.136362 | 0.07752455 |
| set4 | BT1792 | 0.000175258 | 0.0000902 | 0.000145014 | 0.000157687 | 0.487537 | 0.2000056 |
| set4 | BT1793 | 0.00190806 | 0.000671068 | 4.1E-09 | 2.65E-08 | 0.219905 | 0.1118125 |
| set4 | BT1795 | 0.000438079 | 0.000190507 | 3.32E-09 | 2.24E-08 | 0.158838 | 0.087098 |
| set4 | BT1798 | 0.00598491 | 0.001852658 | 2.04E-10 | 2.76E-09 | 0.0430121 | 0.03383616 |
| set4 | BT1803 | 0.00000151 | 0.00000327 | 0.00000104 | 0.00000247 | 0.0442275 | 0.03460682 |
| set4 | BT1813 | 0.00510623 | 0.001608285 | 0.00751876 | 0.004697591 | 0.409554 | 0.1758386 |
| set4 | BT1815 | 0.030951 | 0.008027974 | 0.00078622 | 0.000651382 | 0.0169475 | 0.01804029 |
| set4 | BT1826 | 0.00105993 | 0.000408255 | 0.00755093 | 0.004708905 | 0.0240187 | 0.02259087 |
| set4 | BT1837 | 0.000000392 | 0.00000148 | 0.00000647 | 0.0000116 | 0.695887 | 0.257059 |
| set4 | BT1843 | 0.000000663 | 0.00000202 | 0.000345578 | 0.000327662 | 0.115178 | 0.06856123 |
| set4 | BT1844 | 0.00661355 | 0.002023259 | 0.00655824 | 0.004167295 | 0.110506 | 0.06671487 |
| set4 | BT1849 | 0.0306966 | 0.007975655 | 0.0143971 | 0.008160949 | 0.566996 | 0.2217704 |
| set4 | BT1854 | 0.000033646 | 0.0000255 | 0.0164284 | 0.009094725 | 0.0365324 | 0.03017817 |
| set4 | BT1860 | 4.77116E-05 | 0.0000329 | 3.21296E-05 | 0.0000442 | 0.00807012 | 0.01111414 |
| set4 | BT1875 | 0.000104161 | 0.0000601 | 4.45269E-05 | 0.000058 | 0.00999227 | 0.012844 |
| set4 | BT1877 | 0.00523884 | 0.00164526 | 0.00334392 | 0.002289846 | 0.0755313 | 0.05085956 |
| set4 | BT1894 | 0.007785 | 0.002336308 | 0.00000213 | 0.00000453 | 0.326678 | 0.149435 |
| set4 | BT1895 | 3.32214E-05 | 0.0000252 | 0.00254532 | 0.001811797 | 0.0445015 | 0.03469296 |
| set4 | BT1901 | 0.0048472 | 0.001538222 | 0.0000014 | 0.00000315 | 0.234085 | 0.1170974 |
| set4 | BT1903 | 9.63843E-05 | 0.0000567 | 0.000227071 | 0.000228005 | 0.555255 | 0.218108 |
| set4 | BT1924 | 0.00036788 | 0.000165341 | 0.00168119 | 0.001264504 | 0.0846758 | 0.05489162 |
| set4 | BT1926 | 4.96256E-05 | 0.0000339 | 6.23E-08 | 0.000000248 | 0.931913 | 0.3140434 |
| set4 | BT1927 | 0.000180641 | 0.0000926 | 2.75E-08 | 0.000000125 | 0.0534981 | 0.0395751 |
| set4 | BT1928 | 3.28898E-05 | 0.000025 | 1.66E-11 | 3.71E-10 | 0.862023 | 0.2978065 |
| set4 | BT1929 | 0.00000165 | 0.00000342 | 0.00232668 | 0.001679925 | 0.107376 | 0.06553816 |
| set4 | BT1931 | 0.00364793 | 0.001190077 | 0.000684381 | 0.00057892 | 0.0466823 | 0.03577494 |
| set4 | BT1932 | 0.00000615 | 0.00000754 | 0.00000237 | 0.00000492 | 0.631625 | 0.2395181 |
| set4 | BT1934 | 0.000327146 | 0.000149972 | 0.00681447 | 0.004314225 | 0.594594 | 0.2293866 |
| set4 | BT1938 | 0.00186736 | 0.000658283 | 0.0084889 | 0.005186732 | 0.193453 | 0.1010581 |
| set4 | BT1944 | 0.00000509 | 0.00000668 | 0.00000044 | 0.0000012 | 0.511423 | 0.2065844 |
| set4 | BT1947 | 0.000492585 | 0.000210394 | 5.73E-11 | 9.89E-10 | 0.0130349 | 0.01521968 |
| set4 | BT1951 | 0.00472776 | 0.001503471 | 0.006429 | 0.004089475 | 0.171065 | 0.09204897 |
| set4 | BT1963 | 0.0272321 | 0.007174011 | 0.000195295 | 0.000201114 | 0.578864 | 0.2251525 |
| set4 | BT1967 | 0.00000718 | 0.0000084 | 0.000165349 | 0.000174898 | 0.00820487 | 0.01120014 |
| set4 | BT1972 | 0.00143908 | 0.000527962 | 0.00989851 | 0.005916333 | 0.760415 | 0.2735182 |
| set4 | BT1974 | 0.00172016 | 0.000614695 | 0.000100409 | 0.000115654 | 0.0508349 | 0.03811109 |
| set4 | BT1978 | 0.00267187 | 0.000908054 | 1.43539E-05 | 0.0000225 | 0.972886 | 0.3227385 |
| set4 | BT1988 | 5.62168E-05 | 0.0000371 | 0.00000019 | 0.000000611 | 0.023765 | 0.02241968 |
| set4 | BT1994 | 0.000131859 | 0.0000718 | 0.00155409 | 0.001179186 | 0.923063 | 0.3119921 |
| set4 | BT2000 | 0.00152117 | 0.000553517 | 3.39577E-05 | 0.0000462 | 0.633837 | 0.2400468 |
| set4 | BT2002 | 0.0192202 | 0.005247848 | 0.000104423 | 0.000119449 | 0.0943861 | 0.0595919 |
| set4 | BT2021 | 0.00445166 | 0.001424647 | 0.000176826 | 0.000185737 | 0.568325 | 0.2220535 |
| set4 | BT2026 | 0.00607289 | 0.001876056 | 6.60936E-05 | 0.0000814 | 0.525053 | 0.2102961 |
| set4 | BT2029 | 0.000127544 | 0.0000698 | 3.15416E-05 | 0.0000435 | 0.972553 | 0.3227103 |
| set4 | BT2030 | 0.00450269 | 0.001440369 | 0.011247 | 0.006607888 | 0.334058 | 0.1515744 |
| set4 | BT2032 | 0.0194555 | 0.00530062 | 0.000000171 | 0.000000556 | 0.0489719 | 0.03715079 |
| set4 | BT2033 | 0.00096727 | 0.000376783 | 1.74033E-05 | 0.0000266 | 0.0757653 | 0.05092369 |
| set4 | BT2036 | 0.00000404 | 0.00000575 | 0.00000195 | 0.00000418 | 0.157256 | 0.08638701 |
| set4 | BT2038 | 0.00000927 | 0.00000999 | 0.000637538 | 0.000545407 | 0.301551 | 0.1407928 |
| set4 | BT2039 | 4.76578E-05 | 0.0000329 | 0.00107788 | 0.00085829 | 0.0173456 | 0.01826538 |
| set4 | BT2040 | 0.00000107 | 0.00000266 | 0.00144387 | 0.001103177 | 0.14396 | 0.08081972 |
| set4 | BT2045 | 0.0204191 | 0.005539224 | 0.00243925 | 0.001744519 | 0.0356678 | 0.02964748 |
| set4 | BT2049 | 0.000420817 | 0.000184531 | 0.000301321 | 0.000290952 | 0.284554 | 0.1349167 |
| set4 | BT2051 | 3.38568E-05 | 0.0000256 | 0.000714893 | 0.000600943 | 0.0320219 | 0.02769921 |
| set4 | BT2060 | 0.000605965 | 0.000250618 | 0.00119148 | 0.000934577 | 0.0626286 | 0.04454153 |
| set4 | BT2068 | 0.00000627 | 0.0000076 | 0.000186696 | 0.000193745 | 0.139201 | 0.07874212 |
| set4 | BT2087 | 0.00566284 | 0.001765233 | 0.00292883 | 0.002039125 | 0.0403074 | 0.03234992 |
| set4 | BT2093 | 0.000200657 | 0.000101038 | 0.000000336 | 0.000000963 | 0.108768 | 0.06611289 |
| set4 | BT2097 | 0.0031535 | 0.001046826 | 8.56944E-05 | 0.000100891 | 0.478924 | 0.1977557 |
| set4 | BT2103 | 3.88627E-05 | 0.0000284 | 5.95855E-05 | 0.0000746 | 0.280855 | 0.1338547 |
| set4 | BT2104 | 2.59455E-05 | 0.0000209 | 0.000116015 | 0.000130536 | 0.0725872 | 0.04944415 |
| set4 | BT2105 | 0.000437767 | 0.000190507 | 0.000101399 | 0.000116541 | 0.0105397 | 0.01325744 |
| set4 | BT2108 | 0.00216488 | 0.000750923 | 0.0001644 | 0.000174184 | 0.038967 | 0.03167243 |
| set4 | BT2112 | 0.00000857 | 0.00000947 | 0.00727527 | 0.004577169 | 0.660849 | 0.2475307 |
| set4 | BT2114 | 0.000798783 | 0.000318529 | 0.00000171 | 0.00000375 | 0.338122 | 0.1527657 |
| set4 | BT2131 | 0.00581902 | 0.001805737 | 0.000120926 | 0.000134753 | 0.327601 | 0.1497172 |
| set4 | BT2133 | 0.00172365 | 0.000615651 | 0.0126929 | 0.007332565 | 0.00769754 | 0.01083352 |
| set4 | BT2144 | 0.0151887 | 0.004251385 | 0.0163442 | 0.009056413 | 0.0572706 | 0.04161812 |
| set4 | BT2149 | 0.0125082 | 0.003575143 | 0.000148622 | 0.000160867 | 0.0104596 | 0.01322746 |
| set4 | BT2150 | 0.0380391 | 0.009667676 | 0.000481165 | 0.00042737 | 0.894386 | 0.3052514 |
| set4 | BT2152 | 0.0190115 | 0.005192738 | 0.000000919 | 0.00000223 | 0.00952649 | 0.01242219 |
| set4 | BT2164 | 0.00114191 | 0.000434742 | 0.00370019 | 0.002498404 | 0.310439 | 0.1436178 |
| set4 | BT2165 | 1.48311E-05 | 0.0000139 | 0.000383352 | 0.000355531 | 0.278157 | 0.1331742 |
| set4 | BT2170 | 0.000000075 | 0.000000701 | 2.94E-12 | 1.07E-10 | 0.141816 | 0.07991397 |
| set4 | BT2171 | 0.000000105 | 0.000000846 | 6.02E-10 | 6.25E-09 | 0.0819373 | 0.05374322 |
| set4 | BT2181 | 0.000101789 | 0.000059 | 2.41E-08 | 0.000000113 | 0.239174 | 0.118691 |
| set4 | BT2182 | 0.00261419 | 0.00089005 | 0.000293339 | 0.000284143 | 0.162286 | 0.08851465 |
| set4 | BT2187 | 0.0162318 | 0.004505034 | 0.000195768 | 0.000201258 | 0.0548213 | 0.0402816 |
| set4 | BT2188 | 0.000126807 | 0.0000695 | 0.0147591 | 0.008338756 | 0.0606469 | 0.04347655 |
| set4 | BT2195 | 0.00231482 | 0.000797451 | 0.000158885 | 0.000169695 | 0.0309291 | 0.02697648 |
| set4 | BT2217 | 0.0339476 | 0.008718575 | 0.00190881 | 0.001414569 | 0.0398374 | 0.03218013 |
| set4 | BT2218 | 0.0187727 | 0.005134925 | 0.00342994 | 0.002335893 | 0.023694 | 0.02241968 |
| set4 | BT2226 | 0.000000154 | 0.000000982 | 0.00043731 | 0.000395569 | 0.0247747 | 0.02303183 |
| set4 | BT2227 | 0.000136014 | 0.0000733 | 0.000310709 | 0.000298823 | 0.237539 | 0.1182795 |
| set4 | BT2237 | 0.00178516 | 0.000634925 | 0.000374976 | 0.000350174 | 0.971286 | 0.3225722 |
| set4 | BT2254 | 1.25041E-05 | 0.0000124 | 0.000382072 | 0.000354746 | 0.0204336 | 0.02043247 |
| set4 | BT2256 | 0.000160114 | 0.0000835 | 0.000383507 | 0.000355531 | 0.333669 | 0.1515335 |
| set4 | BT2270 | 0.00111949 | 0.000427282 | 0.000000416 | 0.00000114 | 0.48835 | 0.2000721 |
| set4 | BT2273 | 0.00149682 | 0.000545443 | 0.00501592 | 0.003276897 | 0.0444149 | 0.03469029 |
| set4 | BT2274 | 0.0268066 | 0.007074229 | 0.000104939 | 0.000119812 | 0.0259465 | 0.02373044 |
| set4 | BT2277 | 1.81301E-05 | 0.0000162 | 4.30822E-05 | 0.0000566 | 0.968532 | 0.3220219 |
| set4 | BT2278 | 0.0290206 | 0.007584455 | 0.000152271 | 0.000163936 | 0.0459665 | 0.03545723 |
| set4 | BT2288 | 0.00000855 | 0.00000946 | 7.11E-14 | 6.14E-12 | 0.00822562 | 0.01121128 |
| set4 | BT2293 | 2.05974E-05 | 0.0000176 | 6.4E-09 | 3.73E-08 | 0.0823376 | 0.05395756 |
| set4 | BT2295 | 0.00288983 | 0.000973388 | 0.00000654 | 0.0000117 | 0.0175336 | 0.01835799 |
| set4 | BT2298 | 0.000000585 | 0.00000189 | 0.00217792 | 0.001588659 | 0.0246953 | 0.02297256 |
| set4 | BT2305 | 2.66184E-05 | 0.0000213 | 0.0000015 | 0.00000335 | 0.0335013 | 0.02845903 |
| set4 | BT2320 | 2.06229E-05 | 0.0000176 | 8.5E-10 | 7.9E-09 | 0.00791752 | 0.01099553 |
| set4 | BT2325 | 0.00042508 | 0.000186062 | 1.43E-15 | 4.31E-13 | 0.492002 | 0.2008954 |
| set4 | BT2329 | 0.000003 | 0.00000478 | 0.00000674 | 0.0000119 | 0.0814235 | 0.05350184 |
| set4 | BT2332 | 0.00000718 | 0.0000084 | 0.000000166 | 0.000000543 | 0.00817929 | 0.01117927 |
| set4 | BT2341 | 0.000945348 | 0.000369385 | 0.000000204 | 0.000000642 | 0.0150189 | 0.01672192 |
| set4 | BT2346 | 0.0107243 | 0.003100387 | 9.55E-08 | 0.000000348 | 0.475463 | 0.1966586 |
| set4 | BT2354 | 0.0020394 | 0.000712624 | 0.000757771 | 0.000630756 | 0.0402107 | 0.03228996 |
| set4 | BT2358 | 0.00020419 | 0.000102551 | 0.00961232 | 0.005759526 | 0.284501 | 0.1349167 |
| set4 | BT2362 | 0.0040687 | 0.001312076 | 0.00981618 | 0.005872936 | 0.143985 | 0.08081972 |
| set4 | BT2372 | 0.000654108 | 0.000267605 | 0.000181814 | 0.000189821 | 0.474717 | 0.1965161 |
| set4 | BT2385 | 0.00775017 | 0.002327701 | 8.29515E-05 | 0.0000981 | 0.22872 | 0.1153754 |
| set4 | BT2386 | 1.59358E-05 | 0.0000147 | 7.22884E-05 | 0.0000875 | 0.120528 | 0.07066237 |
| set4 | BT2396 | 0.00965287 | 0.002829546 | 0.00107681 | 0.000858003 | 0.435746 | 0.1843343 |
| set4 | BT2402 | 2.51883E-05 | 0.0000204 | 1.89434E-05 | 0.0000284 | 0.253723 | 0.1242311 |
| set4 | BT2403 | 0.000565124 | 0.00023644 | 1.26E-10 | 1.85E-09 | 0.0124239 | 0.01479098 |
| set4 | BT2405 | 8.55564E-05 | 0.0000516 | 0.00126717 | 0.000985085 | 0.0136016 | 0.01565965 |
| set4 | BT2413 | 0.0204964 | 0.005556211 | 0.000000363 | 0.00000103 | 0.0668245 | 0.04662213 |
| set4 | BT2421 | 0.00114286 | 0.000434868 | 0.00303426 | 0.002104039 | 0.790462 | 0.281135 |
| set4 | BT2422 | 2.67E-09 | 0.000000196 | 0.00237197 | 0.001704475 | 0.203215 | 0.1048517 |
| set4 | BT2423 | 0.00000263 | 0.00000435 | 0.000216616 | 0.000219331 | 0.0528754 | 0.03931956 |
| set4 | BT2424 | 0.0000044 | 0.00000603 | 0.00902611 | 0.005462471 | 0.570868 | 0.2228692 |
| set4 | BT2425 | 0.000000104 | 0.000000843 | 0.00746568 | 0.00466779 | 0.798986 | 0.2833648 |
| set4 | BT2429 | 0.000000518 | 0.00000175 | 0.0034305 | 0.002335893 | 0.0606758 | 0.04347655 |
| set4 | BT2433 | 0.0184364 | 0.005057559 | 0.000404939 | 0.000371448 | 0.0156109 | 0.01707781 |
| set4 | BT2435 | 0.0129792 | 0.003698592 | 0.00661014 | 0.004198066 | 0.631831 | 0.2395343 |
| set4 | BT2439 | 0.00201875 | 0.000705734 | 0.00258675 | 0.001835879 | 0.0637203 | 0.04507771 |
| set4 | BT2440 | 0.000000667 | 0.00000202 | 0.00000586 | 0.0000107 | 0.620619 | 0.2366282 |
| set4 | BT2441 | 0.0388636 | 0.009863974 | 8.55E-08 | 0.000000318 | 0.323614 | 0.1482626 |
| set4 | BT2442 | 0.000395316 | 0.000174971 | 0.000415399 | 0.000380322 | 0.0203812 | 0.02040207 |
| set4 | BT2444 | 0.00737848 | 0.002226383 | 0.000138809 | 0.000151985 | 0.00854939 | 0.01146102 |
| set4 | BT2446 | 8.49112E-05 | 0.0000513 | 5.98E-08 | 0.000000239 | 0.498697 | 0.2026057 |
| set4 | BT2447 | 1.89536E-05 | 0.0000166 | 3.91E-09 | 2.57E-08 | 0.0981955 | 0.06133747 |
| set4 | BT2461 | 0.000407989 | 0.000179568 | 2.79612E-05 | 0.0000394 | 0.0195168 | 0.01975799 |
| set4 | BT2462 | 0.000000129 | 0.000000919 | 0.000000327 | 0.00000094 | 0.408227 | 0.1754741 |
| set4 | BT2463 | 0.00000918 | 0.00000996 | 0.003209 | 0.002209984 | 0.0217934 | 0.02131325 |
| set4 | BT2469 | 0.00107478 | 0.000412296 | 7.32445E-05 | 0.0000885 | 0.804524 | 0.2843674 |
| set4 | BT2473 | 0.021364 | 0.005776918 | 5E-12 | 1.63E-10 | 0.847776 | 0.2948973 |
| set4 | BT2490 | 0.0130656 | 0.003721811 | 0.0176639 | 0.009694281 | 0.0876123 | 0.05618513 |
| set4 | BT2495 | 0.000000162 | 0.000001 | 0.000043009 | 0.0000566 | 0.0481434 | 0.03659199 |
| set4 | BT2497 | 0.00848543 | 0.002518548 | 0.000375443 | 0.000350206 | 0.918795 | 0.3108595 |
| set4 | BT2505 | 0.000295096 | 0.000138295 | 3.01773E-05 | 0.0000419 | 0.311544 | 0.1439929 |
| set4 | BT2506 | 0.0253951 | 0.006744063 | 0.000000886 | 0.00000216 | 0.0674355 | 0.04684802 |
| set4 | BT2509 | 0.0114651 | 0.003299428 | 0.0140255 | 0.007995331 | 0.048849 | 0.03707672 |
| set4 | BT2511 | 0.00312307 | 0.001037635 | 7.04E-09 | 4.03E-08 | 0.0220505 | 0.02144708 |
| set4 | BT2513 | 0.00272983 | 0.000924845 | 0.00000412 | 0.00000803 | 0.819464 | 0.2879834 |
| set4 | BT2520 | 0.000000192 | 0.00000105 | 0.000119008 | 0.000133107 | 0.0123743 | 0.01475587 |
| set4 | BT2526 | 0.00042753 | 0.000186825 | 0.00128617 | 0.000996555 | 0.494856 | 0.2015562 |
| set4 | BT2527 | 0.000351074 | 0.000159397 | 0.00318926 | 0.002197643 | 0.138626 | 0.07850762 |
| set4 | BT2530 | 0.00887819 | 0.002621757 | 0.00052465 | 0.000461245 | 0.804258 | 0.2843674 |
| set4 | BT2546 | 0.0148516 | 0.00416473 | 0.000027668 | 0.000039 | 0.0516772 | 0.03858492 |
| set4 | BT2547 | 0.00592224 | 0.001836263 | 0.000614824 | 0.000529727 | 0.918649 | 0.3108595 |
| set4 | BT2561 | 0.00000372 | 0.00000551 | 0.000367118 | 0.000343768 | 0.594157 | 0.2292825 |
| set4 | BT2564 | 0.000724927 | 0.000292001 | 0.00218366 | 0.001591885 | 0.0825112 | 0.05402813 |
| set4 | BT2566 | 0.0156266 | 0.004364271 | 0.000268462 | 0.000263008 | 0.371868 | 0.1640079 |
| set4 | BT2567 | 2.18048E-05 | 0.0000184 | 0.000000059 | 0.000000238 | 0.00841118 | 0.01135072 |
| set4 | BT2572 | 9.97306E-05 | 0.0000582 | 0.00236405 | 0.001702835 | 0.0695327 | 0.04795641 |
| set4 | BT2579 | 0.0226705 | 0.006079667 | 8.02953E-05 | 0.0000953 | 0.0177271 | 0.01846111 |
| set4 | BT2583 | 0.00131958 | 0.000491264 | 0.000387209 | 0.000358643 | 0.110531 | 0.06671487 |
| set4 | BT2588 | 0.00212061 | 0.000737596 | 0.00122527 | 0.000958592 | 0.537961 | 0.2135346 |
| set4 | BT2592 | 0.000210383 | 0.000105103 | 6.71E-10 | 6.69E-09 | 0.479733 | 0.1979784 |
| set4 | BT2600 | 0.00122524 | 0.000460217 | 9.63E-08 | 0.000000348 | 0.473333 | 0.1960538 |
| set4 | BT2602 | 0.000107814 | 0.0000617 | 0.0102042 | 0.006066027 | 0.0103858 | 0.01318816 |
| set4 | BT2603 | 0.00000165 | 0.00000342 | 7.77874E-05 | 0.0000929 | 0.410254 | 0.1760876 |
| set4 | BT2604 | 0.000874922 | 0.000345974 | 0.000280371 | 0.000272246 | 0.00816674 | 0.01117927 |
| set4 | BT2612 | 0.000195031 | 0.0000988 | 0.0119961 | 0.006986833 | 0.024432 | 0.02284344 |
| set4 | BT2614 | 5.83096E-05 | 0.0000382 | 7.55E-08 | 0.000000292 | 0.0253726 | 0.02338037 |
| set4 | BT2618 | 0.00269307 | 0.000914438 | 0.00431967 | 0.002862286 | 0.067413 | 0.04684802 |
| set4 | BT2620 | 0.0348546 | 0.008921242 | 0.00732075 | 0.004596207 | 0.0691312 | 0.04775483 |
| set4 | BT2631 | 0.00349613 | 0.001145988 | 0.00000354 | 0.000007 | 0.0428191 | 0.03372048 |
| set4 | BT2634 | 3.83872E-05 | 0.0000281 | 0.00778435 | 0.004834503 | 0.00726748 | 0.01045889 |
| set4 | BT2635 | 0.000298241 | 0.000139424 | 0.00867904 | 0.005284198 | 0.0119254 | 0.01439599 |
| set4 | BT2647 | 0.000194729 | 0.0000988 | 0.00022913 | 0.00022931 | 0.0234091 | 0.02222682 |
| set4 | BT2648 | 0.000305968 | 0.000142245 | 0.000000243 | 0.000000738 | 0.274431 | 0.1318365 |
| set4 | BT2677 | 3.31568E-05 | 0.0000252 | 0.00837326 | 0.005129048 | 0.0160604 | 0.0174374 |
| set4 | BT2678 | 0.00000124 | 0.00000291 | 0.000000496 | 0.00000133 | 0.102688 | 0.0634685 |
| set4 | BT2687 | 0.000694526 | 0.000281704 | 0.000540158 | 0.000473501 | 0.0986801 | 0.06156158 |
| set4 | BT2688 | 0.00755045 | 0.002274034 | 0.000377699 | 0.000351879 | 0.320725 | 0.1472165 |
| set4 | BT2689 | 0.021607 | 0.005834289 | 0.00000953 | 0.0000159 | 0.0379442 | 0.03108204 |
| set4 | BT2694 | 0.00329447 | 0.001086474 | 0.00821212 | 0.005045693 | 0.0408576 | 0.03264869 |
| set4 | BT2695 | 0.000323035 | 0.000148538 | 0.000498747 | 0.00044112 | 0.0969218 | 0.06077461 |
| set4 | BT2729 | 0.00336607 | 0.001106796 | 0.0129736 | 0.007466157 | 0.0537297 | 0.03969343 |
| set4 | BT2733 | 0.0288194 | 0.007537077 | 1.64824E-05 | 0.0000255 | 0.912601 | 0.3095408 |
| set4 | BT2745 | 4.57784E-05 | 0.000032 | 4.20121E-05 | 0.0000555 | 0.548047 | 0.2163185 |
| set4 | BT2749 | 0.00000453 | 0.00000615 | 0.000000439 | 0.0000012 | 0.0123524 | 0.01475372 |
| set4 | BT2756 | 0.0116508 | 0.003349048 | 0.0174412 | 0.009585124 | 0.0534827 | 0.0395751 |
| set4 | BT2762 | 2.29973E-05 | 0.0000191 | 0.00184157 | 0.001372316 | 0.00779958 | 0.01089925 |
| set4 | BT2767 | 0.00311672 | 0.00103598 | 0.000473018 | 0.000421373 | 0.0334894 | 0.02845903 |
| set4 | BT2769 | 0.000285361 | 0.000134734 | 0.000214814 | 0.000218302 | 0.210715 | 0.1080002 |
| set4 | BT2775 | 0.00205223 | 0.000716446 | 1.01038E-05 | 0.0000168 | 0.0237729 | 0.02241968 |
| set4 | BT2783 | 0.0168268 | 0.004658215 | 7.82462E-05 | 0.0000933 | 0.67045 | 0.250233 |
| set4 | BT2786 | 4.52532E-05 | 0.0000317 | 0.00484156 | 0.003178459 | 0.48929 | 0.2002895 |
| set4 | BT2790 | 0.000000891 | 0.00000243 | 0.00192119 | 0.001421999 | 0.741335 | 0.2687626 |
| set4 | BT2794 | 0.00666275 | 0.00203502 | 0.000252264 | 0.000249977 | 0.0760856 | 0.05109218 |
| set4 | BT2795 | 0.000301448 | 0.000140576 | 0.000000105 | 0.000000376 | 0.0182871 | 0.01891754 |
| set4 | BT2797 | 0.0230612 | 0.006180644 | 0.00937294 | 0.005638469 | 0.0199545 | 0.02007649 |
| set4 | BT2798 | 0.000225628 | 0.000111178 | 0.00570023 | 0.0036645 | 0.0215919 | 0.02128168 |
| set4 | BT2809 | 0.000873638 | 0.000345647 | 0.00868463 | 0.005284938 | 0.629339 | 0.2390217 |
| set4 | BT2814 | 0.000277552 | 0.000132036 | 9.01596E-05 | 0.000105124 | 0.0233113 | 0.02216263 |
| set4 | BT2815 | 0.000261685 | 0.000125992 | 0.00123749 | 0.000966274 | 0.0451384 | 0.03498244 |
| set4 | BT2822 | 8.33E-09 | 0.000000225 | 0.000375119 | 0.000350174 | 0.0464868 | 0.03568978 |
| set4 | BT2823 | 0.000000134 | 0.000000919 | 4.27987E-05 | 0.0000564 | 0.605049 | 0.2323174 |
| set4 | BT2832 | 0.000739566 | 0.000296865 | 0.00545066 | 0.003526581 | 0.0538718 | 0.03974579 |
| set4 | BT2837 | 0.00013079 | 0.0000713 | 0.00888013 | 0.005382216 | 0.936105 | 0.3151038 |
| set4 | BT2838 | 5.51496E-05 | 0.0000366 | 0.0172843 | 0.009514631 | 0.119871 | 0.07032928 |
| set4 | BT2845 | 0.0379583 | 0.009650381 | 0.000195664 | 0.000201258 | 0.637261 | 0.2408464 |
| set4 | BT2848 | 0.0162269 | 0.004505034 | 0.00031833 | 0.000305422 | 0.169724 | 0.09162968 |
| set4 | BT2855 | 0.00016674 | 0.0000864 | 0.000526488 | 0.000462525 | 0.0269132 | 0.0243266 |
| set4 | BT2861 | 0.000000176 | 0.000001 | 0.000000532 | 0.00000141 | 0.00712528 | 0.01034742 |
| set4 | BT2866 | 1.24045E-05 | 0.0000124 | 0.0028959 | 0.002021359 | 0.394386 | 0.1713815 |
| set4 | BT2871 | 0.000294234 | 0.000137977 | 6.50787E-05 | 0.0000804 | 0.0315935 | 0.02742561 |
| set4 | BT2873 | 0.0368158 | 0.009385136 | 0.000437502 | 0.000395569 | 0.0763757 | 0.0512401 |
| set4 | BT2875 | 0.00300208 | 0.001001834 | 0.00285662 | 0.001998347 | 0.0361961 | 0.02995093 |
| set4 | BT2876 | 0.000481506 | 0.000206361 | 0.00294317 | 0.002046749 | 0.238929 | 0.1186096 |
| set4 | BT2880 | 0.000000649 | 0.00000202 | 0.00224425 | 0.00162624 | 0.0179868 | 0.01865948 |
| set4 | BT2882 | 0.00057444 | 0.000239544 | 0.00259639 | 0.001841638 | 0.0634835 | 0.04495352 |
| set4 | BT2884 | 0.000000064 | 0.000000654 | 0.000000602 | 0.00000156 | 0.0201609 | 0.02022869 |
| set4 | BT2890 | 0.0031025 | 0.001031706 | 8.80864E-05 | 0.000103305 | 0.547661 | 0.2162796 |
| set4 | BT2892 | 0.0218391 | 0.005892755 | 0.000690624 | 0.000583793 | 0.631003 | 0.239344 |
| set4 | BT2893 | 0.00332258 | 0.001094314 | 0.0034285 | 0.002335893 | 0.613109 | 0.2343735 |
| set4 | BT2894 | 0.00358011 | 0.001170028 | 0.0035633 | 0.002415426 | 0.59002 | 0.2282218 |
| set4 | BT2895 | 0.01207 | 0.003457729 | 0.0016254 | 0.001227127 | 0.602287 | 0.2317454 |
| set4 | BT2896 | 0.00184793 | 0.00065185 | 5.63495E-05 | 0.0000712 | 0.146682 | 0.08175708 |
| set4 | BT2898 | 0.0023391 | 0.000803985 | 0.00954126 | 0.005719786 | 0.969898 | 0.322403 |
| set4 | BT2905 | 0.0014546 | 0.000533397 | 8.27E-08 | 0.000000314 | 0.110416 | 0.06670031 |
| set4 | BT2906 | 0.00661745 | 0.002023634 | 2.42E-09 | 1.74E-08 | 0.0330059 | 0.02815223 |
| set4 | BT2912 | 0.0037525 | 0.001219461 | 1.01486E-05 | 0.0000168 | 0.374808 | 0.1649244 |
| set4 | BT2913 | 0.000484247 | 0.000207301 | 0.000066093 | 0.0000814 | 0.0341186 | 0.0288311 |
| set4 | BT2920 | 0.0176679 | 0.004865583 | 0.00086018 | 0.00070349 | 0.0412026 | 0.03281719 |
| set4 | BT2924 | 0.021946 | 0.005917381 | 0.000138765 | 0.000151985 | 0.861347 | 0.2976973 |
| set4 | BT2927 | 3.41261E-05 | 0.0000257 | 0.00000512 | 0.0000096 | 0.593231 | 0.2291912 |
| set4 | BT2932 | 1.60337E-05 | 0.0000147 | 1.79E-09 | 1.41E-08 | 0.00817645 | 0.01117927 |
| set4 | BT2934 | 0.000117927 | 0.0000657 | 1.38E-09 | 1.16E-08 | 0.0200562 | 0.02015118 |
| set4 | BT2941 | 0.00000021 | 0.00000108 | 0.000000266 | 0.000000792 | 0.112307 | 0.06741265 |
| set4 | BT2942 | 0.00000118 | 0.0000028 | 0.00050221 | 0.000443454 | 0.0153725 | 0.01693609 |
| set4 | BT2955 | 0.00146549 | 0.000536351 | 0.00000157 | 0.00000349 | 0.104942 | 0.06437374 |
| set4 | BT2957 | 0.0016218 | 0.00058535 | 0.0103682 | 0.00613634 | 0.0149246 | 0.01664734 |
| set4 | BT2958 | 0.00124472 | 0.000466608 | 0.000177547 | 0.00018617 | 0.0144447 | 0.01633563 |
| set4 | BT2962 | 0.00764241 | 0.002298986 | 0.00000456 | 0.00000875 | 0.287482 | 0.1358662 |
| set4 | BT2965 | 0.000132571 | 0.0000721 | 0.00140149 | 0.001073712 | 0.867571 | 0.2991456 |
| set4 | BT2974 | 0.00841669 | 0.002500106 | 1.47E-09 | 1.21E-08 | 0.0428154 | 0.03372048 |
| set4 | BT2981 | 0.00594746 | 0.001843327 | 0.000000122 | 0.000000424 | 0.0352664 | 0.02946399 |
| set4 | BT2984 | 0.00730174 | 0.002207914 | 1.65769E-05 | 0.0000256 | 0.248507 | 0.1223668 |
| set4 | BT2986 | 0.00000056 | 0.00000186 | 0.00000424 | 0.0000082 | 0.0480081 | 0.03653779 |
| set4 | BT2988 | 5.26537E-05 | 0.0000354 | 6.42353E-05 | 0.0000794 | 0.900376 | 0.3066517 |
| set4 | BT2990 | 0.000733102 | 0.000294823 | 0.00509708 | 0.003320938 | 0.694262 | 0.2566238 |
| set4 | BT2991 | 7.33429E-05 | 0.000046 | 0.00665247 | 0.004218296 | 0.00950514 | 0.01242219 |
| set4 | BT2993 | 0.000200284 | 0.000101038 | 0.0125658 | 0.00726609 | 0.275342 | 0.1321281 |
| set4 | BT2996 | 7.40811E-05 | 0.0000463 | 7.50563E-05 | 0.0000903 | 0.0187043 | 0.01920028 |
| set4 | BT3004 | 0.000143538 | 0.0000762 | 0.00000236 | 0.0000049 | 0.00702196 | 0.01024619 |
| set4 | BT3011 | 0.00147448 | 0.000539075 | 1.14647E-05 | 0.0000188 | 0.713248 | 0.2614208 |
| set4 | BT3016 | 8.05E-08 | 0.000000717 | 1.57179E-05 | 0.0000244 | 0.0115953 | 0.01412528 |
| set4 | BT3023 | 0.018826 | 0.005147644 | 0.00940163 | 0.005652913 | 0.80412 | 0.2843674 |
| set4 | BT3029 | 0.000353619 | 0.000160072 | 0.000136781 | 0.000150341 | 0.253693 | 0.1242311 |
| set4 | BT3030 | 0.00774163 | 0.002326059 | 0.00348402 | 0.00236833 | 0.240935 | 0.1192827 |
| set4 | BT3042 | 1.25348E-05 | 0.0000124 | 0.00314326 | 0.002170899 | 0.393001 | 0.1708808 |
| set4 | BT3047 | 0.0000051 | 0.00000668 | 0.0127597 | 0.007360594 | 0.0224801 | 0.02170985 |
| set4 | BT3052 | 0.0220173 | 0.005930267 | 0.0117552 | 0.006863073 | 0.822131 | 0.2887421 |
| set4 | BT3055 | 0.000000205 | 0.00000108 | 0.00771165 | 0.004799222 | 0.0407198 | 0.03257404 |
| set4 | BT3056 | 0.000138279 | 0.0000743 | 2.22E-08 | 0.000000105 | 0.00909318 | 0.01200364 |
| set4 | BT3060 | 0.00000201 | 0.00000376 | 0.00002135 | 0.0000314 | 0.280967 | 0.1338646 |
| set4 | BT3066 | 0.000100567 | 0.0000585 | 0.0176515 | 0.00969188 | 0.0901335 | 0.05740073 |
| set4 | BT3069 | 0.00000103 | 0.00000259 | 0.00000926 | 0.0000156 | 0.0183306 | 0.01894555 |
| set4 | BT3075 | 7.42842E-05 | 0.0000464 | 0.000229845 | 0.000229836 | 0.0436672 | 0.03420486 |
| set4 | BT3084 | 4.56E-08 | 0.000000576 | 8.25E-10 | 7.79E-09 | 0.635497 | 0.2404274 |
| set4 | BT3085 | 0.00000322 | 0.00000506 | 0.00000277 | 0.00000566 | 0.235966 | 0.1177759 |
| set4 | BT3087 | 0.0102041 | 0.002966997 | 0.000356342 | 0.000335759 | 0.0628224 | 0.04461451 |
| set4 | BT3089 | 6.93955E-05 | 0.0000438 | 1.73E-08 | 8.47E-08 | 0.0225549 | 0.02172491 |
| set4 | BT3107 | 3.13306E-05 | 0.0000241 | 0.00000675 | 0.0000119 | 0.0132473 | 0.01540885 |
| set4 | BT3111 | 0.000265263 | 0.000127229 | 2.93E-09 | 2.04E-08 | 0.0224202 | 0.02168053 |
| set4 | BT3128 | 0.0012294 | 0.000461551 | 0.0127919 | 0.007372128 | 0.282684 | 0.1342904 |
| set4 | BT3130 | 0.00181526 | 0.000643212 | 5.70122E-05 | 0.000072 | 0.0109452 | 0.01365053 |
| set4 | BT3132 | 0.000021895 | 0.0000184 | 0.0141804 | 0.008057017 | 0.0772024 | 0.05165309 |
| set4 | BT3134 | 0.000765254 | 0.000306127 | 0.00802019 | 0.004940332 | 0.0158994 | 0.01728817 |
| set4 | BT3140 | 0.000153437 | 0.0000809 | 0.000087082 | 0.000102226 | 0.610966 | 0.2339298 |
| set4 | BT3142 | 9.80428E-05 | 0.0000573 | 0.00380387 | 0.0025584 | 0.0399346 | 0.03218489 |
| set4 | BT3149 | 0.00298876 | 0.000999151 | 3.30646E-05 | 0.0000454 | 0.00726482 | 0.01045889 |
| set4 | BT3150 | 0.00527468 | 0.001654456 | 0.00445962 | 0.002946936 | 0.0321041 | 0.02770531 |
| set4 | BT3151 | 0.0282704 | 0.007413993 | 0.000000716 | 0.00000179 | 0.982657 | 0.3249518 |
| set4 | BT3152 | 0.000246232 | 0.000119387 | 0.00000023 | 0.000000711 | 0.0240713 | 0.02259087 |
| set4 | BT3155 | 4.22094E-05 | 0.0000301 | 0.00192497 | 0.001423924 | 0.890123 | 0.3042917 |
| set4 | BT3163 | 0.00106084 | 0.000408397 | 0.00000221 | 0.00000464 | 0.26544 | 0.1288507 |
| set4 | BT3166 | 0.0102281 | 0.002972833 | 0.000000933 | 0.00000225 | 0.245758 | 0.121343 |
| set4 | BT3172 | 0.000528261 | 0.000223613 | 0.000317539 | 0.000304906 | 0.0124924 | 0.01483643 |
| set4 | BT3173 | 0.000947743 | 0.000369938 | 0.000626124 | 0.000537546 | 0.0891824 | 0.05696804 |
| set4 | BT3174 | 0.0292242 | 0.007632394 | 0.000157703 | 0.000168881 | 0.0501281 | 0.03761963 |
| set4 | BT3175 | 0.00112052 | 0.000427459 | 1.62E-11 | 3.69E-10 | 0.158434 | 0.08690893 |
| set4 | BT3180 | 0.00780677 | 0.002341913 | 6.89972E-05 | 0.0000843 | 0.107547 | 0.06558799 |
| set4 | BT3194 | 0.0265823 | 0.007027288 | 0.00105182 | 0.000839753 | 0.2472 | 0.121891 |
| set4 | BT3198 | 0.00230649 | 0.000795305 | 0.0141798 | 0.008057017 | 0.0114389 | 0.01401618 |
| set4 | BT3199 | 0.00000216 | 0.00000395 | 0.00170704 | 0.001283148 | 0.724731 | 0.2641784 |
| set4 | BT3200 | 8.78634E-05 | 0.0000528 | 4.58828E-05 | 0.0000595 | 0.178505 | 0.09500758 |
| set4 | BT3203 | 0.00000593 | 0.00000741 | 0.00125633 | 0.000979719 | 0.112747 | 0.06755244 |
| set4 | BT3206 | 0.00196495 | 0.000689154 | 8.62986E-05 | 0.000101504 | 0.0500907 | 0.03761963 |
| set4 | BT3215 | 0.032631 | 0.008414704 | 1.35148E-05 | 0.0000215 | 0.745898 | 0.2698836 |
| set4 | BT3216 | 4.26313E-05 | 0.0000303 | 0.00000476 | 0.00000903 | 0.0105012 | 0.0132433 |
| set4 | BT3224 | 0.00169286 | 0.000606084 | 7.15E-13 | 3.6E-11 | 0.732459 | 0.266334 |
| set4 | BT3225 | 0.00258697 | 0.000881972 | 8.52E-08 | 0.000000318 | 0.0704275 | 0.0483771 |
| set4 | BT3243 | 0.0361342 | 0.009223809 | 0.000348628 | 0.000329701 | 0.46112 | 0.1923099 |
| set4 | BT3249 | 0.0131581 | 0.003743933 | 0.00889307 | 0.005387355 | 0.130209 | 0.07498481 |
| set4 | BT3255 | 0.00069958 | 0.000283147 | 0.000223298 | 0.000224965 | 0.573654 | 0.2235648 |
| set4 | BT3266 | 0.00114437 | 0.000435023 | 0.000000107 | 0.000000382 | 0.0367898 | 0.03033186 |
| set4 | BT3270 | 0.00000739 | 0.00000855 | 0.00000141 | 0.00000317 | 0.0130104 | 0.0152177 |
| set4 | BT3271 | 0.00140273 | 0.000516629 | 0.0108188 | 0.006381139 | 0.36594 | 0.1623738 |
| set4 | BT3276 | 0.000080447 | 0.0000495 | 0.00775957 | 0.004821592 | 0.028789 | 0.02562761 |
| set4 | BT3277 | 0.0138635 | 0.003921058 | 0.0171872 | 0.009475711 | 0.079585 | 0.05264729 |
| set4 | BT3286 | 1.05305E-05 | 0.0000109 | 0.000110349 | 0.000125043 | 0.0144855 | 0.01636916 |
| set4 | BT3295 | 0.0109588 | 0.003162141 | 6.75835E-05 | 0.000083 | 0.114281 | 0.06816549 |
| set4 | BT3297 | 0.000109968 | 0.0000626 | 0.000000238 | 0.000000726 | 0.0981027 | 0.06133746 |
| set4 | BT3298 | 0.0000019 | 0.00000364 | 1.60436E-05 | 0.0000248 | 0.843444 | 0.2937419 |
| set4 | BT3304 | 0.000424959 | 0.000186062 | 4.44376E-05 | 0.000058 | 0.0399483 | 0.03218489 |
| set4 | BT3314 | 0.0279065 | 0.007326175 | 0.00164968 | 0.001242404 | 0.497714 | 0.2023273 |
| set4 | BT3315 | 0.00000838 | 0.00000939 | 1.49275E-05 | 0.0000234 | 0.0404383 | 0.03243724 |
| set4 | BT3316 | 0.00597478 | 0.001850279 | 5.69E-08 | 0.000000232 | 0.184436 | 0.0974225 |
| set4 | BT3319 | 0.000181014 | 0.0000926 | 0.00767023 | 0.004778369 | 0.327726 | 0.1497277 |
| set4 | BT3320 | 4.74046E-05 | 0.0000328 | 0.0148214 | 0.008366128 | 0.110342 | 0.06668305 |
| set4 | BT3321 | 0.000429002 | 0.000187252 | 2.83982E-05 | 0.0000399 | 0.0125925 | 0.01490328 |
| set4 | BT3326 | 0.000134175 | 0.0000726 | 0.000119576 | 0.000133619 | 0.118079 | 0.0697823 |
| set4 | BT3333 | 0.000157385 | 0.0000825 | 0.00000135 | 0.00000307 | 0.0115297 | 0.01407689 |
| set4 | BT3334 | 0.00103524 | 0.000399355 | 0.000469798 | 0.000419433 | 0.144546 | 0.08107827 |
| set4 | BT3339 | 0.0293774 | 0.007666245 | 0.0128568 | 0.007405997 | 0.804525 | 0.2843674 |
| set4 | BT3342 | 2.19166E-05 | 0.0000184 | 0.0011088 | 0.00088001 | 0.0383498 | 0.03132579 |
| set4 | BT3362 | 0.010719 | 0.003100039 | 6.17149E-05 | 0.0000766 | 0.0707871 | 0.04854577 |
| set4 | BT3363 | 0.00287551 | 0.000970291 | 0.00268734 | 0.001897237 | 0.318789 | 0.1466029 |
| set4 | BT3366 | 0.00352883 | 0.001155706 | 5.72E-09 | 3.46E-08 | 0.0154151 | 0.0169499 |
| set4 | BT3378 | 0.0181158 | 0.004978633 | 3.90889E-05 | 0.0000519 | 0.433767 | 0.1836495 |
| set4 | BT3381 | 0.01471 | 0.004132875 | 0.00197806 | 0.001460512 | 0.446952 | 0.1877223 |
| set4 | BT3382 | 1.21163E-05 | 0.0000121 | 1.72131E-05 | 0.0000263 | 0.0213225 | 0.02107724 |
| set4 | BT3383 | 6.36886E-05 | 0.000041 | 0.000500559 | 0.000442319 | 0.281926 | 0.1340814 |
| set4 | BT3389 | 0.00853954 | 0.002530214 | 0.000415852 | 0.000380322 | 0.0121684 | 0.01459332 |
| set4 | BT3390 | 2.36457E-05 | 0.0000195 | 2.56E-08 | 0.000000118 | 0.067407 | 0.04684802 |
| set4 | BT3394 | 0.000303107 | 0.000141175 | 0.000000149 | 0.000000506 | 0.232201 | 0.1165307 |
| set4 | BT3412 | 0.0161183 | 0.004483397 | 0.000228524 | 0.000229254 | 0.046813 | 0.03584371 |
| set4 | BT3417 | 0.00150837 | 0.000549123 | 0.0127884 | 0.007372128 | 0.238901 | 0.1186096 |
| set4 | BT3418 | 0.012727 | 0.003634135 | 1.66E-08 | 8.24E-08 | 0.770307 | 0.276129 |
| set4 | BT3421 | 0.000159077 | 0.0000831 | 0.0122692 | 0.007125281 | 0.864734 | 0.2986026 |
| set4 | BT3423 | 0.0331641 | 0.008531828 | 0.00000012 | 0.00000042 | 0.0896394 | 0.05716046 |
| set4 | BT3432 | 0.000200628 | 0.000101038 | 0.00306485 | 0.002122813 | 0.357651 | 0.1594299 |
| set4 | BT3433 | 0.00209498 | 0.000730023 | 0.00929485 | 0.005602985 | 0.191117 | 0.1001229 |
| set4 | BT3442 | 0.00028778 | 0.000135623 | 0.00276225 | 0.001943309 | 0.111661 | 0.06714437 |
| set4 | BT3445 | 0.000000566 | 0.00000187 | 0.0123477 | 0.007163981 | 0.0231769 | 0.02204913 |
| set4 | BT3450 | 2.36E-08 | 0.000000385 | 0.00413544 | 0.002750774 | 0.107644 | 0.06561989 |
| set4 | BT3451 | 0.00000221 | 0.00000398 | 0.000218901 | 0.000221459 | 0.0453209 | 0.03503287 |
| set4 | BT3453 | 0.00159303 | 0.000576616 | 0.00190045 | 0.001411838 | 0.915911 | 0.3102892 |
| set4 | BT3454 | 0.000078724 | 0.0000488 | 0.00254849 | 0.001812075 | 0.530855 | 0.2116218 |
| set4 | BT3455 | 1.31592E-05 | 0.0000128 | 0.00000221 | 0.00000464 | 0.746608 | 0.2700664 |
| set4 | BT3459 | 8.45893E-05 | 0.0000513 | 0.000288103 | 0.000279305 | 0.169555 | 0.09160582 |
| set4 | BT3461 | 1.47829E-05 | 0.0000139 | 0.000332994 | 0.000317725 | 0.0154492 | 0.01697468 |
| set4 | BT3472 | 0.00100429 | 0.000388841 | 0.00109034 | 0.000867068 | 0.00732473 | 0.01052066 |
| set4 | BT3477 | 0.0014874 | 0.000542795 | 0.000659921 | 0.000561375 | 0.385535 | 0.1684829 |
| set4 | BT3481 | 0.0136582 | 0.003873124 | 0.000000567 | 0.00000148 | 0.912862 | 0.3095408 |
| set4 | BT3487 | 0.0146692 | 0.00412581 | 0.00382606 | 0.002569033 | 0.0777293 | 0.05186378 |
| set4 | BT3493 | 0.000337469 | 0.000154238 | 0.00126817 | 0.000985085 | 0.722766 | 0.2637399 |
| set4 | BT3494 | 0.00142649 | 0.000524614 | 0.000528174 | 0.000463669 | 0.0120472 | 0.01449534 |
| set4 | BT3495 | 0.0000079 | 0.00000903 | 0.000459446 | 0.000412078 | 0.00721081 | 0.01040937 |
| set4 | BT3514 | 0.0259279 | 0.006868686 | 0.00000769 | 0.0000134 | 0.76235 | 0.2740799 |
| set4 | BT3515 | 2.00347E-05 | 0.0000173 | 0.000128216 | 0.00014209 | 0.019121 | 0.01949172 |
| set4 | BT3527 | 0.0154973 | 0.004331356 | 0.00324237 | 0.002226618 | 0.0673324 | 0.04684485 |
| set4 | BT3532 | 0.00154629 | 0.000562387 | 0.000000409 | 0.00000113 | 0.210677 | 0.1080002 |
| set4 | BT3543 | 0.000125705 | 0.0000691 | 0.000681159 | 0.000577003 | 0.977442 | 0.3235509 |
| set4 | BT3547 | 0.00119675 | 0.000451758 | 0.000177065 | 0.000185826 | 0.386535 | 0.1687483 |
| set4 | BT3550 | 0.00137337 | 0.000507297 | 0.00504256 | 0.00329252 | 0.256322 | 0.1252113 |
| set4 | BT3551 | 2.46925E-05 | 0.0000201 | 1.20339E-05 | 0.0000196 | 0.175502 | 0.09388584 |
| set4 | BT3553 | 3.45658E-05 | 0.000026 | 0.00000232 | 0.00000484 | 0.0140489 | 0.01598112 |
| set4 | BT3556 | 9.10348E-05 | 0.0000543 | 0.00265262 | 0.001876015 | 0.245878 | 0.1213615 |
| set4 | BT3563 | 0.000000972 | 0.00000256 | 0.00789943 | 0.004885878 | 0.13017 | 0.07498481 |
| set4 | BT3564 | 0.00000467 | 0.00000631 | 1.89783E-05 | 0.0000284 | 0.0884961 | 0.05665291 |
| set4 | BT3571 | 0.00146036 | 0.000534732 | 0.00592206 | 0.003786953 | 0.375664 | 0.1651752 |
| set4 | BT3575 | 0.00883626 | 0.002610393 | 0.000261671 | 0.000257819 | 0.213768 | 0.1092979 |
| set4 | BT3585 | 0.00000271 | 0.00000442 | 0.000378438 | 0.000352184 | 0.0750593 | 0.05063464 |
| set4 | BT3586 | 0.000922532 | 0.000361777 | 0.0067692 | 0.004290061 | 0.0498526 | 0.03754725 |
| set4 | BT3595 | 0.00578668 | 0.001797175 | 0.00123038 | 0.000961344 | 0.248617 | 0.1223668 |
| set4 | BT3606 | 0.00688877 | 0.002099817 | 0.00345288 | 0.002348484 | 0.60449 | 0.232167 |
| set4 | BT3612 | 0.000168798 | 0.0000872 | 0.00701266 | 0.00443041 | 0.00733238 | 0.01052135 |
| set4 | BT3614 | 0.000316805 | 0.000146295 | 0.0172779 | 0.009514631 | 0.387545 | 0.1690091 |
| set4 | BT3617 | 0.00211797 | 0.000737355 | 0.000180834 | 0.000189288 | 0.0912221 | 0.05794317 |
| set4 | BT3621 | 0.0039396 | 0.00127588 | 3.48032E-05 | 0.0000472 | 0.710563 | 0.2607622 |
| set4 | BT3622 | 0.00000241 | 0.00000414 | 0.0143276 | 0.008125366 | 0.0108138 | 0.01353527 |
| set4 | BT3624 | 0.000595398 | 0.000246788 | 0.00156957 | 0.001189437 | 0.734227 | 0.2667421 |
| set4 | BT3625 | 0.00010276 | 0.0000593 | 1.44584E-05 | 0.0000227 | 0.0248489 | 0.02304244 |
| set4 | BT3629 | 0.00524361 | 0.001646075 | 0.000161084 | 0.000171287 | 0.0350268 | 0.02934732 |
| set4 | BT3640 | 1.94426E-05 | 0.0000169 | 1.60433E-05 | 0.0000248 | 0.107227 | 0.06548055 |
| set4 | BT3641 | 0.000313586 | 0.000144897 | 0.00000822 | 0.0000141 | 0.0368348 | 0.0303426 |
| set4 | BT3642 | 0.0266495 | 0.007040135 | 0.0166435 | 0.009201154 | 0.0134538 | 0.01557499 |
| set4 | BT3651 | 3.59E-08 | 0.000000476 | 3.05745E-05 | 0.0000424 | 0.417964 | 0.1787696 |
| set4 | BT3663 | 0.0261138 | 0.006915513 | 0.00358036 | 0.002425629 | 0.0493377 | 0.03733183 |
| set4 | BT3670 | 0.000477504 | 0.000204762 | 0.000111029 | 0.000125578 | 0.639726 | 0.2415293 |
| set4 | BT3673 | 0.00399983 | 0.001292621 | 4.01E-08 | 0.000000174 | 0.350076 | 0.1571508 |
| set4 | BT3674 | 7.38038E-05 | 0.0000462 | 0.000000224 | 0.000000696 | 0.0639169 | 0.04517325 |
| set4 | BT3676 | 0.0131239 | 0.003737012 | 0.00184093 | 0.001372316 | 0.0582984 | 0.04215632 |
| set4 | BT3679 | 0.00148338 | 0.00054185 | 0.0120643 | 0.007021822 | 0.134809 | 0.07684978 |
| set4 | BT3681 | 0.00000851 | 0.00000945 | 0.0111778 | 0.006570427 | 0.0992635 | 0.06183278 |
| set4 | BT3686 | 0.000076081 | 0.0000472 | 0.000203991 | 0.000208823 | 0.00687976 | 0.01007877 |
| set4 | BT3697 | 0.0029845 | 0.000998609 | 5.82978E-05 | 0.0000733 | 0.49247 | 0.2010306 |
| set4 | BT3715 | 0.000252329 | 0.000121797 | 0.00441625 | 0.002919874 | 0.191952 | 0.1004886 |
| set4 | BT3721 | 0.000871377 | 0.000345294 | 0.0137156 | 0.007848301 | 0.00841299 | 0.01135072 |
| set4 | BT3724 | 0.000888732 | 0.000350445 | 0.00116278 | 0.000915633 | 0.697496 | 0.2572532 |
| set4 | BT3728 | 0.000219509 | 0.000108587 | 0.0150243 | 0.008453008 | 0.0245461 | 0.02287726 |
| set4 | BT3741 | 2.06468E-05 | 0.0000176 | 0.000000163 | 0.000000542 | 0.347539 | 0.1562988 |
| set4 | BT3744 | 0.0159104 | 0.004433338 | 0.00016453 | 0.000174184 | 0.11233 | 0.06741265 |
| set4 | BT3749 | 0.00000129 | 0.00000297 | 4.08E-09 | 2.65E-08 | 0.59922 | 0.2309285 |
| set4 | BT3751 | 0.0342174 | 0.008784885 | 0.00000905 | 0.0000153 | 0.115555 | 0.06868257 |
| set4 | BT3762 | 0.00652862 | 0.001999702 | 0.0151102 | 0.00848658 | 0.511151 | 0.2065313 |
| set4 | BT3774 | 0.000295351 | 0.000138329 | 0.00712113 | 0.004491891 | 0.0578928 | 0.04192499 |
| set4 | BT3794 | 0.00215436 | 0.000747617 | 0.00000498 | 0.0000094 | 0.633537 | 0.2399951 |
| set4 | BT3801 | 0.0100369 | 0.002925123 | 0.0000017 | 0.00000375 | 0.0491845 | 0.03727355 |
| set4 | BT3802 | 1.76596E-05 | 0.0000159 | 0.000266387 | 0.000261825 | 0.0240874 | 0.02259087 |
| set4 | BT3805 | 0.000001 | 0.00000256 | 0.00033995 | 0.000323086 | 0.0289287 | 0.02568965 |
| set4 | BT3807 | 0.00062728 | 0.000258164 | 3.36732E-05 | 0.0000459 | 0.611514 | 0.2339485 |
| set4 | BT3810 | 0.000920039 | 0.000360986 | 0.00716555 | 0.004515195 | 0.127739 | 0.07385211 |
| set4 | BT3813 | 0.00618015 | 0.001904527 | 0.00461405 | 0.003039009 | 0.804384 | 0.2843674 |
| set4 | BT3821 | 0.000014187 | 0.0000136 | 0.0121213 | 0.007049556 | 0.0346301 | 0.02916476 |
| set4 | BT3822 | 0.00999229 | 0.002916615 | 2.83E-08 | 0.000000129 | 0.805506 | 0.2844634 |
| set4 | BT3834 | 0.000304186 | 0.000141591 | 0.0150347 | 0.008454921 | 0.0134236 | 0.0155523 |
| set4 | BT3836 | 0.01355 | 0.003845323 | 0.000581085 | 0.00050498 | 0.0208323 | 0.02073943 |
| set4 | BT3837 | 0.000006 | 0.00000742 | 2.87537E-05 | 0.0000403 | 0.303247 | 0.1414047 |
| set4 | BT3846 | 0.00495566 | 0.001566075 | 0.000104731 | 0.000119688 | 0.0213138 | 0.02107724 |
| set4 | BT3859 | 0.000000598 | 0.0000019 | 0.0158763 | 0.008837845 | 0.0120783 | 0.01452086 |
| set4 | BT3866 | 0.000504165 | 0.000214735 | 0.00207267 | 0.001521071 | 0.186063 | 0.09807017 |
| set4 | BT3869 | 0.00100144 | 0.000388295 | 1.23871E-05 | 0.0000201 | 0.351352 | 0.1574828 |
| set4 | BT3874 | 0.0176723 | 0.004865583 | 0.0132606 | 0.00760957 | 0.204752 | 0.1054594 |
| set4 | BT3876 | 0.0106015 | 0.003073102 | 0.0142011 | 0.008064985 | 0.093287 | 0.05897406 |
| set4 | BT3877 | 0.00294152 | 0.000986846 | 0.0153959 | 0.008617925 | 0.15832 | 0.08687886 |
| set4 | BT3885 | 0.00000169 | 0.00000345 | 0.0174132 | 0.009574092 | 0.0237395 | 0.02241968 |
| set4 | BT3886 | 2.01865E-05 | 0.0000173 | 4.84059E-05 | 0.0000624 | 0.45481 | 0.1902606 |
| set4 | BT3888 | 0.00143843 | 0.000527962 | 0.000101235 | 0.000116464 | 0.0332404 | 0.02830291 |
| set4 | BT3891 | 1.52479E-05 | 0.0000142 | 0.00132149 | 0.001020648 | 0.0205162 | 0.0205011 |
| set4 | BT3903 | 0.000408918 | 0.000179834 | 0.00019203 | 0.000198428 | 0.107937 | 0.06571663 |
| set4 | BT3904 | 0.000638274 | 0.000262261 | 0.00189938 | 0.001411838 | 0.147989 | 0.0823175 |
| set4 | BT3905 | 0.00706397 | 0.002146306 | 0.000479374 | 0.000426093 | 0.422164 | 0.1800892 |
| set4 | BT3911 | 4.12E-09 | 0.000000208 | 0.00188694 | 0.001403528 | 0.428051 | 0.1819178 |
| set4 | BT3913 | 9.21318E-05 | 0.0000548 | 4.12366E-05 | 0.0000546 | 0.409081 | 0.1757898 |
| set4 | BT3914 | 0.00330262 | 0.001088688 | 0.00000318 | 0.00000637 | 0.946811 | 0.317533 |
| set4 | BT3915 | 5.31108E-05 | 0.0000356 | 0.00178835 | 0.001339267 | 0.439562 | 0.1853077 |
| set4 | BT3922 | 0.000364848 | 0.000164173 | 0.00132504 | 0.001022083 | 0.755445 | 0.272398 |
| set4 | BT3929 | 0.00000878 | 0.00000963 | 0.0115305 | 0.006748193 | 0.0998173 | 0.06211264 |
| set4 | BT3958 | 0.00115293 | 0.000437617 | 0.00059742 | 0.000516944 | 0.999448 | 0.3292434 |
| set4 | BT3959 | 0.0266853 | 0.007044675 | 0.000000676 | 0.00000172 | 0.712099 | 0.2611301 |
| set4 | BT3966 | 0.00000432 | 0.00000596 | 0.00158315 | 0.001198225 | 0.029085 | 0.02578547 |
| set4 | BT3970 | 2.06029E-05 | 0.0000176 | 0.000132021 | 0.000145906 | 0.0634559 | 0.04495352 |
| set4 | BT3974 | 0.00000425 | 0.00000592 | 0.000000221 | 0.000000687 | 0.420034 | 0.1793679 |
| set4 | BT3975 | 0.00000733 | 0.00000851 | 2.47125E-05 | 0.0000356 | 0.0417606 | 0.03304646 |
| set4 | BT3976 | 0.000244695 | 0.000118794 | 0.00164975 | 0.001242404 | 0.120694 | 0.07072616 |
| set4 | BT3977 | 0.00101581 | 0.00039266 | 0.000000201 | 0.000000638 | 0.562531 | 0.2204351 |
| set4 | BT3983 | 0.00323749 | 0.001070949 | 0.00918663 | 0.005548496 | 0.270921 | 0.1306901 |
| set4 | BT3990 | 0.00005467 | 0.0000364 | 0.000363889 | 0.000341804 | 0.0401669 | 0.03228996 |
| set4 | BT3997 | 0.000186718 | 0.0000952 | 0.00000401 | 0.00000785 | 0.00738793 | 0.01057458 |
| set4 | BT4003 | 0.000677681 | 0.000275907 | 0.000140077 | 0.000153128 | 0.788242 | 0.2807754 |
| set4 | BT4007 | 0.000311506 | 0.0001442 | 0.000111486 | 0.000125944 | 0.0975797 | 0.06113491 |
| set4 | BT4010 | 0.000428256 | 0.000187034 | 1.92018E-05 | 0.0000287 | 0.0326876 | 0.02801095 |
| set4 | BT4016 | 0.00207866 | 0.000724935 | 0.000116971 | 0.000131072 | 0.171784 | 0.09233432 |
| set4 | BT4017 | 0.00000224 | 0.000004 | 3.17E-09 | 2.17E-08 | 0.137304 | 0.07784902 |
| set4 | BT4022 | 0.000013926 | 0.0000134 | 0.000000929 | 0.00000224 | 0.0412622 | 0.03283121 |
| set4 | BT4026 | 0.00000221 | 0.00000398 | 0.000000013 | 6.64E-08 | 0.0068171 | 0.009999711 |
| set4 | BT4027 | 3.78785E-05 | 0.0000278 | 0.00000134 | 0.00000306 | 0.0103536 | 0.01317934 |
| set4 | BT4036 | 4.51E-08 | 0.000000576 | 5.43505E-05 | 0.0000691 | 0.144856 | 0.0811901 |
| set4 | BT4038 | 0.000546812 | 0.000229795 | 0.00779877 | 0.004840016 | 0.207504 | 0.1066897 |
| set4 | BT4040 | 0.000284198 | 0.00013444 | 0.0150203 | 0.008453008 | 0.238689 | 0.1186096 |
| set4 | BT4048 | 1.11225E-05 | 0.0000114 | 0.00349369 | 0.002373568 | 0.00827395 | 0.01124582 |
| set4 | BT4050 | 0.00000207 | 0.00000383 | 6.23835E-05 | 0.0000774 | 0.395113 | 0.1714636 |
| set4 | BT4071 | 0.0151588 | 0.004244586 | 0.00000315 | 0.00000632 | 0.0477867 | 0.03642108 |
| set4 | BT4072 | 0.00534284 | 0.00167237 | 0.000035344 | 0.0000478 | 0.0253205 | 0.02334701 |
| set4 | BT4073 | 0.0104245 | 0.003026431 | 0.000116769 | 0.000131072 | 0.0123059 | 0.01472884 |
| set4 | BT4110 | 0.000400016 | 0.000176844 | 0.000670227 | 0.00056894 | 0.0214578 | 0.02118246 |
| set4 | BT4121 | 0.000410355 | 0.000180361 | 0.000209543 | 0.000213601 | 0.0664098 | 0.04646727 |
| set4 | BT4131 | 0.000223261 | 0.000110299 | 0.000000202 | 0.000000639 | 0.00995294 | 0.01281589 |
| set4 | BT4132 | 4.23285E-05 | 0.0000301 | 0.00631127 | 0.004023061 | 0.164614 | 0.08949631 |
| set4 | BT4136 | 0.00184825 | 0.00065185 | 0.00000465 | 0.00000887 | 0.104209 | 0.06400434 |
| set4 | BT4140 | 0.000000425 | 0.00000154 | 0.00000954 | 0.0000159 | 0.737767 | 0.2677998 |
| set4 | BT4141 | 0.000000427 | 0.00000155 | 8.46E-10 | 7.9E-09 | 0.0101186 | 0.01296098 |
| set4 | BT4145 | 0.000243716 | 0.000118623 | 0.00046267 | 0.000414294 | 0.176433 | 0.09417795 |
| set4 | BT4146 | 0.00355236 | 0.001162406 | 0.000497024 | 0.000440735 | 0.586188 | 0.2272785 |
| set4 | BT4161 | 0.000000716 | 0.00000213 | 0.011329 | 0.006646367 | 0.490352 | 0.2004265 |
| set4 | BT4172 | 0.0149604 | 0.004192134 | 0.00374453 | 0.002522703 | 0.0130257 | 0.01521968 |
| set4 | BT4179 | 0.0100299 | 0.002924209 | 0.00000148 | 0.00000333 | 0.00835653 | 0.01132152 |
| set4 | BT4180 | 0.0184968 | 0.00507048 | 0.00433503 | 0.002870889 | 0.940032 | 0.3161313 |
| set4 | BT4193 | 1.86874E-05 | 0.0000165 | 0.00000052 | 0.00000138 | 0.267102 | 0.1292301 |
| set4 | BT4195 | 0.00021618 | 0.000107291 | 0.000269046 | 0.000263348 | 0.401951 | 0.1735616 |
| set4 | BT4198 | 0.000558222 | 0.00023407 | 0.00858878 | 0.005237163 | 0.0336736 | 0.02857232 |
| set4 | BT4212 | 0.0180959 | 0.004974971 | 2.32039E-05 | 0.0000338 | 0.361968 | 0.1608658 |
| set4 | BT4214 | 1.44876E-05 | 0.0000138 | 0.000755534 | 0.000629826 | 0.222338 | 0.1129089 |
| set4 | BT4222 | 0.0000026 | 0.00000431 | 0.00523874 | 0.003404055 | 0.45598 | 0.1906413 |
| set4 | BT4223 | 0.00053512 | 0.000225759 | 0.000243939 | 0.000242524 | 0.403734 | 0.1741012 |
| set4 | BT4231 | 0.0000057 | 0.0000072 | 0.000000301 | 0.000000875 | 0.277681 | 0.133033 |
| set4 | BT4232 | 0.000130841 | 0.0000713 | 0.00159627 | 0.001206643 | 0.0110909 | 0.01378209 |
| set4 | BT4237 | 0.0000016 | 0.00000337 | 0.000145658 | 0.000158084 | 0.0435507 | 0.03417992 |
| set4 | BT4239 | 0.00336779 | 0.001106796 | 0.0023332 | 0.001682621 | 0.0828274 | 0.05413654 |
| set4 | BT4248 | 0.000000799 | 0.00000226 | 0.00883164 | 0.005363591 | 0.132181 | 0.0757342 |
| set4 | BT4249 | 0.00000112 | 0.00000273 | 0.000951525 | 0.000768312 | 0.366114 | 0.1623738 |
| set4 | BT4250 | 0.00000318 | 0.00000503 | 0.00071716 | 0.00060243 | 0.623399 | 0.2374414 |
| set4 | BT4258 | 1.23945E-05 | 0.0000124 | 0.0022679 | 0.001641408 | 0.126737 | 0.07350441 |
| set4 | BT4261 | 0.000703856 | 0.000284574 | 0.00179053 | 0.001339598 | 0.108331 | 0.06592916 |
| set4 | BT4263 | 6.75692E-05 | 0.000043 | 0.0104823 | 0.006197796 | 0.388609 | 0.169272 |
| set4 | BT4268 | 0.00733288 | 0.002216444 | 0.0000011 | 0.00000258 | 0.269254 | 0.130057 |
| set4 | BT4270 | 0.00194082 | 0.000681956 | 0.00000394 | 0.00000776 | 0.0218122 | 0.02131325 |
| set4 | BT4273 | 0.00490078 | 0.001551184 | 0.00000621 | 0.0000112 | 0.0226972 | 0.02180475 |
| set4 | BT4280 | 0.00000316 | 0.00000501 | 0.00846269 | 0.005175954 | 0.153938 | 0.08491865 |
| set4 | BT4281 | 0.00255647 | 0.000872753 | 0.016363 | 0.009062673 | 0.0719184 | 0.04903409 |
| set4 | BT4288 | 0.000417699 | 0.000183376 | 0.00000123 | 0.00000282 | 0.00832667 | 0.01130701 |
| set4 | BT4292 | 0.000000368 | 0.00000142 | 2.94E-09 | 2.04E-08 | 0.0166836 | 0.0178404 |
| set4 | BT4302 | 1.36152E-05 | 0.0000132 | 0.00237399 | 0.001704912 | 0.0143304 | 0.01623138 |
| set4 | BT4311 | 2.34864E-05 | 0.0000194 | 0.00246955 | 0.0017641 | 0.542997 | 0.2148452 |
| set4 | BT4318 | 6.15608E-05 | 0.0000399 | 0.00234364 | 0.001689141 | 0.130581 | 0.07508122 |
| set4 | BT4319 | 0.00807903 | 0.002414972 | 0.00098936 | 0.000796732 | 0.843454 | 0.2937419 |
| set4 | BT4321 | 0.000533856 | 0.000225431 | 0.00409307 | 0.002727096 | 0.193364 | 0.1010476 |
| set4 | BT4322 | 8.48776E-05 | 0.0000513 | 0.0100208 | 0.005974644 | 0.00845808 | 0.01139062 |
| set4 | BT4325 | 0.00738047 | 0.002226383 | 0.000160049 | 0.00017075 | 0.439377 | 0.1853077 |
| set4 | BT4326 | 1.17592E-05 | 0.0000119 | 0.00413383 | 0.002750774 | 0.26621 | 0.1289686 |
| set4 | BT4337 | 0.0318934 | 0.008244158 | 0.00130508 | 0.00100995 | 0.140169 | 0.07922863 |
| set4 | BT4354 | 0.0296782 | 0.00773495 | 0.00208853 | 0.001531779 | 0.816855 | 0.2873699 |
| set4 | BT4356 | 0.0000022 | 0.00000398 | 0.016113 | 0.008952945 | 0.171542 | 0.09223801 |
| set4 | BT4360 | 0.000000195 | 0.00000105 | 0.00353981 | 0.002402199 | 0.307742 | 0.1427109 |
| set4 | BT4361 | 0.000353442 | 0.000160072 | 0.000682074 | 0.000577373 | 0.0813274 | 0.05346263 |
| set4 | BT4370 | 0.0258363 | 0.006846816 | 0.0125436 | 0.007256727 | 0.63537 | 0.2404274 |
| set4 | BT4374 | 1.60565E-05 | 0.0000147 | 0.000391722 | 0.000361759 | 0.0529545 | 0.03933756 |
| set4 | BT4379 | 0.0266328 | 0.00703818 | 7.87E-08 | 0.000000303 | 0.0656664 | 0.04619024 |
| set4 | BT4384 | 2.96E-08 | 0.000000423 | 0.000364714 | 0.000342111 | 0.0105781 | 0.01328297 |
| set4 | BT4386 | 0.0332897 | 0.00856123 | 1.29055E-05 | 0.0000208 | 0.269467 | 0.130117 |
| set4 | BT4403 | 2.51408E-05 | 0.0000204 | 2.45E-08 | 0.000000115 | 0.350231 | 0.1571724 |
| set4 | BT4406 | 0.00374326 | 0.001217503 | 2.07669E-05 | 0.0000307 | 0.111159 | 0.0669288 |
| set4 | BT4416 | 0.0014051 | 0.00051725 | 0.00315662 | 0.002177636 | 0.237717 | 0.1182879 |
| set4 | BT4418 | 0.00000105 | 0.00000263 | 0.000321852 | 0.000308312 | 0.00773716 | 0.01084768 |
| set4 | BT4437 | 1.87147E-05 | 0.0000165 | 0.00395761 | 0.002646334 | 0.0329659 | 0.02815223 |
| set4 | BT4440 | 0.00526236 | 0.001651276 | 0.000364782 | 0.000342111 | 0.082832 | 0.05413654 |
| set4 | BT4441 | 0.0014596 | 0.000534712 | 1.70419E-05 | 0.0000261 | 0.62658 | 0.2383438 |
| set4 | BT4443 | 1.94625E-05 | 0.0000169 | 0.000390624 | 0.000361021 | 0.0126047 | 0.01490328 |
| set4 | BT4450 | 4.44103E-05 | 0.0000313 | 0.0155792 | 0.008708422 | 0.0111198 | 0.01378209 |
| set4 | BT4453 | 0.000421794 | 0.000184852 | 0.000671938 | 0.000569991 | 0.426531 | 0.1813482 |
| set4 | BT4459 | 0.012453 | 0.00356071 | 0.00405918 | 0.002707503 | 0.00800556 | 0.01105505 |
| set4 | BT4465 | 0.0145091 | 0.004085336 | 4.55E-09 | 2.88E-08 | 0.805344 | 0.2844634 |
| set4 | BT4466 | 5.91818E-05 | 0.0000386 | 0.000000341 | 0.000000973 | 0.82762 | 0.2897399 |
| set4 | BT4467 | 0.00000227 | 0.000004 | 0.000000686 | 0.00000173 | 0.628066 | 0.2385999 |
| set4 | BT4485 | 0.0158123 | 0.004411251 | 0.00256285 | 0.001819986 | 0.0140094 | 0.01597513 |
| set4 | BT4490 | 0.00110817 | 0.000423816 | 0.00369541 | 0.002496573 | 0.00761404 | 0.01077692 |
| set4 | BT4496 | 0.000000444 | 0.00000158 | 5.73682E-05 | 0.0000723 | 0.391665 | 0.1704009 |
| set4 | BT4501 | 0.0049953 | 0.001576627 | 0.00037967 | 0.000352787 | 0.0140076 | 0.01597513 |
| set4 | BT4502 | 0.00068265 | 0.000277209 | 0.000145031 | 0.000157687 | 0.948668 | 0.3178469 |
| set4 | BT4508 | 0.000203886 | 0.000102466 | 0.000047586 | 0.0000615 | 0.0750276 | 0.05063464 |
| set4 | BT4512 | 8.85632E-05 | 0.0000532 | 0.000274692 | 0.000267632 | 0.654171 | 0.2456562 |
| set4 | BT4516 | 0.000278481 | 0.000132394 | 0.000000364 | 0.00000103 | 0.165616 | 0.08987464 |
| set4 | BT4525 | 8.21576E-05 | 0.0000502 | 0.00781186 | 0.004844116 | 0.020558 | 0.0205289 |
| set4 | BT4526 | 6.72E-08 | 0.000000654 | 0.000000349 | 0.000000993 | 0.0409104 | 0.03265549 |
| set4 | BT4532 | 0.000243399 | 0.000118545 | 0.0151424 | 0.008495711 | 0.0136666 | 0.01569755 |
| set4 | BT4537 | 0.00430919 | 0.001383147 | 0.00175236 | 0.00131476 | 0.248665 | 0.1223668 |
| set4 | BT4539 | 0.000706453 | 0.000285471 | 0.00161988 | 0.001223724 | 0.0597805 | 0.04301615 |
| set4 | BT4546 | 0.00511999 | 0.001611949 | 0.00000861 | 0.0000146 | 0.0406654 | 0.03255339 |
| set4 | BT4547 | 0.00301876 | 0.001006513 | 0.00249661 | 0.001780272 | 0.0866989 | 0.05581885 |
| set4 | BT4557 | 0.000320502 | 0.000147642 | 0.00905472 | 0.005477041 | 0.541174 | 0.214355 |
| set4 | BT4567 | 0.000000991 | 0.00000256 | 0.0179873 | 0.009853861 | 0.0104211 | 0.01321013 |
| set4 | BT4568 | 0.000000011 | 0.000000264 | 0.000044228 | 0.0000579 | 0.981743 | 0.3247227 |
| set4 | BT4569 | 0.00195743 | 0.000687154 | 9.12E-12 | 2.45E-10 | 0.698376 | 0.2574387 |
| set4 | BT4570 | 0.00000395 | 0.00000567 | 4.98E-09 | 3.09E-08 | 0.756413 | 0.27268 |
| set4 | BT4571 | 0.000000321 | 0.0000013 | 0.000000115 | 0.000000404 | 0.08658 | 0.05579124 |
| set4 | BT4575 | 1.01772E-05 | 0.0000107 | 0.00838182 | 0.005131689 | 0.0228576 | 0.02190151 |
| set4 | BT4599 | 2.97571E-05 | 0.0000233 | 0.0073468 | 0.004610166 | 0.0203444 | 0.02038494 |
| set4 | BT4600 | 0.00000989 | 0.0000105 | 0.0137139 | 0.007848301 | 0.0289147 | 0.02568965 |
| set4 | BT4603 | 9.77956E-05 | 0.0000572 | 0.0100268 | 0.005975272 | 0.0959186 | 0.0603518 |
| set4 | BT4604 | 0.000504825 | 0.000214882 | 5.18866E-05 | 0.0000664 | 0.0501082 | 0.03761963 |
| set4 | BT4612 | 0.00000868 | 0.00000957 | 0.0148841 | 0.008394634 | 0.0222014 | 0.02152745 |
| set4 | BT4616 | 0.0187471 | 0.005129777 | 0.00218988 | 0.001594051 | 0.499717 | 0.2027485 |
| set4 | BT4619 | 0.00694071 | 0.002114798 | 0.000000282 | 0.000000826 | 0.112561 | 0.0674961 |
| set4 | BT4622 | 0.00064804 | 0.000265697 | 0.0147899 | 0.00835225 | 0.0368011 | 0.03033186 |
| set4 | BT4625 | 2.51081E-05 | 0.0000204 | 3.24932E-05 | 0.0000447 | 0.0452943 | 0.03503075 |
| set4 | BT4632 | 0.00164989 | 0.000593507 | 0.000100435 | 0.000115654 | 0.0123476 | 0.01475372 |
| set4 | BT4650 | 0.00179752 | 0.00063872 | 0.000000821 | 0.00000202 | 0.0113672 | 0.01393996 |
| set4 | BT4651 | 0.0105284 | 0.003054252 | 0.00353651 | 0.002401309 | 0.702083 | 0.2583942 |
| set4 | BT4659 | 0.000338503 | 0.000154524 | 0.000000052 | 0.000000215 | 0.0116601 | 0.01415722 |
| set4 | BT4663 | 0.00318321 | 0.001054625 | 0.00118051 | 0.000927177 | 0.0105618 | 0.01327386 |
| set4 | BT4667 | 0.030924 | 0.00802372 | 8.07987E-05 | 0.0000957 | 0.140883 | 0.07954033 |
| set4 | BT4668 | 0.00358029 | 0.001170028 | 0.00112411 | 0.000888658 | 0.316705 | 0.145873 |
| set4 | BT4671 | 0.00398841 | 0.001289481 | 0.0137841 | 0.007876305 | 0.354858 | 0.1584736 |
| set4 | BT4674 | 0.0235077 | 0.006282514 | 0.000366475 | 0.000343432 | 0.536735 | 0.213172 |
| set4 | BT4678 | 0.000458021 | 0.000197639 | 0.0055642 | 0.003590426 | 0.292744 | 0.137644 |
| set4 | BT4693 | 0.00647743 | 0.001986434 | 5.60726E-05 | 0.000071 | 0.109083 | 0.06626398 |
| set4 | BT4696 | 0.000717686 | 0.000289392 | 0.00000171 | 0.00000376 | 0.0344217 | 0.02905593 |
| set4 | BT4697 | 0.00361995 | 0.001182478 | 5.43E-14 | 5.46E-12 | 0.0254604 | 0.02344655 |
| set4 | BT4720 | 6.82396E-05 | 0.0000433 | 1.31778E-05 | 0.0000211 | 0.0165261 | 0.01775913 |
| set4 | BT4721 | 0.000000951 | 0.00000254 | 4.83363E-05 | 0.0000624 | 0.0318998 | 0.02760986 |
| set4 | BT4729 | 0.00000516 | 0.00000669 | 0.0113787 | 0.006669046 | 0.128002 | 0.07391683 |
| set4 | BT4732 | 5.07E-08 | 0.000000614 | 0.000138554 | 0.000151985 | 0.297207 | 0.1392073 |
| set4 | BT4733 | 3.53E-08 | 0.000000476 | 0.000424405 | 0.000385459 | 0.0219547 | 0.02137117 |
| set4 | BT4742 | 0.00676303 | 0.002063151 | 0.00000218 | 0.00000461 | 0.0447204 | 0.03478852 |
| set4 | BT4746 | 0.000167589 | 0.0000867 | 0.000178265 | 0.000186761 | 0.0718724 | 0.04903409 |
| set4 | BT4749 | 0.0194231 | 0.005293699 | 6.04E-11 | 1.03E-09 | 0.583423 | 0.2265642 |
| set4 | BT4751 | 0.000262278 | 0.000126117 | 3.07561E-05 | 0.0000426 | 0.00770927 | 0.01083964 |
| set4 | BT4760 | 0.000111936 | 0.0000633 | 0.00042786 | 0.000387738 | 0.0535156 | 0.0395751 |
| set4 | BT4761 | 0.000129021 | 0.0000706 | 7.63E-10 | 7.38E-09 | 0.0424131 | 0.03345609 |
| set4 | BT4766 | 0.00081067 | 0.000322929 | 0.0119532 | 0.006965206 | 0.704607 | 0.258965 |
| set4 | BT4768 | 0.00498153 | 0.001573593 | 9.31E-08 | 0.000000342 | 0.265916 | 0.1289686 |
| set4 | BT4769 | 0.00000323 | 0.00000506 | 0.00033752 | 0.000321282 | 0.0809684 | 0.05331232 |
| set4 | BT4771 | 0.0159149 | 0.004433338 | 2.22E-10 | 2.88E-09 | 0.00929407 | 0.01221391 |
| set4 | BT4772 | 0.000000175 | 0.000001 | 0.00000123 | 0.00000282 | 0.355509 | 0.1587161 |
| set4 | BT4773 | 7.90063E-05 | 0.0000489 | 1.92423E-05 | 0.0000287 | 0.125956 | 0.07310922 |
| set4 | BT4776 | 1.45934E-05 | 0.0000138 | 0.00000754 | 0.0000131 | 0.0137355 | 0.01575203 |
| set4 | BT4781 | 6.22762E-05 | 0.0000402 | 0.00000151 | 0.00000336 | 0.00713359 | 0.01034742 |
| set4 | SusC | 4.47248E-05 | 0.0000314 | 0.00243723 | 0.001744107 | 0.00739829 | 0.01057458 |
| set4 | SusF | 0.00000154 | 0.00000331 | 0.00163196 | 0.00123131 | 0.0094306 | 0.01234911 |
| set4 | tRNA_02 | 0.000000298 | 0.00000127 | 1.70344E-05 | 0.0000261 | 0.591702 | 0.2288121 |
| set4 | tRNA_09 | 2.62077E-05 | 0.000021 | 0.000067214 | 0.0000826 | 0.11605 | 0.06888476 |
| set4 | tRNA_22 | 0.00000517 | 0.00000669 | 1.79841E-05 | 0.0000274 | 0.383372 | 0.1677874 |
| set4 | tRNA_28 | 0.000557988 | 0.00023407 | 2.10965E-05 | 0.0000311 | 0.0140564 | 0.01598112 |
| set4 | tRNA_30 | 0.000000383 | 0.00000146 | 0.000000128 | 0.000000443 | 0.438543 | 0.1850908 |
| set4 | tRNA_35 | 0.000000281 | 0.00000127 | 0.00221644 | 0.001608018 | 0.147604 | 0.08213447 |
| set4 | tRNA_37 | 1.44436E-05 | 0.0000138 | 0.000000132 | 0.000000457 | 0.859524 | 0.2974332 |
| set4 | tRNA_56 | 0.000000138 | 0.000000919 | 0.000133452 | 0.000147083 | 0.0468338 | 0.03584371 |
| set4 | tRNA_65 | 0.000114029 | 0.0000641 | 0.000000798 | 0.00000198 | 0.68675 | 0.2545691 |
| set5 | BT0002 | 0.000000136 | 0.000000919 | 0.028044 | 0.01438404 | 0.000117665 | 0.000966006 |
| set5 | BT0019 | 0.00000279 | 0.00000453 | 0.0711253 | 0.031471 | 0.00332787 | 0.006262877 |
| set5 | BT0072 | 0.00000268 | 0.0000044 | 0.00053547 | 0.000469732 | 0.00137728 | 0.003534503 |
| set5 | BT0155 | 0.00000111 | 0.00000273 | 0.000658979 | 0.000560968 | 0.00145761 | 0.003676381 |
| set5 | BT0234 | 2.89E-08 | 0.000000421 | 0.0141082 | 0.008031105 | 0.000518595 | 0.001981676 |
| set5 | BT0328 | 0.00000184 | 0.00000357 | 0.413886 | 0.1357832 | 0.00556862 | 0.008800664 |
| set5 | BT0338 | 0.00000155 | 0.00000331 | 0.0144719 | 0.008195657 | 0.00468534 | 0.007842303 |
| set5 | BT0339 | 0.00000182 | 0.00000357 | 0.0627328 | 0.0282438 | 0.00101586 | 0.003032208 |
| set5 | BT0342 | 0.00000114 | 0.00000275 | 0.323526 | 0.1108806 | 0.00327926 | 0.006195223 |
| set5 | BT0343 | 0.00000531 | 0.0000068 | 0.00236593 | 0.001703174 | 0.00218301 | 0.004657677 |
| set5 | BT0398 | 0.00000105 | 0.00000263 | 4.45395E-05 | 0.000058 | 0.00136822 | 0.003523572 |
| set5 | BT0402 | 0.00000808 | 0.00000915 | 0.00451046 | 0.002977274 | 0.00493883 | 0.00811848 |
| set5 | BT0405 | 0.00000153 | 0.00000328 | 0.913168 | 0.2542202 | 0.00158373 | 0.003874646 |
| set5 | BT0410 | 0.000000419 | 0.00000154 | 0.374035 | 0.1249145 | 0.00658338 | 0.009761484 |
| set5 | BT0465 | 0.00000184 | 0.00000357 | 0.00151186 | 0.001150033 | 0.0010163 | 0.003032208 |
| set5 | BT0497 | 0.00000013 | 0.000000919 | 0.197826 | 0.07386831 | 0.00034453 | 0.001626083 |
| set5 | BT0525 | 5.48E-10 | 0.000000111 | 0.00000545 | 0.0000101 | 0.0020518 | 0.004495338 |
| set5 | BT0534 | 0.000000991 | 0.00000256 | 0.122272 | 0.0494471 | 0.000530282 | 0.002011398 |
| set5 | BT0539 | 0.00000128 | 0.00000297 | 0.00144097 | 0.001101658 | 0.00235423 | 0.004907002 |
| set5 | BT0566 | 0.00000735 | 0.00000852 | 0.143173 | 0.056547 | 0.00670749 | 0.00991546 |
| set5 | BT0580 | 0.00000243 | 0.00000414 | 0.248627 | 0.08943689 | 0.00138997 | 0.003550322 |
| set5 | BT0681 | 0.00000184 | 0.00000357 | 0.0234546 | 0.01232362 | 0.000797106 | 0.002644624 |
| set5 | BT0793 | 7.67E-08 | 0.000000708 | 0.137476 | 0.05459046 | 0.000633279 | 0.002256637 |
| set5 | BT0805 | 0.00000371 | 0.00000551 | 0.000812987 | 0.000671256 | 0.0067873 | 0.00997318 |
| set5 | BT0821 | 0.00000125 | 0.00000291 | 0.000986051 | 0.000794597 | 0.00653919 | 0.009735296 |
| set5 | BT0905 | 0.000000826 | 0.00000231 | 0.0381971 | 0.01865758 | 0.00378353 | 0.006800131 |
| set5 | BT0907 | 0.00000426 | 0.00000592 | 0.0432019 | 0.02079113 | 0.00507171 | 0.008272768 |
| set5 | BT0920 | 2.45E-08 | 0.000000386 | 0.00534038 | 0.003464508 | 0.00115038 | 0.003247432 |
| set5 | BT0925 | 0.000000411 | 0.00000152 | 0.69057 | 0.2040544 | 0.00529941 | 0.008501754 |
| set5 | BT0960 | 0.00000281 | 0.00000455 | 3.13448E-05 | 0.0000433 | 0.00629603 | 0.009518087 |
| set5 | BT0963 | 7.23E-08 | 0.000000684 | 0.0181488 | 0.009917321 | 0.00017704 | 0.001149913 |
| set5 | BT0985 | 0.00000222 | 0.00000399 | 0.00205676 | 0.001512151 | 0.00129358 | 0.003433763 |
| set5 | BT1087 | 0.000000311 | 0.00000128 | 0.0320862 | 0.01610912 | 0.00105733 | 0.00310415 |
| set5 | BT1092 | 5.19E-08 | 0.000000614 | 0.835963 | 0.2372655 | 0.000162702 | 0.001124178 |
| set5 | BT1096 | 2.39E-08 | 0.000000385 | 0.00738114 | 0.004626907 | 0.00369876 | 0.006727981 |
| set5 | BT1121 | 0.00000226 | 0.000004 | 0.00934762 | 0.005631651 | 0.00214429 | 0.004624398 |
| set5 | BT1145 | 0.000000239 | 0.00000115 | 0.570166 | 0.1756522 | 0.000563805 | 0.002084685 |
| set5 | BT1179 | 0.00000001 | 0.000000253 | 0.000950597 | 0.000768076 | 0.0000086 | 0.00028706 |
| set5 | BT1181 | 0.000000131 | 0.000000919 | 0.0866701 | 0.03708579 | 0.000152646 | 0.001093034 |
| set5 | BT1228 | 5.87E-10 | 0.000000111 | 0.0249198 | 0.012961 | 9.57462E-05 | 0.00091342 |
| set5 | BT1229 | 6.47E-08 | 0.000000654 | 0.584692 | 0.1789425 | 0.00185601 | 0.004223988 |
| set5 | BT1239 | 0.000000905 | 0.00000245 | 0.0781804 | 0.03404399 | 0.00674119 | 0.009945248 |
| set5 | BT1263 | 0.000000292 | 0.00000127 | 0.906959 | 0.2531334 | 0.000103891 | 0.0009299 |
| set5 | BT1274 | 0.00000188 | 0.00000363 | 0.0285366 | 0.0145939 | 0.00304328 | 0.005862584 |
| set5 | BT1301 | 0.000000586 | 0.00000189 | 0.890288 | 0.2492271 | 0.00227091 | 0.004778612 |
| set5 | BT1341 | 0.00000516 | 0.00000669 | 0.019714 | 0.01061209 | 0.00395957 | 0.007028794 |
| set5 | BT1363 | 0.000000597 | 0.0000019 | 0.345301 | 0.1173295 | 0.00254829 | 0.005195394 |
| set5 | BT1419 | 0.00000177 | 0.00000355 | 0.087035 | 0.03720239 | 0.00156977 | 0.003853337 |
| set5 | BT1477 | 0.0000032 | 0.00000505 | 0.000472604 | 0.000421315 | 0.0011421 | 0.003244808 |
| set5 | BT1483 | 0.00000165 | 0.00000342 | 0.000449123 | 0.00040396 | 0.0067794 | 0.009971554 |
| set5 | BT1498 | 0.000000135 | 0.000000919 | 0.708785 | 0.20781 | 0.00013765 | 0.001063469 |
| set5 | BT1597 | 0.00000385 | 0.00000558 | 0.331977 | 0.1134083 | 0.00194556 | 0.004356953 |
| set5 | BT1612 | 2.16E-08 | 0.000000379 | 0.000120451 | 0.000134348 | 0.00010504 | 0.000934401 |
| set5 | BT1622 | 0.00000243 | 0.00000414 | 0.498831 | 0.1575369 | 0.0043505 | 0.007477965 |
| set5 | BT1626 | 0.00000259 | 0.00000431 | 0.00392592 | 0.002628778 | 0.0060627 | 0.009298403 |
| set5 | BT1634 | 0.000000122 | 0.000000919 | 0.000649927 | 0.000554043 | 0.00239703 | 0.004964051 |
| set5 | BT1696 | 1.44706E-05 | 0.0000138 | 0.215721 | 0.0795422 | 0.00657355 | 0.009761484 |
| set5 | BT1712 | 8.63E-09 | 0.000000225 | 0.000129491 | 0.000143372 | 0.00459693 | 0.007738442 |
| set5 | BT1731 | 3.94E-09 | 0.000000208 | 0.035811 | 0.01767071 | 0.000110244 | 0.000948054 |
| set5 | BT1751 | 0.000264186 | 0.000126826 | 0.000000278 | 0.000000818 | 0.0000001 | 0.0000245 |
| set5 | BT1786 | 0.0000003 | 0.00000127 | 0.0113631 | 0.006663136 | 0.00438868 | 0.007534769 |
| set5 | BT1787 | 0.000000192 | 0.00000105 | 0.907516 | 0.253141 | 0.000896349 | 0.002829608 |
| set5 | BT1896 | 6.36E-08 | 0.000000654 | 0.00020506 | 0.000209562 | 0.00005507 | 0.00068507 |
| set5 | BT1912 | 3.52E-08 | 0.000000476 | 0.0408675 | 0.01980181 | 0.000376803 | 0.001695937 |
| set5 | BT1953 | 0.00000197 | 0.00000371 | 0.000417721 | 0.000381684 | 0.00266643 | 0.005339841 |
| set5 | BT2003 | 5.14E-09 | 0.000000216 | 0.119463 | 0.04856391 | 0.000124633 | 0.000999733 |
| set5 | BT2048 | 0.00000441 | 0.00000604 | 0.67673 | 0.2005731 | 0.00215182 | 0.00462474 |
| set5 | BT2065 | 0.00000289 | 0.00000465 | 0.0175609 | 0.009646519 | 0.00166956 | 0.003995083 |
| set5 | BT2075 | 0.000000324 | 0.00000131 | 0.941928 | 0.2596677 | 0.00622602 | 0.009450806 |
| set5 | BT2122 | 0.00000196 | 0.00000371 | 0.609896 | 0.1848691 | 0.00135887 | 0.003515318 |
| set5 | BT2178 | 1.53E-08 | 0.000000297 | 9.86385E-05 | 0.00011402 | 0.000835418 | 0.002725169 |
| set5 | BT2185 | 0.000000202 | 0.00000108 | 0.00134424 | 0.001034252 | 0.00118667 | 0.003292884 |
| set5 | BT2189 | 1.14695E-05 | 0.0000117 | 0.151437 | 0.05921928 | 0.0062697 | 0.009492427 |
| set5 | BT2208 | 0.000000173 | 0.000001 | 0.0169299 | 0.009342384 | 6.11012E-05 | 0.000722189 |
| set5 | BT2215 | 1.42E-08 | 0.000000297 | 0.293131 | 0.1023219 | 0.00257129 | 0.005220574 |
| set5 | BT2232 | 0.000000136 | 0.000000919 | 0.707321 | 0.2075902 | 0.000297803 | 0.001476861 |
| set5 | BT2242 | 0.0000043 | 0.00000593 | 0.00264963 | 0.001874998 | 0.00199466 | 0.004431522 |
| set5 | BT2280 | 0.000000207 | 0.00000108 | 0.741537 | 0.2158409 | 0.00610327 | 0.009302496 |
| set5 | BT2289 | 0.00000223 | 0.000004 | 0.748688 | 0.217221 | 0.000828806 | 0.002709622 |
| set5 | BT2292 | 0.00000205 | 0.0000038 | 0.612608 | 0.1853707 | 0.000814529 | 0.002677616 |
| set5 | BT2300 | 0.00000323 | 0.00000506 | 0.588152 | 0.1796355 | 0.00474939 | 0.00787765 |
| set5 | BT2301 | 0.000000131 | 0.000000919 | 0.100649 | 0.0419817 | 0.00296427 | 0.005763323 |
| set5 | BT2315 | 0.000000735 | 0.00000215 | 0.00241095 | 0.001727347 | 0.00404384 | 0.007138374 |
| set5 | BT2406 | 2.56E-08 | 0.000000387 | 0.00000493 | 0.00000932 | 0.000918187 | 0.002861623 |
| set5 | BT2408 | 2.54E-08 | 0.000000387 | 0.677464 | 0.2007218 | 0.00278379 | 0.005537098 |
| set5 | BT2448 | 0.000000199 | 0.00000107 | 0.666754 | 0.1984736 | 0.000151017 | 0.00108667 |
| set5 | BT2468 | 0.00000014 | 0.000000919 | 0.000274733 | 0.000267632 | 0.000270516 | 0.001379763 |
| set5 | BT2474 | 0.000000332 | 0.00000133 | 0.595498 | 0.1813747 | 0.000627303 | 0.002255036 |
| set5 | BT2488 | 0.00000378 | 0.00000552 | 0.0585873 | 0.02662549 | 0.00669745 | 0.0099106 |
| set5 | BT2533 | 0.00000428 | 0.00000593 | 0.00775513 | 0.004821313 | 0.00159803 | 0.003894256 |
| set5 | BT2548 | 0.000000086 | 0.000000757 | 0.00498916 | 0.003261178 | 0.00578612 | 0.00904532 |
| set5 | BT2559 | 6.04E-09 | 0.000000222 | 0.00482283 | 0.003169608 | 0.00269335 | 0.005386403 |
| set5 | BT2587 | 0.000000691 | 0.00000208 | 0.0931525 | 0.03920683 | 0.00100069 | 0.003010106 |
| set5 | BT2613 | 0.00000485 | 0.00000646 | 0.201305 | 0.07498194 | 0.0018557 | 0.004223988 |
| set5 | BT2644 | 0.0000046 | 0.00000623 | 0.000313704 | 0.000301463 | 0.00341335 | 0.006361529 |
| set5 | BT2662 | 0.00000489 | 0.0000065 | 0.115216 | 0.04719403 | 0.00422685 | 0.007316832 |
| set5 | BT2744 | 0.0346619 | 0.00887492 | 0.000000191 | 0.000000612 | 4.53E-08 | 0.0000245 |
| set5 | BT2780 | 4.98E-08 | 0.000000614 | 0.88732 | 0.2487441 | 0.000105667 | 0.000934401 |
| set5 | BT2801 | 0.000000166 | 0.000001 | 0.000766447 | 0.000636309 | 0.000477891 | 0.001906264 |
| set5 | BT2816 | 0.000000302 | 0.00000127 | 0.000208494 | 0.000212712 | 0.000891721 | 0.002821065 |
| set5 | BT2821 | 0.000000756 | 0.0000022 | 0.00250084 | 0.001781185 | 0.00296184 | 0.005763323 |
| set5 | BT2824 | 1.38E-08 | 0.000000297 | 0.0912391 | 0.03858974 | 0.000200912 | 0.001242379 |
| set5 | BT2902 | 0.0021765 | 0.000753917 | 0.000000203 | 0.000000641 | 7.49E-08 | 0.0000245 |
| set5 | BT2925 | 0.000000101 | 0.000000835 | 0.180756 | 0.06868446 | 0.00319454 | 0.006074257 |
| set5 | BT2933 | 0.000002 | 0.00000376 | 0.493823 | 0.1563601 | 0.0045507 | 0.00768081 |
| set5 | BT2961 | 0.000000463 | 0.00000161 | 0.143972 | 0.056778 | 0.00116179 | 0.003260829 |
| set5 | BT2972 | 0.00000139 | 0.00000312 | 1.51961E-05 | 0.0000237 | 0.00375124 | 0.006773083 |
| set5 | BT3015 | 0.00000129 | 0.00000297 | 0.000101899 | 0.000117005 | 0.000815168 | 0.002677616 |
| set5 | BT3034 | 0.00000289 | 0.00000465 | 0.0718606 | 0.03173822 | 0.00171215 | 0.004047178 |
| set5 | BT3112 | 6.11E-09 | 0.000000222 | 4.86015E-05 | 0.0000625 | 7.52668E-05 | 0.00079486 |
| set5 | BT3138 | 0.000000291 | 0.00000127 | 0.766682 | 0.2213994 | 0.000977204 | 0.002978746 |
| set5 | BT3165 | 0.000000091 | 0.000000783 | 1.38834E-05 | 0.000022 | 4.29572E-05 | 0.000625552 |
| set5 | BT3262 | 0.000715686 | 0.00028874 | 0.0438654 | 0.02102648 | 0.00000147 | 0.000119201 |
| set5 | BT3264 | 3.16E-09 | 0.000000196 | 0.000146175 | 0.000158503 | 0.000175259 | 0.001149913 |
| set5 | BT3291 | 0.000000166 | 0.000001 | 0.636054 | 0.1906505 | 0.00351871 | 0.006497084 |
| set5 | BT3296 | 6.24E-08 | 0.000000654 | 0.0117328 | 0.006853307 | 0.000168369 | 0.00113895 |
| set5 | BT3306 | 0.000000911 | 0.00000246 | 0.326652 | 0.1118422 | 0.00496095 | 0.00814572 |
| set5 | BT3419 | 1.09148E-05 | 0.0000112 | 0.605827 | 0.1840566 | 0.0063269 | 0.009545095 |
| set5 | BT3636 | 0.00000232 | 0.00000406 | 0.0368886 | 0.0181284 | 0.00164939 | 0.003969133 |
| set5 | BT3678 | 0.00000325 | 0.00000507 | 0.000154372 | 0.000165607 | 0.00300465 | 0.005811045 |
| set5 | BT3682 | 0.00000723 | 0.00000844 | 0.036946 | 0.01814292 | 0.00350773 | 0.006493145 |
| set5 | BT3732 | 0.000000643 | 0.00000201 | 0.280117 | 0.09847713 | 0.00260546 | 0.00526805 |
| set5 | BT3756 | 0.00000841 | 0.0000094 | 0.0118241 | 0.006899964 | 0.00449237 | 0.007623625 |
| set5 | BT3771 | 0.00000174 | 0.00000352 | 0.199894 | 0.07457135 | 0.0026498 | 0.005335654 |
| set5 | BT3895 | 0.00000218 | 0.00000396 | 0.612058 | 0.1852507 | 0.000867689 | 0.002790546 |
| set5 | BT3921 | 6.46E-09 | 0.000000222 | 0.000000194 | 0.000000619 | 3.41932E-05 | 0.000583638 |
| set5 | BT3930 | 0.00000257 | 0.0000043 | 0.440547 | 0.1428614 | 0.0028437 | 0.005604444 |
| set5 | BT3945 | 3.22E-09 | 0.000000196 | 0.0151168 | 0.00848658 | 0.000889954 | 0.002821065 |
| set5 | BT3986 | 2.57E-09 | 0.000000196 | 0.775548 | 0.2235062 | 0.000941428 | 0.002903236 |
| set5 | BT3998 | 0.000000173 | 0.000001 | 0.000257947 | 0.000254773 | 9.97055E-05 | 0.000920501 |
| set5 | BT4013 | 0.000000287 | 0.00000127 | 0.0124935 | 0.007231206 | 0.00328486 | 0.006197826 |
| set5 | BT4035 | 0.000000988 | 0.00000256 | 0.000478389 | 0.00042553 | 0.00106297 | 0.003114479 |
| set5 | BT4089 | 0.00000416 | 0.00000584 | 0.00731921 | 0.004596207 | 0.00276505 | 0.005515905 |
| set5 | BT4113 | 0.00000282 | 0.00000456 | 0.152135 | 0.05943451 | 0.00149191 | 0.00373084 |
| set5 | BT4114 | 0.000000129 | 0.000000919 | 0.944823 | 0.2600955 | 0.000159349 | 0.001119193 |
| set5 | BT4129 | 0.00000241 | 0.00000414 | 0.580493 | 0.1779875 | 0.00635579 | 0.009573284 |
| set5 | BT4134 | 0.00000153 | 0.00000328 | 0.610767 | 0.184999 | 0.00165281 | 0.003970853 |
| set5 | BT4149 | 0.00000145 | 0.00000319 | 0.0698557 | 0.03097732 | 0.00309994 | 0.005956053 |
| set5 | BT4201 | 4.72E-09 | 0.000000216 | 0.0245161 | 0.01280339 | 0.000492222 | 0.001942317 |
| set5 | BT4206 | 0.00000371 | 0.00000551 | 0.021235 | 0.01133989 | 0.0017993 | 0.004140705 |
| set5 | BT4259 | 1.94E-08 | 0.00000035 | 0.000150693 | 0.000162527 | 0.000410678 | 0.001773065 |
| set5 | BT4323 | 0.000000517 | 0.00000175 | 0.00641211 | 0.004083031 | 0.00312362 | 0.00597034 |
| set5 | BT4328 | 7.99E-09 | 0.000000225 | 0.000242387 | 0.000241378 | 0.000129366 | 0.00102648 |
| set5 | BT4375 | 0.000000169 | 0.000001 | 0.852279 | 0.2409339 | 0.000375595 | 0.001695937 |
| set5 | BT4433 | 0.000000461 | 0.00000161 | 0.490797 | 0.1557285 | 0.000519746 | 0.001981676 |
| set5 | BT4451 | 0.00000325 | 0.00000507 | 0.000111028 | 0.000125578 | 0.0058396 | 0.009080572 |
| set5 | BT4484 | 0.000000386 | 0.00000146 | 0.012445 | 0.007213504 | 0.00489761 | 0.008059748 |
| set5 | BT4487 | 0.000000845 | 0.00000233 | 0.321771 | 0.110547 | 0.00302565 | 0.00584396 |
| set5 | BT4556 | 1.54E-08 | 0.000000297 | 0.0211933 | 0.01132262 | 0.00549833 | 0.008716095 |
| set5 | BT4593 | 0.000000308 | 0.00000128 | 0.745833 | 0.2166737 | 0.00224068 | 0.00473939 |
| set5 | BT4608 | 0.00000205 | 0.0000038 | 0.110024 | 0.04528229 | 0.00346565 | 0.006431471 |
| set5 | BT4611 | 0.000000156 | 0.000000985 | 0.181171 | 0.06879773 | 0.00259907 | 0.005262379 |
| set5 | BT4624 | 0.000000987 | 0.00000256 | 0.00995777 | 0.005947815 | 0.00588876 | 0.009133231 |
| set5 | BT4639 | 7.98E-08 | 0.000000717 | 0.00544055 | 0.003521926 | 0.00177842 | 0.004124134 |
| set5 | BT4664 | 0.00000728 | 0.00000848 | 0.866754 | 0.2439412 | 0.00507214 | 0.008272768 |
| set5 | BT4682 | 0.00000372 | 0.00000551 | 0.119869 | 0.04867086 | 0.0039599 | 0.007028794 |
| set5 | BT4701 | 0.00000245 | 0.00000416 | 0.353145 | 0.1193569 | 0.00641418 | 0.009627298 |
| set5 | BT4713 | 3.53E-08 | 0.000000476 | 0.000187946 | 0.000194875 | 0.00124108 | 0.003386255 |
| set5 | SusE | 0.000000332 | 0.00000133 | 0.0286454 | 0.01463715 | 0.000243722 | 0.0013492 |
| set5 | SusG | 0.00000111 | 0.00000273 | 0.0015767 | 0.001194091 | 0.00164029 | 0.003955765 |
| set5 | tRNA_17 | 0.000000187 | 0.00000103 | 0.00132645 | 0.001022518 | 0.00400014 | 0.007074557 |
| set6 | BT0006 | 0.00889654 | 0.002626151 | 0.00000253 | 0.0000052 | 0.00277824 | 0.005533557 |
| set6 | BT0052 | 0.000135431 | 0.0000732 | 9.9E-09 | 5.29E-08 | 0.00389338 | 0.006952758 |
| set6 | BT0053 | 2.55027E-05 | 0.0000206 | 0.000000452 | 0.00000122 | 0.00243664 | 0.00500951 |
| set6 | BT0054 | 1.23928E-05 | 0.0000124 | 2.03E-08 | 9.87E-08 | 0.00006308 | 0.00072504 |
| set6 | BT0055 | 2.30199E-05 | 0.0000191 | 0.000000266 | 0.000000792 | 0.000986997 | 0.002986406 |
| set6 | BT0056 | 0.000408076 | 0.000179568 | 2.11E-08 | 0.000000101 | 0.00510406 | 0.008283978 |
| set6 | BT0057 | 1.40826E-05 | 0.0000136 | 9.7E-09 | 5.21E-08 | 0.000119482 | 0.000969005 |
| set6 | BT0059 | 0.0225963 | 0.006064636 | 1.84E-11 | 3.91E-10 | 0.00191334 | 0.004314329 |
| set6 | BT0068 | 0.000653871 | 0.000267605 | 2.03E-09 | 1.52E-08 | 0.00000836 | 0.000285227 |
| set6 | BT0123 | 0.000218002 | 0.000107912 | 0.000000382 | 0.00000106 | 0.00172686 | 0.004055724 |
| set6 | BT0135 | 0.00410358 | 0.001322198 | 0.00000698 | 0.0000123 | 0.00543992 | 0.00865153 |
| set6 | BT0162 | 0.00117055 | 0.000443194 | 0.000000661 | 0.00000169 | 0.00363299 | 0.006626406 |
| set6 | BT0188 | 0.000102257 | 0.0000592 | 6.2E-10 | 6.32E-09 | 0.0002248 | 0.001319953 |
| set6 | BT0240 | 0.0623546 | 0.01507789 | 0.00000416 | 0.00000806 | 0.00537638 | 0.008597049 |
| set6 | BT0272 | 0.00000811 | 0.00000917 | 1.17E-13 | 8.34E-12 | 0.00000418 | 0.000198137 |
| set6 | BT0281 | 0.000211194 | 0.000105369 | 0.000000297 | 0.000000863 | 0.0027966 | 0.005541167 |
| set6 | BT0300 | 0.0053209 | 0.001666881 | 7.17E-08 | 0.00000028 | 0.00153576 | 0.00380806 |
| set6 | BT0455 | 0.983115 | 0.1673223 | 1.05E-08 | 5.57E-08 | 0.00194848 | 0.004356953 |
| set6 | BT0462 | 1.09475E-05 | 0.0000113 | 3.34E-11 | 6.51E-10 | 0.00235696 | 0.004907002 |
| set6 | BT0514 | 0.0144641 | 0.004074181 | 2.08E-09 | 1.54E-08 | 9.55607E-05 | 0.00091342 |
| set6 | BT0531 | 6.49378E-05 | 0.0000416 | 1.65E-08 | 8.21E-08 | 0.000133629 | 0.001048966 |
| set6 | BT0537 | 0.000317408 | 0.000146484 | 0.000000433 | 0.00000119 | 0.00243219 | 0.005007375 |
| set6 | BT0543 | 0.000232805 | 0.000114046 | 0.000000681 | 0.00000172 | 0.00413624 | 0.007236789 |
| set6 | BT0544 | 4.18354E-05 | 0.0000299 | 3.73E-17 | 1.5E-14 | 0.000569181 | 0.002099276 |
| set6 | BT0579 | 0.00103398 | 0.000399072 | 2.81E-14 | 3.77E-12 | 0.00000829 | 0.000285227 |
| set6 | BT0581 | 0.54259 | 0.1013425 | 0.0000007 | 0.00000176 | 0.00111956 | 0.003203555 |
| set6 | BT0591 | 2.78806E-05 | 0.0000221 | 0.00000139 | 0.00000315 | 0.00156228 | 0.003841375 |
| set6 | BT0597 | 0.000142839 | 0.000076 | 3.63E-08 | 0.000000159 | 0.0015218 | 0.003786236 |
| set6 | BT0640 | 0.00051035 | 0.000216759 | 4.82E-08 | 0.000000201 | 0.000256371 | 0.001364989 |
| set6 | BT0649 | 3.96888E-05 | 0.0000289 | 1.99E-09 | 1.52E-08 | 0.00521362 | 0.008428604 |
| set6 | BT0698 | 0.00000822 | 0.00000925 | 7.67E-11 | 1.24E-09 | 0.00133051 | 0.003475991 |
| set6 | BT0756 | 0.00156525 | 0.000567918 | 5.1E-14 | 5.46E-12 | 2.71474E-05 | 0.000524345 |
| set6 | BT0778 | 0.059221 | 0.0144191 | 7.43E-08 | 0.000000289 | 0.000536645 | 0.002019876 |
| set6 | BT0826 | 3.54787E-05 | 0.0000265 | 0.00219726 | 0.001596412 | 0.00000518 | 0.000223749 |
| set6 | BT0832 | 3.64691E-05 | 0.000027 | 7.23E-12 | 2.08E-10 | 4.48635E-05 | 0.000639379 |
| set6 | BT0865 | 0.000442443 | 0.000191902 | 8.25E-10 | 7.79E-09 | 8.76066E-05 | 0.000863083 |
| set6 | BT0867 | 0.00000755 | 0.00000869 | 1.38E-09 | 1.16E-08 | 0.00236254 | 0.004907002 |
| set6 | BT0913 | 0.000238458 | 0.000116589 | 0.000000271 | 0.000000801 | 0.000330566 | 0.001584817 |
| set6 | BT0922 | 0.718599 | 0.12879 | 2.07E-08 | 0.0000001 | 0.00227432 | 0.004778612 |
| set6 | BT0923 | 0.744997 | 0.1328291 | 0.000000094 | 0.000000343 | 0.00323905 | 0.00613505 |
| set6 | BT0928 | 0.00223259 | 0.000771933 | 1.76E-11 | 3.87E-10 | 7.66385E-05 | 0.000797338 |
| set6 | BT0955 | 0.00000385 | 0.00000558 | 4.01E-08 | 0.000000174 | 0.000384777 | 0.001708964 |
| set6 | BT0966 | 0.00116495 | 0.000441515 | 0.00000017 | 0.000000554 | 0.0024581 | 0.005025476 |
| set6 | BT0980 | 4.63028E-05 | 0.0000323 | 1.8E-11 | 3.87E-10 | 0.000369845 | 0.001695937 |
| set6 | BT0998 | 0.953055 | 0.163197 | 7.14E-09 | 4.07E-08 | 0.00000743 | 0.000279479 |
| set6 | BT1024 | 0.427632 | 0.08241871 | 9.32E-12 | 2.45E-10 | 4.71715E-05 | 0.000643974 |
| set6 | BT1034 | 0.703634 | 0.1264372 | 8.14E-08 | 0.000000312 | 0.00344751 | 0.006405906 |
| set6 | BT1067 | 0.00204565 | 0.000714478 | 8.85E-13 | 4.11E-11 | 0.00384088 | 0.006884128 |
| set6 | BT1095 | 0.00000374 | 0.00000551 | 4.73E-11 | 8.4E-10 | 0.00010898 | 0.000946591 |
| set6 | BT1119 | 0.104625 | 0.02372279 | 7.57E-12 | 2.08E-10 | 0.000145671 | 0.001079252 |
| set6 | BT1141 | 1.30702E-05 | 0.0000128 | 1.46E-10 | 2.13E-09 | 0.00236338 | 0.004907002 |
| set6 | BT1150 | 0.000114363 | 0.0000642 | 0.000000819 | 0.00000202 | 0.0015178 | 0.003782695 |
| set6 | BT1186 | 0.00279573 | 0.000944634 | 1.08E-09 | 9.76E-09 | 0.00000785 | 0.000283486 |
| set6 | BT1207 | 0.273883 | 0.05578352 | 2.49E-11 | 5.19E-10 | 1.50914E-05 | 0.00041024 |
| set6 | BT1236 | 0.000101285 | 0.0000587 | 5.84E-09 | 3.51E-08 | 0.000192942 | 0.001210356 |
| set6 | BT1240 | 0.00403796 | 0.001304386 | 3.68E-12 | 1.27E-10 | 0.00429271 | 0.007404643 |
| set6 | BT1254 | 0.666235 | 0.1206912 | 3.25E-08 | 0.000000145 | 1.45304E-05 | 0.000402443 |
| set6 | BT1273 | 0.169052 | 0.03636981 | 8.76E-10 | 8.08E-09 | 0.000054678 | 0.00068507 |
| set6 | BT1291 | 0.000031507 | 0.0000242 | 9.74E-14 | 7.84E-12 | 1.81653E-05 | 0.000437134 |
| set6 | BT1306 | 0.0251028 | 0.006673463 | 6.06E-10 | 6.25E-09 | 0.00084675 | 0.00274013 |
| set6 | BT1327 | 0.00457442 | 0.001462079 | 0.00000309 | 0.00000622 | 0.00510723 | 0.008283978 |
| set6 | BT1387 | 0.00161889 | 0.000584578 | 1.81E-09 | 1.41E-08 | 0.000235647 | 0.001335563 |
| set6 | BT1395 | 0.0105587 | 0.003061868 | 0.00000377 | 0.00000744 | 0.00306076 | 0.00588853 |
| set6 | BT1472 | 0.00000364 | 0.00000549 | 7.6E-11 | 1.24E-09 | 0.00104326 | 0.003086115 |
| set6 | BT1496 | 0.118315 | 0.02643075 | 2.73E-08 | 0.000000125 | 0.000248054 | 0.001351611 |
| set6 | BT1530 | 0.00324998 | 0.001074142 | 0.000000988 | 0.00000236 | 0.00499894 | 0.008198927 |
| set6 | BT1538 | 0.00196261 | 0.000688653 | 2.12E-08 | 0.000000101 | 0.00224028 | 0.00473939 |
| set6 | BT1541 | 0.000465173 | 0.000200268 | 2.03E-09 | 1.52E-08 | 2.34371E-05 | 0.000471285 |
| set6 | BT1606 | 0.0557692 | 0.01364681 | 1.19E-09 | 1.04E-08 | 0.00000154 | 0.000119201 |
| set6 | BT1607 | 0.0204474 | 0.005544914 | 3.04E-08 | 0.000000136 | 5.35702E-05 | 0.000683798 |
| set6 | BT1628 | 1.18075E-05 | 0.0000119 | 3.14E-10 | 3.62E-09 | 1.98771E-05 | 0.000448892 |
| set6 | BT1632 | 0.266414 | 0.05443806 | 9.57E-09 | 5.16E-08 | 0.000202268 | 0.001242379 |
| set6 | BT1635 | 3.78496E-05 | 0.0000278 | 6.91E-12 | 2.06E-10 | 0.00000128 | 0.000119201 |
| set6 | BT1640 | 9.38302E-05 | 0.0000557 | 4.03E-11 | 7.5E-10 | 0.000638666 | 0.002264517 |
| set6 | BT1644 | 0.156771 | 0.03396907 | 0.00000658 | 0.0000118 | 0.00592755 | 0.009175283 |
| set6 | BT1651 | 3.82073E-05 | 0.000028 | 0.000000565 | 0.00000148 | 0.00378475 | 0.006800131 |
| set6 | BT1652 | 0.00100341 | 0.000388841 | 0.00000119 | 0.00000275 | 0.00298964 | 0.005797291 |
| set6 | BT1654 | 0.705344 | 0.1267144 | 1.36E-09 | 1.16E-08 | 0.00575557 | 0.009030287 |
| set6 | BT1671 | 0.0020073 | 0.000702056 | 3.7E-10 | 4.14E-09 | 5.26967E-05 | 0.000683798 |
| set6 | BT1752 | 0.0605145 | 0.01468926 | 2.34E-10 | 2.95E-09 | 0.00206061 | 0.004507912 |
| set6 | BT1771 | 2.15196E-05 | 0.0000182 | 2.25E-10 | 2.89E-09 | 0.000040354 | 0.00061981 |
| set6 | BT1776 | 3.01586E-05 | 0.0000235 | 0.000000541 | 0.00000143 | 0.000256647 | 0.001364989 |
| set6 | BT1823 | 0.000109352 | 0.0000625 | 0.00000823 | 0.0000141 | 0.00620023 | 0.009421776 |
| set6 | BT1828 | 7.47664E-05 | 0.0000465 | 0.0000034 | 0.00000675 | 0.00127089 | 0.003404313 |
| set6 | BT1873 | 0.000167488 | 0.0000867 | 0.0124539 | 0.0072152 | 1.71507E-05 | 0.000428224 |
| set6 | BT1876 | 0.00224947 | 0.00077706 | 2.11E-09 | 1.56E-08 | 0.005146 | 0.008328458 |
| set6 | BT1888 | 0.00125211 | 0.000469146 | 2.82E-09 | 1.98E-08 | 8.64506E-05 | 0.000863083 |
| set6 | BT1935 | 0.236206 | 0.04897953 | 1.87E-12 | 7.29E-11 | 0.000388132 | 0.001716104 |
| set6 | BT1936 | 4.37746E-05 | 0.0000309 | 8.16E-09 | 4.52E-08 | 0.00036093 | 0.001681956 |
| set6 | BT1958 | 0.000155676 | 0.0000818 | 0.000000525 | 0.00000139 | 0.000461818 | 0.001888333 |
| set6 | BT2013 | 0.00327668 | 0.001081078 | 0.00000113 | 0.00000263 | 0.00581307 | 0.00905851 |
| set6 | BT2018 | 0.335172 | 0.06663485 | 0.00000206 | 0.00000438 | 0.00214536 | 0.004624398 |
| set6 | BT2024 | 0.00000352 | 0.00000534 | 6.8E-09 | 3.91E-08 | 5.96479E-05 | 0.000722189 |
| set6 | BT2050 | 0.000342228 | 0.000155754 | 1.28E-09 | 1.11E-08 | 0.00330205 | 0.006222262 |
| set6 | BT2077 | 0.00463872 | 0.001478884 | 1.09E-11 | 2.74E-10 | 0.000146045 | 0.001079252 |
| set6 | BT2092 | 2.66168E-05 | 0.0000213 | 9.78E-11 | 1.5E-09 | 0.000335912 | 0.001590618 |
| set6 | BT2127 | 1.61984E-05 | 0.0000148 | 1.95E-09 | 0.000000015 | 1.74159E-05 | 0.000428224 |
| set6 | BT2128 | 0.00000241 | 0.00000414 | 3.02E-10 | 3.56E-09 | 2.27612E-05 | 0.00046405 |
| set6 | BT2146 | 2.06392E-05 | 0.0000176 | 0.00000416 | 0.00000806 | 0.0017869 | 0.004137263 |
| set6 | BT2194 | 0.00000328 | 0.00000508 | 1.47E-12 | 6.36E-11 | 0.000231201 | 0.00132572 |
| set6 | BT2199 | 0.00409674 | 0.001320556 | 8.3E-09 | 4.55E-08 | 0.00167106 | 0.003995083 |
| set6 | BT2203 | 0.703269 | 0.1264372 | 0.000000597 | 0.00000155 | 0.00200154 | 0.004431522 |
| set6 | BT2210 | 0.0997368 | 0.02273704 | 0.000000145 | 0.000000494 | 0.000141696 | 0.001068886 |
| set6 | BT2279 | 0.000393641 | 0.000174535 | 8.17E-08 | 0.000000312 | 0.00169615 | 0.00402882 |
| set6 | BT2307 | 0.000242883 | 0.000118446 | 2.45E-10 | 3.02E-09 | 0.000323878 | 0.001574261 |
| set6 | BT2308 | 0.000680271 | 0.000276664 | 4.2E-10 | 4.53E-09 | 2.90096E-05 | 0.000532771 |
| set6 | BT2318 | 0.000139663 | 0.0000748 | 0.000000174 | 0.000000562 | 0.00189091 | 0.004276892 |
| set6 | BT2361 | 0.623784 | 0.114342 | 0.0000022 | 0.00000464 | 0.00109913 | 0.003168431 |
| set6 | BT2363 | 0.0000048 | 0.00000644 | 5.77E-08 | 0.000000234 | 0.000295448 | 0.001475149 |
| set6 | BT2366 | 0.000205587 | 0.000102979 | 0.00000015 | 0.000000506 | 0.000380105 | 0.001695937 |
| set6 | BT2409 | 0.00010622 | 0.000061 | 1.2E-10 | 1.8E-09 | 8.87806E-05 | 0.000868818 |
| set6 | BT2491 | 0.0010886 | 0.000417386 | 1.64E-08 | 0.000000082 | 0.000516279 | 0.001981583 |
| set6 | BT2494 | 0.000701032 | 0.000283583 | 1.12E-09 | 0.00000001 | 0.00230632 | 0.004829517 |
| set6 | BT2512 | 0.00000529 | 0.0000068 | 6.46E-09 | 3.75E-08 | 6.52658E-05 | 0.000725795 |
| set6 | BT2524 | 0.0180753 | 0.004971113 | 3.95E-09 | 2.58E-08 | 0.000701519 | 0.002383734 |
| set6 | BT2562 | 0.0174785 | 0.004819238 | 1.82E-09 | 1.41E-08 | 7.31467E-05 | 0.000782832 |
| set6 | BT2581 | 2.92671E-05 | 0.000023 | 6.23E-10 | 6.32E-09 | 0.00227376 | 0.004778612 |
| set6 | BT2584 | 8.02094E-05 | 0.0000495 | 2.29E-14 | 3.46E-12 | 0.00000568 | 0.00023406 |
| set6 | BT2681 | 3.38435E-05 | 0.0000256 | 0.000000161 | 0.000000537 | 0.00172576 | 0.004055724 |
| set6 | BT2684 | 0.000115174 | 0.0000646 | 0.000000231 | 0.000000713 | 0.00359124 | 0.00657313 |
| set6 | BT2748 | 0.000358864 | 0.000162073 | 0.00000249 | 0.00000513 | 0.00373902 | 0.006759334 |
| set6 | BT2820 | 0.189652 | 0.04021864 | 0.000199981 | 0.000205066 | 0.000000436 | 0.000064 |
| set6 | BT2851 | 5.23329E-05 | 0.0000352 | 7.22E-10 | 7.09E-09 | 0.000210911 | 0.001274879 |
| set6 | BT2858 | 0.0146793 | 0.004127118 | 1.05E-08 | 5.57E-08 | 0.00140885 | 0.00358419 |
| set6 | BT2860 | 0.00677011 | 0.002064479 | 1.01E-09 | 9.27E-09 | 0.00509341 | 0.008283978 |
| set6 | BT2945 | 8.08676E-05 | 0.0000496 | 1.84E-10 | 2.56E-09 | 0.00000792 | 0.000283486 |
| set6 | BT2967 | 0.000228779 | 0.000112584 | 6.37E-10 | 6.41E-09 | 6.72803E-05 | 0.000735177 |
| set6 | BT3018 | 0.00000921 | 0.00000996 | 0.000000734 | 0.00000183 | 0.000677372 | 0.002350656 |
| set6 | BT3051 | 0.00336732 | 0.001106796 | 1.07E-08 | 5.62E-08 | 0.00426553 | 0.007366415 |
| set6 | BT3095 | 1.99151E-05 | 0.0000172 | 2.35E-12 | 8.87E-11 | 0.000117796 | 0.000966006 |
| set6 | BT3102 | 0.00431722 | 0.001385137 | 0.000000236 | 0.000000723 | 0.00207421 | 0.004524179 |
| set6 | BT3109 | 0.000328011 | 0.000150096 | 0.000000487 | 0.00000131 | 0.000632107 | 0.002256637 |
| set6 | BT3113 | 0.000438221 | 0.000190507 | 3.77E-11 | 7.23E-10 | 0.000345617 | 0.001626083 |
| set6 | BT3114 | 2.72883E-05 | 0.0000217 | 0.000000164 | 0.000000542 | 0.000264534 | 0.001379763 |
| set6 | BT3139 | 6.78877E-05 | 0.0000431 | 5.73E-08 | 0.000000233 | 0.000175644 | 0.001149913 |
| set6 | BT3185 | 0.000126027 | 0.0000693 | 0.000000466 | 0.00000126 | 0.000270577 | 0.001379763 |
| set6 | BT3189 | 0.0293018 | 0.007650021 | 0.00000181 | 0.00000393 | 0.00193106 | 0.004340949 |
| set6 | BT3202 | 0.0195936 | 0.005328655 | 5.87E-08 | 0.000000237 | 0.00593176 | 0.009175283 |
| set6 | BT3217 | 0.00000715 | 0.00000839 | 3.56E-08 | 0.000000157 | 0.00123997 | 0.003386255 |
| set6 | BT3218 | 9.37801E-05 | 0.0000557 | 1.06E-08 | 5.59E-08 | 0.00068098 | 0.002355848 |
| set6 | BT3222 | 0.0316617 | 0.00819266 | 7.48E-20 | 9.04E-17 | 0.000191652 | 0.001210356 |
| set6 | BT3241 | 0.544931 | 0.1017359 | 0.00000193 | 0.00000416 | 0.000891329 | 0.002821065 |
| set6 | BT3256 | 0.15166 | 0.03297491 | 2.49E-08 | 0.000000116 | 0.000163122 | 0.001124178 |
| set6 | BT3290 | 1.99695E-05 | 0.0000172 | 5.27E-09 | 3.23E-08 | 0.000164299 | 0.001126179 |
| set6 | BT3311 | 9.39055E-05 | 0.0000557 | 5.58E-10 | 5.97E-09 | 0.000503598 | 0.00196062 |
| set6 | BT3313 | 0.000515647 | 0.000218763 | 4.83E-09 | 3.04E-08 | 0.00474094 | 0.00787765 |
| set6 | BT3341 | 6.04521E-05 | 0.0000392 | 0.00000228 | 0.00000477 | 0.00115777 | 0.003260829 |
| set6 | BT3343 | 0.000117604 | 0.0000656 | 9.61E-12 | 2.47E-10 | 0.000175101 | 0.001149913 |
| set6 | BT3391 | 1.85606E-05 | 0.0000164 | 1.63E-12 | 6.79E-11 | 0.00000372 | 0.000188306 |
| set6 | BT3395 | 0.203595 | 0.04291096 | 1.51E-09 | 1.23E-08 | 0.0024529 | 0.00502229 |
| set6 | BT3408 | 0.0205266 | 0.005562405 | 1.55E-08 | 7.81E-08 | 0.000148196 | 0.001080723 |
| set6 | BT3410 | 0.000181538 | 0.0000928 | 0.00000011 | 0.000000389 | 0.000302898 | 0.001492046 |
| set6 | BT3465 | 0.0046625 | 0.001485214 | 0.00000522 | 0.00000974 | 0.00219519 | 0.004670089 |
| set6 | BT3474 | 0.0624538 | 0.01509706 | 1.28E-11 | 3.08E-10 | 0.000150004 | 0.001084698 |
| set6 | BT3483 | 0.208543 | 0.043771 | 0.00000906 | 0.0000153 | 0.00484277 | 0.007996394 |
| set6 | BT3496 | 0.00000593 | 0.00000741 | 1.89E-10 | 2.59E-09 | 0.000067612 | 0.000735177 |
| set6 | BT3508 | 0.185853 | 0.03949046 | 7.46E-09 | 4.23E-08 | 0.00156016 | 0.003841375 |
| set6 | BT3510 | 0.00109121 | 0.000418175 | 0.00000127 | 0.0000029 | 0.00147344 | 0.003697249 |
| set6 | BT3530 | 0.000248497 | 0.000120178 | 3.01E-10 | 3.56E-09 | 0.0012932 | 0.003433763 |
| set6 | BT3531 | 0.0021448 | 0.000744641 | 3.44E-08 | 0.000000152 | 4.83666E-05 | 0.000643974 |
| set6 | BT3534 | 0.000339922 | 0.000154891 | 1.72E-10 | 2.46E-09 | 0.00024229 | 0.0013492 |
| set6 | BT3589 | 0.0420794 | 0.01059138 | 1.13E-09 | 0.00000001 | 0.000114804 | 0.000952107 |
| set6 | BT3613 | 7.82E-08 | 0.000000713 | 0.0134485 | 0.007710071 | 0.00000151 | 0.000119201 |
| set6 | BT3647 | 0.683747 | 0.1234793 | 2.18E-08 | 0.000000103 | 0.000559223 | 0.002078213 |
| set6 | BT3650 | 0.000162256 | 0.0000843 | 8.94E-08 | 0.00000033 | 0.00529694 | 0.008501754 |
| set6 | BT3664 | 0.341013 | 0.06758464 | 3.21E-14 | 3.88E-12 | 0.000043041 | 0.000625552 |
| set6 | BT3667 | 0.000719151 | 0.000289829 | 0.000000551 | 0.00000145 | 0.00385731 | 0.006905145 |
| set6 | BT3696 | 0.0691266 | 0.01647332 | 0.00000713 | 0.0000125 | 0.0060747 | 0.009298403 |
| set6 | BT3720 | 3.12954E-05 | 0.0000241 | 0.000000971 | 0.00000233 | 0.00219374 | 0.004670089 |
| set6 | BT3748 | 0.00016638 | 0.0000863 | 0.000000123 | 0.000000429 | 0.000227247 | 0.001324944 |
| set6 | BT3764 | 0.0704208 | 0.01672382 | 0.000000071 | 0.000000279 | 1.14482E-05 | 0.000359358 |
| set6 | BT3782 | 0.351721 | 0.069324 | 0.000000108 | 0.000000382 | 0.00031168 | 0.001520003 |
| set6 | BT3819 | 0.0250572 | 0.006663681 | 7.88E-08 | 0.000000303 | 0.000451221 | 0.001855339 |
| set6 | BT3881 | 3.84075E-05 | 0.0000281 | 2.65E-11 | 5.42E-10 | 0.00450837 | 0.007635719 |
| set6 | BT3909 | 0.785546 | 0.1386867 | 1.37E-09 | 1.16E-08 | 0.00126899 | 0.003404313 |
| set6 | BT3935 | 0.00771887 | 0.002320142 | 2.32E-10 | 2.95E-09 | 0.000801809 | 0.00265088 |
| set6 | BT3936 | 5.50987E-05 | 0.0000366 | 3.22E-09 | 2.18E-08 | 0.00095711 | 0.002945409 |
| set6 | BT3949 | 0.174127 | 0.03735546 | 0.000000174 | 0.000000562 | 0.00012857 | 0.001025708 |
| set6 | BT3952 | 1.81663E-05 | 0.0000162 | 0.000000289 | 0.000000844 | 0.00471173 | 0.007859589 |
| set6 | BT3968 | 8.04E-09 | 0.000000225 | 0.000000002 | 1.52E-08 | 0.00000061 | 0.0000747 |
| set6 | BT3978 | 0.000309468 | 0.000143608 | 0.00000123 | 0.00000282 | 0.000997202 | 0.003005774 |
| set6 | BT3996 | 0.0431252 | 0.01082581 | 4.04E-11 | 7.5E-10 | 4.63992E-05 | 0.00064255 |
| set6 | BT4037 | 2.31854E-05 | 0.0000192 | 1.53E-09 | 1.24E-08 | 0.00104488 | 0.003086115 |
| set6 | BT4056 | 0.030566 | 0.007949909 | 1.13E-08 | 0.000000059 | 0.00122255 | 0.003354401 |
| set6 | BT4058 | 0.00114339 | 0.000434868 | 3.5E-10 | 3.95E-09 | 0.000144307 | 0.001079252 |
| set6 | BT4097 | 8.77858E-05 | 0.0000528 | 6.7E-11 | 1.11E-09 | 0.000267433 | 0.001379763 |
| set6 | BT4098 | 0.00301261 | 0.001004905 | 1.16E-13 | 8.34E-12 | 1.16706E-05 | 0.000359358 |
| set6 | BT4133 | 0.00000138 | 0.0000031 | 0.00840515 | 0.005143366 | 0.00000661 | 0.000255442 |
| set6 | BT4151 | 0.632033 | 0.1155742 | 0.00000198 | 0.00000424 | 0.00409005 | 0.007195528 |
| set6 | BT4158 | 0.019282 | 0.005262823 | 2.55E-08 | 0.000000118 | 0.000227487 | 0.001324944 |
| set6 | BT4181 | 0.000031979 | 0.0000245 | 4.42E-12 | 1.48E-10 | 9.20209E-05 | 0.000894565 |
| set6 | BT4224 | 0.00000128 | 0.00000297 | 7.09E-10 | 7.02E-09 | 0.00032621 | 0.001580363 |
| set6 | BT4230 | 0.00000242 | 0.00000414 | 2.3E-09 | 1.67E-08 | 0.000465905 | 0.001899752 |
| set6 | BT4252 | 2.18179E-05 | 0.0000184 | 0.00344444 | 0.002344063 | 0.00000277 | 0.000154091 |
| set6 | BT4257 | 5.50004E-05 | 0.0000366 | 3.91E-09 | 2.57E-08 | 0.000147967 | 0.001080723 |
| set6 | BT4265 | 0.00147507 | 0.000539075 | 2.45E-10 | 3.02E-09 | 3.60748E-05 | 0.000608677 |
| set6 | BT4269 | 0.116706 | 0.02614096 | 1.81E-09 | 1.41E-08 | 0.00252143 | 0.005147782 |
| set6 | BT4324 | 0.00027624 | 0.000131743 | 1.5E-11 | 3.47E-10 | 0.00000265 | 0.000154091 |
| set6 | BT4341 | 0.000203599 | 0.00010239 | 8.55E-08 | 0.000000318 | 0.000110788 | 0.000948054 |
| set6 | BT4342 | 0.000232579 | 0.000114009 | 0.000000305 | 0.000000881 | 0.00199698 | 0.004431522 |
| set6 | BT4352 | 0.947306 | 0.1623595 | 0.000000676 | 0.00000172 | 0.00108251 | 0.003159119 |
| set6 | BT4382 | 0.000049886 | 0.000034 | 2.58E-09 | 1.82E-08 | 0.00000283 | 0.000154091 |
| set6 | BT4394 | 2.97996E-05 | 0.0000233 | 2.02E-13 | 1.22E-11 | 2.21859E-05 | 0.00046405 |
| set6 | BT4430 | 0.00000158 | 0.00000336 | 0.000000013 | 6.64E-08 | 0.000183164 | 0.001178599 |
| set6 | BT4458 | 0.799198 | 0.1406376 | 0.000000268 | 0.000000797 | 0.000141992 | 0.001068886 |
| set6 | BT4475 | 0.08571 | 0.01988067 | 0.000000239 | 0.000000727 | 0.00440331 | 0.007542244 |
| set6 | BT4476 | 0.00141209 | 0.000519571 | 1.77E-12 | 7.15E-11 | 0.0051055 | 0.008283978 |
| set6 | BT4477 | 0.00000413 | 0.00000584 | 2.52E-10 | 3.08E-09 | 0.000553724 | 0.002063 |
| set6 | BT4486 | 0.00871182 | 0.002579673 | 3.28E-08 | 0.000000145 | 0.000158557 | 0.001118985 |
| set6 | BT4497 | 0.00837528 | 0.00248976 | 5.73E-09 | 3.46E-08 | 0.000743616 | 0.002497868 |
| set6 | BT4510 | 0.650778 | 0.1184012 | 0.00000669 | 0.0000119 | 0.00474806 | 0.00787765 |
| set6 | BT4515 | 0.000110327 | 0.0000626 | 2.69E-08 | 0.000000124 | 0.00398483 | 0.007055981 |
| set6 | BT4529 | 0.501922 | 0.09511406 | 0.000000166 | 0.000000543 | 0.000582658 | 0.002141782 |
| set6 | BT4544 | 1.82825E-05 | 0.0000163 | 0.000000248 | 0.000000749 | 0.00022935 | 0.001324944 |
| set6 | BT4551 | 0.000106004 | 0.0000609 | 9.37E-08 | 0.000000343 | 0.00175276 | 0.004098743 |
| set6 | BT4562 | 0.514506 | 0.09696462 | 2.12E-10 | 2.84E-09 | 5.90698E-05 | 0.000722189 |
| set6 | BT4563 | 0.0162046 | 0.004500786 | 0.00000102 | 0.00000243 | 0.00413485 | 0.007236789 |
| set6 | BT4635 | 0.824678 | 0.1445836 | 0.000000254 | 0.000000762 | 0.0057642 | 0.009030287 |
| set6 | BT4652 | 6.39816E-05 | 0.0000411 | 8.49E-08 | 0.000000318 | 0.00191949 | 0.004321558 |
| set6 | BT4675 | 0.00000103 | 0.00000259 | 1.74E-09 | 1.38E-08 | 0.000100612 | 0.000923064 |
| set6 | BT4702 | 0.00934065 | 0.00274867 | 1.09E-08 | 5.72E-08 | 0.00134317 | 0.00349651 |
| set6 | BT4714 | 0.0013124 | 0.000488831 | 9.26E-11 | 1.45E-09 | 0.000672526 | 0.002344926 |
| set6 | BT4716 | 0.00000237 | 0.00000412 | 5.37E-09 | 3.28E-08 | 8.13993E-05 | 0.000824052 |
| set6 | BT4724 | 1.14086E-05 | 0.0000116 | 0.000000234 | 0.000000721 | 0.00104016 | 0.003086115 |
| set6 | BT4741 | 0.00344821 | 0.001131261 | 3.8E-10 | 4.18E-09 | 0.00526178 | 0.008487766 |
| set6 | SusR | 0.000821192 | 0.000326948 | 0.00000743 | 0.000013 | 0.00473368 | 0.00787765 |
| set7 | BT0070 | 0.000000835 | 0.00000232 | 8.86E-08 | 0.000000328 | 0.00112874 | 0.003223539 |
| set7 | BT0086 | 0.000000177 | 0.000001 | 2.73E-10 | 3.3E-09 | 7.60283E-05 | 0.000797167 |
| set7 | BT0103 | 3.23E-10 | 0.000000111 | 1.21E-08 | 6.25E-08 | 0.0056926 | 0.008965962 |
| set7 | BT0145 | 0.000000664 | 0.00000202 | 6.45E-11 | 1.08E-09 | 0.00179542 | 0.004140705 |
| set7 | BT0191 | 0.00000341 | 0.00000521 | 3.5E-12 | 1.24E-10 | 0.00508106 | 0.008278119 |
| set7 | BT0277 | 0.000000593 | 0.0000019 | 4.46E-11 | 8.04E-10 | 0.000905638 | 0.002852797 |
| set7 | BT0563 | 0.0226543 | 0.006078048 | 1.55E-08 | 7.81E-08 | 2.07639E-05 | 0.000454921 |
| set7 | BT0696 | 4.94E-09 | 0.000000216 | 9.56E-09 | 5.16E-08 | 0.00000405 | 0.000198137 |
| set7 | BT0838 | 0.000000296 | 0.00000127 | 0.000000496 | 0.00000133 | 0.00354858 | 0.006519435 |
| set7 | BT0939 | 9.73E-08 | 0.000000827 | 5.35E-11 | 9.36E-10 | 2.92904E-05 | 0.000532771 |
| set7 | BT0987 | 0.000000361 | 0.00000142 | 4.03E-13 | 2.29E-11 | 0.000476246 | 0.001906264 |
| set7 | BT1093 | 0.00000158 | 0.00000336 | 2.2E-10 | 2.88E-09 | 0.00132648 | 0.003475991 |
| set7 | BT1136 | 0.00000063 | 0.00000198 | 1.41E-09 | 1.17E-08 | 0.000627097 | 0.002255036 |
| set7 | BT1397 | 0.000000278 | 0.00000127 | 1.21E-10 | 1.8E-09 | 0.000242626 | 0.0013492 |
| set7 | BT1444 | 0.000000586 | 0.00000189 | 4.14E-15 | 7.15E-13 | 0.000176019 | 0.001149913 |
| set7 | BT1490 | 0.000000137 | 0.000000919 | 3.5E-10 | 3.95E-09 | 0.00014631 | 0.001079252 |
| set7 | BT1581 | 0.00000591 | 0.00000741 | 0.000000105 | 0.000000376 | 0.00476214 | 0.007889883 |
| set7 | BT1610 | 0.00000292 | 0.00000468 | 1.48E-09 | 1.22E-08 | 0.00279716 | 0.005541167 |
| set7 | BT1646 | 0.00000447 | 0.00000611 | 0.00000775 | 0.0000134 | 0.00672743 | 0.009934933 |
| set7 | BT1664 | 0.000000483 | 0.00000168 | 0.000000619 | 0.00000159 | 0.000417646 | 0.001790729 |
| set7 | BT1789 | 0.000947681 | 0.000369938 | 1.92E-15 | 4.65E-13 | 0.00000269 | 0.000154091 |
| set7 | BT2057 | 0.0265384 | 0.007018134 | 7.43E-12 | 2.08E-10 | 0.00000582 | 0.00023406 |
| set7 | BT2172 | 0.0000044 | 0.00000603 | 0.000149171 | 0.000161317 | 7.54E-09 | 0.0000111 |
| set7 | BT2186 | 0.000000224 | 0.00000111 | 0.000000253 | 0.00000076 | 0.000662626 | 0.002321435 |
| set7 | BT2201 | 0.000000298 | 0.00000127 | 5.14E-09 | 3.17E-08 | 0.000217548 | 0.001298142 |
| set7 | BT2236 | 0.000000317 | 0.0000013 | 0.000000106 | 0.000000381 | 0.000446378 | 0.001855339 |
| set7 | BT2255 | 0.013838 | 0.003918239 | 1.73E-10 | 2.46E-09 | 9.39E-08 | 0.0000245 |
| set7 | BT2401 | 6.64E-08 | 0.000000654 | 1.86E-08 | 9.05E-08 | 0.0060736 | 0.009298403 |
| set7 | BT2577 | 0.000000125 | 0.000000919 | 2.96E-15 | 5.96E-13 | 0.000654266 | 0.002302989 |
| set7 | BT2595 | 0.000000273 | 0.00000127 | 8.29E-11 | 1.32E-09 | 0.000267188 | 0.001379763 |
| set7 | BT2747 | 0.000000183 | 0.00000103 | 3.96E-10 | 4.31E-09 | 0.0045099 | 0.007635719 |
| set7 | BT2886 | 4.52E-08 | 0.000000576 | 0.00000108 | 0.00000254 | 0.00131696 | 0.003470719 |
| set7 | BT3096 | 0.0296947 | 0.007736586 | 1.34E-09 | 1.16E-08 | 4.43155E-05 | 0.000637761 |
| set7 | BT3231 | 0.000338416 | 0.000154524 | 9.92E-13 | 4.44E-11 | 0.000000069 | 0.0000245 |
| set7 | BT3245 | 0.00000555 | 0.00000703 | 0.000000513 | 0.00000137 | 0.00294909 | 0.005763323 |
| set7 | BT3252 | 0.0027835 | 0.000941343 | 6.38E-14 | 5.93E-12 | 0.0000019 | 0.000126467 |
| set7 | BT3462 | 0.014937 | 0.004187127 | 6.74E-09 | 3.89E-08 | 2.50556E-05 | 0.000490395 |
| set7 | BT3542 | 1.31E-09 | 0.000000165 | 4.59E-13 | 2.41E-11 | 2.25994E-05 | 0.00046405 |
| set7 | BT3557 | 6.28E-09 | 0.000000222 | 0.00000397 | 0.00000779 | 0.00295255 | 0.005763323 |
| set7 | BT3570 | 0.00000474 | 0.00000638 | 0.00000109 | 0.00000258 | 0.00387376 | 0.006926147 |
| set7 | BT3722 | 7.19E-09 | 0.000000225 | 0.000000399 | 0.00000111 | 0.00175991 | 0.004106358 |
| set7 | BT3880 | 0.000000997 | 0.00000256 | 0.000000218 | 0.000000681 | 0.00337234 | 0.006322251 |
| set7 | BT3956 | 0.00000166 | 0.00000342 | 8.11E-09 | 4.51E-08 | 0.000584268 | 0.002141782 |
| set7 | BT4053 | 0.00000142 | 0.00000316 | 0.000000006 | 3.58E-08 | 0.000696635 | 0.002372631 |
| set7 | BT4334 | 0.000206118 | 0.000103177 | 8.5E-13 | 4.11E-11 | 0.000000291 | 0.0000534 |
| set7 | BT4482 | 0.000000311 | 0.00000128 | 0.00000113 | 0.00000263 | 0.000504874 | 0.00196062 |
| set7 | BT4735 | 1.57E-08 | 0.000000297 | 3.21E-09 | 2.18E-08 | 0.000107801 | 0.000941924 |
| set7 | BT4748 | 0.00000181 | 0.00000357 | 0.000000588 | 0.00000153 | 0.00285201 | 0.005604444 |
| set7 | BT4765 | 1.30873E-05 | 0.0000128 | 1.29747E-05 | 0.0000208 | 0.00676947 | 0.009971554 |
| set7 | BT4767 | 0.000000515 | 0.00000175 | 6.34E-09 | 3.73E-08 | 0.000184051 | 0.001178599 |
| set7 | BT4775 | 0.00000599 | 0.00000742 | 0.00000287 | 0.00000583 | 0.00447766 | 0.007618908 |
| set7 | tRNA_29 | 0.000000117 | 0.000000902 | 0.000000351 | 0.000000995 | 0.000176645 | 0.001149913 |
| set7 | tRNA_49 | 0.000000165 | 0.000001 | 0.000000718 | 0.00000179 | 0.00352655 | 0.006501224 |
