# Supplementary material for: Genomic and Metabolic Studies of the Impact of Probiotics on a Model Gut Symbiont and Host
Source: PLoS Biol. 2006 Nov 28;4(12):e413. doi: 10.1371/journal.pbio.0040413 (PMC1661682; doi:10.1371/journal.pbio.0040413)
Supplement: Supplementary file 15 [file pbio.0040413.st006.doc]

**Table S6.** COG functional group-based categorization of *B. thetaiotaomicron* genes that are more highly expressed in the ceca of NMRI mice compared to B6 mice irrespective of colonization state.

| **COG function** | **Gene** | **Description** | **Fold difference in expression (NMRI vs. B6)** |
| --- | --- | --- | --- |
| [Translation, ribosomal structure and biogenesis](http://genome.wustl.edu/GSCGAP/gut/cgi-bin/geneDisplay.cgi?cogId=J&sequencedOrganismId=1) |  |  |  |
|  | BT0423 | translation initiation factor IF-3 | 1.5 |
|  | BT0424 | 50S ribosomal protein L35 | 2.3 |
|  | BT0566 | alanyl-tRNA synthetase | 2.7 |
|  | BT0810 | hypothetical protein | 1.7 |
|  | BT0815 | conserved hypothetical protein | 1.3 |
|  | BT0872 | aspartyl-tRNA synthetase | 1.8 |
|  | BT0893 | tRNA (Guanine-N(1)-methyltransferase | 1.7 |
|  | BT0916 | 50S ribosomal protein L28 | 1.4 |
|  | BT1039 | hypothetical protein | 2.6 |
|  | BT1325 | glutaminyl-tRNA synthetase | 1.6 |
|  | BT1360 | tRNA/rRNA methyltransferase | 1.4 |
|  | BT1500 | Ribonuclease G | 1.5 |
|  | BT1669 | phenylalanyl-tRNA synthetase alpha chain | 2.2 |
|  | BT1729 | peptide chain release factor 3 (RF-3) | 1.7 |
|  | BT1809 | conserved protein, with weak BamHI domain | 1.8 |
|  | BT2122 | lysyl-tRNA synthetase | 2.0 |
|  | BT2161 | 50S ribosomal protein L9 | 2.6 |
|  | BT2162 | 30S ribosomal protein S18 | 2.3 |
|  | BT2249 | ribosome recycling factor (ribosome releasing factor) | 1.2 |
|  | BT2438 | hypothetical protein | 1.2 |
|  | BT2563 | polyribonucleotide nucleotidyltransferase | 2.2 |
|  | BT2700 | 50S ribosomal protein L17 | 3.5 |
|  | BT2702 | 30S ribosomal protein S4 | 3.4 |
|  | BT2703 | 30S ribosomal protein S11 | 2.6 |
|  | BT2704 | 30S ribosomal protein S13 | 2.5 |
|  | BT2705 | translation initiation factor IF-1 | 3.6 |
|  | BT2706 | methionine aminopeptidase | 3.5 |
|  | BT2708 | 50S ribosomal protein L15 | 2.6 |
|  | BT2709 | 50S ribosomal protein L30 | 2.6 |
|  | BT2710 | 30S ribosomal protein S5 | 2.3 |
|  | BT2711 | 50S ribosomal protein L18 | 2.3 |
|  | BT2713 | 30S ribosomal protein S8 | 2.7 |
|  | BT2714 | chloroplast 30S ribosomal protein S14 | 2.5 |
|  | BT2715 | 50S ribosomal protein L5 | 3.1 |
|  | BT2716 | 50S ribosomal protein L24 | 2.2 |
|  | BT2717 | 50S ribosomal protein L14 | 2.6 |
|  | BT2718 | 30S ribosomal protein S17 | 3.2 |
|  | BT2719 | 50S ribosomal protein L29 | 2.3 |
|  | BT2720 | 50S ribosomal protein L16 | 2.6 |
|  | BT2721 | 30S ribosomal protein S3 | 2.1 |
|  | BT2722 | 50S ribosomal protein L22 | 2.1 |
|  | BT2723 | 30S ribosomal protein S19 | 1.8 |
|  | BT2724 | 50S ribosomal protein L2 | 2.0 |
|  | BT2725 | 50S ribosomal protein L23 | 1.9 |
|  | BT2727 | 50S ribosomal protein L3 | 2.4 |
|  | BT2728 | 30S ribosomal protein S10 | 2.0 |
|  | BT2729 | elongation factor G | 1.8 |
|  | BT2731 | 30S ribosomal protein S12 | 1.4 |
|  | BT2735 | 50S ribosomal protein L7/L12 | 2.3 |
|  | BT2736 | ribosomal protein L10 | 2.1 |
|  | BT2737 | 50S ribosomal protein L1 | 2.0 |
|  | BT2738 | 50S ribosomal protein L11 | 2.1 |
|  | BT2740 | elongation factor Tu | 1.8 |
|  | BT2819 | hypothetical protein | 3.7 |
|  | BT2829 | arginyl-tRNA synthetase | 1.2 |
|  | BT2839 | ribosome-binding factor A | 1.3 |
|  | BT2933 | Methionyl-tRNA synthetase | 2.1 |
|  | BT3116 | GTP-binding protein | 1.6 |
|  | BT3165 | tRNA/rRNA methyltransferase | 2.1 |
|  | BT3214 | S-adenosylmethionine:tRNA ribosyltransferase-isomerase | 2.5 |
|  | BT3230 | tyrosyl-tRNA synthetase | 1.8 |
|  | BT3326 | putative nifR3 family TIM-barrel enzyme with dihydrouridine synthase domain | 1.6 |
|  | BT3351 | cysteinyl-tRNA synthetase | 1.8 |
|  | BT3404 | translation initiation factor IF-2 | 1.4 |
|  | BT3611 | glycyl-tRNA synthetase | 1.7 |
|  | BT3709 | elongation factor P | 1.7 |
|  | BT3833 | 50S ribosomal protein L32 (Ribosomal protein I) | 1.3 |
|  | BT3864 | tryptophanyl-tRNA synthetase | 1.2 |
|  | BT3873 | asparaginyl-tRNA synthetase | 2.1 |
|  | BT3875 | 50S ribosomal protein L13 | 1.7 |
|  | BT3876 | 30S ribosomal protein S9 | 1.6 |
|  | BT3877 | 30S ribosomal protein S2 (BS1) | 1.9 |
|  | BT3878 | elongation factor Ts (EF-Ts) | 2.6 |
|  | BT3945 | methionyl-tRNA formyltransferase | 1.9 |
|  | BT3995 | alanyl-tRNA synthetase | 2.2 |
|  | BT4128 | conserved hypothetical protein | 1.2 |
|  | BT4295 | putative chitobiase | 3.5 |
|  | BT4314 | 50S ribosomal protein L21 | 1.6 |
|  | BT4323 | tRNA delta(2)-isopentenylpyrophosphate transferase | 1.8 |
|  | BT4332 | conserved hypothetical protein | 1.3 |
|  | BT4353 | valyl-tRNA synthetase | 1.7 |
|  | BT4588 | peptidyl-tRNA hydrolase | 2.1 |
|  | BT4589 | putative 50S ribosomal protein L25 | 1.9 |
|  |  |  |  |
| [Transcription](http://genome.wustl.edu/GSCGAP/gut/cgi-bin/geneDisplay.cgi?cogId=K&sequencedOrganismId=1) |  |  |  |
|  | BT0223 | two-component system response regulator | 1.2 |
|  | BT0248 | RNA polymerase ECF-type sigma factor | 1.6 |
|  | BT0376 | putative transcriptional regulator | 1.4 |
|  | BT0559 | transcriptional regulator | 1.5 |
|  | BT1103 | RNA polymerase ECF-type sigma factor | 1.9 |
|  | BT1265 | two-component system response regulator | 1.5 |
|  | BT1308 | clostripain-related protein | 1.5 |
|  | BT1311 | RNA polymerase sigma factor rpoD (Sigma-A) | 1.1 |
|  | BT1396 | transcription regulator, AraC family | 1.3 |
|  | BT1464 | putative acetyltransferase, (GNAT) family | 1.5 |
|  | BT1540 | transcriptional regulator | 1.4 |
|  | BT1656 | putative transcriptional regulator | 1.9 |
|  | BT1846 | putative dipeptidyl-peptidase III | 1.5 |
|  | BT1877 | RNA polymerase ECF-type sigma factor | 1.2 |
|  | BT1898 | hypothetical protein | 1.3 |
|  | BT2051 | conserved hypothetical protein | 1.8 |
|  | BT2169 | RNA polymerase ECF-type sigma factor | 1.6 |
|  | BT2372 | transcriptional regulator | 2.1 |
|  | BT2435 | MarR family transcriptional regulator | 1.7 |
|  | BT2463 | RNA polymerase ECF-type sigma factor | 2.4 |
|  | BT2493 | ROK family transcriptional repressor, with glucokinase domain | 1.8 |
|  | BT2509 | putative transcriptional regulator | 1.8 |
|  | BT2689 | conserved hypothetical protein | 1.5 |
|  | BT2701 | DNA-directed RNA polymerase alpha chain | 2.7 |
|  | BT2733 | DNA-directed RNA polymerase beta' chain | 1.4 |
|  | BT2734 | DNA-directed RNA polymerase beta chain | 3.2 |
|  | BT2739 | transcription anti-termination protein | 2.6 |
|  | BT2783 | transcriptional regulator | 1.3 |
|  | BT2978 | transcriptional regulator | 1.2 |
|  | BT3077 | ribonuclease R | 1.6 |
|  | BT3146 | hypothetical protein | 1.8 |
|  | BT3152 | ATP-dependent DNA helicase | 1.4 |
|  | BT3258 | two-component system response regulator | 1.4 |
|  | BT3277 | RNA polymerase ECF-type sigma factor | 1.9 |
|  | BT3756 | transcriptional regulator | 1.7 |
|  | BT3768 | transcriptional regulator | 1.5 |
|  | BT3993 | RNA polymerase ECF-type sigma factor | 1.7 |
|  | BT3997 | transcriptional regulator | 1.4 |
|  | BT4001 | putative chromosome partitioning protein parB | 1.9 |
|  | BT4054 | putative transcriptional regulator | 1.4 |
|  | BT4599 | putative transcriptional regulator | 2.3 |
|  | BT4628 | ATP-dependent helicase | 1.4 |
|  |  |  |  |
| [Replication, recombination and repair](http://genome.wustl.edu/GSCGAP/gut/cgi-bin/geneDisplay.cgi?cogId=L&sequencedOrganismId=1) |  |  |  |
|  | BT0069 | conserved hypothetical protein | 3.6 |
|  | BT0070 | conserved hypothetical protein | 1.4 |
|  | BT0252 | transcription-repair coupling factor | 1.2 |
|  | BT0578 | excinuclease ABC subunit A | 1.6 |
|  | BT0630 | exodeoxyribonuclease | 1.8 |
|  | BT0657 | ATP-dependent DNA helicase | 1.5 |
|  | BT0889 | conserved hypothetical protein | 1.7 |
|  | BT0912 | DNA-binding protein HU | 1.3 |
|  | BT1154 | ATP-independent RNA helicase | 1.6 |
|  | BT1205 | putative ATPase, AAA family | 2.0 |
|  | BT1363 | DNA Pol III Epsilon Chain | 3.1 |
|  | BT1503 | integrase | 1.5 |
|  | BT1664 | crossover junction endodeoxyribonuclease ruvC | 1.7 |
|  | BT1827 | transposase | 1.3 |
|  | BT1832 | conserved hypothetical protein | 1.4 |
|  | BT1904 | transcriptional regulator, AraC-type | 1.5 |
|  | BT2056 | conserved hypothetical protein | 2.0 |
|  | BT2143 | chromosomal replication initiator protein dnaA | 2.4 |
|  | BT2179 | putative DNA mismatch repair protein | 1.7 |
|  | BT2272 | conserved hypothetical protein | 1.3 |
|  | BT2752 | primosomal protein N' (replication factor Y) | 1.2 |
|  | BT3199 | putative non-specific DNA-binding protein | 2.1 |
|  | BT3255 | putative N6-adenine-specific DNA methylase | 1.5 |
|  | BT3412 | ribonuclease HII | 1.3 |
|  | BT3715 | hypothetical protein | 1.6 |
|  | BT3844 | ATP-dependent DNA helicase recQ | 1.3 |
|  | BT3851 | DNA mismatch repair protein mutL | 1.5 |
|  | BT3891 | hypothetical protein | 1.8 |
|  | BT3938 | ATP-dependent DNA helicase recQ | 1.4 |
|  | BT4041 | conserved hypothetical protein | 1.7 |
|  | BT4651 | endonuclease IV | 1.4 |
|  | BT4680 | DNA mismatch repair protein MutS | 2.1 |
|  | BT4754 | putative cytosine-specific methyltransferase | 1.2 |
|  |  |  |  |
| [Cell cycle control, cell division, chromosome partitioning](http://genome.wustl.edu/GSCGAP/gut/cgi-bin/geneDisplay.cgi?cogId=D&sequencedOrganismId=1) |  |  |  |
|  | BT0841 | hypothetical protein | 1.5 |
|  | BT1144 | conserved hypothetical protein | 1.2 |
|  | BT1676 | Maf protein | 1.9 |
|  | BT1945 | conserved protein found in conjugate transposon | 1.6 |
|  | BT3268 | glucose-inhibited division protein A | 1.9 |
|  | BT3444 | cell division protein FtsZ | 1.9 |
|  | BT3445 | cell division protein FtsA | 2.3 |
|  | BT3621 | amidase enhancer precursor | 1.4 |
|  |  |  |  |
| [Defense mechanisms](http://genome.wustl.edu/GSCGAP/gut/cgi-bin/geneDisplay.cgi?cogId=V&sequencedOrganismId=1) |  |  |  |
|  | BT0301 | ATP-binding transport protein natA (Na+ ABC transporter) | 1.3 |
|  | BT0563 | putative ABC transporter ATP-binding protein or permease protein | 1.2 |
|  | BT0694 | ABC transporter, ATP-binding protein | 1.6 |
|  | BT0695 | ABC transporter, permease protein | 2.7 |
|  | BT0702 | chloramphenicol acetyltransferase (CAT-III) | 1.5 |
|  | BT0925 | ABC transporter, ATP-binding protein | 5.0 |
|  | BT0956 | conserved hypothetical protein, putative transmembrane protein | 2.1 |
|  | BT1532 | ABC transporter permease | 1.8 |
|  | BT1533 | conserved hypothetical protein | 1.7 |
|  | BT1694 | transporter, AcrB/D/F family | 2.4 |
|  | BT1931 | putative type IIS restriction/modification enzyme | 1.3 |
|  | BT2794 | putative multidrug resistance protein | 1.5 |
|  | BT3119 | chloramphenicol acetyltransferase | 1.9 |
|  | BT3151 | hypothetical protein | 1.2 |
|  | BT3200 | putative N-acetylmuramoyl-L-alanine amidase | 1.8 |
|  | BT3212 | putative bacitracin resistance protein | 2.0 |
|  | BT3338 | AcrB/AcrD family multidrug resistance protein | 3.2 |
|  | BT3640 | lipoprotein releasing system ATP-binding protein | 1.6 |
|  | BT3902 | conserved hypothetical protein | 2.2 |
|  | BT3903 | conserved hypothetical protein | 1.7 |
|  | BT4318 | ATP-binding protein | 2.2 |
|  | BT4326 | ABC transporter ATP-binding protein | 2.0 |
|  | BT4507 | beta-lactamase precursor | 1.4 |
|  | BT4518 | Type I restriction enzyme StySJI M protein | 1.8 |
|  | BT4520 | Type I restriction enzyme EcoAI specificity protein | 1.7 |
|  |  |  |  |
| [Signal transduction mechanisms](http://genome.wustl.edu/GSCGAP/gut/cgi-bin/geneDisplay.cgi?cogId=T&sequencedOrganismId=1) |  |  |  |
|  | BT0682 | two-component system response regulator | 1.6 |
|  | BT0688 | cAMP-binding domain (catabolite gene activator) transcriptional regulator | 1.9 |
|  | BT0690 | two-component system response regulator | 1.4 |
|  | BT0733 | two-component system sensor histidine kinase | 1.2 |
|  | BT0856 | two-component system response regulator | 1.5 |
|  | BT1058 | two-component system sensor histidine kinase | 1.5 |
|  | BT1075 | putative two-component system sensor protein, but no histidine kinase domain | 1.4 |
|  | BT1183 | Hybrid two-component system | 1.4 |
|  | BT1413 | putative transcription regulator | 1.3 |
|  | BT1483 | two-component system sensor histidine kinase | 1.7 |
|  | BT1529 | two-component system response regulator | 1.9 |
|  | BT1597 | two-component system sensor histidine kinase | 1.3 |
|  | BT1742 | carbon starvation protein A | 1.6 |
|  | BT2165 | two-component system response regulator | 1.3 |
|  | BT2187 | putative cAMP-binding domain-containing regulatory protein | 1.3 |
|  | BT2391 | Hybrid two-component system | 1.6 |
|  | BT2408 | luxR family transcriptional regulator | 2.4 |
|  | BT2750 | putative protein-tyrosine-phosphatase | 1.7 |
|  | BT2770 | hypothetical protein | 1.7 |
|  | BT2787 | GTP-binding elongation factor family protein TypA/BipA | 1.4 |
|  | BT2897 | Hybrid two-component system | 1.5 |
|  | BT3172 | Hybrid two-component system | 1.3 |
|  | BT3304 | conserved hypothetical protein | 1.5 |
|  | BT3334 | Hybrid two-component system | 1.4 |
|  | BT3737 | hypothetical protein | 1.6 |
|  | BT3805 | two-component system sensor histidine kinase | 1.8 |
|  | BT3998 | GTP pyrophosphokinase | 1.8 |
|  | BT4218 | phosphate starvation-inducible protein, PhoH | 2.0 |
|  | BT4354 | two-component system sensor histidine kinase | 1.3 |
|  | BT4375 | transcriptional regulator | 1.6 |
|  | BT4673 | two-component system sensor histidine kinase | 1.3 |
|  | BT4756 | hypothetical protein | 1.4 |
|  |  |  |  |
| [Cell wall/membrane/envelope biogenesis](http://genome.wustl.edu/GSCGAP/gut/cgi-bin/geneDisplay.cgi?cogId=M&sequencedOrganismId=1) |  |  |  |
|  | BT0147 | hypothetical protein | 1.5 |
|  | BT0242 | putative polysaccharide deacetylase | 1.3 |
|  | BT0294 | hypothetical protein | 3.5 |
|  | BT0298 | cation efflux system protein (outer membrane protein) | 1.8 |
|  | BT0418 | outer membrane porin F precursor | 1.3 |
|  | BT0558 | mannose-1-phosphate guanylyltransferase | 1.7 |
|  | BT0561 | conserved hypothetical protein, putative membrane protein | 1.7 |
|  | BT0598 | putative nucleoside-diphosphate sugar epimerases/dehydrase | 1.3 |
|  | BT0656 | conserved hypothetical protein | 1.9 |
|  | BT0817 | lipoprotein, function unknown | 1.5 |
|  | BT0829 | UDP-glucose 6-dehydrogenase | 3.3 |
|  | BT0885 | putative cation efflux pump | 1.3 |
|  | BT0954 | conserved hypothetical protein | 1.3 |
|  | BT1145 | glucose-inhibited division protein | 2.2 |
|  | BT1181 | glycosyltransferase involved in cell wall biogenesis | 2.3 |
|  | BT1194 | outer membrane protein, OmpA family | 1.3 |
|  | BT1202 | UDP-N-acetylglucosamine 2-epimerase | 1.5 |
|  | BT1203 | putative UDP-N-acetylglucosamine 2-epimerase | 1.5 |
|  | BT1314 | dipeptidyl-peptidase VI | 1.4 |
|  | BT1338 | dTDP-4-dehydrorhamnose 3,5-epimerase | 3.0 |
|  | BT1339 | undecaprenyl-phosphatealpha-N-acetylglucosaminyltransferase | 2.5 |
|  | BT1341 | UDP-glucose 6-dehydrogenase | 3.7 |
|  | BT1342 | putative UDP-glucuronic acid epimerase | 3.6 |
|  | BT1346 | capsule biosynthesis protein capA | 1.9 |
|  | BT1348 | CDP-abequose synthase | 2.2 |
|  | BT1349 | conserved hypothetical protein | 2.1 |
|  | BT1350 | CDP-glucose 4,6-dehydratase | 2.4 |
|  | BT1353 | glycosyltransferase | 1.9 |
|  | BT1356 | putative capsule polysaccharide export protein | 1.8 |
|  | BT1368 | UDP-N-acetylenolpyruvoylglucosamine reductase | 1.6 |
|  | BT1452 | conserved hypothetical protein | 1.8 |
|  | BT1501 | major outer membrane protein OmpA | 1.6 |
|  | BT1502 | hypothetical protein | 2.4 |
|  | BT1646 | putative glycosyltransferase | 4.3 |
|  | BT1649 | conserved hypothetical protein | 1.8 |
|  | BT1650 | putative teichoic acid biosynthesis protein F | 5.2 |
|  | BT1693 | periplasmic linker protein, putative multidrug resistance protein | 2.5 |
|  | BT1695 | outer membrane efflux protein | 2.2 |
|  | BT1740 | hypothetical protein | 1.7 |
|  | BT1880 | tetraacyldisaccharide 4'-kinase | 1.4 |
|  | BT2003 | putative membrane peptidase | 3.9 |
|  | BT2016 | dTDP-glucose 4,6-dehydratase | 1.7 |
|  | BT2017 | glucose-1-phosphate thymidylyltransferase | 1.8 |
|  | BT2038 | cation efflux system protein | 2.3 |
|  | BT2040 | hypothetical protein | 2.1 |
|  | BT2059 | TonB | 1.2 |
|  | BT2133 | conserved hypothetical protein | 1.8 |
|  | BT2687 | cation efflux system protein | 1.8 |
|  | BT2688 | outer membrane efflux protein | 1.5 |
|  | BT2798 | carboxy-terminal processing protease precursor | 1.7 |
|  | BT2917 | hypothetical protein | 1.5 |
|  | BT2936 | putative glycosyltransferase | 2.3 |
|  | BT2938 | putative glycosyltransferase | 1.3 |
|  | BT2944 | putative UDP-N-acetylglucosamine 2-epimerase | 1.9 |
|  | BT2947 | putative glycosyltransferase | 1.3 |
|  | BT3118 | prolipoprotein diacylglyceryl transferase | 2.3 |
|  | BT3187 | penicillin-binding protein, D-alanyl-D-alanine carboxypeptidase | 1.6 |
|  | BT3192 | TonB | 1.8 |
|  | BT3306 | putative sugar isomerase | 1.7 |
|  | BT3336 | UDP-N-acetylglucosamine acetyltransferase | 1.4 |
|  | BT3337 | outer membrane protein oprM precursor, multidrug resistance protein | 2.3 |
|  | BT3339 | AcrA/AcrE family multidrug resistance protein | 1.3 |
|  | BT3450 | UDP-N-acetylmuramoylalanine--D-glutamate ligase | 2.2 |
|  | BT3451 | phospho-N-acetylmuramoyl-pentapeptide-transferase | 2.3 |
|  | BT3452 | UDP-N-acetylmuramoylalanyl-D-glutamate--2, 6-diaminopimelate ligase | 2.6 |
|  | BT3453 | penicillin-binding protein | 1.2 |
|  | BT3455 | S-adenosyl-methyltransferase mraW | 1.5 |
|  | BT3577 | conserved hypothetical protein | 2.0 |
|  | BT3622 | putative glycosyltransferase | 1.8 |
|  | BT3713 | D-alanine--D-alanine ligase | 1.6 |
|  | BT3722 | glutamate racemase | 1.5 |
|  | BT3723 | putative outer membrane protein OmpH | 1.9 |
|  | BT3724 | cationic outer membrane protein precursor | 2.0 |
|  | BT3725 | putative outer membrane protein | 2.9 |
|  | BT3807 | conserved hypothetical protein | 1.6 |
|  | BT3808 | conserved hypothetical protein | 1.9 |
|  | BT3814 | rod shape-determining protein | 1.9 |
|  | BT3904 | conserved hypothetical protein | 1.8 |
|  | BT3905 | putative alkaline protease aprF | 1.7 |
|  | BT3988 | putative peptidoglycan bound protein | 5.1 |
|  | BT3999 | membrane-bound lytic murein transglycosylase D presursor | 2.0 |
|  | BT4004 | lipid-A-disaccharide synthase | 1.6 |
|  | BT4123 | exo-poly-alpha-D-galacturonosidase precursor | 2.2 |
|  | BT4129 | outer membrane assembly protein | 2.2 |
|  | BT4146 | exo-poly-alpha-D-galacturonosidase precursor | 1.4 |
|  | BT4163 | conserved hypothetical protein | 3.8 |
|  | BT4166 | putative lipoprotein | 3.7 |
|  | BT4205 | acyl-[acyl-carrier-protein]--UDP-N- acetylglucosamine O-acyltransferase | 2.9 |
|  | BT4206 | UDP-3-O-[3-hydroxymyristoyl] N-acetylglucosamine deacetylase | 2.0 |
|  | BT4259 | carboxy-terminal processing protease precursor | 2.7 |
|  | BT4304 | putative glycosyltransferase | 1.4 |
|  | BT4321 | 2-dehydro-3-deoxyphosphooctonate aldolase | 1.5 |
|  | BT4487 | putative membrane-bound lytic murein transglycosylase | 2.2 |
|  | BT4564 | putative glycosyltransferase | 1.7 |
|  | BT4576 | hypothetical protein | 1.5 |
|  | BT4703 | chloromuconate cycloisomerase | 2.5 |
|  |  |  |  |
| [Cell motility](http://genome.wustl.edu/GSCGAP/gut/cgi-bin/geneDisplay.cgi?cogId=N&sequencedOrganismId=1) |  |  |  |
|  | BT1026 | hypothetical protein | 1.6 |
|  | BT2213 | hypothetical protein | 1.5 |
|  | BT3027 | conserved hypothetical protein | 3.9 |
|  | BT3129 | putative flagellar motor protein MotB | 1.4 |
|  |  |  |  |
| [Intracellular trafficking, secretion, and vesicular transport](http://genome.wustl.edu/GSCGAP/gut/cgi-bin/geneDisplay.cgi?cogId=U&sequencedOrganismId=1) |  |  |  |
|  | BT1108 | conserved hypothetical protein, putative integral membrane protein | 1.7 |
|  | BT1227 | conserved hypothetical protein | 1.8 |
|  | BT1601 | putative signal recognition protein | 2.0 |
|  | BT1955 | putative cell wall biogenesis protein | 2.6 |
|  | BT2052 | hypothetical protein | 1.6 |
|  | BT2055 | putative biopolymer transport protein | 1.6 |
|  | BT2270 | integral membrane protein (MarC family) | 1.2 |
|  | BT2707 | preprotein translocase SecY subunit | 2.7 |
|  | BT3319 | signal peptidase I | 1.9 |
|  | BT3329 | hypothetical protein | 3.5 |
|  | BT3743 | hypothetical protein | 4.6 |
|  | BT3919 | putative biopolymer transport protein | 2.0 |
|  | BT3920 | conserved hypothetical protein | 1.5 |
|  | BT4325 | hypothetical protein | 1.3 |
|  | BT4362 | preprotein translocase SecA subunit | 1.4 |
|  | BT4504 | conserved hypothetical protein | 3.3 |
|  |  |  |  |
| [Posttranslational modification, protein turnover, chaperones](http://genome.wustl.edu/GSCGAP/gut/cgi-bin/geneDisplay.cgi?cogId=O&sequencedOrganismId=1) |  |  |  |
|  | BT0149 | peptidase | 2.5 |
|  | BT0237 | conserved hypothetical protein | 2.8 |
|  | BT0897 | chaperone protein htpG (heat shock protein htpG) | 2.1 |
|  | BT0898 | ATP-dependent Clp protease | 1.2 |
|  | BT0920 | putative O-sialoglycoprotein endopeptidase | 4.5 |
|  | BT1104 | small heat shock protein | 1.5 |
|  | BT1199 | putative protease | 1.6 |
|  | BT1329 | putative thiol peroxidase | 1.3 |
|  | BT1830 | 10 kDa chaperonin (groES) | 1.7 |
|  | BT1999 | anaerobic ribonucleoside-triphosphate reductase activating protein | 3.6 |
|  | BT2406 | putative DNA repair protein | 1.7 |
|  | BT2412 | putative collagenase | 1.7 |
|  | BT2499 | peptide methionine sulfoxide reductase msrA/msrB | 1.8 |
|  | BT2564 | conserved hypothetical protein | 1.5 |
|  | BT2812 | alkyl hydroperoxide reductase C22 protein | 1.6 |
|  | BT2977 | peptidylprolyl isomerase | 2.7 |
|  | BT2979 | conserved hypothetical protein, putative membrane protein | 2.3 |
|  | BT3612 | putative FKBP-type peptidyl-prolyl cis-trans isomerase fkpA | 2.1 |
|  | BT3802 | peptidyl-prolyl cis-trans isomerase, cyclophilin-type | 1.8 |
|  | BT3810 | putative endothelin-converting enzyme | 1.6 |
|  | BT3811 | putative endothelin-converting enzyme | 1.6 |
|  | BT3842 | ATP-dependent Clp protease proteolytic subunit 2 | 2.3 |
|  | BT3843 | ATP-dependent Clp protease ATP-binding subunit | 2.5 |
|  | BT3848 | peptidyl-prolyl cis-trans isomerase | 1.5 |
|  | BT3889 | subtilisin-like serine proteases | 1.5 |
|  | BT4007 | AAA-metalloprotease FtsH, with ATPase domain | 1.3 |
|  | BT4336 | thioredoxin reductase | 1.3 |
|  | BT4371 | peptidyl-prolyl cis-trans isomerase | 2.2 |
|  | BT4454 | conserved hypothetical protein | 1.4 |
|  | BT4611 | putative bacterioferritin co-migratory protein | 3.1 |
|  | BT4614 | conserved hypothetical protein | 1.4 |
|  | BT4615 | chaperone protein dnaK | 1.6 |
|  |  |  |  |
| [Energy production and conversion](http://genome.wustl.edu/GSCGAP/gut/cgi-bin/geneDisplay.cgi?cogId=C&sequencedOrganismId=1) |  |  |  |
|  | BT0125 | NADH:ubiquinone oxidoreductase subunit | 1.6 |
|  | BT0158 | Na(+)/H(+) antiporter | 1.6 |
|  | BT0174 | conserved hypothetical protein | 2.7 |
|  | BT0216 | putative rubrerythrin | 1.7 |
|  | BT0329 | ketoisovalerate oxidoreductase subunit vorA | 2.8 |
|  | BT0330 | ketoisovalerate oxidoreductase subunit vorA | 3.0 |
|  | BT0332 | 2-oxoisovalerate oxidoreductase beta subunit | 1.4 |
|  | BT0333 | conserved hypothetical protein | 2.0 |
|  | BT0429 | indolepyruvate oxidoreductase subunit iorB | 2.2 |
|  | BT0430 | indolepyruvate oxidoreductase subunit iorA | 2.2 |
|  | BT0535 | iron-containing alcohol dehydrogenase | 1.6 |
|  | BT0617 | Na+-transporting NADH:ubiquinone oxidoreductase, Electron transport complex protein rnfB | 3.4 |
|  | BT0618 | Na+-transporting NADH:ubiquinone oxidoreductase, Electron transport complex protein rnfC | 2.2 |
|  | BT0619 | Na+-transporting NADH:ubiquinone oxidoreductase, Electron transport complex protein rnfD | 2.4 |
|  | BT0621 | Na+-transporting NADH:ubiquinone oxidoreductase, Electron transport complex protein rnfE | 2.8 |
|  | BT0622 | Na+-transporting NADH:ubiquinone oxidoreductase, Electron transport complex protein rnfA | 1.6 |
|  | BT0687 | prismane protein | 2.1 |
|  | BT0714 | ATP synthase A subunit | 1.6 |
|  | BT0716 | ATP synthase B subunit | 1.8 |
|  | BT0718 | ATP synthase alpha chain | 2.1 |
|  | BT0719 | ATP synthase gamma chain | 1.8 |
|  | BT1115 | aldo/keto reductase | 2.1 |
|  | BT1155 | Na+-translocating NADH-quinone reductase subunit | 1.8 |
|  | BT1156 | Na+-translocating NADH-quinone reductase subunit | 3.5 |
|  | BT1157 | Na+-translocating NADH-quinone reductase subunit | 3.8 |
|  | BT1158 | Na+-translocating NADH-quinone reductase subunit | 5.5 |
|  | BT1159 | Na+-translocating NADH-quinone reductase subunit | 3.7 |
|  | BT1196 | pyruvate carboxylase subunit B) | 1.4 |
|  | BT1209 | cytochrome D ubiquinol oxidase subunit II | 2.4 |
|  | BT1210 | cytochrome D ubiquinol oxidase subunit I | 1.9 |
|  | BT1295 | V-type sodium ATP synthase subunit K | 3.3 |
|  | BT1296 | V-type ATP synthase subunit I | 1.7 |
|  | BT1297 | V-type ATP synthase subunit D | 3.4 |
|  | BT1299 | V-type ATP synthase subunit A | 2.1 |
|  | BT1301 | ATP synthase subunit E | 2.4 |
|  | BT1316 | putative NADH dehydrogenase/NAD(P)H nitroreductase | 1.2 |
|  | BT1418 | quinol oxidase | 2.3 |
|  | BT1451 | hypothetical protein | 1.4 |
|  | BT1575 | putative dehydrogenase | 2.0 |
|  | BT1680 | putative NADH dehydrogenase/NAD(P)H nitroreductase | 1.3 |
|  | BT1689 | oxaloacetate decarboxylase beta chain | 4.2 |
|  | BT1696 | oxaloacetate decarboxylase beta chain | 4.4 |
|  | BT1697 | pyruvate carboxylase subunit B | 5.8 |
|  | BT1698 | conserved hypothetical protein | 3.4 |
|  | BT1747 | pyruvate-flavodoxin oxidoreductase | 3.3 |
|  | BT1805 | electron transfer flavoprotein alpha-subunit | 2.8 |
|  | BT1969 | NADP-dependent malate dehydrogenase | 1.7 |
|  | BT2067 | putative NADH:flavin oxidoreductase | 1.2 |
|  | BT2070 | citrate synthase | 1.5 |
|  | BT2123 | glycerol-3-phosphate dehydrogenase | 1.5 |
|  | BT2144 | putative oxidoreductase | 1.4 |
|  | BT2256 | fumarate hydratase class I, anaerobic | 1.5 |
|  | BT2510 | malate dehydrogenase | 1.4 |
|  | BT2790 | phosphoenolpyruvate carboxykinase [ATP] | 1.6 |
|  | BT2837 | 2-oxoglutarate synthase subunit korB | 1.8 |
|  | BT2918 | hypothetical protein | 1.7 |
|  | BT2924 | acetyl-CoA synthetase | 1.2 |
|  | BT3054 | succinate dehydrogenase flavoprotein subunit | 2.1 |
|  | BT3055 | succinate dehydrogenase iron-sulfur protein | 2.5 |
|  | BT3182 | rubrerythrin | 1.5 |
|  | BT3186 | dihydrolipoamide dehydrogenase | 1.6 |
|  | BT3614 | putative oxidoreductase | 1.9 |
|  | BT3692 | phosphate acetyltransferase | 2.6 |
|  | BT3911 | malate dehydrogenase | 2.1 |
|  | BT4061 | NADH dehydrogenase I, chain K | 3.4 |
|  | BT4063 | NADH dehydrogenase I, chain I | 2.2 |
|  | BT4066 | NADH dehydrogenase I, chain B | 2.2 |
|  | BT4126 | flavoprotein | 1.4 |
|  | BT4455 | oxidoreductase, putative glycolate oxidase | 1.5 |
|  | BT4456 | putative electron transport protein | 1.6 |
|  | BT4738 | formate acetyltransferase | 2.0 |
|  |  |  |  |
| [Carbohydrate transport and metabolism](http://genome.wustl.edu/GSCGAP/gut/cgi-bin/geneDisplay.cgi?cogId=G&sequencedOrganismId=1) |  |  |  |
|  | BT0258 | glucosamine-6-phosphate isomerase | 1.8 |
|  | BT0290 | beta-galactosidase precursor | 1.3 |
|  | BT0347 | transketolase | 1.9 |
|  | BT0350 | xylulose kinase (xylulokinase) | 2.8 |
|  | BT0351 | L-arabinose isomerase | 2.6 |
|  | BT0353 | putative sugar epimerase/aldolase | 1.5 |
|  | BT0368 | alpha-L-arabinofuranosidase A precursor | 1.6 |
|  | BT0369 | endo-1,4-beta-xylanase D precursor | 1.6 |
|  | BT0370 | galactokinase | 2.6 |
|  | BT0371 | glucose/galactose transporter | 2.5 |
|  | BT0372 | aldose 1-epimerase precursor | 2.5 |
|  | BT0373 | mannose-6-phosphate isomerase | 2.0 |
|  | BT0456 | beta-hexosaminidase precursor | 1.9 |
|  | BT0459 | beta-hexosaminidase precursor | 1.7 |
|  | BT0460 | beta-hexosaminidase precursor | 3.1 |
|  | BT0461 | beta-galactosidase | 3.3 |
|  | BT0488 | 2-dehydro-3-deoxygluconokinase | 2.1 |
|  | BT0644 | pyruvate phosphate dikinase | 2.0 |
|  | BT0757 | beta-galactosidase | 1.5 |
|  | BT0770 | conserved hypothetical protein | 2.4 |
|  | BT0771 | 1,4-alpha-glucan branching enzyme (isoamylase or pullulanase type II) | 2.3 |
|  | BT0773 | alpha-amylase 3 | 1.6 |
|  | BT0786 | putative integral membrane protein | 1.4 |
|  | BT0793 | xylose isomerase | 4.9 |
|  | BT0794 | D-xylose-proton symporter (D-xylose transporter) | 2.0 |
|  | BT0805 | conserved hypothetical protein | 1.8 |
|  | BT0959 | conserved hypothetical protein | 1.4 |
|  | BT1020 | conserved hypothetical protein | 1.4 |
|  | BT1032 | conserved hypothetical protein | 2.0 |
|  | BT1033 | conserved hypothetical protein | 1.5 |
|  | BT1038 | hypothetical protein | 2.5 |
|  | BT1098 | methylglyoxal synthase | 2.8 |
|  | BT1102 | 6-phosphofructokinase | 2.1 |
|  | BT1105 | phosphoglycerate mutase 1 | 2.0 |
|  | BT1106 | fructose-bisphosphate aldolase class I | 1.5 |
|  | BT1220 | 6-phosphogluconolactonase | 1.4 |
|  | BT1228 | fructose-1,6-bisphosphatase | 2.1 |
|  | BT1274 | L-fuculose-1-phosphate aldolase | 3.1 |
|  | BT1275 | L-fuculose kinase | 2.3 |
|  | BT1277 | L-fucose permease | 5.4 |
|  | BT1293 | alpha-glucan phosphorylase | 3.1 |
|  | BT1343 | putative capsule biosynthesis protein | 1.7 |
|  | BT1432 | mannonate dehydratase | 1.6 |
|  | BT1548 | phosphoglucomutase phosphomannomutase | 1.5 |
|  | BT1660 | phosphoglycerate mutase | 1.7 |
|  | BT1670 | putative transmembrane transport protein | 2.0 |
|  | BT1672 | phosphoglycerate kinase | 2.0 |
|  | BT1691 | fructose-bisphosphate aldolase | 1.7 |
|  | BT1760 | glycosylhydrolase | 7.8 |
|  | BT1769 | conserved hypothetical protein | 1.7 |
|  | BT1842 | alpha-L-fucosidase precursor | 2.3 |
|  | BT1883 | putative alpha-1,6-mannanase | 1.7 |
|  | BT1972 | phosphoenolpyruvate synthase/pyruvate phosphate dikinase | 1.3 |
|  | BT1979 | Meso-diaminopimelate D-dehydrogenase | 1.8 |
|  | BT1986 | putative ribose 5-phosphate isomerase | 1.5 |
|  | BT2000 | putative transport protein | 1.4 |
|  | BT2124 | glucose-6-phosphate isomerase | 2.3 |
|  | BT2150 | methylglyoxal synthase | 1.3 |
|  | BT2215 | hypothetical protein | 1.8 |
|  | BT2439 | beta-N-acetylglucosaminidase | 1.5 |
|  | BT2568 | putative sugar transporter | 1.6 |
|  | BT2631 | putative hydrolase | 1.3 |
|  | BT2662 | alpha-galactosidase precursor | 2.2 |
|  | BT2796 | conserved hypothetical protein | 2.0 |
|  | BT2801 | inositol-1-monophosphatase | 1.5 |
|  | BT2804 | ribokinase | 3.6 |
|  | BT2824 | beta-glucanase precursor | 6.1 |
|  | BT2825 | chitinase | 1.9 |
|  | BT2892 | conserved hypothetical protein | 1.9 |
|  | BT2895 | endo-1,4-beta-xylanase | 2.3 |
|  | BT2898 | endo-1,4-beta-xylanase D precursor | 2.1 |
|  | BT3085 | alpha-xylosidase | 1.5 |
|  | BT3173 | conserved hypothetical protein | 1.6 |
|  | BT3293 | beta-galactosidase | 1.7 |
|  | BT3305 | fructokinase | 1.5 |
|  | BT3387 | putative polysaccharide deacetylase | 1.9 |
|  | BT3419 | 2,3-bisphosphoglycerate-independent phosphoglycerate mutase | 3.0 |
|  | BT3527 | alpha-1,2-mannosidase | 1.2 |
|  | BT3529 | aldose 1-epimerase precursor | 1.9 |
|  | BT3532 | aldose 1-epimerase precursor | 1.4 |
|  | BT3587 | glucosamine-6-phosphate isomerase | 2.4 |
|  | BT3606 | sugar-proton symporter | 1.3 |
|  | BT3618 | conserved hypothetical protein | 1.5 |
|  | BT3655 | arabinosidase | 2.6 |
|  | BT3657 | alpha-L-arabinofuranosidase A precursor | 2.4 |
|  | BT3659 | alpha-xylosidase | 1.7 |
|  | BT3663 | putative beta-xylosidase | 1.4 |
|  | BT3673 | TonB | 1.3 |
|  | BT3698 | alpha-amylase, susG | 2.5 |
|  | BT3763 | rhamnulose kinase/L-fuculose kinase | 1.9 |
|  | BT3765 | L-rhamnose/H+ symporter | 2.3 |
|  | BT3766 | rhamnulose-1-phosphate aldolase | 2.6 |
|  | BT3774 | alpha-mannosidase | 1.5 |
|  | BT3792 | putative alpha-1,6-mannanase | 4.9 |
|  | BT3917 | putative inorganic polyphosphate/ATP-NAD kinase | 1.1 |
|  | BT3929 | triosephosphate isomerase | 2.3 |
|  | BT3990 | putative alpha-1,2-mannosidase | 1.9 |
|  | BT3994 | putative alpha-1,2-mannosidase | 1.5 |
|  | BT4050 | beta-galactosidase | 1.6 |
|  | BT4092 | putative alpha-1,2-mannosidase | 1.5 |
|  | BT4106 | 4-deoxy-L-threo-5-hexosulose-uronate ketol-isomerase | 1.5 |
|  | BT4107 | putative phosphotransferase enzyme II, C component | 1.8 |
|  | BT4127 | glucosamine-6-phosphate isomerase | 1.6 |
|  | BT4154 | chitin deacetylase | 2.2 |
|  | BT4241 | beta-galactosidase | 4.1 |
|  | BT4263 | glyceraldehyde 3-phosphate dehydrogenase | 1.9 |
|  | BT4305 | putative alpha-amylase | 1.4 |
|  | BT4311 | glucose/galactose transporter | 1.8 |
|  | BT4337 | beta-hexosaminidase precursor | 1.3 |
|  | BT4379 | putative oxalate:formate antiporter | 1.2 |
|  | BT4406 | hypothetical protein | 1.7 |
|  | BT4446 | sugar phosphate isomerase/epimerase | 1.8 |
|  | BT4572 | enolase | 1.5 |
|  | BT4667 | beta-galactosidase | 1.3 |
|  | BT4668 | arabinogalactan endo-1,4-beta-galactosidase | 1.6 |
|  | BT4681 | beta-hexosaminidase precursor | 3.9 |
|  | BT4682 | conserved hypothetical protein | 2.5 |
|  | BT4713 | conserved hypothetical protein | 2.1 |
|  |  |  |  |
| [Amino acid transport and metabolism](http://genome.wustl.edu/GSCGAP/gut/cgi-bin/geneDisplay.cgi?cogId=E&sequencedOrganismId=1) |  |  |  |
|  | BT0200 | ATP phosphoribosyltransferase | 1.3 |
|  | BT0249 | 5-methyltetrahydrofolate--homocysteine methyltransferase or methionine synthase | 1.7 |
|  | BT0526 | L-asparaginase I | 1.9 |
|  | BT0556 | carbamoyl phosphate synthetase III (glutamine-hydrolyzing) | 2.0 |
|  | BT0557 | carbamyl phosphate synthetase | 2.1 |
|  | BT0569 | conserved hypothetical protein | 1.5 |
|  | BT0580 | putative H+/peptide symporter | 2.0 |
|  | BT0735 | aspartate decarboxylase AsdA | 2.0 |
|  | BT0785 | glutamine synthetase I | 1.6 |
|  | BT1099 | putative arginase | 1.3 |
|  | BT1152 | D-3-phosphoglycerate dehydrogenase | 2.9 |
|  | BT1161 | putative aminopeptidase C | 1.6 |
|  | BT1260 | putative peptidase | 1.2 |
|  | BT1290 | putrescine transport system permease protein potH | 1.7 |
|  | BT1374 | diaminopimelate decarboxylase | 3.1 |
|  | BT1416 | conserved hypothetical protein | 2.1 |
|  | BT1478 | putative histidinol-phosphatase | 1.9 |
|  | BT1492 | tyrosine phenol-lyase | 1.5 |
|  | BT1549 | putative secreted peptidase | 1.7 |
|  | BT1580 | lactoylglutathione lyase | 1.7 |
|  | BT1685 | lactoylglutathione lyase and related protein | 3.0 |
|  | BT1789 | aminopeptidase C (bleomycin hydrolase) | 1.4 |
|  | BT1815 | low-specificity L-threonine aldolase | 1.6 |
|  | BT1923 | O-acetylhomoserine (thiol)-lyase | 2.5 |
|  | BT1970 | glutamate dehydrogenase | 2.6 |
|  | BT1974 | metallopeptidase family M24, putative Xaa-Pro dipeptidase | 1.4 |
|  | BT2074 | ketol-acid reductoisomerase | 3.1 |
|  | BT2129 | aspartate--ammonia ligase | 2.3 |
|  | BT2415 | aspartate aminotransferase | 2.7 |
|  | BT2519 | putative glycine cleavage system H protein | 2.0 |
|  | BT2570 | glutamate decarboxylase | 2.1 |
|  | BT2573 | glutamate/gamma-aminobutyrate anti-porter | 2.4 |
|  | BT2682 | putative periplasmic protein | 1.5 |
|  | BT2743 | putative aminopeptidase | 1.5 |
|  | BT2757 | L-asparaginase II precursor | 2.6 |
|  | BT2834 | peptidyl-dipeptidase | 1.5 |
|  | BT3029 | Na+/glucose symporter | 2.3 |
|  | BT3320 | dihydrodipicolinate reductase | 2.3 |
|  | BT3375 | aspartate aminotransferase | 1.6 |
|  | BT3394 | putative arginine decarboxylase | 1.6 |
|  | BT3623 | sodium/iodide co-transporter | 1.6 |
|  | BT3636 | aspartate-semialdehyde dehydrogenase | 2.6 |
|  | BT3681 | conserved hypothetical protein, putative outer membrane protein | 4.9 |
|  | BT3733 | argininosuccinate lyase | 2.2 |
|  | BT3866 | glutamine-dependent carbamyl phosphate synthetase | 2.0 |
|  | BT3892 | branched-chain amino acid aminotransferase | 1.5 |
|  | BT3934 | putative phospho-2-dehydro-3-deoxyheptonate aldolase/chorismate mutase | 1.6 |
|  | BT3961 | hypothetical protein | 1.8 |
|  | BT3975 | 3-dehydroquinate synthase | 1.7 |
|  | BT4049 | putative ArgK protein with ATPase and kinase domains | 1.4 |
|  | BT4173 | putative rhamnogalacturonan acetylesterase | 1.6 |
|  | BT4193 | dipeptidyl peptidase IV | 1.3 |
|  | BT4215 | shikimate 5-dehydrogenase | 1.9 |
|  | BT4583 | peptidase T (tripeptide aminopeptidase) | 1.3 |
|  | BT4584 | putative aminomethyltransferase | 1.5 |
|  | BT4612 | conserved hypothetical protein | 1.7 |
|  | BT4678 | L-serine dehydratase | 1.7 |
|  |  |  |  |
| [Nucleotide transport and metabolism](http://genome.wustl.edu/GSCGAP/gut/cgi-bin/geneDisplay.cgi?cogId=F&sequencedOrganismId=1) |  |  |  |
|  | BT0185 | conserved hypothetical protein | 1.2 |
|  | BT0518 | Bifunctional purine biosynthesis protein, putative 5-aminoimidazole-4-carboxamide ribonucleotide(AICAR) transformylase/IMP cyclohydrolase | 1.6 |
|  | BT0704 | phosphoribosylglycinamide formyltransferase 2 | 1.6 |
|  | BT0741 | aspartate carbamoyltransferase regulatory chain | 1.7 |
|  | BT0748 | ribose-phosphate pyrophosphokinase | 2.3 |
|  | BT0888 | AMP nucleosidase | 1.2 |
|  | BT0892 | dihydroorotate dehydrogenase | 2.1 |
|  | BT1084 | hypothetical protein | 1.4 |
|  | BT1333 | putative dihydropyrimidine dehydrogenase [NADP+] precursor | 1.9 |
|  | BT1539 | cytidine deaminase | 1.3 |
|  | BT1843 | adenylosuccinate synthetase | 1.9 |
|  | BT1881 | purine nucleoside phosphorylase I | 1.9 |
|  | BT1998 | anaerobic ribonucleoside-triphosphate reductase | 4.7 |
|  | BT2009 | guanylate kinase (GMP kinase) | 2.2 |
|  | BT2060 | cytidylate kinase | 1.3 |
|  | BT2093 | hypothetical protein | 1.4 |
|  | BT2145 | ribonucleoside-diphosphate reductase alpha chain | 3.0 |
|  | BT2242 | uridylate kinase | 1.8 |
|  | BT2419 | putative GMP synthase [glutamine-hydrolyzing] | 1.7 |
|  | BT2440 | 5'-nucleotidase precursor | 1.4 |
|  | BT2441 | putative 5'-nucleotidase/2',3'-cyclic phosphodiesterase | 1.1 |
|  | BT2518 | phosphoribosylaminoimidazole carboxylase | 2.2 |
|  | BT2527 | uridine kinase (uridine monophosphokinase) | 1.8 |
|  | BT2566 | HIT family protein | 1.2 |
|  | BT2791 | uracil phosphoribosyltransferase | 2.3 |
|  | BT2808 | putative inosine-uridine preferring nucleoside hydrolase | 4.1 |
|  | BT2815 | putative mutT family protein | 1.7 |
|  | BT3128 | putative xanthosine triphosphate pyrophosphatase | 1.7 |
|  | BT3263 | putative deoxyribose-phosphate aldolase | 2.5 |
|  | BT3461 | deoxyuridine 5'-triphosphate nucleotidohydrolase | 1.9 |
|  | BT3563 | putative endonuclease precursor | 2.2 |
|  | BT3871 | adenylosuccinate lyase | 1.5 |
|  | BT3925 | nucleoside diphosphate kinase | 1.7 |
|  | BT4209 | orotidine 5'-phosphate decarboxylase | 2.6 |
|  | BT4217 | phosphoribosylamidoimidazole-succinocarboxamidesynthase | 2.5 |
|  | BT4386 | hypoxanthine-guanine phosphoribosyltransferase | 1.2 |
|  | BT4387 | adenylate kinase (ATP-AMP transphosphatase) | 2.3 |
|  | BT4451 | putative MTA/SAH nucleosidase | 1.6 |
|  | BT4554 | purine nucleoside phosphorylase II | 1.8 |
|  | BT4582 | amidophosphoribosyltransferase precursor | 2.2 |
|  |  |  |  |
| [Coenzyme transport and metabolism](http://genome.wustl.edu/GSCGAP/gut/cgi-bin/geneDisplay.cgi?cogId=H&sequencedOrganismId=1) |  |  |  |
|  | BT0205 | glutamine-dependent NAD+ synthetase | 1.3 |
|  | BT0310 | lipoate-protein ligase A | 1.3 |
|  | BT0428 | phenylacetate-coenzyme A ligase | 2.3 |
|  | BT0575 | conserved hypothetical protein | 2.4 |
|  | BT1153 | phosphoserine aminotransferase | 2.2 |
|  | BT1317 | riboflavin synthase alpha chain | 1.3 |
|  | BT1362 | flavoprotein | 2.1 |
|  | BT1371 | 2-amino-3-ketobutyrate coenzyme A ligase | 1.5 |
|  | BT1442 | adenosylmethionine-8-amino-7-oxononanoate aminotransferase | 1.9 |
|  | BT1560 | nicotinate-nucleotide pyrophosphorylase | 1.3 |
|  | BT1763 | SusC/D paralog | 4.3 |
|  | BT1882 | putative thiamine-monophosphate kinase | 1.6 |
|  | BT1953 | putative TonB-linked outer membrane receptor | 2.2 |
|  | BT2048 | dihydrofolate reductase | 1.8 |
|  | BT2149 | dihydroneopterin aldolase | 1.3 |
|  | BT2817 | putative TonB-dependent receptor | 1.7 |
|  | BT2905 | SusC/D paralog | 1.5 |
|  | BT3034 | phosphopantetheine adenylyltransferase | 1.8 |
|  | BT3117 | putative oxidoreductase | 2.3 |
|  | BT3184 | L-aspartate oxidase | 1.1 |
|  | BT3226 | hypothetical protein | 1.7 |
|  | BT3240 | SusC/D paralog | 1.9 |
|  | BT3646 | dihydropteroate synthase | 1.5 |
|  | BT3680 | SusC/D paralog | 3.9 |
|  | BT3728 | riboflavin biosynthesis protein ribD | 1.7 |
|  | BT3852 | major outer membrane protein OmpA | 1.4 |
|  | BT3918 | pyridoxal phosphate biosynthetic protein pdxJ | 1.4 |
|  | BT4147 | hypothetical protein | 2.1 |
|  | BT4192 | lipoic acid synthetase | 1.2 |
|  | BT4216 | ubiquinone/menaquinone biosynthesis methyltransferase | 1.9 |
|  | BT4298 | SusC/D paralog | 4.8 |
|  | BT4374 | 4-hydroxythreonine-4-phosphate dehydrogenase | 1.7 |
|  | BT4453 | 6-pyruvoyl-tetrahydropterin synthase | 1.3 |
|  | BT4560 | thiamine biosynthesis lipoprotein apbE precursor | 2.4 |
|  | BT4594 | putative dephospho-CoA kinase | 1.6 |
|  | BT4701 | 2-oxoglutarate decarboxylase and 2-succinyl-6-hydroxy-2,4-cyclohexadiene-1-carboxylate synthase | 1.8 |
|  |  |  |  |
| [Lipid transport and metabolism](http://genome.wustl.edu/GSCGAP/gut/cgi-bin/geneDisplay.cgi?cogId=I&sequencedOrganismId=1) |  |  |  |
|  | BT1192 | putative xylanase | 1.8 |
|  | BT1525 | phosphatidylglycerophosphatase A | 1.7 |
|  | BT1526 | myo-inositol-1-phosphate synthase | 2.9 |
|  | BT1546 | acyltransferase | 1.7 |
|  | BT1686 | propionyl-CoA carboxylase beta chain | 3.7 |
|  | BT1688 | biotin carboxyl carrier protein (BCCP) | 4.0 |
|  | BT1806 | acyl-CoA dehydrogenase | 2.4 |
|  | BT1854 | putative membrane-associated phospholipid phosphatase | 2.2 |
|  | BT1917 | propionyl-CoA carboxylase beta chain | 2.2 |
|  | BT2091 | methylmalonyl-CoA mutase small subunit | 1.5 |
|  | BT2154 | putative long-chain-fatty-acid--CoA ligase | 1.8 |
|  | BT2232 | CDP-diacylglycerol--serineO-phosphatidyltransferase | 2.0 |
|  | BT2517 | GcpE, 1-hydroxy-2-methyl-2-(E)-butenyl 4-diphosphate synthase | 2.2 |
|  | BT2520 | conserved hypothetical protein | 1.7 |
|  | BT3084 | lipase, putative | 1.3 |
|  | BT3358 | 3-oxoacyl-[acyl-carrier-protein] synthase II | 2.4 |
|  | BT3550 | putative long-chain-fatty-acid--CoA ligase | 1.4 |
|  | BT3726 | undecaprenyl pyrophosphate synthetase | 1.5 |
|  | BT3834 | 3-oxoacyl-[acyl-carrier-protein] synthase III | 1.8 |
|  | BT3884 | 2-C-methyl-D-erythritol 2,4-cyclodiphosphate synthase | 1.7 |
|  | BT4239 | hypothetical protein | 1.3 |
|  | BT4322 | conserved hypothetical protein, with a diacylglycerol kinase catalytic domain | 1.6 |
|  | BT4704 | O-succinylbenzoic acid--CoA ligase | 6.3 |
|  |  |  |  |
| [Inorganic ion transport and metabolism](http://genome.wustl.edu/GSCGAP/gut/cgi-bin/geneDisplay.cgi?cogId=P&sequencedOrganismId=1) |  |  |  |
|  | BT0439 | SusC/D paralog | 1.7 |
|  | BT0499 | cation efflux system protein | 1.9 |
|  | BT0539 | putative Na+/sulphate transporter | 2.2 |
|  | BT0633 | putative Na+/H+ exchange protein | 1.3 |
|  | BT0634 | Na+/H+ antiporter 1 | 2.1 |
|  | BT0655 | superoxide dismutase [Fe] | 1.5 |
|  | BT1025 | SusC/D paralog | 1.5 |
|  | BT1040 | SusC/D paralog | 2.5 |
|  | BT1042 | SusC/D paralog | 1.9 |
|  | BT1101 | putative sodium/proton antiporter | 2.1 |
|  | BT1107 | ferritin A | 1.9 |
|  | BT1109 | ferritin A | 1.5 |
|  | BT1417 | cytochrome C552 precursor | 1.9 |
|  | BT1440 | SusC/D paralog | 1.3 |
|  | BT1592 | putative ferrous iron transport protein | 1.5 |
|  | BT1624 | putative secreted sulfatase | 1.2 |
|  | BT1636 | arylsulfatase precursor | 3.7 |
|  | BT1735 | chromate transport protein | 1.5 |
|  | BT1736 | chromate transport protein | 2.3 |
|  | BT1844 | transcriptional regulator, putative iron uptake regulation protein | 1.2 |
|  | BT1950 | Fe3+ ABC transporter, ATP-binding protein | 4.4 |
|  | BT1951 | Fe3+ ABC transporter, permease protein | 1.3 |
|  | BT1952 | Fe3+ ABC transporter, periplasmic iron-binding protein | 2.5 |
|  | BT2039 | cation efflux system protein, AcrB/AcrD/AcrF family protein | 7.0 |
|  | BT2099 | Fe3+ ABC transporter, permease | 1.8 |
|  | BT2171 | putative anti-sigma factor | 2.6 |
|  | BT2202 | SusC/D paralog | 2.8 |
|  | BT2572 | putative potassium channel subunit | 1.8 |
|  | BT2626 | SusC/D paralog | 2.5 |
|  | BT2805 | SusC/D paralog | 3.9 |
|  | BT2818 | SusC/D paralog | 3.2 |
|  | BT2894 | SusC/D paralog | 2.7 |
|  | BT2920 | SusC/D paralog | 1.4 |
|  | BT3046 | SusC/D paralog | 2.4 |
|  | BT3101 | N-sulphoglucosamine sulphohydrolase precursor | 1.4 |
|  | BT3106 | arylsulfatase (aryl-sulfate sulphohydrolase) | 1.3 |
|  | BT3248 | putative metal ABC transporter, ATP-binding protein | 1.9 |
|  | BT3332 | SusC/D paralog | 1.7 |
|  | BT3349 | putative sulfatase yidJ | 1.6 |
|  | BT3471 | hypothetical protein | 2.5 |
|  | BT3486 | N-acetylgalactosamine-6-sulfatase precursor | 1.3 |
|  | BT3487 | arylsulfatase (aryl-sulfate sulphohydrolase) | 1.2 |
|  | BT3488 | arylsulfatase (aryl-sulfate sulphohydrolase) | 1.3 |
|  | BT3493 | SusC/D paralog | 1.5 |
|  | BT3560 | conserved hypothetical protein | 1.8 |
|  | BT3604 | SusC/D paralog | 1.3 |
|  | BT3702 | SusC, outer membrane protein involved in starch binding | 2.3 |
|  | BT3708 | alkaline phosphatase III precursor | 1.5 |
|  | BT3958 | SusC/D paralog | 1.3 |
|  | BT3983 | SusC/D paralog | 1.8 |
|  | BT4114 | SusC/D paralog | 6.7 |
|  | BT4121 | SusC/D paralog | 2.0 |
|  | BT4168 | SusC/D paralog | 3.7 |
|  | BT4637 | putative inorganic phosphate transporter | 1.8 |
|  | BT4638 | conserved hypothetical protein | 2.5 |
|  | BT4665 | K+ uptake protein | 1.8 |
|  | BT4666 | putative K+ uptake system protein | 1.7 |
|  | BT4671 | SusC/D paralog | 1.5 |
|  | BT4679 | Mg2+/Co2+ transport protein | 1.5 |
|  | BT4683 | arylsulfatase | 1.6 |
|  | BT4707 | SusC/D paralog | 2.3 |
|  |  |  |  |
| [Secondary metabolites biosynthesis, transport and catabolism](http://genome.wustl.edu/GSCGAP/gut/cgi-bin/geneDisplay.cgi?cogId=Q&sequencedOrganismId=1) |  |  |  |
|  | BT1433 | putative oxidoreductase | 1.8 |
|  | BT1445 | putative biotin synthesis protein bioC | 1.3 |
|  | BT1582 | putative methyltransferase | 1.4 |
|  | BT2066 | putative oxidoreductase | 1.5 |
|  | BT3104 | hypothetical protein | 2.2 |
|  | BT3771 | 3-oxoacyl-[acyl-carrier protein] reductase | 1.8 |
|  | BT4261 | hypothetical protein | 1.4 |
|  |  |  |  |
| [General function prediction only](http://genome.wustl.edu/GSCGAP/gut/cgi-bin/geneDisplay.cgi?cogId=R&sequencedOrganismId=1) |  |  |  |
|  | BT0152 | acetyl esterase (acetylxylosidase) | 2.2 |
|  | BT0246 | putative integral membrane protein, with calcineurin-like phosphoesterase domain | 1.1 |
|  | BT0355 | Na+/glucose cotransporter | 1.6 |
|  | BT0421 | tetratricopeptide repeat (TPR) family protein | 2.0 |
|  | BT0572 | conserved hypothetical protein | 1.5 |
|  | BT0584 | putative outer membrane protein | 2.3 |
|  | BT0736 | putative transport protein | 1.5 |
|  | BT0740 | flavoredoxin | 1.4 |
|  | BT0763 | conserved hypothetical protein | 1.9 |
|  | BT0766 | conserved hypothetical protein | 2.0 |
|  | BT0804 | putative carbon-nitrogen hydrolase | 1.5 |
|  | BT0875 | beta-ureidopropionase | 1.8 |
|  | BT0900 | putative tetratricopeptide repeat family protein | 1.8 |
|  | BT0905 | BatC, conserved hypothetical protein | 3.2 |
|  | BT0906 | BatB, conserved hypothetical protein | 2.5 |
|  | BT0907 | BatA (Bacteroides aerotolerance operon) | 2.6 |
|  | BT0909 | conserved hypothetical protein | 2.1 |
|  | BT0910 | magnesium chelatase, subunit I | 1.8 |
|  | BT0926 | ABC-type transport, permease protein | 3.3 |
|  | BT1011 | conserved hypothetical protein | 1.8 |
|  | BT1116 | conserved hypothetical protein | 1.6 |
|  | BT1146 | putative metallo-beta-lactamase | 1.3 |
|  | BT1332 | conserved hypothetical protein | 1.7 |
|  | BT1340 | putative lipopolysaccharide biosynthesis glycosyltransferase | 3.7 |
|  | BT1347 | conserved hypothetical protein | 1.8 |
|  | BT1354 | putative flippase | 2.0 |
|  | BT1359 | Tetratricopeptide repeat family protein | 1.7 |
|  | BT1367 | hydrolase, metal-dependent | 1.7 |
|  | BT1382 | conserved hypothetical protein | 1.2 |
|  | BT1410 | putative metallo-beta-lactamase | 1.5 |
|  | BT1471 | conserved hypothetical protein | 1.7 |
|  | BT1479 | ABC transporter ATP-binding protein | 1.3 |
|  | BT1578 | putative metal dependent hydrolase | 1.4 |
|  | BT1634 | conserved hypothetical protein | 2.0 |
|  | BT1666 | hydrolase, haloacid dehalogenase-like hydrolase | 1.6 |
|  | BT1813 | conserved hypothetical protein, putative integral membrane protein | 1.8 |
|  | BT1924 | putative metal-dependent hydrolase | 1.9 |
|  | BT2035 | putative ABC transporter ATP-binding protein | 1.6 |
|  | BT2136 | putative purple acid phosphatase | 3.8 |
|  | BT2207 | putative ATPase/GTPase | 2.1 |
|  | BT2211 | conserved hypothetical protein | 3.1 |
|  | BT2273 | methyltransferase | 1.5 |
|  | BT2278 | conserved hypothetical protein | 1.3 |
|  | BT2385 | conserved hypothetical protein | 1.3 |
|  | BT2405 | conserved hypothetical protein | 2.1 |
|  | BT2434 | pyridine nucleotide-disulphide oxidoreductase | 2.1 |
|  | BT2547 | conserved hypothetical protein | 1.3 |
|  | BT2548 | leucine aminopeptidase precursor | 1.9 |
|  | BT2624 | hypothetical protein | 2.0 |
|  | BT2749 | putative membrane-associated HD superfamily hydrolase | 1.6 |
|  | BT2775 | putative helicase | 2.2 |
|  | BT2800 | putative amidophosphoribosyl-transferase | 1.4 |
|  | BT2810 | conserved hypothetical protein | 1.8 |
|  | BT2816 | TPR-domain containing protein | 2.4 |
|  | BT2832 | rhomboid family protein | 1.8 |
|  | BT2844 | conserved hypothetical protein | 1.9 |
|  | BT3023 | conserved hypothetical protein | 1.5 |
|  | BT3083 | putative ATPase | 1.2 |
|  | BT3224 | putative lysine decarboxylase | 1.6 |
|  | BT3264 | conserved hypothetical protein | 2.4 |
|  | BT3286 | conserved hypothetical protein | 2.0 |
|  | BT3287 | polysaccharide biosynthesis protein | 1.5 |
|  | BT3364 | hypothetical protein | 1.7 |
|  | BT3373 | spermidine n1-acetyltransferase | 2.0 |
|  | BT3405 | conserved hypothetical protein | 1.3 |
|  | BT3432 | conserved hypothetical protein | 1.5 |
|  | BT3459 | hemolysin A | 1.7 |
|  | BT3462 | TPR domain protein | 1.2 |
|  | BT3470 | putative dehydrogenase | 2.2 |
|  | BT3472 | putative hydrogenase | 1.5 |
|  | BT3515 | putatuve glycosylhydrolase | 1.4 |
|  | BT3551 | putative zinc protease | 1.7 |
|  | BT3581 | putative dehydrogenases and relate proteins | 1.5 |
|  | BT3583 | putative dehydrogenases and relate proteins | 1.3 |
|  | BT3585 | putative oxidoreductase | 1.5 |
|  | BT3586 | putative dehydrogenase | 2.0 |
|  | BT3648 | putative modulator of DNA gyrase | 2.2 |
|  | BT3649 | putative modulator of DNA gyrase | 2.6 |
|  | BT3687 | conserved hypothetical protein | 1.9 |
|  | BT3730 | putative regulatory protein | 1.6 |
|  | BT3832 | conserved hypothetical protein | 1.4 |
|  | BT3835 | putative GTP-binding protein | 1.6 |
|  | BT3836 | putative phosphoglycerate dehydrogenase | 1.2 |
|  | BT3837 | ABC transporter ATP-binding protein | 1.3 |
|  | BT3885 | putative phosphohydrolase, Icc family | 1.8 |
|  | BT3922 | putative ThiJ family intracellular protease/amidase | 1.7 |
|  | BT3937 | TPR-domain containing protein | 2.2 |
|  | BT4003 | stationary-phase survival protein SurE | 1.2 |
|  | BT4069 | conserved hypothetical protein, putative regulatory protein | 1.3 |
|  | BT4174 | conserved hypothetical protein | 2.0 |
|  | BT4208 | phosphohydrolase | 2.8 |
|  | BT4214 | conserved hypothetical protein | 1.8 |
|  | BT4243 | putative oxidoreductase (putative secreted protein) | 2.6 |
|  | BT4254 | conserved hypothetical protein | 1.7 |
|  | BT4273 | conserved hypothetical protein | 1.4 |
|  | BT4294 | hypothetical protein | 3.4 |
|  | BT4320 | putative zinc protease | 1.9 |
|  | BT4369 | conserved hypothetical protein | 1.9 |
|  | BT4370 | putative hemolysin | 1.5 |
|  | BT4417 | HD superfamily hydrolase | 1.7 |
|  | BT4463 | phosphatidate cytidylyltransferase | 1.3 |
|  | BT4482 | conserved hypothetical protein | 2.9 |
|  | BT4485 | conserved hypothetical protein | 1.2 |
|  | BT4539 | conserved hypothetical protein | 1.4 |
|  | BT4556 | hypothetical protein | 2.0 |
|  | BT4664 | tryptophan synthase beta chain | 2.2 |
|  | BT4699 | putative hydrolase, haloacid dehalogenase-like hydrolase | 1.9 |
|  |  |  |  |
| [Function unknown](http://genome.wustl.edu/GSCGAP/gut/cgi-bin/geneDisplay.cgi?cogId=S&sequencedOrganismId=1) |  |  |  |
|  | BT0211 | hypothetical protein | 2.0 |
|  | BT0515 | terminal quinol oxidase, subunit, putative (doxD-like) | 1.8 |
|  | BT0567 | putative protein involved in barrier function of the cell envelope | 1.6 |
|  | BT0627 | conserved hypothetical protein, with a conserved domain of unknown function | 3.5 |
|  | BT0776 | conserved hypothetical protein | 1.5 |
|  | BT0882 | conserved hypothetical protein | 1.4 |
|  | BT0924 | conserved hypothetical protein | 4.6 |
|  | BT0991 | conserved hypothetical protein | 1.3 |
|  | BT1086 | putative oligopeptide transporter | 1.6 |
|  | BT1151 | conserved hypothetical protein | 3.5 |
|  | BT1201 | conserved hypothetical protein | 1.5 |
|  | BT1330 | conserved hypothetical protein | 1.3 |
|  | BT1337 | putative aminopeptidase C | 2.1 |
|  | BT1393 | 4-carboxymuconolactone decarboxylase | 1.2 |
|  | BT1408 | putative Tricorn-like protease | 1.4 |
|  | BT1547 | conserved hypothetical protein | 1.2 |
|  | BT1755 | putative exported 24-amino acid repeat protein | 1.6 |
|  | BT1850 | conserved hypothetical protein | 2.9 |
|  | BT1954 | putative surface layer protein | 4.6 |
|  | BT2068 | 3-oxo-5-alpha-steroid 4-dehydrogenase | 1.8 |
|  | BT2182 | conserved hypothetical protein | 1.2 |
|  | BT2505 | conserved hypothetical protein | 1.2 |
|  | BT2896 | conserved hypothetical protein | 3.3 |
|  | BT2916 | conserved hypothetical protein | 1.9 |
|  | BT3030 | conserved hypothetical protein with conserved domains | 1.5 |
|  | BT3047 | hypothetical protein | 3.3 |
|  | BT3215 | conserved hypothetical protein | 1.3 |
|  | BT3242 | hypothetical protein | 2.4 |
|  | BT3288 | putative integral membrane protein | 1.4 |
|  | BT3443 | conserved hypothetical protein with a conserved domain | 2.1 |
|  | BT3679 | conserved hypothetical protein, with a conserved domain | 2.2 |
|  | BT3689 | conserved hypothetical protein | 1.6 |
|  | BT3697 | UDP-2,3-diacylglucosamine hydrolase | 1.4 |
|  | BT3711 | conserved hypothetical protein | 1.2 |
|  | BT3781 | conserved hypothetical protein | 2.1 |
|  | BT3869 | conserved hypothetical protein | 1.3 |
|  | BT3893 | conserved hypothetical protein | 2.0 |
|  | BT4117 | conserved hypothetical protein | 1.2 |
|  | BT4242 | putative transporter | 2.5 |
|  | BT4316 | conserved hypothetical protein | 2.7 |
|  | BT4328 | conserved hypothetical protein | 3.5 |
|  | BT4329 | conserved hypothetical protein with a conserved domain | 1.8 |
|  | BT4389 | conserved hypothetical protein | 1.6 |
|  | BT4459 | conserved hypothetical protein, putative transport protein | 1.3 |
|  | BT4537 | conserved hypothetical protein | 1.2 |
|  | BT4555 | conserved hypothetical protein | 2.3 |
|  | BT4561 | conserved hypothetical protein, putative integral membrane protein | 1.4 |
|  | BT4698 | conserved hypothetical protein | 2.1 |
|  | BT4726 | glycerophosphoryl diester phosphodiesterase | 2.5 |
|  |  |  |  |
| [Not in COG](http://genome.wustl.edu/GSCGAP/gut/cgi-bin/geneDisplay.cgi?cogId=+&sequencedOrganismId=1) |  |  |  |
|  | BT0062 | hypothetical protein | 1.8 |
|  | BT0117 | arsenical resistance operon trans-acting repressor | 3.0 |
|  | BT0127 | putative transmembrane protein | 1.2 |
|  | BT0132 | alpha-glucosidase, putative | 1.2 |
|  | BT0134 | conserved hypothetical protein | 1.8 |
|  | BT0151 | hypothetical protein | 1.5 |
|  | BT0153 | hypothetical protein | 1.8 |
|  | BT0157 | hypothetical protein | 1.2 |
|  | BT0160 | hypothetical protein | 1.7 |
|  | BT0173 | conserved hypothetical protein | 3.3 |
|  | BT0236 | conserved hypothetical protein | 2.0 |
|  | BT0241 | hypothetical protein | 1.1 |
|  | BT0273 | hypothetical protein | 1.2 |
|  | BT0283 | hypothetical protein | 3.7 |
|  | BT0328 | hypothetical protein | 2.4 |
|  | BT0352 | conserved hypothetical protein | 2.6 |
|  | BT0377 | conserved hypothetical protein | 1.3 |
|  | BT0406 | hypothetical protein | 1.3 |
|  | BT0410 | hypothetical protein | 1.9 |
|  | BT0416 | conserved hypothetical protein | 2.1 |
|  | BT0417 | hypothetical protein | 2.4 |
|  | BT0440 | SusC/D paralog | 1.4 |
|  | BT0450 | conserved hypothetical protein | 2.4 |
|  | BT0511 | hypothetical protein | 1.1 |
|  | BT0574 | hypothetical protein | 1.9 |
|  | BT0576 | hypothetical protein | 5.1 |
|  | BT0631 | conserved hypothetical protein | 3.3 |
|  | BT0645 | conserved hypothetical protein | 1.4 |
|  | BT0658 | hypothetical protein | 1.2 |
|  | BT0701 | hypothetical protein | 1.8 |
|  | BT0713 | hypothetical protein | 1.7 |
|  | BT0715 | ATP synthase C subunit | 1.5 |
|  | BT0727 | conserved hypothetical protein | 1.6 |
|  | BT0764 | hypothetical protein | 3.8 |
|  | BT0777 | hypothetical protein | 1.5 |
|  | BT0840 | hypothetical protein | 1.6 |
|  | BT0842 | hypothetical protein | 1.6 |
|  | BT0843 | hypothetical protein | 1.9 |
|  | BT0848 | hypothetical protein | 1.4 |
|  | BT0866 | SusC/D paralog | 2.4 |
|  | BT0904 | BatD, conserved hypothetical protein | 2.7 |
|  | BT1017 | hypothetical protein | 1.7 |
|  | BT1023 | conserved hypothetical protein | 3.0 |
|  | BT1029 | SusC/D paralog | 1.6 |
|  | BT1030 | hypothetical protein | 2.1 |
|  | BT1036 | hypothetical protein | 3.4 |
|  | BT1037 | conserved hypothetical protein | 2.6 |
|  | BT1043 | hypothetical protein | 3.0 |
|  | BT1044 | hypothetical protein | 3.5 |
|  | BT1049 | putative patatin-like protein | 1.8 |
|  | BT1050 | hypothetical protein | 1.8 |
|  | BT1140 | hypothetical protein | 1.4 |
|  | BT1175 | hypothetical protein | 1.2 |
|  | BT1179 | glycosyltransferase, family 8 | 2.1 |
|  | BT1182 | hypothetical protein | 1.5 |
|  | BT1185 | OmpA-related protein | 1.9 |
|  | BT1191 | hypothetical protein | 1.5 |
|  | BT1206 | conserved hypothetical protein | 2.1 |
|  | BT1211 | hypothetical protein | 1.6 |
|  | BT1282 | hypothetical protein | 3.0 |
|  | BT1283 | conserved hypothetical protein | 4.3 |
|  | BT1284 | putative endo-beta-N-acetylglucosaminidase F1 precursor (mannosyl-glycoprotein endo-beta-N-acetyl-glucosaminidase F1) | 2.7 |
|  | BT1300 | hypothetical protein | 2.5 |
|  | BT1331 | hypothetical protein | 1.9 |
|  | BT1369 | hypothetical protein | 1.5 |
|  | BT1372 | hypothetical protein | 2.8 |
|  | BT1414 | hypothetical protein | 2.4 |
|  | BT1439 | SusC/D paralog | 1.8 |
|  | BT1480 | hypothetical protein | 1.4 |
|  | BT1484 | conserved hypothetical protein with a conserved domain | 1.6 |
|  | BT1487 | hypothetical protein | 2.1 |
|  | BT1488 | hypothetical protein | 3.0 |
|  | BT1491 | hypothetical protein | 3.5 |
|  | BT1507 | hypothetical protein | 2.3 |
|  | BT1534 | hypothetical protein | 1.8 |
|  | BT1573 | conserved hypothetical protein | 1.6 |
|  | BT1579 | hypothetical protein | 2.4 |
|  | BT1612 | hypothetical protein | 2.2 |
|  | BT1629 | hypothetical protein | 4.4 |
|  | BT1631 | SusC/D paralog | 1.2 |
|  | BT1687 | hypothetical protein | 4.5 |
|  | BT1700 | conserved hypothetical protein | 1.2 |
|  | BT1731 | hypothetical protein | 2.4 |
|  | BT1741 | hypothetical protein | 1.6 |
|  | BT1762 | SusC/D paralog | 9.7 |
|  | BT1777 | conserved hypothetical protein | 1.5 |
|  | BT1792 | putative cell surface protein | 1.8 |
|  | BT1908 | hypothetical protein | 1.3 |
|  | BT1934 | hypothetical protein | 2.5 |
|  | BT1956 | putative cell surface protein | 2.4 |
|  | BT1957 | hypothetical protein | 1.8 |
|  | BT1976 | hypothetical protein | 1.3 |
|  | BT2053 | hypothetical protein | 1.6 |
|  | BT2054 | hypothetical protein | 1.7 |
|  | BT2081 | hypothetical protein | 1.7 |
|  | BT2082 | hypothetical protein | 1.6 |
|  | BT2170 | hypothetical protein | 2.5 |
|  | BT2208 | hypothetical protein | 1.8 |
|  | BT2218 | hypothetical protein | 1.4 |
|  | BT2254 | putative pectate lyase | 1.9 |
|  | BT2274 | hypothetical protein | 1.2 |
|  | BT2280 | hypothetical protein | 2.5 |
|  | BT2354 | conserved hypothetical protein | 1.4 |
|  | BT2437 | hypothetical protein | 1.4 |
|  | BT2480 | conserved hypothetical protein | 1.8 |
|  | BT2523 | alpha-rhamnosidase | 1.4 |
|  | BT2535 | hypothetical protein | 1.8 |
|  | BT2559 | hypothetical protein | 2.6 |
|  | BT2620 | alpha-glucosidase | 1.3 |
|  | BT2625 | SusC/D paralog | 2.1 |
|  | BT2633 | hypothetical protein | 1.7 |
|  | BT2751 | conserved hypothetical protein | 1.2 |
|  | BT2768 | hypothetical protein | 1.6 |
|  | BT2809 | putative integral membrane protein | 1.8 |
|  | BT2821 | SusC/D paralog | 3.1 |
|  | BT2822 | conserved hypothetical protein | 4.4 |
|  | BT2823 | hypothetical protein | 9.9 |
|  | BT2853 | hypothetical protein | 1.3 |
|  | BT2893 | hypothetical protein | 3.5 |
|  | BT2906 | conserved hypothetical protein | 1.3 |
|  | BT2919 | SusC/D paralog | 1.8 |
|  | BT2935 | hypothetical protein | 2.9 |
|  | BT2958 | conserved hypothetical protein | 1.4 |
|  | BT2981 | hypothetical protein | 1.2 |
|  | BT3004 | conserved hypothetical protein | 1.4 |
|  | BT3006 | hypothetical protein | 1.3 |
|  | BT3032 | conserved hypothetical protein, with a weak acyltransferase domain | 1.3 |
|  | BT3044 | hypothetical protein | 1.6 |
|  | BT3045 | hypothetical protein | 1.7 |
|  | BT3053 | putative cytochrome B subunit | 2.3 |
|  | BT3140 | conserved hypothetical protein | 1.5 |
|  | BT3145 | hypothetical protein | 1.6 |
|  | BT3147 | hypothetical protein | 1.7 |
|  | BT3149 | hypothetical protein | 1.3 |
|  | BT3206 | hypothetical protein | 1.5 |
|  | BT3211 | conserved hypothetical protein | 2.0 |
|  | BT3225 | hypothetical protein | 2.9 |
|  | BT3233 | hypothetical protein | 1.8 |
|  | BT3238 | hypothetical protein | 1.8 |
|  | BT3243 | hypothetical protein | 1.2 |
|  | BT3262 | hypothetical protein | 1.3 |
|  | BT3294 | putative alpha-glucosidase | 1.4 |
|  | BT3323 | hypothetical protein | 1.4 |
|  | BT3342 | hypothetical protein | 1.5 |
|  | BT3345 | conserved hypothetical protein, putative outer membrane protein | 2.5 |
|  | BT3348 | putative unsaturated glucuronyl hydrolase | 2.0 |
|  | BT3363 | lipopolysaccharide core biosynthesis protein LpsA | 1.3 |
|  | BT3382 | hypothetical protein | 2.2 |
|  | BT3383 | hypothetical protein | 1.8 |
|  | BT3389 | hypothetical protein | 1.3 |
|  | BT3433 | hypothetical protein | 1.5 |
|  | BT3434 | hypothetical protein | 1.8 |
|  | BT3446 | cell division protein FtsQ | 1.9 |
|  | BT3454 | hypothetical protein | 1.7 |
|  | BT3469 | conserved hypothetical protein | 3.0 |
|  | BT3476 | conserved hypothetical protein | 1.3 |
|  | BT3477 | glutaminase A | 1.7 |
|  | BT3484 | hypothetical protein | 1.2 |
|  | BT3495 | SusC/D paralog | 5.6 |
|  | BT3559 | conserved hypothetical protein | 1.3 |
|  | BT3561 | hypothetical protein | 1.9 |
|  | BT3562 | conserved hypothetical protein | 1.6 |
|  | BT3568 | SusC/D paralog | 2.1 |
|  | BT3590 | alpha-N-acetylglucosaminidase precursor | 1.5 |
|  | BT3637 | conserved hypothetical protein | 1.9 |
|  | BT3651 | hypothetical protein | 5.7 |
|  | BT3652 | conserved hypothetical protein | 4.3 |
|  | BT3653 | hypothetical protein | 2.5 |
|  | BT3682 | hypothetical protein | 2.4 |
|  | BT3699 | outer membrane protein SusF | 2.9 |
|  | BT3700 | outer membrane protein SusE | 3.3 |
|  | BT3732 | hypothetical protein | 2.9 |
|  | BT3744 | hypothetical protein | 1.3 |
|  | BT3745 | hypothetical protein | 1.5 |
|  | BT3790 | hypothetical protein | 1.9 |
|  | BT3806 | conserved hypothetical protein | 1.4 |
|  | BT3815 | hypothetical protein | 3.6 |
|  | BT3850 | hypothetical protein | 1.6 |
|  | BT3910 | conserved hypothetical protein | 2.5 |
|  | BT3921 | hypothetical protein | 2.2 |
|  | BT3930 | hypothetical protein | 2.4 |
|  | BT3959 | conserved hypothetical protein, putative outer membrane protein | 1.2 |
|  | BT3981 | hypothetical protein | 1.2 |
|  | BT3984 | hypothetical protein | 4.6 |
|  | BT3985 | hypothetical protein | 7.1 |
|  | BT3986 | putative patatin-like protein | 7.2 |
|  | BT3987 | endo-beta-N-acetylglucosaminidase F1 precursor | 5.8 |
|  | BT4000 | hypothetical protein | 1.5 |
|  | BT4038 | SusC/D paralog | 2.0 |
|  | BT4040 | putative galactose oxidase precursor | 2.1 |
|  | BT4048 | hypothetical protein | 1.7 |
|  | BT4082 | hypothetical protein | 1.9 |
|  | BT4083 | hypothetical protein | 1.7 |
|  | BT4113 | SusC/D paralog | 4.1 |
|  | BT4122 | SusC/D paralog | 8.6 |
|  | BT4165 | SusC/D paralog | 4.0 |
|  | BT4167 | hypothetical protein | 2.5 |
|  | BT4169 | hypothetical protein | 3.7 |
|  | BT4170 | putative pectate lyase L precursor | 1.9 |
|  | BT4171 | hypothetical protein | 3.3 |
|  | BT4180 | acetyl xylan esterase A | 1.5 |
|  | BT4189 | conserved hypothetical protein | 1.5 |
|  | BT4204 | conserved hypothetical protein | 1.9 |
|  | BT4240 | conserved hypothetical protein, with a phosphotransferase enzyme family domain | 3.1 |
|  | BT4284 | hypothetical protein | 1.7 |
|  | BT4293 | conserved hypothetical protein | 1.6 |
|  | BT4296 | hypothetical protein | 3.9 |
|  | BT4297 | SusC/D paralog | 2.8 |
|  | BT4319 | hypothetical protein | 1.4 |
|  | BT4327 | hypothetical protein | 2.9 |
|  | BT4333 | hypothetical protein | 1.5 |
|  | BT4351 | hypothetical protein | 1.3 |
|  | BT4368 | hypothetical protein | 1.8 |
|  | BT4373 | hypothetical protein | 1.2 |
|  | BT4395 | hyaluronoglucosaminidase precursor | 2.4 |
|  | BT4500 | hypothetical protein | 1.5 |
|  | BT4508 | hypothetical protein | 1.4 |
|  | BT4532 | hypothetical protein | 2.7 |
|  | BT4575 | hypothetical protein | 2.3 |
|  | BT4590 | conserved hypothetical protein | 2.3 |
|  | BT4593 | conserved hypothetical protein | 4.1 |
|  | BT4600 | conserved hypothetical protein | 1.8 |
|  | BT4632 | putative galactose oxidase precursor | 1.7 |
|  | BT4633 | SusC/D paralog | 1.3 |
|  | BT4708 | hypothetical protein | 4.4 |
|  | BT4709 | glycosyl hydrolase | 2.9 |
|  | BT4711 | conserved hypothetical protein | 2.6 |
|  | BT4712 | hypothetical protein | 2.3 |
