# Supplementary material for: Genomic and Metabolic Studies of the Impact of Probiotics on a Model Gut Symbiont and Host
Source: PLoS Biol. 2006 Nov 28;4(12):e413. doi: 10.1371/journal.pbio.0040413 (PMC1661682; doi:10.1371/journal.pbio.0040413)
Supplement: Supplementary file 16 [file pbio.0040413.st007.doc]

**Table S7.** COG functional group-based categorization of *B. thetaiotaomicron* that are more highly expressed in the ceca of B6 mice compared to NMRI mice irrespective of colonization state.

| **COG function** | **Gene** | **Description** | **Fold difference in expression (B6 vs. NMRI)** |
| --- | --- | --- | --- |
| Translation, ribosomal structure and biogenesis |  |  |  |
|  | BT0005 | 30S ribosomal protein S17 | 2.1 |
|  | BT0155 | putative tRNA (5-methylaminomethyl-2-thiouridylate)-methyltransferase | 3.1 |
|  | BT0232 | putative protein involved in transposition | 1.5 |
|  | BT0239 | hypothetical protein | 1.5 |
|  | BT0314 | ribosomal protein L11 methyltransferase | 1.7 |
|  | BT0420 | peptide deformylase(PDF) | 1.3 |
|  | BT0626 | phenylalanyl-tRNA synthetase beta chain | 1.4 |
|  | BT0780 | acetyltransferase | 1.6 |
|  | BT1079 | acetyltransferase | 1.4 |
|  | BT1454 | putative peptide chain release factor | 2.6 |
|  | BT1692 | 50S ribosomal protein L31 type B | 1.5 |
|  | BT2475 | putative translation initiation inhibitor | 2.2 |
|  | BT2786 | 30S ribosomal protein S15 | 1.6 |
|  | BT3073 | putative TIM-barrel enzyme, possible dehydrogenase, contains a highly conserved dihydrouridine synthase domain | 1.4 |
|  | BT3503 | glutaminase A | 2.0 |
|  | BT3712 | ribosomal large subunit pseudouridine synthase D | 1.4 |
|  | BT3888 | putative tRNA (5-methylaminomethyl-2-thiouridylate) methyltransferase | 1.2 |
|  | BT4203 | tRNA delta(2)-isopentenylpyrophosphate transferase | 1.8 |
|  | BT4237 | tRNA pseudouridine synthase A | 2.1 |
|  | BT4501 | ribosomal large subunit pseudouridine synthase A | 1.5 |
|  | BT4776 | conserved protein found in conjugate transposon | 5.1 |
|  |  |  |  |
| Transcription |  |  |  |
|  | BT0121 | transcriptional regulator, ArsR family | 1.5 |
|  | BT0326 | putative RNA polymerase ECF-type sigma factor | 3.9 |
|  | BT0507 | transcriptional regulator (TetR/AcrR family) | 3.3 |
|  | BT0582 | conserved hypothetical protein | 1.5 |
|  | BT0583 | hypothetical protein | 1.6 |
|  | BT0636 | putative transcriptional regulator | 1.2 |
|  | BT0728 | transcriptional regulator | 2.0 |
|  | BT0978 | ECF-type sigma factor | 1.4 |
|  | BT1078 | putative transcriptional regulator | 1.5 |
|  | BT1130 | hypothetical protein | 2.2 |
|  | BT1133 | hypothetical protein | 2.5 |
|  | BT1187 | transcriptional regulator | 1.8 |
|  | BT1189 | conserved hypothetical protein | 1.3 |
|  | BT1235 | AraC-type transcription regulator | 1.3 |
|  | BT1253 | conserved hypothetical protein | 1.5 |
|  | BT1457 | two-component system response regulator | 8.7 |
|  | BT1559 | RNA polymerase ECF-type sigma factor | 1.1 |
|  | BT1568 | conserved hypothetical protein | 1.7 |
|  | BT1572 | RNA polymerase ECF-type sigma factor | 3.3 |
|  | BT1797 | putative transcriptional regulator | 1.5 |
|  | BT1817 | RNA polymerase ECF-type sigma factor | 1.7 |
|  | BT1884 | cold shock protein, putative DNA-binding protein | 1.7 |
|  | BT1963 | putative transcriptional regulator | 1.2 |
|  | BT2049 | transcriptional regulator | 1.7 |
|  | BT2103 | transcriptional regulator | 1.8 |
|  | BT2164 | MarR family transcriptional regulator | 1.2 |
|  | BT2226 | transcriptional regulator | 1.8 |
|  | BT2320 | transcription regulator | 2.8 |
|  | BT2338 | putative transcriptional regulator | 1.6 |
|  | BT2342 | transcription regulator | 1.6 |
|  | BT2356 | transcriptional regulator, AraC family | 1.6 |
|  | BT2383 | RNA polymerase ECF-type sigma factor | 3.6 |
|  | BT2386 | AsnC family transcriptional regulator | 1.8 |
|  | BT2410 | putative acetyltransferase | 1.8 |
|  | BT2482 | transcriptional regulator | 1.5 |
|  | BT2546 | transcriptional regulator | 1.3 |
|  | BT2651 | conserved hypothetical protein | 4.5 |
|  | BT2886 | putative transcriptional regulator | 2.4 |
|  | BT2889 | AraC family transcriptional regulator | 2.6 |
|  | BT2932 | transcriptional regulator | 1.2 |
|  | BT2957 | transcriptional regulator | 1.4 |
|  | BT2975 | putative nicotinate mononucleotide:5,6-dimethylbenzimidazole phosphoribosyltransferase | 1.9 |
|  | BT3052 | transcriptional regulator | 1.1 |
|  | BT3058 | transcriptional regulator | 1.8 |
|  | BT3353 | Snf2 family helicase | 1.4 |
|  | BT3556 | transcriptional regulator | 1.8 |
|  | BT3600 | ROK family transcriptional repressor | 1.6 |
|  | BT3609 | transcriptional regulator | 1.3 |
|  | BT3762 | arginine repressor, transcriptional regulator of arginine metabolism | 1.5 |
|  | BT3966 | two-component system response regulator | 1.5 |
|  | BT3974 | conserved hypothetical protein | 1.6 |
|  | BT4013 | restriction endonuclease | 2.3 |
|  | BT4035 | hypothetical protein | 2.5 |
|  | BT4198 | conserved hypothetical protein | 1.4 |
|  | BT4201 | conserved hypothetical protein | 3.1 |
|  | BT4232 | hypothetical protein | 1.7 |
|  | BT4250 | RNA polymerase ECF-type sigma factor | 2.3 |
|  | BT4347 | RNA polymerase ECF-type sigma factor | 2.4 |
|  | BT4418 | hypothetical protein | 2.1 |
|  | BT4571 | RNA polymerase ECF-type sigma factor | 4.7 |
|  | BT4654 | ROK family transcriptional repressor, with glucokinase domain | 1.9 |
|  | BT4696 | conserved hypothetical protein | 1.3 |
|  | BT4720 | putative RNA polymerase ECF-type sigma factor | 1.8 |
|  | BT4733 | conserved hypothetical protein | 3.0 |
|  | BT4749 | putative DNA methylase | 1.3 |
|  |  |  |  |
| Replication, recombination and repair |  |  |  |
|  | BT0025 | putative transposase | 1.4 |
|  | BT0027 | putative transposase | 2.0 |
|  | BT0034 | hypothetical protein | 2.4 |
|  | BT0036 | integrase | 1.4 |
|  | BT0076 | transposase | 1.4 |
|  | BT0078 | putative DNA repair protein | 1.6 |
|  | BT0091 | conserved protein found in conjugate transposon | 2.2 |
|  | BT0105 | DNA topoisomerase I (Omega-protein) | 1.3 |
|  | BT0244 | putative exonuclease | 1.7 |
|  | BT0291 | integrase | 1.3 |
|  | BT0419 | putative endonuclease | 1.3 |
|  | BT0795 | conserved hypothetical protein | 1.7 |
|  | BT0953 | integrase | 1.6 |
|  | BT1041 | integrase | 1.8 |
|  | BT1057 | conserved hypothetical protein | 2.3 |
|  | BT1172 | DNA primase/helicase | 1.8 |
|  | BT1188 | putative transcription regulator | 2.3 |
|  | BT1361 | DNA repair protein recN (Recombination protein N) | 2.6 |
|  | BT1424 | hypothetical protein | 1.7 |
|  | BT1427 | tetracycline resistance element mobilization regulatory protein rteC | 2.7 |
|  | BT1431 | putative DNA polymerase III epsilon chain | 1.5 |
|  | BT1498 | A/G-specific adenine glycosylase | 2.5 |
|  | BT1516 | replicative DNA helicase | 1.9 |
|  | BT1517 | conserved hypothetical protein | 2.4 |
|  | BT1518 | hypothetical protein | 4.1 |
|  | BT1544 | NADH pyrophosphatase, MutT family hydrolase | 1.2 |
|  | BT1610 | DNA polymerase III subunit gamma/tau | 2.1 |
|  | BT1793 | integrase protein | 1.2 |
|  | BT1928 | transposase | 3.8 |
|  | BT1929 | transposase | 2.8 |
|  | BT1932 | hypothetical protein | 2.1 |
|  | BT1978 | Holliday junction DNA helicase ruvA | 1.3 |
|  | BT1990 | hypothetical protein | 1.6 |
|  | BT2021 | conserved hypothetical protein, putative non-specific DNA-binding protein | 1.6 |
|  | BT2236 | conserved hypothetical protein | 1.9 |
|  | BT2277 | transposase | 2.3 |
|  | BT2297 | putative reverse transcriptase | 1.9 |
|  | BT2347 | transposase | 2.0 |
|  | BT2349 | transposase | 1.9 |
|  | BT2445 | hypothetical protein | 1.5 |
|  | BT2446 | integrase | 2.5 |
|  | BT2447 | integrase | 2.2 |
|  | BT2456 | putative reverse transcriptase/maturase | 2.0 |
|  | BT2469 | integrase | 1.5 |
|  | BT2488 | integrase | 2.5 |
|  | BT2576 | transposase | 2.9 |
|  | BT2602 | putative maturase/reverse transcriptase | 1.6 |
|  | BT2634 | putative integrase/recombinase | 1.7 |
|  | BT2635 | putative integrase/recombinase | 4.0 |
|  | BT2644 | DNA topoisomerase I | 2.1 |
|  | BT2890 | transposase | 1.6 |
|  | BT2980 | transposase | 2.0 |
|  | BT2983 | hypothetical protein | 1.9 |
|  | BT2985 | DNA repair protein | 1.9 |
|  | BT2986 | integrase | 1.7 |
|  | BT2990 | hypothetical protein | 1.6 |
|  | BT3020 | conserved hypothetical protein | 1.9 |
|  | BT3041 | hypothetical protein | 2.3 |
|  | BT3266 | excinuclease ABC subunit C | 1.2 |
|  | BT3557 | putative transposase | 2.2 |
|  | BT3641 | conserved hypothetical protein | 1.6 |
|  | BT3932 | DNA primase | 1.3 |
|  | BT4009 | integrase | 1.7 |
|  | BT4010 | conserved hypothetical protein | 1.3 |
|  | BT4022 | integrase | 2.1 |
|  | BT4023 | transposase | 1.6 |
|  | BT4025 | hypothetical protein | 1.5 |
|  | BT4104 | DNA helicase | 1.6 |
|  | BT4231 | hypothetical protein | 1.6 |
|  | BT4432 | putative non-specific DNA-binding protein HU-1 | 1.5 |
|  | BT4437 | transposase | 1.9 |
|  | BT4469 | conserved hypothetical protein | 1.9 |
|  | BT4550 | putative transposase | 1.8 |
|  | BT4603 | conserved hypothetical protein | 1.6 |
|  | BT4610 | RecA | 1.4 |
|  | BT4617 | integrase | 1.9 |
|  | BT4624 | putative resolvase | 1.7 |
|  | BT4739 | transposase | 1.7 |
|  |  |  |  |
| Cell cycle control, cell division, chromosome partitioning |  |  |  |
|  | BT0098 | conserved protein found in conjugate transposon | 2.1 |
|  | BT0116 | arsenical pump-driving ATPase | 1.7 |
|  | BT0802 | arsenical pump-driving ATPase | 2.1 |
|  | BT2245 | hypothetical protein | 1.3 |
|  | BT2303 | conserved protein found in conjugate transposon | 1.7 |
|  | BT2526 | hypothetical protein | 1.6 |
|  | BT2609 | conserved protein found in conjugate transposon | 1.5 |
|  | BT3166 | conserved hypothetical protein | 1.3 |
|  | BT3210 | cell division protein FtsX | 1.4 |
|  | BT3421 | hypothetical protein | 1.7 |
|  | BT3464 | putative peptidase | 2.0 |
|  | BT3540 | hypothetical protein | 1.7 |
|  | BT3813 | rod shape-determining protein MreB | 1.4 |
|  | BT3817 | rod shape-determining protein rodA | 1.3 |
|  | BT3895 | Mrp/Nbp35 family ATP-binding protein | 1.9 |
|  | BT3912 | conserved hypothetical protein | 1.5 |
|  | BT4016 | hypothetical protein | 1.9 |
|  | BT4222 | conserved hypothetical protein | 2.8 |
|  | BT4334 | FtsK/SpoIIIE family protein | 1.3 |
|  | BT4574 | conserved hypothetical protein | 1.2 |
|  | BT4762 | conserved protein found in conjugate transposon | 1.4 |
|  |  |  |  |
| Defense mechanisms |  |  |  |
|  | BT0012 | conserved hypothetical protein | 2.0 |
|  | BT0295 | ABC transporter ATP-binding protein | 1.5 |
|  | BT0299 | cation efflux system (AcrB/AcrD/AcrF family) | 1.9 |
|  | BT0305 | putative aminoglycoside efflux pump (Acriflavine resistance protein) | 1.4 |
|  | BT0588 | BexA, multidrug efflux pump | 1.5 |
|  | BT0672 | drug efflux protein | 2.1 |
|  | BT0696 | putative membrane protein | 1.9 |
|  | BT0706 | putative N-acetylmuramoyl-L-alanine amidase | 5.0 |
|  | BT0859 | conserved hypothetical protein | 1.7 |
|  | BT0864 | putative permease | 1.4 |
|  | BT1000 | conserved hypothetical protein | 1.6 |
|  | BT1193 | conserved hypothetical protein | 2.2 |
|  | BT1268 | transporter, AcrB/D/F family | 1.4 |
|  | BT1466 | ABC transporter ATP-binding protein | 1.3 |
|  | BT1519 | N-acetylmuramoyl-L-alanine amidase | 2.4 |
|  | BT1599 | BexA, membrane protein | 1.6 |
|  | BT1641 | N-acetylmuramoyl-L-alanine amidase | 2.5 |
|  | BT1706 | putative N-acetylmuramoyl-L-alanine amidase | 2.0 |
|  | BT1900 | BexA, putative cation effux pump | 2.1 |
|  | BT2027 | N-acetylmuramoyl-L-alanine amidase | 1.9 |
|  | BT2118 | transporter, AcrB/D/F family | 1.2 |
|  | BT2185 | hypothetical protein | 2.0 |
|  | BT2495 | ABC transporter, putative permease | 1.6 |
|  | BT2589 | type I restriction enzyme, M subunit | 1.4 |
|  | BT2941 | multidrug resistance protein mexB (multidrug-efflux protein) | 2.6 |
|  | BT4288 | ABC transporter ATP-binding protein | 1.4 |
|  | BT4541 | putative Type I restriction modification DNA specificity protein | 2.1 |
|  | BT4542 | Type I restriction enzyme EcoR124II specificity protein | 1.7 |
|  | BT4625 | DNA modification methylase BstVI | 1.6 |
|  |  |  |  |
| Signal transduction mechanisms |  |  |  |
|  | BT0015 | rteB, two-component system response regulator | 2.6 |
|  | BT0115 | arsenate reductase | 2.3 |
|  | BT0204 | conserved hypothetical protein | 1.2 |
|  | BT0267 | Hybrid two-component system | 1.3 |
|  | BT0324 | hypothetical protein | 1.3 |
|  | BT0520 | transcriptional regulator | 1.2 |
|  | BT0536 | two-component system sensor protein | 1.7 |
|  | BT0654 | two-component sensor histidine kinase | 2.1 |
|  | BT0681 | two-component system sensor histidine kinase | 1.6 |
|  | BT0700 | GTP pyrophosphokinase | 1.4 |
|  | BT0927 | two-component system sensor histidine kinase | 1.1 |
|  | BT0989 | two-component system sensor histidine kinase | 1.4 |
|  | BT0990 | two-component system response regulator | 1.4 |
|  | BT1234 | conserved hypothetical protein | 1.3 |
|  | BT1286 | putative two-component system response regulator | 2.2 |
|  | BT1318 | signaling protein without kinase domain | 1.4 |
|  | BT1470 | putative two-component system sensor histidine kinase | 1.6 |
|  | BT1545 | conserved hypothetical protein, putative regulatory protein with a cAMP-binding domain, | 1.6 |
|  | BT1754 | Hybrid two-component system | 1.4 |
|  | BT1795 | putative two-component system sensor protein, no kinase domain | 1.7 |
|  | BT1800 | putative two-component system sensor histidine kinase | 1.5 |
|  | BT1833 | two-component system sensor histidine kinase | 1.3 |
|  | BT2102 | putative cAMP-binding domain, regulatory protein | 1.8 |
|  | BT2181 | transcriptional regulator | 1.3 |
|  | BT2420 | two-component system histidine kinase, FrrD | 2.7 |
|  | BT2421 | FrrC, osmosensitive K+ channel histidine kinase | 2.1 |
|  | BT2433 | two-component system response regulator | 1.1 |
|  | BT2574 | two-component system sensor histidine kinase | 1.4 |
|  | BT2618 | two-component system response regulator | 1.7 |
|  | BT2826 | Hybrid two-component system | 1.4 |
|  | BT3134 | Hybrid two-component system | 1.5 |
|  | BT3150 | transcriptional regulator | 1.7 |
|  | BT3302 | Hybrid two-component system | 2.0 |
|  | BT3316 | hypothetical protein | 1.7 |
|  | BT3543 | hypothetical protein | 1.6 |
|  | BT3645 | two-component system sensor histidine kinase | 1.5 |
|  | BT3678 | Hybrid two-component system | 1.5 |
|  | BT3786 | Hybrid two-component system | 2.2 |
|  | BT3801 | rteB, two-component system response regulator | 1.3 |
|  | BT4236 | Hybrid two-component system | 1.9 |
|  | BT4567 | putative two-component system sensor but no kinase domain | 1.7 |
|  | BT4663 | two-component system sensor histidine kinase/response | 1.8 |
|  |  |  |  |
| Cell wall/membrane/envelope biogenesis |  |  |  |
|  | BT0047 | putative glycosyltransferase | 1.4 |
|  | BT0251 | dolichol-phosphate mannosyltransferase | 2.1 |
|  | BT0304 | acriflavine resistance protein A precursor | 1.5 |
|  | BT0378 | capsular polysaccharide biosynthesis protein capD | 2.3 |
|  | BT0382 | capsular polysaccharide biosynthesis protein Cps4K | 1.6 |
|  | BT0391 | putative protein involved in capsular polysaccharide biosynthesis | 2.4 |
|  | BT0396 | putative UDP-GlcNAc:undecaprenylphosphate GlcNAc-1-phosphate transferase | 2.0 |
|  | BT0398 | polysaccharide export outer membrane protein | 2.4 |
|  | BT0463 | glucose-1-phosphate thymidylyltransferase | 1.6 |
|  | BT0464 | dTDP-4-dehydrorhamnose 3,5-epimerase | 1.5 |
|  | BT0465 | dTDP-4-dehydrorhamnose reductase | 1.8 |
|  | BT0466 | dTDP-glucose 4,6-dehydratase | 1.9 |
|  | BT0471 | glycosyltransferase | 1.9 |
|  | BT0476 | D-mannose-1-phosphate guanyltransferase | 1.3 |
|  | BT0481 | polysaccharide export outer membrane protein | 1.4 |
|  | BT0554 | glucosamine--fructose-6-phosphate aminotransferase | 1.6 |
|  | BT0560 | outer membrane efflux protein | 1.4 |
|  | BT0600 | nucleotide sugar epimerase | 2.0 |
|  | BT0601 | UDP-N-acetylglucosamine 2-epimerase | 1.2 |
|  | BT0610 | lipopolysaccharide biosynthesis protein, putative glycosyltransferase | 1.5 |
|  | BT0614 | putative tyrosine-protein kinase in cps region | 1.6 |
|  | BT0632 | GTP-binding protein, membrane GTPase lepA | 1.4 |
|  | BT0669 | putative cation efflux system protein | 1.8 |
|  | BT0678 | cation efflux system protein czcC | 2.3 |
|  | BT0725 | dTDP-4-dehydrorhamnose reductase | 1.5 |
|  | BT0814 | conserved hypothetical protein, putative outer membrane protein | 1.9 |
|  | BT0858 | conserved hypothetical protein | 1.4 |
|  | BT1018 | polygalacturonase (Pectinase) | 1.3 |
|  | BT1059 | putative UDP-glucose 4-epimerase | 1.3 |
|  | BT1163 | conserved hypothetical protein | 1.2 |
|  | BT1167 | conserved hypothetical protein | 1.6 |
|  | BT1212 | outer membrane efflux protein | 1.7 |
|  | BT1250 | conserved hypothetical protein, putative outer membrane efflux protein | 2.0 |
|  | BT1267 | outer membrane protein | 1.9 |
|  | BT1269 | component of multidrug efflux system | 2.0 |
|  | BT1467 | ABC transporter permease | 1.4 |
|  | BT1468 | putative outer membrane efflux protein | 2.1 |
|  | BT1475 | ABC transporter, permease protein | 1.7 |
|  | BT1505 | hypothetical protein | 1.5 |
|  | BT1511 | outer membrane protein OmpA | 2.5 |
|  | BT1550 | conserved hypothetical protein | 1.5 |
|  | BT1712 | glycosyltransferase | 2.3 |
|  | BT1722 | putative protein involved in capsular polysaccharide biosynthesis | 1.6 |
|  | BT1791 | outer membrane protein | 1.3 |
|  | BT1921 | hypothetical protein | 2.5 |
|  | BT1967 | multidrug efflux protein | 2.1 |
|  | BT2086 | choloylglycine hydrolase | 2.0 |
|  | BT2152 | putative acetyltransferase | 1.3 |
|  | BT2251 | putative membrane fusion protein precursor | 1.7 |
|  | BT2257 | conserved hypothetical protein | 1.1 |
|  | BT2442 | major outer membrane protein OmpA | 1.6 |
|  | BT2497 | membrane fusion efflux protein | 1.4 |
|  | BT2588 | conserved hypothetical protein | 1.8 |
|  | BT2608 | hypothetical protein | 1.7 |
|  | BT2747 | 3-deoxy-D-manno-octulosonic-acid transferase | 2.1 |
|  | BT2762 | TonB | 2.2 |
|  | BT2795 | outer membrane efflux protein precursor | 2.0 |
|  | BT2838 | conserved hypothetical protein | 1.7 |
|  | BT2861 | putative outer membrane protein | 2.8 |
|  | BT2864 | putative glycosyltransferase involved in lipopolysaccharide biosynthesis | 1.5 |
|  | BT2866 | putative glycosyltransferase | 2.2 |
|  | BT2873 | putative stress protein | 2.8 |
|  | BT2876 | putative glycosyltransferase | 1.6 |
|  | BT2880 | putative nucleotide-sugar dehydratase | 2.9 |
|  | BT2882 | glycosyltransferase | 2.4 |
|  | BT2927 | conserved hypothetical protein, putative cell wall-associated protein precursor | 1.4 |
|  | BT2928 | hypothetical protein | 1.8 |
|  | BT2942 | putative multidrug resistance protein | 2.3 |
|  | BT3043 | putative xylanase | 1.3 |
|  | BT3075 | NAD-dependent epimerase/dehydratase family protein | 1.3 |
|  | BT3291 | hypothetical protein | 2.1 |
|  | BT3362 | heptosyltransferase | 1.1 |
|  | BT3366 | putative glycosyltransferase yibD | 1.2 |
|  | BT3367 | putative glycosyltransferase | 1.2 |
|  | BT3448 | UDP-N-acetylglucosamine--N-acetylmuramyl- (pentapeptide) pyrophosphoryl-undecaprenol N-acetylglucosamine transferase | 1.6 |
|  | BT3533 | conserved hypothetical protein | 3.4 |
|  | BT3898 | TonB | 1.7 |
|  | BT4053 | putative membrane transport protein | 2.3 |
|  | BT4149 | exo-poly-alpha-D-galacturonosidase precursor | 1.5 |
|  | BT4280 | putative glycosyltransferase | 2.6 |
|  | BT4281 | putative glycosyltransferase | 1.3 |
|  | BT4317 | putative lipoprotein protein | 1.2 |
|  | BT4410 | hypothetical protein | 1.6 |
|  | BT4443 | N-acetylmuramoyl alanine amidase | 2.0 |
|  | BT4693 | cation efflux system protein | 1.5 |
|  | BT4778 | conserved protein found in conjugate transposon | 1.4 |
|  |  |  |  |
| Cell motility |  |  |  |
|  | BT0525 | outer membrane protein, function unknown | 2.3 |
|  | BT2600 | conserved protein found in conjugate transposon | 1.7 |
|  |  |  |  |
| Intracellular trafficking, secretion, and vesicular transport |  |  |  |
|  | BT0102 | conserved hypothetical protein | 2.0 |
|  | BT0492 | integral membrane protein | 1.8 |
|  | BT0921 | conserved hypothetical protein | 1.5 |
|  | BT2296 | conserved protein found in conjugate transposon | 1.3 |
|  | BT2298 | conserved protein found in conjugate transposon | 1.9 |
|  | BT2603 | conserved protein found in conjugate transposon | 2.0 |
|  | BT3318 | signal peptidase I | 2.0 |
|  | BT4622 | mobilization protein BmgA | 1.9 |
|  |  |  |  |
| Posttranslational modification, protein turnover, chaperones |  |  |  |
|  | BT0165 | peptidyl-prolyl cis-trans isomerase | 1.2 |
|  | BT0781 | hypothetical protein | 1.4 |
|  | BT0800 | conserved hypothetical protein | 2.2 |
|  | BT0833 | conserved hypothetical protein | 1.8 |
|  | BT0987 | putative cytochrome C-type biogenesis protein | 2.1 |
|  | BT1244 | chaperone protein dnaJ | 1.8 |
|  | BT2295 | hypothetical protein | 1.5 |
|  | BT2474 | cytochrome c biogenesis protein ccsA | 2.2 |
|  | BT2577 | conserved hypothetical protein | 1.5 |
|  | BT2745 | thiol:disulfide interchange protein | 1.9 |
|  | BT2811 | alkyl hydroperoxide reductase subunit F | 2.1 |
|  | BT2845 | conserved hypothetical protein | 1.2 |
|  | BT2846 | magnesium chelatase, subunit ChlI | 1.3 |
|  | BT2956 | putative pyruvate formate-lyase 3 activating enzyme | 1.8 |
|  | BT2984 | hypothetical protein | 1.7 |
|  | BT2996 | hypothetical protein | 1.8 |
|  | BT3069 | putative disulphide-isomerase | 5.5 |
|  | BT3418 | putative thiol:disulfide interchange protein | 1.3 |
|  | BT3846 | peptidyl-prolyl cis-trans isomerase | 1.5 |
|  |  |  |  |
| Energy production and conversion |  |  |  |
|  | BT0041 | F420H2:quinone oxidoreductase | 1.7 |
|  | BT0072 | putative ABC transporter, ATP-binding protein | 2.1 |
|  | BT0181 | putative NADH dehydrogenase/NAD(P)H nitroreductase | 1.8 |
|  | BT0468 | putative F420H2-dehydrogenase 40 kDa subunit | 2.4 |
|  | BT0775 | ferredoxin-type protein | 1.3 |
|  | BT0963 | putative ferredoxin-type protein | 1.6 |
|  | BT1148 | putative NADH dehydrogenase/NAD(P)H nitroreductase | 1.7 |
|  | BT1223 | Na+/H+-dicarboxylate symporter | 1.7 |
|  | BT1270 | putative Na+/H+ antiporter | 1.2 |
|  | BT1594 | putative ferredoxin | 1.7 |
|  | BT2292 | conserved protein found in conjugate transposon | 3.1 |
|  | BT2346 | iron-sulfur cluster-binding protein | 2.0 |
|  | BT2444 | hypothetical protein | 1.3 |
|  | BT2478 | conserved hypothetical protein | 2.0 |
|  | BT2552 | putative butyrate kinase | 1.3 |
|  | BT2587 | hypothetical protein | 1.8 |
|  | BT2955 | formate acetyltransferase 2 | 1.6 |
|  | BT3168 | putative Fe-S oxidoreductase | 1.4 |
|  | BT3245 | putative iron-sulfur cluster-binding protein | 1.9 |
|  | BT3252 | hypothetical protein | 1.5 |
|  | BT3411 | pyrophosphate-energized vacuolar membrane proton pump | 1.6 |
|  | BT4027 | hypothetical protein | 1.6 |
|  | BT4064 | NADH dehydrogenase I, chain H | 1.5 |
|  | BT4143 | putative iron-sulfur cluster binding protein | 1.9 |
|  | BT4735 | hypothetical protein | 2.9 |
|  | BT4748 | hypothetical protein | 2.7 |
|  |  |  |  |
| Carbohydrate transport and metabolism |  |  |  |
|  | BT0129 | mannonate dehydratase | 1.5 |
|  | BT0145 | putative beta-xylosidase | 2.0 |
|  | BT0196 | putative hexose phosphate transport protein | 2.0 |
|  | BT0338 | conserved hypothetical protein | 2.8 |
|  | BT0339 | alpha-glucosidase | 2.0 |
|  | BT0348 | alpha-L-arabinofuranosidase | 1.6 |
|  | BT0443 | thermostable beta-glucosidase B | 1.2 |
|  | BT0486 | altronate hydrolase | 1.2 |
|  | BT0506 | beta-hexosaminidase precursor | 2.1 |
|  | BT0675 | N-acetylglucosamine-6-phosphate deacetylase | 1.3 |
|  | BT0973 | hypothetical protein | 1.5 |
|  | BT0983 | beta-galactosidase | 1.8 |
|  | BT0992 | beta-galactosidase | 2.6 |
|  | BT0993 | beta-galactosidase | 2.7 |
|  | BT1051 | beta-hexosaminidase precursor | 1.5 |
|  | BT1397 | putative membrane protein | 2.2 |
|  | BT1438 | putative membrane efflux protein | 2.1 |
|  | BT1477 | conserved hypothetical protein | 1.7 |
|  | BT1625 | conserved hypothetical protein | 1.9 |
|  | BT1626 | beta-galactosidase | 1.7 |
|  | BT1778 | beta-glucosidase (gentiobiase) | 2.0 |
|  | BT1780 | periplasmic beta-glucosidase precursor | 1.6 |
|  | BT2104 | ROK family member transcriptional repressor | 1.5 |
|  | BT2105 | putative alpha-1,2-mannosidase | 1.4 |
|  | BT2111 | conserved hypothetical protein | 1.7 |
|  | BT2112 | conserved hypothetical protein | 1.8 |
|  | BT2147 | O-acetyl transferase | 1.8 |
|  | BT2190 | beta-galactosidase | 1.8 |
|  | BT2192 | conserved hypothetical protein | 1.3 |
|  | BT2248 | putative integral membrane protein | 1.8 |
|  | BT2402 | phosphoglycerate mutase | 1.6 |
|  | BT2506 | hypothetical protein | 1.4 |
|  | BT2567 | putative permease | 1.6 |
|  | BT2854 | periplasmic beta-glucosidase precursor | 2.1 |
|  | BT2855 | beta-mannosidase precursor | 1.6 |
|  | BT2909 | putative L-arabinose isomerase | 1.4 |
|  | BT2953 | L-rhamnose-proton symporter | 1.6 |
|  | BT2969 | beta-galactosidase | 2.1 |
|  | BT3092 | beta-galactosidase | 1.4 |
|  | BT3096 | alpha-L-arabinofuranosidase A precursor | 1.3 |
|  | BT3123 | conserved hypothetical protein | 1.3 |
|  | BT3130 | conserved hypothetical protein | 1.5 |
|  | BT3132 | sialic acid-specific 9-O-acetylesterase | 2.0 |
|  | BT3178 | beta-hexosaminidase precursor | 1.7 |
|  | BT3179 | beta-galactosidase | 1.4 |
|  | BT3198 | conserved hypothetical protein | 1.5 |
|  | BT3231 | 4-deoxy-L-threo-5-hexosulose-uronate ketol-isomerase | 1.5 |
|  | BT3300 | beta-glucosidase | 1.4 |
|  | BT3301 | putative alpha-1,6-mannanase | 1.6 |
|  | BT3314 | thermostable beta-glucosidase B | 1.3 |
|  | BT3501 | putative alpha-1,6-mannanase | 1.8 |
|  | BT3598 | beta-hexosaminidase precursor | 1.4 |
|  | BT3599 | beta-mannosidase precursor | 2.3 |
|  | BT3605 | N-acylglucosamine 2-epimerase | 1.4 |
|  | BT3685 | conserved hypothetical protein | 1.4 |
|  | BT3780 | conserved hypothetical protein | 3.5 |
|  | BT3784 | putative alpha-1,2-mannosidase | 1.9 |
|  | BT3956 | conserved hypothetical protein | 2.0 |
|  | BT4072 | alpha-mannosidase | 1.2 |
|  | BT4073 | conserved hypothetical protein | 1.4 |
|  | BT4091 | sialic acid-specific 9-O-acetylesterase | 1.6 |
|  | BT4110 | putative pectinesterase precursor | 1.6 |
|  | BT4136 | conserved hypothetical protein | 1.4 |
|  | BT4179 | polysaccharide deacetylase | 1.7 |
|  | BT4238 | putative permease | 1.4 |
|  | BT4460 | SusC/D paralog | 3.1 |
|  | BT4717 | integral membrane protein, putative permease | 1.9 |
|  |  |  |  |
| Amino acid transport and metabolism |  |  |  |
|  | BT0201 | histidinol dehydrogenase | 1.6 |
|  | BT0342 | conserved hypothetical protein | 2.4 |
|  | BT0414 | sulfate adenylyltransferase subunit 2 | 1.3 |
|  | BT0457 | sialic acid-specific 9-O-acetylesterase | 1.3 |
|  | BT0469 | hypothetical protein | 1.5 |
|  | BT0528 | putative N-(5'-phosphoribosyl)anthranilate isomerase | 1.9 |
|  | BT0530 | anthranilate phosphoribosyltransferase | 2.6 |
|  | BT0532 | anthranilate synthase component I | 1.6 |
|  | BT0533 | tryptophan synthase beta chain | 2.4 |
|  | BT0545 | nitrogen regulatory protein P-II | 1.8 |
|  | BT0551 | asparagine synthetase B [glutamine-hydrolyzing] | 1.9 |
|  | BT0587 | prolyl oligopeptidase family protein | 1.2 |
|  | BT0821 | putative permease | 2.4 |
|  | BT1082 | Na+/solute symporter | 2.0 |
|  | BT1147 | glycine dehydrogenase [decarboxylating] | 1.3 |
|  | BT1378 | imidazole glycerol phosphate synthase subunit hisF | 1.5 |
|  | BT1398 | putative aminotransferase B | 1.7 |
|  | BT1615 | aminoacyl-histidine dipeptidase | 1.5 |
|  | BT1719 | sulfopyruvate decarboxylase subunit beta | 1.2 |
|  | BT1743 | hypothetical protein | 1.6 |
|  | BT1749 | glycine betaine-binding protein precursor | 2.6 |
|  | BT1750 | glycine betaine/L-proline transport system permease | 2.1 |
|  | BT1751 | Glycine betaine transport ATP-binding protein | 1.6 |
|  | BT1852 | cysteine synthase A | 1.3 |
|  | BT1860 | 3-isopropylmalate dehydratase large subunit | 2.5 |
|  | BT1874 | SusC/D paralog | 1.9 |
|  | BT1901 | lysine-sensitive aspartokinase III | 2.7 |
|  | BT1902 | pyrroline-5-carboxylate reductase | 1.5 |
|  | BT2076 | acetohydroxyacid synthase small subunit | 1.5 |
|  | BT2186 | 3-phosphoshikimate 1-carboxyvinyltransferase | 2.5 |
|  | BT2387 | O-acetylhomoserine (thiol)-lyase | 1.9 |
|  | BT2401 | threonine synthase | 2.5 |
|  | BT2403 | aspartokinase/homoserine dehydrogenase | 1.4 |
|  | BT2413 | homoserine O-succinyltransferase | 1.2 |
|  | BT2694 | putative urocanate hydratase | 1.5 |
|  | BT2814 | dihydrodipicolinate synthase | 1.8 |
|  | BT2875 | serine O-acetyltransferase | 1.5 |
|  | BT3409 | aminotransferase, putative cysteine desulfurase | 1.9 |
|  | BT3420 | hypothetical protein | 2.3 |
|  | BT3617 | sorbitol dehydrogenase | 1.5 |
|  | BT3757 | pyrroline-5-carboxylate reductase | 1.4 |
|  | BT3821 | 5,10-methylenetetrahydrofolate reductase | 1.9 |
|  | BT4360 | hypothetical protein | 2.2 |
|  | BT4385 | di-tripeptide ABC transporter | 2.5 |
|  | BT4474 | conserved hypothetical protein | 1.6 |
|  | BT4502 | conserved hypothetical protein | 1.4 |
|  | BT4512 | putative zinc-type alcohol dehydrogenase | 1.5 |
|  | BT4546 | conserved hypothetical protein | 2.8 |
|  | BT4751 | hypothetical protein | 1.9 |
|  |  |  |  |
| Nucleotide transport and metabolism |  |  |  |
|  | BT0184 | uridine kinase (uridine monophosphokinase) | 1.7 |
|  | BT0881 | hypothetical protein | 1.8 |
|  | BT1009 | dihydroorotate dehydrogenase | 1.5 |
|  | BT1588 | conserved hypothetical protein | 1.6 |
|  | BT4260 | deoxycytidylate deaminase | 1.3 |
|  |  |  |  |
| Coenzyme transport and metabolism |  |  |  |
|  | BT0106 | conserved hypothetical protein | 1.3 |
|  | BT0343 | methylcobamide:CoM methyltransferase mtbA | 2.0 |
|  | BT0495 | cobalamin biosynthesis protein | 2.5 |
|  | BT0648 | molybdopterin biosynthesis protein | 1.5 |
|  | BT0790 | phosphomethylpyrimidine kinase | 1.3 |
|  | BT0870 | 8-amino-7-oxononanoate synthase | 1.4 |
|  | BT0960 | putative TonB-dependent outer membrane receptor | 1.9 |
|  | BT1089 | lipoate-protein ligase B | 1.5 |
|  | BT1446 | dethiobiotin synthetase | 1.2 |
|  | BT1849 | hypothetical protein | 1.5 |
|  | BT2168 | coproporphyrinogen III oxidase | 1.3 |
|  | BT2238 | putative biotin--(acetyl-CoA carboxylase) synthetase | 1.3 |
|  | BT2301 | conserved protein found in conjugate transposon | 2.3 |
|  | BT2396 | putative membrane transporter involved in nicotinamide mononucleotide transport | 1.4 |
|  | BT2649 | ThiF family protein, ubiquitin-activating enzyme | 2.9 |
|  | BT2797 | adenosylhomocysteinase | 1.2 |
|  | BT3216 | 2-amino-4-hydroxy-6- hydroxymethyldihydropteridine | 2.1 |
|  | BT3261 | octaprenyl-diphosphate synthase | 1.2 |
|  | BT4258 | putative 5-formyltetrahydrofolate cyclo-ligase | 2.0 |
|  |  |  |  |
| Lipid transport and metabolism |  |  |  |
|  | BT0122 | 3-oxoacyl-[acyl-carrier-protein] synthase III | 1.4 |
|  | BT0316 | putative hemin receptor | 1.4 |
|  | BT0871 | conserved hypothetical protein | 1.8 |
|  | BT1087 | putative xylanase | 1.7 |
|  | BT1170 | hypothetical protein | 2.1 |
|  | BT1574 | putative outer membrane protein precursor | 1.4 |
|  | BT2002 | 1-deoxy-D-xylulose 5-phosphate reductoisomerase | 1.2 |
|  | BT2046 | putative cardiolipin synthetase | 1.4 |
|  | BT2075 | acyl-ACP thioesterase | 2.6 |
|  | BT2090 | methylmalonyl-CoA mutase large subunit | 2.7 |
|  | BT2114 | hypothetical protein | 1.4 |
|  | BT2782 | long-chain-fatty-acid--CoA ligase | 1.8 |
|  | BT3976 | hypothetical protein | 1.4 |
|  | BT4367 | putative outer membrane protein | 1.2 |
|  |  |  |  |
| Inorganic ion transport and metabolism |  |  |  |
|  | BT0114 | putative symporter, arsenic resistance membrane protein | 2.7 |
|  | BT0189 | conserved hypothetical protein, putative anti-sigma factor | 1.6 |
|  | BT0190 | SusC/D paralog | 1.8 |
|  | BT0215 | iron uptake regulatory protein | 1.5 |
|  | BT0411 | CysQ, sulfite synthesis pathway protein | 1.7 |
|  | BT0496 | TonB-dependent receptor | 4.1 |
|  | BT0504 | TonB-dependent outer membrane receptor | 1.7 |
|  | BT0680 | cation efflux system protein czcA | 2.2 |
|  | BT0753 | putative anti-sigma factor | 1.9 |
|  | BT0988 | Mg2+ transport ATPase protein B | 1.5 |
|  | BT1052 | putative anti-sigma factor | 2.8 |
|  | BT1092 | putative heavy-metal binding protein | 2.5 |
|  | BT1093 | conserved hypothetical protein, putative TonB-dependent outer membrane receptor protein | 2.6 |
|  | BT1279 | putative anti-sigma factor | 1.7 |
|  | BT1321 | putative phosphate transport ATP-binding protein | 1.9 |
|  | BT1322 | putative ABC transporter permease protein | 1.9 |
|  | BT1323 | putative ABC transporter permease protein | 1.4 |
|  | BT1324 | phosphate ABC transporter, phosphate-binding protein | 1.5 |
|  | BT1388 | integral membrane protein | 1.2 |
|  | BT1622 | N-acetylgalactosamine-6-sulfatase precursor | 1.8 |
|  | BT1799 | putative vitamin B12 receptor precursor | 1.2 |
|  | BT1875 | SusC/D paralog | 1.4 |
|  | BT2020 | putative phosphate/sulphate permeases | 1.6 |
|  | BT2032 | SusC/D paralog | 1.3 |
|  | BT2065 | putative TonB-linked outer membrane receptor | 1.9 |
|  | BT2094 | TonB-dependent receptor | 1.3 |
|  | BT2172 | SusC/D paralog | 1.6 |
|  | BT2362 | SusC/D paralog | 1.8 |
|  | BT2423 | K+-transporting ATPase C chain | 4.2 |
|  | BT2424 | K+-transporting ATPase B chain | 3.3 |
|  | BT2425 | K+-transporting ATPase A chain | 2.8 |
|  | BT2461 | SusC/D paralog | 2.0 |
|  | BT2462 | putative anti-sigma factor | 3.1 |
|  | BT2511 | putative transcription regulator | 1.5 |
|  | BT2531 | SusC/D paralog | 1.6 |
|  | BT2561 | putative anti-sigma factor | 2.9 |
|  | BT2952 | SusC/D paralog | 2.5 |
|  | BT3011 | putative anti-sigma factor | 1.6 |
|  | BT3016 | TonB-dependent receptor | 2.6 |
|  | BT3036 | conserved hypothetical protein | 1.8 |
|  | BT3060 | hypothetical protein | 2.7 |
|  | BT3090 | SusC/D paralog | 1.6 |
|  | BT3107 | arylsulfatase (aryl-sulfate sulphohydrolase) | 1.6 |
|  | BT3174 | SusC/D paralog | 1.6 |
|  | BT3177 | mucin-desulfating sulfatase | 1.8 |
|  | BT3249 | putative zinc ABC transporter, zinc-binding protein | 1.4 |
|  | BT3270 | putative anti-sigma factor | 1.8 |
|  | BT3271 | SusC/D paralog | 1.9 |
|  | BT3297 | SusC/D paralog | 1.3 |
|  | BT3333 | arylsulfatase A precursor (ASA) | 1.7 |
|  | BT3518 | putative anti-sigma factor | 3.6 |
|  | BT3670 | SusC/D paralog | 1.3 |
|  | BT3749 | putative anti-sigma factor | 4.1 |
|  | BT3977 | conserved hypothetical protein | 1.4 |
|  | BT4055 | TonB-dependent receptor | 1.4 |
|  | BT4248 | putative anti-sigma factor | 4.2 |
|  | BT4356 | putative anti-sigma factor | 4.2 |
|  | BT4403 | putative anti-sigma factor | 2.0 |
|  | BT4416 | Cu2+ homeostasis protein CutC | 1.5 |
|  | BT4568 | conserved hypothetical protein | 2.4 |
|  | BT4650 | voltage-gated chloride channel protein | 1.6 |
|  | BT4706 | putative anti-sigma factor | 2.5 |
|  |  |  |  |
| Secondary metabolites biosynthesis, transport and catabolism |  |  |  |
|  | BT0130 | putative oxidoreductase | 2.0 |
|  | BT0972 | putative oxidoreductase | 1.6 |
|  | BT2333 | conserved hypothetical protein | 1.6 |
|  | BT2692 | imidazolonepropionase | 1.7 |
|  | BT2972 | conserved hypothetical protein | 2.7 |
|  | BT3838 | conserved hypothetical protein | 2.3 |
|  |  |  |  |
| General function prediction only |  |  |  |
|  | BT0031 | conserved hypothetical protein | 2.3 |
|  | BT0124 | NADH:ubiquinone oxidoreductase subunit | 1.3 |
|  | BT0163 | conserved hypothetical protein | 2.3 |
|  | BT0164 | conserved hypothetical protein | 1.4 |
|  | BT0168 | conserved hypothetical protein | 2.2 |
|  | BT0194 | hypothetical protein | 1.5 |
|  | BT0238 | transcriptional regulator | 1.4 |
|  | BT0264 | conserved hypothetical protein | 2.2 |
|  | BT0269 | SusC/D paralog | 1.9 |
|  | BT0296 | putative xanthan lyase XalB precursor | 1.4 |
|  | BT0303 | putative patatin-like phospholipase | 1.8 |
|  | BT0340 | trimethylamine corrinoid protein 2 (TCP 2) | 1.6 |
|  | BT0390 | putative O-antigen export protein | 1.9 |
|  | BT0472 | putative acyltransferase in colanic acid biosynthesis | 2.3 |
|  | BT0474 | D-glycero-D-manno-heptose 1-phosphate kinase | 2.0 |
|  | BT0479 | putative glycosyltransferase | 1.7 |
|  | BT0758 | acetyltransferase | 1.8 |
|  | BT0796 | conserved hypothetical protein | 1.4 |
|  | BT0838 | putative RNA methyltransferase | 2.1 |
|  | BT0896 | putative patatin-like phospholipase | 1.3 |
|  | BT0917 | competence-damage inducible protein | 1.5 |
|  | BT0970 | haloacid dehalogenase-like hydrolase | 1.2 |
|  | BT1083 | putative GTP-binding protein | 1.5 |
|  | BT1121 | conserved hypothetical protein, with a carboxymuconolactone decarboxylase domain | 2.1 |
|  | BT1177 | putative polysaccharide biosynthesis protein | 1.4 |
|  | BT1190 | putative ATP GTP-binding protein | 2.1 |
|  | BT1229 | conserved hypothetical protein, putative membrane protein | 1.8 |
|  | BT1239 | putative metal-dependent membrane protease | 2.1 |
|  | BT1263 | putative protease I | 2.2 |
|  | BT1384 | conserved hypothetical protein | 1.5 |
|  | BT1402 | iron-sulfur flavoprotein | 2.1 |
|  | BT1429 | putative general stress protein | 1.6 |
|  | BT1463 | putative acetyltransferase | 1.7 |
|  | BT1473 | conserved hypothetical protein | 1.8 |
|  | BT1616 | conserved hypothetical protein | 1.3 |
|  | BT1677 | putative hydrolase | 1.6 |
|  | BT1707 | conserved hypothetical protein | 1.6 |
|  | BT1713 | putative acylneuraminate cytidylyltransferase | 6.9 |
|  | BT1717 | putative lipopolysaccharide biosynthesis protein | 1.5 |
|  | BT1807 | conserved protein, with rhomboid family domain | 2.1 |
|  | BT1837 | putative GTP-binding protein, putative GTPase | 1.6 |
|  | BT1890 | conserved hypothetical protein | 1.3 |
|  | BT1894 | TPR-repeat-containing proteins | 1.3 |
|  | BT2180 | conserved hypothetical protein | 1.5 |
|  | BT2188 | putative oxidoreductase | 1.8 |
|  | BT2189 | aminopeptidase | 2.3 |
|  | BT2345 | hypothetical protein | 1.3 |
|  | BT2379 | hypothetical protein | 1.7 |
|  | BT2384 | acetyltransferase | 1.4 |
|  | BT2417 | conserved hypothetical protein | 2.3 |
|  | BT2457 | putative purple acid phosphatase | 1.3 |
|  | BT2544 | putative metal-dependent membrane protease | 1.8 |
|  | BT2630 | conserved hypothetical protein with endonuclease/exonuclease/phosphatase family domain | 1.5 |
|  | BT2656 | hypothetical protein | 1.3 |
|  | BT2744 | acetyltransferase | 1.1 |
|  | BT2756 | anaerobic C4-dicarboxylate transporter dcuB | 1.6 |
|  | BT2769 | putative serine/threonine-protein kinase pknB | 1.6 |
|  | BT2878 | lipopolysaccharide biosynthesis protein | 1.9 |
|  | BT2912 | putative glycosyslhydrolase | 1.3 |
|  | BT2921 | conserved hypothetical protein | 1.5 |
|  | BT2934 | putative transmembrane transport protein, putative lipopolysaccharide biosynthesis protein | 1.6 |
|  | BT2963 | conserved hypothetical protein | 1.7 |
|  | BT2992 | conserved hypothetical protein | 2.0 |
|  | BT3040 | conserved hypothetical protein | 1.6 |
|  | BT3042 | conserved hypothetical protein | 1.9 |
|  | BT3071 | conserved hypothetical protein | 1.6 |
|  | BT3091 | putative regulatory protein | 2.0 |
|  | BT3111 | hypothetical protein | 1.8 |
|  | BT3112 | conserved hypothetical protein | 1.6 |
|  | BT3257 | putative two-component system sensor histidine kinase | 1.4 |
|  | BT3272 | conserved hypothetical protein, putative outer membrane protein | 1.3 |
|  | BT3284 | conserved hypothetical protein | 1.4 |
|  | BT3385 | conserved hypothetical protein | 1.7 |
|  | BT3442 | conserved hypothetical protein | 1.7 |
|  | BT3545 | hypothetical protein | 2.3 |
|  | BT3548 | ATPase | 1.5 |
|  | BT3570 | TPR-repeat-containing proteins | 2.2 |
|  | BT3642 | Na+-dependent transporter | 1.4 |
|  | BT3894 | putative methyltransferase | 1.5 |
|  | BT4020 | conserved hypothetical protein | 1.4 |
|  | BT4131 | conserved hypothetical protein | 1.4 |
|  | BT4140 | hypothetical protein | 2.2 |
|  | BT4190 | putative S-adenosylmethionine-dependent methytransferase | 1.5 |
|  | BT4292 | conserved hypothetical protein | 2.6 |
|  | BT4302 | conserved hypothetical protein | 2.2 |
|  | BT4372 | conserved hypothetical protein | 1.2 |
|  | BT4465 | conserved hypothetical protein, putative outer membrane protein | 1.6 |
|  | BT4492 | conserved hypothetical protein | 1.9 |
|  | BT4496 | putative oxidoreductase | 1.5 |
|  | BT4509 | conserved hypothetical protein | 2.0 |
|  | BT4526 | conserved hypothetical protein | 2.8 |
|  | BT4528 | conserved hypothetical protein | 2.2 |
|  | BT4557 | hypothetical protein | 1.3 |
|  | BT4616 | conserved hypothetical protein | 1.4 |
|  | BT4697 | transcriptional regulator | 1.5 |
|  |  |  |  |
| Function unknown |  |  |  |
|  | BT0137 | conserved hypothetical protein | 1.5 |
|  | BT0167 | conserved hypothetical protein | 1.9 |
|  | BT0210 | leucine-rich repeat protein, function unknown | 1.9 |
|  | BT0284 | putative peptidoglycan binding protein (LPXTG motif) | 1.3 |
|  | BT0446 | conserved hypothetical protein | 1.7 |
|  | BT0493 | hypothetical protein | 1.9 |
|  | BT0637 | conserved hypothetical protein | 1.9 |
|  | BT0726 | conserved hypothetical protein | 1.5 |
|  | BT0761 | conserved hypothetical protein | 1.8 |
|  | BT0874 | conserved hypothetical protein | 1.7 |
|  | BT0974 | conserved hypothetical protein, putative integral membrane protein | 1.7 |
|  | BT0975 | conserved hypothetical protein, putative integral membrane protein | 1.5 |
|  | BT1003 | conserved hypothetical protein | 1.3 |
|  | BT1256 | conserved hypothetical protein | 1.8 |
|  | BT1276 | conserved hypothetical protein | 1.4 |
|  | BT1394 | conserved hypothetical protein | 1.4 |
|  | BT1422 | conserved hypothetical protein | 1.5 |
|  | BT1423 | conserved hypothetical protein | 1.8 |
|  | BT1444 | conserved hypothetical protein | 1.7 |
|  | BT1455 | protein rtcB | 2.2 |
|  | BT1510 | conserved hypothetical protein | 1.3 |
|  | BT1521 | conserved hypothetical protein | 1.6 |
|  | BT1562 | conserved hypothetical protein | 1.3 |
|  | BT1744 | conserved hypothetical protein | 2.9 |
|  | BT1787 | conserved hypothetical protein | 2.1 |
|  | BT1826 | conserved hypothetical protein | 1.4 |
|  | BT2036 | conserved hypothetical protein | 1.6 |
|  | BT2097 | conserved hypothetical protein | 1.4 |
|  | BT2237 | conserved hypothetical protein | 1.3 |
|  | BT2255 | conserved hypothetical protein | 1.2 |
|  | BT2513 | conserved hypothetical protein | 1.4 |
|  | BT2530 | conserved hypothetical protein, with a conserved domain | 2.6 |
|  | BT2780 | conserved hypothetical protein, putative membrane-associated protein | 1.8 |
|  | BT2848 | hypothetical protein | 1.6 |
|  | BT2911 | conserved hypothetical protein | 1.3 |
|  | BT3066 | conserved hypothetical protein | 1.4 |
|  | BT3180 | conserved hypothetical protein | 1.2 |
|  | BT3378 | conserved hypothetical protein | 1.3 |
|  | BT3390 | conserved hypothetical protein | 1.7 |
|  | BT3619 | conserved hypothetical protein | 1.6 |
|  | BT3674 | conserved hypothetical protein | 1.9 |
|  | BT3863 | conserved hypothetical protein | 1.9 |
|  | BT4195 | conserved hypothetical protein | 1.7 |
|  | BT4212 | conserved hypothetical protein | 1.2 |
|  | BT4450 | conserved hypothetical protein | 1.8 |
|  | BT4484 | conserved hypothetical protein | 2.4 |
|  | BT4516 | conserved hypothetical protein | 1.3 |
|  | BT4608 | conserved hypothetical protein | 2.1 |
|  | BT4639 | conserved hypothetical protein, putative membrane protein | 2.5 |
|  | BT4732 | conserved hypothetical protein | 4.5 |
|  |  |  |  |
| Not in COG |  |  |  |
|  | BT0002 | hypothetical protein | 1.8 |
|  | BT0003 | hypothetical protein | 1.9 |
|  | BT0007 | hypothetical protein | 1.8 |
|  | BT0010 | hypothetical protein | 2.4 |
|  | BT0013 | hypothetical protein | 1.7 |
|  | BT0017 | hypothetical protein | 1.8 |
|  | BT0018 | hypothetical protein | 3.3 |
|  | BT0019 | hypothetical protein | 1.9 |
|  | BT0021 | hypothetical protein | 1.5 |
|  | BT0035 | hypothetical protein | 2.0 |
|  | BT0048 | hypothetical protein | 3.4 |
|  | BT0049 | putative glycosyltransferase | 1.4 |
|  | BT0058 | hypothetical protein | 1.8 |
|  | BT0063 | hypothetical protein | 1.6 |
|  | BT0065 | alpha-galactosidase precursor | 1.8 |
|  | BT0067 | hypothetical protein | 1.5 |
|  | BT0071 | hypothetical protein | 1.4 |
|  | BT0074 | hypothetical protein | 2.6 |
|  | BT0080 | hypothetical protein | 1.6 |
|  | BT0084 | conserved protein found in conjugate transposon | 1.6 |
|  | BT0085 | conserved protein found in conjugate transposon | 2.8 |
|  | BT0086 | conserved protein found in conjugate transposon | 2.0 |
|  | BT0089 | conserved protein found in conjugate transposon | 1.9 |
|  | BT0092 | conserved protein found in conjugate transposon | 2.5 |
|  | BT0099 | hypothetical protein | 3.4 |
|  | BT0100 | hypothetical protein | 1.9 |
|  | BT0103 | conserved hypothetical protein | 2.9 |
|  | BT0104 | hypothetical protein | 1.6 |
|  | BT0107 | conserved hypothetical protein | 2.3 |
|  | BT0110 | putative regulatory protein | 2.1 |
|  | BT0111 | hypothetical protein | 1.8 |
|  | BT0136 | hypothetical protein | 1.7 |
|  | BT0141 | SusC/D paralog | 1.4 |
|  | BT0175 | conserved hypothetical protein | 1.3 |
|  | BT0191 | SusC/D paralog | 2.2 |
|  | BT0198 | hypothetical protein | 2.6 |
|  | BT0220 | hypothetical protein | 1.7 |
|  | BT0231 | excisionase | 1.9 |
|  | BT0234 | putative transposase | 2.4 |
|  | BT0253 | hypothetical protein | 1.7 |
|  | BT0255 | hypothetical protein | 2.0 |
|  | BT0262 | hypothetical protein | 1.2 |
|  | BT0263 | hypothetical protein | 1.5 |
|  | BT0274 | hypothetical protein | 1.7 |
|  | BT0276 | hypothetical protein | 1.5 |
|  | BT0277 | conserved hypothetical protein | 1.6 |
|  | BT0325 | hypothetical protein | 3.4 |
|  | BT0385 | conserved hypothetical protein | 1.6 |
|  | BT0395 | hypothetical protein | 1.9 |
|  | BT0402 | hypothetical protein | 1.8 |
|  | BT0403 | hypothetical protein | 2.1 |
|  | BT0404 | hypothetical protein | 2.1 |
|  | BT0405 | hypothetical protein | 2.6 |
|  | BT0407 | hypothetical protein | 1.2 |
|  | BT0451 | SusC/D paralog | 1.4 |
|  | BT0467 | conserved hypothetical protein | 1.4 |
|  | BT0497 | conserved hypothetical protein | 3.5 |
|  | BT0498 | hypothetical protein | 1.8 |
|  | BT0501 | hypothetical protein | 2.0 |
|  | BT0534 | hypothetical protein | 2.0 |
|  | BT0594 | hypothetical protein | 1.5 |
|  | BT0659 | hypothetical protein | 1.4 |
|  | BT0663 | hypothetical protein | 1.3 |
|  | BT0664 | hypothetical protein | 1.3 |
|  | BT0668 | hypothetical protein | 1.8 |
|  | BT0683 | alpha-glucosidase | 3.7 |
|  | BT0708 | hypothetical protein | 3.2 |
|  | BT0709 | hypothetical protein | 2.4 |
|  | BT0724 | hypothetical protein | 1.8 |
|  | BT0731 | hypothetical protein | 1.5 |
|  | BT0739 | hypothetical protein | 1.3 |
|  | BT0759 | hypothetical protein | 1.9 |
|  | BT0779 | hypothetical protein | 2.0 |
|  | BT0799 | hypothetical protein | 1.3 |
|  | BT0839 | hypothetical protein | 1.7 |
|  | BT0854 | hypothetical protein | 1.6 |
|  | BT0880 | hypothetical protein | 1.3 |
|  | BT0930 | hypothetical protein | 2.1 |
|  | BT0932 | hypothetical protein | 1.8 |
|  | BT0933 | hypothetical protein | 1.7 |
|  | BT0939 | hypothetical protein | 2.1 |
|  | BT0941 | hypothetical protein | 1.3 |
|  | BT0946 | hypothetical protein | 1.7 |
|  | BT0948 | hypothetical protein | 1.9 |
|  | BT0949 | hypothetical protein | 2.5 |
|  | BT0950 | hypothetical protein | 1.2 |
|  | BT0968 | conserved hypothetical protein | 1.4 |
|  | BT0977 | hypothetical protein | 1.4 |
|  | BT0979 | hypothetical protein | 1.6 |
|  | BT0984 | hypothetical protein | 2.0 |
|  | BT0985 | putative sialic acid-specific acetylesterase II | 2.4 |
|  | BT1010 | conserved hypothetical protein | 1.8 |
|  | BT1062 | hypothetical protein | 1.9 |
|  | BT1063 | hypothetical protein | 1.7 |
|  | BT1066 | hypothetical protein | 2.0 |
|  | BT1074 | hypothetical protein | 2.8 |
|  | BT1096 | transposase | 2.7 |
|  | BT1097 | hypothetical protein | 1.6 |
|  | BT1112 | transposase | 2.0 |
|  | BT1120 | hypothetical protein | 2.1 |
|  | BT1129 | conserved hypothetical protein | 2.0 |
|  | BT1132 | hypothetical protein | 1.8 |
|  | BT1136 | hypothetical protein | 2.1 |
|  | BT1137 | hypothetical protein | 5.0 |
|  | BT1143 | conserved hypothetical protein | 1.2 |
|  | BT1178 | conserved hypothetical protein | 1.6 |
|  | BT1231 | hypothetical protein | 2.3 |
|  | BT1233 | hypothetical protein | 1.7 |
|  | BT1302 | hypothetical protein | 1.4 |
|  | BT1303 | hypothetical protein | 1.5 |
|  | BT1305 | hypothetical protein | 1.6 |
|  | BT1310 | conserved hypothetical protein | 1.3 |
|  | BT1357 | conserved hypothetical protein | 2.3 |
|  | BT1399 | conserved hypothetical protein | 1.7 |
|  | BT1419 | hypothetical protein | 2.2 |
|  | BT1481 | hypothetical protein | 1.9 |
|  | BT1490 | conserved hypothetical protein, putative surface protein | 2.6 |
|  | BT1506 | hypothetical protein | 1.8 |
|  | BT1561 | hypothetical protein | 1.3 |
|  | BT1581 | hypothetical protein | 1.5 |
|  | BT1586 | hypothetical protein | 2.1 |
|  | BT1589 | hypothetical protein | 1.8 |
|  | BT1590 | hypothetical protein | 1.5 |
|  | BT1611 | hypothetical protein | 1.5 |
|  | BT1637 | hypothetical protein | 2.2 |
|  | BT1667 | hypothetical protein | 1.5 |
|  | BT1679 | hypothetical protein | 1.5 |
|  | BT1701 | hypothetical protein | 1.7 |
|  | BT1766 | putative ribonucleoprotein-related protein | 2.1 |
|  | BT1775 | hypothetical protein | 1.8 |
|  | BT1779 | sialic acid-specific 9-O-acetylesterase | 1.4 |
|  | BT1781 | xylosidase/arabinosidase | 1.7 |
|  | BT1786 | hypothetical protein | 2.3 |
|  | BT1788 | hypothetical protein | 1.8 |
|  | BT1798 | hypothetical protein | 1.2 |
|  | BT1803 | conserved hypothetical protein | 1.9 |
|  | BT1816 | hypothetical protein | 2.5 |
|  | BT1889 | hypothetical protein | 1.3 |
|  | BT1895 | hypothetical protein | 1.9 |
|  | BT1896 | putative cell surface antigen | 2.8 |
|  | BT1899 | conserved hypothetical protein | 1.2 |
|  | BT1903 | hypothetical protein | 2.8 |
|  | BT1912 | hypothetical protein | 2.4 |
|  | BT1926 | hypothetical protein | 2.9 |
|  | BT1927 | hypothetical protein | 2.0 |
|  | BT1938 | hypothetical protein | 1.5 |
|  | BT1941 | hypothetical protein | 1.6 |
|  | BT1943 | hypothetical protein | 1.5 |
|  | BT1944 | putative transposase | 2.5 |
|  | BT1946 | hypothetical protein | 2.0 |
|  | BT1947 | hypothetical protein | 1.9 |
|  | BT1948 | hypothetical protein | 1.6 |
|  | BT1988 | hypothetical protein | 2.5 |
|  | BT1994 | hypothetical protein | 1.8 |
|  | BT1995 | hypothetical protein | 3.0 |
|  | BT2026 | endonuclease | 2.1 |
|  | BT2028 | conserved hypothetical protein | 1.7 |
|  | BT2029 | conserved hypothetical protein | 2.0 |
|  | BT2030 | conserved hypothetical protein | 1.4 |
|  | BT2031 | hypothetical protein | 1.3 |
|  | BT2033 | hypothetical protein | 1.5 |
|  | BT2045 | hypothetical protein | 1.2 |
|  | BT2057 | hypothetical protein | 1.2 |
|  | BT2087 | hypothetical protein | 1.5 |
|  | BT2108 | hypothetical protein | 1.4 |
|  | BT2131 | hypothetical protein | 1.4 |
|  | BT2178 | hypothetical protein | 5.1 |
|  | BT2193 | hypothetical protein | 1.3 |
|  | BT2195 | SusC/D paralog | 1.3 |
|  | BT2201 | SusC/D paralog | 2.2 |
|  | BT2217 | hypothetical protein | 1.2 |
|  | BT2220 | hypothetical protein | 1.8 |
|  | BT2227 | conserved hypothetical protein | 2.8 |
|  | BT2233 | hypothetical protein | 1.9 |
|  | BT2244 | hypothetical protein | 2.2 |
|  | BT2246 | hypothetical protein | 1.5 |
|  | BT2266 | hypothetical protein | 1.7 |
|  | BT2284 | hypothetical protein | 1.7 |
|  | BT2288 | conserved protein found in conjugate transposon | 2.7 |
|  | BT2289 | conserved protein found in conjugate transposon | 1.9 |
|  | BT2290 | conserved protein found in conjugate transposon | 2.0 |
|  | BT2293 | conserved protein found in conjugate transposon | 1.9 |
|  | BT2299 | conserved protein found in conjugate transposon | 1.8 |
|  | BT2300 | conserved protein found in conjugate transposon | 2.6 |
|  | BT2302 | conserved protein found in conjugate transposon | 2.1 |
|  | BT2305 | putative mobilization protein | 1.7 |
|  | BT2309 | hypothetical protein | 1.4 |
|  | BT2315 | hypothetical protein | 2.3 |
|  | BT2319 | conserved hypothetical protein | 2.2 |
|  | BT2322 | putative integrase | 1.4 |
|  | BT2323 | conserved hypothetical protein | 1.5 |
|  | BT2325 | hypothetical protein | 2.3 |
|  | BT2329 | conserved hypothetical protein | 1.6 |
|  | BT2332 | conserved hypothetical protein | 2.1 |
|  | BT2334 | conserved hypothetical protein | 1.8 |
|  | BT2337 | conserved hypothetical protein | 1.7 |
|  | BT2339 | conserved hypothetical protein | 1.6 |
|  | BT2341 | conserved hypothetical protein | 1.3 |
|  | BT2358 | hypothetical protein | 1.9 |
|  | BT2368 | hypothetical protein | 4.8 |
|  | BT2375 | hypothetical protein | 1.8 |
|  | BT2422 | conserved hypothetical protein | 3.4 |
|  | BT2429 | hypothetical protein | 1.8 |
|  | BT2431 | conserved hypothetical protein | 1.3 |
|  | BT2443 | hypothetical protein | 1.8 |
|  | BT2448 | conserved hypothetical protein | 2.5 |
|  | BT2453 | hypothetical protein | 2.0 |
|  | BT2465 | conserved hypothetical protein | 1.7 |
|  | BT2467 | hypothetical protein | 1.6 |
|  | BT2468 | conserved hypothetical protein | 2.4 |
|  | BT2470 | hypothetical protein | 2.0 |
|  | BT2473 | hypothetical protein | 1.5 |
|  | BT2481 | hypothetical protein | 1.6 |
|  | BT2483 | hypothetical protein | 1.9 |
|  | BT2490 | hypothetical protein | 1.4 |
|  | BT2515 | hypothetical protein | 2.0 |
|  | BT2532 | SusC/D paralog | 2.2 |
|  | BT2533 | hypothetical protein | 2.3 |
|  | BT2555 | hypothetical protein | 1.8 |
|  | BT2579 | conserved hypothetical protein | 1.2 |
|  | BT2582 | conserved hypothetical protein | 5.4 |
|  | BT2583 | hypothetical protein | 1.9 |
|  | BT2585 | hypothetical protein | 2.0 |
|  | BT2592 | hypothetical protein | 1.9 |
|  | BT2595 | conserved protein found in conjugate transposon | 2.0 |
|  | BT2596 | conserved protein found in conjugate transposon | 2.2 |
|  | BT2597 | hypothetical protein | 1.5 |
|  | BT2598 | conserved protein found in conjugate transposon | 1.7 |
|  | BT2604 | conserved protein found in conjugate transposon | 1.7 |
|  | BT2607 | hypothetical protein | 1.5 |
|  | BT2612 | hypothetical protein | 2.0 |
|  | BT2613 | putative mobilization protein | 2.1 |
|  | BT2614 | putative mobilization protein | 2.2 |
|  | BT2637 | hypothetical protein | 1.8 |
|  | BT2641 | hypothetical protein | 2.3 |
|  | BT2646 | conserved hypothetical protein | 2.0 |
|  | BT2647 | hypothetical protein | 1.8 |
|  | BT2648 | hypothetical protein | 1.6 |
|  | BT2652 | conserved hypothetical protein | 1.8 |
|  | BT2655 | hypothetical protein | 2.1 |
|  | BT2657 | hypothetical protein | 1.8 |
|  | BT2677 | hypothetical protein | 2.1 |
|  | BT2678 | hypothetical protein | 4.4 |
|  | BT2695 | hypothetical protein | 1.8 |
|  | BT2732 | hypothetical protein | 1.5 |
|  | BT2759 | conserved hypothetical protein | 1.6 |
|  | BT2761 | hypothetical protein | 2.4 |
|  | BT2765 | hypothetical protein | 1.9 |
|  | BT2767 | hypothetical protein | 1.3 |
|  | BT2784 | hypothetical protein | 1.7 |
|  | BT2869 | putative succinyltransferase involved in succinoglycan biosynthesis | 1.5 |
|  | BT2870 | putative glycosyltransferase | 2.0 |
|  | BT2871 | putative glycosyltransferase | 1.6 |
|  | BT2884 | conserved hypothetical protein | 1.9 |
|  | BT2902 | hypothetical protein | 1.3 |
|  | BT2913 | unsaturated glucuronylhydrolase | 1.3 |
|  | BT2925 | hypothetical protein | 1.9 |
|  | BT2931 | hypothetical protein | 1.9 |
|  | BT2950 | conserved hypothetical protein | 1.4 |
|  | BT2961 | hypothetical protein | 2.0 |
|  | BT2962 | hypothetical protein | 2.0 |
|  | BT2964 | chondroitinase AC precursor | 1.7 |
|  | BT2965 | hypothetical protein | 1.4 |
|  | BT2974 | hypothetical protein | 1.4 |
|  | BT2982 | hypothetical protein | 1.5 |
|  | BT2988 | hypothetical protein | 1.7 |
|  | BT2989 | hypothetical protein | 2.0 |
|  | BT2991 | hypothetical protein | 2.1 |
|  | BT2993 | hypothetical protein | 2.0 |
|  | BT2994 | conserved hypothetical protein | 1.7 |
|  | BT2995 | conserved hypothetical protein with relaxase/mobilization nuclease domain | 1.5 |
|  | BT3015 | conserved hypothetical protein | 2.4 |
|  | BT3056 | hypothetical protein | 1.7 |
|  | BT3061 | conserved hypothetical protein | 2.9 |
|  | BT3079 | conserved hypothetical protein | 1.7 |
|  | BT3087 | cycloisomaltooligosaccharide glucanotransferase | 1.6 |
|  | BT3088 | hypothetical protein | 2.6 |
|  | BT3089 | SusC/D paralog | 1.7 |
|  | BT3120 | hypothetical protein | 1.3 |
|  | BT3138 | hypothetical protein | 1.5 |
|  | BT3142 | mobilization protein BmgB | 1.8 |
|  | BT3155 | conserved hypothetical protein | 1.8 |
|  | BT3163 | alpha-glucosidase, putative | 1.3 |
|  | BT3175 | SusC/D paralog | 2.5 |
|  | BT3194 | hypothetical protein | 2.3 |
|  | BT3203 | hypothetical protein | 1.5 |
|  | BT3208 | hypothetical protein | 1.5 |
|  | BT3273 | hypothetical protein | 1.4 |
|  | BT3275 | conserved hypothetical protein | 2.8 |
|  | BT3276 | conserved hypothetical protein | 1.7 |
|  | BT3295 | hypothetical protein | 1.2 |
|  | BT3296 | conserved hypothetical protein, putative outer membrane protein | 1.7 |
|  | BT3298 | conserved hypothetical protein, with a conserved domain | 1.9 |
|  | BT3315 | hypothetical protein | 3.1 |
|  | BT3321 | hypothetical protein | 1.5 |
|  | BT3377 | putative glycosyltransferase | 1.4 |
|  | BT3381 | hypothetical protein | 1.2 |
|  | BT3415 | hypothetical protein | 1.9 |
|  | BT3417 | hypothetical protein | 1.8 |
|  | BT3423 | hypothetical protein | 1.5 |
|  | BT3466 | hypothetical protein | 2.0 |
|  | BT3481 | putative glutaminase A | 1.5 |
|  | BT3494 | SusC/D paralog | 1.4 |
|  | BT3497 | conserved hypothetical protein | 2.2 |
|  | BT3498 | conserved hypothetical protein | 1.6 |
|  | BT3500 | hypothetical protein | 2.3 |
|  | BT3514 | conserved hypothetical protein | 1.4 |
|  | BT3519 | SusC/D paralog | 1.5 |
|  | BT3522 | hypothetical protein | 1.7 |
|  | BT3524 | alpha-1,6-mannanase | 2.0 |
|  | BT3541 | hypothetical protein | 1.8 |
|  | BT3542 | hypothetical protein | 2.3 |
|  | BT3544 | hypothetical protein | 1.8 |
|  | BT3546 | glutaminase | 1.9 |
|  | BT3547 | hypothetical protein | 1.4 |
|  | BT3553 | hypothetical protein | 1.7 |
|  | BT3564 | hypothetical protein | 1.8 |
|  | BT3571 | hypothetical protein | 1.9 |
|  | BT3575 | hypothetical protein | 1.4 |
|  | BT3595 | hypothetical protein | 1.4 |
|  | BT3602 | hypothetical protein | 2.0 |
|  | BT3624 | hypothetical protein | 1.5 |
|  | BT3625 | hypothetical protein | 2.0 |
|  | BT3629 | hypothetical protein | 1.6 |
|  | BT3630 | hypothetical protein | 2.8 |
|  | BT3661 | alpha-glucosidase | 1.2 |
|  | BT3676 | hypothetical protein | 1.4 |
|  | BT3686 | hypothetical protein | 2.0 |
|  | BT3721 | hypothetical protein | 1.6 |
|  | BT3740 | hypothetical protein | 1.8 |
|  | BT3741 | hypothetical protein | 1.6 |
|  | BT3751 | outer membrane protein | 1.3 |
|  | BT3794 | hypothetical protein | 1.8 |
|  | BT3822 | hypothetical protein | 1.5 |
|  | BT3859 | hypothetical protein | 2.0 |
|  | BT3874 | hypothetical protein | 1.8 |
|  | BT3880 | conserved hypothetical protein | 2.1 |
|  | BT3886 | hypothetical protein | 2.4 |
|  | BT3887 | hypothetical protein | 1.7 |
|  | BT3900 | hypothetical protein | 1.9 |
|  | BT3901 | hypothetical protein | 1.5 |
|  | BT3913 | hypothetical protein | 3.2 |
|  | BT3914 | hypothetical protein | 1.7 |
|  | BT3915 | hypothetical protein | 2.1 |
|  | BT3953 | conserved hypothetical protein, putative outer membrane protein | 2.0 |
|  | BT3960 | conserved hypothetical protein | 2.0 |
|  | BT3970 | hypothetical protein | 1.8 |
|  | BT4017 | hypothetical protein | 3.0 |
|  | BT4026 | conserved hypothetical protein | 3.2 |
|  | BT4029 | hypothetical protein | 2.9 |
|  | BT4034 | hypothetical protein | 3.1 |
|  | BT4036 | hypothetical protein | 4.4 |
|  | BT4071 | hypothetical protein | 1.2 |
|  | BT4076 | alpha-rhamnosidase | 1.4 |
|  | BT4085 | SusC/D paralog | 2.0 |
|  | BT4087 | hypothetical protein | 1.5 |
|  | BT4089 | SusC/D paralog | 1.6 |
|  | BT4132 | putative chitobiase | 1.7 |
|  | BT4134 | SusC/D paralog | 2.3 |
|  | BT4141 | hypothetical protein | 1.9 |
|  | BT4145 | conserved hypothetical protein | 1.9 |
|  | BT4161 | hypothetical protein | 2.1 |
|  | BT4172 | conserved hypothetical protein | 1.2 |
|  | BT4186 | hypothetical protein | 1.9 |
|  | BT4223 | hypothetical protein | 2.5 |
|  | BT4228 | hypothetical protein | 2.8 |
|  | BT4235 | hypothetical protein | 2.1 |
|  | BT4249 | hypothetical protein | 4.2 |
|  | BT4268 | SusC/D paralog | 1.4 |
|  | BT4270 | hypothetical protein | 1.5 |
|  | BT4287 | hypothetical protein | 1.5 |
|  | BT4361 | hypothetical protein | 1.6 |
|  | BT4365 | hypothetical protein | 1.7 |
|  | BT4384 | hypothetical protein | 8.6 |
|  | BT4431 | hypothetical protein | 1.6 |
|  | BT4433 | hypothetical protein | 2.0 |
|  | BT4438 | hypothetical protein | 2.2 |
|  | BT4440 | conserved hypothetical protein, putative cell surface protein | 1.4 |
|  | BT4441 | cell surface protein | 1.6 |
|  | BT4444 | hypothetical protein | 1.6 |
|  | BT4466 | hypothetical protein | 1.8 |
|  | BT4467 | hypothetical protein | 2.3 |
|  | BT4471 | hypothetical protein | 1.3 |
|  | BT4490 | hypothetical protein | 1.5 |
|  | BT4491 | hypothetical protein | 2.1 |
|  | BT4525 | hypothetical protein | 2.0 |
|  | BT4527 | hypothetical protein | 3.6 |
|  | BT4547 | excisionase | 1.7 |
|  | BT4548 | putative protein involved in transposition | 1.4 |
|  | BT4569 | hypothetical protein | 1.4 |
|  | BT4570 | hypothetical protein | 2.4 |
|  | BT4601 | hypothetical protein | 2.1 |
|  | BT4604 | hypothetical protein | 1.5 |
|  | BT4605 | conserved hypothetical protein | 1.9 |
|  | BT4619 | hypothetical protein | 1.8 |
|  | BT4620 | hypothetical protein | 2.3 |
|  | BT4623 | hypothetical protein | 1.6 |
|  | BT4658 | glucuronyl hydrolase | 1.3 |
|  | BT4659 | SusC/D paralog | 1.7 |
|  | BT4662 | heparinase III protein, heparitin sulfate lyase | 1.4 |
|  | BT4674 | hypothetical protein | 1.2 |
|  | BT4692 | hypothetical protein | 1.7 |
|  | BT4721 | hypothetical protein | 3.1 |
|  | BT4725 | SusC/D paralog | 1.4 |
|  | BT4729 | hypothetical protein | 4.9 |
|  | BT4742 | tetracycline resistance element regulator RteB | 1.5 |
|  | BT4743 | hypothetical protein | 1.7 |
|  | BT4746 | conserved hypothetical protein | 1.9 |
|  | BT4747 | hypothetical protein | 2.3 |
|  | BT4750 | hypothetical protein | 1.6 |
|  | BT4760 | hypothetical protein | 1.5 |
|  | BT4761 | conserved protein found in conjugate transposon | 2.7 |
|  | BT4764 | hypothetical protein | 1.9 |
|  | BT4765 | conserved hypothetical protein | 2.2 |
|  | BT4766 | hypothetical protein | 2.5 |
|  | BT4767 | conserved protein found in conjugate transposon | 2.1 |
|  | BT4768 | conserved protein found in conjugate transposon | 1.4 |
|  | BT4769 | conserved protein found in conjugate transposon | 2.1 |
|  | BT4771 | hypothetical protein | 1.4 |
|  | BT4772 | conserved protein found in conjugate transposon | 1.8 |
|  | BT4773 | conserved protein found in conjugate transposon | 2.1 |
|  | BT4775 | conserved protein found in conjugate transposon | 2.0 |
|  | BT4780 | conserved protein found in conjugate transposon | 1.3 |
