# Supplementary material for: Genomic and Metabolic Studies of the Impact of Probiotics on a Model Gut Symbiont and Host
Source: PLoS Biol. 2006 Nov 28;4(12):e413. doi: 10.1371/journal.pbio.0040413 (PMC1661682; doi:10.1371/journal.pbio.0040413)
Supplement: Supplementary file 17 [file pbio.0040413.st008.doc]

**Table S8.** COG functional group-based categorization of *B. thetaiotaomicron* genes with increased expression upon co-colonization with *B. longum*.

| **COG function** | **Gene** | **Description** | **Fold higher expression in co-colonization (vs. mono-association)** |
| --- | --- | --- | --- |
| Translation, ribosomal structure and biogenesis |  |  |  |
|  | BT0005 | 30S ribosomal protein S17 | 1.9 |
|  | BT0232 | putative protein involved in transposition | 1.5 |
|  | BT0423 | translation initiation factor IF-3 | 1.5 |
|  | BT0642 | RNA pseudouridylate synthase | 1.4 |
|  | BT0835 | tRNA-guanine transglycosylase | 1.4 |
|  | BT0865 | putative chitobiase | 3.0 |
|  | BT0913 | putative Fe-S oxidoreductase | 1.5 |
|  | BT0997 | hypothetical protein | 1.5 |
|  | BT1674 | hypothetical protein | 2.4 |
|  | BT2004 | RimM protein, required for 16S rRNA processing | 2.0 |
|  | BT2024 | conserved hypothetical protein | 3.8 |
|  | BT2729 | elongation factor G | 1.6 |
|  | BT2748 | glutamyl-tRNA synthetase | 1.9 |
|  | BT3326 | putative nifR3 family TIM-barrel enzyme with dihydrouridine synthase domain | 1.6 |
|  | BT3872 | ribosomal large subunit pseudouridine synthase B | 2.4 |
|  | BT3876 | 30S ribosomal protein S9 | 1.6 |
|  | BT3877 | 30S ribosomal protein S2 (BS1) | 1.6 |
|  | BT3888 | putative tRNA (5-methylaminomethyl-2-thiouridylate) methyltransferase | 1.3 |
|  | BT3943 | conserved hypothetical protein, putative translation factor | 1.3 |
|  | BT4237 | tRNA pseudouridine synthase A | 1.6 |
|  | BT4501 | ribosomal large subunit pseudouridine synthase A | 1.7 |
|  | BT4562 | hypothetical protein | 7.3 |
|  | BT4652 | conserved hypothetical protein | 3.0 |
|  |  |  |  |
| Transcription |  |  |  |
|  | BT0139 | RNA polymerase ECF-type sigma factor | 1.5 |
|  | BT0223 | two-component system response regulator | 2.0 |
|  | BT0376 | putative transcriptional regulator | 2.0 |
|  | BT0462 | putative transcriptional regulator | 7.3 |
|  | BT1133 | hypothetical protein | 5.0 |
|  | BT1187 | transcriptional regulator | 5.1 |
|  | BT1656 | putative transcriptional regulator | 5.4 |
|  | BT2308 | transcriptional regulator | 3.8 |
|  | BT2326 | putative DNA methylase | 2.6 |
|  | BT2386 | AsnC family transcriptional regulator | 1.7 |
|  | BT2509 | putative transcriptional regulator | 1.8 |
|  | BT2733 | DNA-directed RNA polymerase beta' chain | 2.5 |
|  | BT2886 | putative transcriptional regulator | 2.0 |
|  | BT2932 | transcriptional regulator | 1.5 |
|  | BT3613 | LacI family transcriptional regulator | 1.1 |
|  | BT3748 | RNA polymerase ECF-type sigma factor | 1.7 |
|  | BT3762 | arginine repressor, transcriptional regulator of arginine metabolism | 1.4 |
|  | BT3966 | two-component system response regulator | 1.3 |
|  | BT4199 | conserved hypothetical protein | 1.2 |
|  | BT4396 | transcriptional regulator | 2.2 |
|  | BT4477 | putative ATP-dependent DNA helicase | 2.4 |
|  | BT4749 | putative DNA methylase | 4.8 |
|  |  |  |  |
| Replication, recombination and repair |  |  |  |
|  | BT0025 | putative transposase | 2.0 |
|  | BT0034 | hypothetical protein | 5.1 |
|  | BT0091 | conserved protein found in conjugate transposon | 1.5 |
|  | BT0105 | DNA topoisomerase I (Omega-protein) | 1.4 |
|  | BT0252 | transcription-repair coupling factor | 1.7 |
|  | BT0570 | excinuclease ABC subunit B | 1.5 |
|  | BT0595 | integrase | 4.8 |
|  | BT0831 | ATP-dependent RNA helicase | 1.3 |
|  | BT0899 | DNA gyrase subunit A | 1.5 |
|  | BT1054 | ATP-dependent helicase | 2.0 |
|  | BT1057 | conserved hypothetical protein | 1.8 |
|  | BT1081 | recombination protein recR | 1.4 |
|  | BT1095 | conserved hypothetical protein | 7.6 |
|  | BT1361 | DNA repair protein recN (Recombination protein N) | 5.7 |
|  | BT1610 | DNA polymerase III subunit gamma/tau | 3.8 |
|  | BT1671 | endonuclease III | 2.8 |
|  | BT1928 | transposase | 48.0 |
|  | BT1929 | transposase | 1.7 |
|  | BT1932 | hypothetical protein | 2.3 |
|  | BT1960 | integrase | 4.2 |
|  | BT2021 | conserved hypothetical protein, putative non-specific DNA-binding protein | 1.9 |
|  | BT2267 | integrase protein | 1.6 |
|  | BT2277 | transposase | 2.2 |
|  | BT2446 | integrase | 5.2 |
|  | BT2469 | integrase | 1.7 |
|  | BT2602 | putative maturase/reverse transcriptase | 1.3 |
|  | BT2890 | transposase | 2.0 |
|  | BT2990 | hypothetical protein | 1.4 |
|  | BT2999 | helicase IV | 1.6 |
|  | BT3070 | Smf protein DNA processing chain A | 1.7 |
|  | BT3135 | integrase | 6.6 |
|  | BT3139 | conserved hypothetical protein | 3.2 |
|  | BT3255 | putative N6-adenine-specific DNA methylase | 1.6 |
|  | BT3266 | excinuclease ABC subunit C | 1.4 |
|  | BT3579 | topoisomerase IV subunit A | 1.2 |
|  | BT3641 | conserved hypothetical protein | 1.9 |
|  | BT3890 | putative exodeoxyribonuclease VII large subunit | 1.4 |
|  | BT3924 | ATP-dependent DNA helicase recG | 1.4 |
|  | BT3939 | single-stranded-DNA-specific exonuclease recJ | 1.2 |
|  | BT4224 | hypothetical protein | 4.1 |
|  | BT4344 | conserved hypothetical protein, putative DNA helicase | 5.2 |
|  | BT4544 | transposase | 2.3 |
|  |  |  |  |
| Cell cycle control, cell division, chromosome partitioning |  |  |  |
|  | BT0400 | putative tyrosine-protein kinase in capsular polysaccharide biosynthesis region | 3.3 |
|  | BT0841 | hypothetical protein | 1.7 |
|  | BT1676 | Maf protein | 1.3 |
|  | BT3813 | rod shape-determining protein MreB | 1.4 |
|  | BT4016 | hypothetical protein | 2.4 |
|  | BT4334 | FtsK/SpoIIIE family protein | 3.0 |
|  |  |  |  |
| Defense mechanisms |  |  |  |
|  | BT0299 | cation efflux system (AcrB/AcrD/AcrF family) | 1.9 |
|  | BT0300 | cation efflux system (AcrB/AcrD/AcrF family) | 4.1 |
|  | BT0301 | ATP-binding transport protein natA (Na+ ABC transporter) | 1.4 |
|  | BT0563 | putative ABC transporter ATP-binding protein or permease protein | 1.8 |
|  | BT0588 | BexA, multidrug efflux pump | 2.0 |
|  | BT0696 | putative membrane protein | 1.9 |
|  | BT0706 | putative N-acetylmuramoyl-L-alanine amidase | 220.4 |
|  | BT0859 | conserved hypothetical protein | 1.4 |
|  | BT0862 | putative ABC-transporter permease protein | 2.7 |
|  | BT0886 | putative cation efflux pump | 1.4 |
|  | BT1000 | conserved hypothetical protein | 1.3 |
|  | BT1254 | inner membrane protein | 1.9 |
|  | BT1387 | putative Na+-driven multidrug efflux pump | 2.8 |
|  | BT1519 | N-acetylmuramoyl-L-alanine amidase | 3.4 |
|  | BT2941 | multidrug resistance protein mexB (multidrug-efflux protein) | 2.6 |
|  | BT3386 | ABC transporter, ATP-binding protein | 1.4 |
|  | BT4288 | ABC transporter ATP-binding protein | 1.7 |
|  | BT4326 | ABC transporter ATP-binding protein | 1.4 |
|  |  |  |  |
| Signal transduction mechanisms |  |  |  |
|  | BT0059 | hypothetical protein | 74.9 |
|  | BT0537 | conserved hypothetical protein | 1.8 |
|  | BT0818 | two-component system sensor histidine kinase | 1.4 |
|  | BT0855 | two-component system sensor histidine kinase | 1.5 |
|  | BT1183 | Hybrid two-component system | 2.0 |
|  | BT1264 | two-component system sensor histidine kinase | 2.8 |
|  | BT1286 | putative two-component system response regulator | 3.7 |
|  | BT1545 | conserved hypothetical protein, putative regulatory protein with a cAMP-binding domain, | 2.0 |
|  | BT1598 | putative two-component system sensor histidine kinase | 1.9 |
|  | BT1635 | Hybrid two-component system | 3.2 |
|  | BT1738 | two-component system sensor histidine kinase | 1.6 |
|  | BT2618 | two-component system response regulator | 1.7 |
|  | BT2619 | two-component system sensor histidine kinase | 1.4 |
|  | BT2860 | Hybrid two-component system | 2.8 |
|  | BT2939 | putative transcriptional regulator | 2.4 |
|  | BT2971 | Hybrid two-component system | 2.3 |
|  | BT3465 | Hybrid two-component system | 1.4 |
|  | BT4111 | Hybrid two-component system | 1.3 |
|  | BT4124 | Hybrid two-component system | 1.6 |
|  | BT4182 | Hybrid two-component system | 3.3 |
|  | BT4354 | two-component system sensor histidine kinase | 1.6 |
|  | BT4567 | putative two-component system sensor but no kinase domain | 1.2 |
|  | BT4663 | two-component system sensor histidine kinase/response | 1.9 |
|  |  |  |  |
| Cell wall/membrane/envelope biogenesis |  |  |  |
|  | BT0053 | glycosyltransferase | 2.4 |
|  | BT0054 | putative glycosyltransferase | 3.4 |
|  | BT0055 | putative glycosyltransferase | 2.2 |
|  | BT0162 | penicillin-binding protein 1C (PBP-1c) | 1.9 |
|  | BT0294 | hypothetical protein | 10.0 |
|  | BT0297 | putative outer membrane lipoprotein silC precursor | 1.6 |
|  | BT0379 | UDP-glucose 6-dehydrogenase | 1.5 |
|  | BT0387 | NADH dehydrogenase subunit 2 | 3.6 |
|  | BT0554 | glucosamine--fructose-6-phosphate aminotransferase | 2.3 |
|  | BT0600 | nucleotide sugar epimerase | 1.8 |
|  | BT0678 | cation efflux system protein czcC | 4.6 |
|  | BT0743 | penicillin-binding protein 1A (PBP-1a) | 1.6 |
|  | BT0828 | dTDP-4-dehydrorhamnose 3,5-epimerase | 1.4 |
|  | BT0954 | conserved hypothetical protein | 1.3 |
|  | BT0955 | conserved hypothetical protein, putative membrane protein | 2.2 |
|  | BT1163 | conserved hypothetical protein | 1.2 |
|  | BT1164 | conserved hypothetical protein | 1.5 |
|  | BT1165 | hypothetical protein | 2.4 |
|  | BT1167 | conserved hypothetical protein | 2.1 |
|  | BT1176 | putative glycosyltransferase | 1.3 |
|  | BT1180 | putative glycosyltransferase | 1.5 |
|  | BT1224 | GDP-mannose 4,6-dehydratase | 1.3 |
|  | BT1250 | conserved hypothetical protein, putative outer membrane efflux protein | 1.4 |
|  | BT1269 | component of multidrug efflux system | 26.7 |
|  | BT1452 | conserved hypothetical protein | 1.6 |
|  | BT1472 | conserved hypothetical protein | 2.5 |
|  | BT1644 | putative CPS biosynthesis glycosyltransferase | 2.1 |
|  | BT1645 | putative glycosyltransferase | 3.3 |
|  | BT1646 | putative glycosyltransferase | 4.1 |
|  | BT1649 | conserved hypothetical protein | 4.9 |
|  | BT1650 | putative teichoic acid biosynthesis protein F | 5.4 |
|  | BT1654 | polysialic acid transport protein kpsD precursor | 3.9 |
|  | BT1708 | putative capsular polysaccharide biosynthesis glycosyltransferase | 5.2 |
|  | BT1967 | multidrug efflux protein | 1.7 |
|  | BT2001 | membrane-associated zinc metalloprotease | 4.5 |
|  | BT2152 | putative acetyltransferase | 2.2 |
|  | BT2442 | major outer membrane protein OmpA | 1.6 |
|  | BT2498 | putative outer membrane efflux protein | 3.0 |
|  | BT2588 | conserved hypothetical protein | 1.9 |
|  | BT2681 | alginate O-acetylation protein | 2.5 |
|  | BT2747 | 3-deoxy-D-manno-octulosonic-acid transferase | 3.2 |
|  | BT2795 | outer membrane efflux protein precursor | 4.1 |
|  | BT2838 | conserved hypothetical protein | 1.3 |
|  | BT2861 | putative outer membrane protein | 2.5 |
|  | BT2866 | putative glycosyltransferase | 1.6 |
|  | BT2873 | putative stress protein | 7.5 |
|  | BT2876 | putative glycosyltransferase | 1.5 |
|  | BT2880 | putative nucleotide-sugar dehydratase | 1.6 |
|  | BT2882 | glycosyltransferase | 2.1 |
|  | BT2942 | putative multidrug resistance protein | 1.6 |
|  | BT2945 | UDP-N-acetyl-D-mannosamine dehydrogenase | 3.4 |
|  | BT3026 | glycosylhydrolase, putative xylanase | 2.7 |
|  | BT3075 | NAD-dependent epimerase/dehydratase family protein | 1.3 |
|  | BT3202 | cell well associated RhsD protein precursor | 2.4 |
|  | BT3339 | AcrA/AcrE family multidrug resistance protein | 1.4 |
|  | BT3690 | putative membrane protein | 1.6 |
|  | BT3775 | conserved hypothetical protein | 2.2 |
|  | BT3776 | conserved hypothetical protein | 1.4 |
|  | BT3968 | cation efflux system protein | 2.3 |
|  | BT3996 | putative peptidase | 2.8 |
|  | BT4053 | putative membrane transport protein | 3.4 |
|  | BT4146 | exo-poly-alpha-D-galacturonosidase precursor | 1.6 |
|  | BT4155 | conserved hypothetical protein | 2.2 |
|  | BT4280 | putative glycosyltransferase | 1.5 |
|  | BT4281 | putative glycosyltransferase | 1.2 |
|  | BT4321 | 2-dehydro-3-deoxyphosphooctonate aldolase | 1.4 |
|  | BT4443 | N-acetylmuramoyl alanine amidase | 1.7 |
|  | BT4688 | conserved hypothetical protein | 1.3 |
|  |  |  |  |
| Cell motility |  |  |  |
|  | BT1127 | mobilization protein BmgB | 87.9 |
|  | BT1512 | conserved hypothetical protein, putative surface protein | 1.3 |
|  | BT1538 | hemagglutinin | 3.2 |
|  | BT2600 | conserved protein found in conjugate transposon | 3.2 |
|  |  |  |  |
| Intracellular trafficking, secretion, and vesicular transport |  |  |  |
|  | BT0102 | conserved hypothetical protein | 1.4 |
|  | BT0921 | conserved hypothetical protein | 1.3 |
|  | BT2298 | conserved protein found in conjugate transposon | 1.3 |
|  | BT2603 | conserved protein found in conjugate transposon | 1.6 |
|  | BT4103 | putative Sec-independent protein translocase | 1.3 |
|  | BT4325 | hypothetical protein | 1.5 |
|  | BT4770 | conserved protein found in conjugate transposon | 80.6 |
|  | BT4779 | conserved protein found in conjugate transposon | 268.2 |
|  |  |  |  |
| Posttranslational modification, protein turnover, chaperones |  |  |  |
|  | BT1055 | pyruvate formate-lyase activating enzyme | 1.7 |
|  | BT1244 | chaperone protein dnaJ | 5.4 |
|  | BT1292 | thiol:disulfide interchange protein | 1.3 |
|  | BT2295 | hypothetical protein | 2.0 |
|  | BT2577 | conserved hypothetical protein | 3.4 |
|  | BT2746 | thiol:disulfide interchange protein tlpA | 3.7 |
|  | BT2984 | hypothetical protein | 3.1 |
|  | BT2996 | hypothetical protein | 1.8 |
|  | BT3069 | putative disulphide-isomerase | 4.2 |
|  | BT3408 | conserved hypothetical protein, putative ABC transporter permease component | 1.6 |
|  | BT3810 | putative endothelin-converting enzyme | 1.4 |
|  | BT3841 | FKBP-type peptidyl-prolyl cis-transisomerase (trigger factor) | 1.7 |
|  | BT3846 | peptidyl-prolyl cis-trans isomerase | 2.0 |
|  | BT4007 | AAA-metalloprotease FtsH, with ATPase domain | 1.4 |
|  | BT4430 | cytochrome c biogenesis protein (ccsA) | 3.0 |
|  |  |  |  |
| Energy production and conversion |  |  |  |
|  | BT0123 | NADH-ubiquinone oxidoreductase subunit | 2.4 |
|  | BT0331 | ketoisovalerate oxidoreductase subunit vorB | 2.5 |
|  | BT0332 | 2-oxoisovalerate oxidoreductase beta subunit | 1.3 |
|  | BT0622 | Na+-transporting NADH:ubiquinone oxidoreductase, Electron transport complex protein rnfA | 2.1 |
|  | BT0640 | Fe-S oxidoreductases family 2 | 2.4 |
|  | BT0712 | ATP synthase epsilon chain | 1.6 |
|  | BT1113 | flavodoxin | 1.4 |
|  | BT1155 | Na+-translocating NADH-quinone reductase subunit | 2.1 |
|  | BT1207 | glycerate dehydrogenase (NADH-dependent) | 6.1 |
|  | BT1296 | V-type ATP synthase subunit I | 1.6 |
|  | BT1299 | V-type ATP synthase subunit A | 1.6 |
|  | BT1395 | conserved hypothetical protein | 2.2 |
|  | BT1451 | hypothetical protein | 1.5 |
|  | BT1594 | putative ferredoxin | 1.3 |
|  | BT1935 | hypothetical protein | 2.9 |
|  | BT2256 | fumarate hydratase class I, anaerobic | 1.4 |
|  | BT2318 | hypothetical protein | 3.5 |
|  | BT2346 | iron-sulfur cluster-binding protein | 9.2 |
|  | BT2444 | hypothetical protein | 1.5 |
|  | BT2790 | phosphoenolpyruvate carboxykinase [ATP] | 1.3 |
|  | BT2836 | 2-oxoglutarate synthase subunit korA | 1.7 |
|  | BT2837 | 2-oxoglutarate synthase subunit korB | 1.4 |
|  | BT2955 | formate acetyltransferase 2 | 2.4 |
|  | BT3055 | succinate dehydrogenase iron-sulfur protein | 1.4 |
|  | BT3245 | putative iron-sulfur cluster-binding protein | 2.2 |
|  | BT3252 | hypothetical protein | 13.2 |
|  | BT3582 | hypothetical protein | 2.3 |
|  | BT3614 | putative oxidoreductase | 1.5 |
|  | BT3767 | lactaldehyde reductase | 1.5 |
|  | BT3911 | malate dehydrogenase | 1.3 |
|  | BT4027 | hypothetical protein | 1.9 |
|  | BT4058 | NADH dehydrogenase I, chain N | 3.5 |
|  | BT4735 | hypothetical protein | 3.3 |
|  | BT4748 | hypothetical protein | 3.0 |
|  |  |  |  |
| Carbohydrate transport and metabolism |  |  |  |
|  | BT0144 | putative beta-xylosidase | 1.9 |
|  | BT0145 | putative beta-xylosidase | 3.7 |
|  | BT0196 | putative hexose phosphate transport protein | 1.9 |
|  | BT0258 | glucosamine-6-phosphate isomerase | 1.4 |
|  | BT0307 | phosphofructokinase | 1.8 |
|  | BT0437 | N-acylglucosamine 2-epimerase | 3.0 |
|  | BT0455 | sialidase (Neuraminidase) | 2.7 |
|  | BT0486 | altronate hydrolase | 1.5 |
|  | BT0675 | N-acetylglucosamine-6-phosphate deacetylase | 1.3 |
|  | BT0823 | uronate isomerase | 1.4 |
|  | BT0825 | altronate oxidoreductase | 1.5 |
|  | BT0983 | beta-galactosidase | 1.5 |
|  | BT0993 | beta-galactosidase | 3.8 |
|  | BT1001 | putative alpha-rhamnosidase | 3.1 |
|  | BT1273 | L-fucose isomerase | 4.7 |
|  | BT1397 | putative membrane protein | 3.8 |
|  | BT1522 | putative aureobasidin A resistance protein | 1.8 |
|  | BT1632 | chitinase | 3.9 |
|  | BT1663 | pullulanase precursor | 1.4 |
|  | BT1672 | phosphoglycerate kinase | 1.7 |
|  | BT1691 | fructose-bisphosphate aldolase | 1.3 |
|  | BT1776 | beta-galactosidase | 2.3 |
|  | BT1873 | endo-arabinase | 1.2 |
|  | BT1878 | conserved hypothetical protein | 1.2 |
|  | BT2112 | conserved hypothetical protein | 1.3 |
|  | BT2156 | putative sugar phosphate isomerase/epimerase | 3.2 |
|  | BT2199 | alpha-1,2-mannosidase | 3.6 |
|  | BT2402 | phosphoglycerate mutase | 1.7 |
|  | BT2567 | putative permease | 2.1 |
|  | BT2851 | alpha-galactosidase (melibiase) | 3.9 |
|  | BT2892 | conserved hypothetical protein | 2.8 |
|  | BT2895 | endo-1,4-beta-xylanase | 3.0 |
|  | BT2898 | endo-1,4-beta-xylanase D precursor | 1.9 |
|  | BT2949 | putative alpha-1,6-mannanase | 30.5 |
|  | BT3009 | beta-glucosidase (gentiobiase) | 1.5 |
|  | BT3086 | alpha-glucosidase II | 2.8 |
|  | BT3096 | alpha-L-arabinofuranosidase A precursor | 3.5 |
|  | BT3113 | putative transmembrane efflux protein | 9.5 |
|  | BT3114 | beta-galactosidase | 3.2 |
|  | BT3130 | conserved hypothetical protein | 1.8 |
|  | BT3132 | sialic acid-specific 9-O-acetylesterase | 1.4 |
|  | BT3198 | conserved hypothetical protein | 1.3 |
|  | BT3231 | 4-deoxy-L-threo-5-hexosulose-uronate ketol-isomerase | 5.2 |
|  | BT3290 | conserved hypothetical protein | 5.0 |
|  | BT3314 | thermostable beta-glucosidase B | 1.5 |
|  | BT3513 | beta-galactosidase | 1.8 |
|  | BT3516 | arabinan endo-1,5-alpha-L-arabinosidase A precursor | 1.6 |
|  | BT3530 | putative alpha-1,2-mannosidase | 4.9 |
|  | BT3532 | aldose 1-epimerase precursor | 2.2 |
|  | BT3589 | glucose/galactose transporter | 2.4 |
|  | BT3594 | conserved hypothetical protein | 2.0 |
|  | BT3656 | putative beta-xylosidase | 3.2 |
|  | BT3663 | putative beta-xylosidase | 1.6 |
|  | BT3764 | L-rhamnose isomerase | 1.7 |
|  | BT3773 | conserved hypothetical protein | 1.7 |
|  | BT3782 | putative hydrolase | 2.0 |
|  | BT3929 | triosephosphate isomerase | 1.5 |
|  | BT3956 | conserved hypothetical protein | 2.8 |
|  | BT3990 | putative alpha-1,2-mannosidase | 1.7 |
|  | BT4050 | beta-galactosidase | 1.4 |
|  | BT4073 | conserved hypothetical protein | 1.7 |
|  | BT4093 | conserved hypothetical protein | 1.5 |
|  | BT4095 | beta-xylosidase (1,4-beta-D-xylan xylosidase) | 3.7 |
|  | BT4110 | putative pectinesterase precursor | 1.6 |
|  | BT4119 | conserved hypothetical protein | 1.9 |
|  | BT4151 | beta-glucuronidase | 2.1 |
|  | BT4160 | beta-galactosidase precursor | 1.6 |
|  | BT4179 | polysaccharide deacetylase | 3.7 |
|  | BT4181 | beta-galactosidase | 6.8 |
|  | BT4263 | glyceraldehyde 3-phosphate dehydrogenase | 1.4 |
|  | BT4311 | glucose/galactose transporter | 1.4 |
|  | BT4379 | putative oxalate:formate antiporter | 2.0 |
|  | BT4394 | beta-hexosaminidase precursor | 9.5 |
|  | BT4397 | xylose/H+ symporter | 1.3 |
|  | BT4667 | beta-galactosidase | 1.9 |
|  | BT4668 | arabinogalactan endo-1,4-beta-galactosidase | 1.7 |
|  | BT4714 | periplasmic beta-glucosidase precursor, xylosidase/arabinosidase | 5.4 |
|  |  |  |  |
| Amino acid transport and metabolism |  |  |  |
|  | BT0201 | histidinol dehydrogenase | 1.4 |
|  | BT0202 | histidinol-phosphate aminotransferase | 1.6 |
|  | BT0203 | histidine biosynthesis bifunctional protein hisB | 1.4 |
|  | BT0225 | hypothetical protein | 5.8 |
|  | BT0414 | sulfate adenylyltransferase subunit 2 | 2.0 |
|  | BT0457 | sialic acid-specific 9-O-acetylesterase | 3.0 |
|  | BT0528 | putative N-(5'-phosphoribosyl)anthranilate isomerase | 2.2 |
|  | BT0530 | anthranilate phosphoribosyltransferase | 3.1 |
|  | BT0531 | anthranilate synthase component II | 2.7 |
|  | BT0532 | anthranilate synthase component I | 1.4 |
|  | BT0533 | tryptophan synthase beta chain | 1.8 |
|  | BT0547 | aspartate aminotransferase | 4.5 |
|  | BT0556 | carbamoyl phosphate synthetase III (glutamine-hydrolyzing) | 1.7 |
|  | BT0587 | prolyl oligopeptidase family protein | 1.1 |
|  | BT0674 | carboxynorspermidine decarboxylase | 1.6 |
|  | BT0738 | serine hydroxymethyltransferase | 1.5 |
|  | BT0832 | putative phosphoserine phosphatase | 5.7 |
|  | BT0878 | ABC transporter, ATP-binding protein | 8.7 |
|  | BT0895 | dihydrodipicolinate synthase | 1.4 |
|  | BT1099 | putative arginase | 1.8 |
|  | BT1147 | glycine dehydrogenase [decarboxylating] | 1.6 |
|  | BT1261 | peptidase | 3.0 |
|  | BT1291 | spermidine/putrescine transport ATP-binding protein | 11.2 |
|  | BT1398 | putative aminotransferase B | 1.9 |
|  | BT1732 | conserved hypothetical protein | 1.5 |
|  | BT1749 | glycine betaine-binding protein precursor | 4.5 |
|  | BT1750 | glycine betaine/L-proline transport system permease | 1.5 |
|  | BT1789 | aminopeptidase C (bleomycin hydrolase) | 11.7 |
|  | BT1857 | 3-isopropylmalate dehydrogenase | 1.9 |
|  | BT1858 | 2-isopropylmalate synthase | 1.5 |
|  | BT1860 | 3-isopropylmalate dehydratase large subunit | 2.6 |
|  | BT1901 | lysine-sensitive aspartokinase III | 9.8 |
|  | BT2077 | acetolactate synthase large subunit | 14.0 |
|  | BT2078 | dihydroxy-acid dehydratase | 1.7 |
|  | BT2186 | 3-phosphoshikimate 1-carboxyvinyltransferase | 2.5 |
|  | BT2401 | threonine synthase | 2.7 |
|  | BT2403 | aspartokinase/homoserine dehydrogenase | 3.0 |
|  | BT2413 | homoserine O-succinyltransferase | 1.9 |
|  | BT2814 | dihydrodipicolinate synthase | 1.9 |
|  | BT2875 | serine O-acetyltransferase | 1.5 |
|  | BT3080 | cysteine synthase A | 1.4 |
|  | BT3256 | serine acetyltransferase | 3.2 |
|  | BT3394 | putative arginine decarboxylase | 2.4 |
|  | BT3395 | putative acetylglutamate kinase | 2.4 |
|  | BT3617 | sorbitol dehydrogenase | 1.7 |
|  | BT3681 | conserved hypothetical protein, putative outer membrane protein | 2.0 |
|  | BT3718 | gamma-glutamyl phosphate reductase | 1.6 |
|  | BT3720 | putative aspartate aminotransferase | 2.9 |
|  | BT3758 | acetylornithine aminotransferase | 1.7 |
|  | BT3760 | argininosuccinate synthase | 1.8 |
|  | BT3821 | 5,10-methylenetetrahydrofolate reductase | 1.3 |
|  | BT3866 | glutamine-dependent carbamyl phosphate synthetase | 1.8 |
|  | BT3933 | chorismate mutase/prephenate dehydratase (tyrA) | 2.5 |
|  | BT3935 | aminotransferase | 3.2 |
|  | BT3936 | prephenate dehydratase | 4.5 |
|  | BT4257 | putative histidinol-phosphatase | 3.1 |
|  | BT4476 | amino acid carrier protein | 19.6 |
|  | BT4546 | conserved hypothetical protein | 7.5 |
|  | BT4612 | conserved hypothetical protein | 1.2 |
|  | BT4751 | hypothetical protein | 2.2 |
|  |  |  |  |
| Nucleotide transport and metabolism |  |  |  |
|  | BT0184 | uridine kinase (uridine monophosphokinase) | 1.5 |
|  | BT0250 | dihydroorotase (DHOase) | 3.8 |
|  | BT0354 | conserved hypothetical protein | 1.6 |
|  | BT0748 | ribose-phosphate pyrophosphokinase | 1.6 |
|  | BT1236 | 2',3'-cyclic-nucleotide 2'-phosphodiesterase precursor | 2.7 |
|  | BT1381 | formyltetrahydrofolate deformylase | 1.5 |
|  | BT2279 | putative ribose phosphate pyrophosphokinase | 3.9 |
|  | BT3128 | putative xanthosine triphosphate pyrophosphatase | 1.5 |
|  | BT3253 | phosphoribosylamine--glycine ligase | 1.7 |
|  | BT4265 | GMP synthase (glutamine-hydrolyzing) | 3.7 |
|  | BT4386 | hypoxanthine-guanine phosphoribosyltransferase | 1.7 |
|  |  |  |  |
| Coenzyme transport and metabolism |  |  |  |
|  | BT0068 | hypothetical protein | 3.0 |
|  | BT0648 | molybdopterin biosynthesis protein | 1.4 |
|  | BT0649 | thiamine biosynthesis protein ThiH | 5.3 |
|  | BT0698 | 3-methyl-2-oxobutanoate hydroxymethyltransferase | 3.8 |
|  | BT0790 | phosphomethylpyrimidine kinase | 1.4 |
|  | BT0870 | 8-amino-7-oxononanoate synthase | 2.1 |
|  | BT1446 | dethiobiotin synthetase | 1.7 |
|  | BT1474 | ribosomal protein S6 modification protein-related protein | 1.7 |
|  | BT1607 | methylenetetrahydrofolate dehydrogenase/methenyltetrahydrofolate cyclohydrolase | 2.2 |
|  | BT1836 | thiamine biosynthesis protein ThiH | 1.8 |
|  | BT2416 | GTP cyclohydrolase II | 1.5 |
|  | BT2543 | riboflavin biosynthesis protein ribF, putative riboflavin kinase | 1.4 |
|  | BT2797 | adenosylhomocysteinase | 1.3 |
|  | BT3216 | 2-amino-4-hydroxy-6- hydroxymethyldihydropteridine | 2.4 |
|  | BT3219 | S-adenosylmethionine synthetase | 1.4 |
|  | BT3468 | conserved hypothetical protein | 1.5 |
|  | BT4253 | 6,7-dimethyl-8-ribityllumazine synthase | 2.7 |
|  | BT4458 | pyridoxine kinase | 2.0 |
|  | BT4702 | dihydroxynapthoic acid synthetase | 2.1 |
|  |  |  |  |
| Lipid transport and metabolism |  |  |  |
|  | BT1523 | CDP-diacylglycerol-inositol 3-phosphatidyltransferase | 1.9 |
|  | BT1546 | acyltransferase | 1.8 |
|  | BT1651 | pyrophosphorylase | 3.4 |
|  | BT3550 | putative long-chain-fatty-acid--CoA ligase | 1.3 |
|  | BT3834 | 3-oxoacyl-[acyl-carrier-protein] synthase III | 1.4 |
|  | BT3978 | putative cardiolipin synthetase | 2.2 |
|  |  |  |  |
| Inorganic ion transport and metabolism |  |  |  |
|  | BT0029 | SusC/D paralog | 1.5 |
|  | BT0190 | SusC/D paralog | 1.5 |
|  | BT0206 | SusC/D paralog | 1.8 |
|  | BT0272 | SusC/D paralog | 5.2 |
|  | BT0289 | conserved hypothetical protein | 1.4 |
|  | BT0412 | putative Na+/sulfate symporter | 1.3 |
|  | BT0415 | sulfate adenylyltransferase subunit 1/adenylylsulfate kinase | 3.1 |
|  | BT0504 | TonB-dependent outer membrane receptor | 2.0 |
|  | BT0544 | ammonium transporter | 2149.1 |
|  | BT0680 | cation efflux system protein czcA | 2.5 |
|  | BT0756 | iduronate 2-sulfatase precursor | 20.8 |
|  | BT0867 | SusC/D paralog | 4.7 |
|  | BT1025 | SusC/D paralog | 2.0 |
|  | BT1093 | conserved hypothetical protein, putative TonB-dependent outer membrane receptor protein | 5.9 |
|  | BT1323 | putative ABC transporter permease protein | 1.6 |
|  | BT1388 | integral membrane protein | 1.2 |
|  | BT1440 | SusC/D paralog | 1.7 |
|  | BT1606 | cytochrome C peroxidase | 5.0 |
|  | BT1628 | putative sulfatase yidJ | 4.9 |
|  | BT1844 | transcriptional regulator, putative iron uptake regulation protein | 1.2 |
|  | BT1951 | Fe3+ ABC transporter, permease protein | 1.3 |
|  | BT2409 | putative TonB-dependent outer membrane protein | 5.8 |
|  | BT2425 | K+-transporting ATPase A chain | 1.4 |
|  | BT2512 | cation-transporting ATPase, P-type, putative zinc-transporting ATPase | 3.8 |
|  | BT2820 | SusC/D paralog | 1.9 |
|  | BT2894 | SusC/D paralog | 2.7 |
|  | BT2920 | SusC/D paralog | 1.6 |
|  | BT3016 | TonB-dependent receptor | 1.9 |
|  | BT3051 | mucin-desulfating sulfatase | 1.8 |
|  | BT3060 | hypothetical protein | 2.3 |
|  | BT3095 | putative sulfatase | 10.2 |
|  | BT3107 | arylsulfatase (aryl-sulfate sulphohydrolase) | 1.7 |
|  | BT3109 | arylsulfatase A precursor | 2.5 |
|  | BT3174 | SusC/D paralog | 2.5 |
|  | BT3249 | putative zinc ABC transporter, zinc-binding protein | 1.5 |
|  | BT3270 | putative anti-sigma factor | 2.0 |
|  | BT3310 | SusC/D paralog | 2.0 |
|  | BT3333 | arylsulfatase A precursor (ASA) | 2.3 |
|  | BT3475 | SusC/D paralog | 1.2 |
|  | BT3483 | SusC/D paralog | 2.6 |
|  | BT3487 | arylsulfatase (aryl-sulfate sulphohydrolase) | 1.3 |
|  | BT3633 | SusC/D paralog | 2.5 |
|  | BT3670 | SusC/D paralog | 1.4 |
|  | BT3702 | SusC, outer membrane protein involved in starch binding | 1.7 |
|  | BT3952 | SusC/D paralog | 3.6 |
|  | BT3958 | SusC/D paralog | 1.4 |
|  | BT3983 | SusC/D paralog | 1.7 |
|  | BT4097 | Trk system K+ uptake protein trkH | 5.0 |
|  | BT4098 | Trk system K+ uptake protein trkA | 11.7 |
|  | BT4121 | SusC/D paralog | 2.1 |
|  | BT4416 | Cu2+ homeostasis protein CutC | 1.4 |
|  | BT4486 | voltage-gated K+ channel protein | 1.6 |
|  | BT4568 | conserved hypothetical protein | 1.6 |
|  | BT4585 | putative Na+/H+ anitporter | 1.4 |
|  | BT4671 | SusC/D paralog | 1.4 |
|  | BT4724 | SusC/D paralog | 2.1 |
|  |  |  |  |
| Secondary metabolites biosynthesis, transport and catabolism |  |  |  |
|  | BT2525 | cephalosporin-C deacetylase | 2.0 |
|  | BT3343 | putative phenylacetic acid degradation protein | 9.2 |
|  |  |  |  |
| General function prediction only |  |  |  |
|  | BT0057 | hypothetical protein | 4.1 |
|  | BT0148 | putative hemolysin | 1.8 |
|  | BT0194 | hypothetical protein | 1.2 |
|  | BT0264 | conserved hypothetical protein | 3.6 |
|  | BT0278 | conserved hypothetical protein | 1.3 |
|  | BT0388 | galactoside O-acetyltransferase | 1.7 |
|  | BT0409 | putative short-chain dehydrogenase | 1.2 |
|  | BT0521 | putative acetyl transferase | 3.1 |
|  | BT0543 | glutamine synthetase | 2.8 |
|  | BT0740 | flavoredoxin | 1.7 |
|  | BT0746 | putative zinc protease | 1.4 |
|  | BT0838 | putative RNA methyltransferase | 2.1 |
|  | BT1146 | putative metallo-beta-lactamase | 1.8 |
|  | BT1166 | putative glycosyltransferase | 2.0 |
|  | BT1177 | putative polysaccharide biosynthesis protein | 1.6 |
|  | BT1186 | ABC transporter ATP-binding protein | 2.2 |
|  | BT1384 | conserved hypothetical protein | 1.5 |
|  | BT1402 | iron-sulfur flavoprotein | 3.1 |
|  | BT1463 | putative acetyltransferase | 1.5 |
|  | BT1473 | conserved hypothetical protein | 2.9 |
|  | BT1479 | ABC transporter ATP-binding protein | 1.4 |
|  | BT1496 | hemolysin-related protein, containing CBS domain | 3.5 |
|  | BT1652 | lipopolysaccharide biosynthesis protein | 3.9 |
|  | BT1713 | putative acylneuraminate cytidylyltransferase | 13.0 |
|  | BT1837 | putative GTP-binding protein, putative GTPase | 1.4 |
|  | BT2013 | putative phosphohydrolases | 2.1 |
|  | BT2092 | putative membrane protein | 5.3 |
|  | BT2127 | putative beta-phosphoglucomutase | 3.5 |
|  | BT2128 | conserved hypothetical protein | 2.4 |
|  | BT2159 | putative oxidoreductase | 2.3 |
|  | BT2271 | haloacid dehalogenase-like hydrolase | 1.2 |
|  | BT2547 | conserved hypothetical protein | 1.5 |
|  | BT2877 | putative acetyltransferase | 2.2 |
|  | BT2912 | putative glycosyslhydrolase | 1.6 |
|  | BT2934 | putative transmembrane transport protein, putative lipopolysaccharide biosynthesis protein | 3.1 |
|  | BT3111 | hypothetical protein | 4.7 |
|  | BT3137 | conserved hypothetical protein | 2.3 |
|  | BT3283 | conserved hypothetical protein with conserved domain | 1.4 |
|  | BT3391 | ribonuclease H-related protein | 4.0 |
|  | BT3424 | hypothetical protein | 1.7 |
|  | BT3442 | conserved hypothetical protein | 1.5 |
|  | BT3462 | TPR domain protein | 1.9 |
|  | BT3508 | hypothetical protein | 3.0 |
|  | BT3551 | putative zinc protease | 1.7 |
|  | BT3570 | TPR-repeat-containing proteins | 2.4 |
|  | BT3642 | Na+-dependent transporter | 1.4 |
|  | BT3836 | putative phosphoglycerate dehydrogenase | 1.3 |
|  | BT3840 | putative RNA-binding protein rbpA | 1.7 |
|  | BT3885 | putative phosphohydrolase, Icc family | 1.2 |
|  | BT4131 | conserved hypothetical protein | 1.9 |
|  | BT4230 | conserved hypothetical protein | 5.0 |
|  | BT4292 | conserved hypothetical protein | 3.9 |
|  | BT4324 | conserved hypothetical protein | 2.5 |
|  | BT4370 | putative hemolysin | 1.6 |
|  |  |  |  |
| Function unknown |  |  |  |
|  | BT0210 | leucine-rich repeat protein, function unknown | 1.6 |
|  | BT0493 | hypothetical protein | 7.3 |
|  | BT0579 | putative transcription regulator | 36.3 |
|  | BT0637 | conserved hypothetical protein | 1.4 |
|  | BT0761 | conserved hypothetical protein | 3.3 |
|  | BT0778 | conserved hypothetical protein | 3.5 |
|  | BT0874 | conserved hypothetical protein | 5.2 |
|  | BT1003 | conserved hypothetical protein | 1.6 |
|  | BT1327 | putative alkaline phosphatase | 1.5 |
|  | BT1408 | putative Tricorn-like protease | 1.2 |
|  | BT1444 | conserved hypothetical protein | 7.0 |
|  | BT1521 | conserved hypothetical protein | 2.4 |
|  | BT1541 | conserved hypothetical protein | 4.3 |
|  | BT1949 | conserved hypothetical protein | 20.0 |
|  | BT2255 | conserved hypothetical protein | 2.5 |
|  | BT2530 | conserved hypothetical protein, with a conserved domain | 4.0 |
|  | BT2632 | conserved hypothetical protein | 1.8 |
|  | BT2848 | hypothetical protein | 2.2 |
|  | BT2896 | conserved hypothetical protein | 5.6 |
|  | BT3066 | conserved hypothetical protein | 1.2 |
|  | BT3127 | conserved hypothetical protein | 2.2 |
|  | BT3215 | conserved hypothetical protein | 1.8 |
|  | BT3250 | conserved hypothetical protein | 3.2 |
|  | BT3531 | conserved hypothetical protein | 3.2 |
|  | BT3647 | conserved hypothetical protein | 1.5 |
|  | BT3669 | conserved hypothetical protein | 1.3 |
|  | BT3674 | conserved hypothetical protein | 2.8 |
|  | BT3679 | conserved hypothetical protein, with a conserved domain | 1.8 |
|  | BT3819 | conserved hypothetical protein | 3.0 |
|  | BT4266 | conserved hypothetical protein | 7.7 |
|  | BT4390 | conserved hypothetical protein | 2.3 |
|  | BT4450 | conserved hypothetical protein | 1.3 |
|  | BT4559 | conserved hypothetical protein | 2.2 |
|  |  |  |  |
| Not in COG |  |  |  |
|  | BT0013 | hypothetical protein | 1.8 |
|  | BT0018 | hypothetical protein | 2.0 |
|  | BT0032 | hypothetical protein | 1.8 |
|  | BT0052 | hypothetical protein | 6.1 |
|  | BT0058 | hypothetical protein | 6.6 |
|  | BT0063 | hypothetical protein | 1.4 |
|  | BT0065 | alpha-galactosidase precursor | 2.2 |
|  | BT0085 | conserved protein found in conjugate transposon | 2.8 |
|  | BT0086 | conserved protein found in conjugate transposon | 3.0 |
|  | BT0092 | conserved protein found in conjugate transposon | 10.8 |
|  | BT0099 | hypothetical protein | 5.3 |
|  | BT0103 | conserved hypothetical protein | 2.3 |
|  | BT0110 | putative regulatory protein | 5.1 |
|  | BT0127 | putative transmembrane protein | 1.3 |
|  | BT0141 | SusC/D paralog | 1.6 |
|  | BT0157 | hypothetical protein | 1.2 |
|  | BT0191 | SusC/D paralog | 8.4 |
|  | BT0198 | hypothetical protein | 3.9 |
|  | BT0207 | SusC/D paralog | 2.8 |
|  | BT0213 | hypothetical protein | 1.4 |
|  | BT0214 | hypothetical protein | 2.2 |
|  | BT0236 | conserved hypothetical protein | 1.4 |
|  | BT0273 | hypothetical protein | 2.3 |
|  | BT0276 | hypothetical protein | 3.0 |
|  | BT0277 | conserved hypothetical protein | 2.5 |
|  | BT0279 | hypothetical protein | 2.1 |
|  | BT0292 | conserved hypothetical protein | 3.8 |
|  | BT0293 | hypothetical protein | 7.8 |
|  | BT0361 | hypothetical protein | 1.3 |
|  | BT0490 | hypothetical protein | 1.9 |
|  | BT0501 | hypothetical protein | 2.5 |
|  | BT0663 | hypothetical protein | 1.7 |
|  | BT0664 | hypothetical protein | 2.3 |
|  | BT0709 | hypothetical protein | 7.3 |
|  | BT0710 | hypothetical protein | 3.5 |
|  | BT0739 | hypothetical protein | 1.6 |
|  | BT0866 | SusC/D paralog | 4.1 |
|  | BT0939 | hypothetical protein | 3.4 |
|  | BT0941 | hypothetical protein | 1.3 |
|  | BT0946 | hypothetical protein | 1.6 |
|  | BT0950 | hypothetical protein | 1.3 |
|  | BT0979 | hypothetical protein | 1.4 |
|  | BT0980 | conserved hypothetical protein | 5.8 |
|  | BT1024 | SusC/D paralog | 9.4 |
|  | BT1065 | hypothetical protein | 4.6 |
|  | BT1066 | hypothetical protein | 1.9 |
|  | BT1067 | hypothetical protein | 57.4 |
|  | BT1136 | hypothetical protein | 3.1 |
|  | BT1173 | hypothetical protein | 1.9 |
|  | BT1175 | hypothetical protein | 1.6 |
|  | BT1184 | conserved hypothetical protein | 4.6 |
|  | BT1302 | hypothetical protein | 1.3 |
|  | BT1303 | hypothetical protein | 1.8 |
|  | BT1490 | conserved hypothetical protein, putative surface protein | 4.3 |
|  | BT1507 | hypothetical protein | 2.1 |
|  | BT1552 | SusC/D paralog | 3.9 |
|  | BT1565 | hypothetical protein | 2.6 |
|  | BT1611 | hypothetical protein | 1.6 |
|  | BT1637 | hypothetical protein | 2.0 |
|  | BT1640 | hypothetical protein | 6.9 |
|  | BT1702 | hypothetical protein | 5.4 |
|  | BT1752 | hypothetical protein | 3.4 |
|  | BT1761 | hypothetical protein | 3.7 |
|  | BT1771 | putative cell surface protein | 3.9 |
|  | BT1775 | hypothetical protein | 3.0 |
|  | BT1803 | conserved hypothetical protein | 1.9 |
|  | BT1895 | hypothetical protein | 1.5 |
|  | BT1909 | hypothetical protein | 7.2 |
|  | BT1926 | hypothetical protein | 6.3 |
|  | BT1927 | hypothetical protein | 4.3 |
|  | BT1934 | hypothetical protein | 1.9 |
|  | BT1936 | hypothetical protein | 2.5 |
|  | BT1938 | hypothetical protein | 1.4 |
|  | BT1944 | putative transposase | 3.0 |
|  | BT1947 | hypothetical protein | 9.6 |
|  | BT1987 | hypothetical protein | 29.8 |
|  | BT1988 | hypothetical protein | 4.3 |
|  | BT2025 | hypothetical protein | 5.1 |
|  | BT2026 | endonuclease | 3.5 |
|  | BT2029 | conserved hypothetical protein | 2.2 |
|  | BT2033 | hypothetical protein | 1.8 |
|  | BT2045 | hypothetical protein | 1.4 |
|  | BT2057 | hypothetical protein | 3.9 |
|  | BT2108 | hypothetical protein | 1.6 |
|  | BT2131 | hypothetical protein | 1.6 |
|  | BT2157 | conserved hypothetical protein | 1.7 |
|  | BT2194 | sialidase precursor, exo-alpha-sialidase | 6.4 |
|  | BT2201 | SusC/D paralog | 2.9 |
|  | BT2203 | conserved hypothetical protein | 2.3 |
|  | BT2217 | hypothetical protein | 1.3 |
|  | BT2227 | conserved hypothetical protein | 2.6 |
|  | BT2254 | putative pectate lyase | 1.6 |
|  | BT2288 | conserved protein found in conjugate transposon | 39.7 |
|  | BT2293 | conserved protein found in conjugate transposon | 3.4 |
|  | BT2305 | putative mobilization protein | 1.9 |
|  | BT2307 | tetracycline resistance element mobilization regulatory protein rteC | 8.4 |
|  | BT2325 | hypothetical protein | 336.4 |
|  | BT2329 | conserved hypothetical protein | 1.5 |
|  | BT2332 | conserved hypothetical protein | 2.7 |
|  | BT2363 | SusC/D paralog | 2.8 |
|  | BT2429 | hypothetical protein | 1.3 |
|  | BT2464 | hypothetical protein | 1.5 |
|  | BT2477 | conserved hypothetical protein | 2.1 |
|  | BT2524 | alpha-rhamnosidase | 2.9 |
|  | BT2536 | hypothetical protein | 11.3 |
|  | BT2575 | hypothetical protein | 6.9 |
|  | BT2581 | hypothetical protein | 3.6 |
|  | BT2584 | hypothetical protein | 21.8 |
|  | BT2592 | hypothetical protein | 5.7 |
|  | BT2595 | conserved protein found in conjugate transposon | 3.4 |
|  | BT2604 | conserved protein found in conjugate transposon | 1.8 |
|  | BT2614 | putative mobilization protein | 3.8 |
|  | BT2620 | alpha-glucosidase | 1.5 |
|  | BT2638 | hypothetical protein | 2.9 |
|  | BT2648 | hypothetical protein | 2.5 |
|  | BT2684 | hypothetical protein | 6.0 |
|  | BT2758 | hypothetical protein | 1.3 |
|  | BT2767 | hypothetical protein | 1.4 |
|  | BT2785 | hypothetical protein | 2.2 |
|  | BT2858 | SusC/D paralog | 3.1 |
|  | BT2871 | putative glycosyltransferase | 1.7 |
|  | BT2893 | hypothetical protein | 3.5 |
|  | BT2899 | hypothetical protein | 2.2 |
|  | BT2913 | unsaturated glucuronylhydrolase | 1.4 |
|  | BT2960 | hypothetical protein | 5.1 |
|  | BT2967 | SusC/D paralog | 7.0 |
|  | BT2974 | hypothetical protein | 4.4 |
|  | BT2991 | hypothetical protein | 1.5 |
|  | BT3005 | hypothetical protein | 2.1 |
|  | BT3056 | hypothetical protein | 3.0 |
|  | BT3087 | cycloisomaltooligosaccharide glucanotransferase | 2.0 |
|  | BT3089 | SusC/D paralog | 3.0 |
|  | BT3102 | SusC/D paralog | 3.1 |
|  | BT3163 | alpha-glucosidase, putative | 1.5 |
|  | BT3175 | SusC/D paralog | 50.4 |
|  | BT3194 | hypothetical protein | 3.9 |
|  | BT3241 | hypothetical protein | 2.2 |
|  | BT3243 | hypothetical protein | 1.5 |
|  | BT3282 | conserved hypothetical protein | 2.5 |
|  | BT3295 | hypothetical protein | 1.5 |
|  | BT3298 | conserved hypothetical protein, with a conserved domain | 1.7 |
|  | BT3311 | SusC/D paralog | 3.3 |
|  | BT3313 | hypothetical protein | 2.5 |
|  | BT3321 | hypothetical protein | 1.6 |
|  | BT3330 | hypothetical protein | 2.6 |
|  | BT3331 | SusC/D paralog | 1.9 |
|  | BT3344 | hypothetical protein | 1.4 |
|  | BT3363 | lipopolysaccharide core biosynthesis protein LpsA | 1.3 |
|  | BT3380 | putative membrane protein | 2.2 |
|  | BT3423 | hypothetical protein | 4.5 |
|  | BT3427 | conserved hypothetical protein | 1.3 |
|  | BT3474 | SusC/D paralog | 9.3 |
|  | BT3494 | SusC/D paralog | 1.4 |
|  | BT3496 | hypothetical protein | 8.8 |
|  | BT3510 | hypothetical protein | 2.1 |
|  | BT3542 | hypothetical protein | 4.1 |
|  | BT3553 | hypothetical protein | 1.9 |
|  | BT3624 | hypothetical protein | 1.5 |
|  | BT3625 | hypothetical protein | 2.2 |
|  | BT3629 | hypothetical protein | 2.0 |
|  | BT3664 | putative alpha-glucosidase | 26.9 |
|  | BT3668 | hypothetical protein | 2.5 |
|  | BT3671 | SusC/D paralog | 1.5 |
|  | BT3676 | hypothetical protein | 1.6 |
|  | BT3686 | hypothetical protein | 1.8 |
|  | BT3699 | outer membrane protein SusF | 1.7 |
|  | BT3714 | conserved hypothetical protein | 1.8 |
|  | BT3741 | hypothetical protein | 1.9 |
|  | BT3752 | hypothetical protein | 2.2 |
|  | BT3789 | hypothetical protein | 1.3 |
|  | BT3794 | hypothetical protein | 2.8 |
|  | BT3818 | conserved hypothetical protein | 1.4 |
|  | BT3867 | hypothetical protein | 1.6 |
|  | BT3880 | conserved hypothetical protein | 2.3 |
|  | BT3881 | hypothetical protein | 8.4 |
|  | BT3909 | conserved hypothetical protein | 11.1 |
|  | BT3915 | hypothetical protein | 1.6 |
|  | BT3959 | conserved hypothetical protein, putative outer membrane protein | 2.0 |
|  | BT4017 | hypothetical protein | 6.1 |
|  | BT4026 | conserved hypothetical protein | 5.4 |
|  | BT4028 | hypothetical protein | 6.2 |
|  | BT4037 | hypothetical protein | 6.4 |
|  | BT4038 | SusC/D paralog | 1.6 |
|  | BT4040 | putative galactose oxidase precursor | 1.5 |
|  | BT4056 | conserved hypothetical protein | 2.4 |
|  | BT4133 | hypothetical protein | 1.3 |
|  | BT4145 | conserved hypothetical protein | 1.8 |
|  | BT4172 | conserved hypothetical protein | 1.3 |
|  | BT4223 | hypothetical protein | 2.8 |
|  | BT4226 | hypothetical protein | 2.9 |
|  | BT4227 | hypothetical protein | 1.9 |
|  | BT4268 | SusC/D paralog | 2.2 |
|  | BT4269 | hypothetical protein | 5.3 |
|  | BT4270 | hypothetical protein | 2.1 |
|  | BT4377 | hypothetical protein | 1.3 |
|  | BT4382 | hypothetical protein | 2.2 |
|  | BT4475 | hypothetical protein | 2.3 |
|  | BT4497 | conserved hypothetical protein | 2.2 |
|  | BT4619 | hypothetical protein | 5.0 |
|  | BT4655 | conserved hypothetical protein | 4.0 |
|  | BT4659 | SusC/D paralog | 3.1 |
|  | BT4669 | hypothetical protein | 1.4 |
|  | BT4675 | heparin lyase I precursor | 3.8 |
|  | BT4710 | conserved hypothetical protein | 1.5 |
|  | BT4742 | tetracycline resistance element regulator RteB | 2.4 |
|  | BT4759 | putative mobilization protein | 1.4 |
|  | BT4761 | conserved protein found in conjugate transposon | 13.3 |
|  | BT4765 | conserved hypothetical protein | 2.2 |
|  | BT4766 | hypothetical protein | 1.9 |
|  | BT4767 | conserved protein found in conjugate transposon | 2.8 |
|  | BT4768 | conserved protein found in conjugate transposon | 2.8 |
|  | BT4769 | conserved protein found in conjugate transposon | 1.6 |
|  | BT4771 | hypothetical protein | 5.3 |
|  | BT4772 | conserved protein found in conjugate transposon | 1.7 |
|  | BT4773 | conserved protein found in conjugate transposon | 2.3 |
|  | BT4774 | conserved protein found in conjugate transposon | 7.2 |
|  | BT4775 | conserved protein found in conjugate transposon | 2.0 |
