# Supplementary material for: Genomic and Metabolic Studies of the Impact of Probiotics on a Model Gut Symbiont and Host
Source: PLoS Biol. 2006 Nov 28;4(12):e413. doi: 10.1371/journal.pbio.0040413 (PMC1661682; doi:10.1371/journal.pbio.0040413)
Supplement: Supplementary file 18 [file pbio.0040413.st009.doc]

**Table S9.** COG functional group-based categorization of *B. thetaiotaomicron* genes that show decreased

expression upon co-colonization with *B. longum*.

| **COG function** | **Gene** | **Description** | **Fold lower expression in co-colonization (vs. mono-association)** |
| --- | --- | --- | --- |
| Translation, ribosomal structure and biogenesis |  |  |  |
|  | BT0239 | hypothetical protein | 1.2 |
|  | BT0810 | hypothetical protein | 1.8 |
|  | BT0811 | putative tRNA/rRNA methyltransferase | 1.2 |
|  | BT0893 | tRNA (Guanine-N(1)-methyltransferase | 1.4 |
|  | BT1336 | putative translation initiation inhibitor | 1.3 |
|  | BT1454 | putative peptide chain release factor | 1.9 |
|  | BT1500 | Ribonuclease G | 1.4 |
|  | BT1840 | histidyl-tRNA synthetase | 1.1 |
|  | BT1975 | tRNA nucleotidyltransferase | 1.4 |
|  | BT2786 | 30S ribosomal protein S15 | 1.3 |
|  | BT3141 | hypothetical protein | 1.5 |
|  | BT3772 | ribosomal large subunit pseudouridine synthase D | 1.7 |
|  | BT3879 | putative translation initiation factor SUI1 | 2.0 |
|  | BT4429 | putative pteridine-dependent dioxygenase | 1.3 |
|  | BT4513 | putative rRNA methylase | 1.2 |
|  | BT4776 | conserved protein found in conjugate transposon | 5.6 |
|  |  |  |  |
| Transcription |  |  |  |
|  | BT0121 | transcriptional regulator, ArsR family | 2.6 |
|  | BT0188 | RNA polymerase ECF-type sigma factor | 2.0 |
|  | BT0248 | RNA polymerase ECF-type sigma factor | 1.7 |
|  | BT0326 | putative RNA polymerase ECF-type sigma factor | 5.3 |
|  | BT0507 | transcriptional regulator (TetR/AcrR family) | 2.2 |
|  | BT0559 | transcriptional regulator | 1.5 |
|  | BT0581 | putative acetyltransferase, GNAT family | 1.5 |
|  | BT0582 | conserved hypothetical protein | 2.0 |
|  | BT0583 | hypothetical protein | 1.3 |
|  | BT0596 | putative transcriptional regulator | 3.2 |
|  | BT0636 | putative transcriptional regulator | 1.5 |
|  | BT0728 | transcriptional regulator | 1.7 |
|  | BT0812 | putative transcriptional regulator | 2.4 |
|  | BT0890 | hypothetical protein | 1.9 |
|  | BT0966 | RNA polymerase ECF-type sigma factor | 2.5 |
|  | BT1078 | putative transcriptional regulator | 1.5 |
|  | BT1090 | AraC-type transcription regulator | 1.4 |
|  | BT1130 | hypothetical protein | 1.5 |
|  | BT1135 | hypothetical protein | 1.4 |
|  | BT1189 | conserved hypothetical protein | 1.5 |
|  | BT1258 | putative acetyltransferase | 1.5 |
|  | BT1265 | two-component system response regulator | 1.7 |
|  | BT1304 | putative transcriptional regulator | 1.4 |
|  | BT1319 | two-component system response regulator | 1.8 |
|  | BT1396 | transcription regulator, AraC family | 1.4 |
|  | BT1464 | putative acetyltransferase, (GNAT) family | 1.4 |
|  | BT1559 | RNA polymerase ECF-type sigma factor | 1.6 |
|  | BT1568 | conserved hypothetical protein | 1.8 |
|  | BT1572 | RNA polymerase ECF-type sigma factor | 1.7 |
|  | BT1587 | conserved hypothetical protein | 1.4 |
|  | BT1877 | RNA polymerase ECF-type sigma factor | 1.3 |
|  | BT1963 | putative transcriptional regulator | 1.4 |
|  | BT1964 | hypothetical protein | 2.4 |
|  | BT1984 | transcriptional regulator | 1.5 |
|  | BT2044 | RNA polymerase ECF-type sigma factor | 1.8 |
|  | BT2049 | transcriptional regulator | 1.8 |
|  | BT2051 | conserved hypothetical protein | 1.5 |
|  | BT2096 | putative transcriptional regulator | 1.6 |
|  | BT2103 | transcriptional regulator | 1.7 |
|  | BT2116 | conserved hypothetical protein | 1.5 |
|  | BT2164 | MarR family transcriptional regulator | 1.2 |
|  | BT2198 | RNA polymerase ECF-type sigma factor | 3.1 |
|  | BT2226 | transcriptional regulator | 1.3 |
|  | BT2310 | conserved hypothetical protein | 9.1 |
|  | BT2320 | transcription regulator | 9.4 |
|  | BT2360 | transcriptional regulator | 1.5 |
|  | BT2372 | transcriptional regulator | 2.4 |
|  | BT2435 | MarR family transcriptional regulator | 1.8 |
|  | BT2463 | RNA polymerase ECF-type sigma factor | 1.6 |
|  | BT2521 | RNA polymerase sigma-54 | 1.2 |
|  | BT2546 | transcriptional regulator | 1.7 |
|  | BT2554 | hypothetical protein | 1.6 |
|  | BT2562 | putative RNA polymerase ECF-type sigma factor | 1.9 |
|  | BT2565 | transcription elongation factor greA | 1.4 |
|  | BT2689 | conserved hypothetical protein | 2.6 |
|  | BT2783 | transcriptional regulator | 1.6 |
|  | BT2792 | AraC/XylS family transcriptional regulator | 4.3 |
|  | BT2957 | transcriptional regulator | 1.3 |
|  | BT2966 | hypothetical protein | 1.5 |
|  | BT3010 | RNA polymerase ECF-type sigma factor | 1.4 |
|  | BT3052 | transcriptional regulator | 1.2 |
|  | BT3152 | ATP-dependent DNA helicase | 1.8 |
|  | BT3153 | hypothetical protein | 2.0 |
|  | BT3269 | RNA polymerase ECF-type sigma factor | 1.6 |
|  | BT3277 | RNA polymerase ECF-type sigma factor | 1.9 |
|  | BT3517 | RNA polymerase ECF-type sigma factor | 2.1 |
|  | BT3556 | transcriptional regulator | 1.5 |
|  | BT3667 | transcriptional regulator | 2.2 |
|  | BT3770 | transcriptional regulator | 1.7 |
|  | BT3899 | transcriptional regulator | 2.2 |
|  | BT3974 | conserved hypothetical protein | 1.9 |
|  | BT3997 | transcriptional regulator | 1.5 |
|  | BT4198 | conserved hypothetical protein | 1.3 |
|  | BT4232 | hypothetical protein | 1.5 |
|  | BT4250 | RNA polymerase ECF-type sigma factor | 1.6 |
|  | BT4355 | RNA polymerase ECF-type sigma factor | 2.1 |
|  | BT4418 | hypothetical protein | 1.6 |
|  | BT4515 | hypothetical protein | 2.3 |
|  | BT4529 | hypothetical protein | 1.7 |
|  | BT4534 | hypothetical protein | 1.9 |
|  | BT4571 | RNA polymerase ECF-type sigma factor | 5.3 |
|  | BT4599 | putative transcriptional regulator | 1.6 |
|  | BT4641 | putative acetyltransferase | 1.6 |
|  | BT4643 | RNA polymerase ECF-type sigma factor | 3.6 |
|  | BT4696 | conserved hypothetical protein | 1.6 |
|  | BT4716 | redox-sensitive transcriptional activator | 1.8 |
|  | BT4720 | putative RNA polymerase ECF-type sigma factor | 2.0 |
|  | BT4733 | conserved hypothetical protein | 1.6 |
|  |  |  |  |
| Replication, recombination and repair |  |  |  |
|  | BT0023 | transposase | 2.2 |
|  | BT0036 | integrase | 1.3 |
|  | BT0070 | conserved hypothetical protein | 1.5 |
|  | BT0078 | putative DNA repair protein | 2.0 |
|  | BT0281 | putative DNA-binding protein | 2.4 |
|  | BT0419 | putative endonuclease | 1.4 |
|  | BT0615 | conserved hypothetical protein | 1.8 |
|  | BT0657 | ATP-dependent DNA helicase | 1.2 |
|  | BT0707 | conserved hypothetical protein | 10.2 |
|  | BT0953 | integrase | 1.7 |
|  | BT1124 | putative integrase | 4.1 |
|  | BT1125 | hypothetical protein | 1.8 |
|  | BT1154 | ATP-independent RNA helicase | 1.7 |
|  | BT1188 | putative transcription regulator | 1.8 |
|  | BT1411 | methylated-DNA--protein-cysteinemethyltransferase | 2.0 |
|  | BT1424 | hypothetical protein | 1.6 |
|  | BT1431 | putative DNA polymerase III epsilon chain | 1.2 |
|  | BT1497 | single-strand binding protein (SSB) | 1.3 |
|  | BT1503 | integrase | 2.0 |
|  | BT1513 | integrase | 1.9 |
|  | BT1517 | conserved hypothetical protein | 1.9 |
|  | BT1664 | crossover junction endodeoxyribonuclease ruvC | 1.7 |
|  | BT1753 | putative methylated-DNA methyltransferase | 1.4 |
|  | BT1793 | integrase protein | 1.9 |
|  | BT1845 | conserved hypothetical protein | 1.4 |
|  | BT1978 | Holliday junction DNA helicase ruvA | 1.5 |
|  | BT1980 | transposase | 1.6 |
|  | BT2236 | conserved hypothetical protein | 2.0 |
|  | BT2321 | putative integrase | 2.9 |
|  | BT2400 | DNA-3-methyladenine glycosylase I | 2.8 |
|  | BT2447 | integrase | 4.6 |
|  | BT2484 | hypothetical protein | 1.8 |
|  | BT2617 | reverse transcriptase | 2.5 |
|  | BT2634 | putative integrase/recombinase | 1.3 |
|  | BT2635 | putative integrase/recombinase | 2.4 |
|  | BT2830 | DNA-binding protein HU-beta | 1.4 |
|  | BT2843 | integrase | 1.2 |
|  | BT2986 | integrase | 1.6 |
|  | BT3033 | DNA topoisomerase IV subunit B | 2.1 |
|  | BT3167 | conserved hypothetical protein | 5.6 |
|  | BT3199 | putative non-specific DNA-binding protein | 1.5 |
|  | BT3412 | ribonuclease HII | 1.5 |
|  | BT3478 | integrase | 1.8 |
|  | BT3557 | putative transposase | 1.6 |
|  | BT3715 | hypothetical protein | 1.4 |
|  | BT3891 | hypothetical protein | 1.5 |
|  | BT4010 | conserved hypothetical protein | 1.4 |
|  | BT4011 | DNA-methyltransferase | 1.6 |
|  | BT4022 | integrase | 2.5 |
|  | BT4231 | hypothetical protein | 1.8 |
|  | BT4255 | DNA replication and repair protein RecF, ABC family ATPase | 1.2 |
|  | BT4342 | RNA-directed DNA polymerase | 1.7 |
|  | BT4409 | DNA damage-inducible protein | 1.2 |
|  | BT4437 | transposase | 1.4 |
|  | BT4488 | formamidopyrimidine-DNA glycosylase | 1.4 |
|  | BT4603 | conserved hypothetical protein | 1.3 |
|  | BT4651 | endonuclease IV | 1.5 |
|  |  |  |  |
| Cell cycle control, cell division, chromosome partitioning |  |  |  |
|  | BT0519 | hypothetical protein | 1.3 |
|  | BT1144 | conserved hypothetical protein | 1.4 |
|  | BT1593 | putative cell-cycle protein | 1.2 |
|  | BT2526 | hypothetical protein | 1.5 |
|  | BT3166 | conserved hypothetical protein | 2.1 |
|  | BT3421 | hypothetical protein | 1.4 |
|  | BT3437 | hypothetical protein | 2.7 |
|  | BT3445 | cell division protein FtsA | 1.3 |
|  | BT3621 | amidase enhancer precursor | 1.7 |
|  | BT4222 | conserved hypothetical protein | 1.6 |
|  |  |  |  |
| Defense mechanisms |  |  |  |
|  | BT0694 | ABC transporter, ATP-binding protein | 3.7 |
|  | BT0695 | ABC transporter, permease protein | 3.2 |
|  | BT0957 | conserved hypothetical protein | 1.4 |
|  | BT1215 | ABC transporter, permease protein | 3.1 |
|  | BT1268 | transporter, AcrB/D/F family | 1.4 |
|  | BT1306 | putative ABC transporter ATP-binding protein | 6.6 |
|  | BT1532 | ABC transporter permease | 3.2 |
|  | BT1533 | conserved hypothetical protein | 3.0 |
|  | BT1535 | ABC transporter ATP-binding protein | 3.5 |
|  | BT1931 | putative type IIS restriction/modification enzyme | 1.4 |
|  | BT2241 | DNA-damage-inducible protein F | 2.2 |
|  | BT2494 | ABC transporter, ATP-binding protein | 2.2 |
|  | BT2495 | ABC transporter, putative permease | 1.3 |
|  | BT2794 | putative multidrug resistance protein | 1.9 |
|  | BT3151 | hypothetical protein | 1.6 |
|  | BT3200 | putative N-acetylmuramoyl-L-alanine amidase | 1.9 |
|  | BT3640 | lipoprotein releasing system ATP-binding protein | 1.6 |
|  | BT3903 | conserved hypothetical protein | 1.8 |
|  | BT4318 | ATP-binding protein | 1.7 |
|  | BT4408 | hypothetical protein | 1.6 |
|  | BT4522 | Type I restriction-modification enzyme | 1.9 |
|  | BT4523 | Type I restriction enzyme EcoR124II specificity protein | 2.8 |
|  | BT4535 | Type I restriction enzyme EcoR124II R protein | 1.9 |
|  | BT4540 | putative Type I restriction enzyme MjaXP specificity protein | 2.9 |
|  | BT4625 | DNA modification methylase BstVI | 1.6 |
|  |  |  |  |
| Signal transduction mechanisms |  |  |  |
|  | BT0156 | transcriptional regulator | 1.2 |
|  | BT0267 | Hybrid two-component system | 1.3 |
|  | BT0324 | hypothetical protein | 7.6 |
|  | BT0520 | transcriptional regulator | 1.3 |
|  | BT0682 | two-component system response regulator | 1.6 |
|  | BT0689 | putative two-component system sensor histidine kinase | 1.3 |
|  | BT0690 | two-component system response regulator | 1.4 |
|  | BT0700 | GTP pyrophosphokinase | 1.7 |
|  | BT0729 | transcriptional regulator | 1.7 |
|  | BT0733 | two-component system sensor histidine kinase | 1.3 |
|  | BT0807 | DnaK suppressor protein, putative | 1.4 |
|  | BT0901 | putative universal stress protein UspA | 1.5 |
|  | BT0927 | two-component system sensor histidine kinase | 1.7 |
|  | BT0928 | two-component system response regulator | 3.6 |
|  | BT0994 | two-component system histidine kinase | 3.7 |
|  | BT0998 | two-component system sensor histidine kinase | 2.0 |
|  | BT1075 | putative two-component system sensor protein, but no histidine kinase domain | 2.8 |
|  | BT1117 | conserved hypothetical protein, with a cAMP-binding domain | 1.4 |
|  | BT1123 | transcriptional regulator | 2.7 |
|  | BT1216 | putative two-component system sensor protein, without kinase domain | 2.2 |
|  | BT1234 | conserved hypothetical protein | 1.6 |
|  | BT1318 | signaling protein without kinase domain | 1.5 |
|  | BT1334 | PhoH-like protein | 1.8 |
|  | BT1413 | putative transcription regulator | 1.6 |
|  | BT1459 | putative two-component system sensor histidine kinase, no kinase domain | 1.3 |
|  | BT1469 | two-component system response regulator | 1.9 |
|  | BT1482 | two-component system response regulator | 1.3 |
|  | BT1529 | two-component system response regulator | 2.1 |
|  | BT1531 | two-component system sensor histidine kinase | 1.4 |
|  | BT1742 | carbon starvation protein A | 1.6 |
|  | BT1795 | putative two-component system sensor protein, no kinase domain | 4.1 |
|  | BT1888 | transcriptional regulator, LuxR family | 1.7 |
|  | BT2165 | two-component system response regulator | 1.2 |
|  | BT2166 | two-component system sensor histidine kinase | 1.6 |
|  | BT2181 | transcriptional regulator | 1.6 |
|  | BT2187 | putative cAMP-binding domain-containing regulatory protein | 1.6 |
|  | BT2366 | conserved hypothetical protein | 2.0 |
|  | BT2421 | FrrC, osmosensitive K+ channel histidine kinase | 1.9 |
|  | BT2433 | two-component system response regulator | 1.2 |
|  | BT3097 | Hybrid two-component system | 1.3 |
|  | BT3134 | Hybrid two-component system | 1.3 |
|  | BT3150 | transcriptional regulator | 1.7 |
|  | BT3172 | Hybrid two-component system | 1.3 |
|  | BT3304 | conserved hypothetical protein | 1.7 |
|  | BT3316 | hypothetical protein | 5.1 |
|  | BT3334 | Hybrid two-component system | 1.4 |
|  | BT3543 | hypothetical protein | 1.5 |
|  | BT3801 | rteB, two-component system response regulator | 2.0 |
|  | BT3805 | two-component system sensor histidine kinase | 1.4 |
|  | BT4057 | two-component system sensor histidine kinase | 1.4 |
|  | BT4178 | Hybrid two-component system | 1.6 |
|  | BT4300 | transcription regulator, CRP family | 1.8 |
|  |  |  |  |
| Cell wall/membrane/envelope biogenesis |  |  |  |
|  | BT0056 | putative glycosyltransferase | 2.1 |
|  | BT0060 | putative polysaccharide export protein | 1.8 |
|  | BT0251 | dolichol-phosphate mannosyltransferase | 1.5 |
|  | BT0381 | capsular polysaccharide biosynthesis protein capD | 1.8 |
|  | BT0382 | capsular polysaccharide biosynthesis protein Cps4K | 1.3 |
|  | BT0384 | hypothetical protein | 1.6 |
|  | BT0471 | glycosyltransferase | 1.6 |
|  | BT0476 | D-mannose-1-phosphate guanyltransferase | 1.9 |
|  | BT0481 | polysaccharide export outer membrane protein | 1.5 |
|  | BT0560 | outer membrane efflux protein | 1.4 |
|  | BT0561 | conserved hypothetical protein, putative membrane protein | 2.0 |
|  | BT0598 | putative nucleoside-diphosphate sugar epimerases/dehydrase | 1.7 |
|  | BT0599 | UDP-glucose 6-dehydrogenase | 2.1 |
|  | BT0601 | UDP-N-acetylglucosamine 2-epimerase | 1.6 |
|  | BT0602 | UDP-N-acetyl-D-mannosaminuronic acid dehydrogenase | 1.5 |
|  | BT0608 | glycosyltransferase | 6.4 |
|  | BT0610 | lipopolysaccharide biosynthesis protein, putative glycosyltransferase | 1.9 |
|  | BT0611 | glycosyltransferase | 5.3 |
|  | BT0613 | putative membrane protein involved in polysaccharide export | 3.0 |
|  | BT0623 | putative UDP-glucose 4-epimerase | 1.2 |
|  | BT0632 | GTP-binding protein, membrane GTPase lepA | 1.2 |
|  | BT0813 | TonB | 1.7 |
|  | BT0817 | lipoprotein, function unknown | 1.7 |
|  | BT1212 | outer membrane efflux protein | 4.0 |
|  | BT1213 | periplasmic component of efflux system | 3.1 |
|  | BT1225 | GDP-fucose synthetase | 1.3 |
|  | BT1346 | capsule biosynthesis protein capA | 2.0 |
|  | BT1348 | CDP-abequose synthase | 1.5 |
|  | BT1353 | glycosyltransferase | 1.7 |
|  | BT1530 | putative outer membrane protein OprM precursor | 4.8 |
|  | BT1536 | ABC transporter permease | 5.9 |
|  | BT1623 | putative membrane protein | 2.2 |
|  | BT1716 | putative teichoic acid biosynthesis protein B precursor | 2.5 |
|  | BT1740 | hypothetical protein | 1.7 |
|  | BT1791 | outer membrane protein | 1.5 |
|  | BT2038 | cation efflux system protein | 1.8 |
|  | BT2040 | hypothetical protein | 1.4 |
|  | BT2117 | outer membrane protein, putative | 1.4 |
|  | BT2133 | conserved hypothetical protein | 1.6 |
|  | BT2497 | membrane fusion efflux protein | 1.6 |
|  | BT2665 | TonB | 1.4 |
|  | BT2687 | cation efflux system protein | 1.9 |
|  | BT2688 | outer membrane efflux protein | 1.8 |
|  | BT2762 | TonB | 1.6 |
|  | BT2798 | carboxy-terminal processing protease precursor | 1.5 |
|  | BT2874 | putative colanic acid biosynthesis glycosyltransferase | 1.8 |
|  | BT2879 | conserved hypothetical protein | 3.4 |
|  | BT2888 | glycosyltransferase | 4.5 |
|  | BT2927 | conserved hypothetical protein, putative cell wall-associated protein precursor | 1.5 |
|  | BT3018 | putative mechano-sensitive ion channel | 3.7 |
|  | BT3207 | hypothetical protein | 2.4 |
|  | BT3362 | heptosyltransferase | 1.3 |
|  | BT3366 | putative glycosyltransferase yibD | 2.0 |
|  | BT3368 | putative glycosyltransferase | 1.9 |
|  | BT3379 | putative glycosyltransferase | 1.4 |
|  | BT3450 | UDP-N-acetylmuramoylalanine--D-glutamate ligase | 1.3 |
|  | BT3451 | phospho-N-acetylmuramoyl-pentapeptide-transferase | 1.7 |
|  | BT3453 | penicillin-binding protein | 1.2 |
|  | BT3455 | S-adenosyl-methyltransferase mraW | 1.5 |
|  | BT3622 | putative glycosyltransferase | 1.3 |
|  | BT3722 | glutamate racemase | 1.3 |
|  | BT3724 | cationic outer membrane protein precursor | 2.0 |
|  | BT3807 | conserved hypothetical protein | 2.0 |
|  | BT3904 | conserved hypothetical protein | 1.7 |
|  | BT3905 | putative alkaline protease aprF | 2.1 |
|  | BT4335 | conserved hypothetical protein | 2.2 |
|  | BT4642 | conserved hypothetical protein | 2.6 |
|  | BT4693 | cation efflux system protein | 2.1 |
|  |  |  |  |
| Cell motility |  |  |  |
|  | BT3413 | hypothetical protein | 2.5 |
|  |  |  |  |
| Intracellular trafficking, secretion, and vesicular transport |  |  |  |
|  | BT1072 | hypothetical protein | 13.1 |
|  | BT1227 | conserved hypothetical protein | 1.4 |
|  | BT2270 | integral membrane protein (MarC family) | 1.5 |
|  | BT3319 | signal peptidase I | 1.5 |
|  | BT3398 | conserved hypothetical protein | 2.2 |
|  | BT4301 | conserved hypothetical protein, putative membrane protein | 1.5 |
|  | BT4622 | mobilization protein BmgA | 1.5 |
|  | BT4758 | putative mobilization protein | 5.9 |
|  |  |  |  |
| Posttranslational modification, protein turnover, chaperones |  |  |  |
|  | BT0165 | peptidyl-prolyl cis-trans isomerase | 1.7 |
|  | BT0166 | putative thiol-disulfide isomerase | 1.3 |
|  | BT0219 | thioredoxin C-2 | 2.0 |
|  | BT0781 | hypothetical protein | 1.5 |
|  | BT0833 | conserved hypothetical protein | 1.5 |
|  | BT0987 | putative cytochrome C-type biogenesis protein | 6.8 |
|  | BT1415 | cytochrome c biogenesis protein ccsA | 1.6 |
|  | BT2564 | conserved hypothetical protein | 1.4 |
|  | BT2745 | thiol:disulfide interchange protein | 1.9 |
|  | BT2845 | conserved hypothetical protein | 1.4 |
|  | BT3072 | collagenase precursor | 1.5 |
|  | BT3418 | putative thiol:disulfide interchange protein | 2.4 |
|  | BT3521 | alpha-1,6-mannanase | 1.7 |
|  | BT3612 | putative FKBP-type peptidyl-prolyl cis-trans isomerase fkpA | 1.6 |
|  | BT3802 | peptidyl-prolyl cis-trans isomerase, cyclophilin-type | 1.5 |
|  | BT4194 | putative integral membrane protein | 2.2 |
|  | BT4602 | chaperone protein dnaK | 3.3 |
|  |  |  |  |
| Energy production and conversion |  |  |  |
|  | BT0125 | NADH:ubiquinone oxidoreductase subunit | 1.4 |
|  | BT0181 | putative NADH dehydrogenase/NAD(P)H nitroreductase | 1.4 |
|  | BT0217 | conserved hypothetical protein | 2.5 |
|  | BT0604 | putative coenzyme F420-reducing hydrogenase | 1.9 |
|  | BT1148 | putative NADH dehydrogenase/NAD(P)H nitroreductase | 1.6 |
|  | BT1270 | putative Na+/H+ antiporter | 1.2 |
|  | BT1575 | putative dehydrogenase | 1.9 |
|  | BT1680 | putative NADH dehydrogenase/NAD(P)H nitroreductase | 1.4 |
|  | BT2144 | putative oxidoreductase | 1.4 |
|  | BT2414 | ferredoxin | 1.3 |
|  | BT2539 | rubredoxin | 1.2 |
|  | BT2924 | acetyl-CoA synthetase | 1.4 |
|  | BT3223 | hypothetical protein | 174.4 |
|  | BT3341 | conserved hypothetical protein | 1.6 |
|  | BT3626 | hypothetical protein | 2.1 |
|  | BT4657 | heparinase III protein | 1.5 |
|  |  |  |  |
| Carbohydrate transport and metabolism |  |  |  |
|  | BT0290 | beta-galactosidase precursor | 1.9 |
|  | BT0348 | alpha-L-arabinofuranosidase | 1.4 |
|  | BT0443 | thermostable beta-glucosidase B | 1.5 |
|  | BT0487 | transcriptional regulator | 1.3 |
|  | BT0676 | N-acetylglucosamine-6-phosphate deacetylase | 2.0 |
|  | BT0959 | conserved hypothetical protein | 2.1 |
|  | BT1034 | putative signal transducer | 5.2 |
|  | BT1051 | beta-hexosaminidase precursor | 1.7 |
|  | BT1527 | hypothetical protein | 1.6 |
|  | BT1621 | beta-hexosaminidase precursor | 1.4 |
|  | BT1768 | beta-galactosidase | 2.8 |
|  | BT1972 | phosphoenolpyruvate synthase/pyruvate phosphate dikinase | 1.2 |
|  | BT2000 | putative transport protein | 1.6 |
|  | BT2104 | ROK family member transcriptional repressor | 1.4 |
|  | BT2105 | putative alpha-1,2-mannosidase | 1.5 |
|  | BT2146 | 4-alpha-glucanotransferase | 1.9 |
|  | BT2150 | methylglyoxal synthase | 1.8 |
|  | BT2439 | beta-N-acetylglucosaminidase | 1.5 |
|  | BT2506 | hypothetical protein | 2.7 |
|  | BT2631 | putative hydrolase | 1.8 |
|  | BT2799 | putative PfkB family carbohydrate kinase | 1.6 |
|  | BT2852 | putative beta-xylosidase | 1.6 |
|  | BT2855 | beta-mannosidase precursor | 1.5 |
|  | BT2970 | alpha-L-fucosidase precursor | 1.4 |
|  | BT3085 | alpha-xylosidase | 1.6 |
|  | BT3133 | alpha-galactosidase | 1.6 |
|  | BT3173 | conserved hypothetical protein | 1.6 |
|  | BT3217 | hypothetical protein | 2.3 |
|  | BT3527 | alpha-1,2-mannosidase | 1.3 |
|  | BT3588 | N-acetylglucosamine-6-phosphate deacetylase | 1.8 |
|  | BT3606 | sugar-proton symporter | 1.4 |
|  | BT3620 | AmpG protein, beta-lactamase induction signal transducer | 1.6 |
|  | BT3673 | TonB | 2.0 |
|  | BT3774 | alpha-mannosidase | 1.3 |
|  | BT3963 | putative sugar hydrolase | 1.8 |
|  | BT4051 | conserved hypothetical protein | 1.3 |
|  | BT4072 | alpha-mannosidase | 1.5 |
|  | BT4136 | conserved hypothetical protein | 1.9 |
|  | BT4156 | beta-galactosidase | 2.1 |
|  | BT4337 | beta-hexosaminidase precursor | 1.5 |
|  | BT4406 | hypothetical protein | 2.4 |
|  | BT4653 | glucose/galactose transporter | 2.0 |
|  | BT4684 | beta-galactosidase | 1.4 |
|  |  |  |  |
| Amino acid transport and metabolism |  |  |  |
|  | BT0249 | 5-methyltetrahydrofolate--homocysteine methyltransferase or methionine synthase | 1.3 |
|  | BT0469 | hypothetical protein | 1.7 |
|  | BT0477 | putative phosphatase | 2.5 |
|  | BT1260 | putative peptidase | 1.4 |
|  | BT1743 | hypothetical protein | 1.4 |
|  | BT1815 | low-specificity L-threonine aldolase | 2.2 |
|  | BT1974 | metallopeptidase family M24, putative Xaa-Pro dipeptidase | 1.6 |
|  | BT2404 | L-asparaginase I | 1.5 |
|  | BT2522 | Xaa-Pro aminopeptidase | 1.4 |
|  | BT2683 | putative periplasmic protein | 1.9 |
|  | BT2685 | hypothetical protein | 1.6 |
|  | BT2694 | putative urocanate hydratase | 1.4 |
|  | BT3029 | Na+/glucose symporter | 2.5 |
|  | BT3161 | hypothetical protein | 1.3 |
|  | BT3320 | dihydrodipicolinate reductase | 1.5 |
|  | BT3549 | acetylornithine deacetylase | 1.3 |
|  | BT3608 | conserved hypothetical protein | 1.9 |
|  | BT3975 | 3-dehydroquinate synthase | 1.6 |
|  | BT4096 | conserved hypothetical protein | 1.4 |
|  | BT4150 | putative rhamnogalacturonan acetylesterase | 1.3 |
|  | BT4193 | dipeptidyl peptidase IV | 1.4 |
|  | BT4360 | hypothetical protein | 1.4 |
|  | BT4421 | conserved hypothetical protein | 1.8 |
|  | BT4502 | conserved hypothetical protein | 1.5 |
|  | BT4512 | putative zinc-type alcohol dehydrogenase | 1.5 |
|  | BT4678 | L-serine dehydratase | 1.4 |
|  |  |  |  |
| Nucleotide transport and metabolism |  |  |  |
|  | BT0881 | hypothetical protein | 1.2 |
|  | BT0888 | AMP nucleosidase | 1.6 |
|  | BT1539 | cytidine deaminase | 1.4 |
|  | BT1588 | conserved hypothetical protein | 2.2 |
|  | BT1843 | adenylosuccinate synthetase | 1.4 |
|  | BT2060 | cytidylate kinase | 1.3 |
|  | BT2093 | hypothetical protein | 1.8 |
|  | BT2440 | 5'-nucleotidase precursor | 1.3 |
|  | BT2441 | putative 5'-nucleotidase/2',3'-cyclic phosphodiesterase | 1.4 |
|  | BT2527 | uridine kinase (uridine monophosphokinase) | 1.5 |
|  | BT2566 | HIT family protein | 1.4 |
|  | BT2815 | putative mutT family protein | 1.5 |
|  | BT2883 | phosphoribosylglycinamide formyltransferase | 2.1 |
|  | BT3360 | phosphoribosylglycinamide formyltransferase | 2.9 |
|  | BT3461 | deoxyuridine 5'-triphosphate nucleotidohydrolase | 1.6 |
|  | BT3563 | putative endonuclease precursor | 1.4 |
|  | BT4586 | conserved hypothetical protein | 1.4 |
|  |  |  |  |
| Coenzyme transport and metabolism |  |  |  |
|  | BT0310 | lipoate-protein ligase A | 1.6 |
|  | BT0495 | cobalamin biosynthesis protein | 1.5 |
|  | BT0923 | putative periplasmic protein | 6.9 |
|  | BT1089 | lipoate-protein ligase B | 1.6 |
|  | BT1317 | riboflavin synthase alpha chain | 1.5 |
|  | BT1849 | hypothetical protein | 1.6 |
|  | BT2058 | putative isoprenyl synthetase | 1.3 |
|  | BT2149 | dihydroneopterin aldolase | 1.7 |
|  | BT2396 | putative membrane transporter involved in nicotinamide mononucleotide transport | 1.6 |
|  | BT2779 | 3-demethylubiquinone-9 3-methyltransferase | 1.8 |
|  | BT2905 | SusC/D paralog | 2.6 |
|  | BT2968 | SusC/D paralog | 1.6 |
|  | BT3728 | riboflavin biosynthesis protein ribD | 1.3 |
|  | BT4258 | putative 5-formyltetrahydrofolate cyclo-ligase | 1.5 |
|  | BT4374 | 4-hydroxythreonine-4-phosphate dehydrogenase | 1.5 |
|  | BT4453 | 6-pyruvoyl-tetrahydropterin synthase | 1.3 |
|  | BT4563 | hypothetical protein | 3.2 |
|  |  |  |  |
| Lipid transport and metabolism |  |  |  |
|  | BT0135 | conserved hypothetical protein | 1.8 |
|  | BT0243 | putative 1-acyl-sn-glycerol-3-phosphate acyltransferase | 1.7 |
|  | BT0260 | acyltransferase family protein | 1.4 |
|  | BT0316 | putative hemin receptor | 1.4 |
|  | BT1170 | hypothetical protein | 3.2 |
|  | BT1574 | putative outer membrane protein precursor | 1.5 |
|  | BT1854 | putative membrane-associated phospholipid phosphatase | 1.5 |
|  | BT2002 | 1-deoxy-D-xylulose 5-phosphate reductoisomerase | 1.5 |
|  | BT2114 | hypothetical protein | 1.8 |
|  | BT2231 | phosphatidylserine decarboxylase | 1.4 |
|  | BT2382 | putative cardiolipin synthetase | 3.9 |
|  | BT2520 | conserved hypothetical protein | 1.4 |
|  | BT3084 | lipase, putative | 1.4 |
|  | BT3100 | lipase, putative esterase | 1.7 |
|  | BT3976 | hypothetical protein | 1.3 |
|  | BT4239 | hypothetical protein | 1.3 |
|  | BT4322 | conserved hypothetical protein, with a diacylglycerol kinase catalytic domain | 1.3 |
|  |  |  |  |
| Inorganic ion transport and metabolism |  |  |  |
|  | BT0150 | putative ferric aerobactin receptor | 2.3 |
|  | BT0189 | conserved hypothetical protein, putative anti-sigma factor | 2.2 |
|  | BT0215 | iron uptake regulatory protein | 1.4 |
|  | BT0411 | CysQ, sulfite synthesis pathway protein | 1.3 |
|  | BT0499 | cation efflux system protein | 1.8 |
|  | BT0965 | putative anti-sigma factor | 1.9 |
|  | BT1118 | SusC/D paralog | 2.0 |
|  | BT1119 | SusC/D paralog | 3.6 |
|  | BT1141 | cation efflux family protein | 9.3 |
|  | BT1279 | putative anti-sigma factor | 1.4 |
|  | BT1420 | conserved hypothetical protein | 4.5 |
|  | BT1618 | putative anti-sigma factor | 2.0 |
|  | BT1783 | cation/H+ antiporter | 1.8 |
|  | BT1875 | SusC/D paralog | 1.5 |
|  | BT1876 | putative anti-sigma factor | 3.6 |
|  | BT2019 | polyphosphate kinase | 1.4 |
|  | BT2032 | SusC/D paralog | 2.4 |
|  | BT2039 | cation efflux system protein, AcrB/AcrD/AcrF family protein | 4.0 |
|  | BT2100 | putative ABC transporter, ATP-binding protein | 1.2 |
|  | BT2107 | SusC/D paralog | 2.7 |
|  | BT2171 | putative anti-sigma factor | 4.0 |
|  | BT2172 | SusC/D paralog | 1.4 |
|  | BT2197 | putative anti-sigma factor | 2.0 |
|  | BT2362 | SusC/D paralog | 1.7 |
|  | BT2423 | K+-transporting ATPase C chain | 2.6 |
|  | BT2424 | K+-transporting ATPase B chain | 1.7 |
|  | BT2461 | SusC/D paralog | 2.4 |
|  | BT2462 | putative anti-sigma factor | 2.9 |
|  | BT2479 | iron-regulated protein A precursor | 1.9 |
|  | BT2511 | putative transcription regulator | 3.7 |
|  | BT2561 | putative anti-sigma factor | 2.0 |
|  | BT2572 | putative potassium channel subunit | 1.5 |
|  | BT2664 | putative phosphate ABC transporter, phosphate-binding component | 5.9 |
|  | BT2788 | chromate transport protein | 1.3 |
|  | BT2789 | chromate transport protein | 1.5 |
|  | BT3011 | putative anti-sigma factor | 2.1 |
|  | BT3271 | SusC/D paralog | 1.6 |
|  | BT3297 | SusC/D paralog | 1.5 |
|  | BT3493 | SusC/D paralog | 1.4 |
|  | BT3749 | putative anti-sigma factor | 8.6 |
|  | BT3750 | SusC/D paralog | 1.9 |
|  | BT3944 | putative chloride channel protein | 3.1 |
|  | BT3977 | conserved hypothetical protein | 2.0 |
|  | BT4090 | SusC/D paralog | 1.7 |
|  | BT4248 | putative anti-sigma factor | 1.7 |
|  | BT4352 | hypothetical protein | 1.9 |
|  | BT4356 | putative anti-sigma factor | 1.7 |
|  | BT4403 | putative anti-sigma factor | 3.2 |
|  | BT4404 | SusC/D paralog | 1.7 |
|  | BT4631 | putative arylsulfatase precursor | 1.5 |
|  | BT4634 | SusC/D paralog | 1.5 |
|  | BT4635 | putative anti-sigma factor | 2.5 |
|  | BT4650 | voltage-gated chloride channel protein | 2.7 |
|  |  |  |  |
| Secondary metabolites biosynthesis, transport and catabolism |  |  |  |
|  | BT0199 | conserved hypothetical protein | 1.3 |
|  | BT1015 | putative oxidoreductase | 1.6 |
|  | BT1855 | putative methyl transferase | 1.4 |
|  | BT4261 | hypothetical protein | 1.3 |
|  |  |  |  |
| General function prediction only |  |  |  |
|  | BT0031 | conserved hypothetical protein | 1.4 |
|  | BT0164 | conserved hypothetical protein | 1.5 |
|  | BT0168 | conserved hypothetical protein | 1.7 |
|  | BT0176 | conserved hypothetical protein | 1.4 |
|  | BT0240 | conserved hypothetical protein | 1.9 |
|  | BT0259 | putative amidohydrolase | 1.7 |
|  | BT0335 | putative ATPase | 1.2 |
|  | BT0660 | conserved hypothetical protein | 2.3 |
|  | BT0763 | conserved hypothetical protein | 2.1 |
|  | BT0879 | ABC transporter, permease protein | 1.9 |
|  | BT0910 | magnesium chelatase, subunit I | 1.5 |
|  | BT0917 | competence-damage inducible protein | 1.3 |
|  | BT0962 | aldo/keto reductase | 1.3 |
|  | BT0969 | hypothetical protein | 1.5 |
|  | BT1016 | conserved protein with a conserved patatin-like phospholipase domain | 1.6 |
|  | BT1190 | putative ATP GTP-binding protein | 1.7 |
|  | BT1247 | hypothetical protein | 1.6 |
|  | BT1257 | conserved hypothetical protein | 1.3 |
|  | BT1347 | conserved hypothetical protein | 1.5 |
|  | BT1354 | putative flippase | 1.7 |
|  | BT1412 | iron-sulfur flavoprotein | 1.7 |
|  | BT1666 | hydrolase, haloacid dehalogenase-like hydrolase | 1.3 |
|  | BT1690 | TPR-domain containing protein | 1.3 |
|  | BT1707 | conserved hypothetical protein | 1.6 |
|  | BT1813 | conserved hypothetical protein, putative integral membrane protein | 1.7 |
|  | BT1894 | TPR-repeat-containing proteins | 1.8 |
|  | BT1924 | putative metal-dependent hydrolase | 1.7 |
|  | BT2012 | conserved hypothetical protein, putative ATPase | 1.5 |
|  | BT2018 | conserved hypothetical protein | 1.5 |
|  | BT2174 | ABC transporter ATP-binding protein | 1.3 |
|  | BT2188 | putative oxidoreductase | 1.4 |
|  | BT2273 | methyltransferase | 1.4 |
|  | BT2278 | conserved hypothetical protein | 1.7 |
|  | BT2343 | hypothetical protein | 2.2 |
|  | BT2371 | putative protease/amidase | 1.4 |
|  | BT2385 | conserved hypothetical protein | 1.6 |
|  | BT2405 | conserved hypothetical protein | 1.7 |
|  | BT2749 | putative membrane-associated HD superfamily hydrolase | 1.7 |
|  | BT2756 | anaerobic C4-dicarboxylate transporter dcuB | 1.5 |
|  | BT2769 | putative serine/threonine-protein kinase pknB | 1.6 |
|  | BT2775 | putative helicase | 3.8 |
|  | BT2832 | rhomboid family protein | 1.6 |
|  | BT2840 | O-methyltransferase | 1.3 |
|  | BT2943 | putative hexapeptide transferase family protein | 2.4 |
|  | BT3023 | conserved hypothetical protein | 1.5 |
|  | BT3042 | conserved hypothetical protein | 1.4 |
|  | BT3220 | TPR-repeat-containing protein | 1.6 |
|  | BT3224 | putative lysine decarboxylase | 11.2 |
|  | BT3286 | conserved hypothetical protein | 1.8 |
|  | BT3352 | haloacid dehalogenase-like hydrolase | 2.1 |
|  | BT3422 | hypothetical protein | 3.3 |
|  | BT3432 | conserved hypothetical protein | 1.4 |
|  | BT3459 | hemolysin A | 1.6 |
|  | BT3472 | putative hydrogenase | 1.5 |
|  | BT3509 | conserved hypothetical protein | 1.4 |
|  | BT3515 | putatuve glycosylhydrolase | 1.4 |
|  | BT3585 | putative oxidoreductase | 1.3 |
|  | BT3586 | putative dehydrogenase | 1.7 |
|  | BT3696 | conserved hypothetical protein | 1.7 |
|  | BT3705 | regulatory protein SusR | 1.7 |
|  | BT3837 | ABC transporter ATP-binding protein | 1.3 |
|  | BT3922 | putative ThiJ family intracellular protease/amidase | 1.6 |
|  | BT3942 | conserved hypothetical protein | 1.4 |
|  | BT4003 | stationary-phase survival protein SurE | 1.2 |
|  | BT4140 | hypothetical protein | 1.8 |
|  | BT4214 | conserved hypothetical protein | 1.5 |
|  | BT4252 | putative oxidoreductase | 1.4 |
|  | BT4256 | conserved hypothetical protein | 1.4 |
|  | BT4273 | conserved hypothetical protein | 2.1 |
|  | BT4302 | conserved hypothetical protein | 1.6 |
|  | BT4339 | glutamine synthetase | 3.7 |
|  | BT4350 | conserved hypothetical protein | 1.4 |
|  | BT4465 | conserved hypothetical protein, putative outer membrane protein | 7.8 |
|  | BT4482 | conserved hypothetical protein | 2.6 |
|  | BT4485 | conserved hypothetical protein | 1.3 |
|  | BT4496 | putative oxidoreductase | 1.3 |
|  | BT4510 | putative helicase | 2.0 |
|  | BT4526 | conserved hypothetical protein | 2.5 |
|  | BT4539 | conserved hypothetical protein | 1.3 |
|  | BT4551 | putative GTPase, ThdF family | 2.2 |
|  | BT4557 | hypothetical protein | 1.2 |
|  | BT4616 | conserved hypothetical protein | 1.7 |
|  | BT4697 | transcriptional regulator | 17.7 |
|  |  |  |  |
| Function unknown |  |  |  |
|  | BT0167 | conserved hypothetical protein | 1.7 |
|  | BT0170 | conserved hypothetical protein | 2.0 |
|  | BT0284 | putative peptidoglycan binding protein (LPXTG motif) | 2.5 |
|  | BT0776 | conserved hypothetical protein | 1.3 |
|  | BT0882 | conserved hypothetical protein | 1.6 |
|  | BT0975 | conserved hypothetical protein, putative integral membrane protein | 1.2 |
|  | BT1004 | conserved hypothetical protein | 1.3 |
|  | BT1061 | hypothetical protein | 2.7 |
|  | BT1086 | putative oligopeptide transporter | 1.5 |
|  | BT1150 | conserved hypothetical protein | 2.5 |
|  | BT1201 | conserved hypothetical protein | 1.9 |
|  | BT1256 | conserved hypothetical protein | 1.8 |
|  | BT1330 | conserved hypothetical protein | 1.3 |
|  | BT1393 | 4-carboxymuconolactone decarboxylase | 1.4 |
|  | BT1405 | conserved hypothetical protein | 1.9 |
|  | BT1422 | conserved hypothetical protein | 2.0 |
|  | BT1423 | conserved hypothetical protein | 1.3 |
|  | BT1494 | conserved hypothetical protein | 1.6 |
|  | BT1510 | conserved hypothetical protein | 1.6 |
|  | BT1562 | conserved hypothetical protein | 1.2 |
|  | BT1567 | conserved hypothetical protein | 1.8 |
|  | BT1823 | conserved hypothetical protein, putative membrane protein | 1.8 |
|  | BT1826 | conserved hypothetical protein | 1.3 |
|  | BT1831 | conserved hypothetical protein | 1.5 |
|  | BT1897 | conserved hypothetical protein | 1.7 |
|  | BT2036 | conserved hypothetical protein | 1.7 |
|  | BT2068 | 3-oxo-5-alpha-steroid 4-dehydrogenase | 1.6 |
|  | BT2080 | conserved hypothetical protein, putative inner membrane protein | 1.6 |
|  | BT2095 | putative surface layer protein | 1.5 |
|  | BT2097 | conserved hypothetical protein | 1.6 |
|  | BT2177 | putative membrane protein | 3.3 |
|  | BT2182 | conserved hypothetical protein | 1.2 |
|  | BT2210 | conserved hypothetical protein | 2.2 |
|  | BT2237 | conserved hypothetical protein | 1.4 |
|  | BT2282 | conserved hypothetical protein | 19.1 |
|  | BT2505 | conserved hypothetical protein | 1.3 |
|  | BT2513 | conserved hypothetical protein | 2.0 |
|  | BT2606 | conserved hypothetical protein | 2.2 |
|  | BT2760 | conserved hypothetical protein | 1.6 |
|  | BT3030 | conserved hypothetical protein with conserved domains | 1.6 |
|  | BT3047 | hypothetical protein | 1.7 |
|  | BT3180 | conserved hypothetical protein | 1.5 |
|  | BT3251 | conserved hypothetical protein, putative integral membrane protein | 4.0 |
|  | BT3378 | conserved hypothetical protein | 1.8 |
|  | BT3390 | conserved hypothetical protein | 2.4 |
|  | BT3410 | conserved hypothetical protein | 3.4 |
|  | BT3554 | conserved hypothetical protein | 1.4 |
|  | BT3643 | conserved hypothetical protein | 1.4 |
|  | BT3650 | conserved hypothetical protein | 1.9 |
|  | BT3697 | UDP-2,3-diacylglucosamine hydrolase | 1.6 |
|  | BT3869 | conserved hypothetical protein | 1.5 |
|  | BT4177 | conserved hypothetical protein | 2.3 |
|  | BT4195 | conserved hypothetical protein | 1.7 |
|  | BT4212 | conserved hypothetical protein | 1.6 |
|  | BT4427 | putative surface layer protein, with cytochrome c domain | 1.5 |
|  | BT4459 | conserved hypothetical protein, putative transport protein | 1.4 |
|  | BT4516 | conserved hypothetical protein | 1.6 |
|  | BT4537 | conserved hypothetical protein | 1.2 |
|  | BT4607 | conserved hypothetical protein | 2.0 |
|  | BT4732 | conserved hypothetical protein | 2.2 |
|  |  |  |  |
| Not in COG |  |  |  |
|  | BT0003 | hypothetical protein | 2.6 |
|  | BT0006 | hypothetical protein | 2.0 |
|  | BT0035 | hypothetical protein | 4.6 |
|  | BT0042 | conserved hypothetical protein | 2.2 |
|  | BT0062 | hypothetical protein | 2.9 |
|  | BT0064 | hypothetical protein | 2.7 |
|  | BT0067 | hypothetical protein | 4.5 |
|  | BT0074 | hypothetical protein | 1.3 |
|  | BT0077 | conserved hypothetical protein | 1.4 |
|  | BT0082 | hypothetical protein | 1.8 |
|  | BT0083 | conserved hypothetical protein | 1.7 |
|  | BT0084 | conserved protein found in conjugate transposon | 1.6 |
|  | BT0094 | conserved protein found in conjugate transposon | 1.6 |
|  | BT0107 | conserved hypothetical protein | 4.0 |
|  | BT0126 | hypothetical protein | 2.0 |
|  | BT0151 | hypothetical protein | 1.7 |
|  | BT0153 | hypothetical protein | 1.4 |
|  | BT0175 | conserved hypothetical protein | 1.3 |
|  | BT0177 | hypothetical protein | 1.3 |
|  | BT0231 | excisionase | 1.4 |
|  | BT0253 | hypothetical protein | 1.4 |
|  | BT0255 | hypothetical protein | 1.7 |
|  | BT0263 | hypothetical protein | 1.4 |
|  | BT0325 | hypothetical protein | 4.5 |
|  | BT0377 | conserved hypothetical protein | 1.4 |
|  | BT0397 | conserved hypothetical protein | 2.4 |
|  | BT0444 | hypothetical protein | 1.5 |
|  | BT0445 | endoglucanase E precursor (EGE) | 2.0 |
|  | BT0450 | conserved hypothetical protein | 1.8 |
|  | BT0484 | SusC/D paralog | 1.7 |
|  | BT0503 | hypothetical protein | 1.8 |
|  | BT0511 | hypothetical protein | 1.5 |
|  | BT0514 | conserved hypothetical protein | 1.9 |
|  | BT0586 | hypothetical protein | 4.3 |
|  | BT0591 | hypothetical protein | 1.8 |
|  | BT0592 | conserved hypothetical protein | 2.0 |
|  | BT0594 | hypothetical protein | 1.6 |
|  | BT0597 | conserved hypothetical protein | 6.1 |
|  | BT0603 | conserved hypothetical protein | 2.0 |
|  | BT0641 | hypothetical protein | 1.9 |
|  | BT0646 | hypothetical protein | 2.1 |
|  | BT0658 | hypothetical protein | 1.5 |
|  | BT0683 | alpha-glucosidase | 2.0 |
|  | BT0708 | hypothetical protein | 2.0 |
|  | BT0731 | hypothetical protein | 1.6 |
|  | BT0759 | hypothetical protein | 1.6 |
|  | BT0764 | hypothetical protein | 2.1 |
|  | BT0777 | hypothetical protein | 1.4 |
|  | BT0826 | hypothetical protein | 1.5 |
|  | BT0827 | hypothetical protein | 1.3 |
|  | BT0848 | hypothetical protein | 1.5 |
|  | BT0868 | hypothetical protein | 1.5 |
|  | BT0904 | BatD, conserved hypothetical protein | 1.7 |
|  | BT0922 | hypothetical protein | 5.1 |
|  | BT0932 | hypothetical protein | 2.4 |
|  | BT0933 | hypothetical protein | 1.4 |
|  | BT0968 | conserved hypothetical protein | 1.4 |
|  | BT0971 | conserved hypothetical protein | 2.5 |
|  | BT0977 | hypothetical protein | 1.4 |
|  | BT1010 | conserved hypothetical protein | 1.4 |
|  | BT1030 | hypothetical protein | 3.2 |
|  | BT1035 | conserved hypothetical protein | 1.8 |
|  | BT1036 | hypothetical protein | 1.5 |
|  | BT1043 | hypothetical protein | 1.4 |
|  | BT1044 | hypothetical protein | 1.6 |
|  | BT1049 | putative patatin-like protein | 1.6 |
|  | BT1050 | hypothetical protein | 1.8 |
|  | BT1073 | hypothetical protein | 9.3 |
|  | BT1074 | hypothetical protein | 27.2 |
|  | BT1097 | hypothetical protein | 3.0 |
|  | BT1111 | hypothetical protein | 1.4 |
|  | BT1112 | transposase | 2.2 |
|  | BT1120 | hypothetical protein | 1.9 |
|  | BT1140 | hypothetical protein | 1.6 |
|  | BT1142 | hypothetical protein | 9.1 |
|  | BT1143 | conserved hypothetical protein | 1.4 |
|  | BT1169 | hypothetical protein | 1.7 |
|  | BT1178 | conserved hypothetical protein | 1.6 |
|  | BT1191 | hypothetical protein | 1.9 |
|  | BT1230 | hypothetical protein | 12.8 |
|  | BT1231 | hypothetical protein | 3.8 |
|  | BT1233 | hypothetical protein | 1.7 |
|  | BT1240 | conserved hypothetical protein | 2.8 |
|  | BT1255 | hypothetical protein | 1.6 |
|  | BT1282 | hypothetical protein | 1.6 |
|  | BT1283 | conserved hypothetical protein | 2.2 |
|  | BT1284 | putative endo-beta-N-acetylglucosaminidase F1 precursor (mannosyl-glycoprotein endo-beta-N-acetyl-glucosaminidase F1) | 1.7 |
|  | BT1285 | endo-beta-N-acetylglucosaminidase | 2.6 |
|  | BT1421 | hypothetical protein | 1.6 |
|  | BT1480 | hypothetical protein | 1.2 |
|  | BT1484 | conserved hypothetical protein with a conserved domain | 1.5 |
|  | BT1534 | hypothetical protein | 5.5 |
|  | BT1556 | hypothetical protein | 5.6 |
|  | BT1557 | hypothetical protein | 1.5 |
|  | BT1558 | hypothetical protein | 1.7 |
|  | BT1569 | conserved hypothetical protein | 1.5 |
|  | BT1581 | hypothetical protein | 1.7 |
|  | BT1585 | hypothetical protein | 1.5 |
|  | BT1586 | hypothetical protein | 1.4 |
|  | BT1590 | hypothetical protein | 2.1 |
|  | BT1665 | hypothetical protein | 1.5 |
|  | BT1667 | hypothetical protein | 1.4 |
|  | BT1700 | conserved hypothetical protein | 1.7 |
|  | BT1741 | hypothetical protein | 1.7 |
|  | BT1777 | conserved hypothetical protein | 2.4 |
|  | BT1788 | hypothetical protein | 2.3 |
|  | BT1790 | hypothetical protein | 1.7 |
|  | BT1792 | putative cell surface protein | 1.8 |
|  | BT1798 | hypothetical protein | 2.4 |
|  | BT1818 | hypothetical protein | 1.6 |
|  | BT1828 | conserved protein, with a weak D-galactarate dehydratase/altronate hydrolase domain | 1.8 |
|  | BT1856 | hypothetical protein | 1.4 |
|  | BT1903 | hypothetical protein | 2.6 |
|  | BT1958 | hypothetical protein | 2.1 |
|  | BT1961 | transposase | 2.6 |
|  | BT1981 | hypothetical protein | 1.8 |
|  | BT1982 | hypothetical protein | 1.9 |
|  | BT1985 | hypothetical protein | 2.1 |
|  | BT1994 | hypothetical protein | 1.5 |
|  | BT2022 | hypothetical protein | 2.0 |
|  | BT2030 | conserved hypothetical protein | 1.3 |
|  | BT2037 | hypothetical protein | 1.6 |
|  | BT2043 | hypothetical protein | 3.2 |
|  | BT2050 | conserved hypothetical protein | 2.8 |
|  | BT2087 | hypothetical protein | 1.6 |
|  | BT2109 | hypothetical protein | 1.6 |
|  | BT2170 | hypothetical protein | 6.1 |
|  | BT2173 | hypothetical protein | 2.2 |
|  | BT2195 | SusC/D paralog | 1.5 |
|  | BT2218 | hypothetical protein | 1.5 |
|  | BT2235 | hypothetical protein | 1.6 |
|  | BT2247 | putative ryanodine receptor | 1.7 |
|  | BT2261 | hypothetical protein | 1.7 |
|  | BT2262 | hypothetical protein | 1.8 |
|  | BT2265 | hypothetical protein | 1.4 |
|  | BT2274 | hypothetical protein | 1.4 |
|  | BT2314 | hypothetical protein | 6.8 |
|  | BT2317 | hypothetical protein | 1.8 |
|  | BT2341 | conserved hypothetical protein | 1.6 |
|  | BT2354 | conserved hypothetical protein | 1.5 |
|  | BT2358 | hypothetical protein | 1.5 |
|  | BT2361 | hypothetical protein | 1.6 |
|  | BT2369 | hypothetical protein | 1.7 |
|  | BT2388 | hypothetical protein | 1.8 |
|  | BT2418 | hypothetical protein | 1.5 |
|  | BT2422 | conserved hypothetical protein | 1.5 |
|  | BT2449 | hypothetical protein | 1.6 |
|  | BT2472 | hypothetical protein | 1.9 |
|  | BT2473 | hypothetical protein | 16.6 |
|  | BT2490 | hypothetical protein | 1.4 |
|  | BT2491 | hypothetical protein | 2.3 |
|  | BT2500 | hypothetical protein | 8.4 |
|  | BT2502 | conserved hypothetical protein | 6.2 |
|  | BT2503 | hypothetical protein | 8.4 |
|  | BT2504 | hypothetical protein | 7.5 |
|  | BT2507 | hypothetical protein | 1.9 |
|  | BT2534 | hypothetical protein | 1.3 |
|  | BT2579 | conserved hypothetical protein | 1.4 |
|  | BT2583 | hypothetical protein | 2.1 |
|  | BT2605 | hypothetical protein | 7.5 |
|  | BT2612 | hypothetical protein | 1.5 |
|  | BT2647 | hypothetical protein | 1.8 |
|  | BT2669 | hypothetical protein | 1.9 |
|  | BT2677 | hypothetical protein | 1.5 |
|  | BT2678 | hypothetical protein | 4.9 |
|  | BT2695 | hypothetical protein | 1.8 |
|  | BT2696 | hypothetical protein | 5.9 |
|  | BT2698 | hypothetical protein | 1.5 |
|  | BT2699 | hypothetical protein | 2.3 |
|  | BT2754 | conserved hypothetical protein | 1.8 |
|  | BT2766 | hypothetical protein | 3.0 |
|  | BT2776 | hypothetical protein | 1.6 |
|  | BT2809 | putative integral membrane protein | 1.6 |
|  | BT2822 | conserved hypothetical protein | 1.8 |
|  | BT2823 | hypothetical protein | 4.2 |
|  | BT2884 | conserved hypothetical protein | 1.7 |
|  | BT2906 | conserved hypothetical protein | 2.4 |
|  | BT2926 | hypothetical protein | 2.7 |
|  | BT2951 | SusC/D paralog | 77.5 |
|  | BT2958 | conserved hypothetical protein | 1.5 |
|  | BT2962 | hypothetical protein | 5.0 |
|  | BT2965 | hypothetical protein | 1.3 |
|  | BT2981 | hypothetical protein | 1.5 |
|  | BT2987 | hypothetical protein | 1.9 |
|  | BT2988 | hypothetical protein | 1.7 |
|  | BT2993 | hypothetical protein | 1.5 |
|  | BT2997 | hypothetical protein | 2.1 |
|  | BT2998 | conserved hypothetical protein | 1.7 |
|  | BT3004 | conserved hypothetical protein | 1.6 |
|  | BT3013 | hypothetical protein | 1.8 |
|  | BT3039 | hypothetical protein | 1.3 |
|  | BT3098 | hypothetical protein | 1.3 |
|  | BT3140 | conserved hypothetical protein | 1.6 |
|  | BT3142 | mobilization protein BmgB | 1.5 |
|  | BT3149 | hypothetical protein | 1.6 |
|  | BT3155 | conserved hypothetical protein | 1.5 |
|  | BT3160 | hypothetical protein | 1.6 |
|  | BT3170 | hypothetical protein | 1.6 |
|  | BT3183 | hypothetical protein | 1.7 |
|  | BT3185 | hypothetical protein | 2.0 |
|  | BT3188 | hypothetical protein | 5.1 |
|  | BT3189 | hypothetical protein | 4.0 |
|  | BT3201 | hypothetical protein | 1.4 |
|  | BT3203 | hypothetical protein | 1.3 |
|  | BT3205 | hypothetical protein | 1.8 |
|  | BT3206 | hypothetical protein | 1.8 |
|  | BT3218 | hypothetical protein | 1.8 |
|  | BT3221 | hypothetical protein | 92.4 |
|  | BT3222 | hypothetical protein | 121.0 |
|  | BT3225 | hypothetical protein | 15.8 |
|  | BT3234 | hypothetical protein | 2.2 |
|  | BT3274 | hypothetical protein | 3.8 |
|  | BT3276 | conserved hypothetical protein | 1.4 |
|  | BT3281 | hypothetical protein | 1.3 |
|  | BT3315 | hypothetical protein | 3.0 |
|  | BT3324 | chondroitinase (chondroitin lyase) precursor | 5.1 |
|  | BT3335 | hypothetical protein | 1.5 |
|  | BT3342 | hypothetical protein | 1.3 |
|  | BT3381 | hypothetical protein | 1.3 |
|  | BT3382 | hypothetical protein | 2.1 |
|  | BT3383 | hypothetical protein | 1.6 |
|  | BT3384 | hypothetical protein | 1.9 |
|  | BT3389 | hypothetical protein | 1.5 |
|  | BT3400 | hypothetical protein | 2.0 |
|  | BT3417 | hypothetical protein | 1.5 |
|  | BT3428 | hypothetical protein | 2.0 |
|  | BT3433 | hypothetical protein | 1.4 |
|  | BT3435 | hypothetical protein | 2.0 |
|  | BT3436 | hypothetical protein | 2.2 |
|  | BT3438 | hypothetical protein | 2.4 |
|  | BT3454 | hypothetical protein | 1.5 |
|  | BT3477 | glutaminase A | 1.7 |
|  | BT3481 | putative glutaminase A | 3.1 |
|  | BT3491 | glutaminase A | 1.5 |
|  | BT3492 | conserved hypothetical protein, with a conserved domain | 1.5 |
|  | BT3495 | SusC/D paralog | 3.2 |
|  | BT3502 | hypothetical protein | 2.4 |
|  | BT3511 | hypothetical protein | 1.4 |
|  | BT3514 | conserved hypothetical protein | 2.5 |
|  | BT3534 | hypothetical protein | 2.5 |
|  | BT3535 | hypothetical protein | 1.6 |
|  | BT3538 | hypothetical protein | 2.0 |
|  | BT3547 | hypothetical protein | 1.6 |
|  | BT3564 | hypothetical protein | 1.7 |
|  | BT3571 | hypothetical protein | 1.7 |
|  | BT3575 | hypothetical protein | 1.7 |
|  | BT3578 | hypothetical protein | 1.8 |
|  | BT3595 | hypothetical protein | 1.5 |
|  | BT3596 | hypothetical protein | 1.6 |
|  | BT3632 | hypothetical protein | 2.6 |
|  | BT3651 | hypothetical protein | 2.8 |
|  | BT3721 | hypothetical protein | 1.4 |
|  | BT3742 | hypothetical protein | 1.5 |
|  | BT3744 | hypothetical protein | 1.7 |
|  | BT3746 | hypothetical protein | 2.1 |
|  | BT3751 | outer membrane protein | 2.0 |
|  | BT3769 | hypothetical protein | 1.5 |
|  | BT3822 | hypothetical protein | 3.6 |
|  | BT3856 | hypothetical protein | 1.6 |
|  | BT3859 | hypothetical protein | 1.3 |
|  | BT3874 | hypothetical protein | 1.8 |
|  | BT3886 | hypothetical protein | 2.3 |
|  | BT3906 | conserved hypothetical protein | 1.7 |
|  | BT3913 | hypothetical protein | 3.5 |
|  | BT3914 | hypothetical protein | 2.9 |
|  | BT3949 | hypothetical protein | 1.9 |
|  | BT3970 | hypothetical protein | 1.6 |
|  | BT4036 | hypothetical protein | 2.3 |
|  | BT4046 | conserved hypothetical protein | 1.7 |
|  | BT4048 | hypothetical protein | 1.3 |
|  | BT4071 | hypothetical protein | 1.7 |
|  | BT4079 | hypothetical protein | 2.5 |
|  | BT4088 | SusC/D paralog | 2.6 |
|  | BT4100 | hypothetical protein | 1.3 |
|  | BT4116 | conserved hypothetical protein | 4.0 |
|  | BT4132 | putative chitobiase | 1.4 |
|  | BT4141 | hypothetical protein | 2.7 |
|  | BT4158 | hypothetical protein | 4.5 |
|  | BT4161 | hypothetical protein | 1.3 |
|  | BT4180 | acetyl xylan esterase A | 1.6 |
|  | BT4191 | hypothetical protein | 1.7 |
|  | BT4202 | hypothetical protein | 1.7 |
|  | BT4249 | hypothetical protein | 2.1 |
|  | BT4319 | hypothetical protein | 1.5 |
|  | BT4341 | hypothetical protein | 7.3 |
|  | BT4361 | hypothetical protein | 1.6 |
|  | BT4381 | hypothetical protein | 1.5 |
|  | BT4384 | hypothetical protein | 2.6 |
|  | BT4400 | hypothetical protein | 2.5 |
|  | BT4413 | hypothetical protein | 1.5 |
|  | BT4419 | hypothetical protein | 1.3 |
|  | BT4440 | conserved hypothetical protein, putative cell surface protein | 1.6 |
|  | BT4441 | cell surface protein | 2.0 |
|  | BT4466 | hypothetical protein | 2.5 |
|  | BT4467 | hypothetical protein | 2.4 |
|  | BT4478 | hypothetical protein | 2.2 |
|  | BT4490 | hypothetical protein | 1.4 |
|  | BT4508 | hypothetical protein | 1.5 |
|  | BT4525 | hypothetical protein | 1.5 |
|  | BT4532 | hypothetical protein | 1.8 |
|  | BT4547 | excisionase | 1.7 |
|  | BT4569 | hypothetical protein | 4.8 |
|  | BT4570 | hypothetical protein | 4.2 |
|  | BT4575 | hypothetical protein | 1.5 |
|  | BT4596 | hypothetical protein | 1.7 |
|  | BT4600 | conserved hypothetical protein | 1.3 |
|  | BT4604 | hypothetical protein | 1.7 |
|  | BT4606 | hypothetical protein | 1.6 |
|  | BT4632 | putative galactose oxidase precursor | 2.0 |
|  | BT4649 | hypothetical protein | 1.8 |
|  | BT4674 | hypothetical protein | 1.5 |
|  | BT4685 | hypothetical protein | 3.3 |
|  | BT4721 | hypothetical protein | 2.3 |
|  | BT4729 | hypothetical protein | 1.9 |
|  | BT4741 | hypothetical protein | 2.4 |
|  | BT4746 | conserved hypothetical protein | 1.9 |
|  | BT4760 | hypothetical protein | 1.4 |
