# Supplementary material for: Genomic and Metabolic Studies of the Impact of Probiotics on a Model Gut Symbiont and Host
Source: PLoS Biol. 2006 Nov 28;4(12):e413. doi: 10.1371/journal.pbio.0040413 (PMC1661682; doi:10.1371/journal.pbio.0040413)
Supplement: Supplementary file 19 [file pbio.0040413.st010.doc]

**Table S10.** COG functional categorization of *B. thetaiotaomicron* genes with increased expression upon co-colonization with *L. casei* in B6 mice.

| **COG function** | **Gene** | **Description** | **Fold difference in expression (co-colonization versus mono-association)** |
| --- | --- | --- | --- |
| Translation |  |  |  |
|  | BT0865 | putative chitobiase | 2.5 |
|  | BT2730 | 30S ribosomal protein S7 | 1.5 |
|  | BT2731 | 30S ribosomal protein S12 | 1.6 |
| Transcription |  |  |  |
|  | BT4001 | putative chromosome partitioning protein parB | 1.6 |
|  | BT4628 | ATP-dependent helicase | 1.6 |
| Defense mechanisms |  |  |  |
|  | BT3200 | putative N-acetylmuramoyl-L-alanine amidase | 3.1 |
| Cell wall/membrane biogenesis |  |  |  |
|  | BT1370 | NAD dependent nucleotide-diphosphate-sugar epimerase | 1.6 |
|  | BT2016 | dTDP-glucose 4,6-dehydratase | 1.5 |
| Posttranslational modification, protein turnover, chaperones |  |  |  |
|  | BT4194 | putative integral membrane protein | 1.4 |
| Energy production and conversion |  |  |  |
|  | BT2144 | putative oxidoreductase | 1.5 |
|  | BT4738 | formate acetyltransferase | 1.8 |
| Carbohydrate transport and metabolism |  |  |  |
|  | BT0360 | putative arabinan endo-1,5-alpha-L-arabinosidase | 1.8 |
|  | BT0368 | alpha-L-arabinofuranosidase A precursor | 1.5 |
|  | BT0455 | sialidase (Neuraminidase) | 1.4 |
|  | BT0459 | beta-hexosaminidase precursor | 1.5 |
|  | BT0460 | beta-hexosaminidase precursor | 1.5 |
|  | BT0461 | beta-galactosidase | 1.6 |
|  | BT3704 | alpha-amylase (neopullulanase) SusA | 3.5 |
|  | BT4050 | beta-galactosidase | 1.6 |
|  | BT4241 | beta-galactosidase | 1.8 |
|  | BT4311 | glucose/galactose transporter | 1.8 |
|  | BT4337 | beta-hexosaminidase precursor | 1.5 |
|  | BT4668 | arabinogalactan endo-1,4-beta-galactosidase | 1.3 |
| Amino acid transport and metabolism |  |  |  |
|  | BT2570 | glutamate decarboxylase | 1.6 |
|  | BT3394 | putative arginine decarboxylase | 1.6 |
| Inorganic ion transport and metabolism |  |  |  |
|  | BT0362 | SusC/D paralog | 1.8 |
|  | BT0364 | SusC/D paralog | 2.0 |
|  | BT0867 | SusC/D paralog | 2.6 |
|  | BT1025 | SusC/D paralog | 1.8 |
|  | BT1636 | arylsulfatase precursor | 1.5 |
|  | BT2626 | SusC/D paralog | 1.4 |
|  | BT2805 | SusC/D paralog | 10.6 |
|  | BT4247 | SusC/D paralog | 2.9 |
|  | BT4248 | putative anti-sigma factor | 2.5 |
|  | BT4267 | SusC/D paralog | 4.5 |
|  | BT4671 | SusC/D paralog | 1.7 |
| General function prediction only |  |  |  |
|  | BT3023 | hypothetical protein | 1.5 |
|  | BT4243 | putative oxidoreductase (putative secreted protein) | 1.8 |
|  | BT4271 | hypothetical protein | 2.2 |
| Function unknown |  |  |  |
|  | BT4242 | putative transporter | 1.7 |
|  | BT4266 | hypothetical protein | 4.3 |
|  | BT4272 | hypothetical protein | 2.0 |
| not in COGs |  |  |  |
|  | BT0361 | hypothetical protein | 1.8 |
|  | BT0365 | hypothetical protein | 2.0 |
|  | BT0724 | hypothetical protein | 2.8 |
|  | BT0866 | SusC/D paralog | 2.8 |
|  | BT4244 | conserved hypothetical protein | 3.6 |
|  | BT4245 | conserved hypothetical protein | 3.3 |
|  | BT4246 | SusC/D paralog | 2.8 |
|  | BT4268 | SusC/D paralog | 3.9 |
|  | BT4269 | hypothetical protein | 4.2 |
|  | BT4270 | hypothetical protein | 2.9 |
|  | BT4297 | SusC/D paralog | 1.4 |
