# Supplementary material for: Genomic and Metabolic Studies of the Impact of Probiotics on a Model Gut Symbiont and Host
Source: PLoS Biol. 2006 Nov 28;4(12):e413. doi: 10.1371/journal.pbio.0040413 (PMC1661682; doi:10.1371/journal.pbio.0040413)
Supplement: Supplementary file 20 [file pbio.0040413.st011.doc]

**Table S11.** COG functional categorization of *B. thetaiotaomicron* genes with increased expression upon co-colonization with *B. animalis* in B6 mice.

| **COG function** | **Gene** | **Description** | **Fold difference in expression (co-colonization versus mono-association)** |
| --- | --- | --- | --- |
| Translation |  |  |  |
|  | BT0420 | peptide deformylase(PDF) | 1.5 |
| Transcription |  |  |  |
|  | BT1587 | hypothetical protein | 1.4 |
|  | BT2103 | transcriptional regulator | 1.8 |
|  | BT3396 | RNA polymerase ECF-type sigma factor | 1.5 |
|  | BT4250 | RNA polymerase ECF-type sigma factor | 1.5 |
|  | BT4628 | ATP-dependent helicase | 2.0 |
|  | BT4647 | RNA polymerase ECF-type sigma factor | 1.7 |
|  | BT4696 | hypothetical protein | 1.5 |
| Replication, recombination and repair |  |  |  |
|  | BT1980 | transposase | 1.9 |
|  | BT2236 | hypothetical protein | 1.8 |
|  | BT2349 | transposase | 1.9 |
|  | BT3891 | hypothetical protein | 1.4 |
|  | BT4255 | DNA replication and repair protein RecF, ABC family ATPase | 1.4 |
| Intracellular trafficking and secretion |  |  |  |
|  | BT4301 | conserved hypothetical protein, putative membrane protein | 1.7 |
| Posttranslational modification, protein turnover, chaperones |  |  |  |
|  | BT0165 | peptidyl-prolyl cis-trans isomerase | 1.7 |
|  | BT4194 | putative integral membrane protein | 1.5 |
| Energy production and conversion |  |  |  |
|  | BT2551 | phosphate butyryltransferase | 1.6 |
|  | BT2918 | hypothetical protein | 1.6 |
|  | BT3411 | pyrophosphate-energized vacuolar membrane proton pump | 1.4 |
| Carbohydrate transport and metabolism |  |  |  |
|  | BT3356 | 6-phosphofructokinase | 1.4 |
| Amino acid transport and metabolism |  |  |  |
|  | BT1554 | alanine dehydrogenase | 1.5 |
|  | BT2693 | formiminotransferase-cyclodeaminase | 1.4 |
| Nucleotide transport and metabolism |  |  |  |
|  | BT1588 | hypothetical protein | 1.8 |
|  | BT3360 | phosphoribosylglycinamide formyltransferase | 1.6 |
| Lipid transport and metabolism |  |  |  |
|  | BT1170 | hypothetical protein | 1.6 |
| Inorganic ion transport and metabolism |  |  |  |
|  | BT1618 | putative anti-sigma factor | 1.5 |
|  | BT1876 | putative anti-sigma factor | 1.5 |
|  | BT2019 | polyphosphate kinase | 1.7 |
|  | BT4248 | putative anti-sigma factor | 1.8 |
| Secondary metabolites biosynthesis, transport and catabolism |  |  |  |
|  | BT0199 | hypothetical protein | 1.6 |
| General function prediction only |  |  |  |
|  | BT1239 | putative metal-dependent membrane protease | 1.9 |
|  | BT1257 | hypothetical protein | 1.5 |
|  | BT2537 | hypothetical protein | 1.7 |
|  | BT3284 | hypothetical protein | 1.5 |
|  | BT3696 | hypothetical protein | 1.3 |
|  | BT4496 | putative oxidoreductase | 1.6 |
| Function unknown |  |  |  |
|  | BT0284 | putative peptidoglycan binding protein (LPXTG motif) | 2.0 |
|  | BT0542 | hypothetical protein | 1.5 |
|  | BT1256 | hypothetical protein | 1.8 |
|  | BT2237 | hypothetical protein | 1.4 |
|  | BT4349 | hypothetical protein | 1.7 |
| not in COGs |  |  |  |
|  | BT0374 | conserved hypothetical protein | 1.4 |
|  | BT0819 | hypothetical protein | 1.5 |
|  | BT1178 | conserved hypothetical protein | 1.5 |
|  | BT1200 | hypothetical protein | 1.5 |
|  | BT1366 | hypothetical protein | 1.5 |
|  | BT1569 | conserved hypothetical protein | 1.6 |
|  | BT1699 | conserved hypothetical protein | 1.6 |
|  | BT1856 | hypothetical protein | 1.4 |
|  | BT1994 | hypothetical protein | 1.6 |
|  | BT2125 | hypothetical protein | 1.5 |
|  | BT2281 | hypothetical protein | 1.6 |
|  | BT2695 | hypothetical protein | 1.8 |
|  | BT4036 | hypothetical protein | 1.9 |
|  | BT4196 | hypothetical protein | 1.8 |
|  | BT4235 | hypothetical protein | 1.5 |
|  | BT4467 | hypothetical protein | 1.5 |
|  | BT4498 | hypothetical protein | 1.7 |
