# Supplementary material for: Genomic and Metabolic Studies of the Impact of Probiotics on a Model Gut Symbiont and Host
Source: PLoS Biol. 2006 Nov 28;4(12):e413. doi: 10.1371/journal.pbio.0040413 (PMC1661682; doi:10.1371/journal.pbio.0040413)
Supplement: Supplementary file 21 [file pbio.0040413.st012.doc]

Table S12. Genes enriched^a^ in Ingenuity functional categories for host epithelial responses to colonization.

| ***B. thetaiotaomicron* monoassociation** | | ***B. thetaiotaomicron/B. longum* bi-association** | | | |
| --- | --- | --- | --- | --- | --- |
| small molecule biochemistry | immune response | immune response | cell death | cell morphology | cell-to-cell signaling and interaction |
| AKR1B10 | CCL6 | ADA | ADA | ADA | ADAM8 |
| AKR1C3 | Ly6a | CAPG | CCNA2 | CAPG | CLDN1 |
| CTE1 | PGLYRP1 | FOXO3A | FOXO3A | CCNA2 | Ly6a |
| CYBA | PRAP1 | Ifi47 | G1P2 | CLDN1 | MARCKSL1 |
| Cyp4a10 | REG3A (PAP) | Ly6a | Ly6a | FOXO3A | MUC4 |
| FABP1 | SOCS3 | REG3A (PAP) | SKIL | MAL | REG3A (PAP) |
| PDK4 | TGM2 | SOCS3 | SOCS3 | MARCKSL1 | TGM2 |
| Rdh6 | VNN1 | TGM2 | TGM2 | SOCS3 | TIFA |
| RETNLB |  | VNN1 |  |  |  |
| SOCS3 |  |  |  |  |  |
| TGM2 |  |  |  |  |  |
| USF1 |  |  |  |  |  |

^a^Enrichment determined by p<0.05 using a right-tailed Fisher’s exact test. Only categories with ≥8 genes are listed.
