# Supplementary material for: Genomic and Metabolic Studies of the Impact of Probiotics on a Model Gut Symbiont and Host
Source: PLoS Biol. 2006 Nov 28;4(12):e413. doi: 10.1371/journal.pbio.0040413 (PMC1661682; doi:10.1371/journal.pbio.0040413)
Supplement: Supplementary file 22 [file pbio.0040413.st013.doc]

**Table S13**. Nutrient information^a^ for B&K Universal autoclaved mouse chow diet.

|  | per 100g | |
| --- | --- | --- |
| Calories | 374 | kcal |
| Calories from Fat | 60 | kcal |
| Fat | 6.6 | g |
| Saturated Fat | 0.1 | g |
| Protein | 21.7 | g |
| Carbohydrates^b^ | 57 | g |
| Sugar^c^ | 2.0 | g |
| Total Dietary Fiber | 14.7 | g |
| Ash | 5.7 | g |
| Moisture | 9.2 | g |
|  |  |  |
| Cholesterol | 15 | mg |
| Sodium | 386.4 | mg |
| Calcium | 907.4 | mg |
| Potassium | 752.7 | mg |
| Iron | 18.2 | mg |
| Vitamin A | 2000 | IU |
| Vitamin C | 1.2 | mg |

^a^Based on the Official Methods of Analysis of the Association of Official Analytic Chemists (AOAC), 17^th^ edition.

^b^Based on the Atwater method: Carbohydrate = 100 – (% fat + % protein + % moisture + % ash)

^c^Fructose, Glucose, Lactose, Maltose, and Sucrose
