# Supplementary material for: Genomic and Metabolic Studies of the Impact of Probiotics on a Model Gut Symbiont and Host
Source: PLoS Biol. 2006 Nov 28;4(12):e413. doi: 10.1371/journal.pbio.0040413 (PMC1661682; doi:10.1371/journal.pbio.0040413)
Supplement: Supplementary file 23 [file pbio.0040413.st014.doc]

Table S14. *B. thetaiotaomicron* genes used for aggregate signal determination.

| glucose | galactose | mannose | fructose | hexosamine | arabinose | xylose |
| --- | --- | --- | --- | --- | --- | --- |
| BT0771 | BT0065 | BT3774 | BT1760 | BT0438 | BT0360 | BT3085 |
| BT0773 | BT2662 | BT4072 | BT1765 | BT3590 | BT0367 | BT3169 |
| BT1663 | BT3065 | BT1883 | BT3082 | BT4359 | BT1021 | BT3659 |
| BT4689 | BT3592 | BT2623 |  | BT1044 | BT1873 | BT3026 |
| BT4690 | BT4157 | BT2631 |  | BT1048 | BT2900 | BT0145 |
| BT3698 | BT2851 | BT2949 |  | BT1285 | BT3516 | BT0369 |
| BT0339 | BT3131 | BT3301 |  | BT1632 | BT3655 | BT2852 |
| BT3086 | BT4152 | BT3501 |  | BT2825 | BT0348 | BT2895 |
| BT4305 | BT0461 | BT3521 |  | BT3987 | BT0368 | BT2898 |
| BT0132 | BT0757 | BT3524 |  | BT4709 | BT3096 | BT3094 |
| BT0683 | BT0983 | BT3782 |  | BT0456 | BT3657 | BT3108 |
| BT1871 | BT0992 | BT3792 |  | BT0459 | BT2892 | BT3656 |
| BT2620 | BT0993 | BT1032 |  | BT0460 |  | BT3662 |
| BT3163 | BT0996 | BT1769 |  | BT0506 |  | BT3663 |
| BT3294 | BT1626 | BT1878 |  | BT1051 |  | BT3675 |
| BT3661 | BT1768 | BT2105 |  | BT1621 |  | BT4095 |
| BT3664 | BT1776 | BT2111 |  | BT1627 |  | BT3043 |
| BT4581 | BT2190 | BT2199 |  | BT2459 |  |  |
| BT3703 | BT2191 | BT2629 |  | BT3178 |  |  |
| BT2550 | BT2680 | BT2948 |  | BT3598 |  |  |
| BT2824 | BT2922 | BT3130 |  | BT4337 |  |  |
| BT3683 | BT2969 | BT3527 |  | BT4394 |  |  |
| BT1778 | BT3092 | BT3530 |  | BT4681 |  |  |
| BT1780 | BT3114 | BT3773 |  | BT2439 |  |  |
| BT1872 | BT3179 | BT3784 |  | BT4395 |  |  |
| BT2854 | BT3292 | BT3858 |  |  |  |  |
| BT3009 | BT3293 | BT3963 |  |  |  |  |
| BT3300 | BT3340 | BT3965 |  |  |  |  |
| BT3314 | BT3513 | BT3990 |  |  |  |  |
| BT3567 | BT4050 | BT3994 |  |  |  |  |
| BT4714 | BT4156 | BT4073 |  |  |  |  |
| BT3312 | BT4181 | BT4092 |  |  |  |  |
|  | BT4241 | BT4093 |  |  |  |  |
|  | BT4667 | BT0458 |  |  |  |  |
|  | BT4684 | BT2432 |  |  |  |  |
|  | BT0290 | BT2855 |  |  |  |  |
|  | BT4160 | BT3599 |  |  |  |  |
|  | BT4668 | BT4074 |  |  |  |  |
