# Supplementary material for: Genomic and Metabolic Studies of the Impact of Probiotics on a Model Gut Symbiont and Host
Source: PLoS Biol. 2006 Nov 28;4(12):e413. doi: 10.1371/journal.pbio.0040413 (PMC1661682; doi:10.1371/journal.pbio.0040413)
Supplement: Supplementary file 24 [file pbio.0040413.st015.doc]

Table S15. *B. longum* genes used for aggregate signal determination.

| glucose | galactose | mannose | fructose | hexosamine | arabinose | xylose |
| --- | --- | --- | --- | --- | --- | --- |
| BL0388 | BL0978 | BL1327 | BL0105 | BL0056 | BL0146 | BL0523 |
| BL0529 | BL0177 | BL1328 |  | BL1031 | BL0182 | BL0420 |
| BL0536 | BL1518 | BL1329 |  | BL1335 | BL0183 | BL1543 |
| BL0907 | BL0259 |  |  |  | BL0184 | BL1544 |
| BL0982 | BL1168 |  |  |  | BL0187 |  |
| BL0999 |  |  |  |  | BL0181 |  |
| BL1526 |  |  |  |  | BL0544 |  |
| BL1573 |  |  |  |  | BL1138 |  |
| BL1757 |  |  |  |  | BL1166 |  |
| BL1763 |  |  |  |  | BL1611 |  |
| BL0542 |  |  |  |  | BL0257 |  |
| BL1761 |  |  |  |  |  |  |
